# Genome-wide analysis of small RNAs reveals eight fiber elongation-related and 257 novel microRNAs in elongating cotton fiber cells

**DOI:** 10.1186/1471-2164-14-629

**Published:** 2013-09-17

**Authors:** Wei Xue, Zhengming Wang, Mingjian Du, Yidi Liu, Jin-Yuan Liu

**Affiliations:** 1 Laboratory of Molecular Biology, MOE Key Laboratory of Bioinformatics, School of Life Sciences, Tsinghua University, Beijing 100084, China

**Keywords:** Cotton, Comparative miRNAome analysis, Fiber cell elongation, High-throughput sequencing, miRNAs, tasiRNA

## Abstract

**Background:**

MicroRNAs (miRNAs) and other types of small regulatory RNAs play critical roles in the regulation of gene expression at the post-transcriptional level in plants. Cotton is one of the most economically important crops, but little is known about the roles of miRNAs during cotton fiber elongation.

**Results:**

Here, we combined high-throughput sequencing with computational analysis to identify small RNAs (sRNAs) related to cotton fiber elongation in *Gossypium hirsutum* L. (*G. hirsutum*). The sequence analysis confirmed the expression of 79 known miRNA families in elongating fiber cells and identified 257 novel miRNAs, primarily derived from corresponding specific loci in the *Gossypium raimondii* Ulbr. (*G. raimondii*) genome. Furthermore, a comparison of the miRNAomes revealed that 46 miRNA families were differentially expressed throughout the elongation period. Importantly, the predicted and experimentally validated targets of eight miRNAs were associated with fiber elongation, with obvious functional relationships with calcium and auxin signal transduction, fatty acid metabolism, anthocyanin synthesis and the xylem tissue differentiation. Moreover, one tasiRNA was also identified, and its target, ARF4, was experimentally validated *in vivo*.

**Conclusion:**

This study not only facilitated the discovery of 257 novel low-abundance miRNAs in elongating cotton fiber cells but also revealed a potential regulatory network of nine sRNAs important for fiber elongation. The identification and characterization of miRNAs in elongating cotton fiber cells might promote the further study of fiber miRNA regulation mechanisms and provide insight into the importance of miRNAs in cotton.

## Background

Cotton (*Gossypium hirsutum* L.) is one of the most economically important crops and provides the largest renewable source of material for the textile industry. Cotton fiber is a single-cell trichome derived from the epidermal cells of the ovule, and its morphogenesis is comprised of four overlapping developmental stages: initiation, elongation, secondary wall thickening, and maturation [1]. After initiation on the day of anthesis, single-celled fibers undergo rapid elongation (approximately 15 days) and secondary wall deposition (approximately 20 days), followed by maturation into spinnable fibers [2]. One of the most significant features in fiber development is that the rate of fiber elongation and the length of the mature fiber are much greater than those commonly observed for plant cells; the cotton fiber is likely the fastest growing and the longest single cell in higher plants [3,4]. Thus, the cotton fiber is an excellent model in which to study the molecular mechanisms of plant cell elongation without the interference of cell division and multicellular development. Over the past decade, a number of studies have reported that several important proteins, such as sucrose/K^+^ transporters [5], sucrose synthase [6], vacuolar invertase [7], kinesin-like calmodulin-binding protein [8] and calcium dependent protein kinase [9], play critical roles in the process of rapid fiber cell elongation. In addition to calcium signal transduction, the important second messenger molecule H_2_O_2_ might function as a termination signal in cotton fiber cell elongation [10]. Furthermore, recent pioneering experiments revealed that plant hormones play important roles in fiber elongation. Genes involved in auxin and brassinosteroid (BR) signaling are expressed at higher rates in *G. barbadense* than in *G. hirsutum*, and these expression patterns have been associated with the development of longer fibers [11], while the overexpression of the gibberellin 20-oxidase gene (*GhGA20ox1*) might promote the initiation and elongation of cotton fibers through the regulation of gibberellin (GA) synthesis [12]. Interestingly, many genes encoding these proteins and phytohormone-responsive factors might be potential targets for new types of small molecule miRNAs.

Small RNAs (sRNAs), which range in size from 18 to 24 nt, can regulate gene expression at the transcriptional and/or post-transcriptional level [13]. In plants, there are two classes of endogenous sRNAs: small interfering RNAs (siRNAs) and miRNAs. Endogenous siRNAs consist of repeat-associated siRNAs (rasiRNAs), *trans*-acting siRNAs (tasiRNAs), and natural antisense siRNAs (nat-siRNAs). Of these, tasiRNAs are in-phase-generated sRNAs that depend on the miRNA-mediated cleavage of transcripts of tasiRNA genes for their production. Both miRNAs and tasiRNAs can trigger the cleavage of mRNAs containing complementary sequences in plants. As a result, miRNAs play fundamental regulatory roles in various aspects of plant development and adaptive responses to stresses, including plant organ development [14], auxin signaling [15], growth phase changes [16], and disease resistance [17]. Similarly, miRNAs have also been implicated in the development of cotton fibers [18].

A total of 31 miRNA families, including 27 conserved and four novel miRNA families, have been identified in developing cotton ovules using a high-throughput sequencing approach, which showed that the general repression of miRNAs in fibers is associated with the upregulation of a dozen validated miRNA targets that are highly expressed in cotton fibers [18]. Another investigation into cotton fiber sRNAs has also identified more than 100 conserved miRNAs, representing 33 families [19]. In our previous studies, we characterized the dynamic changes of cotton ovule miRNAomes during initiation, ranging from −3 to +3 days pre-/post-anthesis (dpa), and identified seven fiber initiation-related miRNAs [20]. These studies explored the miRNAs that are differentially expressed in the cotton ovules of wild type upland cotton and a fiberless mutant, and the results suggested potential regulatory functions for these miRNAs in cotton fiber initiation. However, there have been no systematic studies of miRNAs expressed during fiber rapid elongation, and the role of miRNAs during cotton fiber elongation remains elusive. A total of 675 plant miRNAs have been identified in the model species *Oryza sativa* (462) and *Arabidopsis thaliana* (213) [21]; however, only 43 miRNAs from *Gossypium spp.* have been registered in miRBase (version 19.0). It is obvious that there are many miRNAs to be identified and much to learn about the specific roles of miRNAs in cotton fiber cells. Furthermore, the recently published genome sequence of the diploid cotton *G. raimondii* has facilitated the identification of sRNAs, particularly miRNAs in this genus [22,23].

In the present study, we performed high-throughput sequencing of cotton fiber sRNAs to identify novel and potential fiber elongation-related miRNAs. Four small RNA libraries were constructed from cotton fibers sampled at four different time points from 5 to 20 dpa, and more than 18 million short RNA sequence reads were generated for each library. The comparative miRNAomic analysis revealed 257 novel miRNAs and nine fiber elongation-associated sRNAs, including eight miRNAs and one tasiRNA, in allotetraploid cotton fiber cells. The predicted targets of the nine sRNAs were experimentally validated and implicated in different cellular responses and metabolic processes, demonstrating the regulatory importance of miRNAs and tasiRNAs in cotton fiber elongation. Thus, this study advances our understanding of the important regulation of miRNAs and tasiRNAs in allotetraploid cotton through the identification of new miRNAs and a putative miRNA-mediated regulatory network supporting cotton fiber cell elongation.

## Results and discussion

### High-throughput sequencing data overview

**Figure 1:**
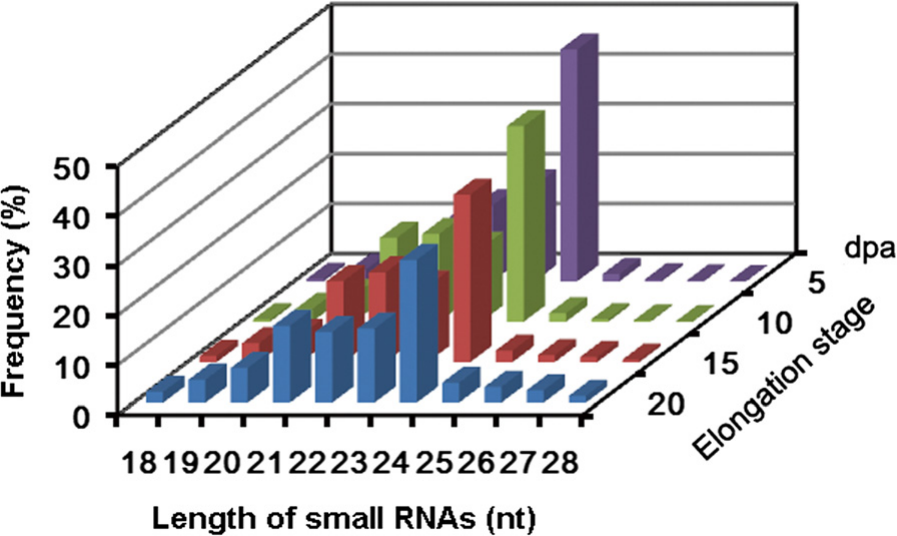
**Sequence length distribution of the clean reads from cotton fiber small RNA libraries isolated at 5, 10, 15 and 20 dpa.** The 24 nt group is clearly much larger than the others.

To identify new miRNAs and potential fiber elongation-associated miRNAs, four small RNA libraries were generated from allotetraploid cotton fibers at 5, 10, 15 and 20 dpa and then subjected to Illumina high-throughput sequencing. The obtained raw sequence reads (a total of 76.2 million reads from four fiber libraries) were computationally processed to remove low-quality reads, contaminated adapters and reads shorter than 18 nt, yielding more than 74.2 million clean reads (97.4% of total raw reads); 21–24 nt sequences accounted for 84.3% of the total clean reads (Figure 1 and Table 1). In the 20 dpa library, for example, the 24 nt reads were the most abundant class (28.7%) and the 21 nt reads were the second most abundant class (15.5%), followed by 23 nt (14.9%) and 22 nt (14.2%) reads. Furthermore, the decrease in the percentage of 24 nt small RNAs from 5 dpa to 20 dpa implied roles for these 24 nt small RNAs in cotton fiber elongation, although the roles need be studied further (Figure 1).

**Table 1:** Distribution of sequence reads in elongating cotton fiber small RNA libraries

**RNA class**	**Total reads**				**Unique reads**			
	**5 dpa**	**10 dpa**	**15 dpa**	**20 dpa**	**5 dpa**	**10 dpa**	**15 dpa**	**20 dpa**
Clean reads	17,979,849	18,747,051	19,597,538	17,845,500	7,335,047	7,111,913	6,491,658	4,878,094
Match genome^a^	4,983,316	7,113,695	7,107,883	8,871,116	1,828,847	1,876,311	1,731,526	1,299,290
Protein-coding genes^a^	198,666	175,439	189,071	160,087	82,988	90,480	112,705	101,770
Known miRNAs^b^	307,112	641,621	553,022	419,974	803	942	909	809
Repeats^a^	467,372	509,173	454,975	441,256	197,195	182,749	162,696	125,578
rRNA^a^	395,288	992,921	1,363,365	2,212,532	14,564	22,415	26,009	33,079
tRNA^a^	174,510	1,106,240	1,104,728	2,040,671	2,418	3,772	4,456	6,779
snRNA^a^	1,487	2,743	3,654	5,026	585	940	1,111	1,288
snoRNA^a^	301	511	629	540	187	267	302	256
Others^c^	16,435,113	15,318,403	15,928,094	12,565,414	7,036,307	6,810,348	6,183,470	4,608,535

^a^Based on the *G. raimondii* whole genome sequence (http://cgp.genomics.org.cn/page/species/index.jsp);

^b^The number of reads includes the defined mature miRNAs and their 2-nt mismatch variants;

^c^Contains all of the unclassified reads that potentially include new miRNAs.

The clean reads were mapped to the diploid cotton *G. raimondii* genome, generating 4983316, 7113695, 7107883 and 8871116 genome-matched reads from the 5, 10, 15 and 20 dpa fiber libraries, respectively (Table 1). These small RNA reads were grouped into several RNA classes, such as known miRNAs, rRNA, tRNA, snRNA, snoRNA, protein-coding genes and repeats. The remaining unclassified small RNA reads had the highest fraction of unique and total clean reads and likely included new types of regulatory small RNAs and novel miRNAs. For example, in the 10 dpa library, the unclassified small RNA reads accounted for 95.8% and 81.7% of the unique and total clean reads, respectively (Table 1).

### Known miRNAs in elongating cotton fiber cells

Currently, miRBase (version 19.0, released on August 1, 2012) lists just 43 mature miRNA genes belonging to 21 miRNA gene families as cloned or predicted to be expressed in cotton. Based on our previous work, 36 new miRNAs from cotton ovules were officially named by miRBase and included in the subsequent release [20]. To identify the known miRNAs, the clean reads were subjected to a BLASTn search for known miRNA sequences deposited in miRBase (version 19.0) and the newly identified cotton miRNAs [20]. Nearly 6000 unique sequences of known miRNAs belonging to 79 families were obtained from these four sRNA libraries (Additional file 1: Table S1). In addition to the 21 known cotton miRNAs families in miRBase (version 19.0) and 23 miRNAs families described in the previous study [20], 35 known miRNA families were also identified based on their conservation in other plant species. In total, the 79 miRNA families included not only nearly all the known miRNA families previously identified in cotton [18,20] but also all 24 miRNA families that are conserved between eudicotyledons and monocotyledons [24].

To accurately measure miRNA read abundances, the abundances of known miRNAs were normalized to reads per ten million (RPTM). The four miRNA families with the greatest abundance were 167 (164706 RPTM), 156/157 (86430 RPTM), 165/166 (71375 RPTM), and 894 (39985 RPTM), accounting for 41.3%, 21.7%, 17.9% and 10.0% of all the known miRNA reads, respectively. The miRNA families 164, 168, 3476, 7508, 172, 390, 535, 159/319, 2911, 2950 and 396-3p also exhibited average abundances of more than 1000 RPTM. In contrast, certain miRNA families were expressed at low abundance (Additional file 1: Table S1). The varied abundances of the miRNAs in different families suggested that these miRNA genes are differentially transcribed during fiber elongation.

A total of 213 potential miRNA gene loci, belonging to 48 miRNA families, were identified based on the EST sequences (CGI11), genome survey sequences (GSS) and *G. raimondii* genome sequences using mireap_0.2 (Additional file 2: Table S2), comprising 37 of the 43 cotton miRNA genes in miRBase (version 19.0). Of the 213 potential loci, 174 originated from the *G. raimondii* genome sequence, including 32 of the 43 *Gossypium* precursors deposited in miRBase (19 version); 34 loci originated from CGI11; and six loci originated from GSS, suggesting that many miRNA genes with low expression were absent from the ESTs database (CGI11) and that the *G. raimondii* genome sequences could be used for the analysis of miRNA precursors identified in *G. hirsutum* high-throughput small RNA sequencing (Additional file 2: Table S2). Furthermore, as shown in Additional file 3: Figure S1, 139 novel precursors of known miRNAs (i.e., precursors not present in miRBase version 19) were identified.

Of the 48 known families with precursors, 26 had miRNA* sequences, providing evidence for a DCL1-processed stem-loop (Additional file 1: Table S1 and Additional file 2: Table S2). As expected, the majority of miRNAs showed dominant abundance of one arm of the stem loop, suggesting that the miRNA* on the complementary arm was degraded quickly after biogenesis [13]. However, for miR162, miR169, miR2111 and miR393, similar numbers of 5p and 3p reads were observed. Furthermore, miR160-3p and miR396-3p were even more abundant than miR160-5p and miR396-5p (Additional file 1: Table S1); similar phenomena were also reported in a study of the small RNAs in *Brassica napus*[25].

The sequence of the most abundant miRNA in each miRNA family (i.e. the representative miRNA sequence) is shown in the “Sequence” column of Additional file 1: Table S1. However, a subset of the representative sequences diverged among the different time-course small RNA libraries (superscript d to j in Additional file 1: Table S1). In fact, many known miRNA families not only had multiple loci but also certain members that varied by few nucleotides (“location” and “miRNA sequences” columns in Additional file 2: Table S2). The miR156 (six members), miR159/319 (four members), miR165/166 (five members), miR167 (three members), miR169_1 (six members), miR169_2 (five members), miR172 (five members), miR396 (three members), miR399 (six members), miR477 (six members), miR482 (seven members) and miR7504b (three members) families all contained more than two distinct members (“member” column in Additional file 2: Table S2). The representative sequence of miR167 from 5–15 dpa was “UGAAGCUGCCAGCAUGAUCUCA” and contained an adenylation modification at the 3’ end of the mature miRNA compared to the representative sequence of miR167 shown in Additional file 1: Table S1. This adenylation might protect the miRNA from degradation [26]. Hence, the presence of this highly abundant and stable form of miR167 might indicate a crucial role for miR167 in cotton fiber elongation. Similarly, the representative sequences of miR172 and 397 differed between 5–15 dpa and 20 dpa. The representative sequences of miR172 and miR397 at 5–15 dpa were “AGAAUCCUGAUGAUGCUGCAG” (83 in “index” column in Additional file 2: Table S2) and “UCAUUGAGUGCAGCGUUGAUG” (118 in “index” column in Additional file 2: Table S2), whereas the representative sequences at 20 dpa were “AGAAUCUUGAUGAUGCUGCAU” (79 and 81 in “index” column in Additional file 2: Table S2) and “UUGAGUGCAGCGUUGAUGAGC” (45 in “index” column in Additional file 2: Table S2), respectively. This observation suggested that the members of a known miRNA family are expressed differentially during different stages of cotton fiber elongation.

### Newly identified miRNAs in elongating cotton fibers

As shown in the analysis of known miRNA precursors, 174 of 213 precursors originated from *G. raimondii*, indicating that the *G. raimondii* genome sequence could be used for exploring the novel miRNAs from *G. hirsutum* high-throughput small RNA sequencing. Thus, after excluding sRNA reads homologous to known miRNAs (≤ 2 mismatches) in miRBase (19.0) and other annotated sRNAs (Table 1), the remaining small RNAs were mapped to the *G. raimondii* genome and subjected to a rigorous secondary structural analysis of their precursors. The precursors with a canonical stem-loop structure were further analyzed to ensure adherence to research community standards: (1) The set of reads from each candidate miRNA locus should account for more than 95% of all the precursor-mapped small RNA reads, and reliable candidate miRNA reads (at least five reads) should account for more than 75% of the corresponding set of reads (Additional file 4: Table S3 and Additional file 5: Figure S2); (2) the miRNA* reads should have two-nucleotide 3’ overhangs; or (3) the base-paring mismatch between the miRNA and the other arm of the hairpin, as well as between the miRNA* sequence and the other arm, should be less than five; meanwhile, no asymmetric bulges larger than two nucleotides and no more than two asymmetric bulges should be present within the miRNA/miRNA* duplex [27] (Additional file 6: Figure S3). Of these three rules, (1) is a fundamental feature of plant miRNAs, guaranteeing the precise excision of an approximately 21 nt miRNA/miRNA* duplex from the precursor. Although the miRNA* structure is also an important feature of plant miRNAs, because the abundance of many candidate novel miRNAs was low, rule (2) can be replaced by rule (3). In total, 257 novel miRNAs from 314 miRNA gene loci were identified based on these three rules (Additional file 4: Table S3); 36 of the 257 novel miRNAs were associated more than one miRNA gene locus, and the other 221 were associated with a single unique locus (Additional file 4: Table S3). Moreover, 18 of the 257 novel miRNAs had the miRNA* sequence (Additional file 4: Table S3, Additional file 5: Figure S2 and Additional file 6: Figure S3). The 24 nt miRNAs were the dominant species (130) among the 257 novel miRNAs, followed by 21 nt species (87), and other species were much less frequent (Table 2). miRNAs are typically 21 nt in plants; however, 24 nt miRNAs (lmiRNAs) were recently identified and shown to be loaded in AGO4 [28]. Notably, AGO4 displayed a preference for sRNAs with 5’ terminal adenine. As expected, the more novel 24 nt miRNAs (54) had 5’ terminal adenine nucleotides (Table 2) [29]. Furthermore, a majority of the novel 21 nt miRNAs (48) had 5’ terminal uridine nucleotides, consistent with the observation that AGO1 usually harbored miRNAs with a 5’ terminal uridine (Table 2) [29]. Seven of the 257 novel miRNAs, including novel_mir_5042, novel_mir_2748, novel_mir_4168, novel_mir_5106, novel_mir_5046, novel_mir_2543 and novel_mir_4061, might be specifically expressed in the cotton fiber (Additional file 4: Table S3). Although the abundances of these novel miRNAs were lower than those of the conserved miRNAs, these miRNAs were detected in all four fiber sRNA libraries but not in young seeds (Additional file 4: Table S3, young seed reads not shown). Specifically, novel_mir_5042 possessed a miRNA^*^ sequence and is predicted to target glucosyltransferase, which is important for generating the cell wall. The 174 known and 311 novel miRNA gene loci were dispersed throughout all thirteen *G. raimondii* chromosomes (Additional file 7: Figure S4, two loci of GhmiRnA and one locus of GhmiRnB could not be anchored to any of the 13 *G. raimondii* chromosomes). To date, a total of 43 miRNA precursors have been found in *Gossypium*, including 37 precursors in *G. hirsutum*, four precursors in *G. raimondii*, one precursor in *G. arboretum,* and one precursor in *G. herbaceum* (miRBase, 19.0). In this study, 257 novel miRNA families, including 314 miRNA loci, were identified, significantly increasing the number of known cotton miRNA families.

**Table 2:** The 5’ terminal nucleotides and lengths of the 257 novel cotton miRNAs

**5’ terminal**	**miRNA length (nt)**					
	20	21	22	23	24	Total
A	3	23	1	6	54	87
C	1	11	1	2	15	30
G	0	5	2	2	8	17
U	3	48	6	13	53	123
Total	7	87	10	23	130	257

### Cotton fiber elongation-related miRNAs

**Figure 2:**
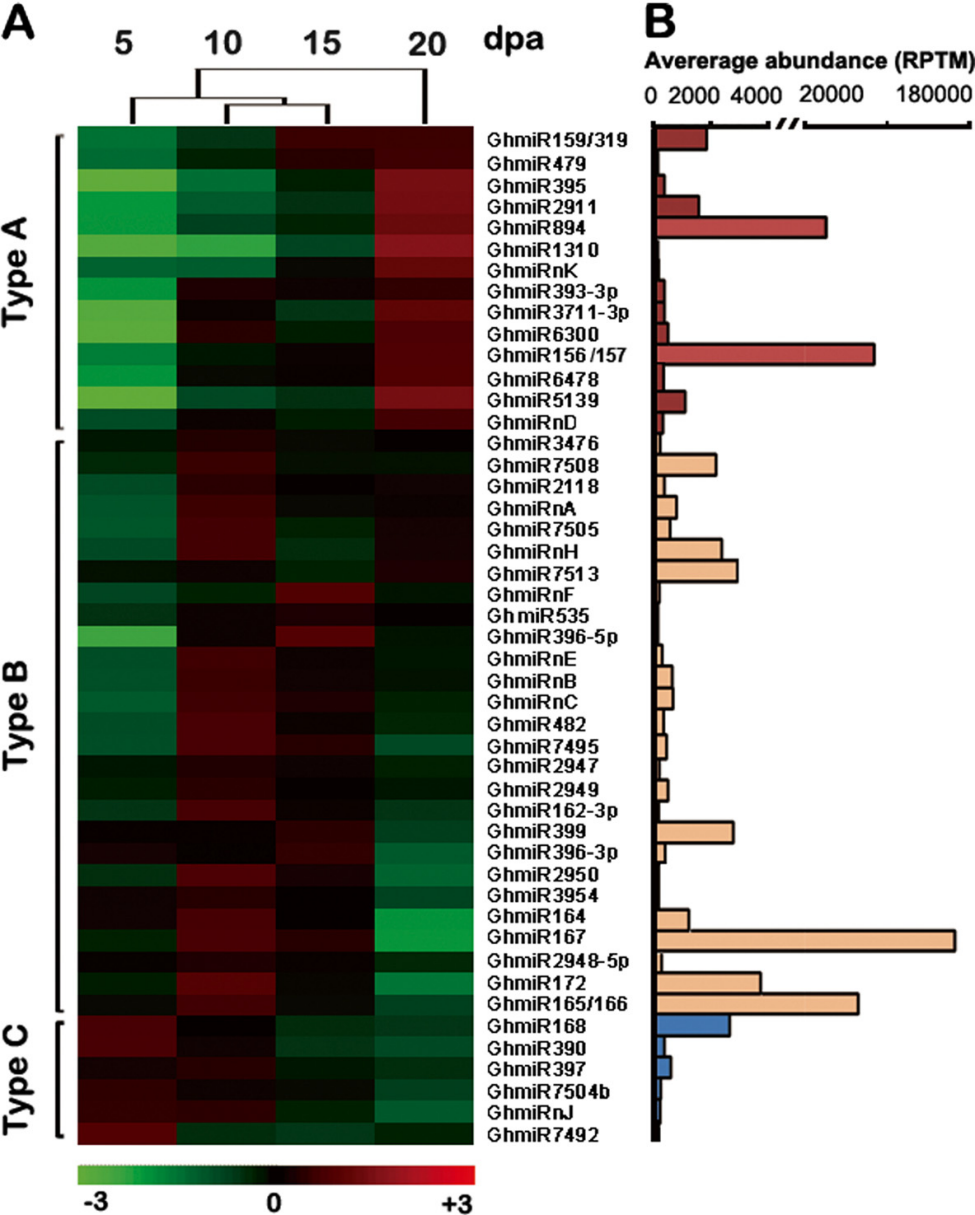
**The 46 cotton fiber elongation-related miRNAs. (A)** Complete linkage hierarchical cluster analysis of differential expression miRNAs in the cotton fibers (5–20 dpa) was performed by comparing the RPTM of GhmiRNA in every library to the average of the four cotton fiber sRNA libraries. The color indicates the fold-change as log2, from high (red) to low (green), as indicated in the color scale. On the right are names of the GhmiRNAs, on the left are the types to which they belong, and on the top is the dendrogram of the 5, 10, 15 and 20 dpa small RNA libraries generated by the cluster analysis of the 46 differentially expressed miRNAs. **(B)** The average abundances of 46 cotton fiber elongation-related miRNAs. RPTM, reads per ten million. Types A, B and C are shown in red, yellow, and blue, respectively, in Figure 2A.

To elucidate the potential regulatory roles of miRNAs in cotton fiber elongation, we analyzed the differential expression profiles of miRNAs. To minimize noise and improve accuracy, only miRNAs expressed at greater than 100 RPTM in at least one library were used for comparison. After Fisher’s test (p < 0.01) and cluster analyses, as shown in Figure 2A, the remaining 37 known and nine novel miRNAs exhibited differential expression during cotton fiber elongation and clustered into three groups with similar profiles. The known GhmiR159/319, 479, 395, 2911, 894, 1310, 393-3p, 3711-3p, 6300, 156/157, 6478 and 5139 and novel GhmiRNAs K and D, belonging to Type A, showed a gradual increase in read number during cotton fiber elongation, particularly at 20 dpa, the point at which fiber elongation is terminated [10]. Thus, Type A miRNAs might serve as negative regulators of fiber elongation. The accumulation of miRNAs in the known GhmiR3476, 7508, 2118, 7505, 7513, 535, 396-5p, 482, 7495, 2947, 2949, 162-3p, 399, 396-3p, 2950, 3954, 164, 167, 2948-5p, 172 and 165/166 and the novel GhmiRNAs A, H, F, E, B and C, belonging to Type B, peaked at 10 or 15 dpa, during the rapid fiber elongation period [5], indicating that these miRNAs could promote rapid fiber elongation. The accumulation of GhmiR168, 390, 397, 7504b, 7492 and the novel GhmiRNA J, belonging to Type C, were gradually reduced during cotton fiber elongation, implying that these miRNAs might play important negative regulatory roles in fiber elongation after 5 dpa (Figure 2A). We selected GhmiR156, GhmiR167 and GhmiR168 for qRT-PCR analysis to test the reliability of the deep sequencing analyses. The qRT-PCR results of these three miRNAs showed trends similar to those observed in the deep sequencing data (Additional file 8: Figure S5). Hence, the miRNA analysis from our deep sequencing library should be considered highly reliable and appropriate for cluster analysis. Meanwhile, the small RNA libraries generated at the four time points formed two clusters, the fiber elongation cluster (5, 10 and 15 dpa) and the termination of fiber elongation cluster (20 dpa). The 10 and 15 dpa profiles were most similar to one another, likely because cotton fibers at 10 and 15 dpa were undergoing rapid elongation. Hence, the results of cluster analysis were consistent with the characteristics of fiber development.

### A newly identified tasiRNA in cotton

**Figure 3:**
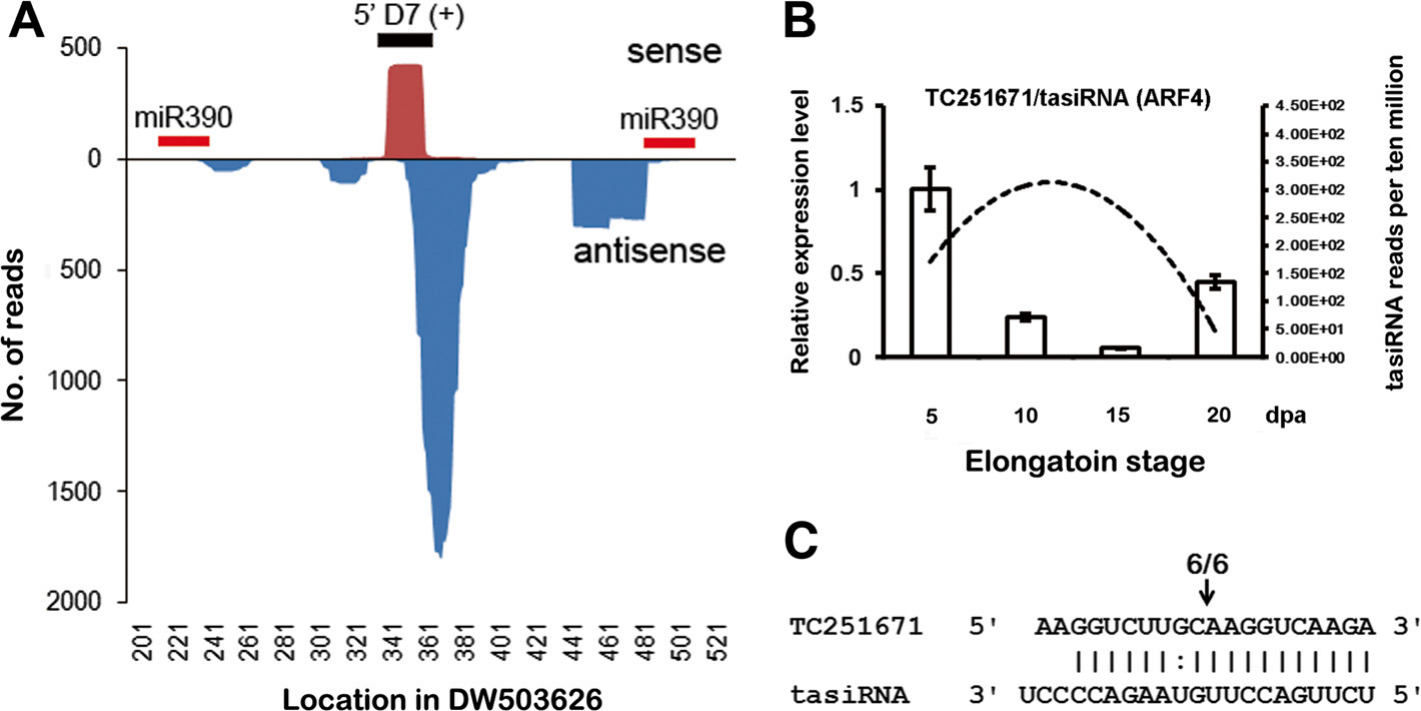
**miRNA-derived tasiRNA in *G. hirsutum*. (A)** The expression levels of tasiRNAs generated from DW503626. The locations of the two miR390 target sites and the 5’ D7 (+) are indicated by short bars. **(B)** Sequencing reads of tasiR-ARF generated from DW503626 and quantitative RT-PCR analysis of the tasiR-ARF target gene TC251671 at different fiber developmental stages. The sequencing reads are indicated as dotted lines and the relative expression level of TC251671 is indicated as a histogram. The error bars indicate the SD of three replicates. **(C)** Validation of the cleavage of *G. hirsutum* TC251671 (ARF4) mRNAs by tasiR-ARF using 5’-RLM-RACE.

tasiRNA is an important clade of regulatory sRNAs in plants, although they have not been previously identified in cotton. In Arabidopsis, four canonical *TAS* gene families produce transcripts that are cleaved by miR173, miR390, or miR828 [30]. Because miR173 was not detected in any deep sequencing libraries, and the abundance of miR828 was very low (Additional file 1: Table S1), the predicted targets of miR390 were analyzed through BLAST analysis to search for small RNA sequences in the sequencing library and identify conserved *TAS3* genes in cotton. Phased small RNA clusters from one gene, DW503626, were identified (Figure 3A). This gene sequence showed high homology to the Arabidopsis *TAS3* gene (data not shown); thus, DW503626 was considered to represent the cotton *TAS3* gene.

Similar to the gene structure in Arabidopsis, the tasiRNA-generating locus in *G. hirsutum* is flanked by two miR390 binding sites (Figure 3A), and most of the tasiRNAs were 21 nt long (Additional file 9: Figure S6). The most abundant sense strand-produced sRNAs were produced at the 5’D7 (+) position (Figure 3A), similar to the pattern observed in Arabidopsis, suggesting that the tasiRNA biogenesis pathway is highly conserved between plant species.

We analyzed the expression patterns of DW503626 5’D7 (+), considered a homolog of functional tasiRNAs in Arabidopsis, during fiber development based on the sequence number. The accumulation of tasiRNA increased from 5 to 10 dpa and decreased from 10 to 20 dpa (Figure 3B). Target prediction revealed that genes of the Auxin Response Factor (ARF) family, such as ARF2 (TC239405), ARF3 (TC232322), and ARF4 (TC251617), were putative targets of tasiRNAs. The *in vivo* cleavage of ARF4 (TC251617) transcripts was detected using RNA ligase-mediated (RLM) 5’ RACE (Rapid Amplification of cDNA Ends) (5’-RLM-RACE) (Figure 3C). Furthermore, the expression of *ARF4*, as determined using quantitative RT-PCR (qRT-PCR), was negatively correlated with tasiRNA accumulation (Figure 3B), suggesting that its expression was strongly regulated by tasiRNA during fiber development.

tasiR-ARF, a conserved tasiRNA that targets ARFs in plant species, was required for proper leaf polarity development [31,32] and the suppression of the juvenile-to-adult phase transition in Arabidopsis [33]. In developing cotton fibers, tasiRNA repressed ARF4 during the rapid elongation phase (Figure 3B). It remains unclear whether this regulatory pathway contributes to fiber elongation, although ARF genes are crucial components of the auxin-signaling pathway and have been suggested to play an important role in fiber development [4]. Nevertheless, this represents the first identification of a tasiRNA in *G. hirsutum*, which expands the plant tasiRNA family and demonstrates its conservation among different species.

### Target prediction, validation, and expression correlated with miRNAs

To characterize the functions of miRNAs with differential RPTM in cotton fiber elongation, we first predicted and experimentally validated their targets. Using the psRNATarget Web server (http://plantgrn.noble.org/psRNATarget/), the targets of the miRNAs shown in Figure 2A were determined (Additional file 10: Table S4 and Additional file 11: Table S5). Subsequently, 5’-RLM-RACE was performed to obtain the target cleavage transcripts. To maximize the detection efficiency of miRNA-triggered target cleavage, RNAs extracted from 5, 10, 15 and 20 dpa fibers were separately ligated to a 5’ RACE adaptor and used to construct RACE libraries. The RACE library with the greatest abundance of a particular miRNA (according to sequencing reads) was used to validate the corresponding target.

**Figure 4:**
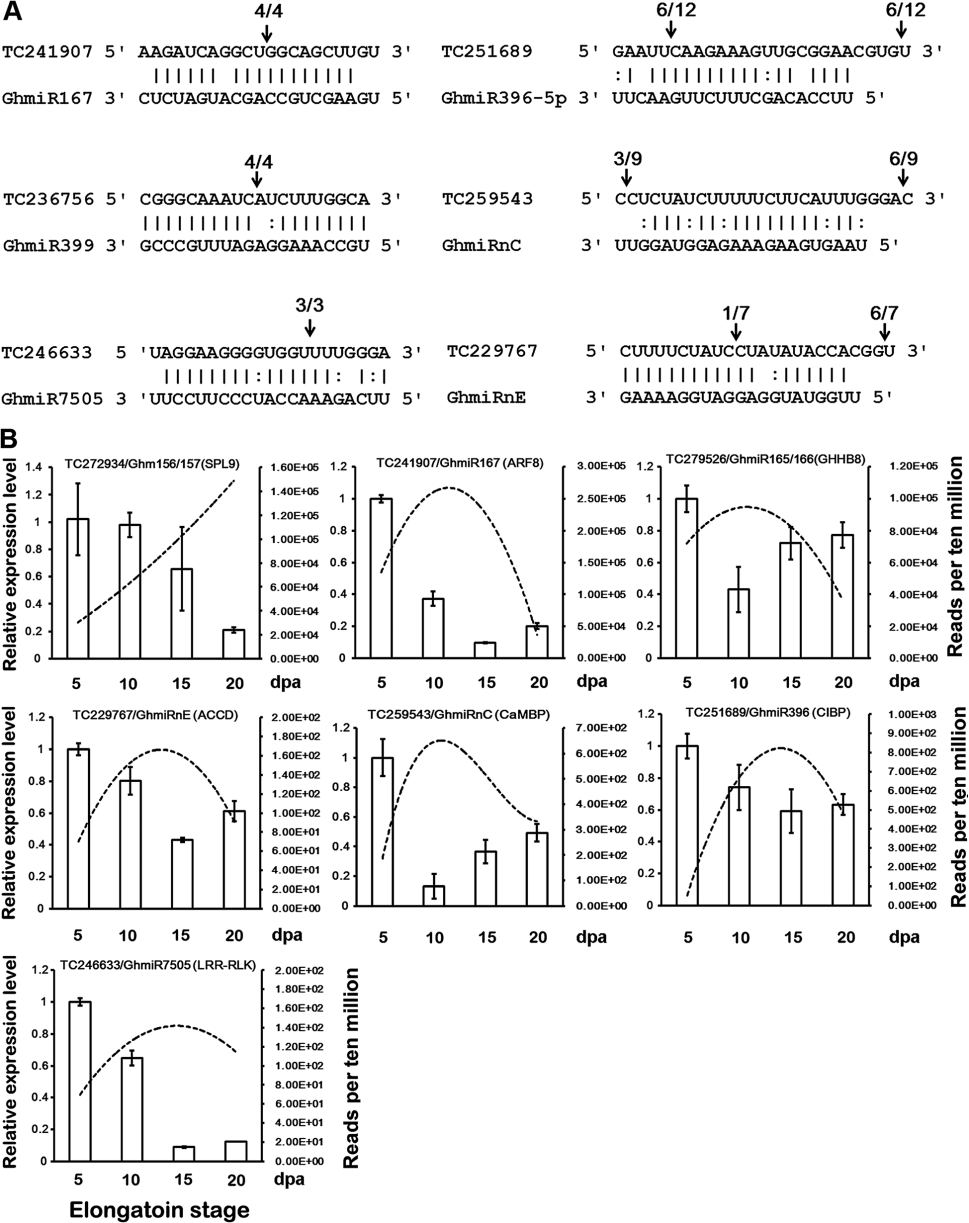
**Target gene validation and expression analysis. (A)** Mapping of target mRNA cleavage sites using 5’-RLM-RACE. The arrows indicate the cleavage sites and the number shows the frequency of the clones sequenced. **(B)** Quantitative real-time PCR analysis of the target mRNAs. The data represent the mean values ± SD of three replicates. The dots represent the corresponding abundance on the right axis in four small RNA libraries. TC241907: ARF8 (auxin response factor 8); TC236756: MYB; TC259543: CaMBP (calmodulin-binding protein); TC229767: ACCD (acetyl-CoA carboxylase beta subunit); TC251689: CIBP (calcium ion-binding protein); TC246633: LRR-RLK (leucine-rich repeat receptor-like protein kinase); TC277934: SPL9 (squamosa promoter-binding protein-like 9); TC279526: GHHB8 (HD-Zip protein 8).

Using this strategy, miRNA-guided target cleavage was examined for targets of the miRNAs listed in Figure 2 (Additional file 10: Table S4 and Additional file 11: Table S5). In total, six target genes of miRNA were identified for the first time (Figure 4A). Calmodulin-binding protein (*CaMBP*, TC259543) and the beta subunit of acetyl-CoA carboxylase (*ACCD*, TC229767) were identified as targets of the novel miRNAs GhmiRnC and GhmiRnE, respectively. Similarly, ARF8 and LRR receptor-like protein kinase (*LRR-RLK*) were also newly identified as targets of the known miRNAs GhmiR167 and GhmiR7505, respectively. Additionally, calcium ion binding protein (*CIBP*, TC251689) and the MYB transcription factor (*MYB*, TC236756) were identified as targets of GhmiR396 and GhmiR399, respectively; in contrast, the corresponding miRNAs in Arabidopsis target growth-regulating factor (*GRF*) and the putative ubiquitin-conjugating enzyme (*UBC*). Consistent with previous research, the cleavages of *GhSPL9* and class III HD-Zip protein 8 (*GHHB8*), the targets of GhmiR156/157 and GhmiR165/166, respectively, were also detected (data not shown) [18,20].

As shown in Figure 4A, the cleavage products of TC241907 (*ARF8*) and TC236756 (*MYB*) were precisely terminated at the tenth position from the 5’ end of the complementary region of each associated miRNA. However, the TC246633 (*LRR-RLK*) sequences were cleaved three nucleotides downstream of the predicted site (Figure 4A), which could reflect further nuclease-mediated degradation of the targets after miRNA-mediated cleavage [34]. Two cleavage sites were found for TC229767 (*ACCD*), TC259543 (*CaMBP*), and TC251689 (*CIBP*). One cleavage site was located downstream of the predicted site. As described for the cleavage product of LRR-RLK, these cleavage products may be further degraded after silencing by the corresponding miRNAs [34]. The other cleavage site was located upstream of the predicted site. This type of cleavage was also found in many other 5’-RLM-RACE experiments [35,36], indicating that the potential function of the cleavage by these miRNAs warrants further investigation.

The expression of validated miRNA target genes was examined using qRT–PCR to determine whether there was a negative correlation between the accumulation of miRNAs and their targets at various periods during development. As shown in Figure 4B, for the target of Type A GhmiRNAs, TC272934 (*GhSPL9*) transcript levels were negatively correlated with the accumulation of GhmiR156/157. In contrast to GhmiR156/157 expression, which increased from 5 to 20 dpa, TC272934 (*GhSPL9*) expression was reduced from 5 to 20 dpa. For the targets of Type B GhmiRNAs, which increased from 5 dpa and reached peak levels at 10 or 15 dpa, the expression level was reduced from 5 dpa to 10 or 15 dpa and subsequently upregulated at 20 dpa. These data suggested that the accumulation of these GhmiRNAs was negatively correlated with the expression of their targets in elongating fibers and showed that the target genes were strictly controlled through these GhmiRNAs. Additionally, MYB, which is a target of miR399, was expressed at low levels and did not meet MIQE guidelines [37]; thus, no qRT-PCR analysis was performed for this gene.

### A potential functional network of fiber elongation-related tasiRNA and miRNAs

**Figure 5:**
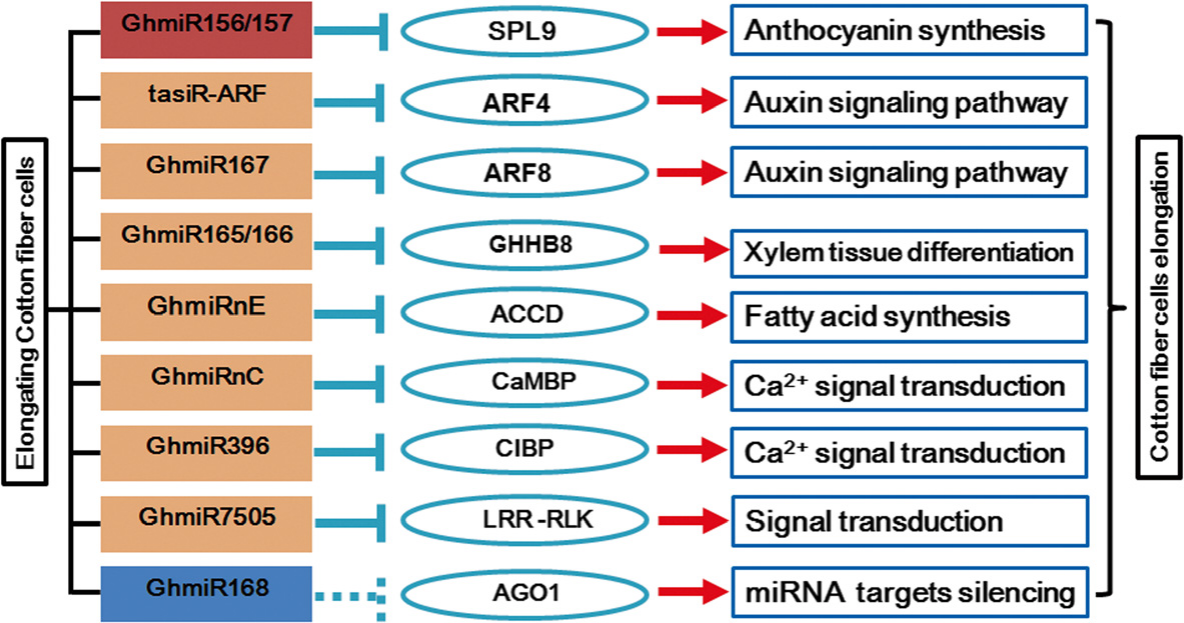
**A potential functional network mediated through miRNAs during fiber elongation.** The color of the miRNA box indicates the type of expression pattern and is consistent with the data shown in Figure 2; red indicates the gradually increased miRNAs, yellow indicates miRNAs that initially increased and subsequently decreased, and blue indicates the gradually reduced miRNAs. The arrows indicate positive regulation, and the nail shapes represent negative regulation.

The unique biological process of cotton fiber elongation relies on the complex regulation of gene expression. miRNAs, which are small but crucial molecules in the regulation of gene expression, play an important role in the process of fiber elongation [19]. However, no systemic investigation has been conducted to determine how cotton fiber elongation via miRNA-mediated regulation occurs. In this study, a comparative miRNAome analysis combined with 5’-RLM-RACE revealed eight fiber elongation-related miRNAs expressed in cotton fibers and a negative correlation between miRNA and target expression in elongating cotton fibers (Figure 4). Therefore, these results have fully demonstrated that miRNA-mediated regulation occurs in response to fiber elongation. A regulatory network connecting fiber elongation-related miRNAs to their targets plays a crucial role in fiber elongation (Figure 5).

For a cotton fiber cell, the precise control of the elongation period is critical. It is not known how the fiber cell knows when to start or end the rapid elongation stage. However, an H_2_O_2_ burst at 20 dpa may serve as a termination signal for rapid fiber elongation [10]. Anthocyanins are widely identified in plant species to provide flowers and fruits with color [38] and protect the plants from damage by active oxygen species [39]. Previous studies reported that the SPL9, targeted by miR156, negatively regulates anthocyanin accumulation by directly preventing the expression of the anthocyanin biosynthetic genes *DFR*, *F3H*, and *ANS*[40]. In this study, the reads of GhmiR156/157 increased gradually from 5 dpa to 20 dpa and were negatively correlated with the level of *GhSPL9* expression (Figure 4B). However, the expression levels of the anthocyanin biosynthetic homologous genes in cotton, *GhDFR*, *GhANS*, *GhF3H*, and the levels of anthocyanin in developing fibers were reduced from 5 to 20 dpa, similar to the expression pattern of *GhSPL9*, suggesting that GhSPL9 might play a positive role in regulating the expression of these genes through an unknown mechanism that might be distinct from that in Arabidopsis (Additional file 12: Figure S7A and B). Hence, GhmiR156/157, via its mediation of the cleavage of *GhSPL9* mRNA, might negatively regulate the expression of the anthocyanin biosynthetic genes *GhDFR*, *GhF3H*, and *GhANS*. This in turn may lead to a strong decline in anthocyanin synthesis at 20 dpa, which may be crucial for the H_2_O_2_ signaling in fiber elongation termination.

Calcium-mediated signal transduction plays crucial roles in a wide array of growth and developmental processes. In plants, changes in cellular Ca^2+^ concentration are transmitted through several calcium sensors or calmodulin (CaM) [41]. The obvious inhibition of fiber growth was observed when ovules were cultured in the presence of the CaM antagonist TFP [9]. Here, we identified the calcium sensor CIBP as a target of GhmiR396-5p. Therefore, we speculated that GhmiR396-5p, via its mediation of the cleavage of *CIBP* mRNA, might promote fiber rapid elongation from 5 dpa. Furthermore, we also identified the calmodulin (CaM)-binding protein CaMBP as a target of GhmiRnC. CaM isoforms, which might bind CaMBP, show high affinity to the kinesin-like CaM-binding motor protein (KCBP), which regulates cotton fiber elongation [8,42]. Therefore, we speculated that the regulation of CaMBP through miRNAs could play important roles in cotton fiber elongation through Ca^2+^ signal transduction.

Various studies on cotton fiber cell development have identified plant hormones as critical regulators of fiber development. Auxin promotes the fiber cell development of *in vitro* cultured ovules, and a positive correlation between final fiber length and *in vivo*-quantified IAA levels has also been observed [43]. ARF8, a target of GhmiR167, was involved in IAA signal transduction [44]. Previous evidence showed that an exogenous auxin signal was transduced sequentially through miR167 and ARF8 [45]. The reduced expression of *GhARF8*, which is regulated by GhmiR167 during rapid fiber elongation (Figure 4B), was consistent with the negative roles of *AtARF8* in cell expansion during hypocotyl and lateral root elongation [46,47]. Hence, GhmiR167, via its mediation of the cleavage of *ARF8* mRNA, might be critical for rapid fiber elongation. The accumulation of tasiR-ARF exhibited dynamics similar to those of GhmiR167, and its target, ARF4, also showed an expression pattern similar to that of ARF8, which is targeted by GhmiR167 (Figure 3B, 4B). This observation suggests that tasiR-ARF might be involved in fiber elongation, similar to GhmiR167. In addition to auxin, BR has been suggested to promote cotton fiber elongation. Cotton fiber responses to BR are mediated through a plasma membrane-bound leucine-rich (LRR) receptor-like protein kinase known as BRI, which has an expression pattern and domain structure similar to that of LRR-RLK, which is targeted by GhmiR7505 (Figure 4B) [48]. Moreover, we detected significantly higher *LRR-RLK* expression in *G. hirsutum* compared with *G. arboreum*, which has shorter fibers than does *G. hirsutum,* throughout the elongation phase (Additional file 13: Figure S8). The expression of *LRR-RLK* was high in 5 to 10 dpa and sharply declined at 15 dpa (Figure 4B), suggesting that GhmiR7505-regulated *LRR-RLK* might play a positive role in initiating rapid fiber elongation.

The rapid elongation of cotton fiber cells requires substantial lipid synthesis to support the developing organelles and membranes. ACCD, the target of GhmiRnE, encodes the β-carboxyl transferase subunit of acetyl-CoA carboxylase (ACCase, EC 6.4.1.2), which is a regulatory enzyme in fatty acid synthesis [49]. However, fatty acid synthesis should be regulated in the rapid elongation stage of the cotton fiber. A recent report showed that the down-regulation of *WRI1*, a positive regulator of lipid biosynthesis, reduced carbon flow into fatty acid biosynthesis, including the expression of ACCase; meanwhile, the fixed carbon was channeled into fiber growth, resulting in longer fibers [50]. Hence, the GhmiRnD promoted cotton fiber elongation by mediating the decrease in *ACCD* expression and diverting more carbon into fiber growth. Unlike many other plant cells, cotton fibers contain little to no lignin in their secondary cell walls [4,51]. Lignin is primarily deposited into the cell walls of xylem vessel elements, and *irregular xylem4* (*irx4*) mutant plants have 50% less lignin than wild-type plants [52]. The ectopic expression of *ATHB-8* in Arabidopsis could promote vascular cell differentiation, specifically the differentiation of xylem tissue [53,54]. miR166 exhibited strong downregulation in high-lignin tissues (wood) in *Acacia mangium*, and the expression of the miR166 target *HD-ZIP 3* is much higher in high-lignin tissue (compression wood) [55]. The number of reads of GhmiR165/166 in rapid elongation fibers (10 and 15 dpa) was much higher than that in 15 dpa seeds (Additional file 14: Table S6), and the expression of *GHHB8* at this stage was clearly repressed (Figure 4B). We speculated that the lower *GHHB8* expression mediated by GhmiR165/166 might contribute to fiber elongation by inhibiting the differentiation of high-lignin tissues, such as xylem, because a number of lignification mutants revealed the link bewteen cell expansion and xylem tissue differentiation [56,57].

In this study, 46 GhmiRNAs were differentially expressed during the cotton fiber elongation progress, 40 (Type of A and B GhmiRNAs) of which were dramatically upregulated during rapid fiber cell elongation (10 and 15 dpa). In Arabidopsis, miRNAs are loaded into AGO1, which acts an RNA slicer and is the target of miR168 [58,59]. Although no AGO1 (CO084924) cleavage site was detected in the complementary region of GhmiR168 (data not shown), we speculated that GhmiR168 might cleave *AGO1* mRNA. According to the tendency of GhmiR168 to decrease from 5 to 20 dpa, the expression of *AGO1* mRNA should be upregulated. The increased expression of AGO1, mediated through GhmiR168, might facilitate the gene silencing of the type A and B GhmiRNA targets discussed above.

Furthermore, the abundance of GhmiRNAs in this network accounted for approximately 80% of the total miRNA reads. As shown in Additional file 14: Table S6, the read numbers of Type A and B miRNAs were increased at 10 and 15 dpa (rapid elongation) compared with 5 dpa, and the reads of Type A and B (except for Ghmi167) were higher in 15 dpa fibers than in 15 dpa seeds. Similarly, the Type C read numbers were decreased at 10 and 15 dpa compared with 5 dpa, and the Type C reads numbers were lower in 15 dpa fibers than in 15 dpa seeds. These trends indicated that the miRNAs in this regulatory network played special roles in cotton fiber cell elongation to some extent. Taken together, the data provided in this study showed that miRNAs are precisely regulated during fiber elongation and play important roles in various biochemical processes that promote fiber elongation. Hence, we propose the miRNA-mediated response plays an important role in fiber elongation, opening new avenues for the functional study of miRNAs in fibers and even plant cell elongation.

## Conclusions

A total of 79 known miRNA families and 257 novel miRNAs from cotton fibers were identified through the high-throughput sequencing of sRNAs isolated from 5, 10, 15 and 20 dpa cotton fibers. This expands the population of known *G*. *hirsutum* miRNAs and confirmed the existence of ta-siRNAs in *G*. *hirsutum*. Comparisons of the miRNAomes of each stage revealed that 46 miRNAs were differentially expressed during the cotton fiber elongation; further, the target genes of eight miRNAs and one ta-siRNA were validated through 5’-RLM-RACE. The detailed analysis of the expression of their targets at different developmental time points hinted at their varied roles in the regulation of fiber elongation in *G*. *hirsutum*.

## Methods

### Plant materials

Upland cotton (*Gossypium hirsutum* cv. CRI35) was field grown under normal agronomic conditions. The Cotton Research Institute, Chinese Academy of Agricultural Sciences, kindly provided these seeds. The flowers were bagged one day prior to anthesis to prevent hybridization with other varieties and tagged on the day of anthesis after the removal of the bag. Normal bolls were harvested at 5, 10, 15 and 20 dpa and temporarily stored on ice. The young seeds with fibers were stripped of hulls, frozen in liquid nitrogen, and stored at −80°C until further use.

### Small RNA library construction and Solexa sequencing

Total RNA was extracted from the frozen 5, 10, 15 and 20 dpa fibers and the 15 dpa young seeds (as a reference) using the PureLink™ Plant RNA Reagent kit (Invitrogen, Carlsbad, CA) according to the manufacturer’s instructions. The RNA quality was examined on an Agilent 2100 Bioanalyzer (Agilent Technologies, Palo Alto, CA) (Additional file 15: Figure S9). The sequencing libraries were constructed as previously described [20]. Briefly, sRNAs of 15–30 nt in length were isolated using 15% PAGE (7 M urea). After ligation to both 5’ and 3’ RNA adaptors, the purified RNAs were reverse transcribed using primers with partial complementarity to the adaptors. The DNA pool amplified from the first-strand cDNA was subsequently sequenced using Hiseq 2000 (Illumina, San Diego, CA) at the Beijing Genomics Institute (BGI), Shenzhen.

### Analysis of sequencing data

Sequences were trimmed to eliminate low-quality reads and other contaminants from the adaptor tags. Repeats and protein coding sequences derived small RNAs were identified using a BLAST search against the annotated *G. raimondii* genome (http://cgp.genomics.org.cn/page/species/index.jsp). The rRNA, tRNA, snRNA, and snoRNA sequences were analyzed with a BLAST search against Sanger Rfam data (http://ftp.sanger.ac.uk/pub/databases/Rfam/). Known miRNA family sequences were analyzed through BLAST analysis against miRBase (version 19, http://microrna.sanger.ac.uk). Sequences that were identical or related to known miRNA sequences (two or fewer nucleotide substitutions) were considered as known miRNAs (Additional file 1: Table S1). Then, the *G. raimondii* (http://cgp.genomics.org.cn/page/species/index.jsp) sequences matching known miRNAs, rRNA, tRNA, snRNA, and snoRNA (Table 1) were obtained using the BLAST method. To identify novel miRNAs, the remaining sequences were mapped to the cotton nucleotide database, including the cotton genome survey sequences (GSS) from NCBI, ESTs from the cotton gene index (CGI, http://occams.dfci.harvard.edu/pub/bio/tgi/data/Gossypium/CGI.release_11.zip), and the *G. raimondii* genome (http://cgp.genomics.org.cn/page/species/index.jsp). Potential pre-miRNAs and secondary structures from the corresponding ESTs and genomic sequences were examined using mireap_0.2 (http://sourceforge.net/projects/mireap). After this analysis, candidate miRNAs that satisfied the following rules were accepted as novel miRNAs: (1) The set of reads from the candidate miRNA locus should account for more than 95% of all the precursor-mapped small RNA reads, and reliable candidate miRNA reads (five reads at least) should account for more than 75% of the corresponding set of reads; (2) the miRNA* reads should have two-nucleotide 3’ overhangs; or (3) the base-paring mismatch between the miRNA and the other arm of the hairpin, or between the miRNA* sequence and the other arm, should be less than five nt in length, meanwhile, no asymmetric bulges larger than two nucleotides and no more than two asymmetric bulges should be present within the miRNA/miRNA* duplex [27]. A previously reported strategy for TAS3 homolog identification was used to identify the cotton TAS3 locus [60].

### Identification of elongation-related miRNAs and target prediction

The abundances of known and novel miRNAs in each library were reported as normalized reads (reads per ten million, RPTM). The miRNA families with reads greater than 100 RPTM in at least one library were subjected to Student’s *t*-test to assess the statistical significance of the differences between the highest and lowest abundance levels of each miRNA, as previously described [20]. miRNAs with statistically significant differences in expression were considered cotton fiber elongation-related miRNAs. A complete linkage hierarchical cluster analysis of fiber elongation-related miRNAs in cotton fibers (5–20 dpa) was performed through a comparison of the RPTM of each miRNA to the average value in the four libraries [61], and the fiber elongation-related miRNA families were further subjected to target prediction.

The psRNATarget web server (http://plantgrn.noble.org/psRNATarget/) was used for target prediction. The most abundant sequence from each miRNA family served as the query, and CGI Release 11 served as the database. To annotate the potential target ESTs, a BLASTx search was performed against the NCBI non-redundant protein database of flowering plants (tax id: 3398). The top three hits (P < 0.001) were chosen to annotate the potential function of each predicted target EST (Additional file 10: Table S4 and Additional file 11: Table S5).

### 5’-RACE and quantitative RT-PCR

To validate the predicted cotton miRNA target genes *in vivo*, RNA Ligase-Mediated 5’-RACE (RLM-RACE) was performed using the First Choice RLM-RACE Kit (Ambion, Austin, TX), as previously described [20]. Quantitative RT-PCR of targets was also performed as previously described [20]. Briefly, cDNA was synthesized from 2 μg of total RNA using the TaKaRa RNA PCR Kit (TaKaRa), and the mRNA for *UBQ10* was amplified in parallel reactions to normalize the cDNA quantity added to each reaction [62]. Quantitative RT-PCR of three mature miRNAs was assayed by stem–loop reverse transcription-PCR [63]. DNA-free RNA (2 μg) was reverse-transcribed using miRNA-specific stem-loop primers with the TaKaRa RNA PCR Kit (TaKaRa), and *U6* was used as an internal control miRNA. Quantitative RT–PCR was performed on an iCycler iQ5 Multicolor real-time PCR detection system (Bio-Rad) using the Power SYBR Green PCR MasterMix (Applied Biosystems). The Minimum Information for Publication of Quantitative Real-Time PCR Experiments (MIQE) guidelines were followed to ensure the reliability of the obtained results [37]. All primers are listed in Additional file 16: Table S7.

### Anthocyanin measurement

A previously described method [64] was used to measure the anthocyanin levels. The (A_535_-A_650_) per gram of cotton fiber was used to determine the amount of anthocyanin in the cotton fibers,. All experiments were repeated at least three times.

## Abbreviations

miRNAs: MicroRNAs; ARF: Auxin response factor; siRNA: Small interfering RNA; rasiRNAs: Repeat-associated siRNAs; tasiRNAs or TAS: *trans*-acting siRNAs; nat-siRNAs: natural antisense siRNAs; dpa: Days pre-/post-anthesis; nt: nucleotide; snRNA: small nuclear ribonucleic acid; snoRNA: small nucleolar RNA; sRNA: small RNAs; RPTM: Reads per ten million; CGI: Cotton Gene Index; GSS: Genome survey sequences; G. raimondii: *Gossypium raimondii* Ulbr.; G. hirsutum: *Gossypium hirsutum* L.; G. arboretum: *Gossypium arboretum* L.; G. herbaceum: *Gossypium herbaceum* L.; DCL: Dicer-like; RACE Rapid: Amplification of cDNA Ends; qRT-PCR: quantitative RT-PCR; SPL9: Squamosa promoter-binding protein-like 9; GHHB8: class III HD-Zip protein 8; ACCD: Acetyl-CoA carboxylase beta subunit; CaMBP: Calmodulin binding protein; LRR-RLK: Leucine-rich repeat receptor-like protein kinase; CIBP: Calcium ion binding protein; AGO1: Argonaute 1; GRF: Growth regulating factor; UBC: Ubiquitin-conjugating enzyme; IAA: Indole acetic acid.

## Competing interests

The authors declare no competing financial interests.

## Authors’ contributions

JYL conceived and designed the study. WX prepared the RNA and analyzed the sequencing data. ZW performed the tasiRNA analysis. XW and ZW performed qRT-PCR. ZW performed the anthocyanin measurement. WX, ZW, MD and YL performed the 5’ RACE. JYL, WX and ZW wrote the manuscript. All authors read and approved the final manuscript.

## Supplementary Material

Additional file 1: Table S1 The 79 known cotton miRNA families expressed in cotton fibers. Click here for file

Additional file 2: Table S2 The 213 known cotton miRNA precursors derived from ESTs (CGI11), genome survey sequences (GSS) and G. raimondii genome sequences. Click here for file

Additional file 3: Figure S1 The 139 novel stem-loop structures of known miRNA precursors in elongating cotton fibers. In most cases, the red highlighting indicates the miRNA sequence and the blue highlighting indicates the miRNA* sequence. For example, in ‘17-MIR159-[hbr-MIR159a MI0022053]’, the ‘17’ came from the “index” column in the Additional file 2; “MIR159” came from the “name” column in the Additional file 2; and [hbr-MIR159a MI0022053] was the homolog of the precursor in miRBase. *tcc*: *Theobroma cacao*; *ctr*: *Citrus trifoliate*; *hbr*: *Hevea brasiliensis*; *vvi*: *Vitis vinifera*; *ptc*: *Populus trichocarpa*; *mdm*: *Malus domestica*. Click here for file

Additional file 4: Table S3 The 257 novel miRNAs in cotton fibers. Click here for file

Additional file 5: Figure S2 The 314 Vienna RNA secondary structures of 257 novel miRNAs. The Vienna structures showed that the reliable candidate miRNA reads (at least five reads) accounted for more than 75% of the corresponding sets of reads according to rule (1) described in the “newly identified miRNAs in elongating cotton fibers” section. Click here for file

Additional file 6: Figure S3 The 310 stem-loop structures of novel miRNA precursors in cotton fibers. The stem-loop structure shows the miRNA* sequence, mismatches between the miRNA and the other arm of the hairpin, and the size and frequency of asymmetric bulges. There were 314 novel miRNA gene loci, as shown in Additional file 5. The two miRNA gene loci each for novel_mir_2455, GhmiRnJ, novel_mir_4037 and novel_mir_860 had the same precursor sequences, so there were 310 distinct stem-loop structures. Click here for file

Additional file 7: Figure S4 Genomic overview of 174 known and 311 novel GhmiRNA gene loci on the 13 *G. raimondii* chromosomes. There were 314 novel miRNA gene loci, as shown in Additional file 5. The three miRNA gene loci of GhmiRnA and GhmiRnB were not anchored on any of the 13 chromosomes of the *G. raimondii* genome, so 311 novel miRNA gene loci were mapped on the 13 *G. raimondii* chromosomes. Click here for file

Additional file 8: Figure S5 Quantitative RT-PCR analysis of GhmiR156, GhmiR167 and GhmiR168. The data represent the mean values ± SD of three replicates. *U6* was used as a reference gene. Types A, B and C are shown in red, yellow, and blue, respectively, in Figure 2A. Click here for file

Additional file 9: Figure S6 Size distribution of cotton ta-siRNA generated from the DW503626 gene. Click here for file

Additional file 10: Table S4 Target prediction for novel cotton fiber elongation-related miRNAs. Click here for file

Additional file 11: Table S5 Target prediction for known cotton fiber elongation-related miRNAs. Click here for file

Additional file 12: Figure S7 GhSPL9, a target of GhmiR156/157, may positively regulate anthocyanin synthesis in cotton fiber. (A) Quantitative RT-PCR analysis of *GhDFR, GhANS, and GhF3H* at different fiber developmental stages. Error bars indicate the ± SD of three replicates. (B) The anthocyanin content in the cotton fiber at different development stages. Error bars represent the SD. Click here for file

Additional file 13: Figure S8 Quantitative RT-PCR analysis of *LRR-RLK* in the elongating fibers of *G. hirsutum* and *G. arboretum*. Click here for file

Additional file 14: Table S6 Normalized abundances of miRNAs and tasiRNA in fibers and seeds at 15 dpa. Click here for file

Additional file 15: Figure S9 RNA integrity of samples from 5, 10, 15 and 20 dpa fibers and 15 dpa seeds. Right: Electropherograms of the samples, as generated by the Agilent 2100 Bioanalyzer. The x-axis indicates the size of the nucleic acid, and the y-axis indicates the fluorescence. The red and black arrowheads indicate the 28S and 18S ribosomal RNA peaks, respectively. The RIN and 28S to 18S ratio are also shown in the image. RIN: RNA integrity number; FU: Fluorescence units. Click here for file

Additional file 16: Table S7 The primers used in this study. Click here for file
