# Supplementary material for: Genome-wide analysis of small RNAs reveals eight fiber elongation-related and 257 novel microRNAs in elongating cotton fiber cells
Source: BMC Genomics. 2013 Sep 17;14:629. doi: 10.1186/1471-2164-14-629 (PMC3849097; doi:10.1186/1471-2164-14-629)
Supplement: Additional file 1: Table S1 — The 79 known cotton miRNA families expressed in cotton fibers. [file 1471-2164-14-629-S1.docx]

**Additional Table S1:**

**The 79 known cotton miRNA families expressed in cotton fibers**

| **miRNA family** | **Sequence^a^ (5’ to 3’)** | **Length (nt)** | **Reads in fibers (dpa)^b^** | | | | **miRNA*^k^** |
| --- | --- | --- | --- | --- | --- | --- | --- |
|  |  |  | **5** | **10** | **15** | **20** |  |
| 1310 | AGGCAUCGGGGGCGCAACGCCCU | 24 | 9 | 13 | 36 | 195 |  |
| 156/157 | UUGACAGAAGAUAGAGAGCAC | 21 | 27154 | 73229 | 92994 | 152342 | Y |
| 158 | UCCCAAAUGUAGACAAAGCA**^e^** | 20 | 0 | 1 | 1 | 0 |  |
| 159/319 | UUUGGAUUGAAGGGAGCUCUA | 21 | 640 | 1200 | 2710 | 2719 | Y |
| 159a.2 | CUUCCAUAUCUCAGGAGCUUC | 21 | 2 | 14 | 4 | 3 |  |
| 160-3p | GCGUAUGAGGAGCCAUGCAUG | 21 | 76 | 96 | 49 | 57 |  |
| 160-5p | UGCCUGGCUCCCUGUAUGCCA | 21 | 9 | 6 | 6 | 24 | Y |
| 162-3p | UCGAUAAACCUCUGCAUCCAG**^g^** | 21 | 42 | 103 | 68 | 42 |  |
| 162-5p | UGGAGGCAGCGGUUCAUCGAUC | 22 | 37 | 89 | 46 | 49 | Y |
| 164 | UGGAGAAGCAGGGCACGUGCA | 21 | 4497 | 6421 | 3936 | 1006 | Y |
| 165/166 | UCGGACCAGGCUUCAUUCCCC | 21 | 66205 | 111265 | 65860 | 42169 | Y |
| 167 | UGAAGCUGCCAGCAUGAUCUC**^d^** | 21 | 129754 | 274066 | 214214 | 40790 | Y |
| 168 | UCGCUUGGUGCAGGUCGGGAA | 21 | 6127 | 3742 | 2665 | 2448 |  |
| 169_1 | UAGCCAAGGAUGACUUGCCUG**^f^** | 21 | 24 | 58 | 85 | 41 | Y |
| 169_2-5p | CAGCCAAGGAUGACUUGCCGG | 21 | 56 | 47 | 38 | 26 | Y |
| 169_2-3p | GGCAGGUUGUCUUUGGCUACA | 21 | 24 | 49 | 60 | 29 |  |
| 171_1 | UGAUUGAGCCGUGCCAAUAUC | 21 | 71 | 89 | 85 | 49 | Y |
| 171_2 | CGAGCCGAAUCAAUAUCACUC | 21 | 9 | 45 | 43 | 58 | Y |
| 172 | AGAAUCCUGAUGAUGCUGCAG**^f^** | 21 | 2236 | 5238 | 2593 | 936 | Y |
| 2111-3p | GUCCUUGGGAUGCAGAUUACC | 21 | 4 | 12 | 8 | 19 |  |
| 2111-5p | UAAUCUGCAUCCUGAGGUUUG**^f^** | 21 | 6 | 27 | 20 | 3 | Y |
| 2118 | UUGCCGAUUCCACCCAUGCCUA | 22 | 76 | 195 | 142 | 164 |  |
| 2911 | GCCGGGGGACGGACUGGGAA | 22 | 370 | 675 | 1039 | 4003 |  |
| 2947 | UAUACCGUGCCCAUGACUGUAG | 22 | 498 | 737 | 659 | 465 |  |
| 2948-5p | UGUGGGAGAGUUGGGCAAGAAU | 22 | 317 | 404 | 340 | 243 | Y |
| 2949 | ACUUUUGAACUGGAUUUGCCGA | 22 | 174 | 288 | 219 | 183 |  |
| 2950 | UGGUGUGCAGGGGGUGGAAUA | 21 | 906 | 2252 | 1545 | 523 | Y |
| 3476 | UGAACUGGGUUUGUUGGCUGC | 21 | 2665 | 4027 | 2913 | 3140 | Y |
| 3711-3p | AGGCCCUCCUUCUAGCGCCA | 20 | 61 | 445 | 276 | 885 |  |
| 390 | AAGCUCAGGAGGGAUAGCGCC | 21 | 4375 | 2963 | 1746 | 1405 | Y |
| 393-3p^c^ | AUCAUGCGAUCCCUUCGGAAU | 21 | 76 | 372 | 334 | 422 |  |
| 393-5p | UCCAAAGGGAUCGCAUUGAUCU**^g^** | 22 | 14 | 59 | 84 | 58 | Y |
| 394 | UUGGCAUUCUGUCCACCUCG**^j^** | 20 | 2 | 2 | 12 | 5 |  |
| 395 | CUGAAGUGUUUGGGGGAACUC | 21 | 37 | 107 | 234 | 795 | Y |
| 3954 | UGGACAGAGUAAUCACGGUCG | 21 | 1112 | 1291 | 990 | 564 |  |
| 396-3p | GCUCAAGAAAGCUGUGGGAGA | 21 | 1358 | 1131 | 1628 | 515 |  |
| 396-5p | UUCCACAGCUUUCUUGAACUU | 21 | 93 | 548 | 934 | 440 | Y |
| 397 | UCAUUGAGUGCAGCGUUGAUG**^f^** | 21 | 616 | 691 | 443 | 370 |  |
| 398 | UGUGUUCUCAGGUCGCCCCUG | 21 | 0 | 0 | 2 | 2 |  |
| 399 | UGCCAAAGGAGAUUUGCCCGG | 21 | 200 | 201 | 260 | 118 | Y |
| 403 | UUAGAUUCACGCACAAACUCG | 21 | 4 | 13 | 19 | 19 |  |
| 408 | AUGCACUGCCUCUUCCCUGGC | 21 | 7 | 18 | 11 | 10 |  |
| 473/477 | ACUCUCCCUCAAGGGCUUCCC | 21 | 12 | 36 | 31 | 69 |  |
| 479 | CGUGAUAUUGGUUCGGCUCAUC | 22 | 26 | 54 | 91 | 106 |  |
| 482 | UUGCCUACUCCACCCAUGCCAC | 22 | 142 | 454 | 290 | 208 | Y |
| 5054 | CUCCCCACGGUGGGCGCCA | 19 | 0 | 0 | 0 | 1 |  |
| 5077 | GUUUCGCGUCGGGUUCACCA | 20 | 2 | 4 | 3 | 10 |  |
| 5083 | AGACUACAAUUAUCUGAUCAUU | 22 | 1 | 2 | 1 | 1 |  |
| 5139 | AACCUGGCUCUGAUACCA | 18 | 18 | 159 | 202 | 789 |  |
| 5538 | CUACUGAACUCAAUCACUUGCUGC**^i^** | 24 | 10 | 7 | 4 | 5 |  |
| 5745 | UUAAUUUAUAUAAAUCGUCAA | 21 | 0 | 1 | 0 | 1 |  |
| 528 | UGGAAGGGGCAUGCAGAGGAG | 21 | 0 | 1 | 1 | 0 |  |
| 530 | UGCAUUUGCACCUGCACCUUC | 21 | 12 | 23 | 24 | 29 |  |
| 535 | UGACAAUGAGAGAGAGCACGC | 21 | 1594 | 2565 | 2877 | 2392 |  |
| 5368 | AGGGACAGUCUCAGGUAGA | 19 | 0 | 2 | 0 | 4 |  |
| 6478 | CCGACCUUAGCUCAGUUGGUA | 21 | 77 | 280 | 319 | 548 |  |
| 6118-3p | UUUCCGAGGCCACCCAUUCCAG | 22 | 0 | 1 | 0 | 0 |  |
| 6300 | GUCGUUGUAGUAUAGUGGUG**^i^** | 20 | 98 | 965 | 587 | 1273 |  |
| 7484**^c^** | UUUGUAUAUUAGAUCAAAGAGCAA**^f^** | 24 | 23 | 23 | 18 | 8 |  |
| 7485**^c^** | AAAGACAUCUUUGAAUUCUUGGAG**^e^** | 24 | 7 | 6 | 5 | 4 |  |
| 7486**^c^** | AAGGAAGCGCUUUGUCCACGUGGA**^f^** | 24 | 33 | 28 | 13 | 11 |  |
| 7487**^c^** | AUACUCUUAUAGGACACUUGUUAA | 24 | 9 | 23 | 15 | 6 |  |
| 7490**^c^** | AGUCUAGAAAACUUCACUGACGGU | 24 | 8 | 6 | 6 | 3 |  |
| 7491**^c^** | UGGGAUCUUCGAGAGGAUUGAGCC**^e^** | 24 | 58 | 26 | 24 | 22 |  |
| 7492**^c^** | CUAUAGAACAUGAUCUUUAGCGG**^g^** | 23 | 185 | 70 | 65 | 77 |  |
| 7493**^c^** | UAUGAGGAGCCAUGCAUGUAU**^j^** | 21 | 10 | 18 | 5 | 2 |  |
| 7495**^c^** | UUACUUUAGAUGUCUCCUUCA | 21 | 305 | 1002 | 789 | 327 | Y |
| 7496**^c^** | AUGACCAAAUUGAUAGAAUGUGUA**^h^** | 24 | 13 | 33 | 16 | 11 |  |
| 7497**^c^** | ACAUGUGGACUGUCAUAUGGGUU**^h^** | 23 | 36 | 15 | 13 | 10 |  |
| 7498**^c^** | AUGGUGACACAUGGUAGUCUCACA | 24 | 29 | 17 | 15 | 10 |  |
| 7501**^c^** | AUAUCUGAUUCUGACACGAAAAAA | 24 | 17 | 29 | 19 | 7 |  |
| 7502 **^c^** | UUUUUAACAGUAGAAAAGGAUGAA | 24 | 0 | 2 | 3 | 0 |  |
| 7503**^c^** | AGAUCGAUGGCUGAACAAGUUAGA | 24 | 4 | 4 | 5 | 2 |  |
| 7504a**^c^** | UAUGAAACUGTGAUUCUACGUCAU | 23 | 8 | 3 | 4 | 3 |  |
| 7504b**^c^** | AGGAGGAAAAAUCUGAUUUGUCAU | 24 | 240 | 192 | 159 | 104 |  |
| 7505**^c^** | UUCAGAAACCAUCCCUUCCUU | 21 | 52 | 179 | 89 | 132 | Y |
| 7508**^c^** | CAAGAAAAGAAGUCGGGAGAG | 21 | 2153 | 4294 | 2598 | 2568 | Y |
| 7509**^c^** | UCAAAAGCACUUUUUGACAGCAAU**^g^** | 24 | 65 | 97 | 66 | 39 |  |
| 7511**^c^** | AGAAGUUUUGCAUGUGUAGCUGAG | 24 | 61 | 44 | 22 | 12 |  |
| 7512**^c^** | UGCUACUUGUAGUUAUGCAUG | 21 | 24 | 21 | 15 | 22 |  |
| 7513**^c^** | AAUCAGCCAGGAAUCGUUUGA | 21 | 226 | 282 | 199 | 311 | Y |
| 827 | UUAGAUGACCAUCAACAAACA | 21 | 2 | 10 | 8 | 3 |  |
| 828-5p | UCUUGCUCAAAUGAGUAUUCCA | 22 | 0 | 0 | 0 | 1 |  |
| 828-3p | AGAUGCUCAUUUAAGCAAGCAA | 22 | 2 | 1 | 1 | 1 |  |
| 858 | UUCGUUGUCUGUUCGACCUUG | 21 | 2 | 25 | 63 | 7 |  |
| 894 | GUUUCACGUCGGGUUCACCA | 20 | 9785 | 23398 | 30855 | 95903 |  |

a: Sequences in this column represent the most abundant miRNA sequences (i.e. representative miRNA sequence) in each miRNA family, as identified by Illumina sequencing.

b: Small RNA abundance is normalized to reads per ten million (RPTM) in every library, and miRNA reads include the number of defined miRNAs and variants with less than 3 nt of mismatch.

c: These known miRNAs were previously identified and will be updated in the next miRBase version [1].

d-j: The representative sequences (as shown in the sequence column) of certain miRNA families in the four small RNA libraries are different. The origins of the representative sequences are shown as follows: d: 20 dpa; e: except for 10 dpa; f: except for 20 dpa; g: 5 and 10 dpa; h:10 and 20 dpa; i: except for 15 dpa; j: 15 dpa.

k: Y indicates the precise miRNA* sequence (two-nucleotide 3’ overhangs).

**References:**

1. Wang ZM, Xue W, Dong CJ, Jin LG, Bian SM, Wang C, Wu XY, Liu JY: **A Comparative miRNAome Analysis Reveals Seven Fiber Initiation-Related and 36 Novel miRNAs in Developing Cotton Ovules**. *Mol Plant* 2012, **5**(4):889-900.
