# Supplementary material for: Genome-wide analysis of small RNAs reveals eight fiber elongation-related and 257 novel microRNAs in elongating cotton fiber cells
Source: BMC Genomics. 2013 Sep 17;14:629. doi: 10.1186/1471-2164-14-629 (PMC3849097; doi:10.1186/1471-2164-14-629)
Supplement: Additional file 2: Table S2 — The 213 known cotton miRNA precursors derived from ESTs (CGI11), genome survey sequences (GSS) and G. raimondii genome sequences. [file 1471-2164-14-629-S2.docx]

**Additional Table S2:**

**The 213 known cotton miRNA precursors derived from ESTs (CGI11), genome survey sequences (GSS), and *G. raimondii* genome sequences**

| **Index** | **Name** | **Origin** | **Location** | **Start** | **End** | **Strand** | **Mfe (kcal/mol)** | **Star^a^** | **Annotation^b^** | **miRNA sequence^c^** | **Member^d^** |
| --- | --- | --- | --- | --- | --- | --- | --- | --- | --- | --- | --- |
| 1 | MIR156 | *G. raimondii* | Chr1 | 30965080 | 30965203 | - | -61.10 | Y | gra-MIR157b | UUGACAGAAGAUAGAGAGCAC | 6-6 |
| 2 | MIR156 | *G. raimondii* | Chr1 | 33532986 | 33533110 | + | -69.50 | Y | ghr-MIR156d | _UGACAGAAGAGAGUGAGCAC | 6-1 |
| 3 | MIR156 | *G. raimondii* | Chr1 | 34056209 | 34056308 | + | -47.60 |  | tcc-MIR156g | _UGACAGAAGAGAGUGAGCAC | 6-1 |
| 4 | MIR156 | *G. raimondii* | Chr6 | 16131508 | 16131625 | - | -48.40 |  | gra-MIR157a | UUGACAGAAGAUAGAGAGCAC | 6-6 |
| 5 | MIR156 | *G. raimondii* | Chr7 | 2180292 | 2180496 | - | -74.10 |  |  | UUGACAGAAGAGAGAGAGCAC | 6-4 |
| 6 | MIR156 | *G. raimondii* | Chr8 | 2415194 | 2415317 | + | -56.80 | Y | gra-MIR157b | UUGACAGAAGAUAGAGAGCAC | 6-6 |
| 7 | MIR156 | *G. raimondii* | Chr8 | 25067854 | 25068051 | + | -61.24 |  |  | _UGACAGAAGAGAGAGAGCAC | 6-2 |
| 8 | MIR156 | *G. raimondii* | Chr9 | 2131081 | 2131181 | - | -53.30 | Y | tcc-MIR156g | UGACAGAAGAGAGUGAGCAC | 6-1 |
| 9 | MIR156 | *G. raimondii* | Chr9 | 3470288 | 3470397 | + | -53.30 | Y | ghr-MIR156a | UGACAGAAGAGAGUGAGCAC | 6-1 |
| 10 | MIR156 | *G. raimondii* | Chr9 | 39888861 | 39888990 | - | -46.81 |  | tcc-MIR156e | UUGACAGAAGAUAGAGAGCAC | 6-6 |
| 11 | MIR156 | *G. raimondii* | Chr10 | 9756656 | 9756772 | - | -58.30 |  | tcc-MIR156d | _UGACAGAAGAGAGGGAGCAC | 6-5 |
| 12 | MIR156 | *G. raimondii* | Chr11 | 2198481 | 2198762 | - | -67.70 |  |  | UUGACAGAAGAGAGAGAGCA | 6-3 |
| 13 | MIR156 | *G. raimondii* | Chr13 | 7486769 | 7486892 | + | -48.80 | Y |  | _UGACAGAAGAGAGUGAGCAC | 6-1 |
| 14 | MIR156 | *G. raimondii* | Chr13 | 9382546 | 9382645 | - | -45.90 | Y | tcc-MIR156g | _UGACAGAAGAGAGUGAGCAC | 6-1 |
| 15 | MIR159 | *G. raimondii* | Chr3 | 11969266 | 11969479 | + | -85.00 |  | ctr-MIR319 | UUGGACUGAAGGGAGCUCCC^f^ | 4-2 |
| 16 | MIR159 | *G. raimondii* | Chr6 | 42191477 | 42191685 | - | -81.87 | Y | hbr-MIR159a | UUUGGAUUGAAGGGAGCUCUA | 4-1 |
| 17 | MIR159 | *G. raimondii* | Chr8 | 2377807 | 2378009 | - | -92.45 | Y | hbr-MIR159a | UUUGGAUUGAAGGGAGCUCUA^g^ | 4-1 |
| 18 | MIR159 | *G. raimondii* | Chr8 | 2505580 | 2505776 | - | -104.20 |  |  | UUGGACUGAAGGGAGCUCCC | 4-2 |
| 19 | MIR159 | *G. raimondii* | Chr8 | 7780287 | 7780465 | + | -86.10 |  | vvi-MIR319c | UUGGACUGAAGGGAGCUCCC | 4-2 |
| 20 | MIR159 | *G. raimondii* | Chr12 | 23814359 | 23814566 | + | -87.00 |  | ptc-MIR319f | UUGGACUGAAGGGAGCUCCUU | 4-3 |
| 21 | MIR160 | *G. raimondii* | Chr4 | 29259636 | 29259756 | + | -59.50 | Y | mdm-MIR160c | UGCCUGGCUCCCUGUAUGCCA | 2-1 |
| 22 | MIR160 | *G. raimondii* | Chr6 | 34537043 | 34537146 | - | -55.00 | Y | tcc-MIR160b | UGCCUGGCUCCCUGUAUGCCA | 2-1 |
| 23 | MIR160 | *G. raimondii* | Chr9 | 7618325 | 7618428 | - | -53.30 | Y | tcc-MIR160a | UGCCUGGCUCCCUGAAUGCCA | 2-2 |
| 24 | MIR160 | *G. raimondii* | Chr9 | 34426956 | 34427076 | - | -54.20 | Y | mdm-MIR160e | UGCCUGGCUCCCUGUAUGCCA | 2-1 |
| 25 | MIR160 | *G. raimondii* | Chr11 | 41416945 | 41417065 | - | -54.09 | Y | mdm-MIR160c | UGCCUGGCUCCCUGUAUGCCA | 2-1 |
| 26 | MIR160 | *G. raimondii* | Chr13 | 36788029 | 36788141 | - | -63.80 |  |  | UGCCUGGCUCCCUGAAUGCCA | 2-2 |
| 27 | MIR162_1 | *G. raimondii* | Chr7 | 23217211 | 23217340 | - | -47.30 | Y | ghr-MIR162a | UGGAGGCAGCGGUUCAUCGAUC | 1-1 |
| 28 | MIR164 | *G. raimondii* | Chr6 | 41309748 | 41309902 | + | -60.45 | Y |  | UGGAGAAGCAGGGCACGUGCA | 2-1 |
| 29 | MIR164 | *G. raimondii* | Chr7 | 45742332 | 45742442 | + | -50.50 | Y |  | UGGAGAAGCAGGGCACGUGCA | 2-1 |
| 30 | MIR164 | *G. raimondii* | Chr8 | 32008251 | 32008340 | - | -34.73 |  |  | UGGAGAAGCAGGGCACGUGCA | 2-1 |
| 31 | MIR164 | *G. raimondii* | Chr11 | 6568914 | 6569008 | - | -41.30 |  |  | UGGAGAAGCAGGGCACAUGCU | 2-2 |
| 32 | MIR166 | *G. raimondii* | Chr1 | 16234591 | 16234757 | + | -70.10 | Y |  | UCGGACCAGGCUUCAUUCCCC | 5-1 |
| 33 | MIR166 | *G. raimondii* | Chr2 | 6115515 | 6115652 | + | -60.73 | Y |  | UCGGACCAGGCUUCAUUCCCA | 5-3 |
| 34 | MIR166 | *G. raimondii* | Chr3 | 10358379 | 10358547 | + | -62.24 | Y |  | UCGGACCAGGCUUCAUUCCCC | 5-1 |
| 35 | MIR166 | *G. raimondii* | Chr5 | 42580582 | 42580730 | + | -43.40 | Y |  | UCGGACCAGGCUUCAUUCCCC | 5-1 |
| 36 | MIR166 | *G. raimondii* | Chr5 | 42580678 | 42580849 | + | -85.32 | Y | tcc-MIR166b | UCGGACCAGGCUUCAUUCCCG | 5-4 |
| 37 | MIR166 | *G. raimondii* | Chr6 | 45491778 | 45491884 | - | -57.00 | Y |  | UCGGACCAGGCUUCAUUCCCC | 5-1 |
| 38 | MIR166 | *G. raimondii* | Chr7 | 2320376 | 2320474 | + | -50.00 |  |  | UCGGACCAGGCUUCAUUCCUC | 5-5 |
| 39 | MIR166 | *G. raimondii* | Chr7 | 37876157 | 37876273 | + | -56.20 | Y | tcc-MIR166a | UCGGACCAGGCUUCAUUCCCC | 5-1 |
| 40 | MIR166 | *G. raimondii* | Chr7 | 41363884 | 41363987 | + | -47.80 |  |  | UCGGACCAGGCUUCAUUCCCC | 5-1 |
| 41 | MIR166 | *G. raimondii* | Chr8 | 10709170 | 10709358 | + | -62.60 |  |  | UCGGACCAGGCUUCAUUCCCC | 5-1 |
| 42 | MIR166 | *G. raimondii* | Chr8 | 34947056 | 34947222 | - | -65.39 |  | ghr-MIR166b | UCGGACCAGGCUUCAUUCCCC | 5-1 |
| 43 | MIR166 | *G. raimondii* | Chr13 | 13338471 | 13338589 | + | -55.90 |  |  | UCUCGGACCAGGCUUCAUUCC^e^ | 5-2 |
| 44 | MIR166 | *G. raimondii* | Chr13 | 13346009 | 13346127 | + | -55.90 |  |  | UCUCGGACCAGGCUUCAUUCC^e^ | 5-2 |
| 46 | MIR167_1 | *G. raimondii* | Chr7 | 41733185 | 41733272 | - | -45.30 |  | ghr-MIR167b | UGAAGCUGCCAGCAUGAUCUA | 3-1 |
| 47 | MIR167_1 | *G. raimondii* | Chr7 | 41899464 | 41899554 | - | -38.90 | Y | tcc-MIR167c | UGAAGCUGCCAGCAUGAUCUUA | 3-3 |
| 48 | MIR167_1 | *G. raimondii* | Chr8 | 64215676 | 64215782 | - | -50.20 |  | ghr-MIR167a | UGAAGCUGCCAGCAUGAUCU(C) | 3-2 |
| 49 | MIR167_1 | *G. raimondii* | Chr9 | 19090415 | 19090771 | + | -110.30 |  |  | UGAAGCUGCCAGCAUGAUCU(C) | 3-2 |
| 50 | MIR167_1 | *G. raimondii* | Chr10 | 33840357 | 33840721 | - | -109.50 |  |  | UGAAGCUGCCAGCAUGAUCU(C) | 3-2 |
| 51 | MIR167_1 | *G. raimondii* | Chr11 | 3913611 | 3913860 | - | -75.80 |  |  | UGAAGCUGCCAGCAUGAUCU(C) | 3-2 |
| 52 | MIR169_1 | *G. raimondii* | Chr5 | 35654669 | 35654838 | - | -74.10 | Y |  | UAGCCAAGGAUGACUUGCCUG | 6-1 |
| 53 | MIR169_1 | *G. raimondii* | Chr7 | 50295200 | 50295442 | - | -94.20 | Y |  | UAGCCAAGGAUGACUUGCCUG | 6-1 |
| 54 | MIR169_1 | *G. raimondii* | Chr7 | 50295394 | 50295510 | - | -50.90 | Y |  | UAGCCAAGAAUGACUUGCCUG(C) | 6-2 |
| 55 | MIR169_1 | *G. raimondii* | Chr7 | 52355718 | 52355829 | - | -49.30 | Y |  | UAGCCAAGGAUGACUUGCCU_ | 6-3 |
| 56 | MIR169_1 | *G. raimondii* | Chr8 | 14095773 | 14095890 | + | -49.40 | Y |  | UAGCCAAGGAUGACUUGCCU_ | 6-3 |
| 57 | MIR169_1 | *G. raimondii* | Chr9 | 10666126 | 10666226 | + | -39.60 |  |  | UAGCCAAGAAUGACUUGCCU_ | 6-4 |
| 58 | MIR169_1 | *G. raimondii* | Chr9 | 10671286 | 10671420 | + | -50.70 |  |  | GGCAGUCACCUUGGCUAAUC^e^ | 6-5 |
| 59 | MIR169_1 | *G. raimondii* | Chr9 | 10675183 | 10675311 | + | -56.30 | Y |  | _AGCCAAGGAUGACUUGCCUG(C) | 6-6 |
| 60 | MIR169_1 | *G. raimondii* | Chr10 | 36015276 | 36015407 | + | -57.20 | Y | ghr-MIR169;ghb-MIR169a | UAGCCAAGGAUGACUUGCCUG | 6-1 |
| 61 | MIR169_1 | *G. raimondii* | Chr10 | 36015347 | 36015582 | + | -86.60 | Y | ghb-MIR169a | UAGCCAAGAAUGACUUGCCU_ | 6-4 |
| 62 | MIR169_2 | *G. raimondii* | Chr6 | 47272296 | 47272451 | + | -59.73 |  |  | (GG)UAGCCAAGGAUGACUUGCCU_ | 5-2 |
| 63 | MIR169_2 | *G. raimondii* | Chr7 | 1394857 | 1395055 | + | -66.16 | Y |  | CAGCCAAGGAUGACUUGCCGG | 5-1 |
| 64 | MIR169_2 | *G. raimondii* | Chr7 | 11804070 | 11804167 | - | -52.20 | Y |  | UAGCCAAGGAUGACUUGCCGA | 5-3 |
| 65 | MIR169_2 | *G. raimondii* | Chr7 | 49713326 | 49713500 | - | -57.40 |  |  | CAGCCAAGGAUGACUUGCCGG | 5-1 |
| 66 | MIR169_2 | *G. raimondii* | Chr9 | 290075 | 290207 | - | -60.73 |  |  | (UU)GAGUCAAGAAUGACUUGCCG_ | 5-4 |
| 67 | MIR169_2 | *G. raimondii* | Chr13 | 25072675 | 25072884 | + | -67.80 |  |  | CAGCCAAGGAUGAUUUGCCGG | 5-5 |
| 68 | MIR171_1 | *G. raimondii* | Chr1 | 2462632 | 2462730 | - | -53.90 |  | tcc-MIR171g | UGAUUGAGCCGUGCCAAUAUC | 1-1 |
| 69 | MIR171_1 | *G. raimondii* | Chr3 | 25589774 | 25589903 | - | -43.56 | Y |  | UGAUUGAGCCGUGCCAAUAUC | 1-1 |
| 70 | MIR171_1 | *G. raimondii* | Chr8 | 14668100 | 14668198 | - | -43.00 | Y | tcc-MIR171f | UGAUUGAGCCGUGCCAAUAUC | 1-1 |
| 71 | MIR171_1 | *G. raimondii* | Chr8 | 42114286 | 42114427 | - | -57.10 | Y |  | UGAUUGAGCCGUGCCAAUAUC | 1-1 |
| 72 | MIR171_1 | *G. raimondii* | Chr9 | 22217694 | 22217809 | + | -46.80 | Y |  | UGAUUGAGCCGUGCCAAUAUC | 1-1 |
| 73 | MIR171_1 | *G. raimondii* | Chr10 | 37503995 | 37504111 | - | -48.40 | Y | tcc-MIR171d | UGAUUGAGCCGUGCCAAUAUC | 1-1 |
| 74 | MIR171_1 | *G. raimondii* | Chr11 | 963517 | 963632 | + | -54.67 | Y |  | UGAUUGAGCCGUGCCAAUAUC | 1-1 |
| 75 | MIR171_1 | *G. raimondii* | Chr11 | 10248320 | 10248415 | + | -48.00 |  | tcc-MIR171g | UGAUUGAGCCGUGCCAAUAUC | 1-1 |
| 76 | MIR171_1 | *G. raimondii* | Chr12 | 14661055 | 14661155 | + | -53.90 | Y | tcc-MIR171g | UGAUUGAGCCGUGCCAAUAUC | 1-1 |
| 77 | MIR171_2 | *G. raimondii* | Chr4 | 22314919 | 22315018 | + | -38.60 | Y | tcc-MIR172e | CGAGCCGAAUCAAUAUCACUC | 2-1 |
| 78 | MIR172 | *G. raimondii* | Chr7 | 31055941 | 31056102 | + | -70.47 | Y |  | GGAAUCUUGAUGAUGCUGCAU | 5-3 |
| 79 | MIR172 | *G. raimondii* | Chr8 | 25740646 | 25740776 | - | -59.30 | Y |  | AGAAUCUUGAUGAUGCUGCAU | 5-2 |
| 80 | MIR172 | *G. raimondii* | Chr9 | 11991989 | 11992105 | - | -57.80 |  |  | UGAAUCUUGAUGAUGCUGCAU | 5-5 |
| 81 | MIR172 | *G. raimondii* | Chr9 | 12982000 | 12982183 | - | -59.52 | Y |  | AGAAUCUUGAUGAUGCUGCAU | 5-2 |
| 82 | MIR172 | *G. raimondii* | Chr11 | 44104702 | 44104847 | + | -54.60 | Y |  | AGAAUCUUGAUGAUGCUGCAG | 5-4 |
| 83 | MIR172 | *G. raimondii* | Chr11 | 44110983 | 44111103 | + | -56.60 | Y | ghr-MIR172 | AGAAUCCUGAUGAUGCUGCAG | 5-1 |
| 84 | MIR172 | *G. raimondii* | Chr11 | 44115433 | 44115573 | + | -70.30 | Y | ghr-MIR172 | AGAAUCUUGAUGAUGCUGCAG | 5-4 |
| 85 | MIR172 | *G. raimondii* | Chr13 | 2369026 | 2369134 | - | -54.60 |  |  | UGAAUCUUGAUGAUGCUGCAU | 5-5 |
| 86 | MIR172 | *G. raimondii* | Chr13 | 41641129 | 41641300 | - | -61.93 | Y |  | AGAAUCUUGAUGAUGCUGCAG | 5-4 |
| 87 | MIR2111 | *G. raimondii* | Chr6 | 40687819 | 40687906 | + | -49.80 |  |  | UAAUCUGCAUCCUGAGGUUU | 1-1 |
| 88 | MIR2111 | *G. raimondii* | Chr8 | 50879399 | 50879490 | + | -49.60 | Y |  | UAAUCUGCAUCCUGAGGUUUG | 1-1 |
| 89 | MIR2947 | *G. raimondii* | Chr8 | 62058576 | 62058681 | + | -52.32 |  | gar-MIR2947 | UAUACCGUGCCCAUGACUGUAG | 1-1 |
| 90 | MIR2950 | *G. raimondii* | Chr7 | 25911192 | 25911292 | - | -47.40 | Y |  | UGGUGUGCAGGGGGUGGAAUA | 1-1 |
| 91 | MIR2950 | *G. raimondii* | Chr8 | 44905574 | 44905688 | - | -51.10 | Y | ghr-MIR2950 | UGGUGUGCAGGGGGUGGAAUA | 1-1 |
| 92 | MIR3476 | *G. raimondii* | Chr3 | 1163514 | 1163628 | + | -43.20 | Y | ghr-MIR3476 | UGAACUGGGUUUGUUGGCUGC | 1-1 |
| 93 | MIR390 | *G. raimondii* | Chr2 | 189943 | 190080 | - | -57.20 | Y | ghr-MIR390a_b_c | AAGCUCAGGAGGGAUAGCGCC | 1-1 |
| 94 | MIR390 | *G. raimondii* | Chr2 | 36177582 | 36177711 | + | -57.30 | Y |  | AAGCUCAGGAGGGAUAGCGCC | 1-1 |
| 95 | MIR390 | *G. raimondii* | Chr3 | 3160248 | 3160354 | - | -50.91 | Y |  | AAGCUCAGGAGGGAUAGCGCC | 1-1 |
| 96 | MIR390 | *G. raimondii* | Chr6 | 41222174 | 41222264 | + | -41.90 | Y |  | AAGCUCAGGAGGGAUAGCGCC | 1-1 |
| 97 | MIR393 | *G. raimondii* | Chr2 | 8178873 | 8179031 | - | -41.04 | Y |  | UCCAAAGGGAUCGCAUUGAUCC | 2-2 |
| 98 | MIR393 | *G. raimondii* | Chr8 | 3294115 | 3294275 | - | -64.10 | Y | ghr-MIR393 | UCCAAAGGGAUCGCAUUGAUCU | 2-1 |
| 99 | MIR393 | *G. raimondii* | Chr12 | 19628926 | 19629035 | + | -45.21 | Y |  | UCCAAAGGGAUCGCAUUGAUCC | 2-2 |
| 100 | MIR393 | *G. raimondii* | Chr13 | 41736458 | 41736579 | + | -56.30 | Y |  | UCCAAAGGGAUCGCAUUGAUCC | 2-2 |
| 101 | MIR394 | *G. raimondii* | Chr2 | 5949814 | 5949931 | - | -38.72 |  | ghr-MIR394b | UUGGCAUUCUGUCCACCUCC | 2-2 |
| 102 | MIR394 | *G. raimondii* | Chr2 | 32070519 | 32070634 | + | -46.60 |  |  | UUGGCAUUCUGUCCACCUCC | 2-2 |
| 103 | MIR394 | *G. raimondii* | Chr8 | 22437368 | 22437446 | - | -39.00 |  |  | UUGGCAUUCUGUCCACCUCC | 2-2 |
| 104 | MIR394 | *G. raimondii* | Chr9 | 22164883 | 22164987 | - | -45.00 |  | ghr-MIR394a | UUGGCAUUCUGUCCACCUCC | 2-2 |
| 105 | MIR394 | *G. raimondii* | Chr9 | 30830950 | 30831056 | - | -49.30 |  | ghr-MIR394a | UUGGCAUUCUGUCCACCUCG | 2-1 |
| 106 | MIR395 | *G. raimondii* | Chr2 | 1076309 | 1076417 | + | -48.70 | Y |  | CUGAAGUGUUUGGGGGAACUC | 1-1 |
| 107 | MIR395 | *G. raimondii* | Chr13 | 23227929 | 23228054 | - | -52.01 |  |  | CUGAAGUGUUUGGGGGAACUC | 1-1 |
| 108 | MIR395 | *G. raimondii* | Chr13 | 24398722 | 24398826 | - | -51.20 | Y |  | CUGAAGUGUUUGGGGGAACUC | 1-1 |
| 109 | MIR3954 | *G. raimondii* | Chr1 | 4632809 | 4632939 | + | -32.70 |  |  | UGGAC(G)AGAG_AAUCACGGUCG | 2-1 |
| 110 | MIR3954 | *G. raimondii* | Chr5 | 740254 | 740343 | + | -35.80 |  |  | (U)UGGACAGAGUAAUCACGGUCG | 2-2 |
| 111 | MIR3954 | *G. raimondii* | Chr6 | 45508596 | 45508723 | - | -44.22 |  |  | (U)UGGACAGAGUAAUCACGGUCG | 2-2 |
| 112 | MIR396 | *G. raimondii* | Chr4 | 533538 | 533651 | + | -50.50 | Y |  | UUCCACAGCUUUCUUGAACUG | 3-1 |
| 113 | MIR396 | *G. raimondii* | Chr4 | 555201 | 555348 | - | -56.71 | Y |  | UUCCACAGCUUUCUUGAACUU | 3-2 |
| 114 | MIR396 | *G. raimondii* | Chr5 | 38503763 | 38503865 | - | -49.20 | Y |  | UUCCACGGCUUUCUUGAACUU | 3-3 |
| 115 | MIR396 | *G. raimondii* | Chr8 | 28077001 | 28077160 | - | -58.51 | Y |  | UUCCACAGCUUUCUUGAACUU | 3-2 |
| 116 | MIR396 | *G. raimondii* | Chr13 | 5796697 | 5796835 | + | -50.60 | Y | ghr-MIR396a_b | UUCCACAGCUUUCUUGAACUG | 3-1 |
| 117 | MIR396 | *G. raimondii* | Chr13 | 5803606 | 5803763 | - | -59.11 | Y |  | UUCCACAGCUUUCUUGAACUU | 3-2 |
| 118 | MIR397 | *G. raimondii* | Chr9 | 21023020 | 21023108 | - | -48.40 |  |  | UCAUUGAGUGCAGCGUUGAUG | 2-1 |
| 45 | MIR397 | *G. raimondii* | Chr11 | 1754349 | 1754430 | - | -32.90 |  |  | ___UUGAGUGCAGCGUUGAUG(AGC) | 2-2 |
| 119 | MIR399 | *G. raimondii* | Chr6 | 6537492 | 6537601 | - | -50.00 |  |  | CGCCAAAGGAGAGUUGCCCUU | 6-6 |
| 120 | MIR399 | *G. raimondii* | Chr8 | 2929964 | 2930099 | - | -56.50 |  |  | UGCCAAAGGAGAGUUGCCCUG | 6-1 |
| 121 | MIR399 | *G. raimondii* | Chr8 | 2932571 | 2932670 | - | -47.90 |  |  | UGCCAAAGGAGAGUUGCCCUG | 6-1 |
| 122 | MIR399 | *G. raimondii* | Chr8 | 8473216 | 8473311 | + | -42.70 |  | ghr-MIR399c | CAGGGCUUCUCUGCAUUGGCA^i^ | 6-4 |
| 123 | MIR399 | *G. raimondii* | Chr9 | 19883201 | 19883330 | + | -51.50 |  |  | UGCCAAAGGAGAUUUGCCCGG | 6-3 |
| 124 | MIR399 | *G. raimondii* | Chr9 | 19896838 | 19896936 | - | -34.90 |  |  | UGCCAAAGGAGAAUUGCCCUG | 6-2 |
| 125 | MIR399 | *G. raimondii* | Chr10 | 30719862 | 30720013 | - | -45.92 | Y |  | UGCCAAAGGAGAUUUGUCCGG | 6-5 |
| 126 | MIR403 | *G. raimondii* | Chr7 | 45110828 | 45110939 | - | -36.80 |  | tcc-MIR403a | UUAGAUUCACGCACAAACUCG | 1-1 |
| 127 | MIR403 | *G. raimondii* | Chr9 | 41510559 | 41510665 | - | -46.00 |  |  | UUAGAUUCACGCACAAACUCG | 1-1 |
| 128 | MIR408 | *G. raimondii* | Chr7 | 44607389 | 44607507 | - | -51.80 |  |  | AUGCACUGCCUCUUCCCUGGC | 1-1 |
| 129 | MIR477 | *G. raimondii* | Chr5 | 38245187 | 38245300 | + | -61.30 |  |  | ACUCUCCCUCAAGGGCUUCCCC | 6-1 |
| 130 | MIR477 | *G. raimondii* | Chr5 | 38249934 | 38250040 | + | -59.90 |  |  | ACUCUCCCUCAAGGGCUUCCCC | 6-1 |
| 131 | MIR477 | *G. raimondii* | Chr7 | 53648673 | 53648801 | - | -48.40 |  |  | ACUCUCCCUCAAGGGCUUCCG_ | 6-2 |
| 132 | MIR477 | *G. raimondii* | Chr7 | 53648750 | 53648863 | - | -53.80 |  |  | ACUCUCCCUCAAGGGCUUCCUG | 6-3 |
| 133 | MIR477 | *G. raimondii* | Chr8 | 7613866 | 7613954 | - | -41.34 |  |  | ACUCUCCCUCAAGGGCUUCUG_ | 6-4 |
| 134 | MIR477 | *G. raimondii* | Chr8 | 9765202 | 9765287 | + | -36.50 |  |  | ACUCUCUUCCAAAGGCUUCAA_ | 6-5 |
| 135 | MIR477 | *G. raimondii* | Chr9 | 7088772 | 7088873 | - | -43.14 |  |  | AAUCUCCCUCAAACGCUUCCAG | 6-6 |
| 136 | MIR482 | *G. raimondii* | Chr3 | 19868022 | 19868139 | - | -43.70 |  |  | UCUUCCCAACACCUCCCAUACC | 7-2 |
| 137 | MIR482 | *G. raimondii* | Chr3 | 19868201 | 19868325 | - | -46.71 |  |  | UUGCCAGCUCCGCCCAUUCCAA | 7-3 |
| 138 | MIR482 | *G. raimondii* | Chr5 | 34781561 | 34781678 | - | -43.70 |  |  | UCUUCCCAACACCUCCCAUACC | 7-2 |
| 139 | MIR482 | *G. raimondii* | Chr5 | 34781740 | 34781864 | - | -46.71 |  |  | UUGCCAGCUCCGCCCAUUCCAA | 7-3 |
| 140 | MIR482 | *G. raimondii* | Chr6 | 21992955 | 21993056 | + | -49.09 |  | gra-MIR482 | UUUCCAAUUCCUCCCAUUCCAC | 7-4 |
| 141 | MIR482 | *G. raimondii* | Chr7 | 46474621 | 46474722 | - | -43.06 |  | gra-MIR482 | UUUCCAAUUCCUCCCAUUCCAC | 7-4 |
| 142 | MIR482 | *G. raimondii* | Chr8 | 15397392 | 15397493 | + | -50.09 |  | gra-MIR482 | UUUCCAAUUCCUCCCAUUCCAC | 7-4 |
| 143 | MIR482 | *G. raimondii* | Chr8 | 43787745 | 43787846 | - | -46.79 |  | gra-MIR482 | UUUCCAAUUCCUCCCAUUCCAC | 7-4 |
| 144 | MIR482 | *G. raimondii* | Chr12 | 7292313 | 7292418 | + | -45.40 |  | ghr-MIR482a | (UC)UUUCCUACUCCUCCCAUUCC__ | 7-5 |
| 145 | MIR482 | *G. raimondii* | Chr12 | 17610498 | 17610611 | + | -54.50 | Y | ghr-MIR2948 | UGUGGGAGAGUUGGGCAAGAAU | 7-6 |
| 146 | MIR482 | *G. raimondii* | Chr13 | 9097157 | 9097277 | - | -62.00 |  | ghr-MIR482b | UUGCCUACUCCACCCAUGCCAC | 7-1 |
| 147 | MIR482 | *G. raimondii* | Chr13 | 22268583 | 22268683 | - | -50.39 |  | gra-MIR482 | UUUCCAAUUCCUCCCAUUCCAC | 7-4 |
| 148 | MIR530 | *G. raimondii* | Chr9 | 13024665 | 13024806 | + | -49.62 |  |  | UGCAUUUGCACCUGCACCUUC | 1-1 |
| 149 | MIR535 | *G. raimondii* | Chr1 | 28759006 | 28759103 | - | -47.30 |  | tcc-MIR535 | UGACAACGAGAGAGAGCACGU | 1-1 |
| 150 | MIR535 | *G. raimondii* | Chr7 | 40293953 | 40294050 | + | -58.90 |  | tcc-MIR535 | UGACAAUGAGAGAGAGCACGC | 1-1 |
| 151 | MIR6118 | *G. raimondii* | Chr8 | 41287164 | 41287273 | + | -46.30 |  |  | UUCCCGAGGCCACCCAUUCCAG | 1-1 |
| 152 | MIR6300 | *G. raimondii* | Chr1 | 34366019 | 34366085 | - | -19.30 |  |  | GUCGUUGUAGUAUAGUGGUG | 2-1 |
| 153 | MIR6300 | *G. raimondii* | Chr9 | 3590627 | 3590695 | - | -23.40 |  |  | GUCGUUGUAGUAUAGUGGUG | 2-1 |
| 154 | MIR6300 | *G. raimondii* | Chr13 | 20448969 | 20449077 | + | -19.30 |  |  | GUUGUUGUAGUAUAGUGGUG | 2-2 |
| 155 | MIR6478 | *G. raimondii* | Chr9 | 11132439 | 11132663 | - | -36.33 |  |  | CCGACCUUAGCUCAGUUGGUA | 1-1 |
| 156 | MIR7486 | *G. raimondii* | Chr4 | 23411008 | 23411108 | + | -70.10 |  |  | UCCACGUAGGCAAAGCGCUUCCUU^e^ | 1-1 |
| 157 | MIR7486 | *G. raimondii* | Chr10 | 14419022 | 14419122 | - | -69.60 |  |  | UCCACGUAGGCAAAGCGCUUCCUU^e^ | 1-1 |
| 158 | MIR7495 | *G. raimondii* | Chr8 | 5654650 | 5654818 | - | -60.90 | Y |  | UUACUUUAGAUGUCUCCUUCA | 1-1 |
| 159 | MIR7496 | *G. raimondii* | Chr1 | 14032311 | 14032456 | - | -50.96 |  |  | AUGACCAAAUUGAUAGAAUGUGUA | 2-1 |
| 160 | MIR7496 | *G. raimondii* | Chr1 | 22895845 | 22895991 | - | -37.20 |  |  | AUGACCAAAUUGAUAGAAUGUGUA | 2-1 |
| 161 | MIR7496 | *G. raimondii* | Chr4 | 4048282 | 4048428 | - | -36.10 |  |  | AAGACCAAAUUGAUAGAAUGUGUA | 2-2 |
| 162 | MIR7497 | *G. raimondii* | Chr6 | 3015898 | 3016078 | + | -54.40 |  |  | ACAUGUGGACUGUCAUAUGGGUU | 1-1 |
| 163 | MIR7498 | *G. raimondii* | Chr13 | 44494972 | 44495089 | + | -59.20 |  |  | AUGGUGACACAUGGUAGUCUCACA | 1-1 |
| 164 | MIR7502 | *G. raimondii* | Chr6 | 46563472 | 46563617 | - | -43.90 |  |  | UUUUUAACAGUAGAAAAGGAUGAA | 1-1 |
| 165 | MIR7504a | *G. raimondii* | Chr2 | 2946928 | 2947136 | + | -86.30 |  |  | UAUGAAACUGUGAUUCUACGUCAU | 1-1 |
| 166 | MIR7504b | *G. raimondii* | Chr1 | 21810930 | 21811052 | - | -47.90 |  |  | AGGAGAAAAAAUCUGAUUUGUCAU | 3-1 |
| 167 | MIR7504b | *G. raimondii* | Chr4 | 29593700 | 29593825 | - | -61.40 |  |  | AGGAAGAAAAAUCUGAUUUGUCAU | 3-2 |
| 168 | MIR7504b | *G. raimondii* | Chr5 | 20159758 | 20159853 | + | -47.90 |  |  | AGGAAGAAAAAUCUGAUUUGUCAU | 3-2 |
| 169 | MIR7504b | *G. raimondii* | Chr7 | 1135708 | 1135833 | + | -51.71 |  |  | AGGAGAAAAAAUCUGAUUUGUCAU | 3-1 |
| 170 | MIR7504b | *G. raimondii* | Chr11 | 3915380 | 3915485 | + | -33.14 |  |  | AGGAGAAAAAAUCUGAUUUGUCAU | 3-1 |
| 171 | MIR7504b | *G. raimondii* | Chr12 | 13761875 | 13762001 | - | -32.60 |  |  | AAGAGGAAAAAUCUGAUUUGUCAU | 3-3 |
| 172 | MIR7508 | *G. raimondii* | Chr8 | 5714972 | 5715083 | - | -50.39 | Y |  | CAAGAAAAGAAGUCGGGAGAG | 1-1 |
| 173 | MIR7513 | *G. raimondii* | Chr10 | 44401906 | 44402008 | - | -37.24 | Y |  | AAUCAGCCAGGAAUCGUUUGA | 1-1 |
| 174 | MIR827_2 | *G. raimondii* | Chr11 | 39394836 | 39394978 | + | -42.30 |  | ghr-MIR827a_b_c | UUAGAUGACCAUCAACAAACA | 1-1 |
| 175 | MIR156 | CGI11 | TC275383 | 203 | 327 | - | -65.40 | Y | ghr-MIR156d | UGACAGAAGAGAGUGAGCAC | 6-1 |
| 176 | MIR156 | CGI11 | TC248903 | 2313 | 2517 | + | -77.90 |  |  | UUGACAGAAGAGAGAGAGCAC | 6-4 |
| 177 | MIR156 | CGI11 | TC271335 | 1048 | 1171 | - | -56.80 | Y | gra-MIR157b | UUGACAGAAGAUAGAGAGCAC | 6-6 |
| 178 | MIR156 | CGI11 | TC258549 | 856 | 973 | - | -48.40 |  | gra-MIR157a | UUGACAGAAGAUAGAGAGCAC | 6-6 |
| 179 | MIR159 | CGI11 | ES824206 | 590 | 686 | + | -46.10 |  |  | AGCUGCUUGGCUAUGGAUCCC^h^ | 4-4 |
| 180 | MIR162_1 | CGI11 | TC251997 | 186 | 315 | + | -45.00 |  | ghr-MIR162a | UGGAGGCAGCGGUUCAUCGAUC | 1-1 |
| 181 | MIR164 | CGI11 | DR461140 | 223 | 307 | - | -43.10 | Y | ghr-MIR164 | UGGAGAAGCAGGGCACGUGCA | 2-1 |
| 182 | MIR166 | CGI11 | DW502146 | 173 | 339 | + | -63.69 |  | ghr-MIR166b | UCGGACCAGGCUUCAUUCCCC | 5-1 |
| 183 | MIR172 | CGI11 | TC275039 | 620 | 825 | - | -64.10 |  |  | UGAAUCUUGAUGAUGCUGCAU | 5-5 |
| 184 | MIR172 | CGI11 | TC274900 | 602 | 734 | - | -58.41 | Y | ghr-MIR172 | AGAAUCUUGAUGAUGCUGCAG | 5-4 |
| 185 | MIR2118 | CGI11 | TC257237 | 1153 | 1320 | - | -48.20 |  | gra-MIR482 | UUGCCGAUUCCACCCAUGCCUA | 1-1 |
| 186 | MIR2947 | CGI11 | TC260378 | 35 | 141 | + | -51.22 |  | gar-MIR2947 | UAUACCGUGCCCAUGACUGUAG | 1-1 |
| 187 | MIR482 | CGI11 | DW517596 | 155 | 268 | + | -54.50 | Y | ghr-MIR2948 | UGUGGGAGAGUUGGGCAAGAAU | 7-6 |
| 188 | MIR2949 | CGI11 | TC244560 | 403 | 501 | - | -31.20 | Y | ghr-MIR2949c_b_a | ACUUUUGAACUGGAUUUGCCGA | 2-1 |
| 189 | MIR2949 | CGI11 | EV497941 | 273 | 391 | + | -41.00 | Y | ghr-MIR2949c_b_a | __UUUUGAACUGGAUUUGCCGA(CU) | 2-2 |
| 190 | MIR2949 | CGI11 | AI054573 | 103 | 223 | + | -46.80 | Y | ghr-MIR2949a_c_b | ACUUUUGAACUGGAUUUGCCGA | 2-1 |
| 191 | MIR2950 | CGI11 | DW514754 | 87 | 201 | + | -51.10 | Y | ghr-MIR2950 | UGGUGUGCAGGGGGUGGAAUA | 1-1 |
| 192 | MIR3476 | CGI11 | TC257664 | 1579 | 1693 | - | -43.20 |  | ghr-MIR3476 | UGAACUGGGUUUGUUGGCUGC | 1-1 |
| 193 | MIR3476 | CGI11 | CO119151 | 193 | 307 | + | -43.20 | Y | ghr-MIR3476 | UGAACUGGGUUUGUUGGCUGC | 1-1 |
| 194 | MIR390 | CGI11 | TC237382 | 56 | 192 | + | -55.00 | Y | ghr-MIR390b_a_c | AAGCUCAGGAGGGAUAGCGCC | 1-1 |
| 195 | MIR390 | CGI11 | DW238152 | 51 | 187 | + | -55.00 | Y | ghr-MIR390b_a_c | AAGCUCAGGAGGGAUAGCGCC | 1-1 |
| 196 | MIR393 | CGI11 | TC269580 | 64 | 224 | + | -62.30 | Y | ghr-MIR393 | UCCAAAGGGAUCGCAUUGAUCU | 2-2 |
| 197 | MIR394 | CGI11 | ES802173 | 447 | 551 | + | -45.00 |  | ghr-MIR394a | UUGGCAUUCUGUCCACCUCC | 2-2 |
| 198 | MIR394 | CGI11 | DW517361 | 103 | 220 | + | -38.72 |  | ghr-MIR394b | UUGGCAUUCUGUCCACCUCC | 2-2 |
| 199 | MIR396 | CGI11 | TC275676 | 175 | 331 | + | -59.10 | Y | ghr-MIR396b_a | UUCCACAGCUUUCUUGAACUG | 3-1 |
| 200 | MIR171_2 | CGI11 | ES809290 | 22 | 128 | + | -43.70 | Y | ghr-MIR479 | CGUGAUAUUGGUUCGGCUCAUC | 2-2 |
| 201 | MIR482 | CGI11 | TC257237 | 1093 | 1194 | - | -47.59 |  |  | UUUCCAAUUCCUCCCAUUCCAC | 7-4 |
| 202 | MIR482 | CGI11 | DR457519 | 174 | 279 | + | -40.90 |  | ghr-MIR482a | (UC)UUUCCUACUCCUCCCAUACC__ | 7-7 |
| 203 | MIR482 | CGI11 | DT527030 | 40 | 170 | + | -55.10 |  | ghr-MIR482b | UUGCCUACUCCACCCAUGCCAC | 7-1 |
| 204 | MIR827_2 | CGI11 | TC257800 | 68 | 210 | + | -43.10 |  | ghr-MIR827b_a_c | UUAGAUGACCAUCAACAAACA | 1-1 |
| 205 | MIR827_2 | CGI11 | TC250696 | 442 | 584 | - | -43.10 |  | ghr-MIR827a_c_b | UUAGAUGACCAUCAACAAACA | 1-1 |
| 206 | MIR827_2 | CGI11 | TC235276 | 75 | 217 | + | -41.70 |  | ghr-MIR827c_a_b | UUAGAUGACCAUCAACAAACA | 1-1 |
| 207 | MIR7505 | CGI11 | CO070343 | 242 | 348 | - | -53.20 | Y |  | UUCAGAAACCAUCCCUUCCUU | 1-1 |
| 208 | MIR156 | CGI11 | gi\|88714468\|gb\|DX383155.1\|DX383155 | 185 | 308 | - | -53.53 | Y | ghr-MIR156a_b_c | UGACAGAAGAGAGUGAGCAC | 6-1 |
| 209 | MIR169_1 | GSS | gi\|89126590\|gb\|DX401397.1\|DX401397 | 285 | 416 | + | -58.60 | Y | ghr-MIR169 | UAGCCAAGGAUGACUUGCCUG | 6-1 |
| 210 | MIR169_1 | GSS | gi\|89126590\|gb\|DX401397.1\|DX401397 | 356 | 591 | + | -88.30 | Y |  | UAGCCAAGAAUGACUUGCCU_ | 6-4 |
| 211 | MIR169_1 | GSS | gi\|254957047\|gb\|GS015924.1\|GS015924 | 451 | 576 | - | -45.70 |  |  | GGCAGUCACCUUGGCUAAUC^e^ | 6-5 |
| 212 | MIR169_1 | GSS | gi\|254954903\|gb\|GS017517.1\|GS017517 | 458 | 594 | - | -52.00 |  |  | GGCAGUCACCUUGGCUAAUC^e^ | 6-5 |
| 213 | MIR477 | GSS | gi\|254961508\|gb\|GS022450.1\|GS022450 | 67 | 180 | - | -58.90 |  |  | ACUCUCCCUCAAGGGCUUCCCC | 6-1 |

a: Y indicates the precise miRNA* sequence (two-nucleotide 3’ overhangs).

b: Annotation of 213 precursors based on the miRs in miRBase (19.0); gar: *Gossypium arboretum*; ghb: *Gossypium herbaceum*; ghr: *Gossypium hirsutum*; gra: *Gossypium raimondii*; tcc: *Theobroma cacao*; ctr: *Citrus trifoliate*; hbr*:* *Hevea brasiliensis;* vvi: *Vitis vinifera*; ptc: *Populus trichocarpa*; *mdm*: *Malus domestica*.

c: Various miRNA sequences generated from the corresponding precursors. Compared to the representative sequence in the “Sequence” column of Additional file 1, “_” and nucleotides in “()” indicate there are fewer or more nucleotides; red highlighting indicates mutant.

d: The number preceding “-” represents the total number of distinct miRNA members in the same miRNA family, and the number following “-” represents the position of the member in the miRNA family.

e: miRNA* sequence of representative sequence in Additional file 1.

f-g: The sequences of miR159 and miR319 are similar and both belong to the miR159 family. f: the annotation of MIR159 (4-2) is from ath-miR319a; g: the annotation of MIR159 (4-1) is from ath-miR159a. ath: *Arabidopsis thaliana*.

h-i: h: The annotation of MIR159 (4-4) is from the gma-miR159d; i: the annotation of MIR399 (6-4) is from the cme-miR399g. Both of these miRNA sequences are significantly different from the corresponding representative miRNA sequences in the “Sequence” column of Additional file 1. gma: *Glycine max*; cme: *Cucumis melo*.
