# Supplementary material for: Genome-wide analysis of small RNAs reveals eight fiber elongation-related and 257 novel microRNAs in elongating cotton fiber cells
Source: BMC Genomics. 2013 Sep 17;14:629. doi: 10.1186/1471-2164-14-629 (PMC3849097; doi:10.1186/1471-2164-14-629)
Supplement: Additional file 3: Figure S1 — The 139 novel stem-loop structures of known miRNA precursors in elongating cotton fibers. In most cases, the red highlighting indicates the miRNA sequence and the blue highlighting indicates the miRNA* sequence. For example, in ‘17-MIR159-[hbr-MIR159a MI0022053]’, the ‘17’ came from the “index” column in the Additional file 2; “MIR159” came from the “name” column in the Additional file 2; and [hbr-MIR159a MI0022053] was the homolog of the precursor in miRBase. tcc: Theobroma cacao; ctr: Citrus trifoliate; hbr: Hevea brasiliensis; vvi: Vitis vinifera; ptc: Populus trichocarpa; mdm: Malus domestica. [file 1471-2164-14-629-S3.pdf]

Additional Figure S1:

The 139 novel stem-loop structures of known miRNA precursors in elongating cotton fibers. In most cases, the red highlighting indicates the miRNA sequence and the blue highlighting indicates the miRNA* sequence. For example, in '17-MIR159-[hbr-MIR159a MI0022053]', the '17' came from the "index" column in the additional file 2; "MIR159" came from the "name" column in the additional file 2; and [hbr-MIR159a MI0022053] was the homolog of the precursor in miRBase. *tcc*: *Theobroma cacao*; *ctr*: *Citrus trifoliate*; *hbr*: *Hevea brasiliensis*; *vvi*: *Vitis vinifera*; *ptc*: *Populus trichocarpa*; *mdm*: *Malus domestica*.

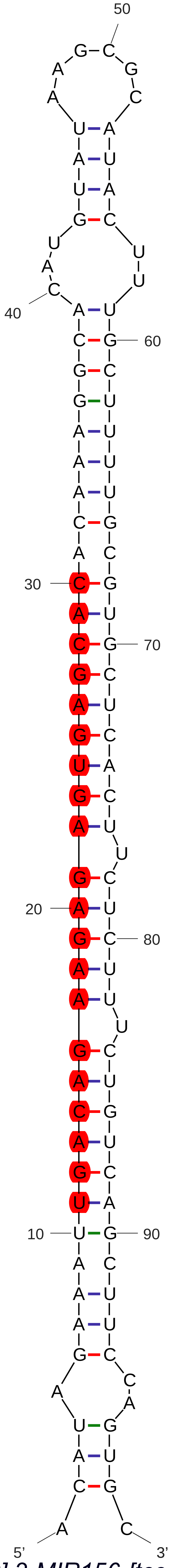

dG = -47.60 [Initially -47.60] 3-MIR156-[tcc-MIR156g MI0017459]

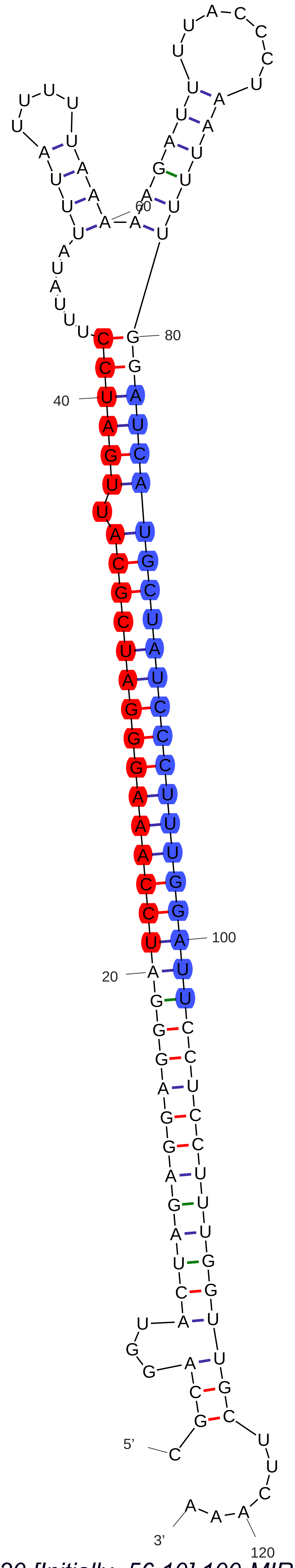

dG = -54.20 [Initially -56.10] 100-MIR393

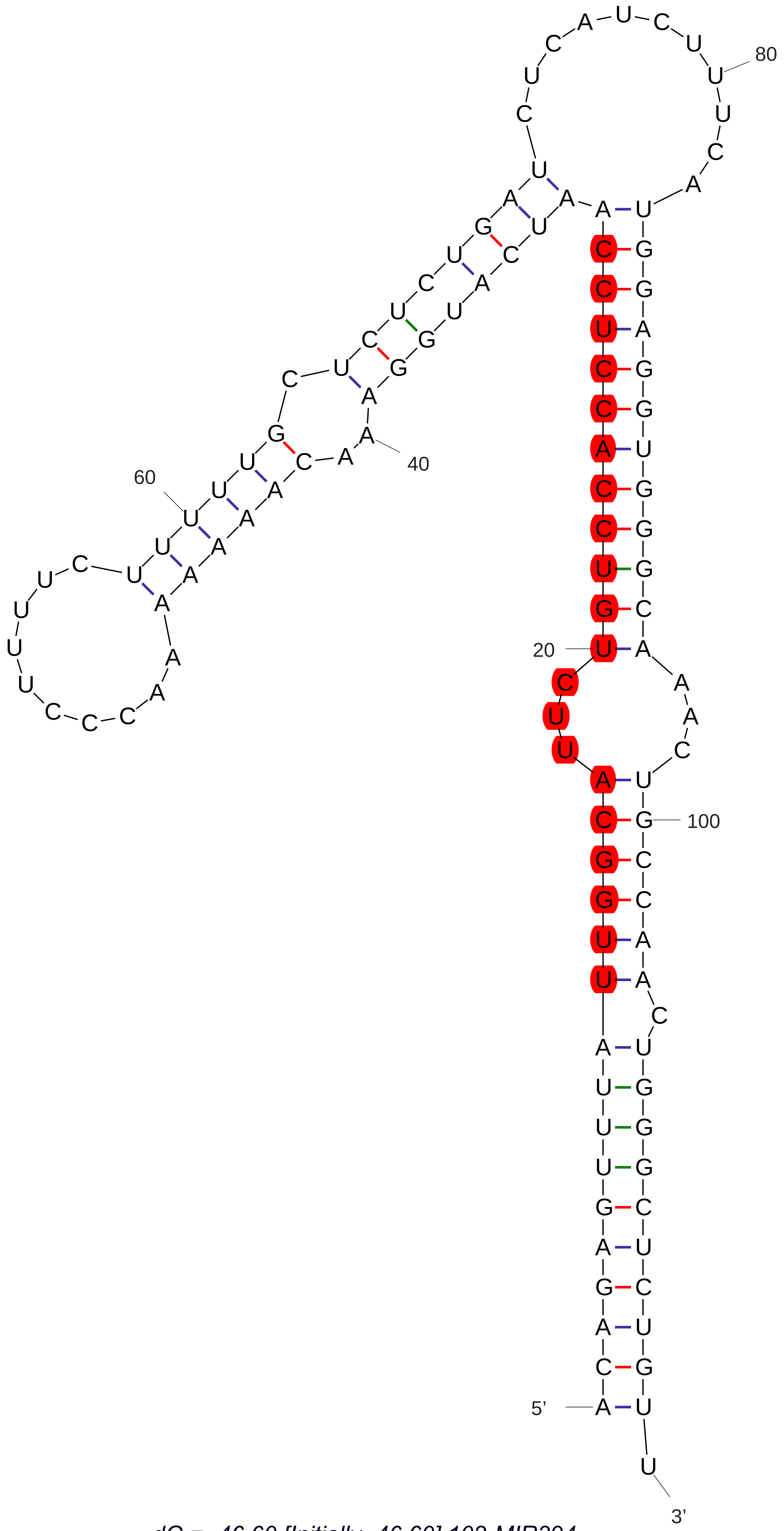

dG = -46.60 [Initially -46.60] 102-MIR394

Output of sir_graph (©)
mfold_util 4.6

Created Thu Jan 24 21:11:39 2013

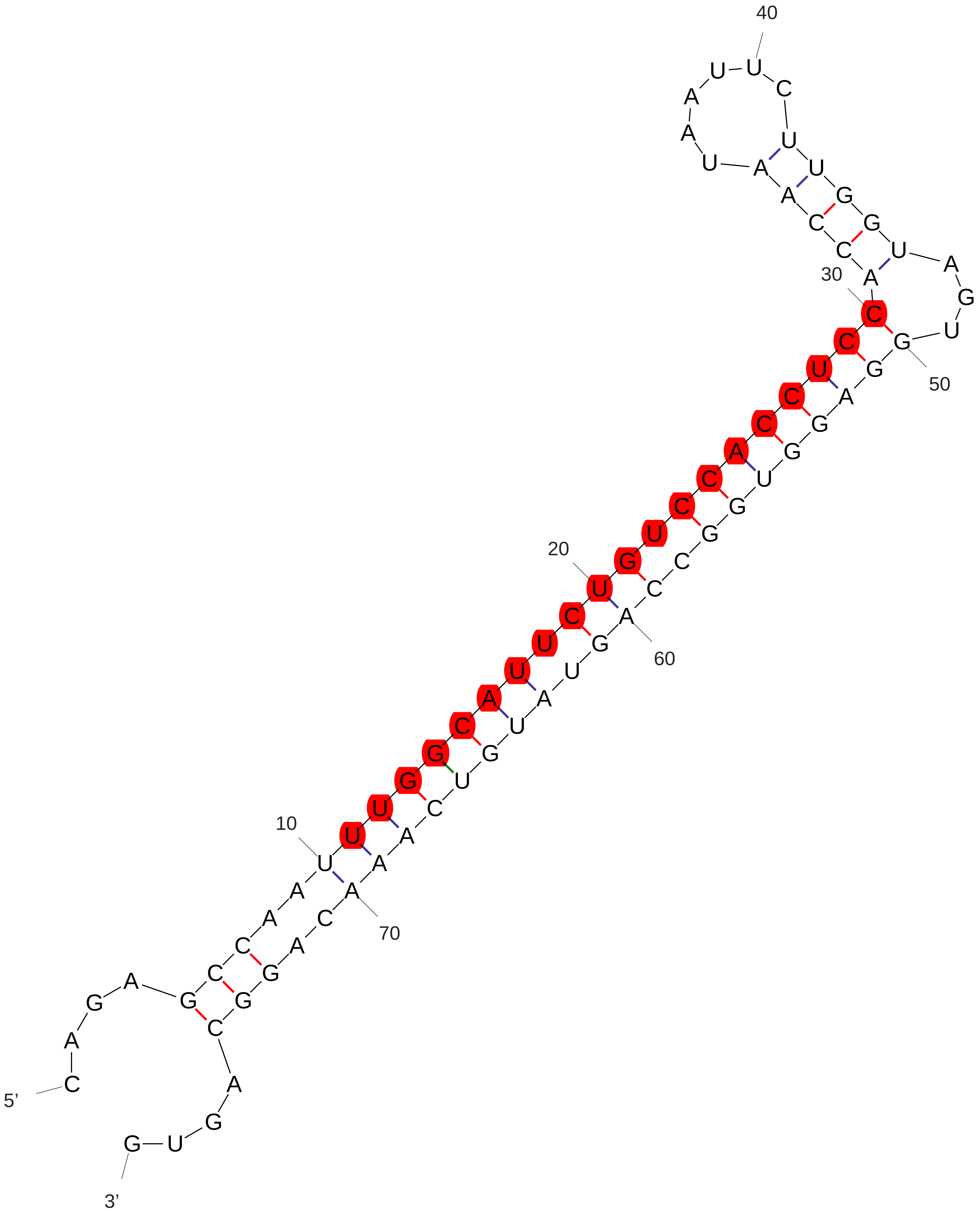

dG = -39.00 [Initially -39.00] 103-MIR394

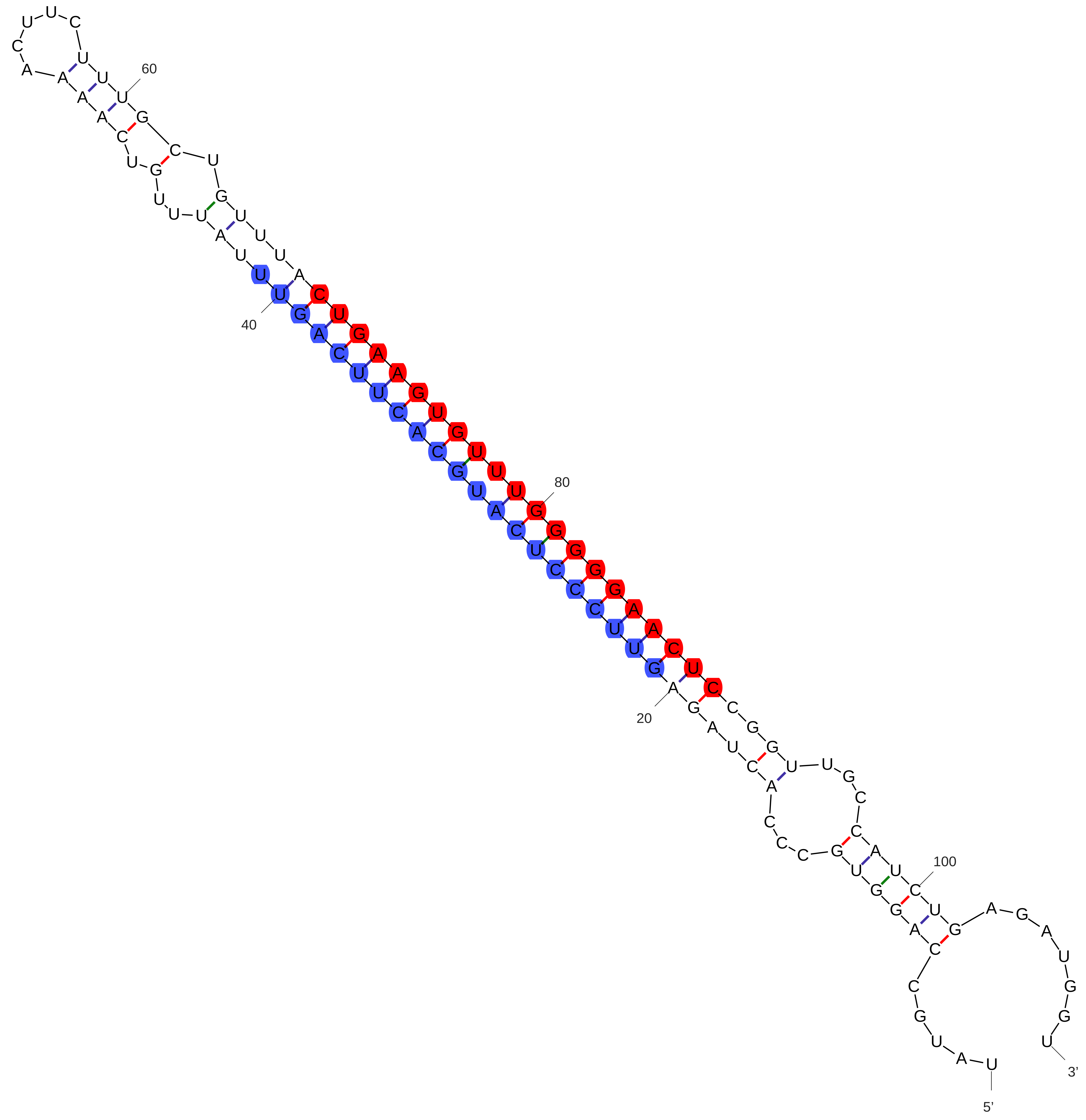

dG = -47.20 [Initially -47.50] 106-MIR395

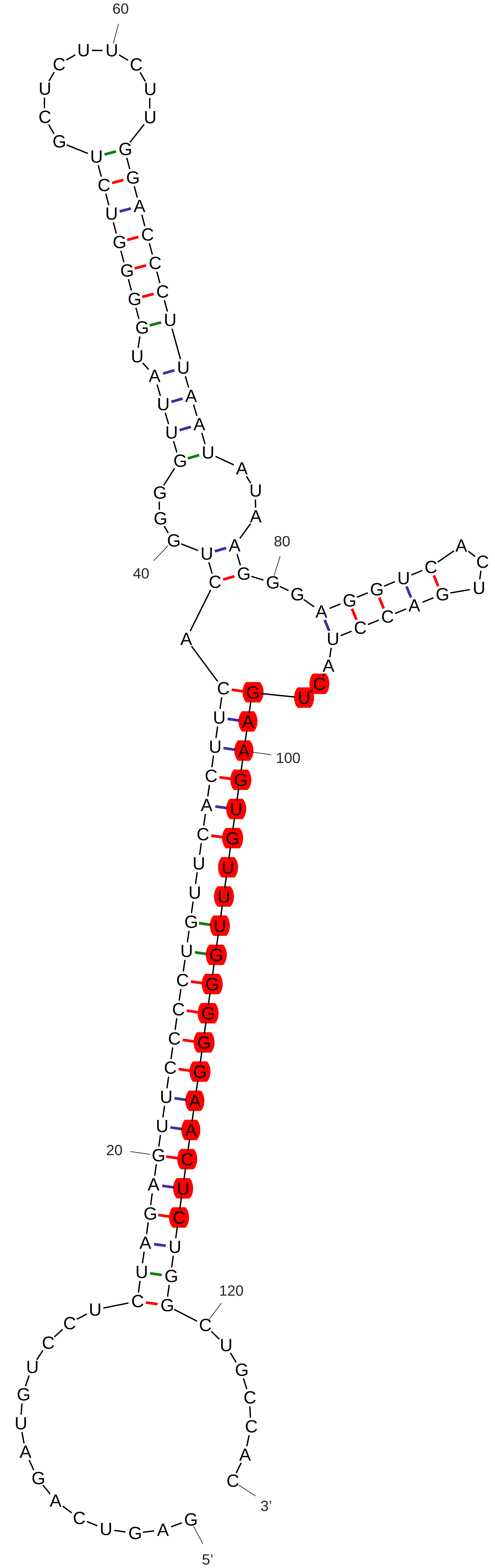

dG = -48.90 [Initially -51.40] 107-MIR395

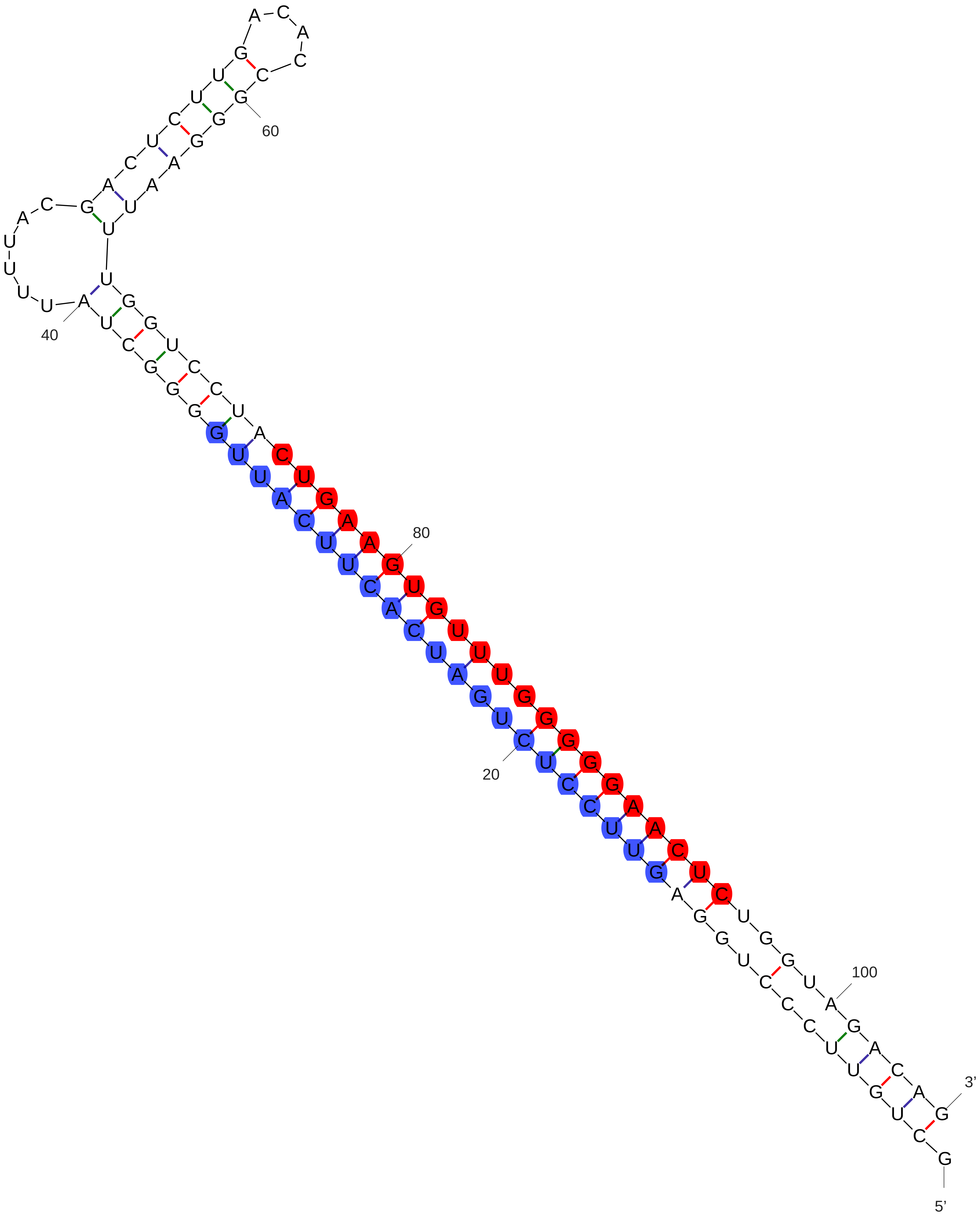

dG = -51.20 [Initially -51.20] 108-MIR395

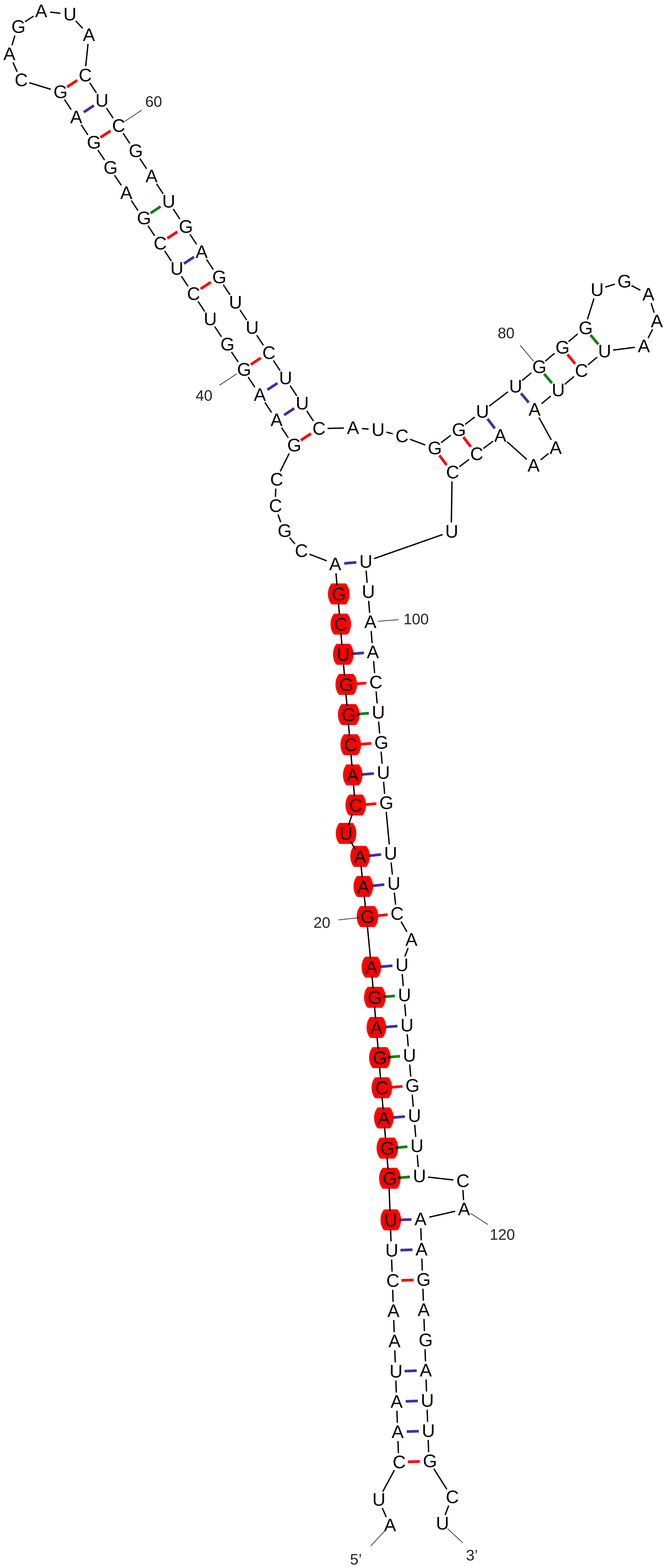

dG = -32.18 [Initially -34.10] 109-MIR3954

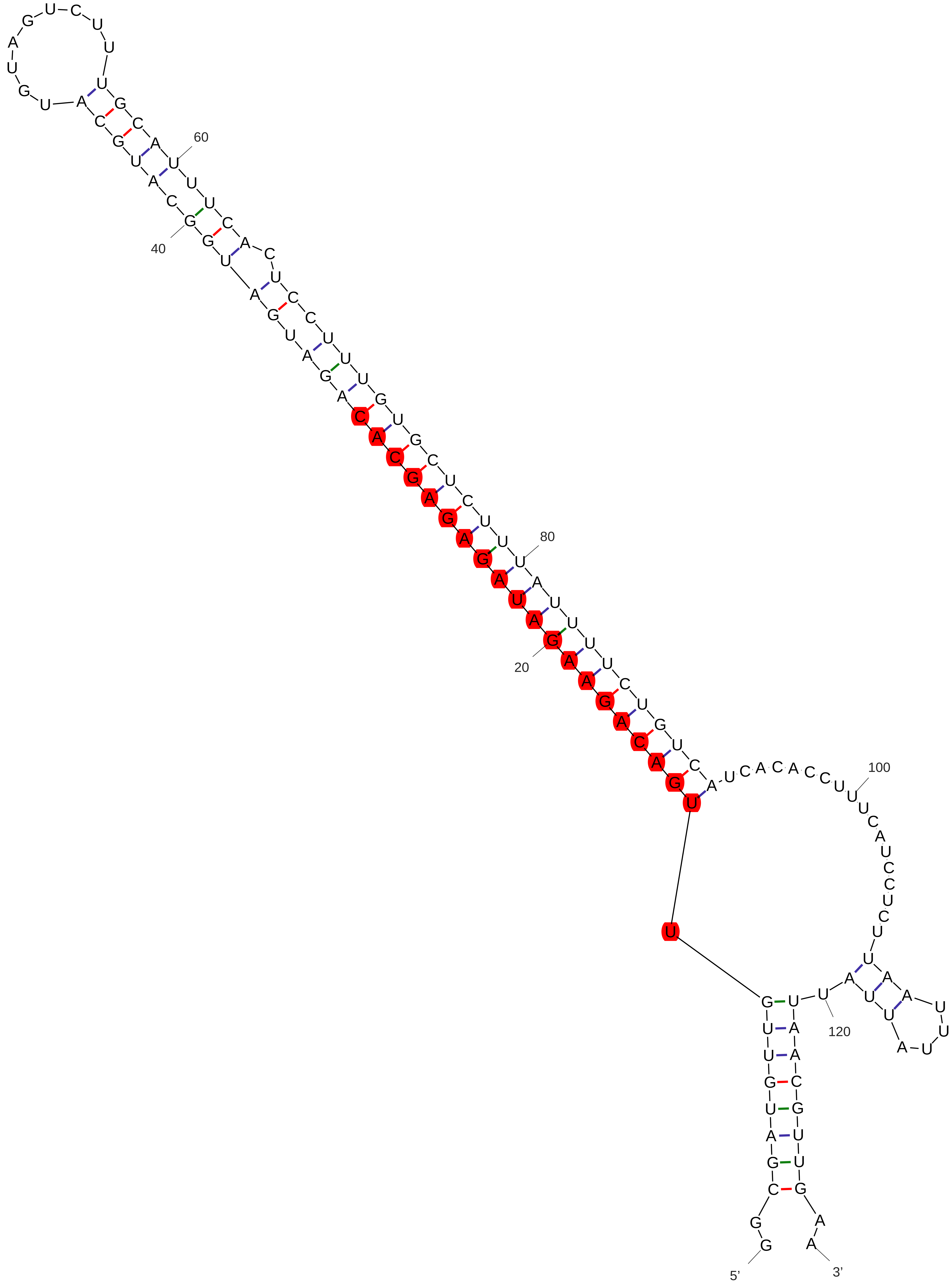

dG = -43.08 [Initially -46.30] 10-MIR156-[tcc-MIR156e MI0017457]

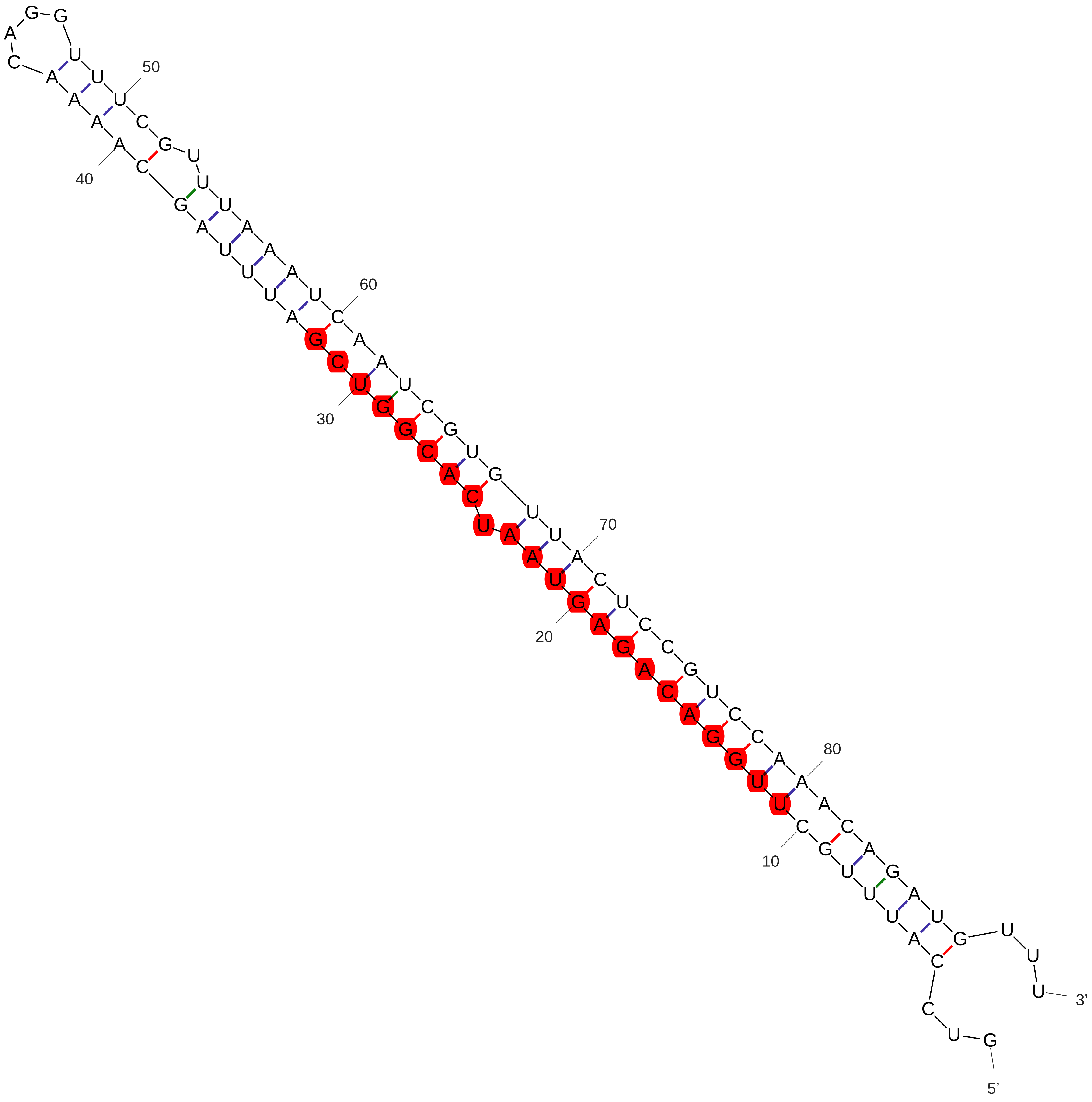

dG = -35.80 [Initially -35.80] 110-MIR3954

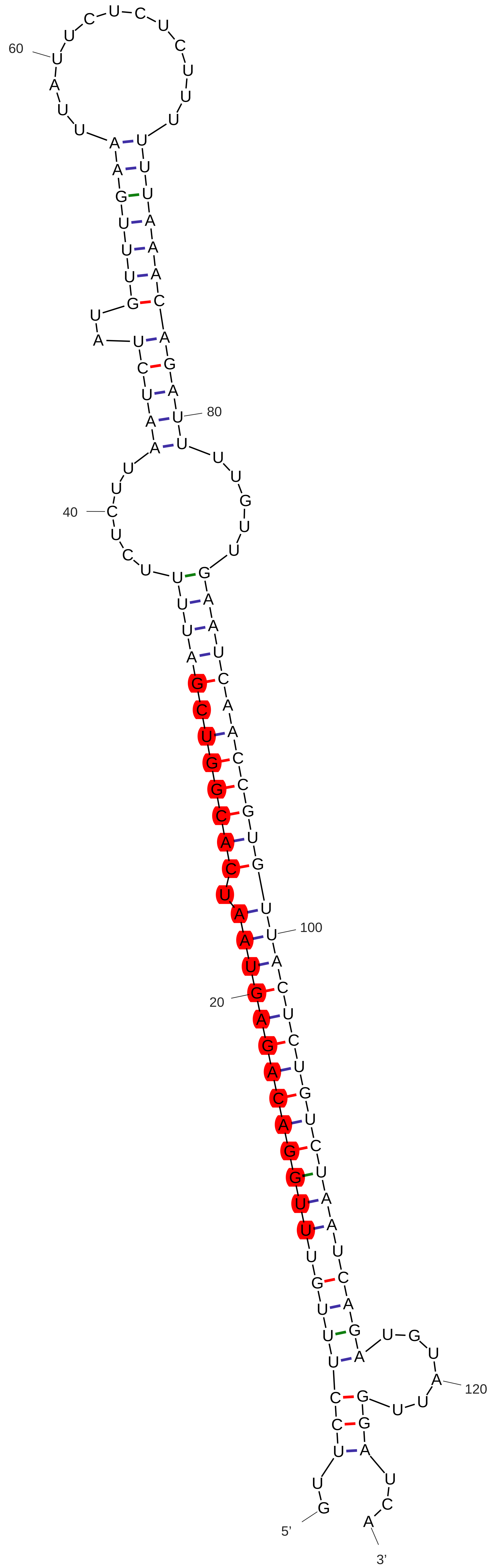

dG = -42.60 [Initially -42.60] 111-MIR3954

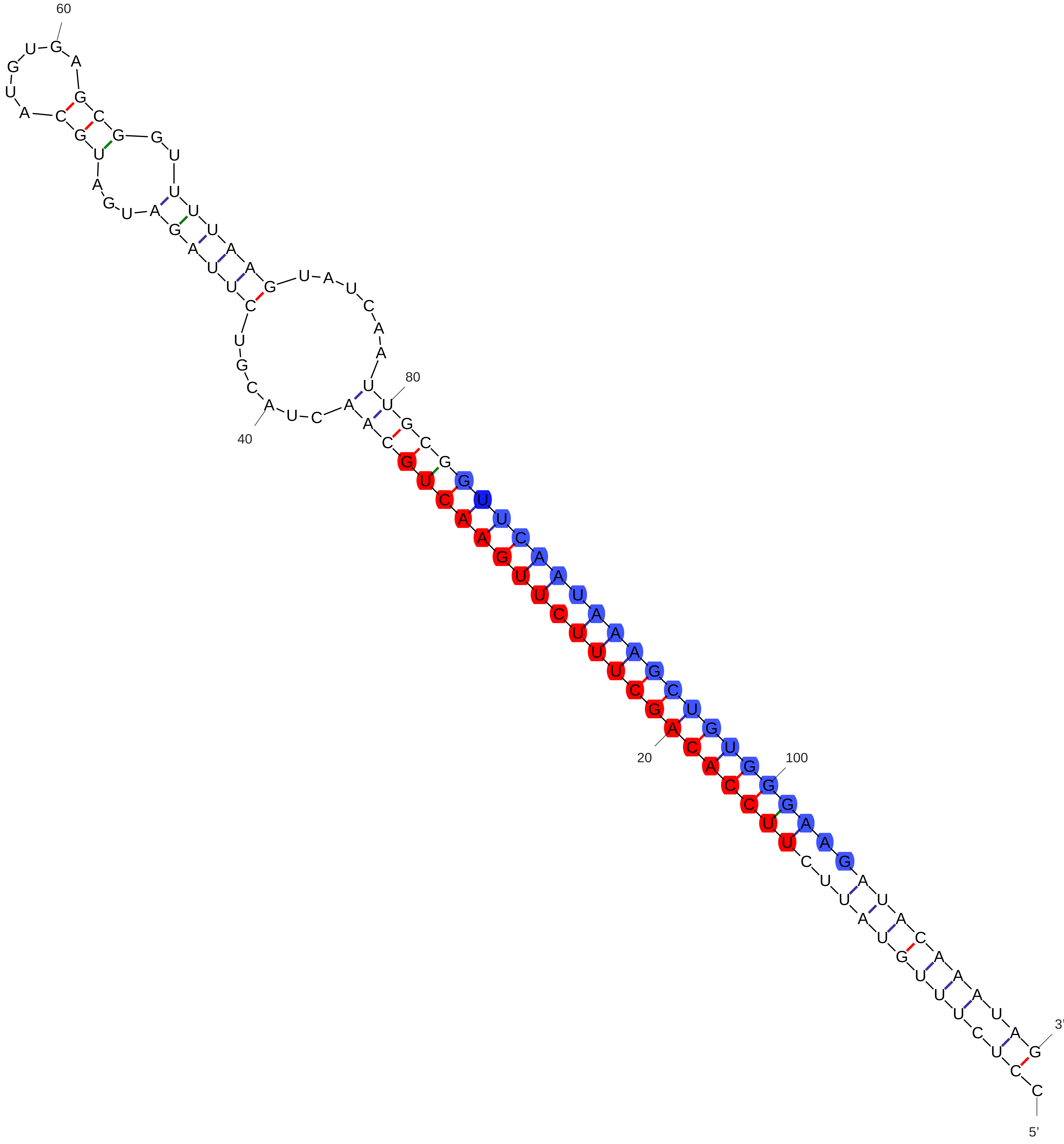

dG = -50.50 [Initially -50.50] 112-MIR396

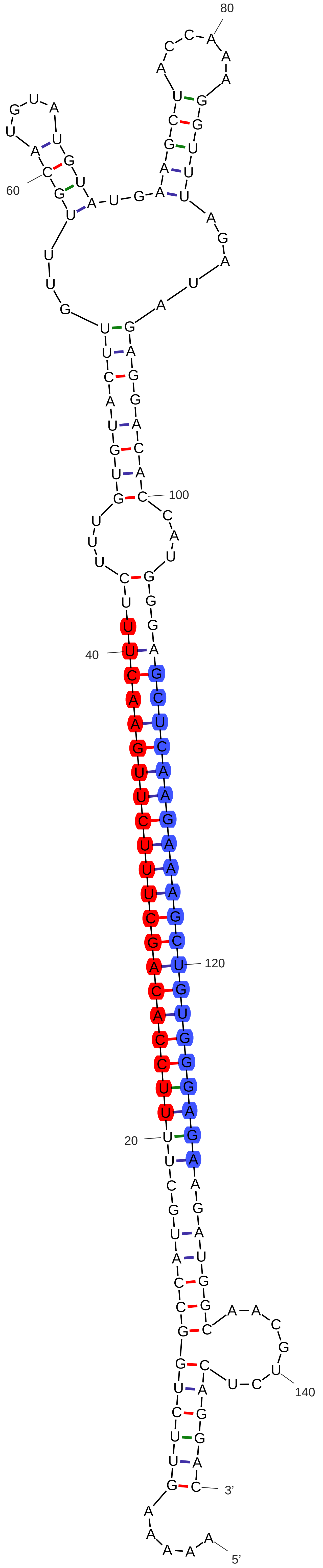

dG = -53.34 [Initially -56.70] 113-MIR396

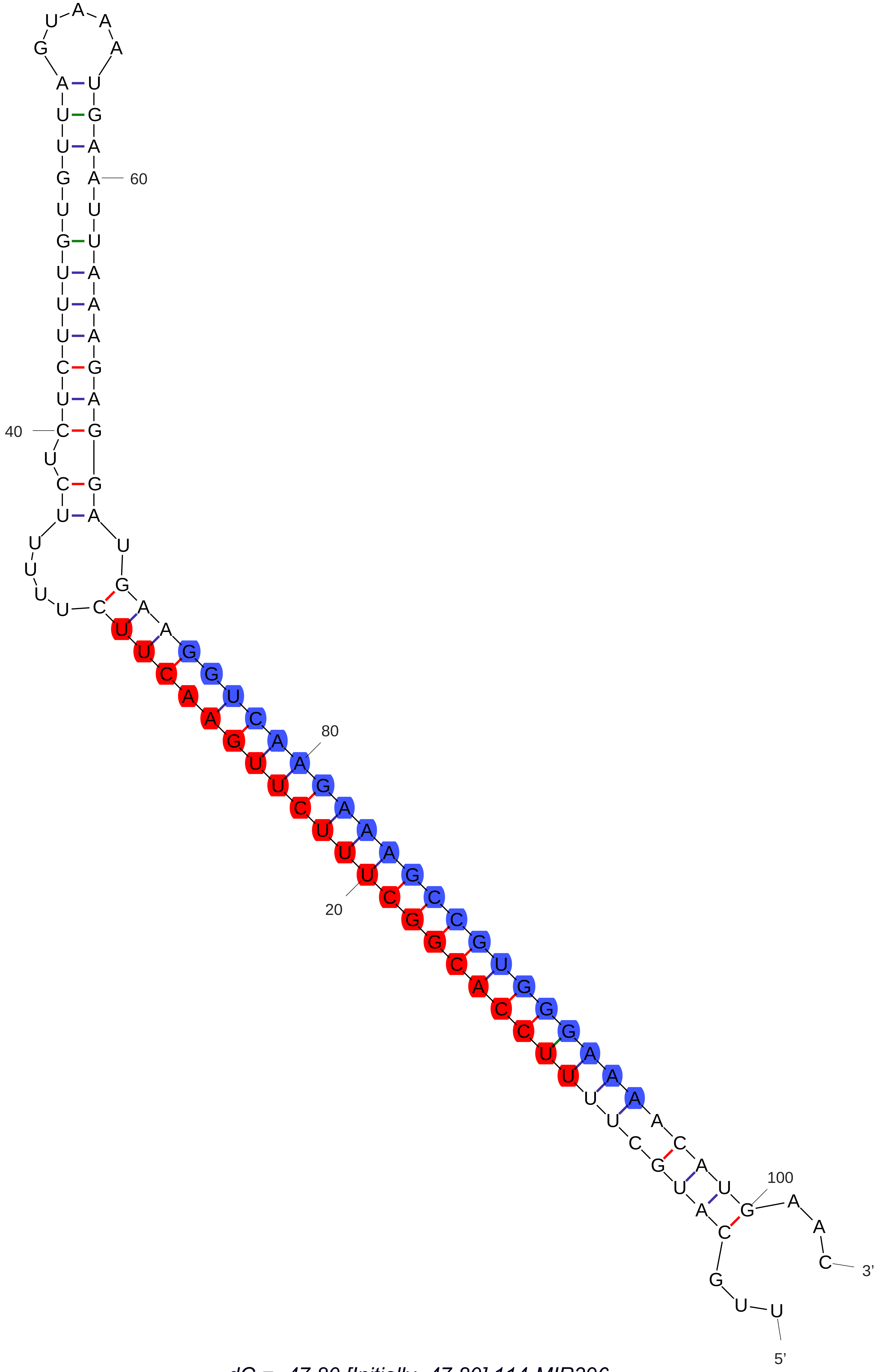

dG = -47.80 [Initially -47.80] 114-MIR396

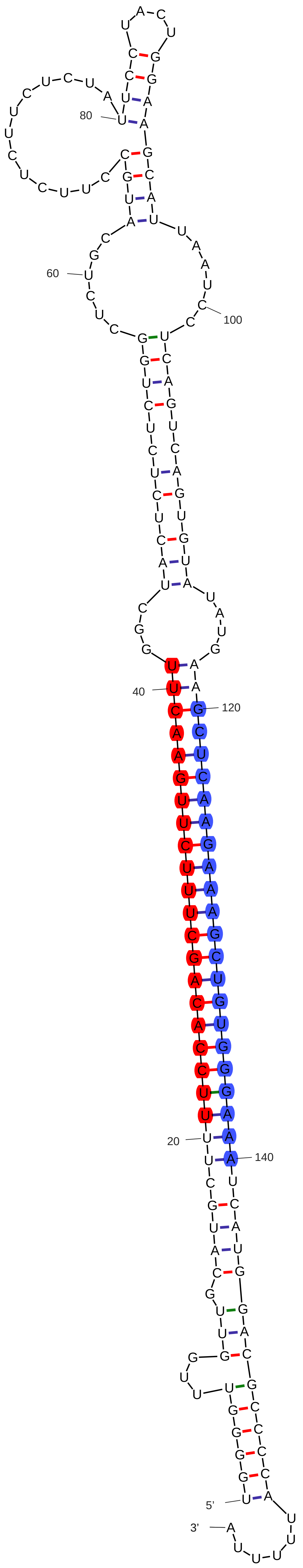

dG = -58.00 [Initially -58.00] 115-MIR396

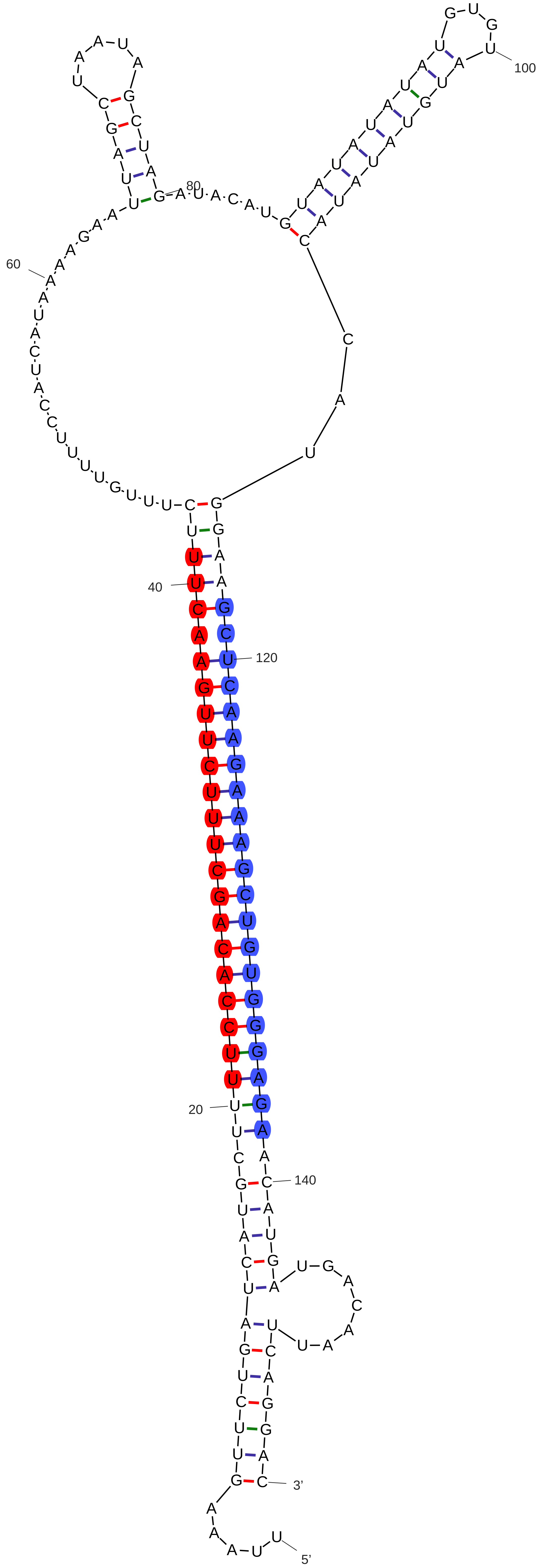

dG = -53.99 [Initially -59.10] 117-MIR396

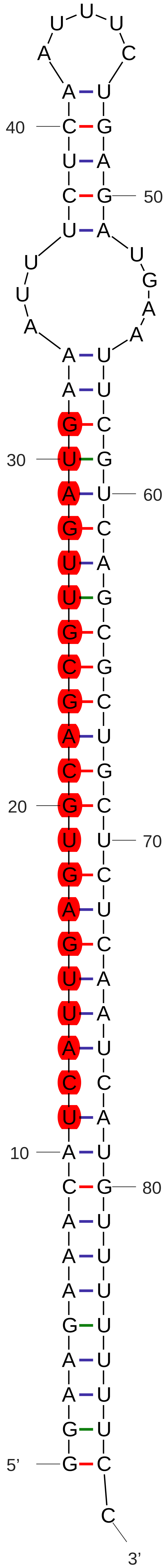

dG = -48.40 [Initially -48.40] 118-MIR397

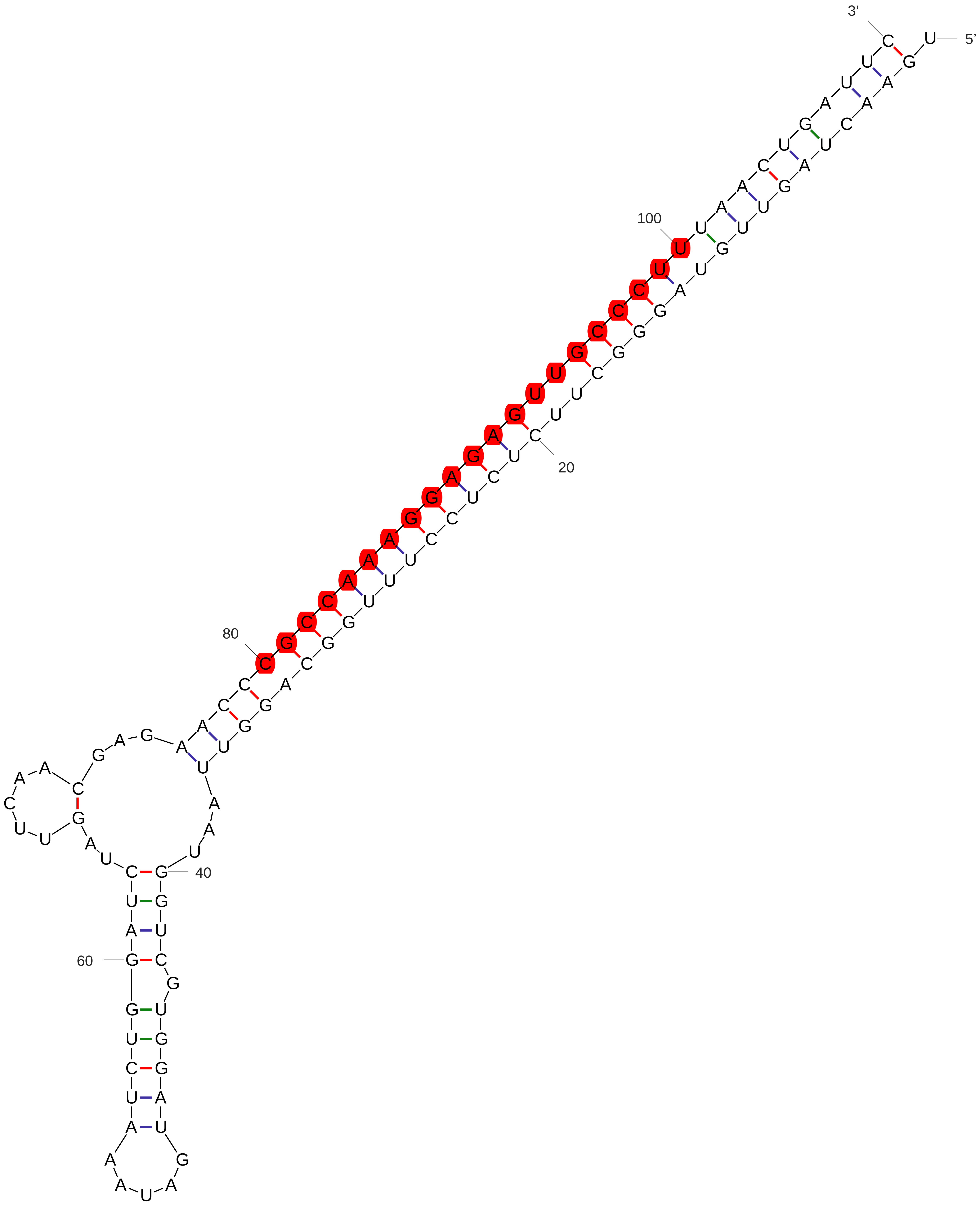

dG = -46.88 [Initially -49.70] 119-MIR399

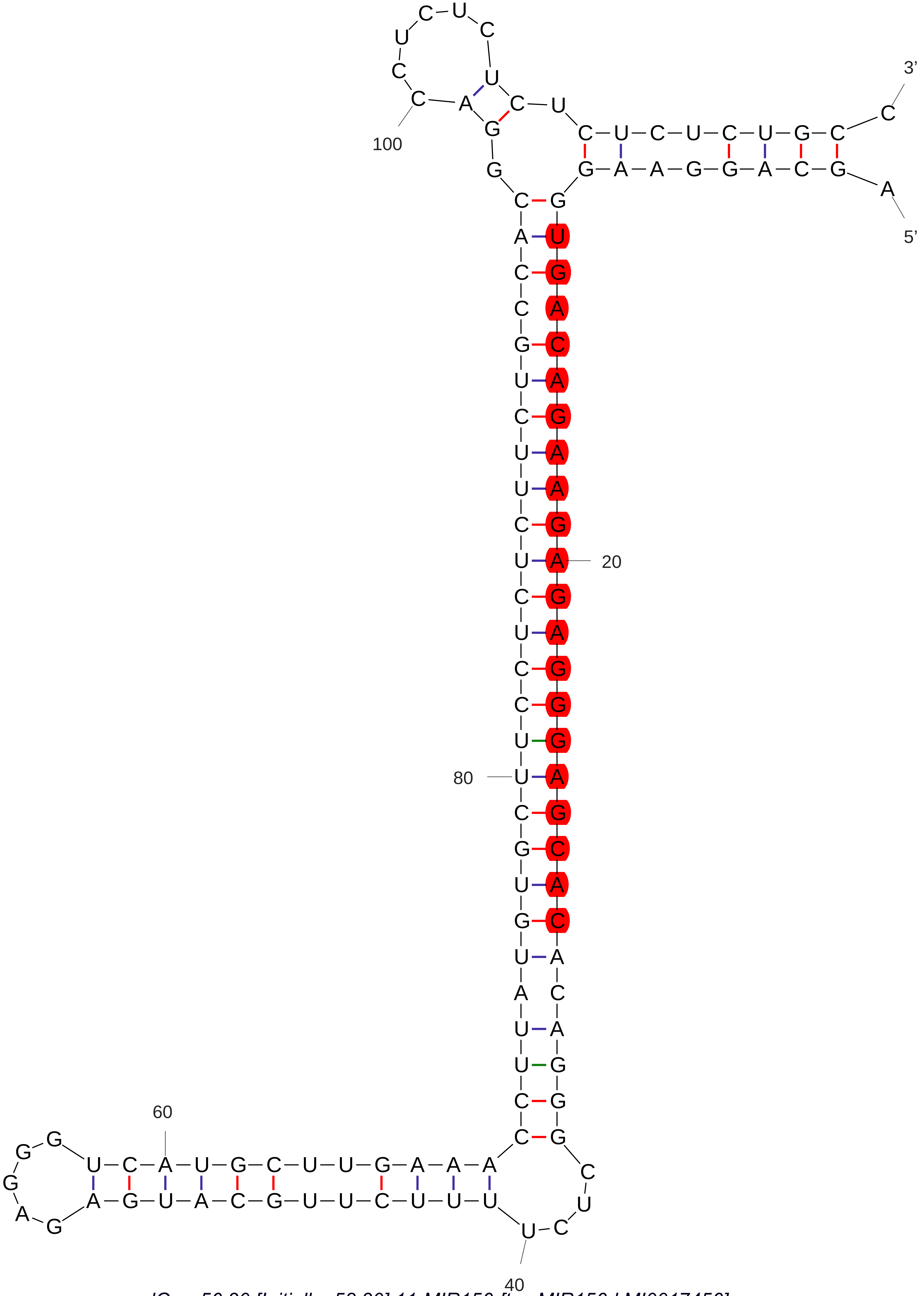

dG = -56.30 [Initially -58.30] 11-MIR156-[tcc-MIR156d MI0017456]

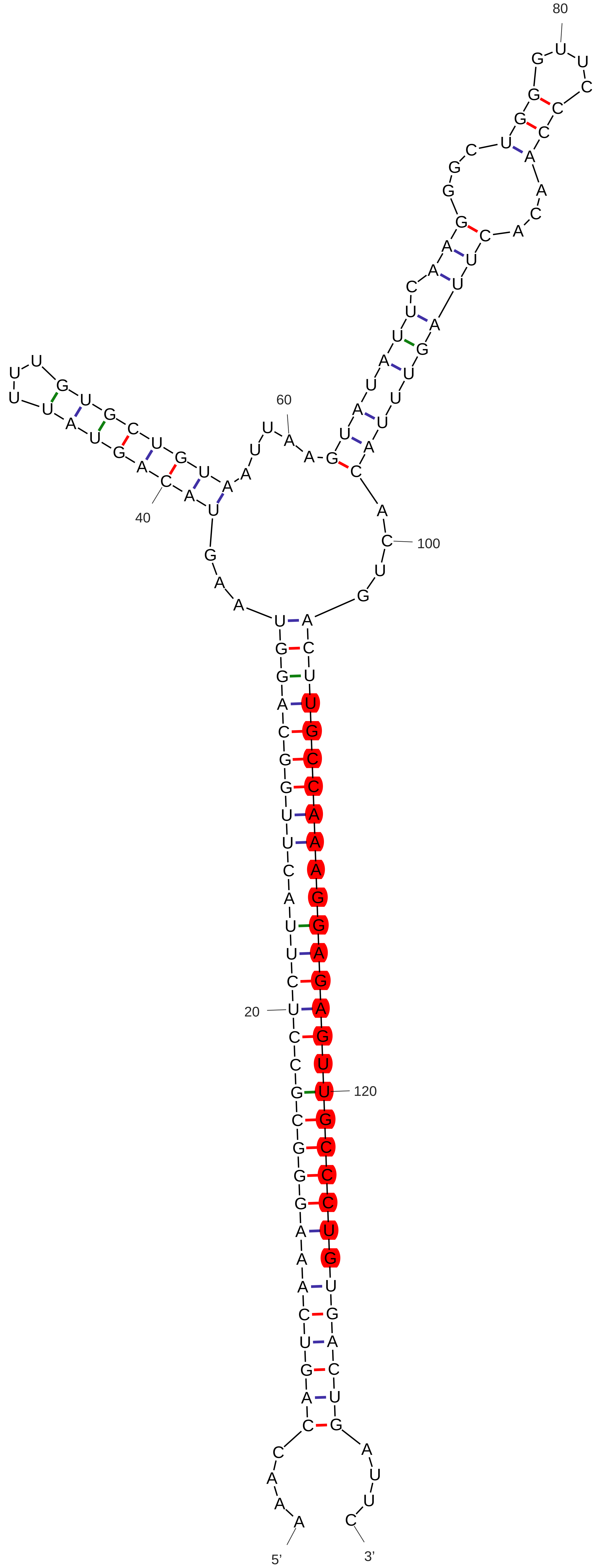

dG = -52.94 [Initially -56.40] 120-MIR399

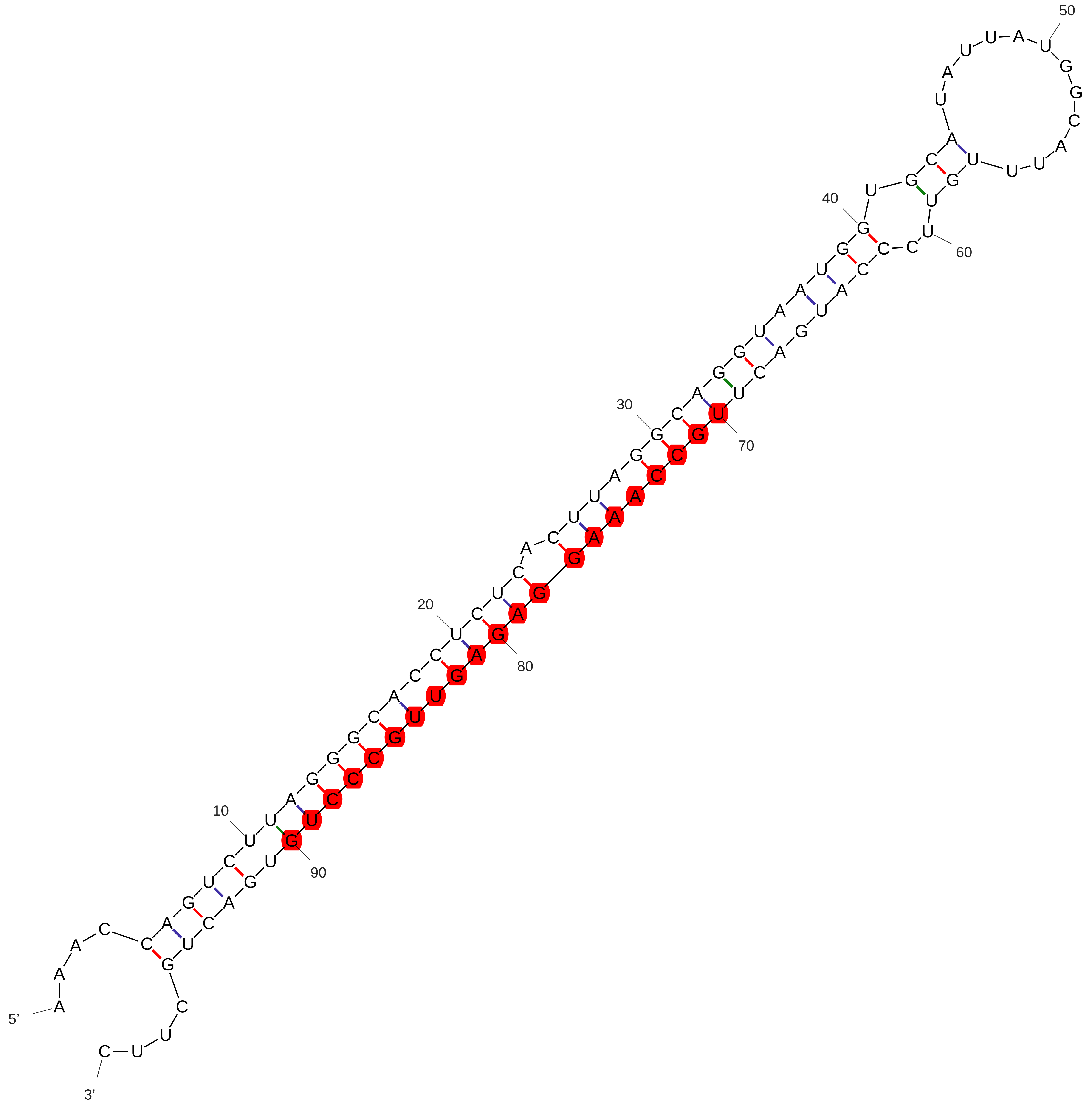

dG = -47.90 [Initially -47.90] 121-MIR399

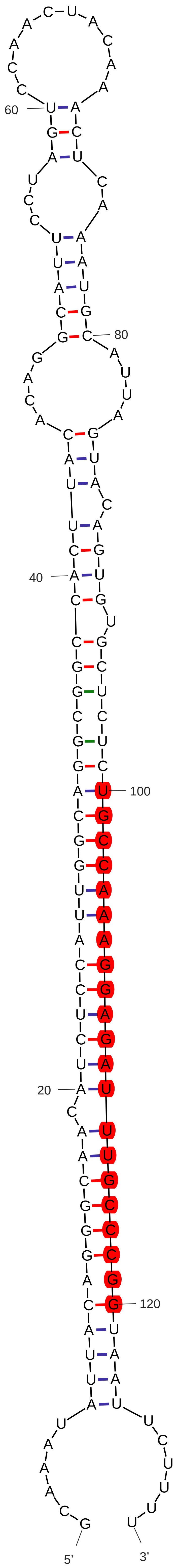

dG = -51.50 [Initially -51.50] 123-MIR399

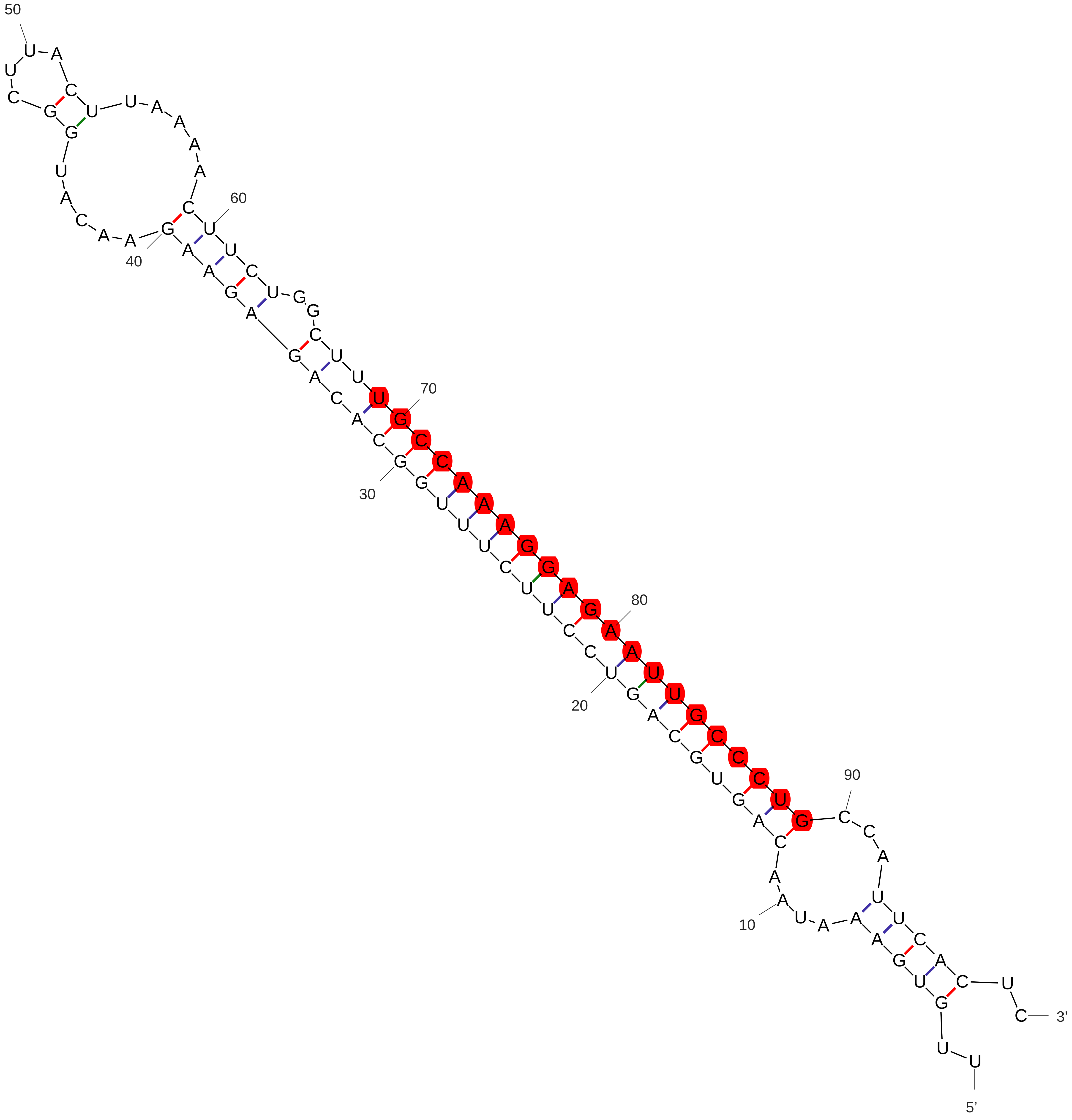

dG = -34.90 [Initially -34.90] 124-MIR399

Output of sir_graph (©)
mfold_util 4.6

Created Thu Jan 24 21:24:51 2013

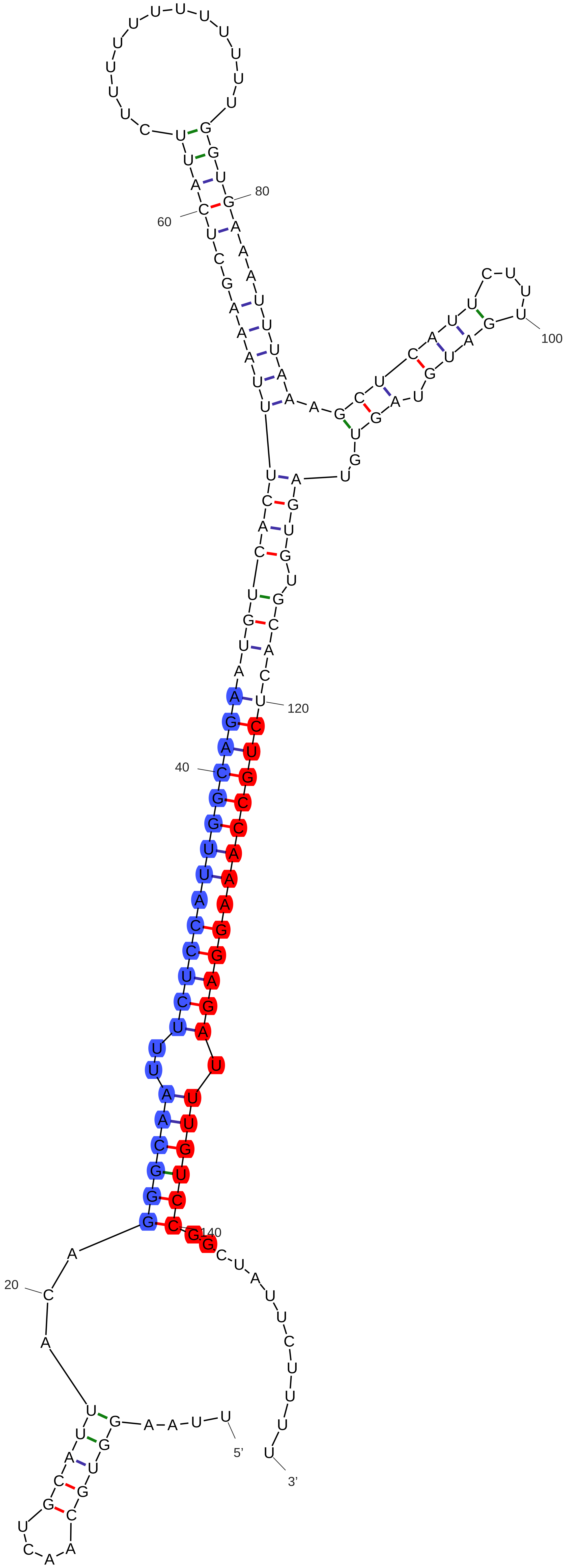

dG = -45.30 [Initially -45.90] 125-MIR399

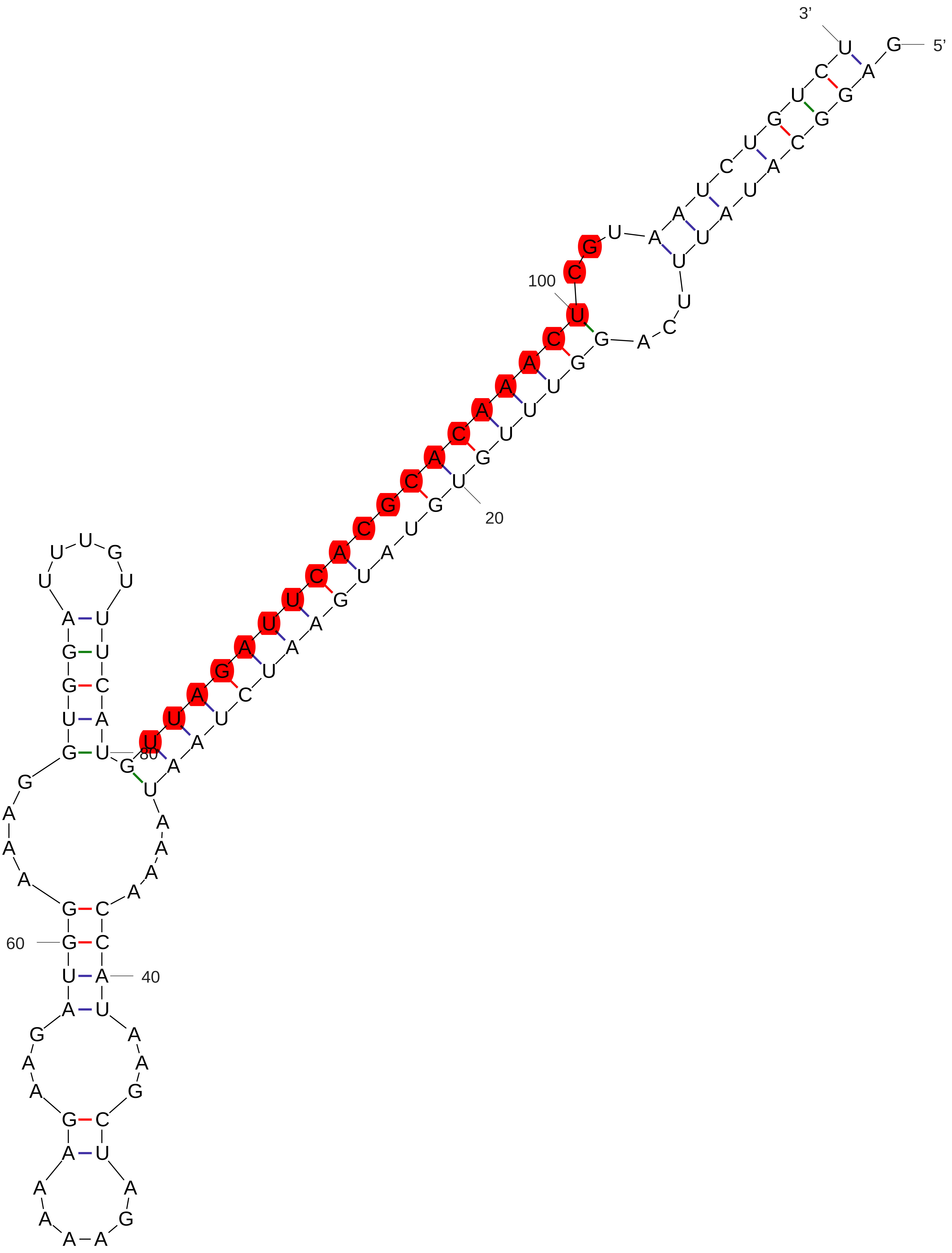

dG = -33.08 [Initially -36.20] 126-MIR403-[tcc-MIR403a MI0017528]

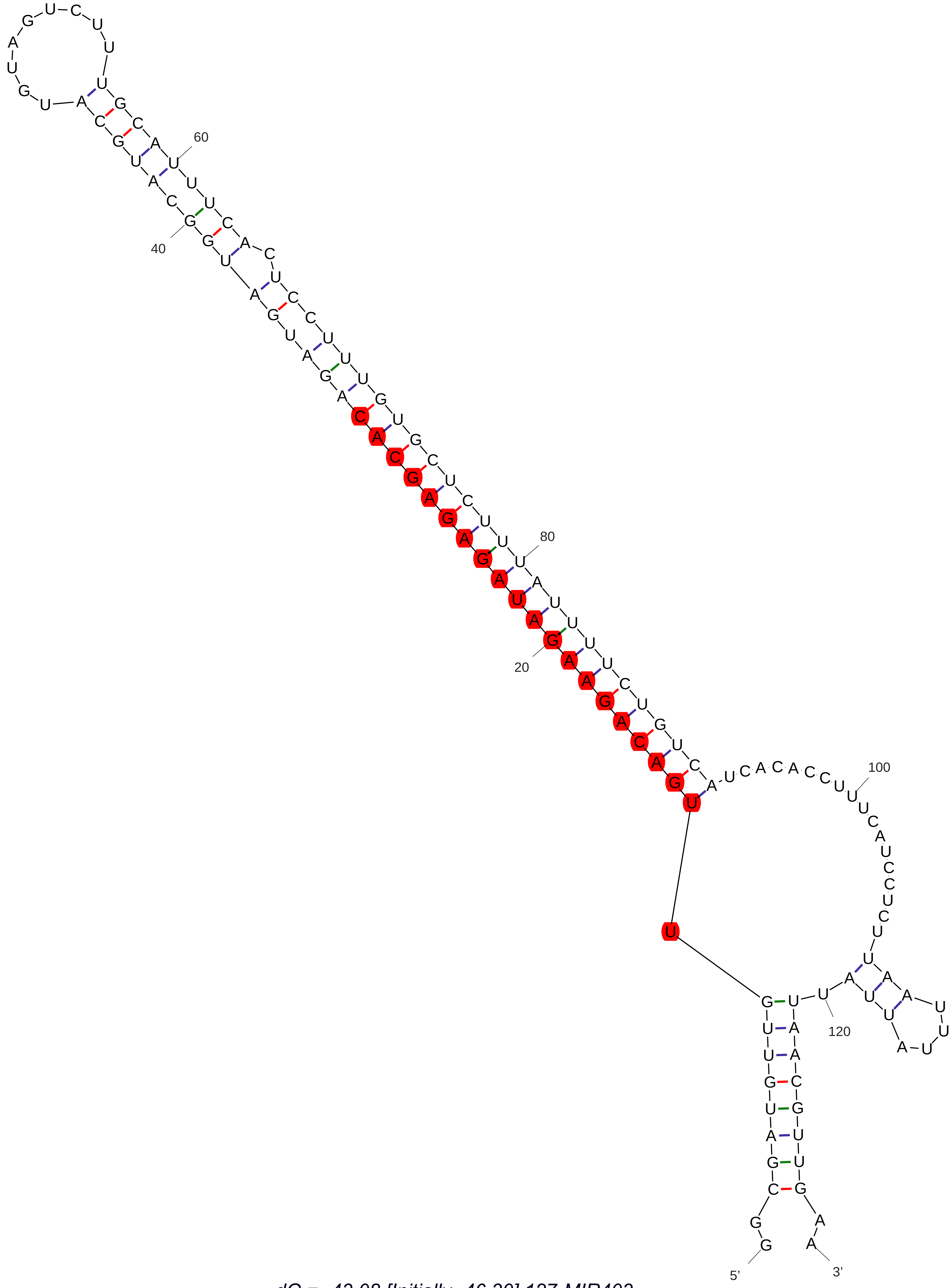

dG = -43.08 [Initially -46.30] 127-MIR403

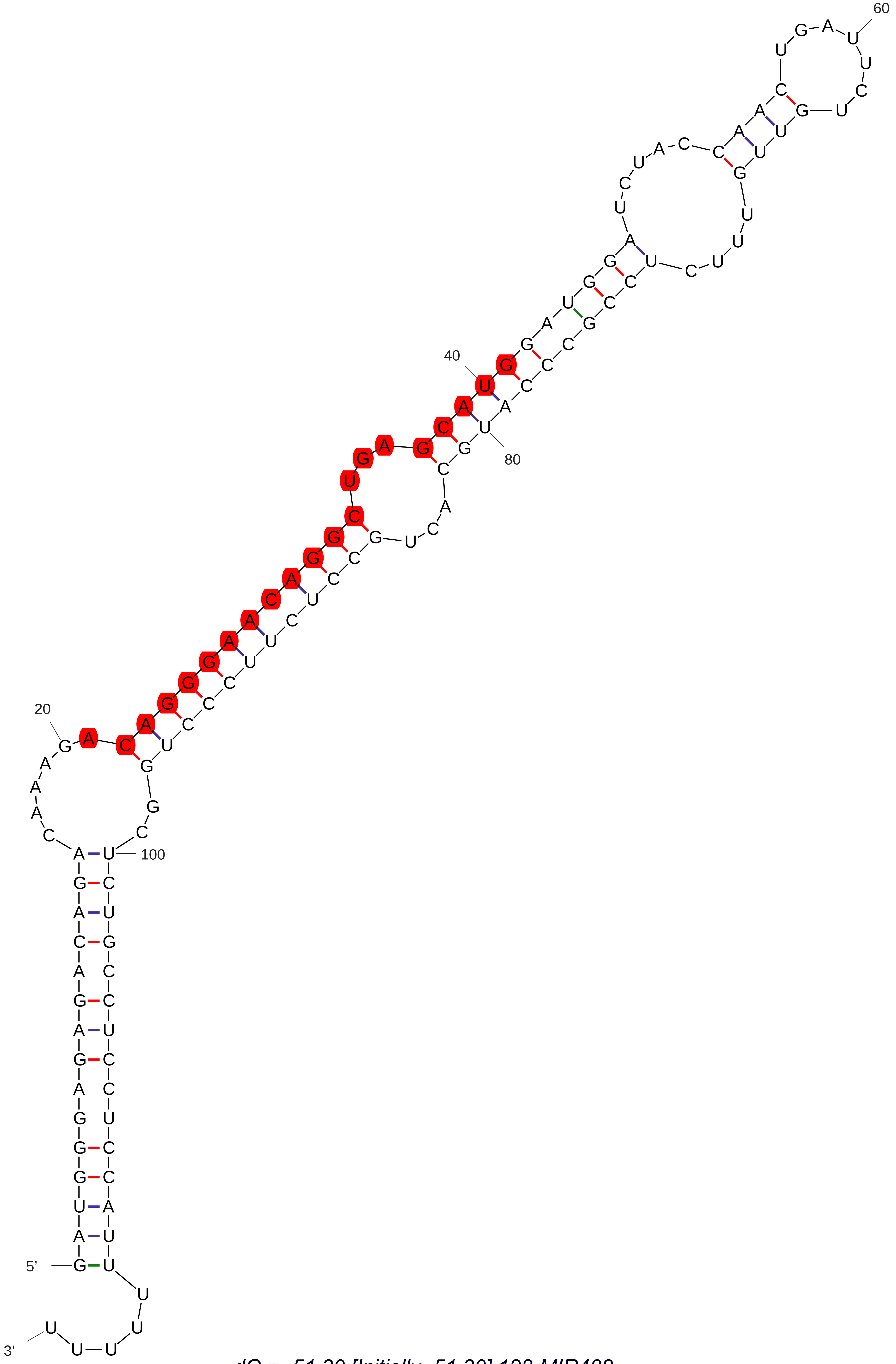

dG = -51.30 [Initially -51.30] 128-MIR408

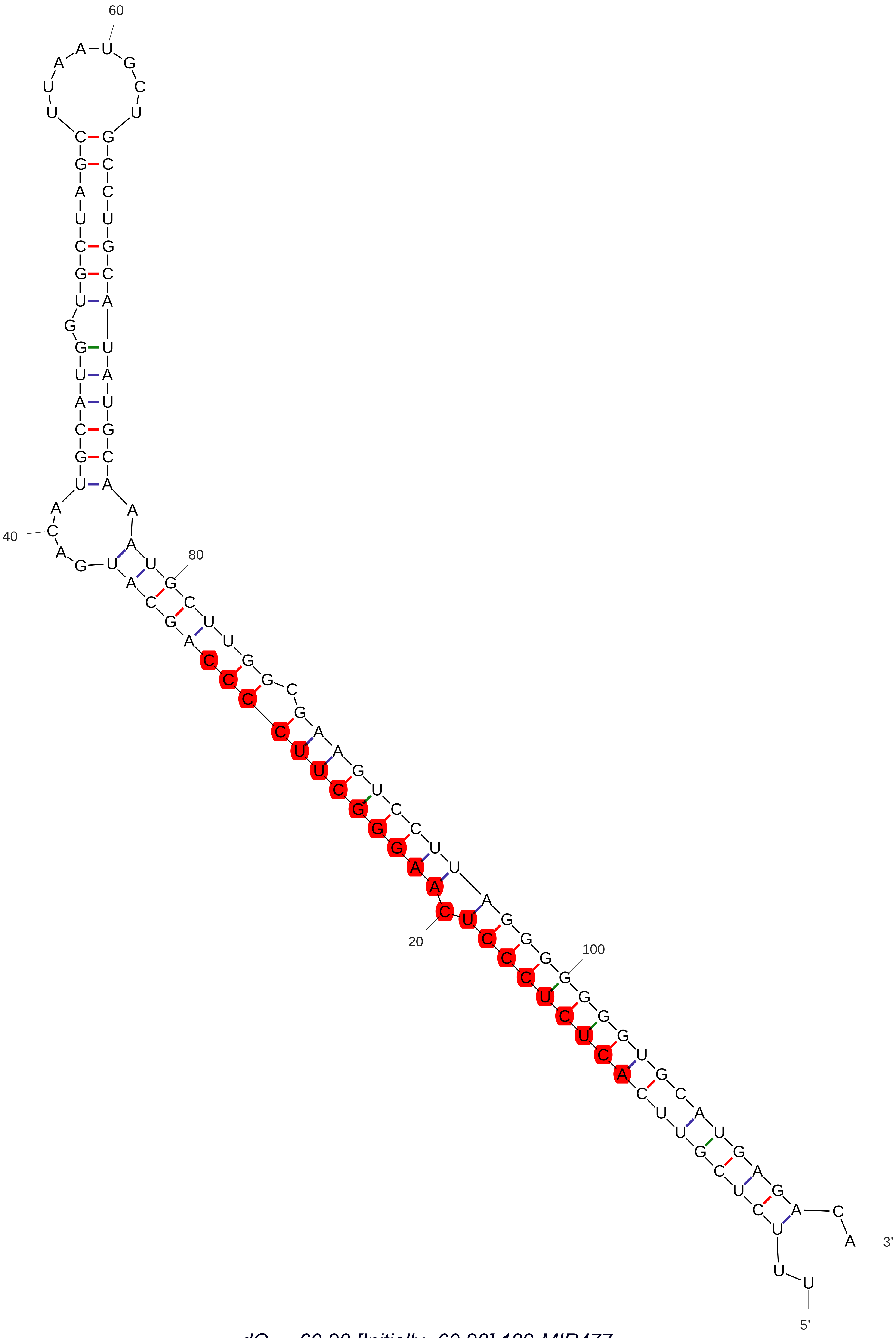

dG = -60.20 [Initially -60.20] 129-MIR477

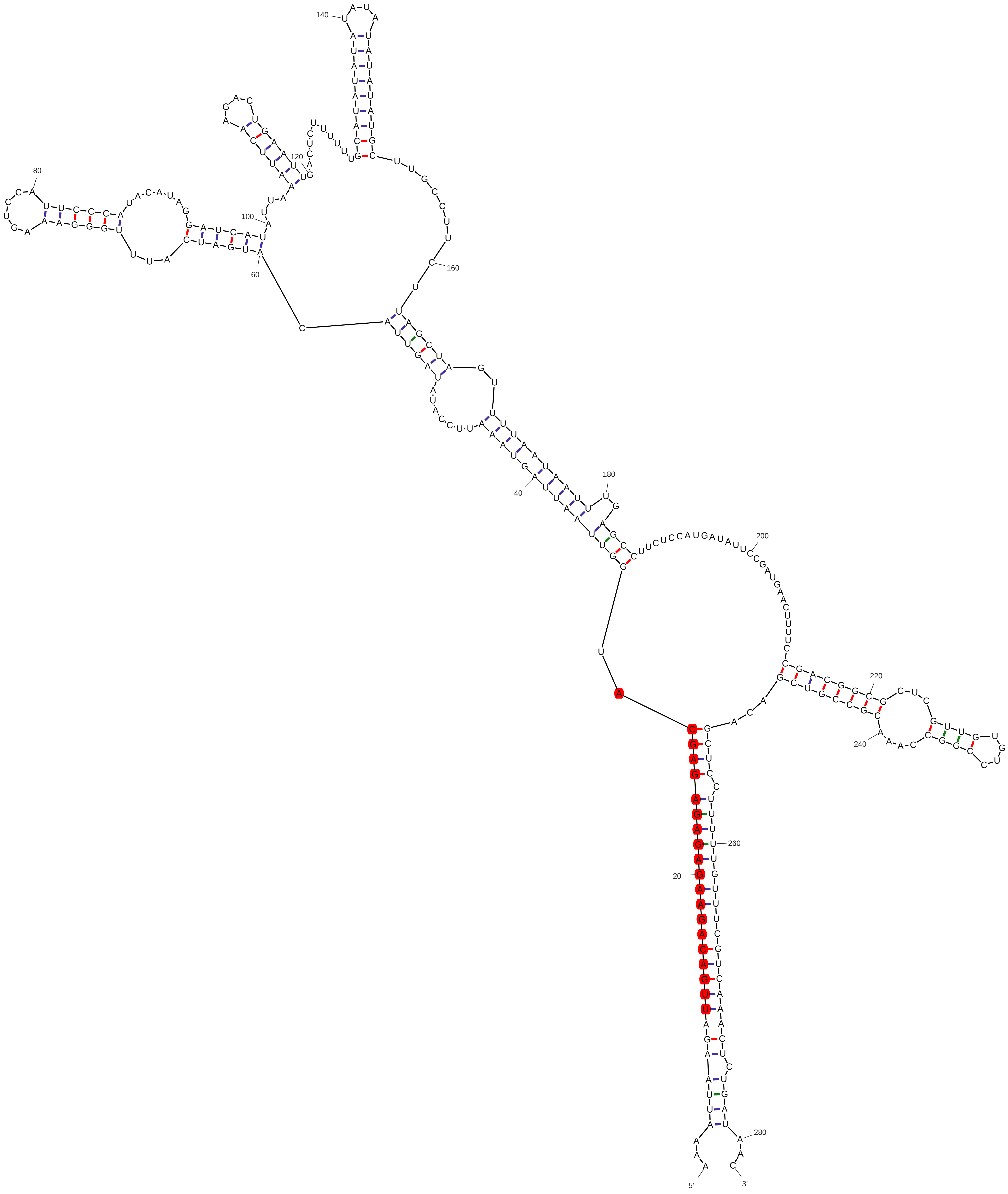

dG = -59.95 [Initially -67.70] 12-MIR156

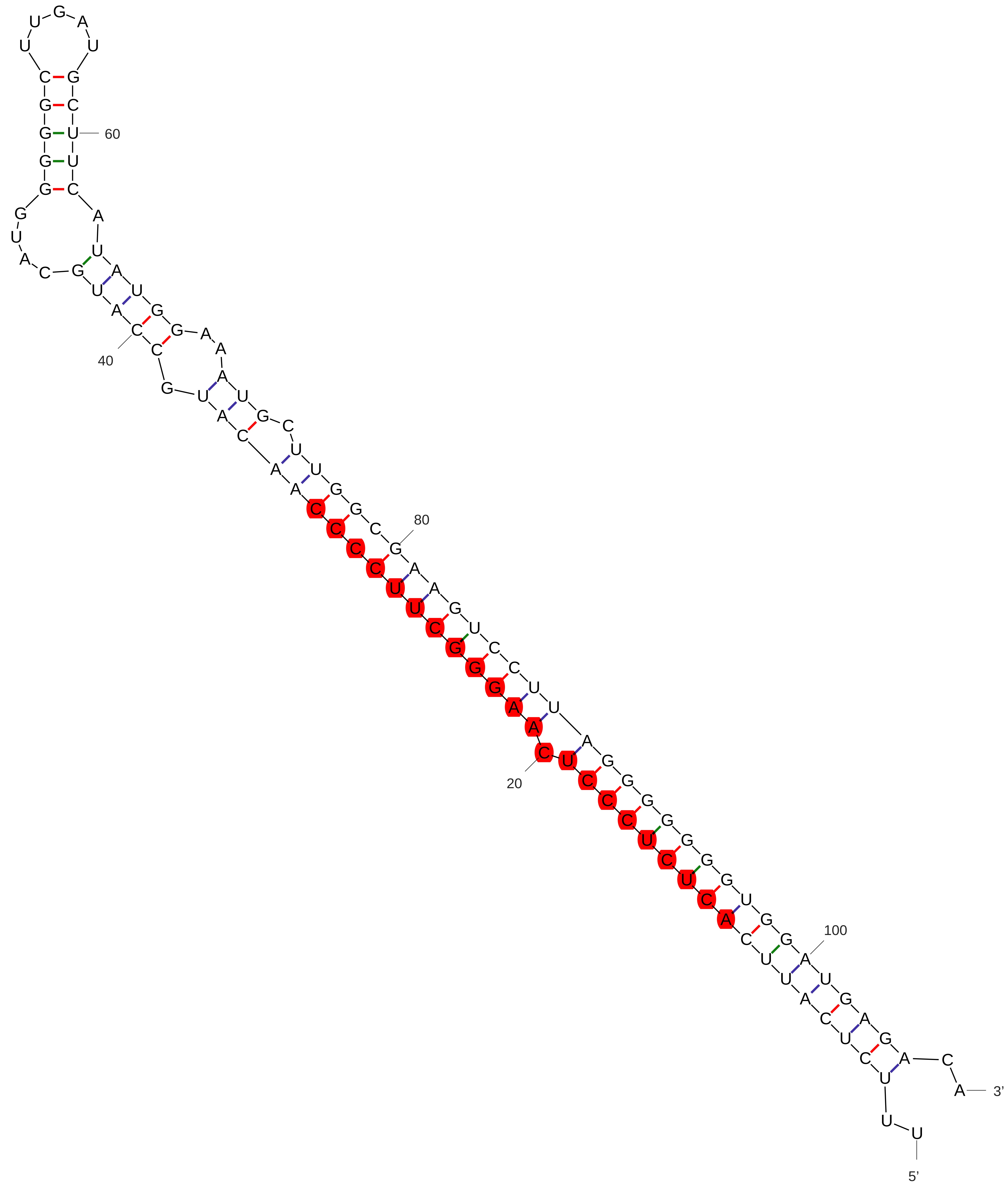

dG = -58.80 [Initially -58.80] 130-MIR477

Output of sir_graph (©)
mfold_util 4.6

Created Thu Jan 24 21:27:46 2013

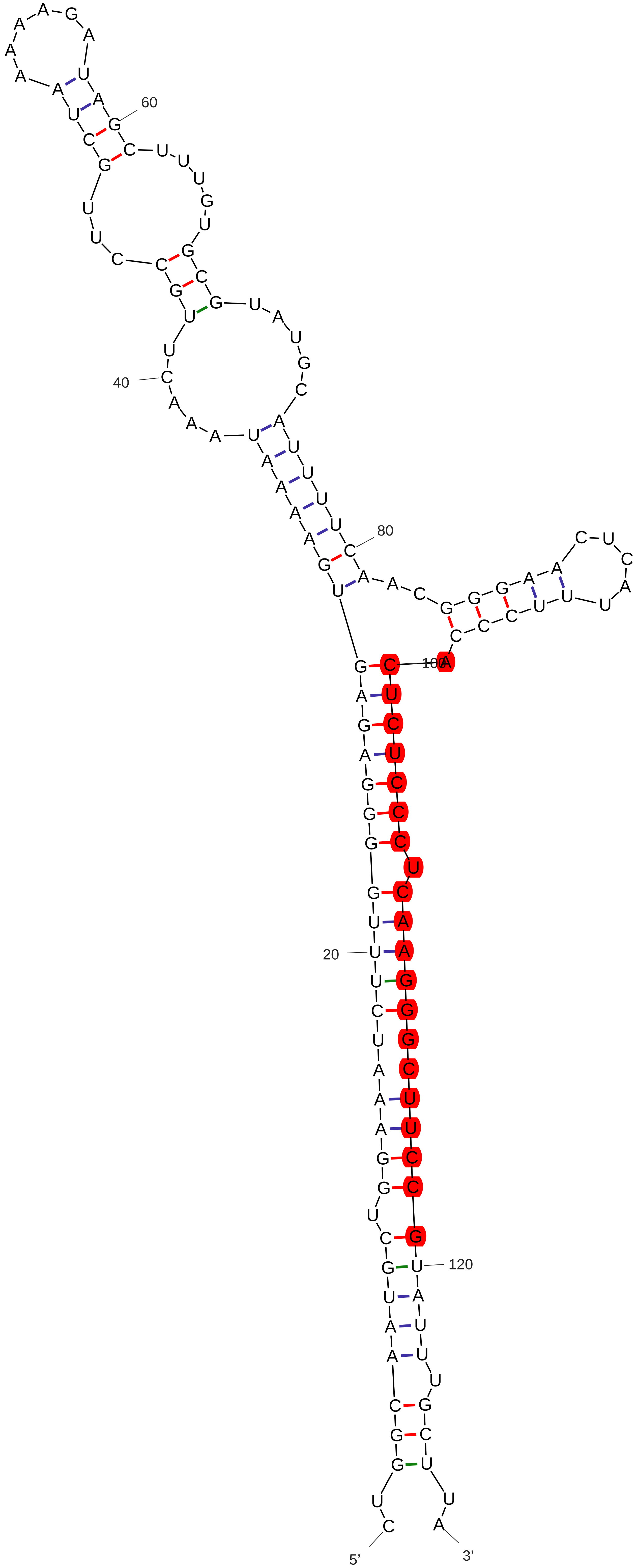

dG = -44.50 [Initially -47.10] 131-MIR477

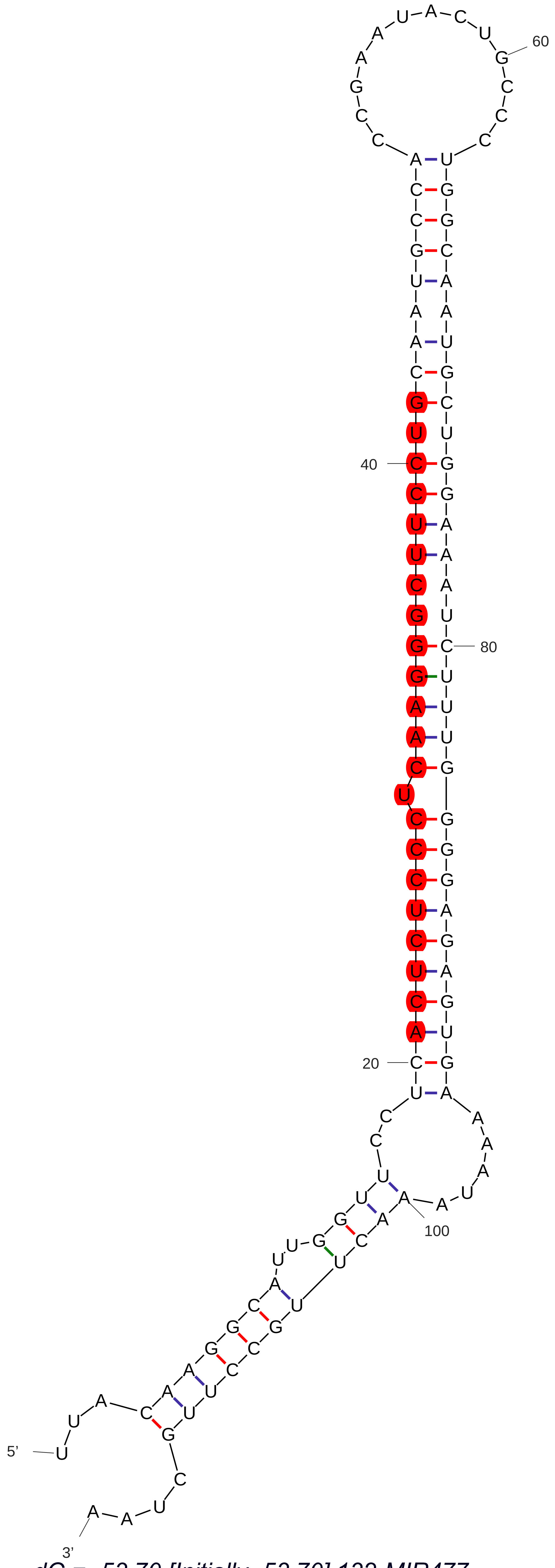

dG = -53.70 [Initially -53.70] 132-MIR477

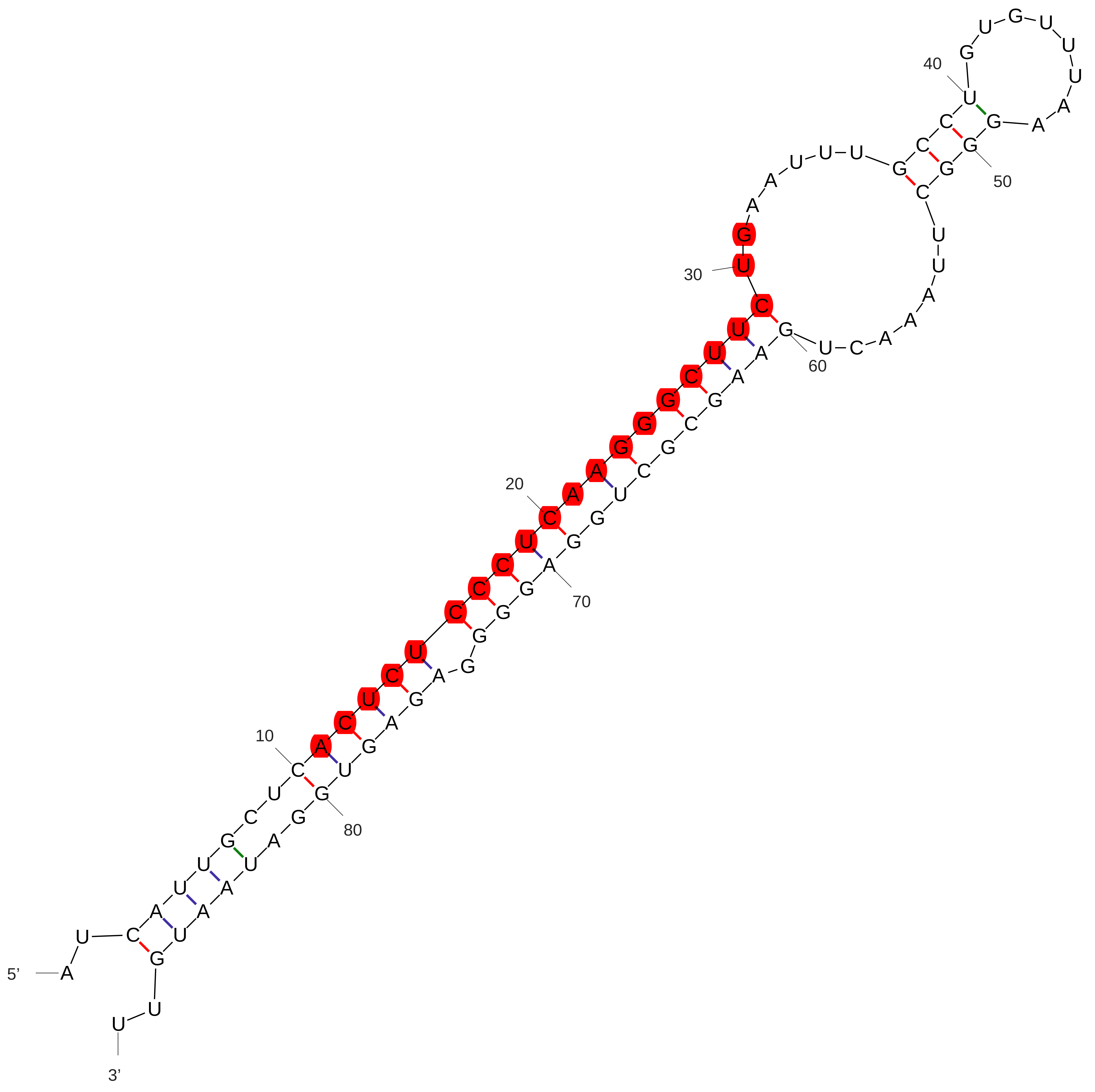

dG = -41.30 [Initially -41.30] 133-MIR477

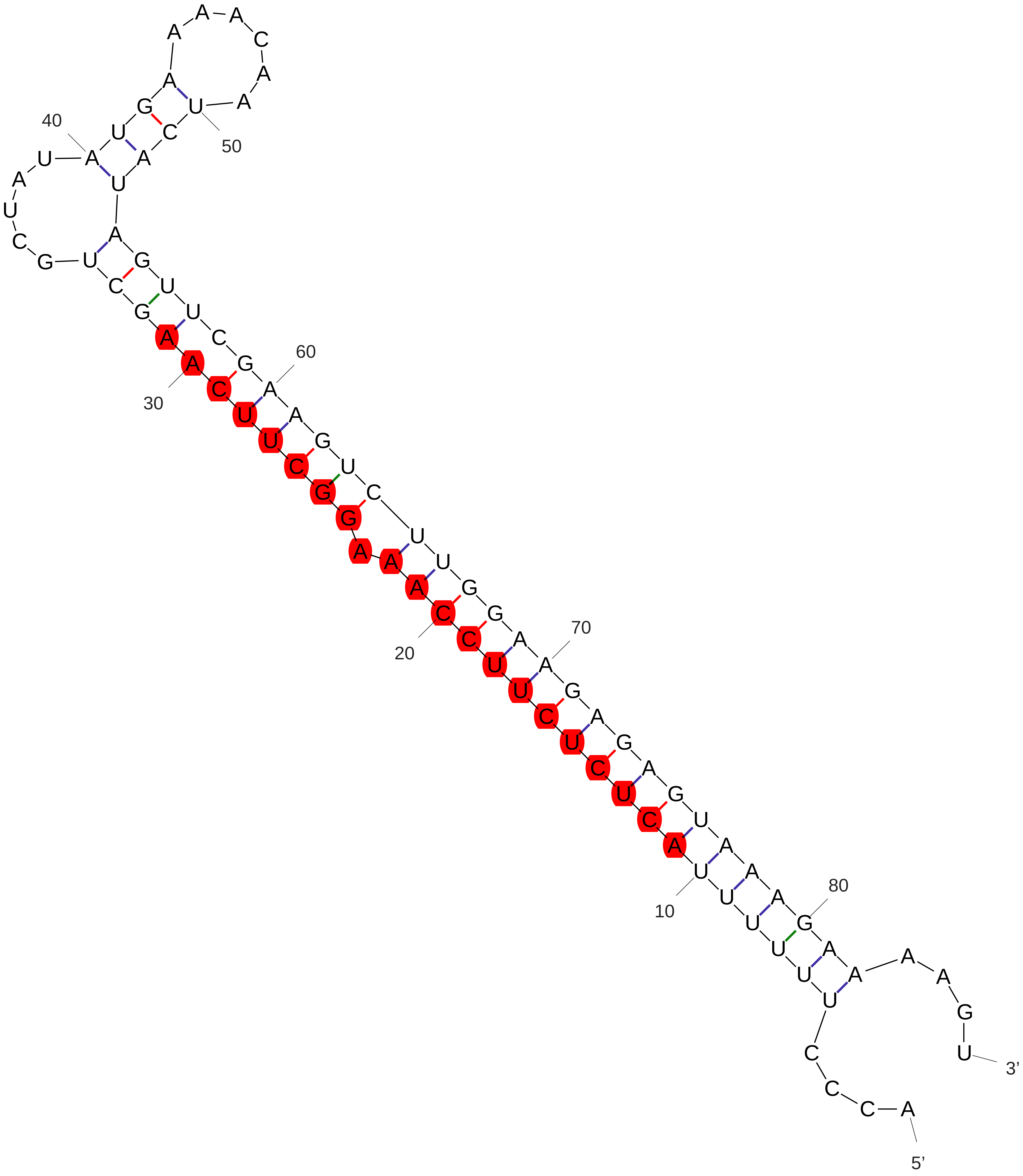

dG = -36.50 [Initially -36.50] 134-MIR477

Output of sir_graph (©)
mfold_util 4.6

Created Thu Jan 24 21:33:40 2013

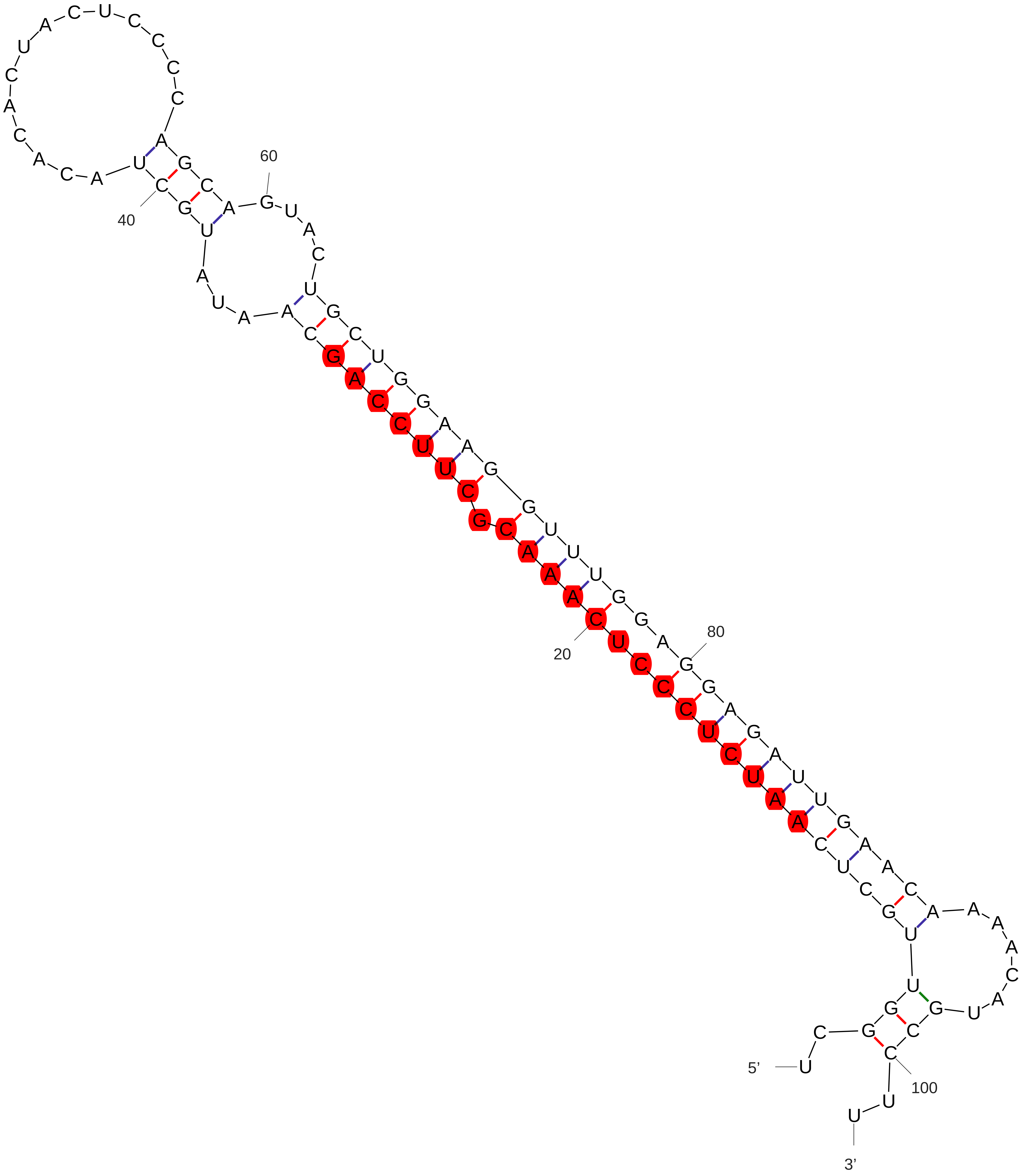

dG = -43.10 [Initially -43.10] 135-MIR477

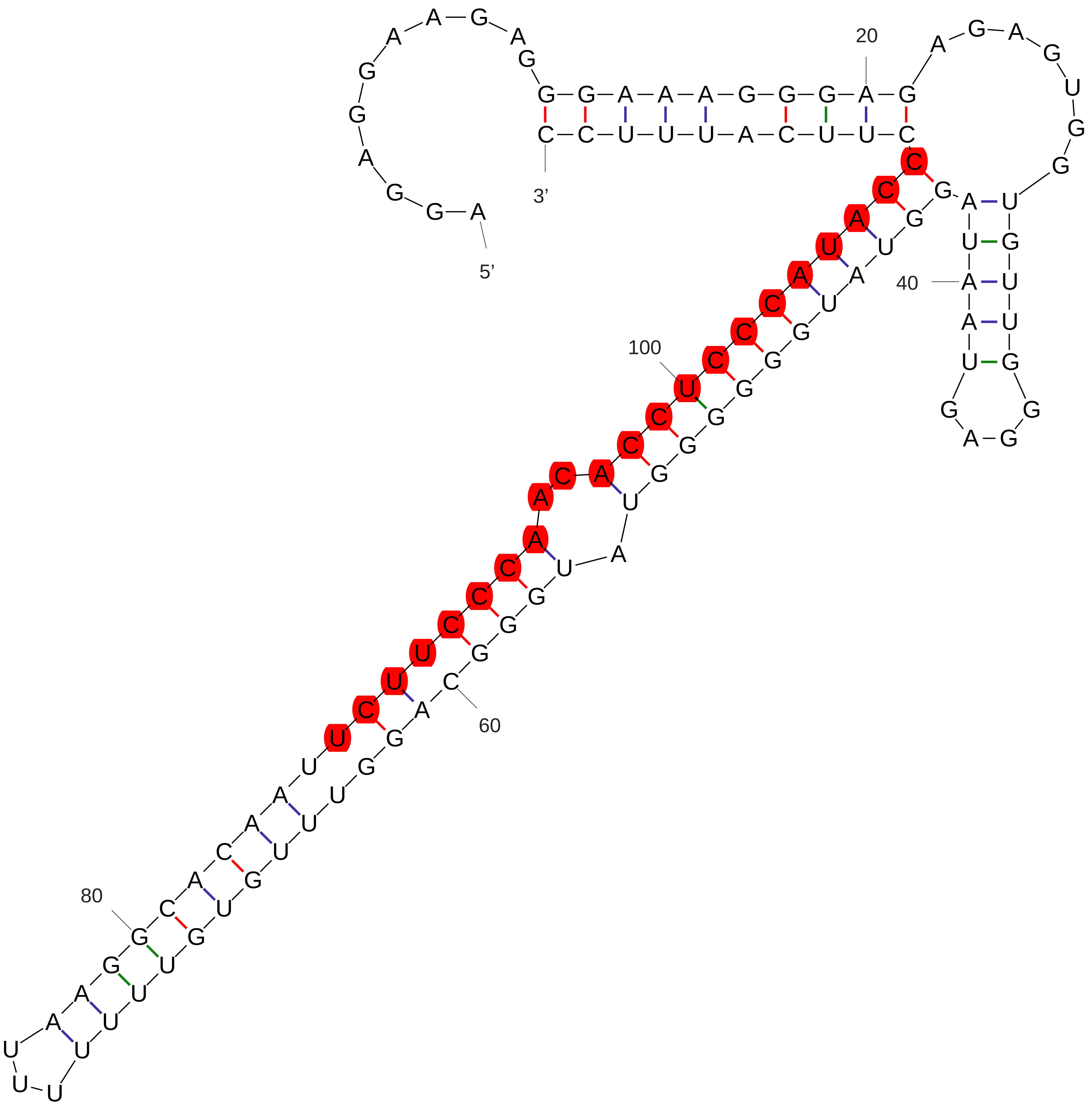

dG = -42.93 [Initially -43.70] 136-MIR482

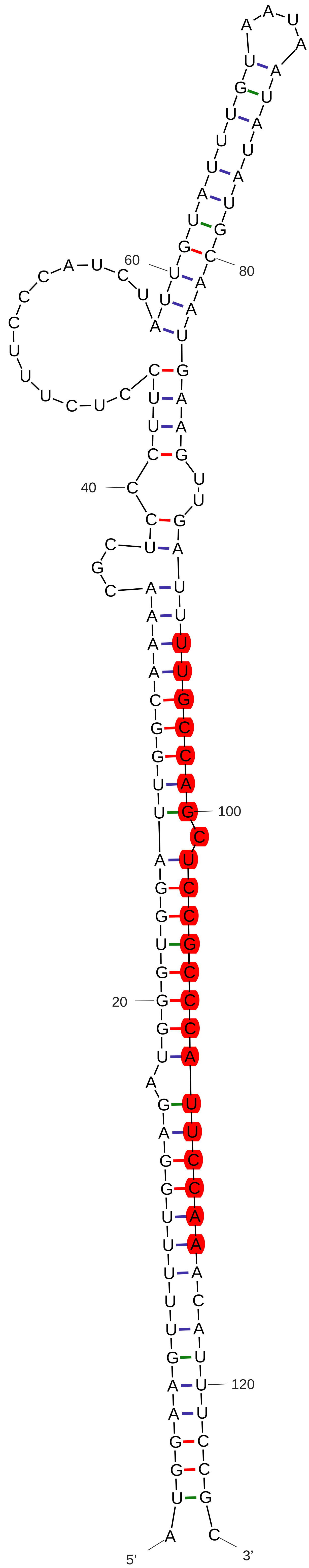

dG = -46.70 [Initially -46.70] 137-MIR482

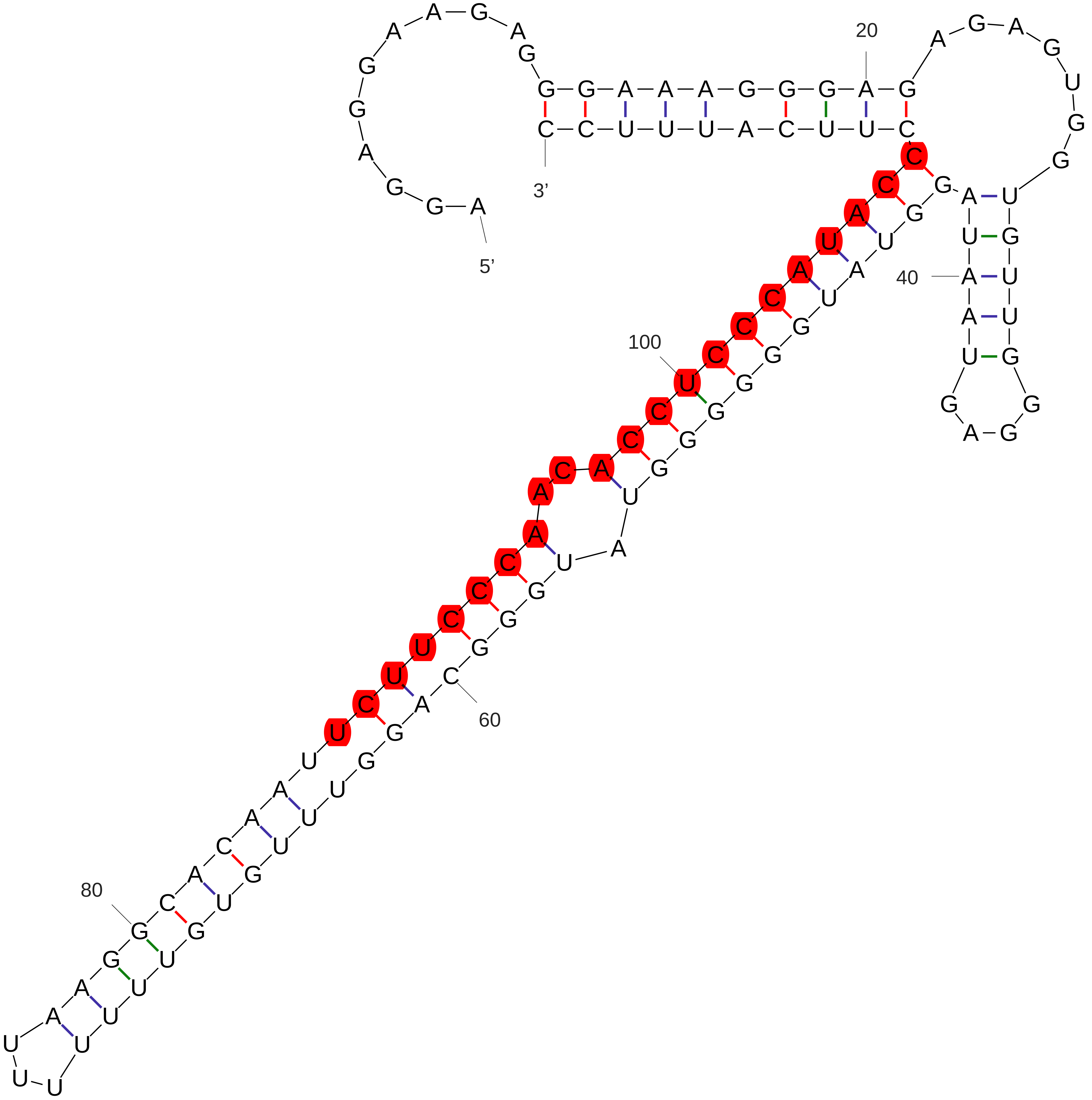

dG = -42.93 [Initially -43.70] 138-MIR482

Output of sir_graph (©)
mfold_util 4.6

Created Thu Jan 24 21:35:56 2013

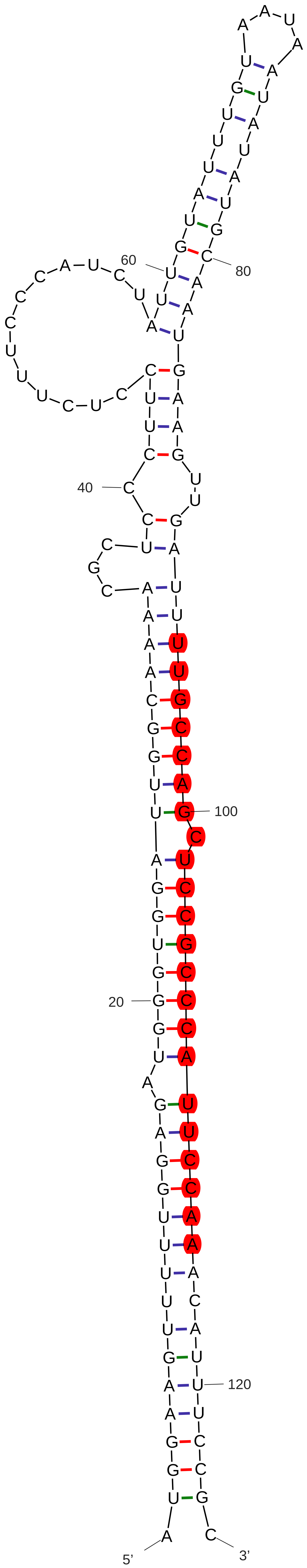

dG = -46.70 [Initially -46.70] 139-MIR482

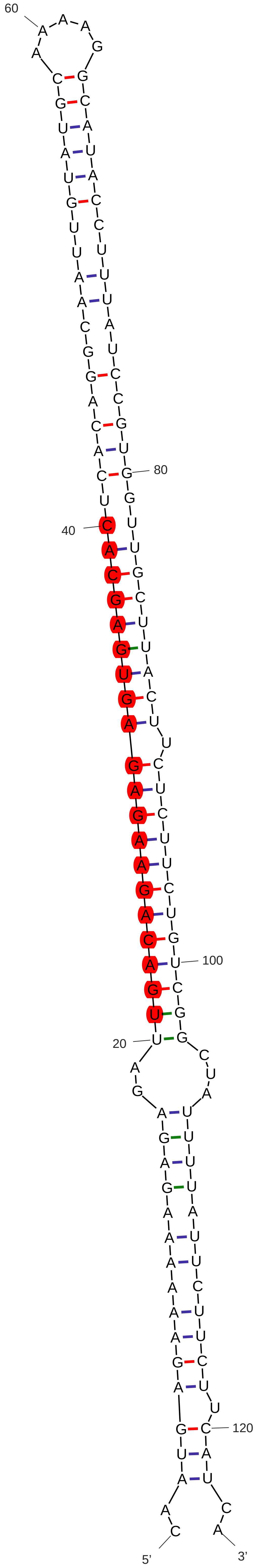

dG = -48.80 [Initially -48.80] 13-MIR156

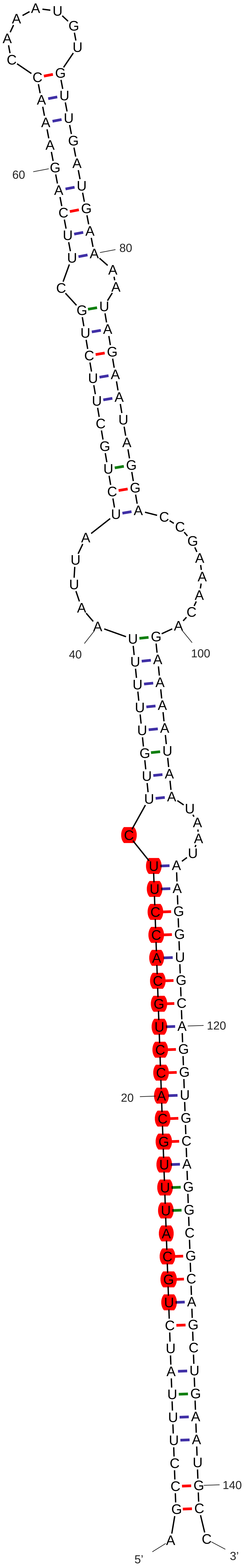

dG = -48.80 [Initially -48.80] 148-MIR530

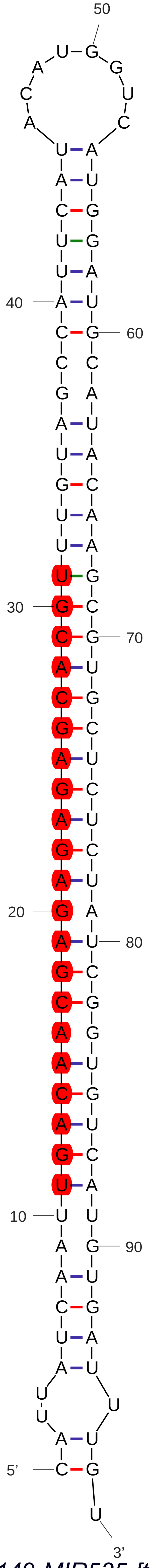

dG = -47.30 [Initially -47.30] 149-MIR535-[tcc-MIR535 MI0017532]

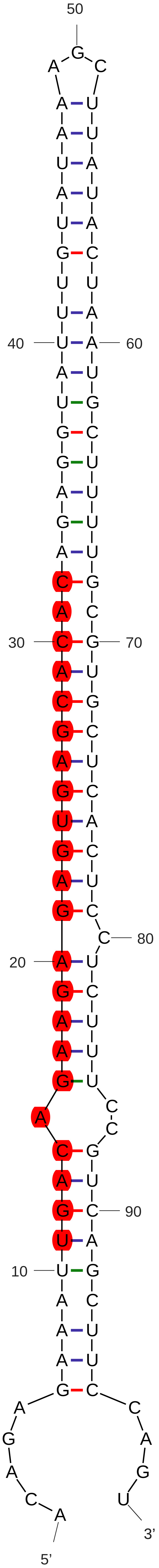

dG = -45.30 [Initially -45.50] 14-MIR156-[tcc-MIR156g MI0017459]

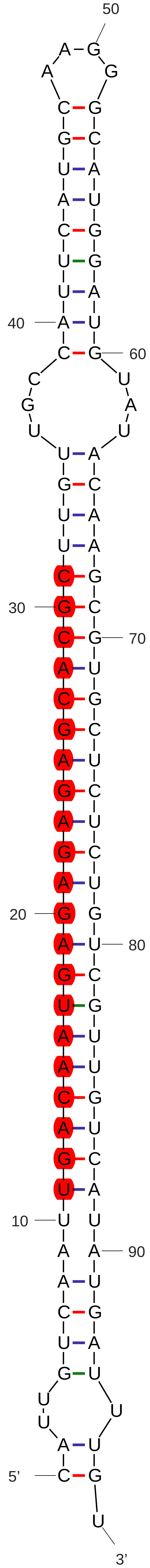

dG = -58.90 [Initially -58.90] 150-MIR535-[tcc-MIR535 MI0017532]

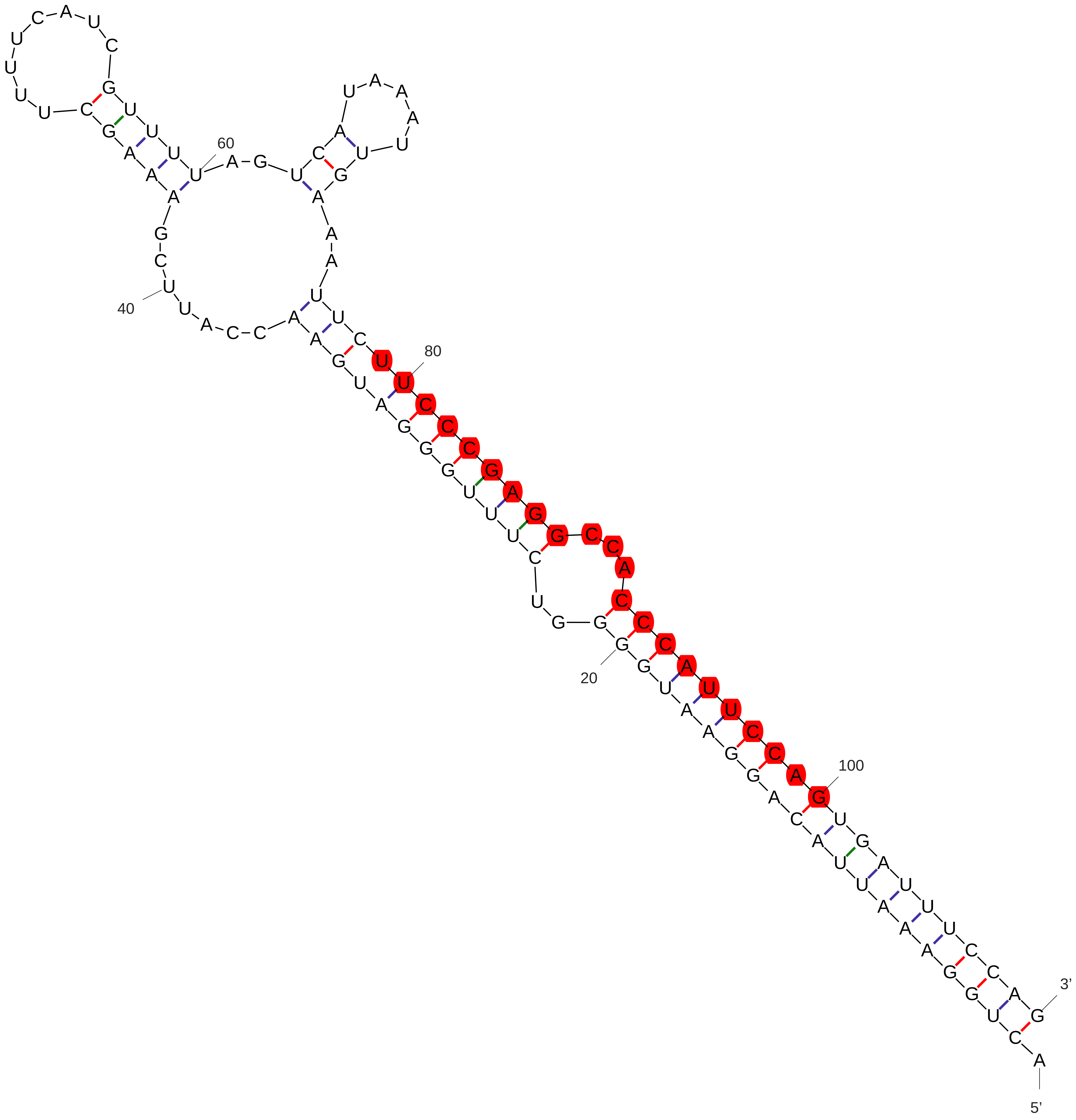

dG = -42.63 [Initially -46.00] 151-MIR6118

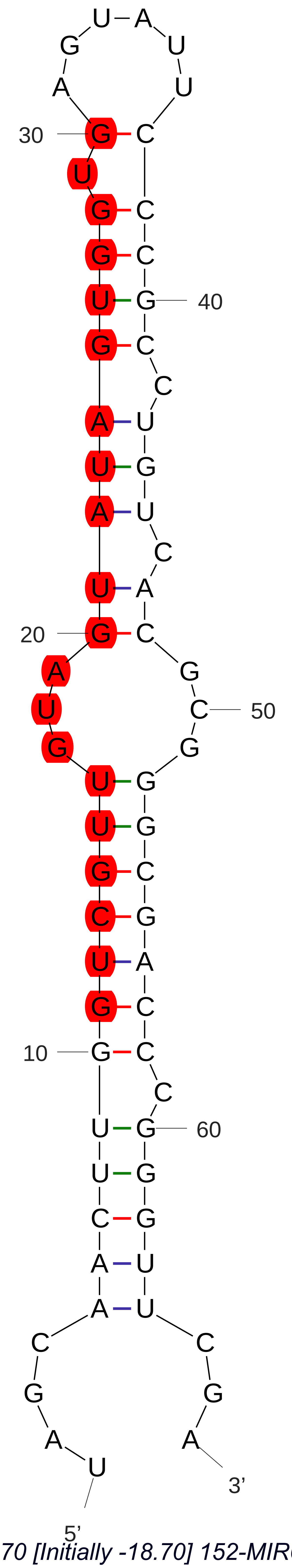

dG = -18.70 [Initially -18.70] 152-MIR6300

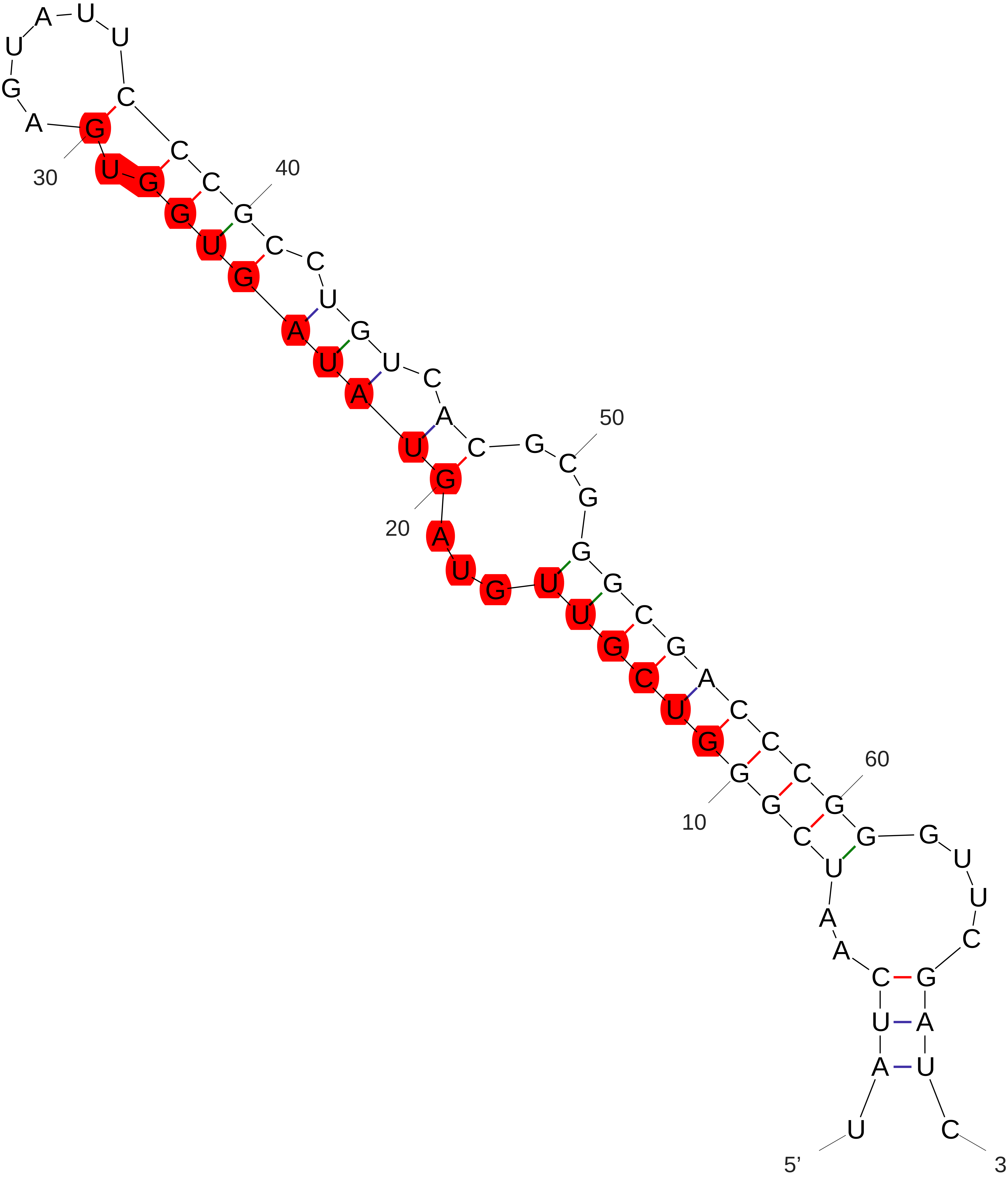

dG = -23.40 [Initially -23.40] 153-MIR6300

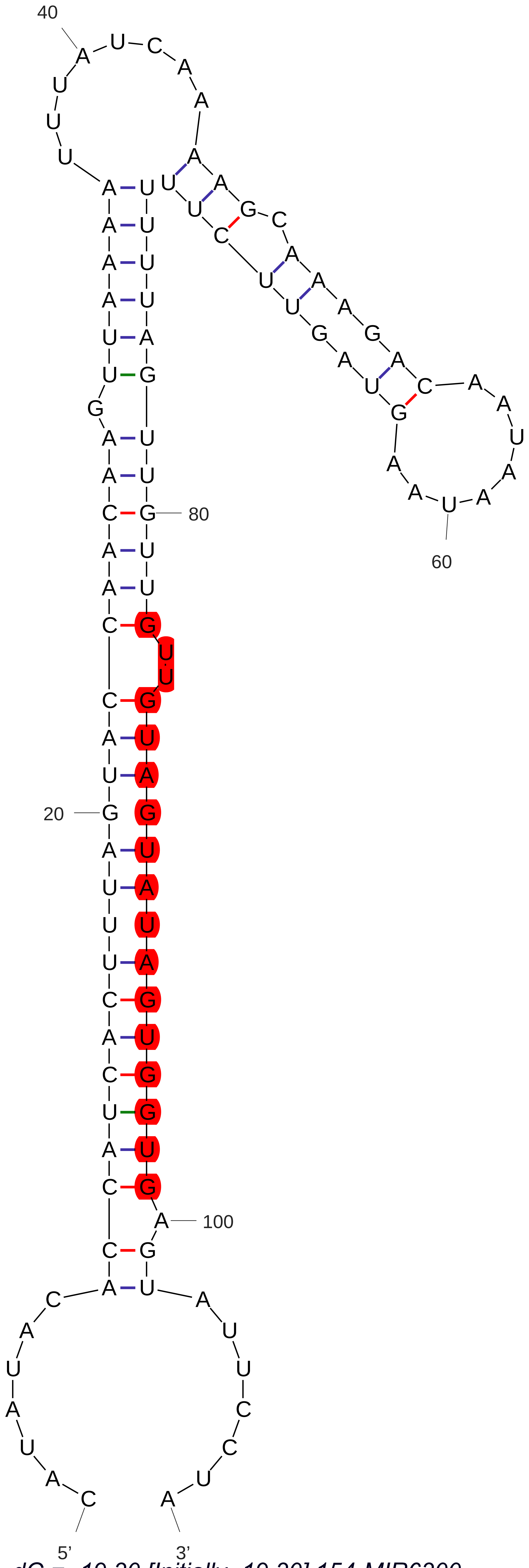

dG = -19.30 [Initially -19.30] 154-MIR6300

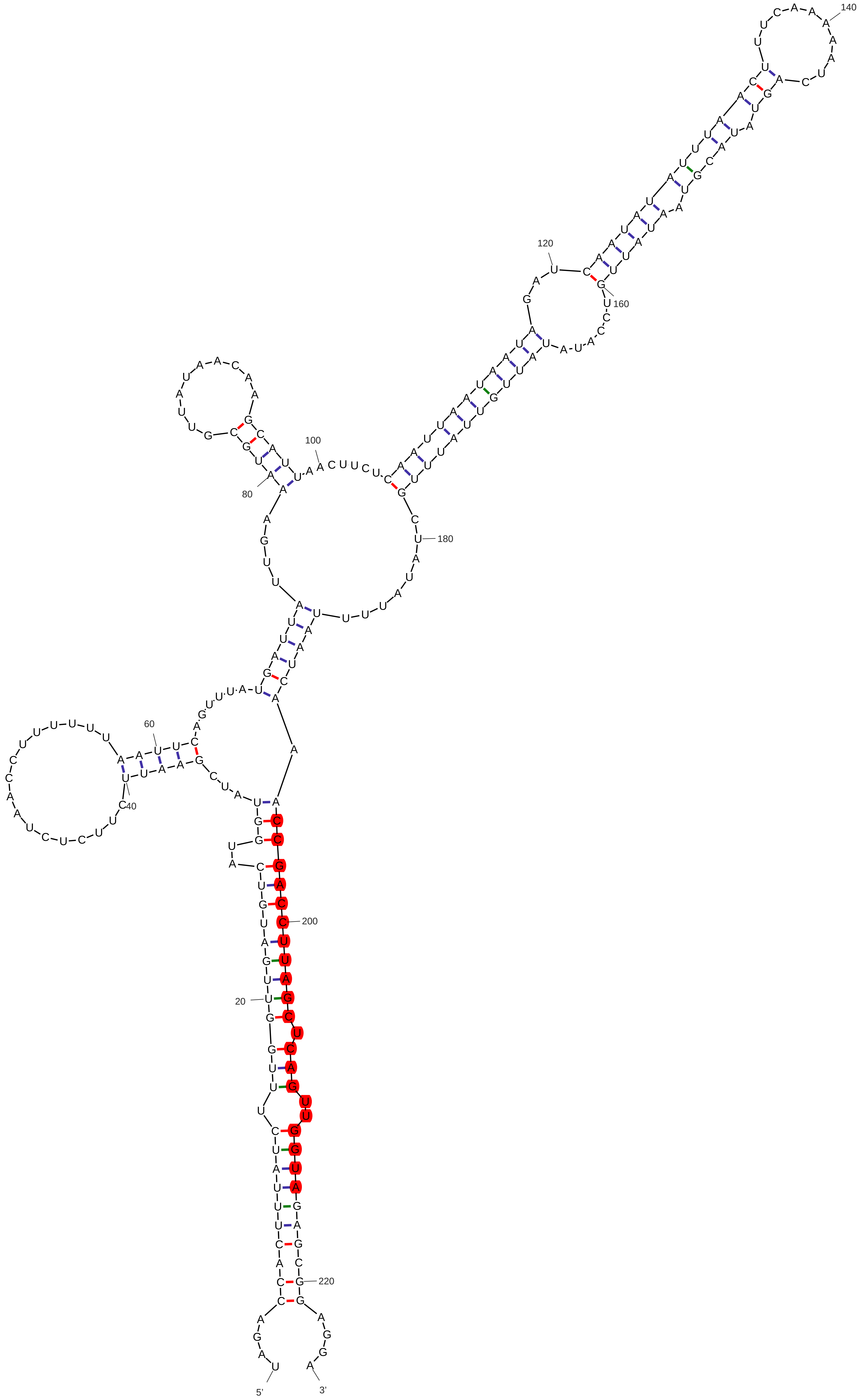

dG = -29.57 [Initially -36.30] 155-MIR6478

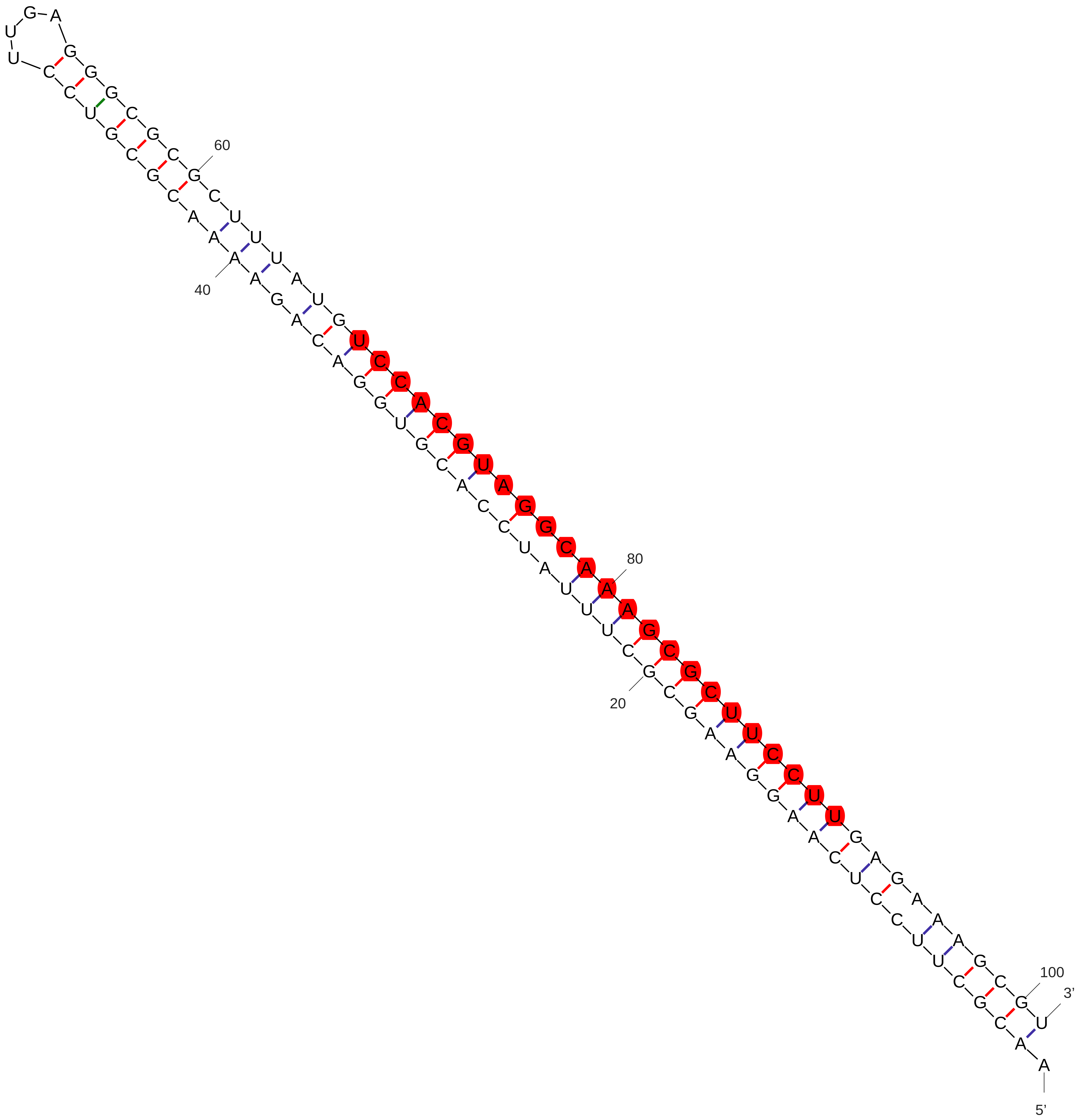

dG = -70.10 [Initially -70.10] 156-MIR7486

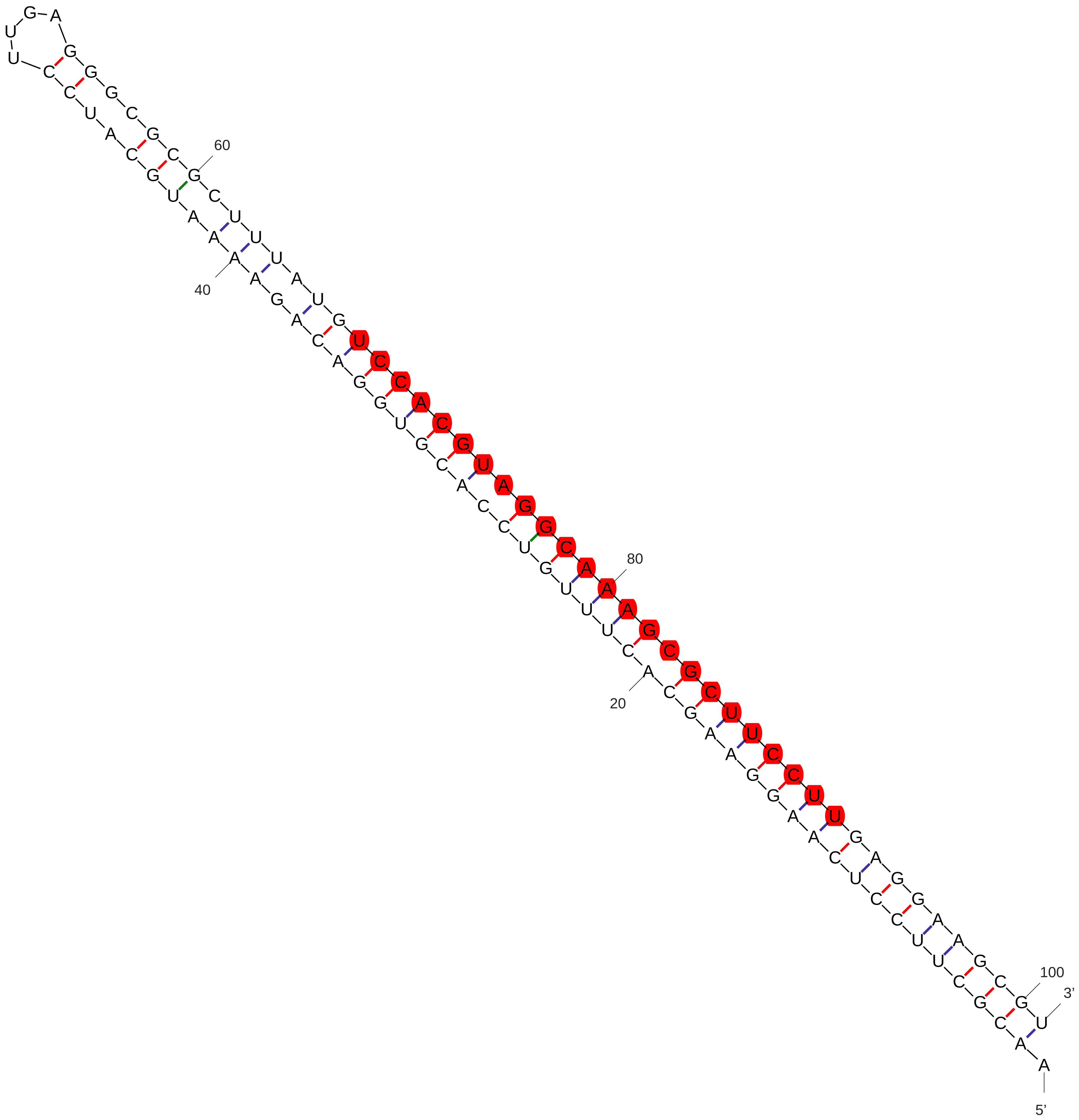

dG = -69.60 [Initially -69.60] 157-MIR7486

Output of sir_graph (©)
mfold_util 4.6

Created Fri Jan 25 03:00:00 2013

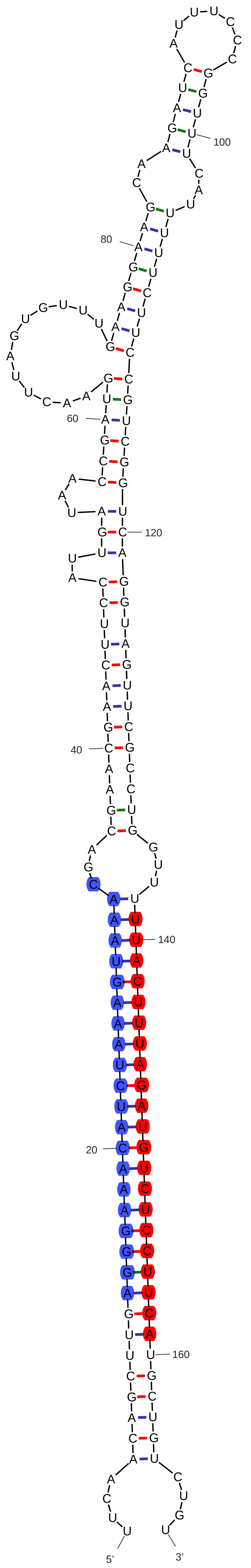

dG = -60.20 [Initially -60.20] 158-MIR7495

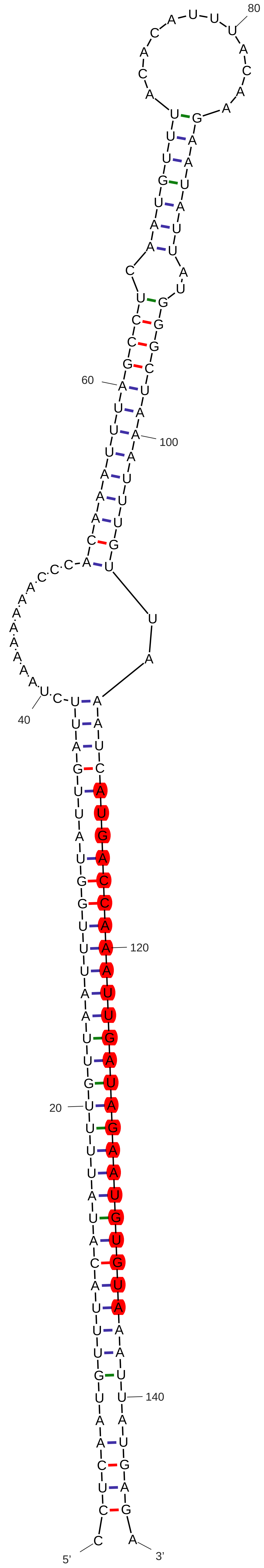

dG = -50.90 [Initially -50.90] 159-MIR7496

Output of sir_graph (©)
mfold_util 4.6

Created Fri Jan 25 11:26:03 2013

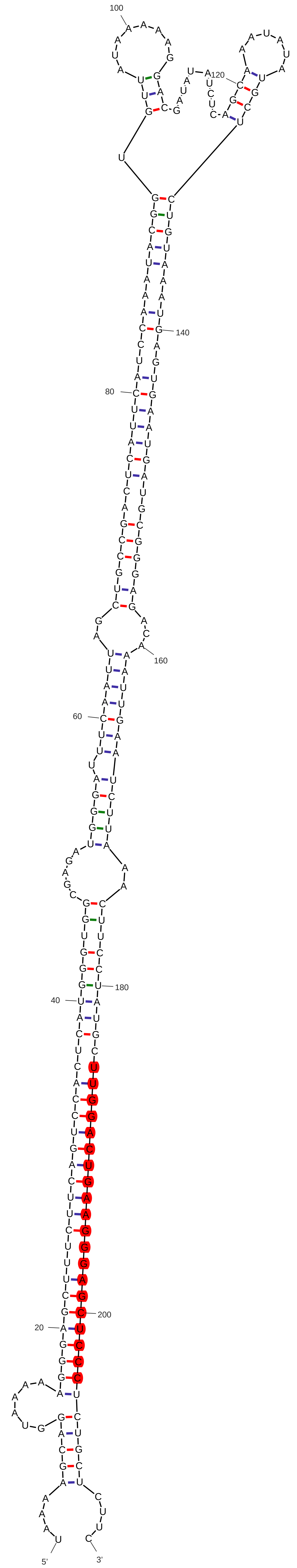

dG = -82.63 [Initially -84.60] 15-MIR159-[ctr-MIR319 MI0013303]

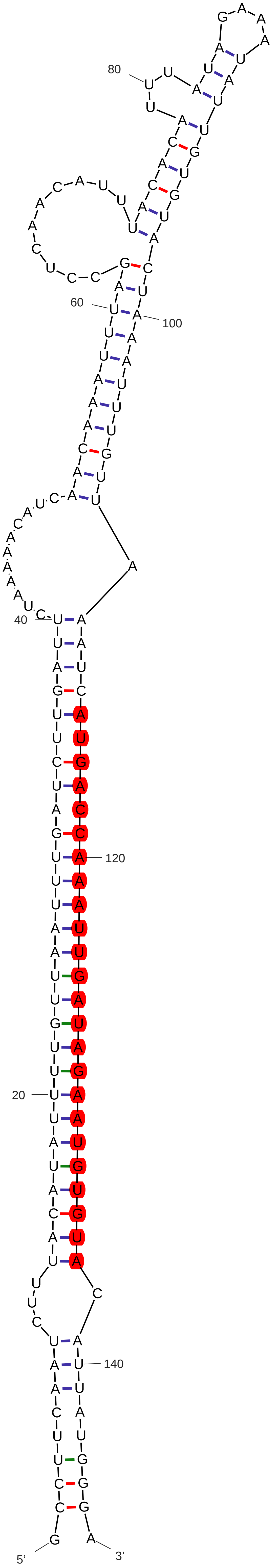

dG = -38.30 [Initially -38.30] 160-MIR7496

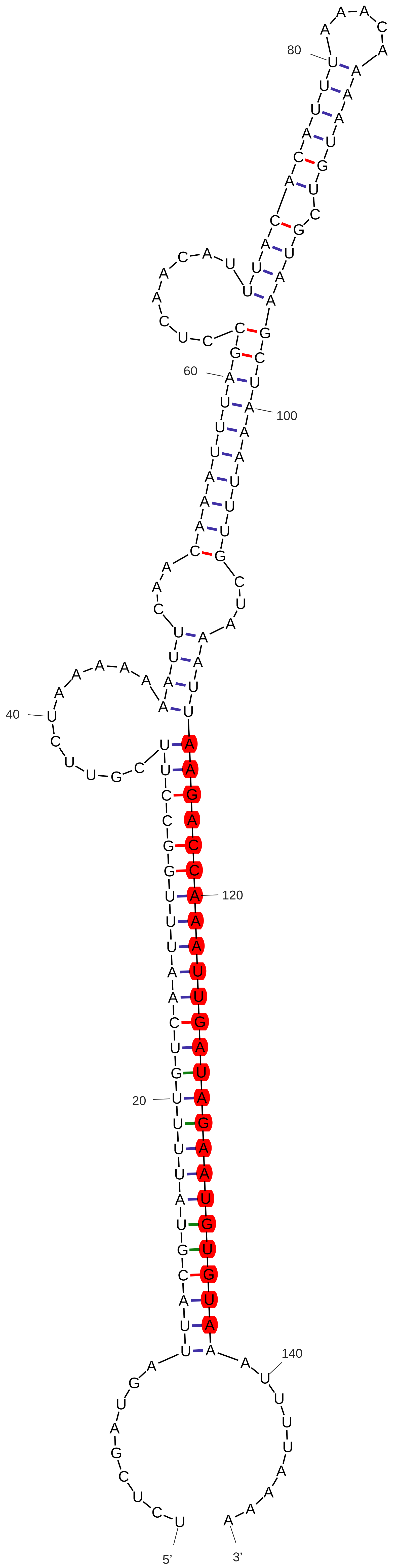

dG = -36.10 [Initially -36.10] 161-MIR7496

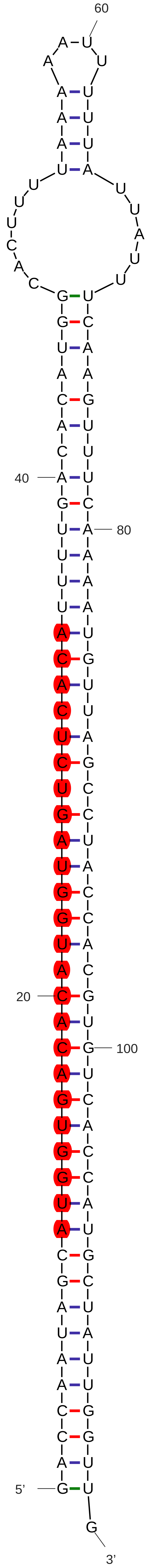

dG = -59.20 [Initially -59.20] 163-MIR7498

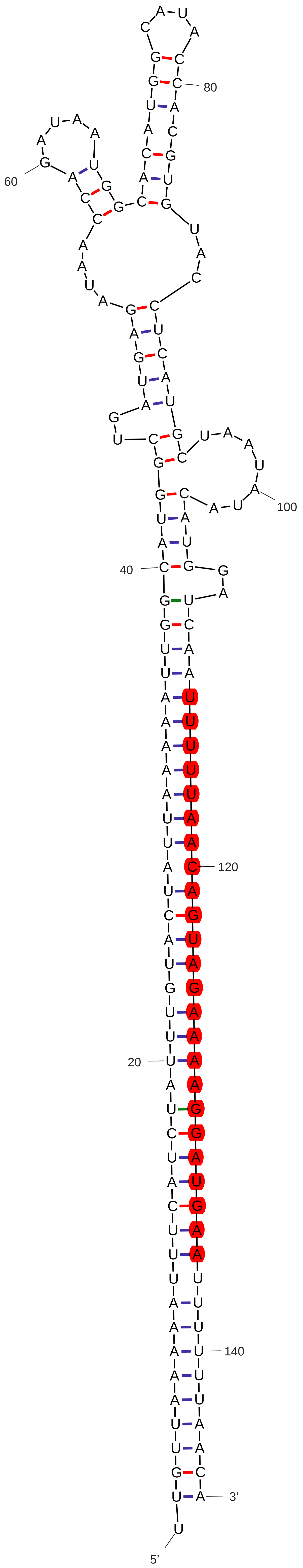

dG = -42.93 [Initially -43.50] 164-MIR7502

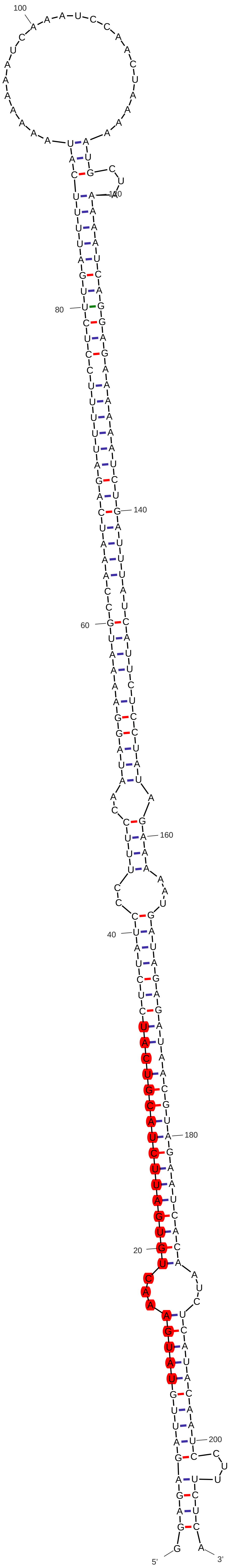

dG = -86.30 [Initially -86.30] 165-MIR7504a

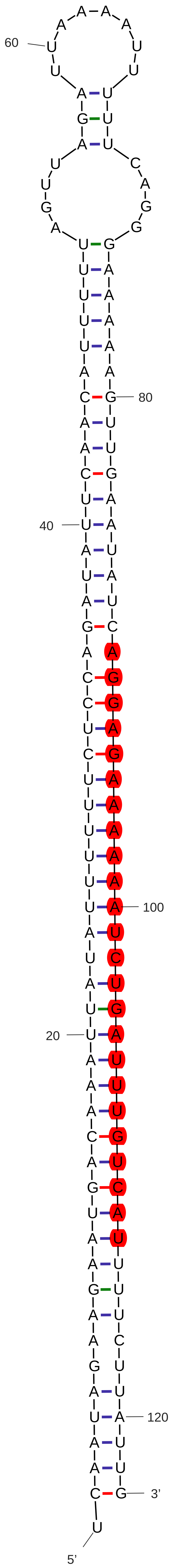

dG = -47.60 [Initially -47.60] 166-MIR7504b

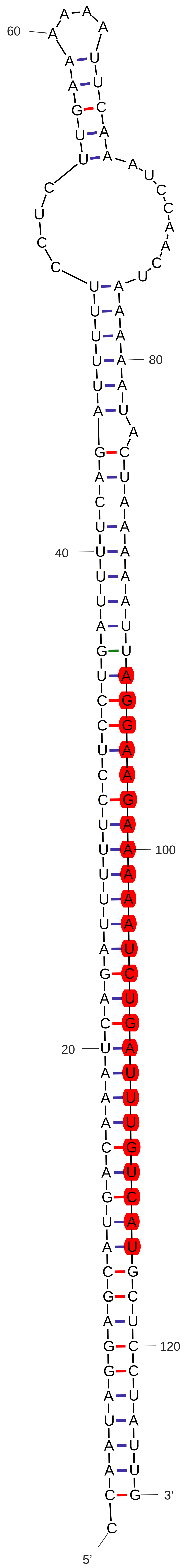

dG = -61.40 [Initially -61.40] 167-MIR75047b

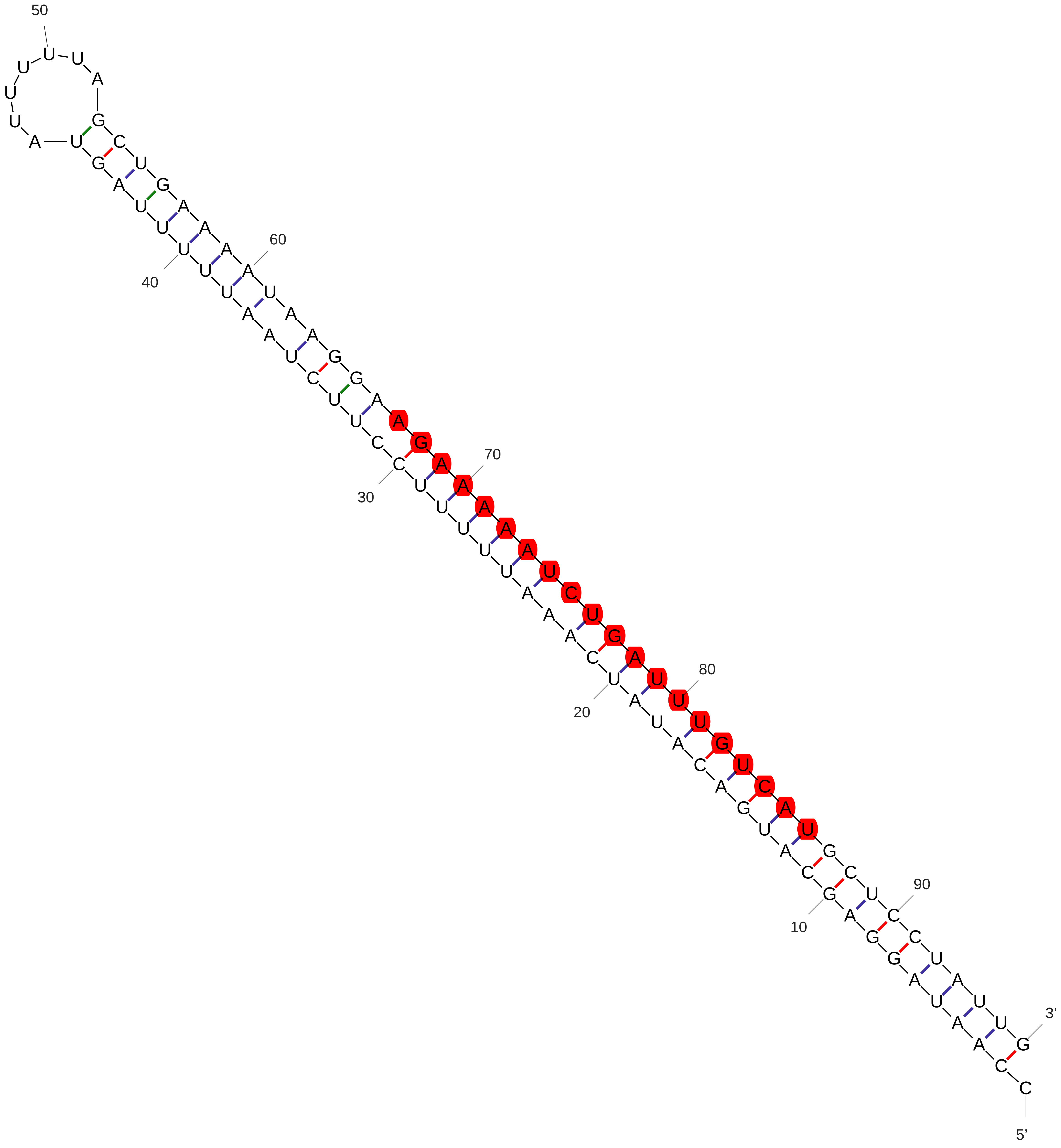

dG = -47.90 [Initially -47.90] 168-MIR7504b

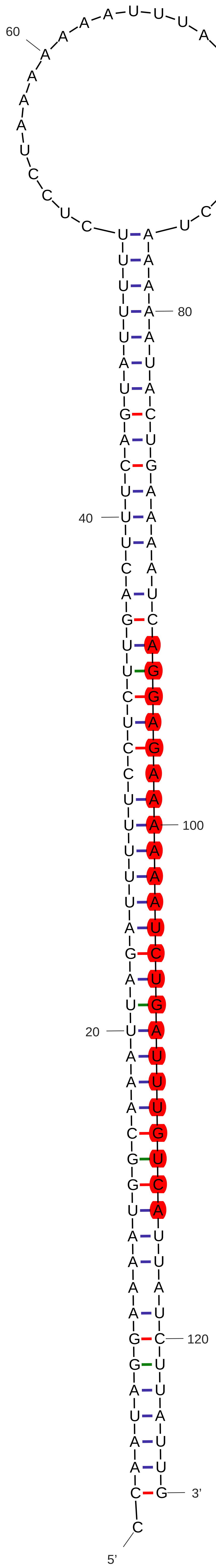

dG = -51.70 [Initially -51.70] 169-MIR7504b

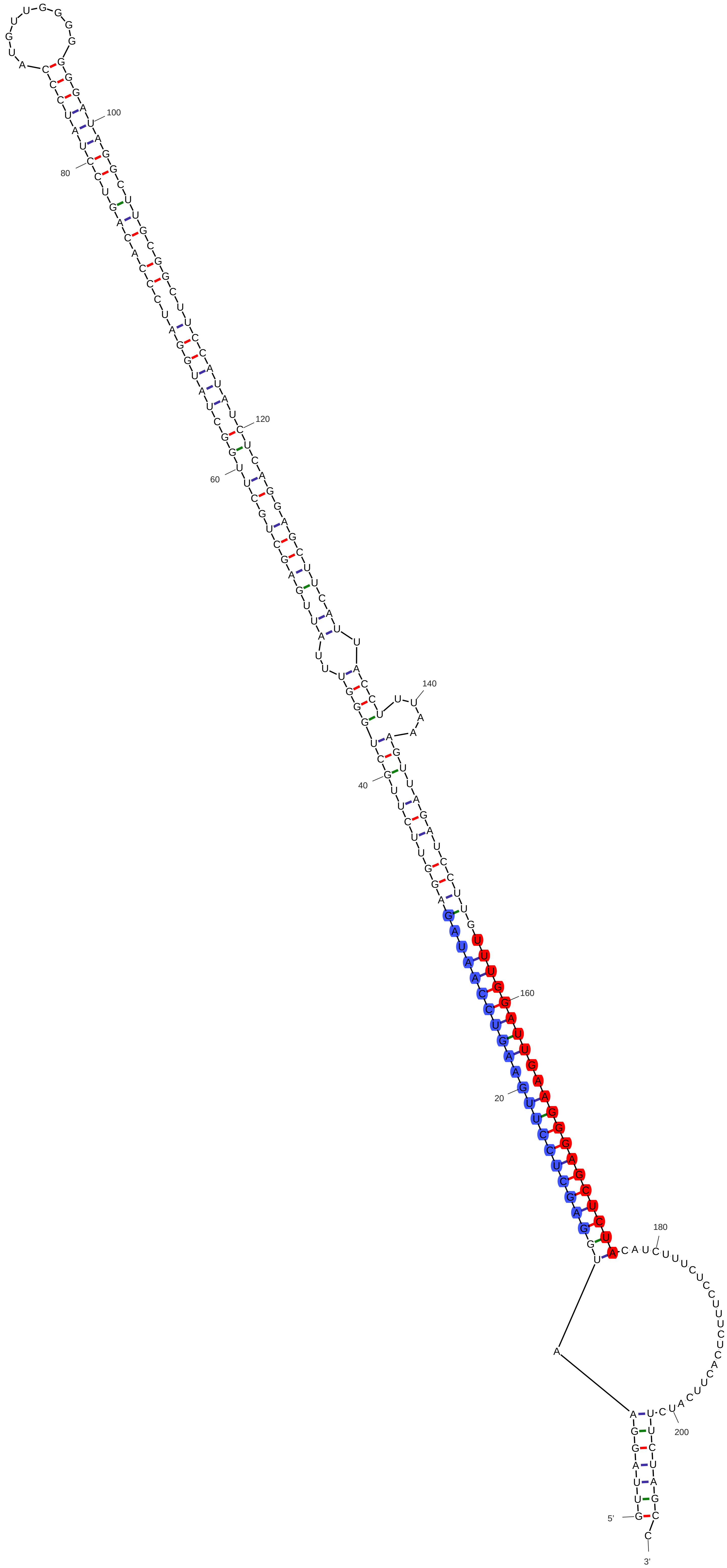

dG = -81.90 [Initially -81.90] 16-MIR159-[hbr-MIR159a MI0022053]

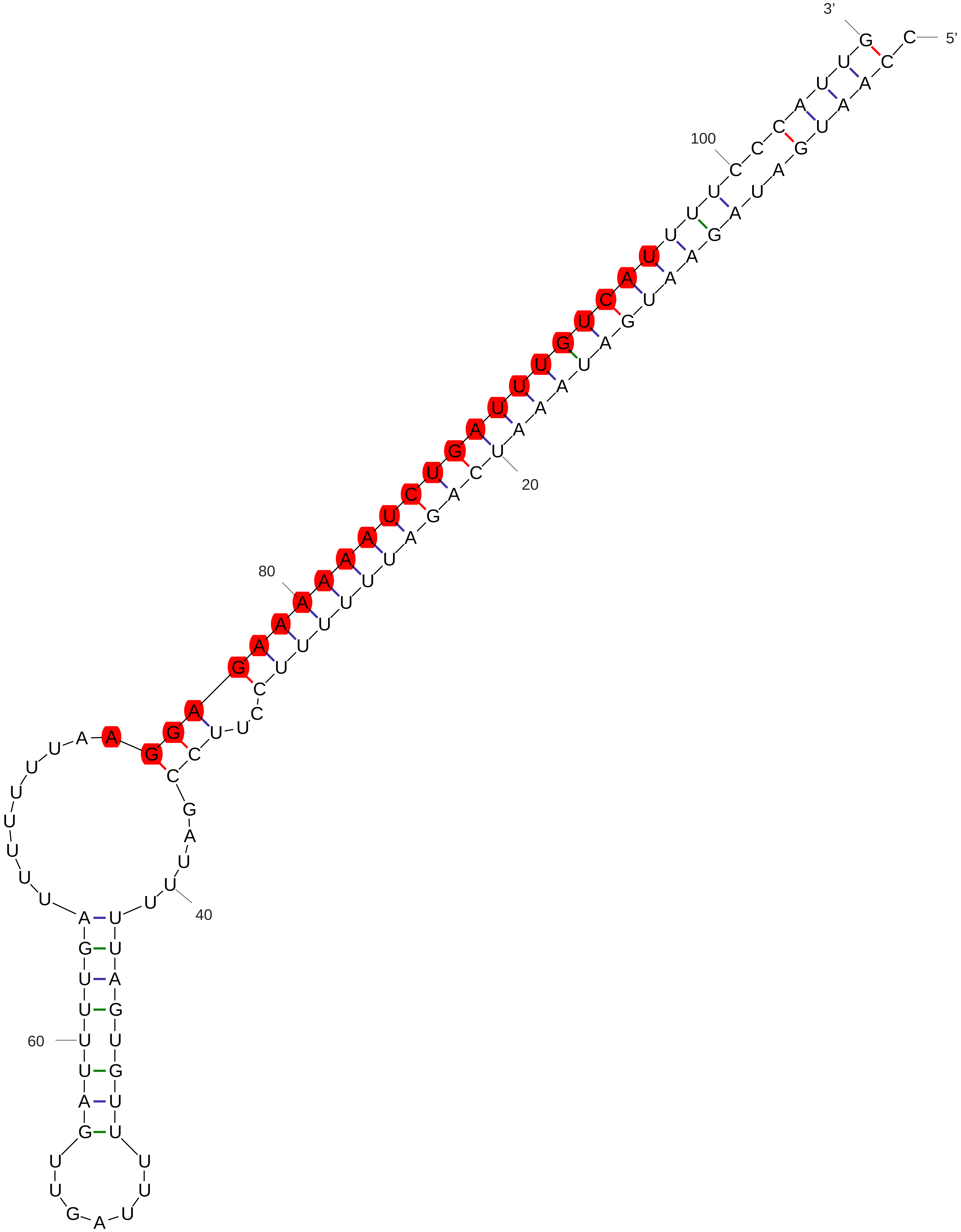

dG = -33.10 [Initially -33.10] 170-MIR7504b

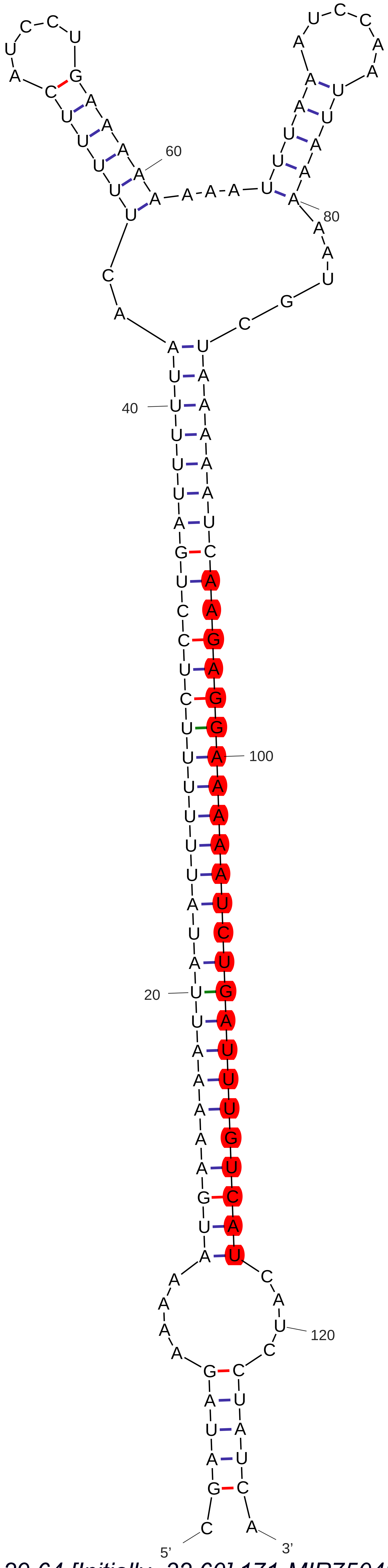

dG = -29.64 [Initially -32.60] 171-MIR7504b

Output of sir_graph (©)
mfold_util 4.6

Created Thu Jan 24 19:54:47 2013

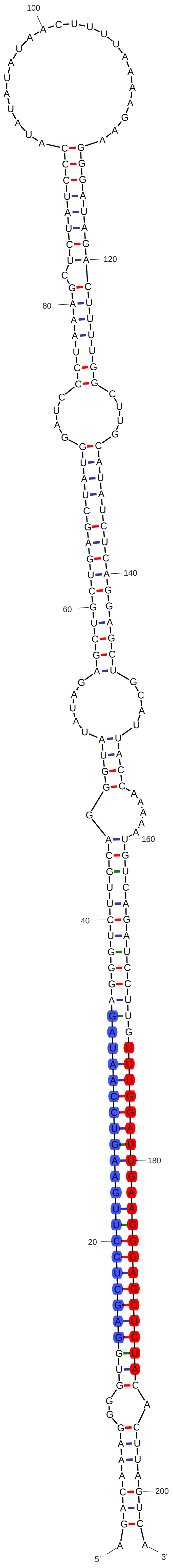

dG = -87.80 [Initially -87.80] 17-MIR159-[hbr-MIR159a MI0022053]

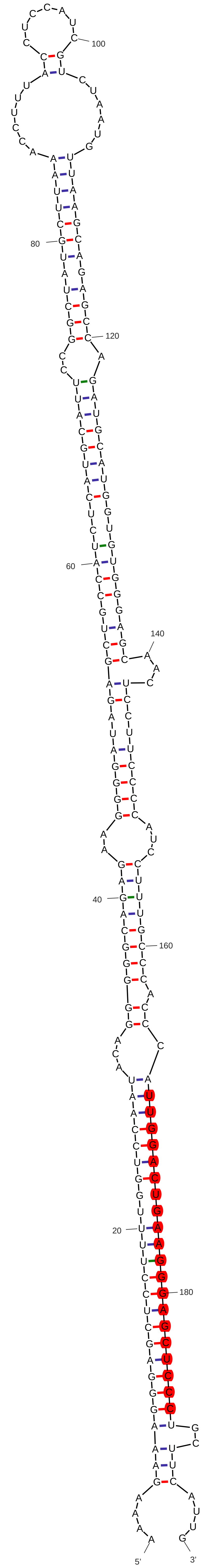

dG = -104.20 [Initially -104.20] 18-MIR159

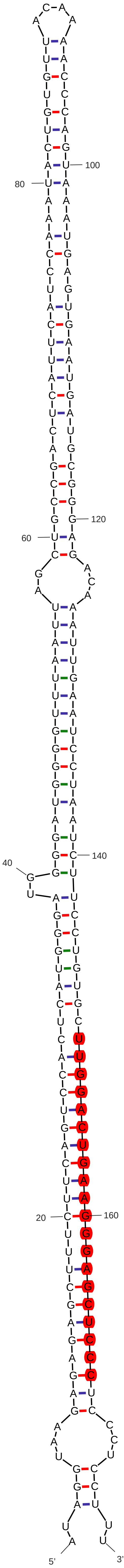

dG = -85.70 [Initially -85.70] 19-MIR159-[vvi-MIR319c MI0006549]

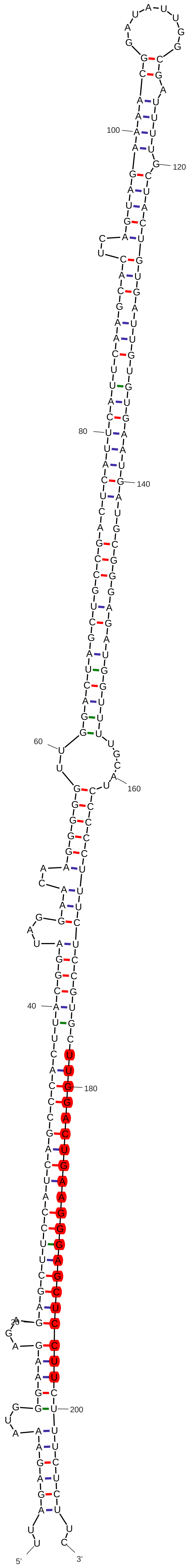

dG = -86.80 [Initially -86.80] 20-MIR159-[ptc-MIR319f MI0002301]

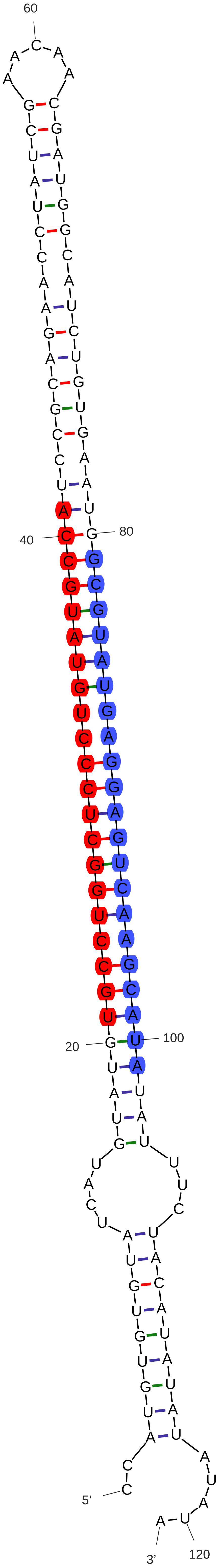

dG = -59.50 [Initially -59.50] 21-MIR160-[mdm-MIR160c MI0023006]

Output of sir_graph (©)
mfold_util 4.6

Created Thu Jan 24 20:00:40 2013

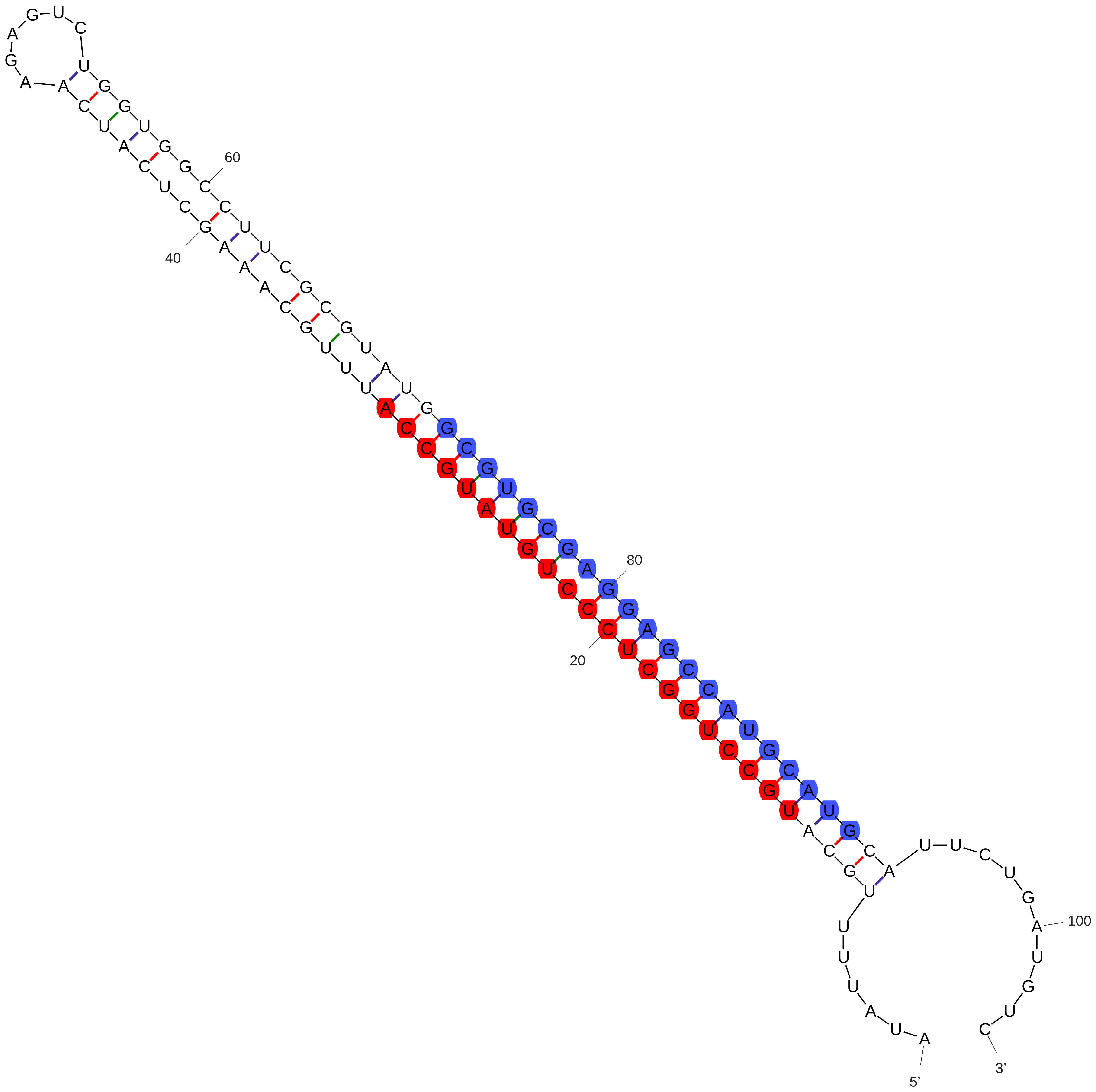

dG = -54.20 [Initially -54.20] 22-MIR160-[tcc-MIR160b MI0017461]

Output of sir_graph (©)
mfold_util 4.6

Created Thu Jan 24 20:01:25 2013

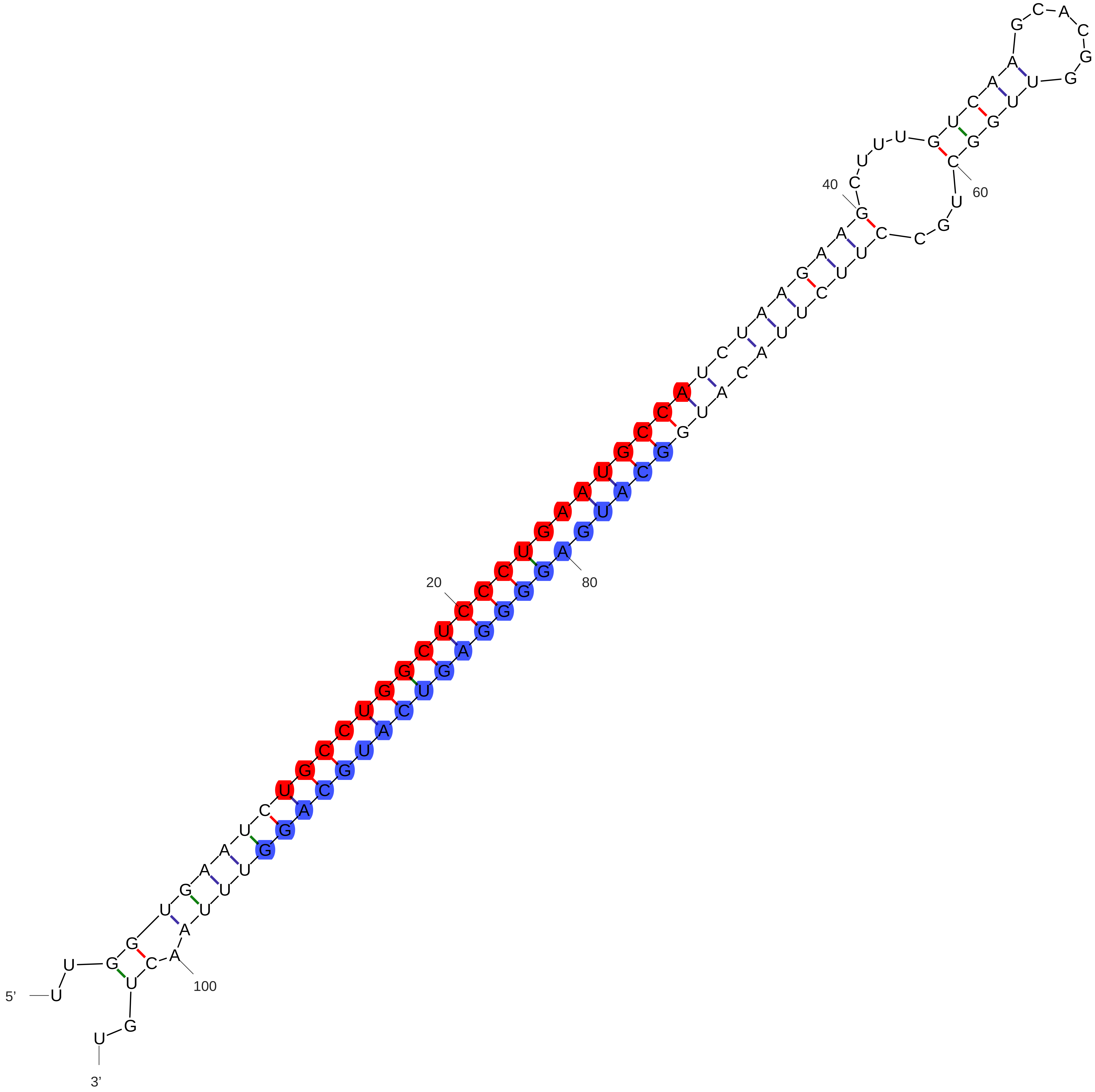

dG = -53.00 [Initially -53.00] 23-MIR160-[tcc-MIR160a MI0017460]

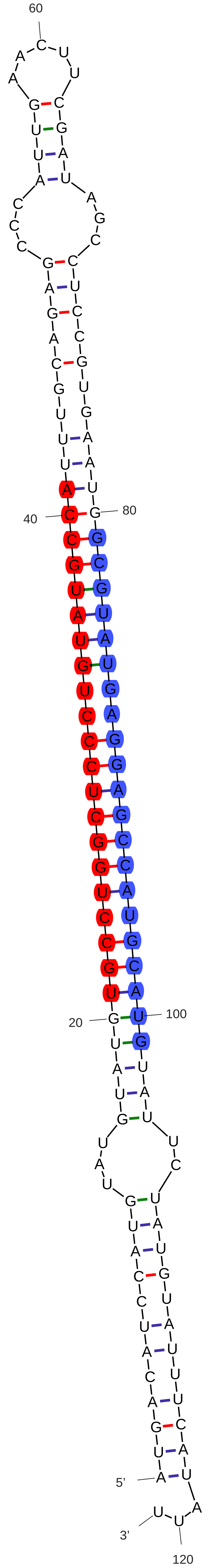

dG = -53.00 [Initially -53.00] 24-MIR160-[mdm-MIR160e MI0023008]

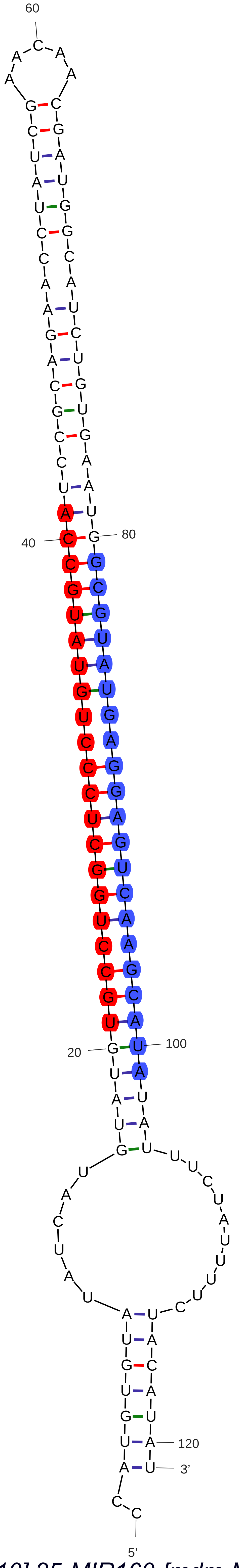

dG = -54.10 [Initially -54.10] 25-MIR160-[mdm-MIR160c MI0023006]

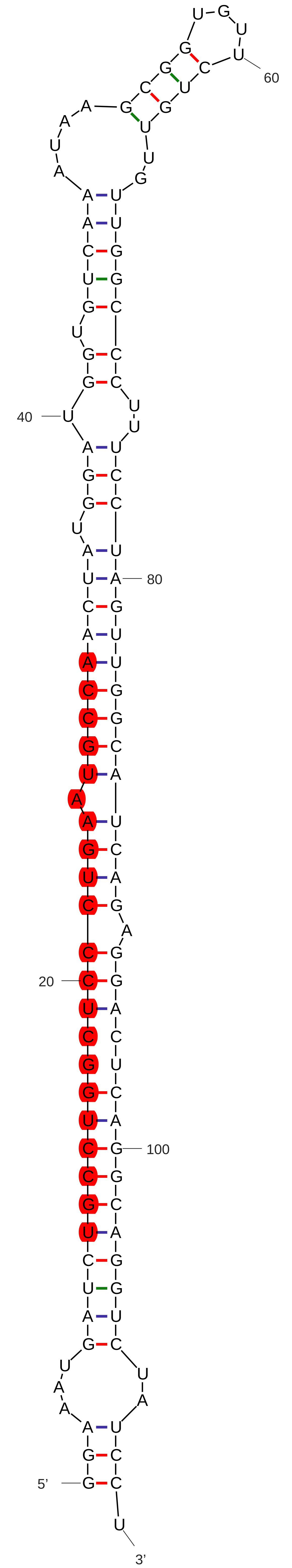

dG = -61.80 [Initially -61.80] 26-MIR160

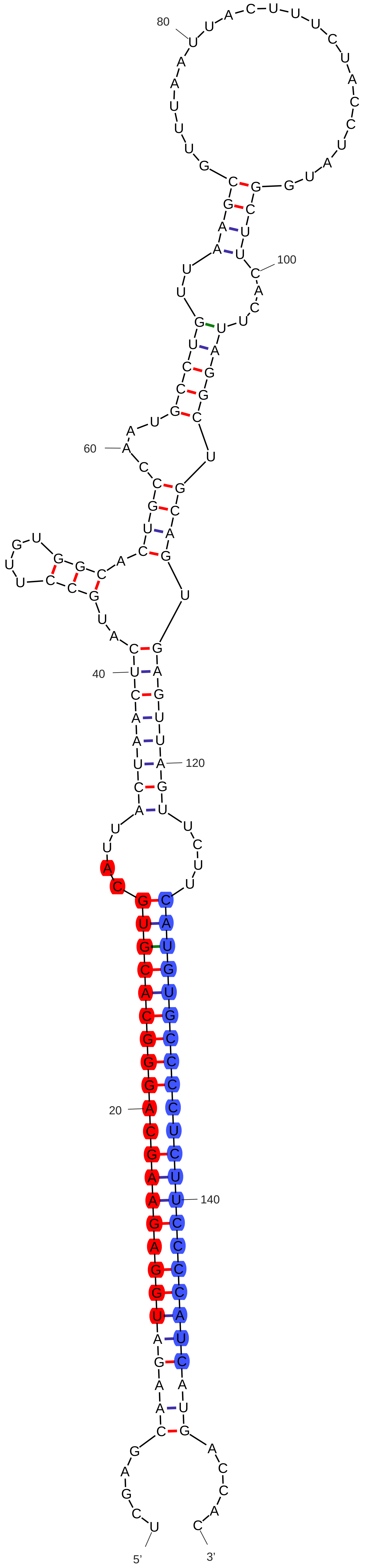

dG = -57.10 [Initially -58.90] 28-MIR164

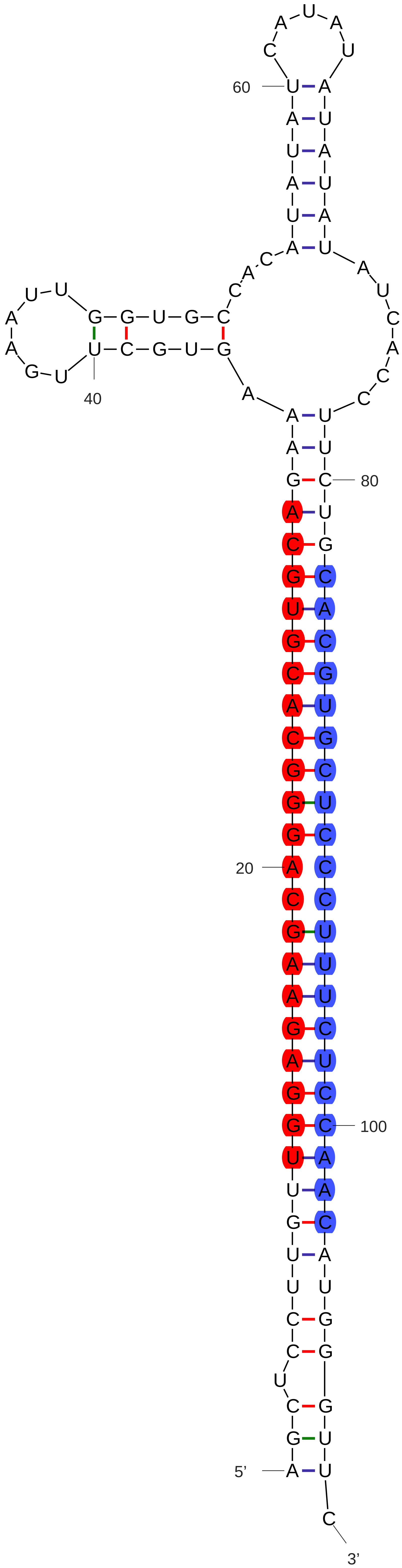

dG = -48.54 [Initially -50.50] 29-MIR164

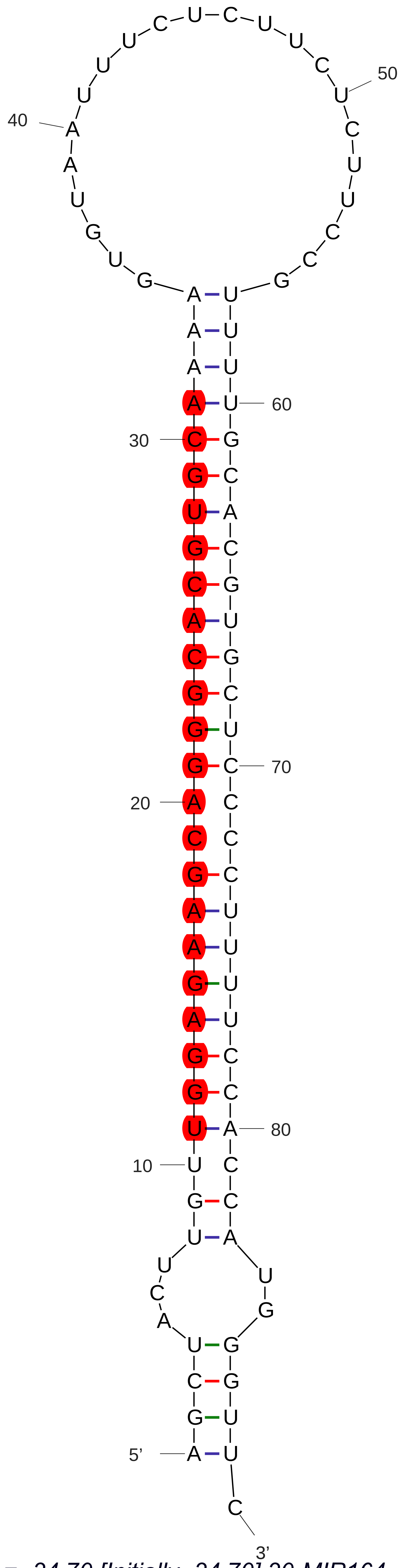

dG = -34.70 [Initially -34.70] 30-MIR164

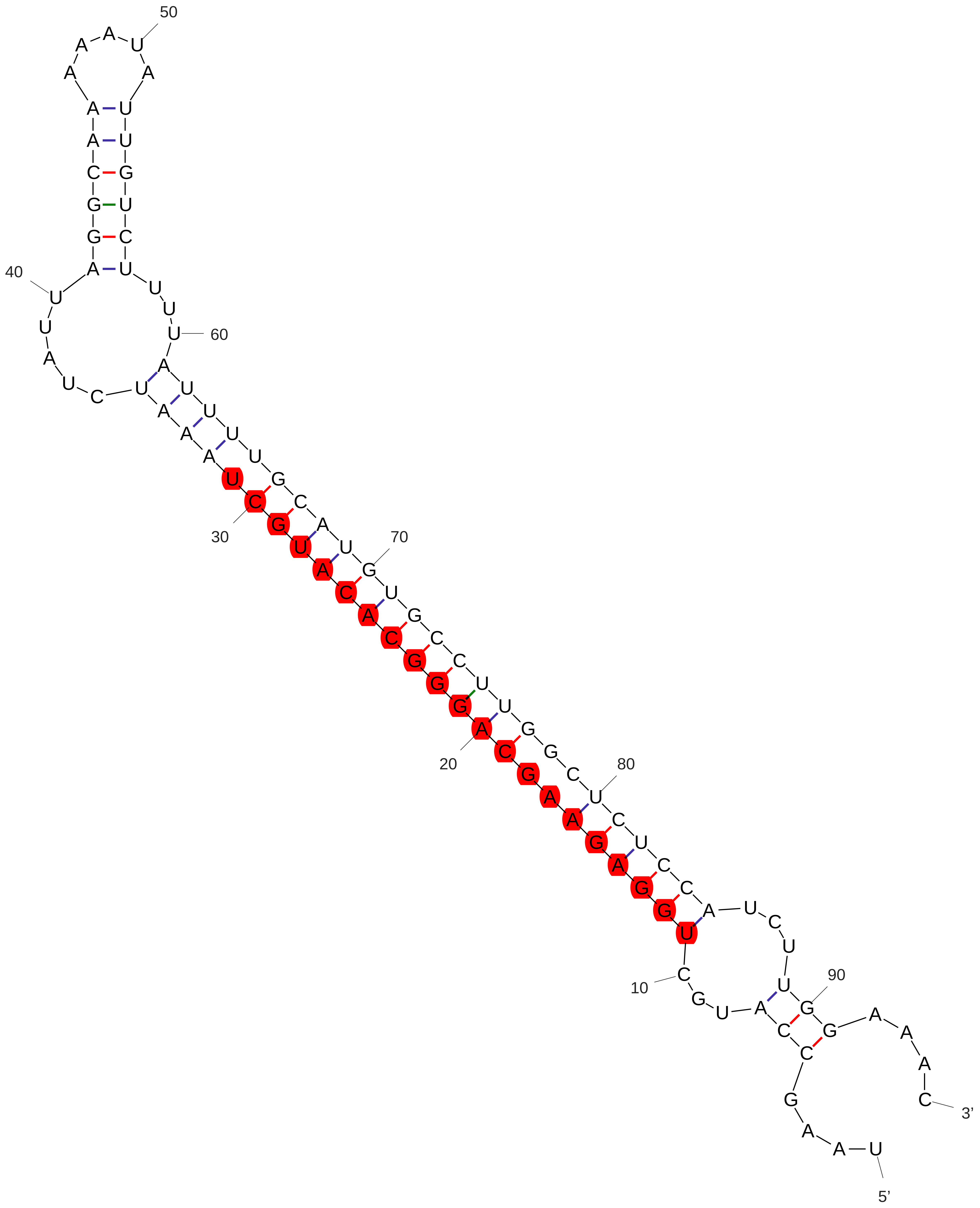

dG = -40.80 [Initially -40.80] 31-MIR164

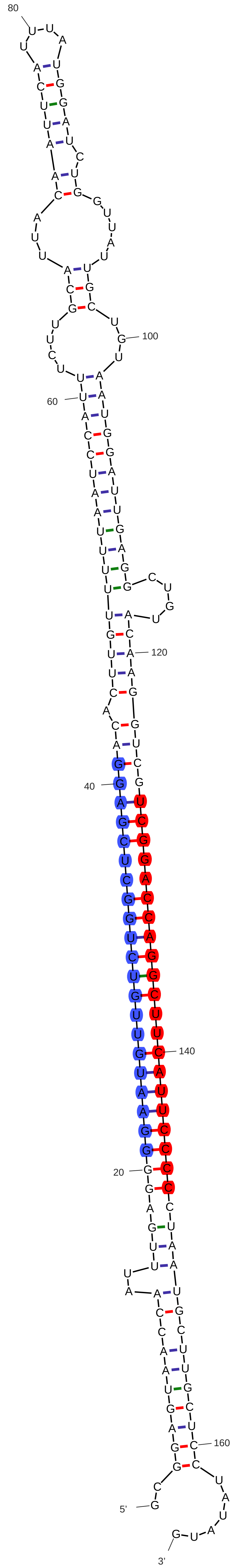

dG = -69.50 [Initially -69.50] 32-MIR166

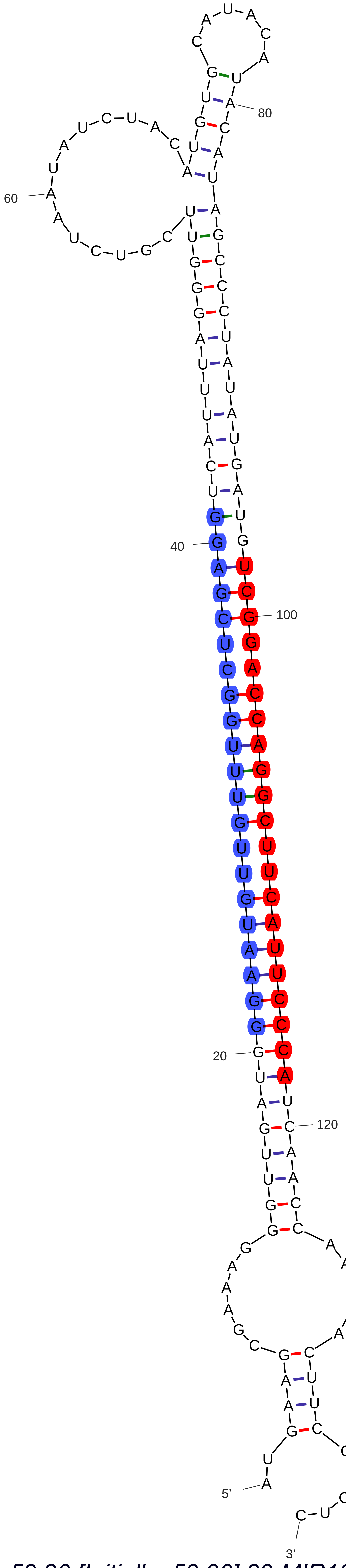

dG = -59.90 [Initially -59.90] 33-MIR166

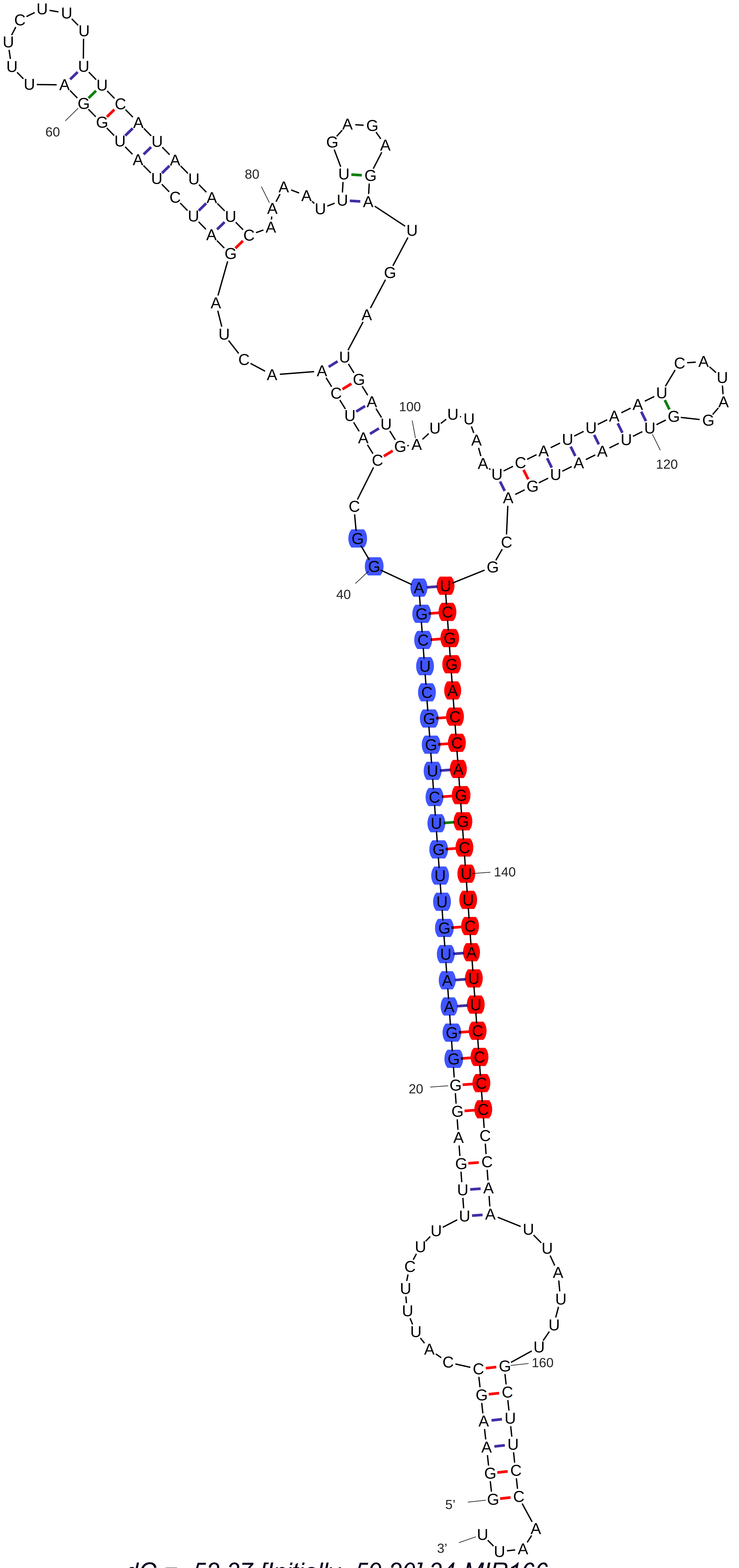

dG = -52.37 [Initially -59.20] 34-MIR166

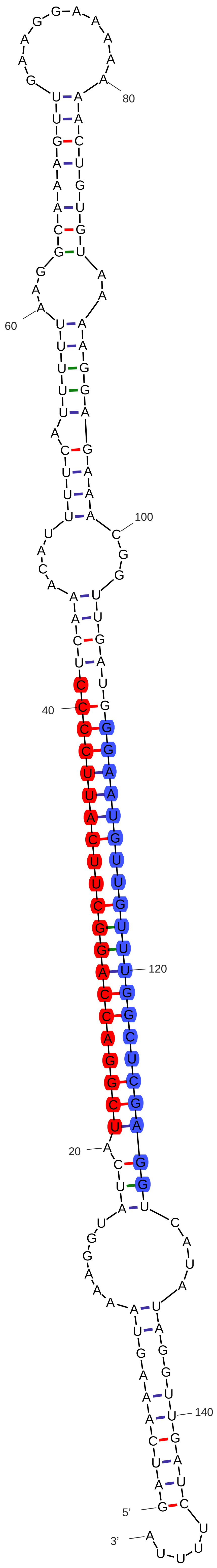

dG = -42.20 [Initially -42.20] 35-MIR166

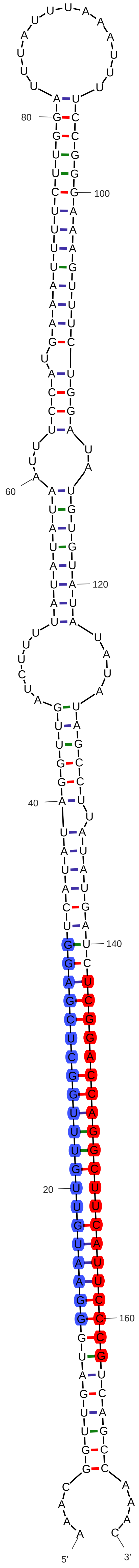

dG = -85.30 [Initially -85.30] 36-MIR166-[tcc-MIR166b MI0017468]

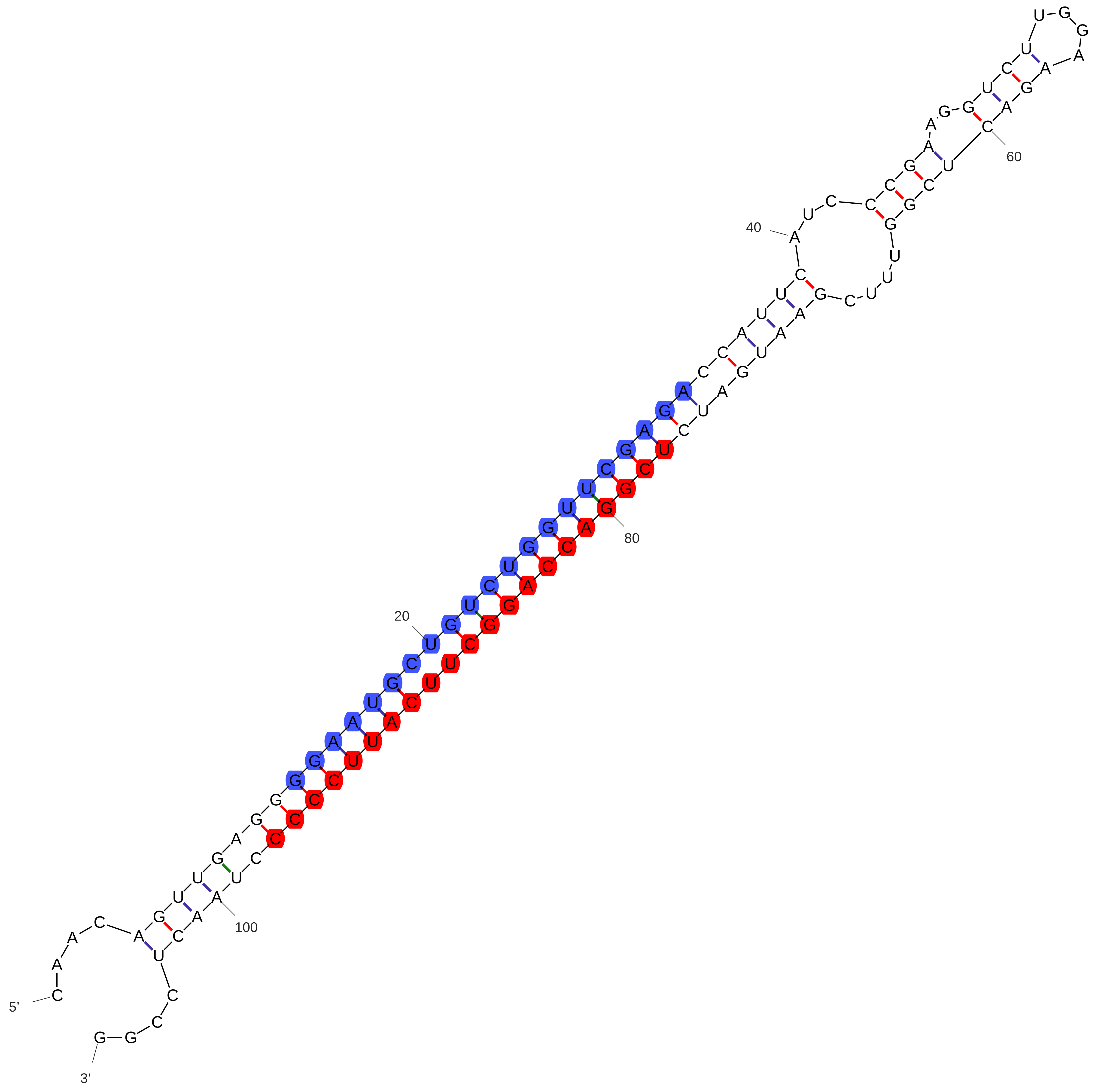

dG = -55.80 [Initially -55.80] 37-MIR166

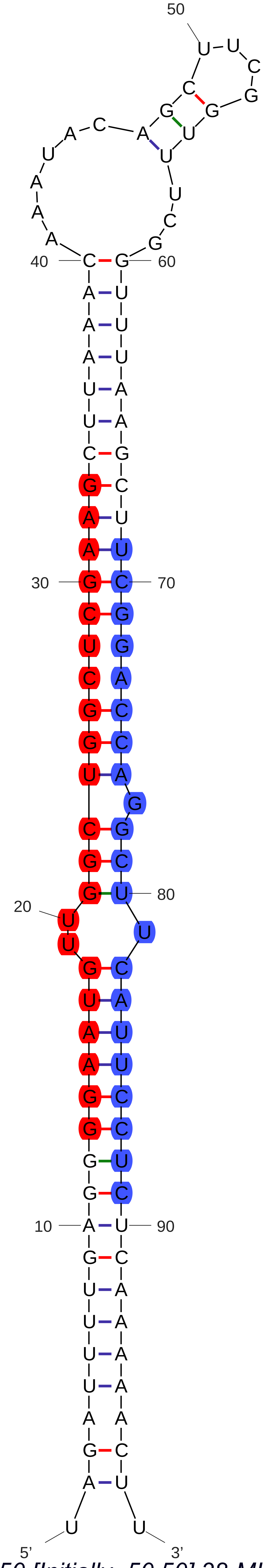

dG = -50.50 [Initially -50.50] 38-MIR166

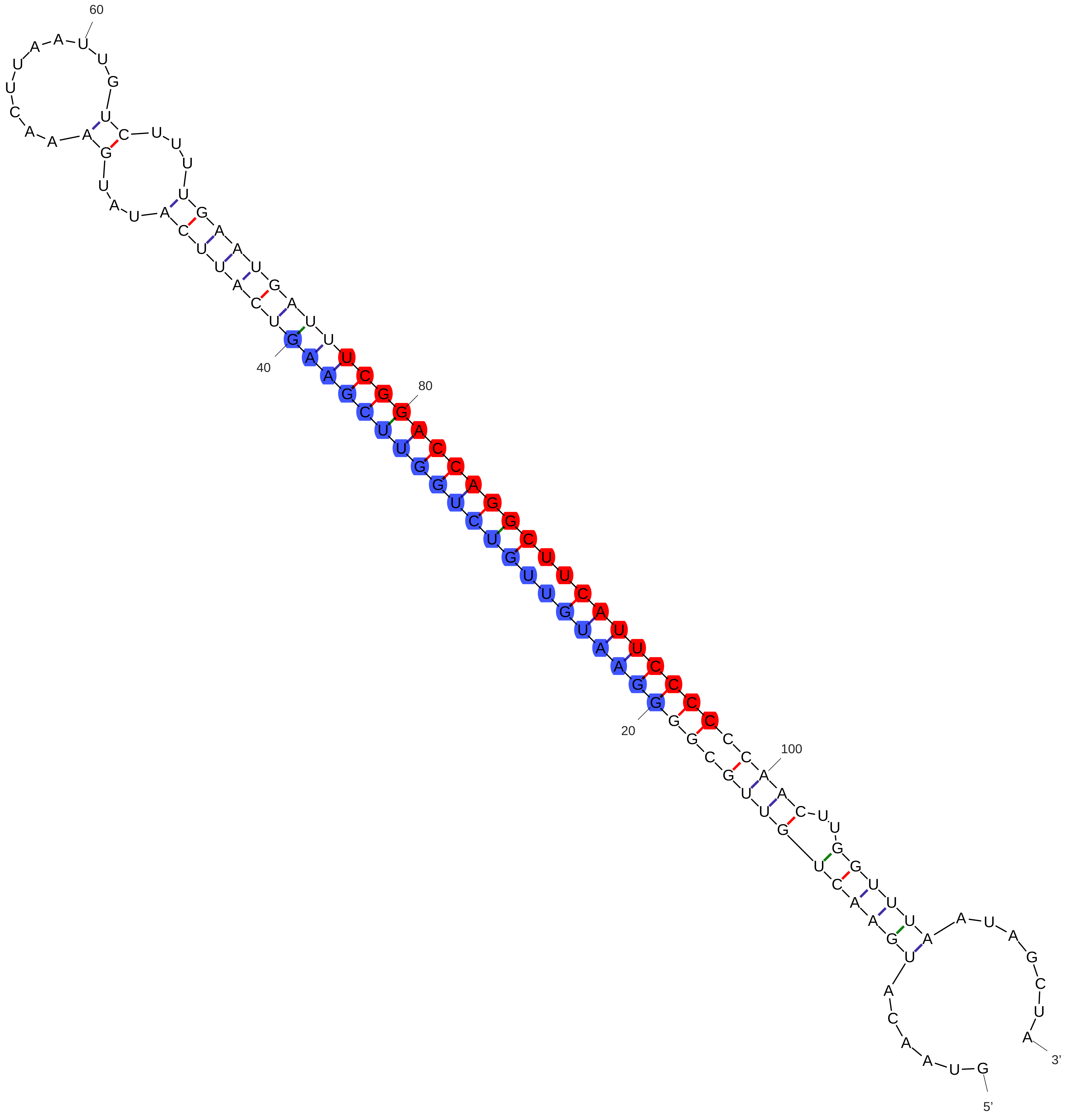

dG = -55.40 [Initially -55.40] 39-MIR166-[tcc-MIR166a MI0017467]

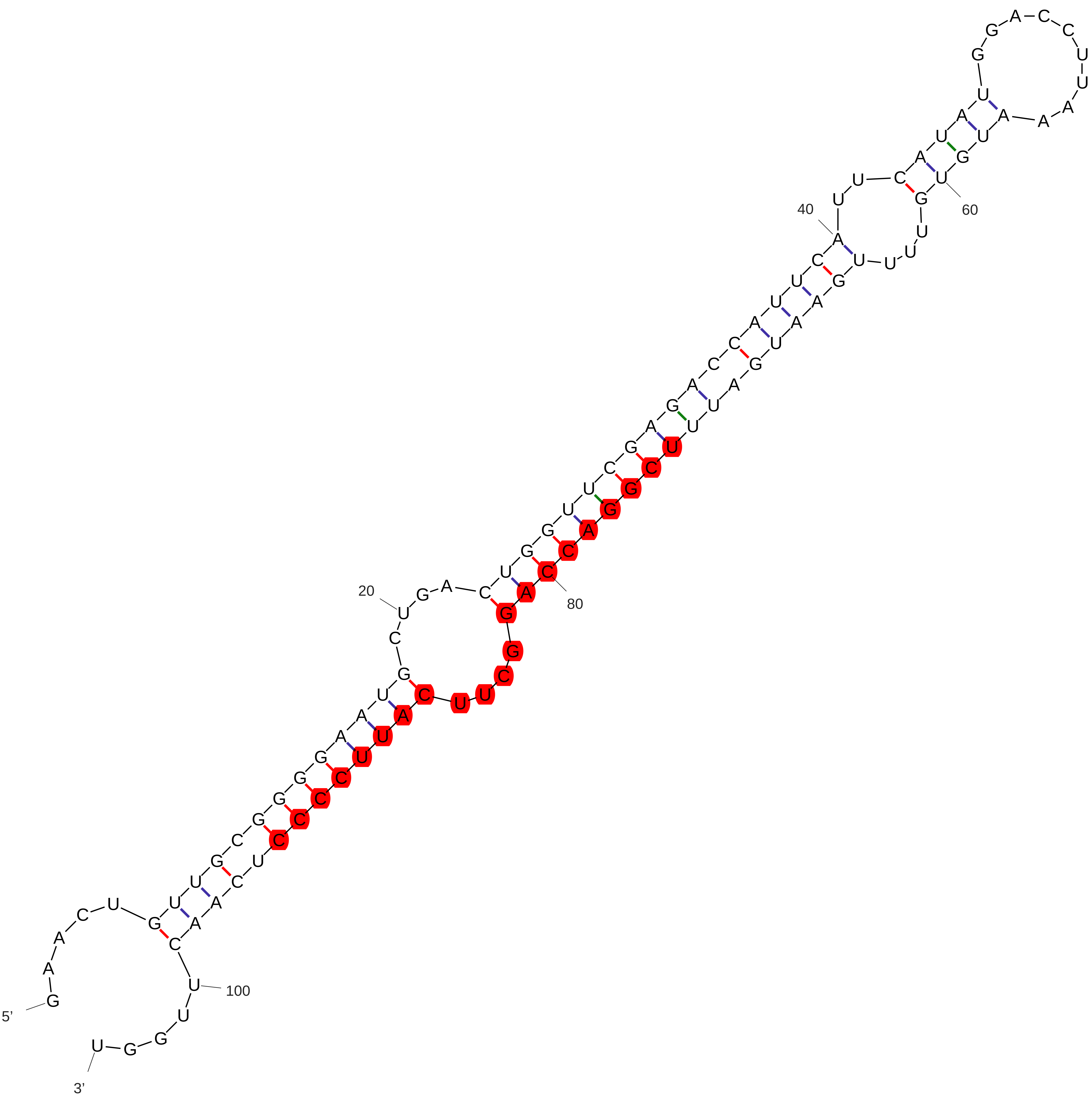

dG = -47.30 [Initially -47.30] 40-MIR166

Output of sir_graph (©)
mfold_util 4.6

Created Thu Jan 24 20:29:30 2013

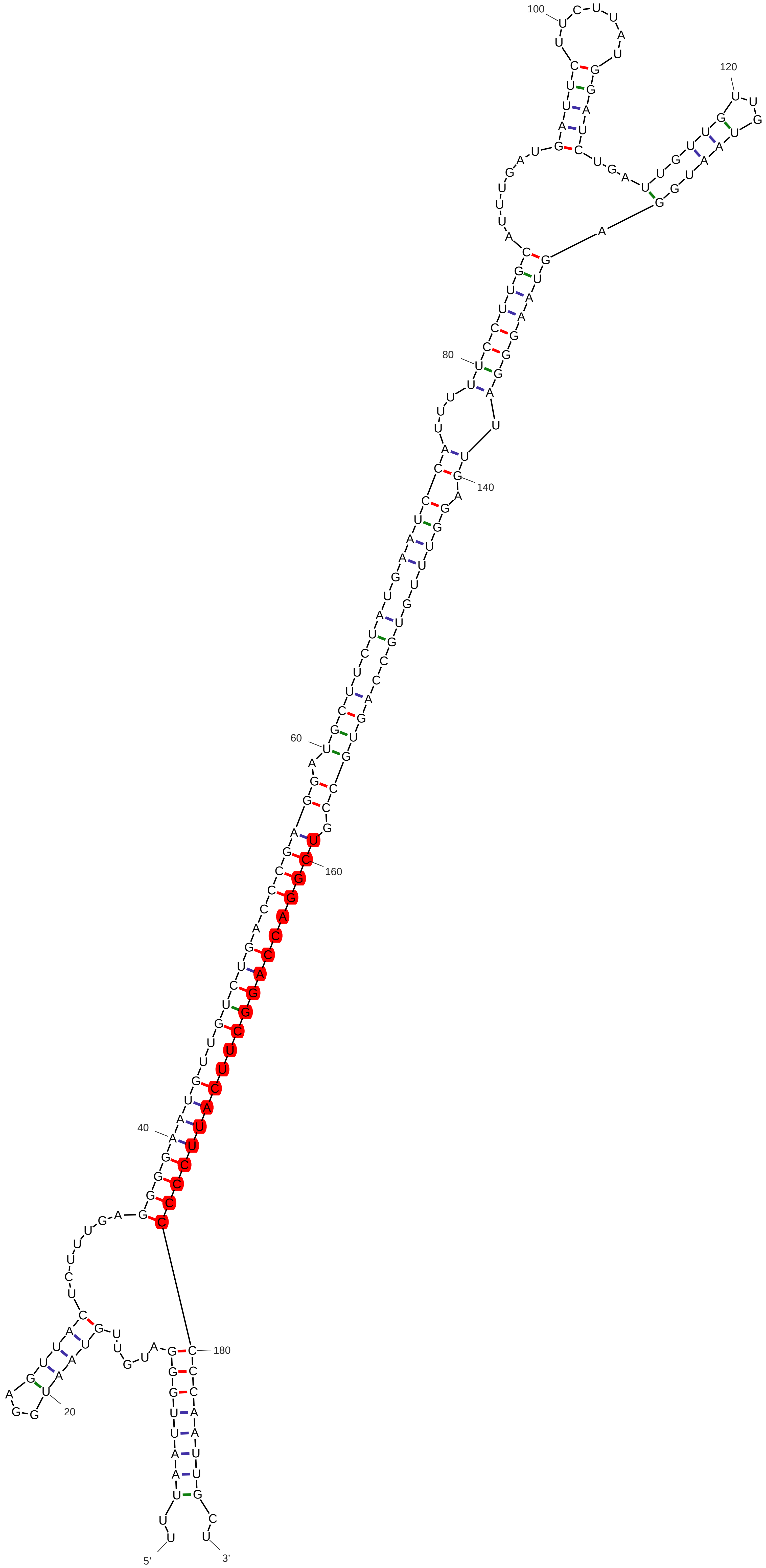

dG = -55.77 [Initially -60.50] 41-MIR166

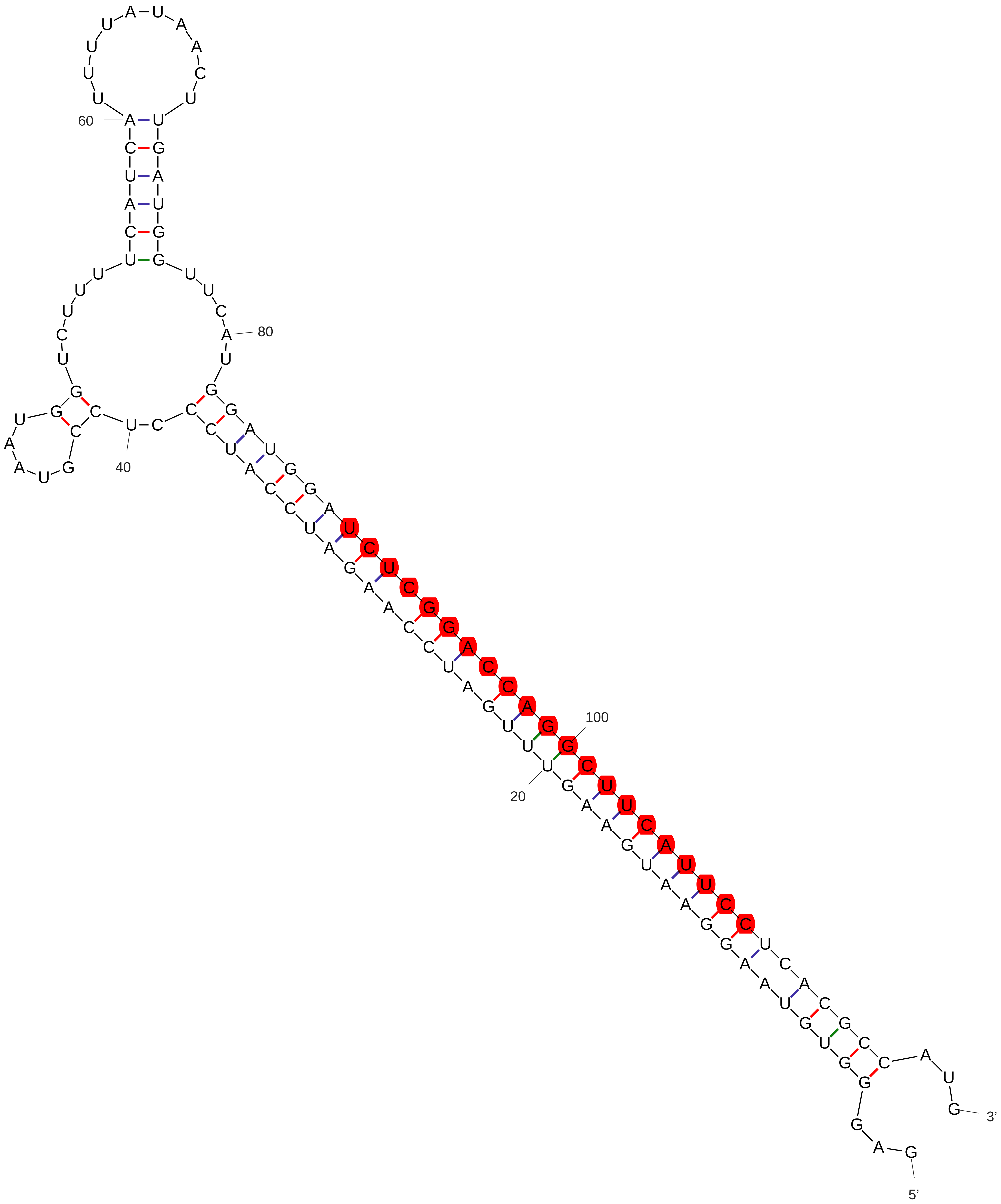

dG = -52.34 [Initially -55.90] 43-MIR166

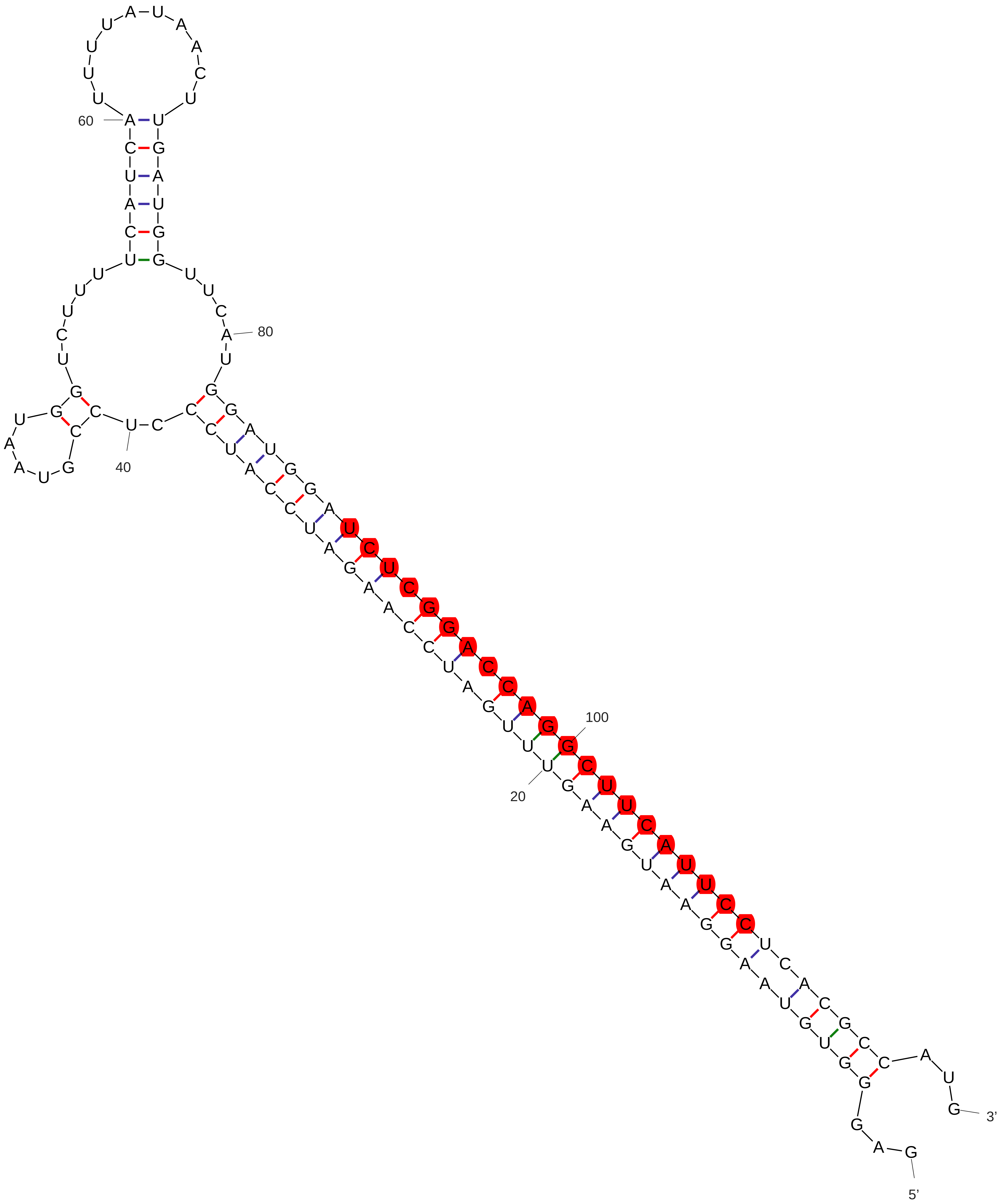

dG = -52.34 [Initially -55.90] 44-MIR166

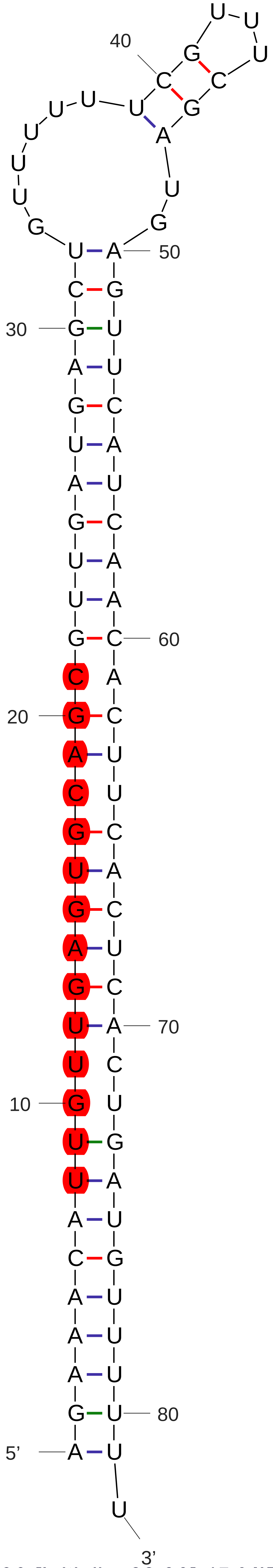

dG = -32.90 [Initially -32.90] 45-MIR397

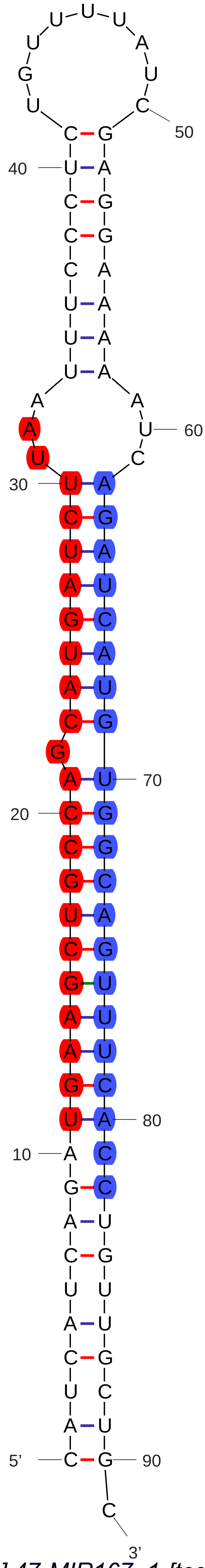

dG = -38.90 [Initially -38.90] 47-MIR167_1-[tcc-MIR167c MI0017473]

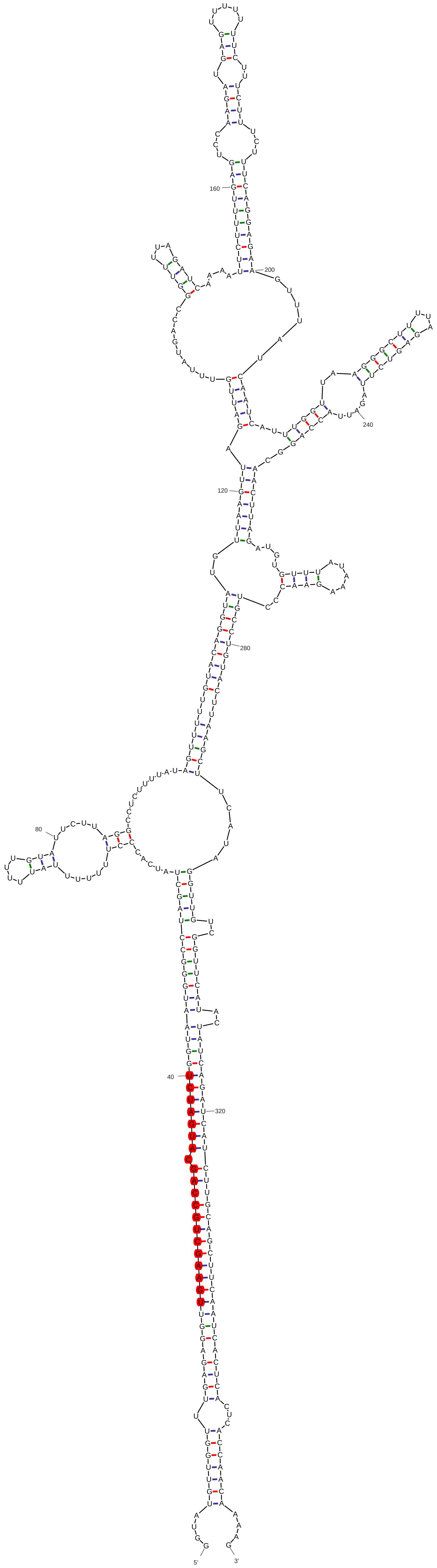

dG = -96.79 [Initially -110.00] 49-MIR167_1

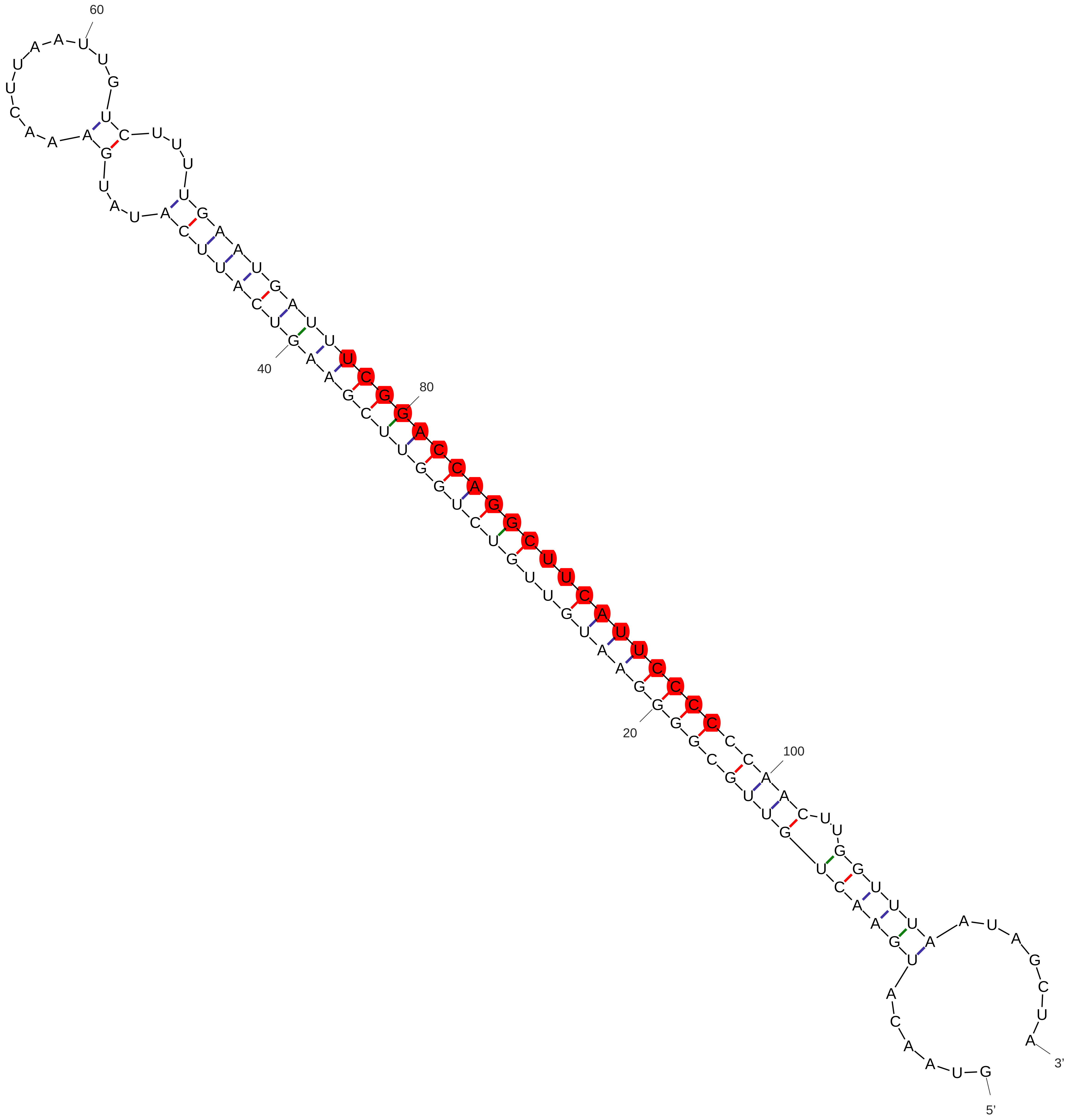

dG = -55.40 [Initially -55.40] 50-MIR167_1

Output of sir_graph (©)
mfold_util 4.6

Created Thu Jan 24 20:37:28 2013

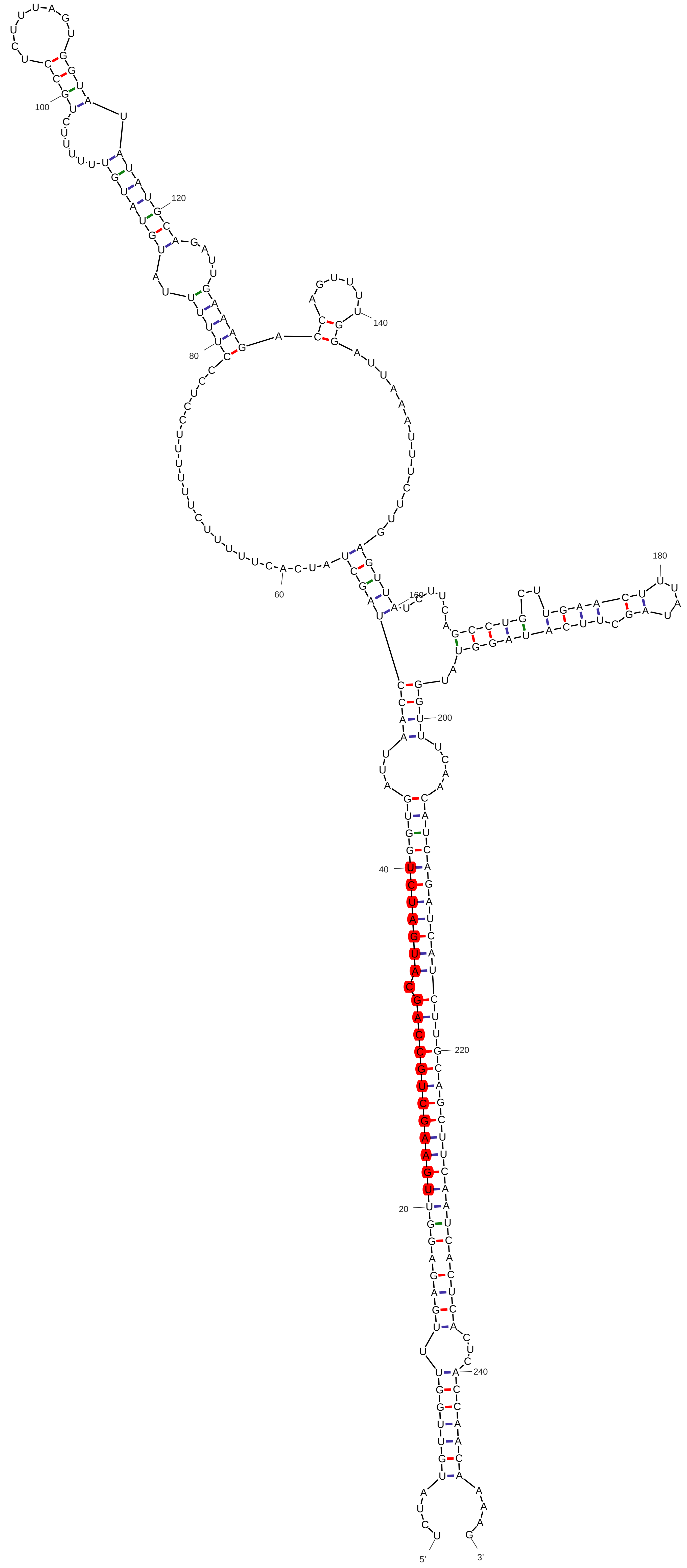

dG = -69.31 [Initially -75.10] 51-MIR167_1

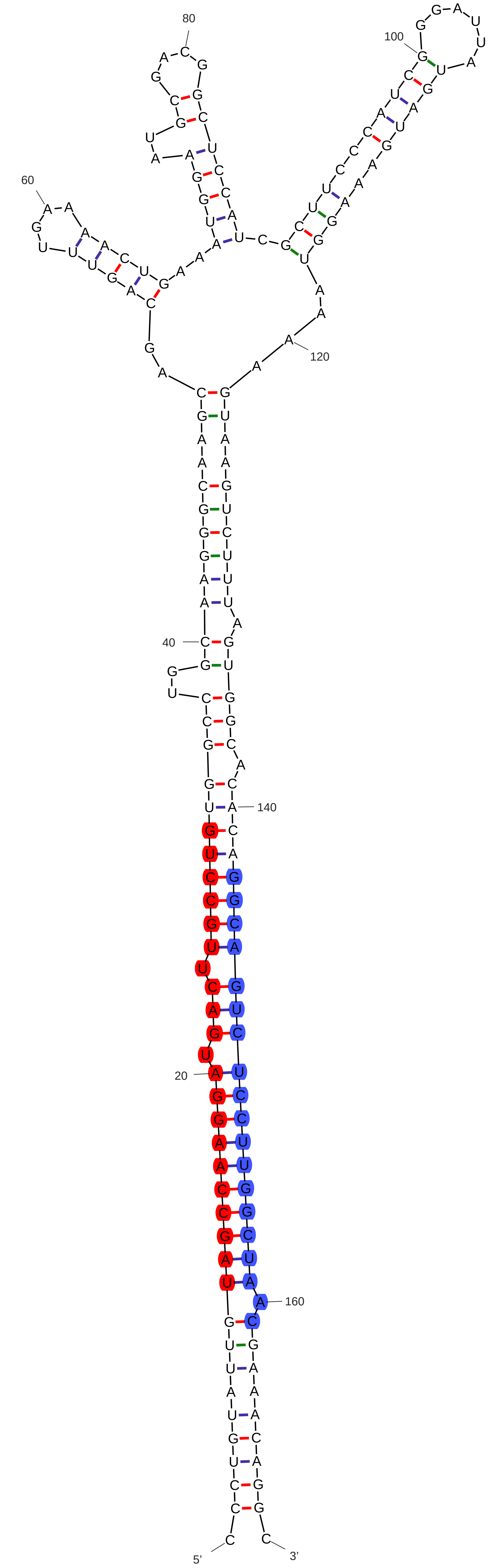

dG = -71.95 [Initially -72.80] 52-MIR169_1

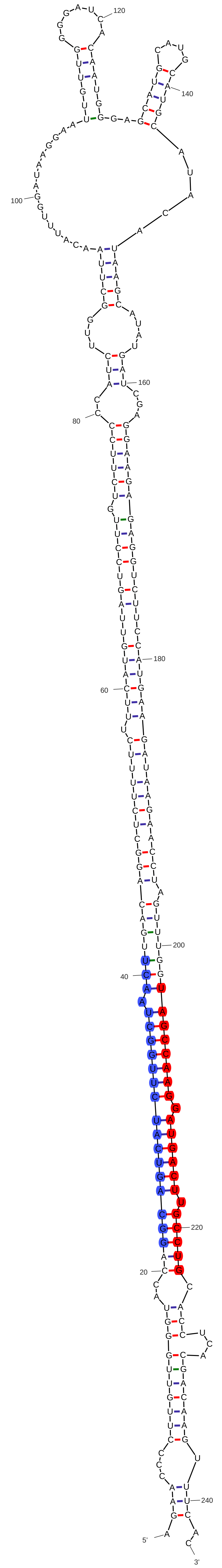

dG = -88.22 [Initially -92.80] 53_MIR169_1

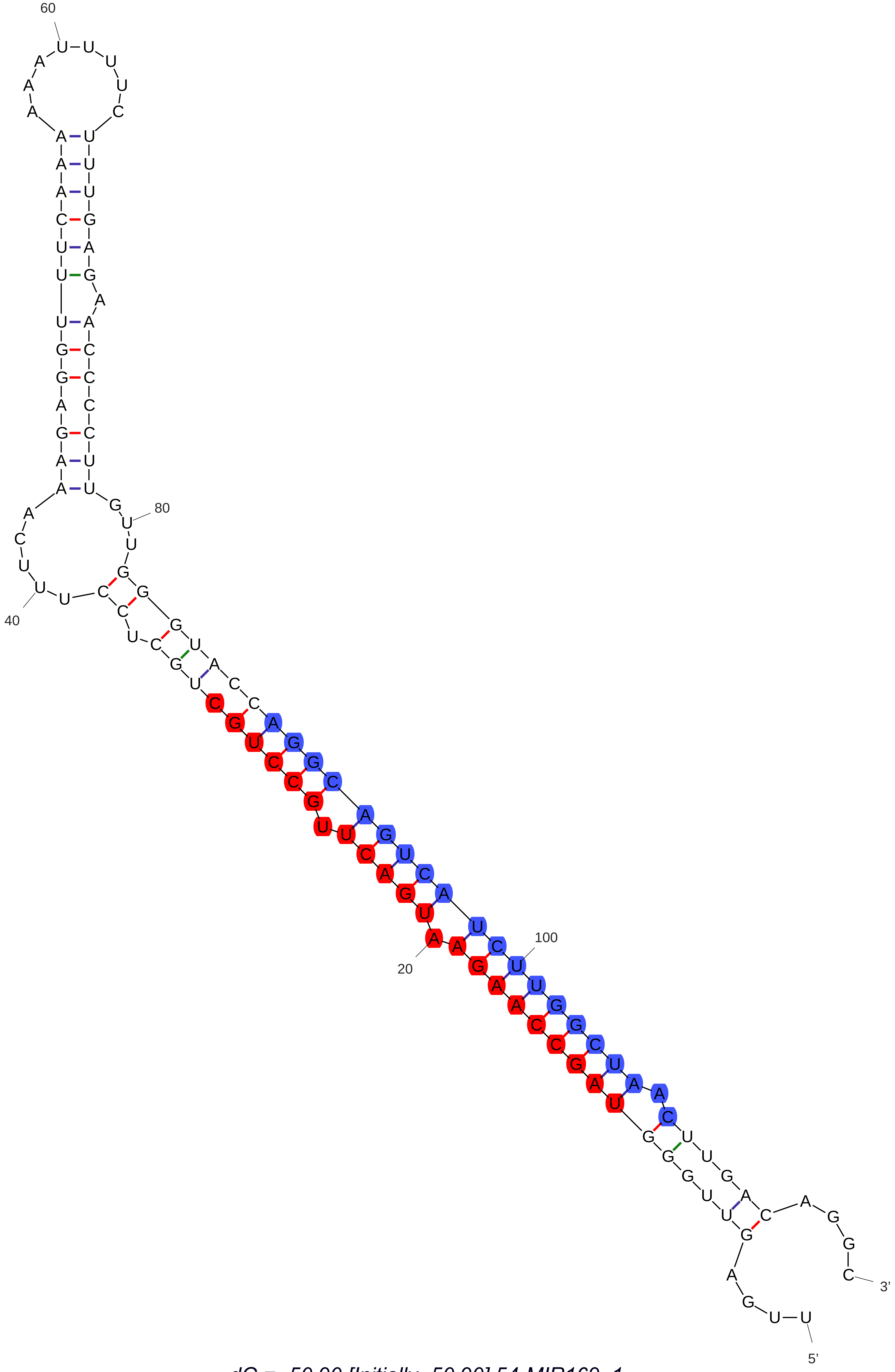

dG = -50.90 [Initially -50.90] 54-MIR169_1

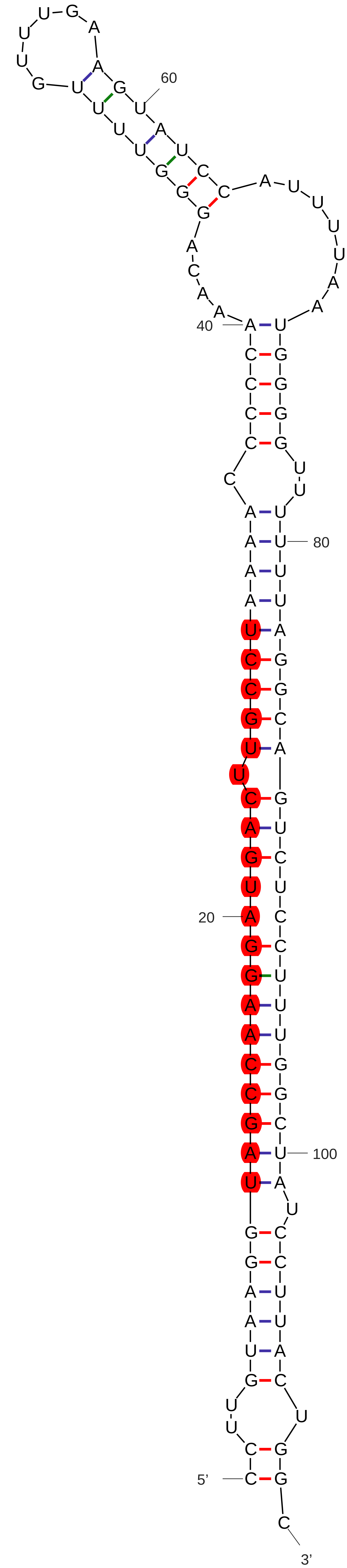

dG = -49.30 [Initially -49.30] 55-MIR169_1

Output of sir_graph (©)
mfold_util 4.6

Created Thu Jan 24 20:40:56 2013

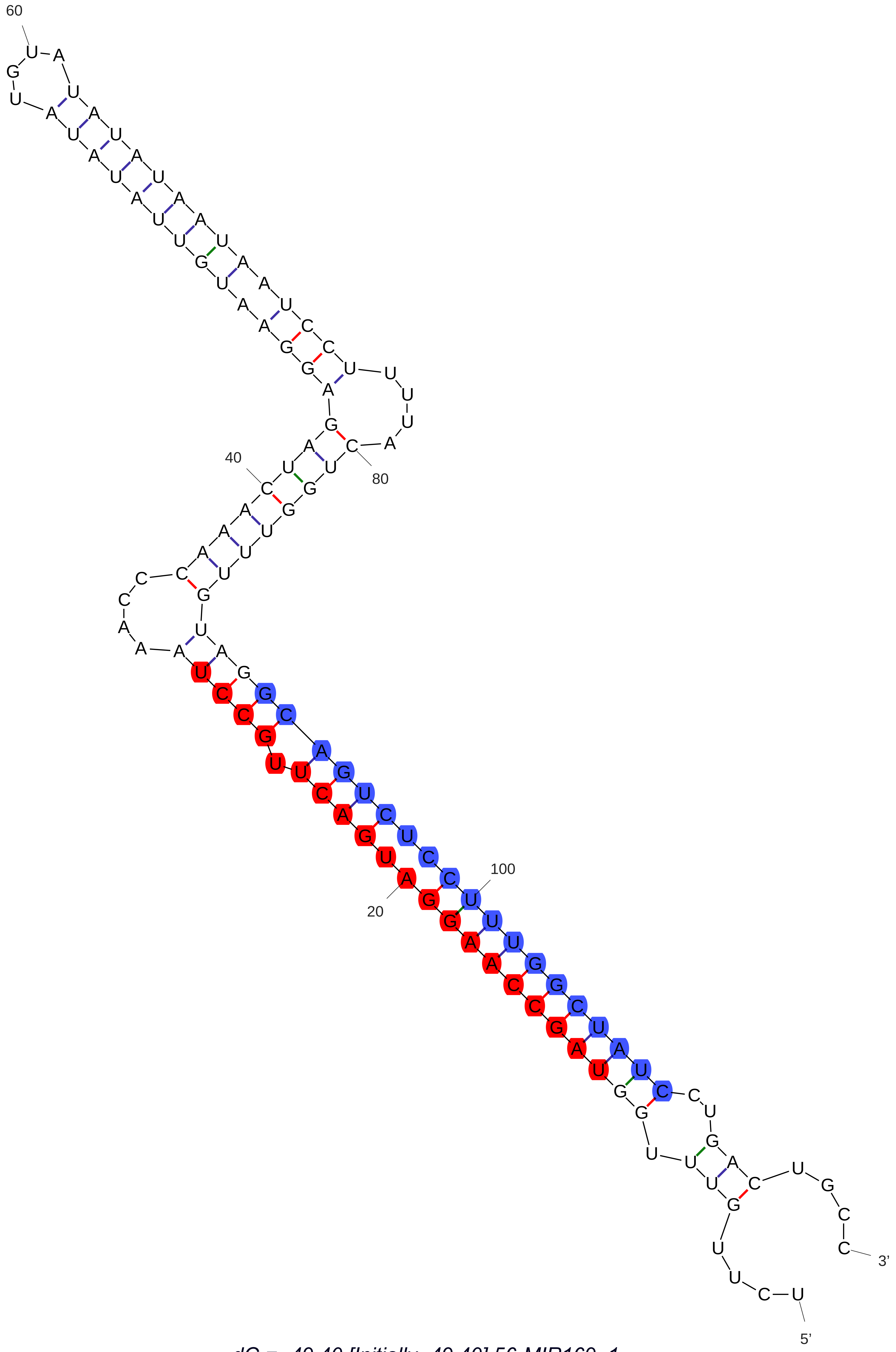

dG = -49.40 [Initially -49.40] 56-MIR169_1

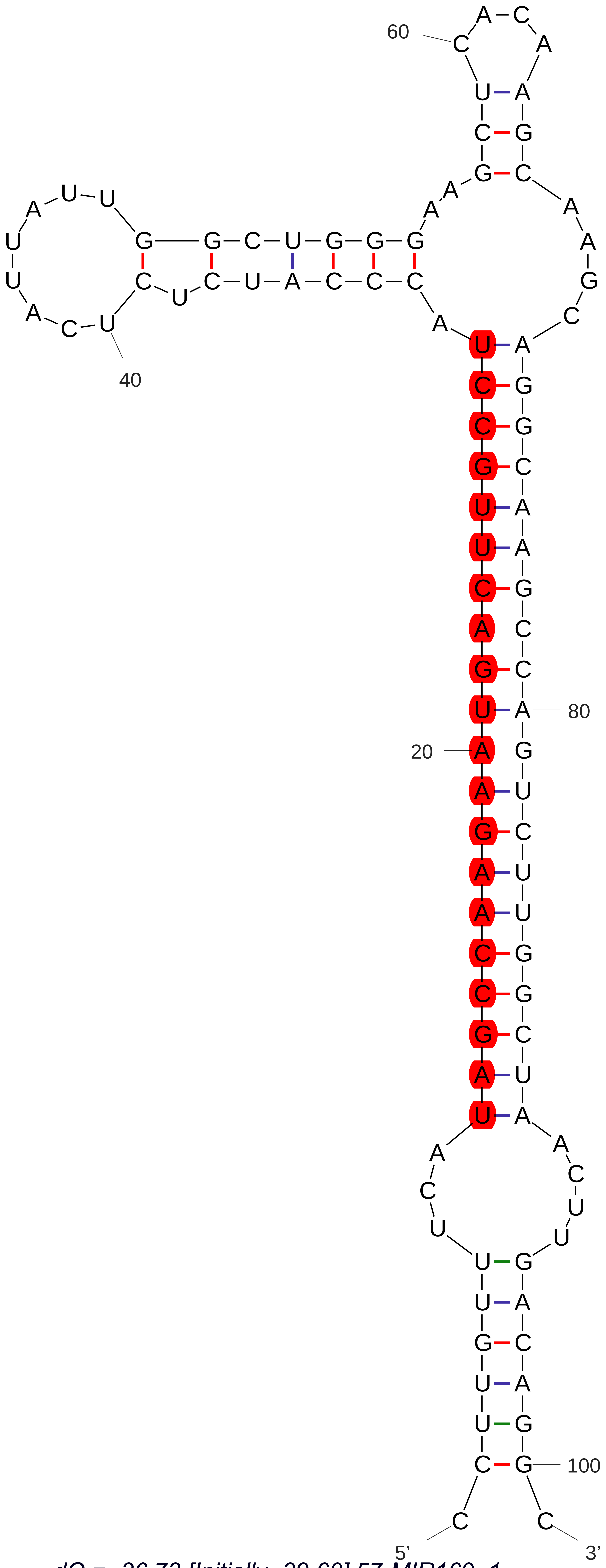

dG = -36.73 [Initially -39.60] 57-MIR169_1

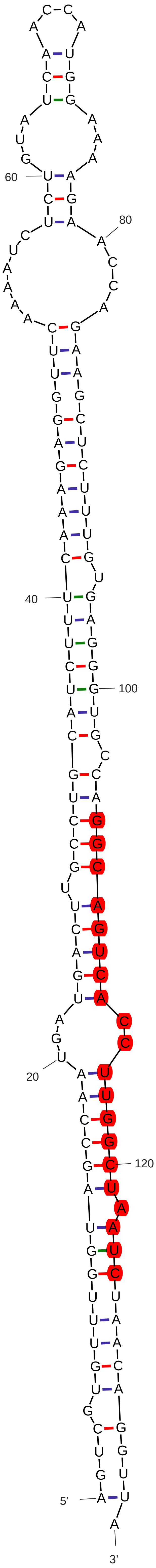

dG = -48.60 [Initially -48.60] 58-MIR169_1

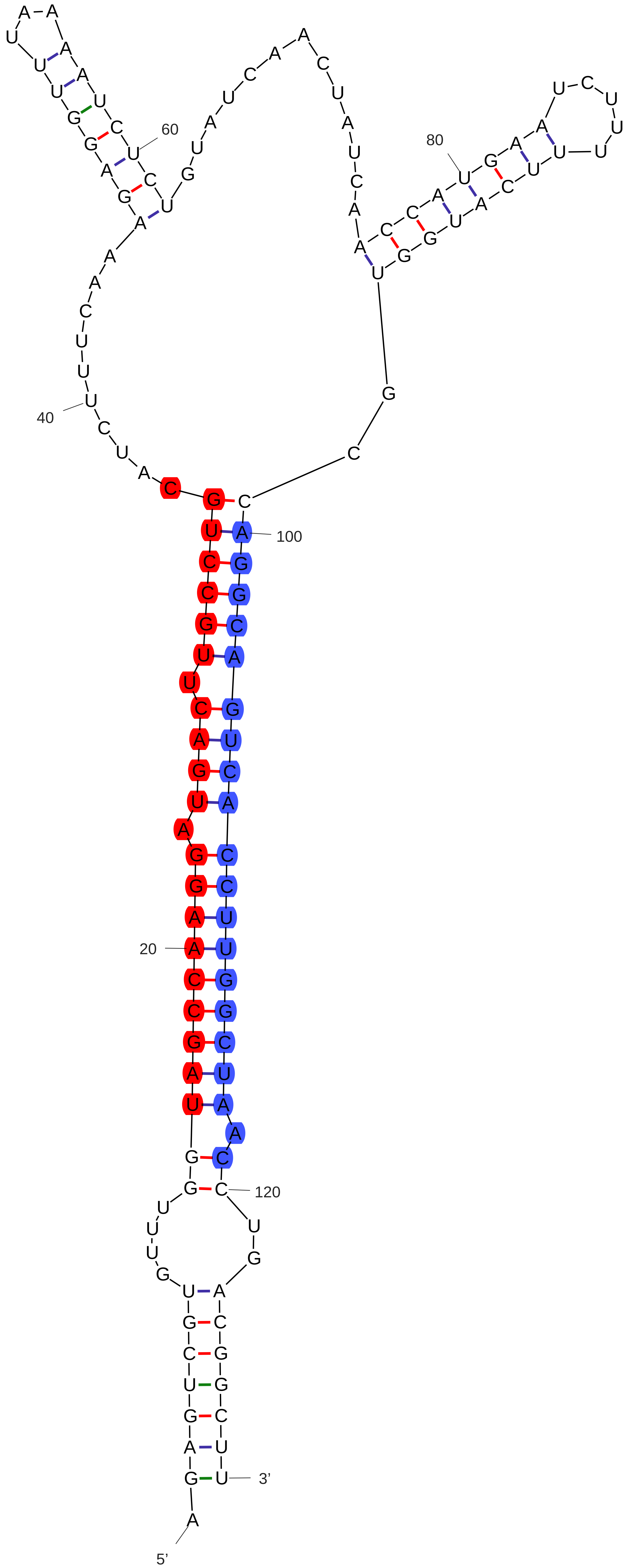

dG = -52.13 [Initially -56.30] 59-MIR169_1

Output of sir_graph (©)
mfold_util 4.6

Created Thu Jan 24 20:19:03 2013

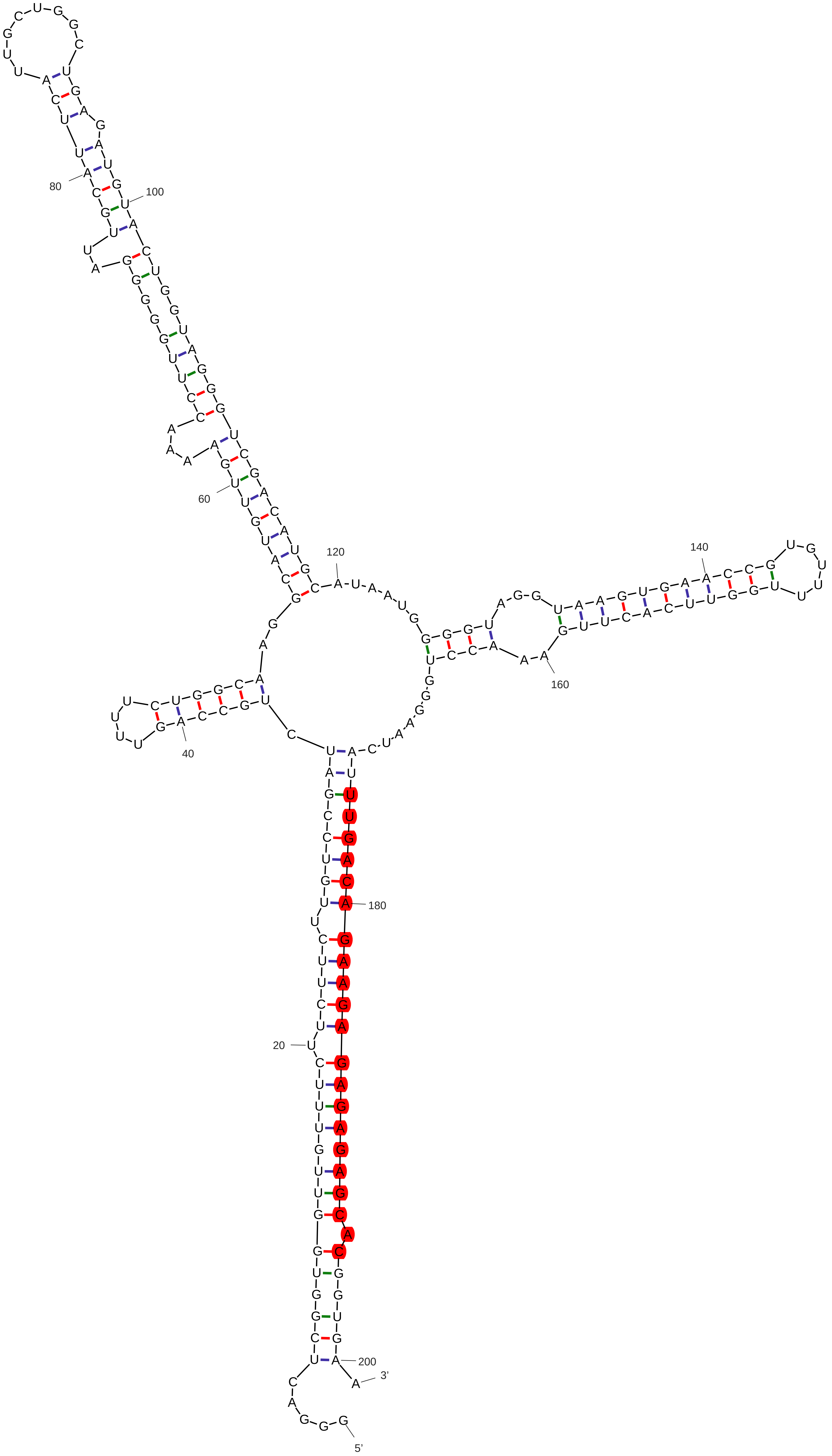

dG = -69.62 [Initially -70.80] 5-MIR156

Output of sir_graph (©)
mfold_util 4.6

Created Thu Jan 24 20:44:00 2013

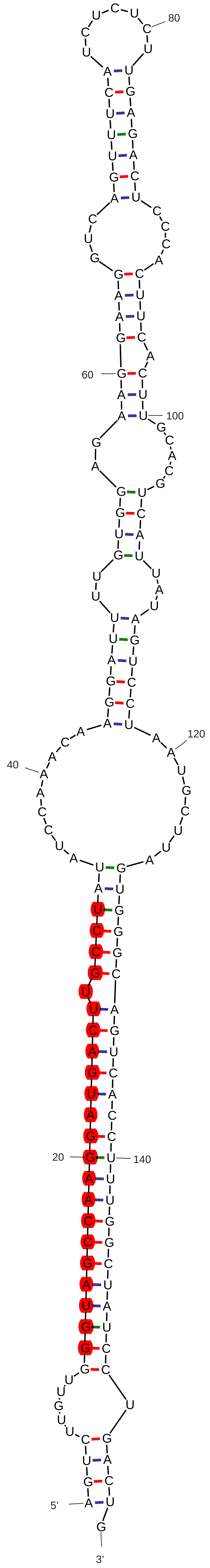

dG = -57.10 [Initially -57.10] 62-MIR169_2

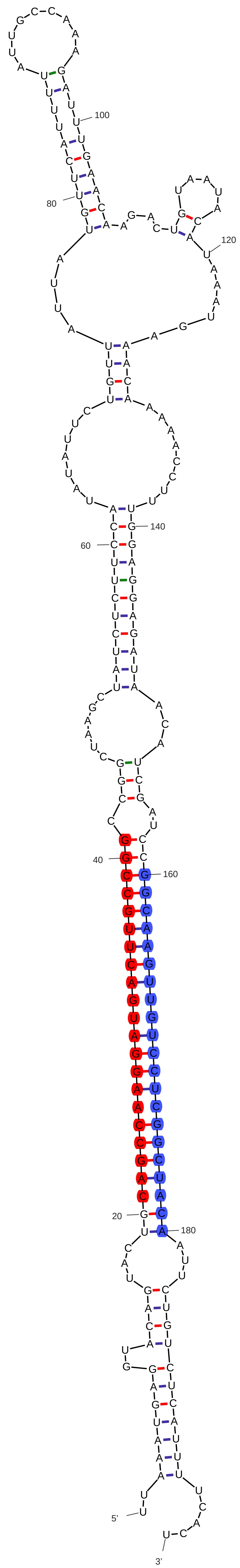

dG = -62.39 [Initially -65.90] 63-MIR169_2

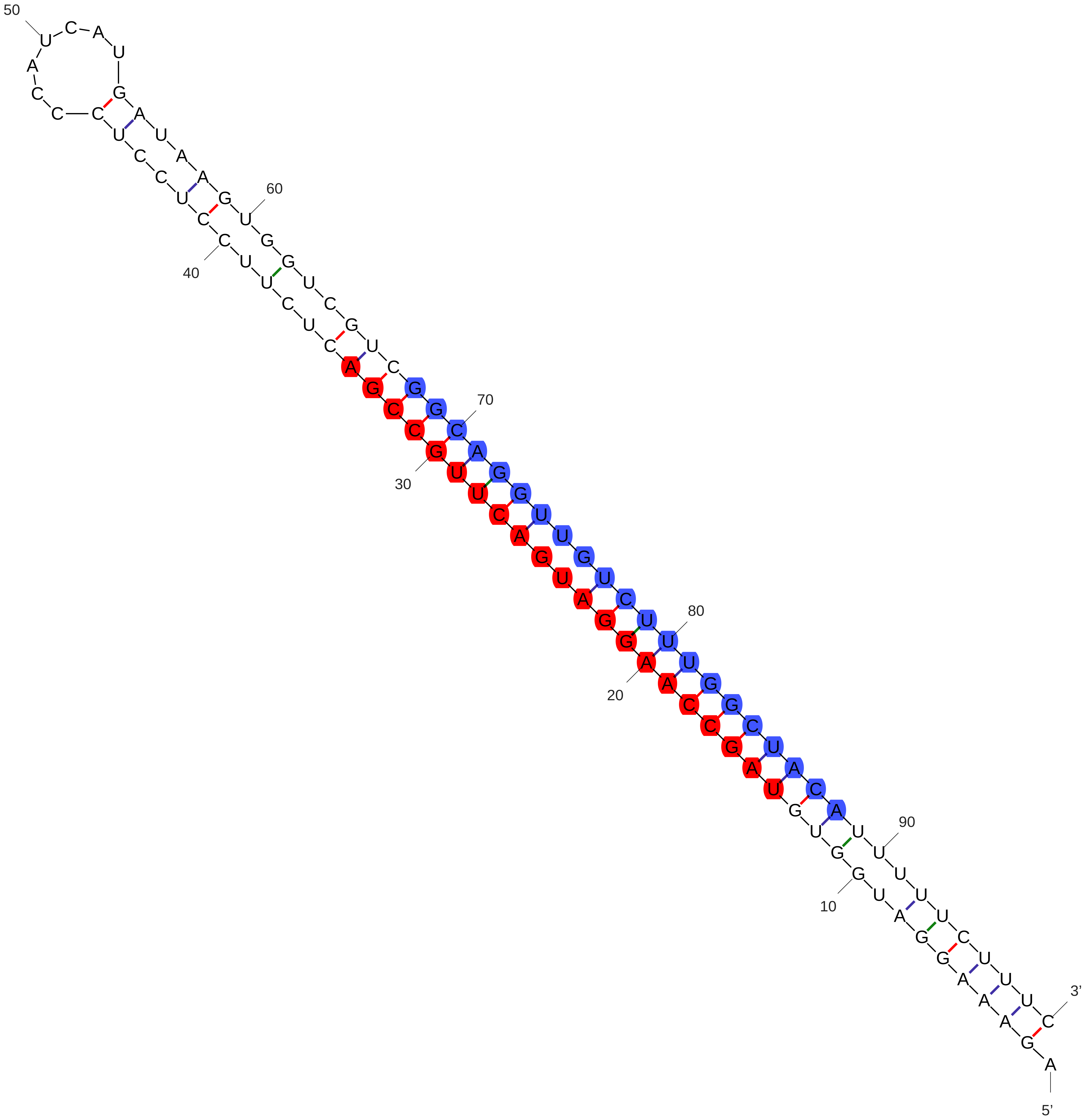

dG = -52.20 [Initially -52.20] 64-MIR169_2

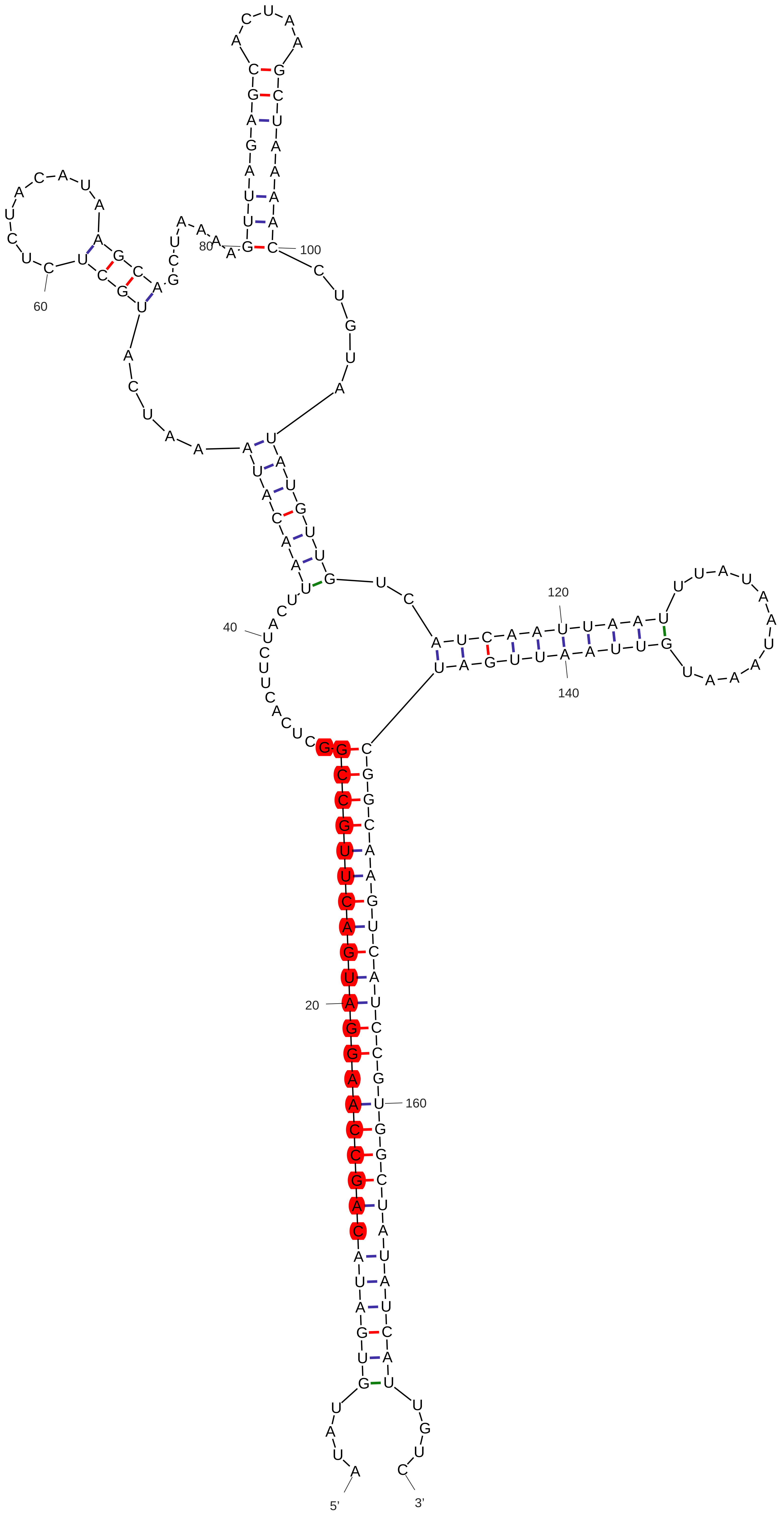

dG = -50.34 [Initially -57.40] 65-MIR169_2

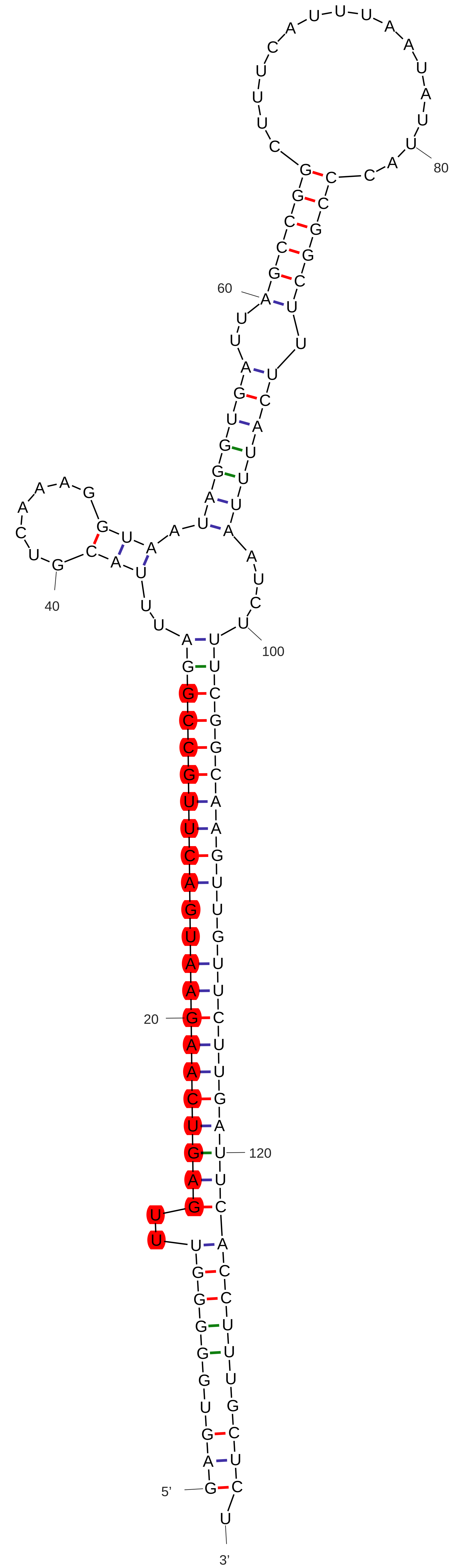

dG = -58.83 [Initially -60.70] 66-MIR169_2

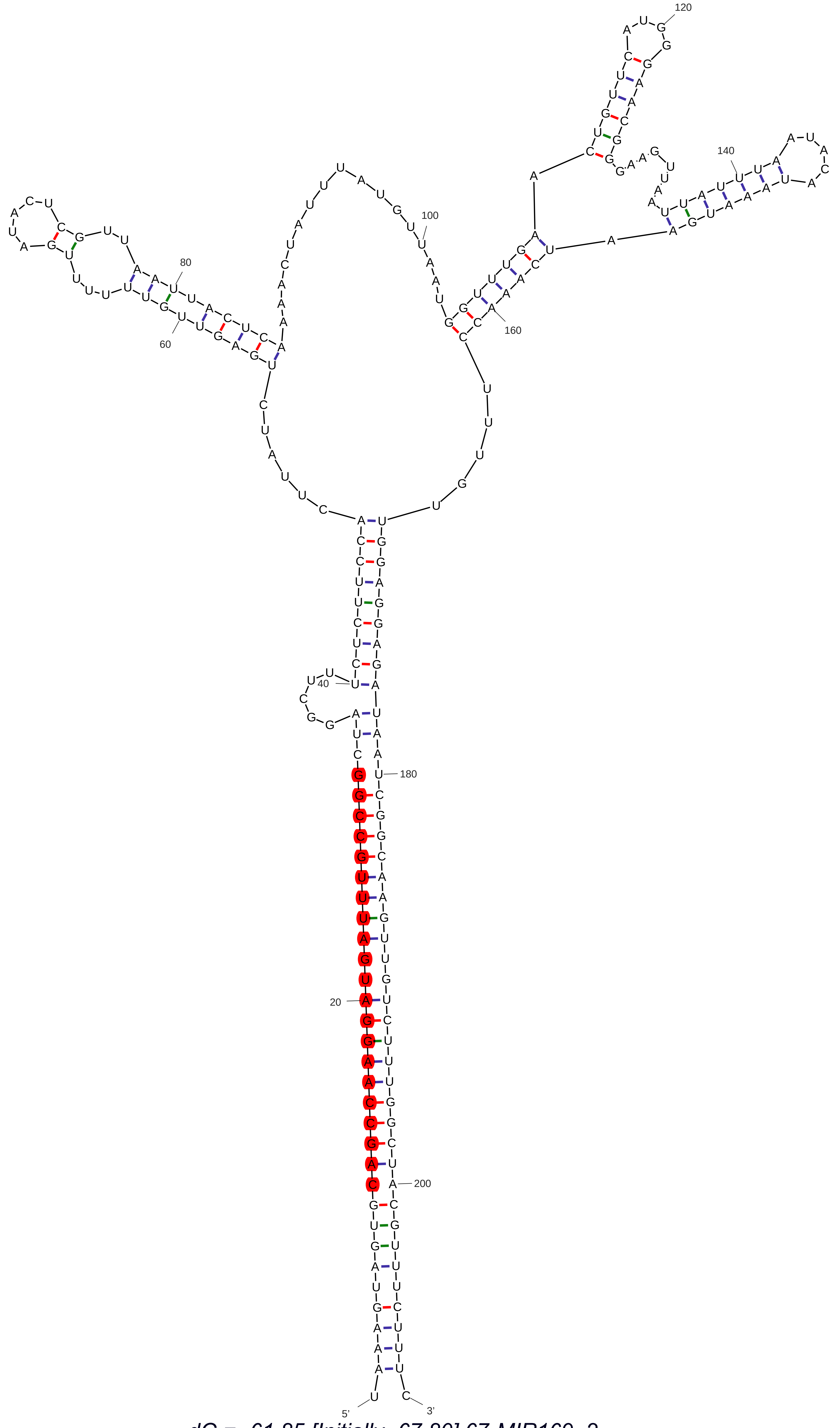

dG = -61.85 [Initially -67.80] 67-MIR169_2

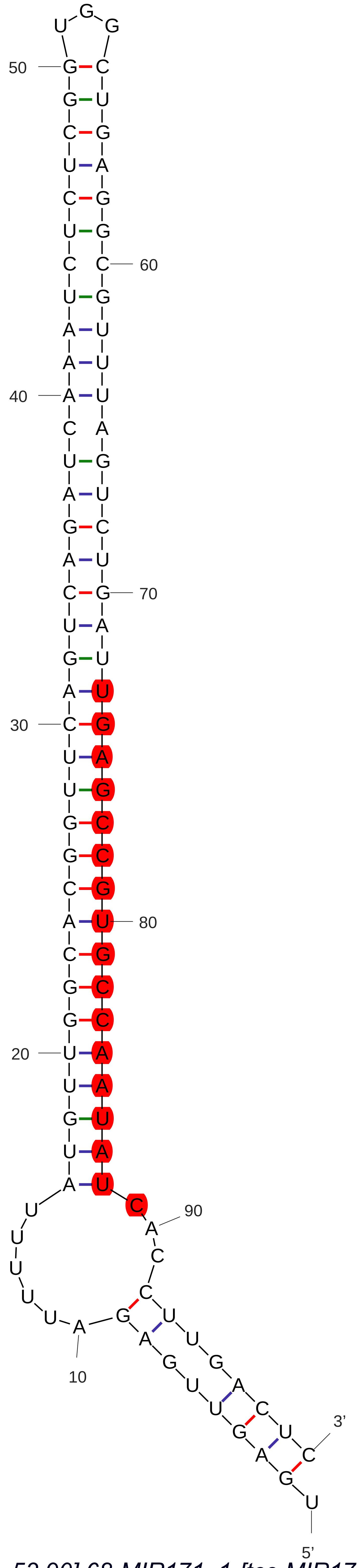

dG = -53.90 [Initially -53.90] 68-MIR171_1-[tcc-MIR171g MI0017495]

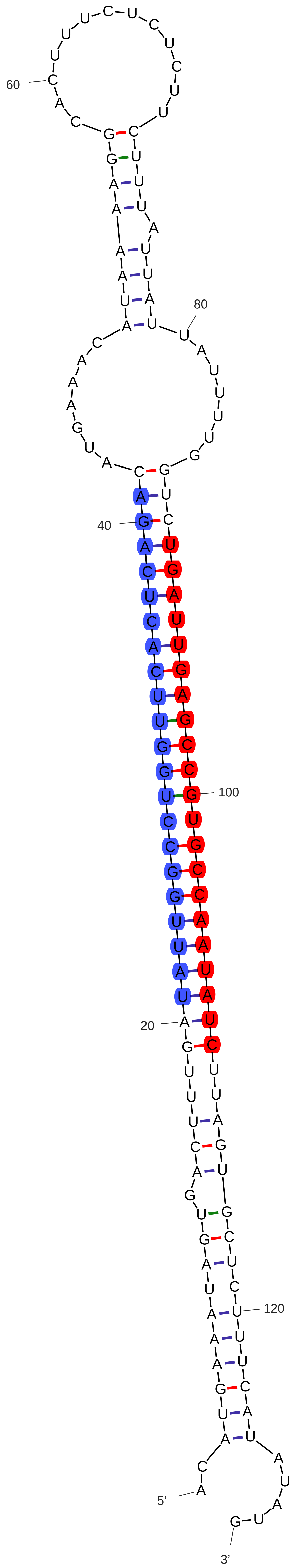

dG = -43.50 [Initially -43.50] 69-MIR171_1

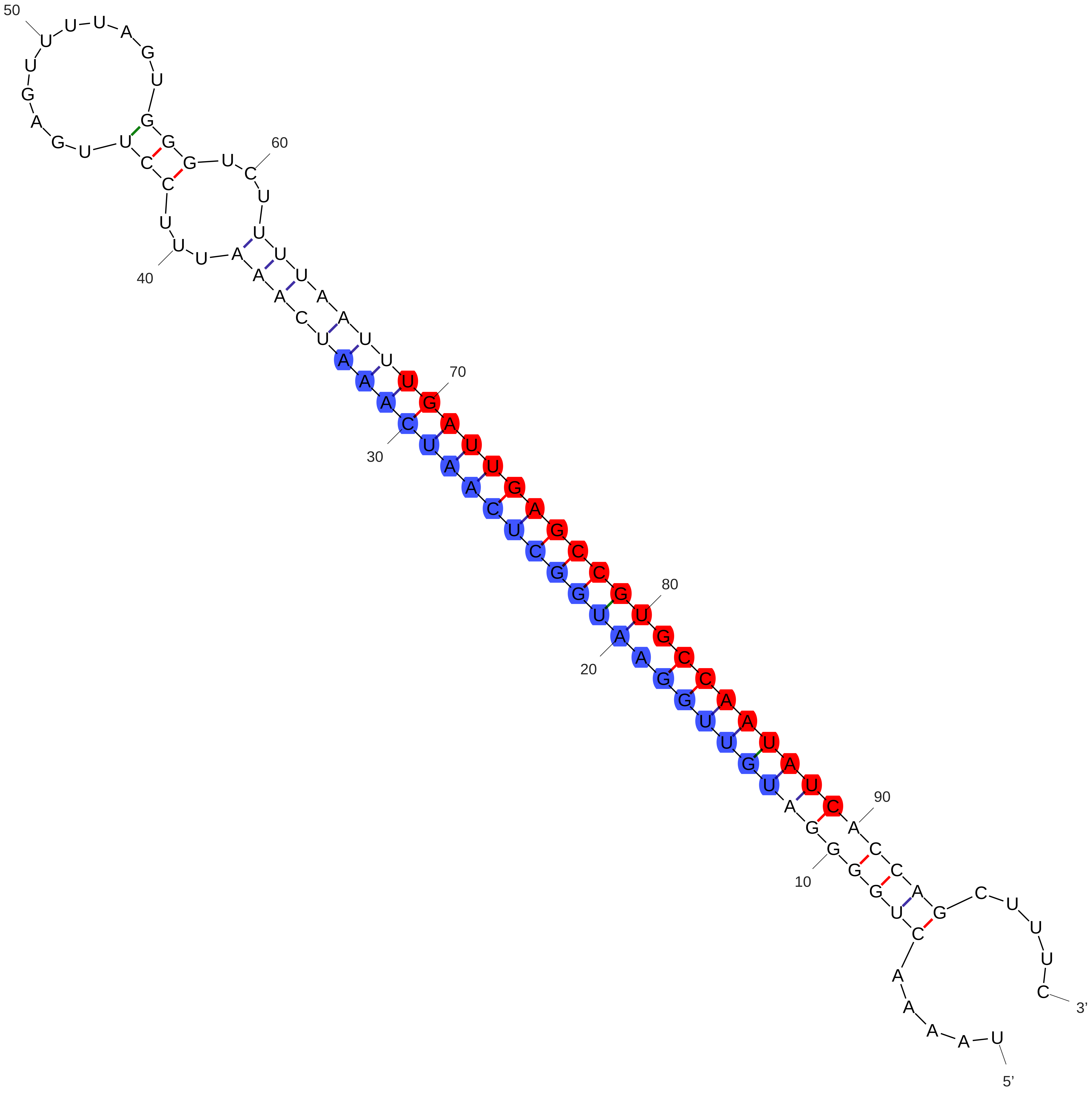

dG = -42.50 [Initially -43.00] 70-MIR171_1-[tcc-MIR171f MI0017494]

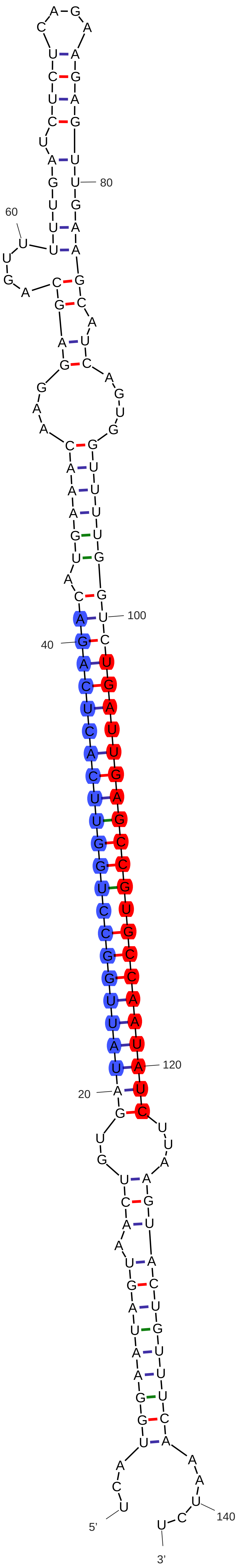

dG = -56.20 [Initially -56.20] 71-MIR171_1

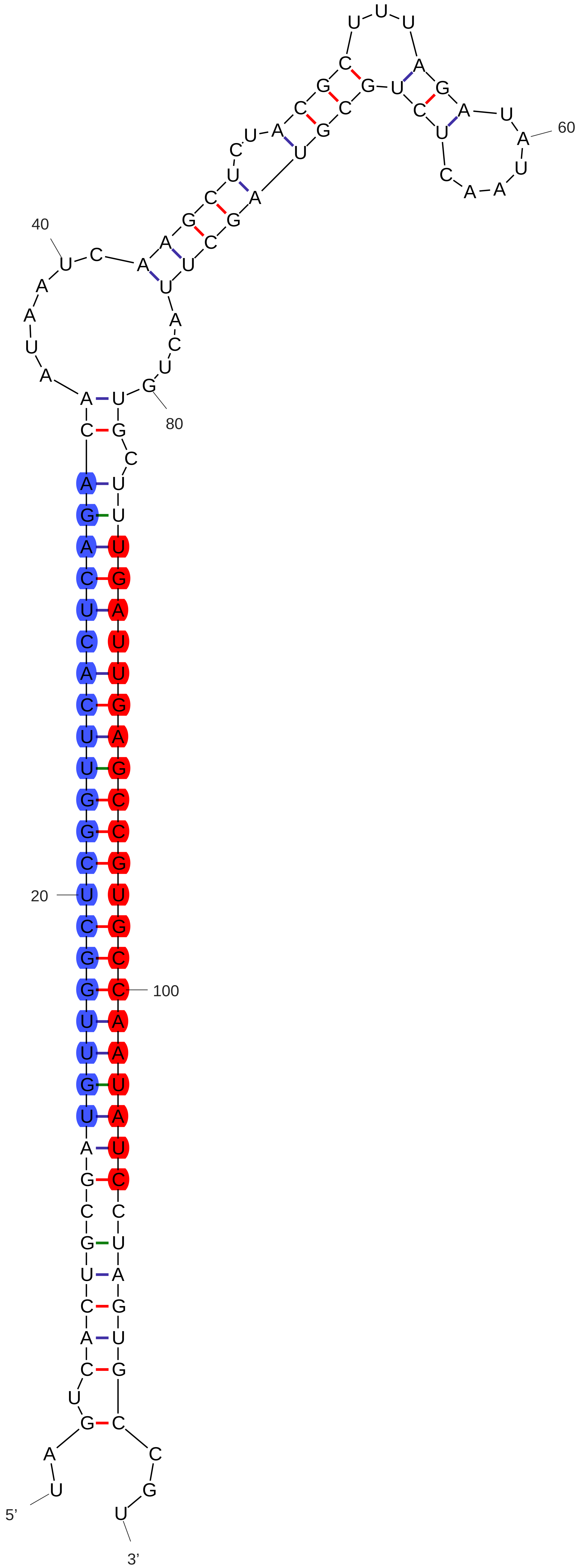

dG = -45.90 [Initially -45.90] 72-MIR171_1

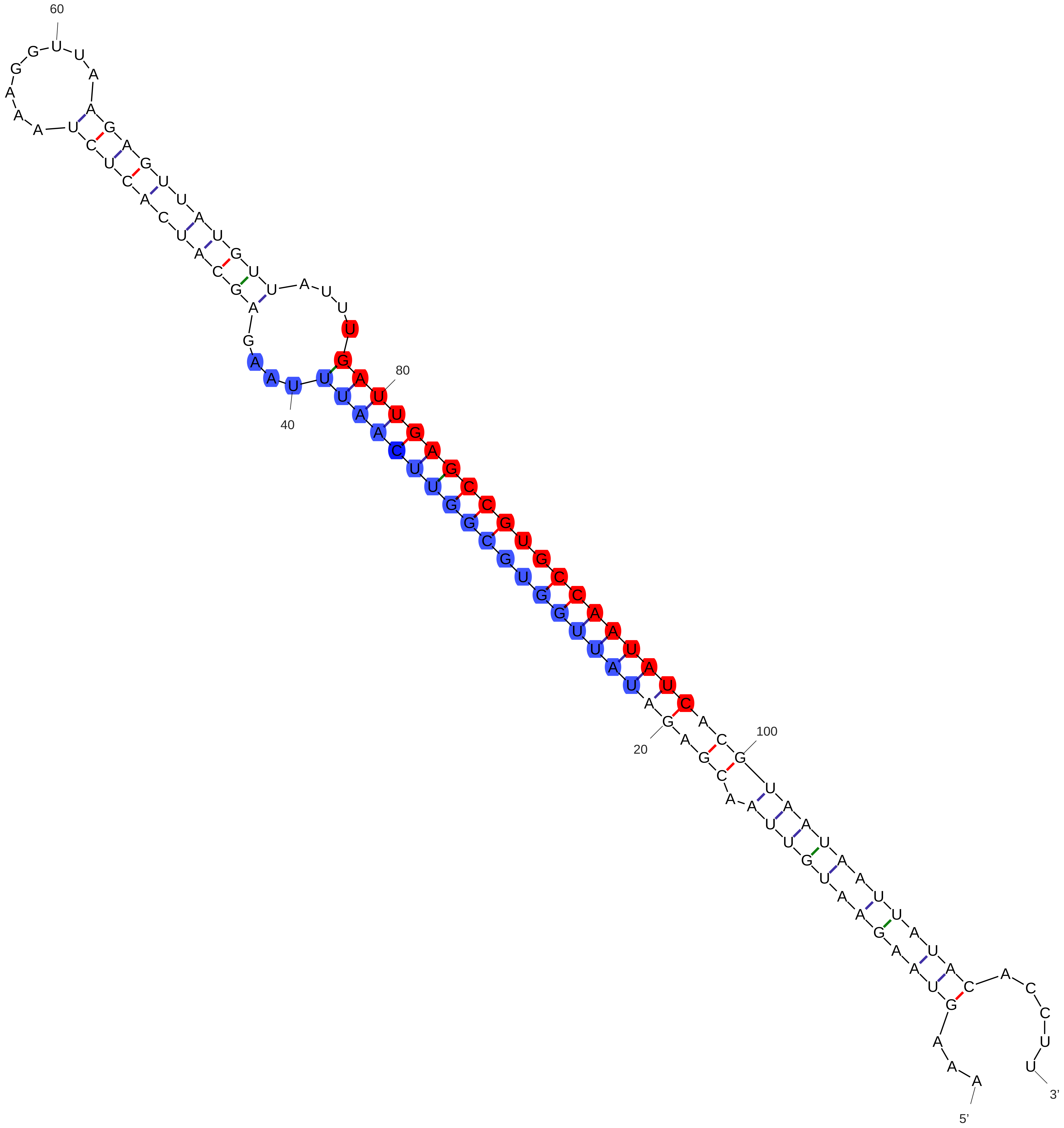

dG = -48.40 [Initially -48.40] 73_MIR171_1-[tcc-MIR171d MI0017492]

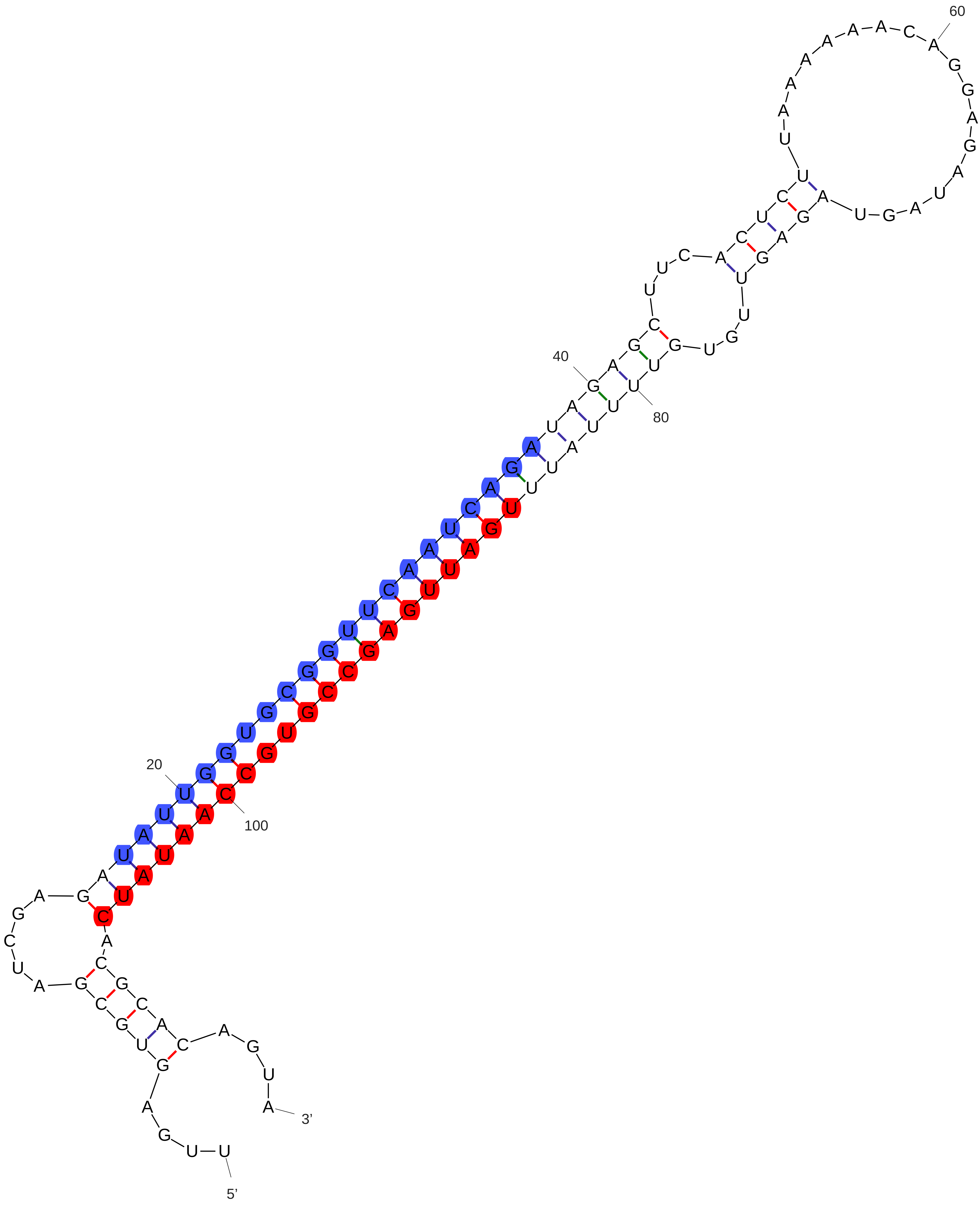

dG = -54.70 [Initially -54.70] 74_MIR171_1

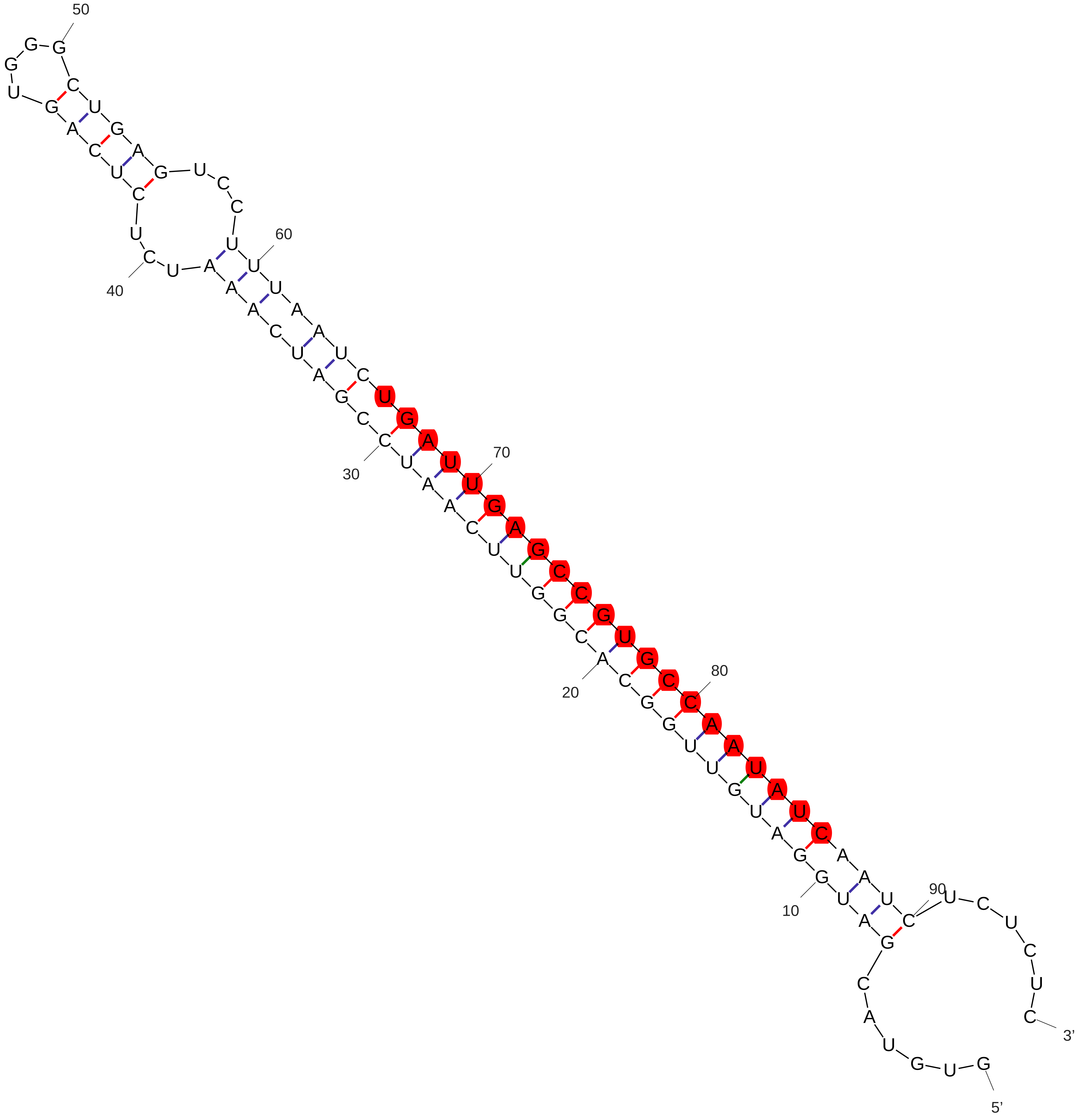

dG = -48.00 [Initially -48.00] 75-MIR171-1-[tcc-MIR171g MI0017495]

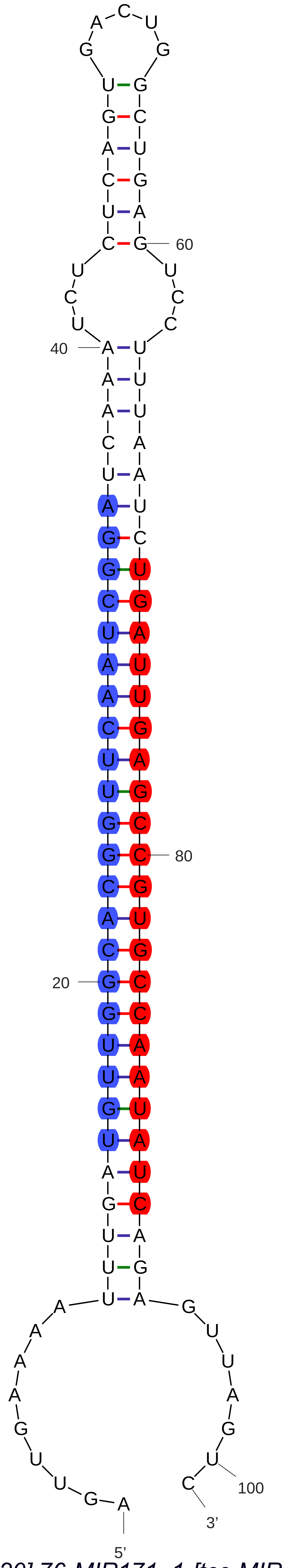

dG = -53.20 [Initially -53.20] 76-MIR171_1-[tcc-MIR171g MI0017495]

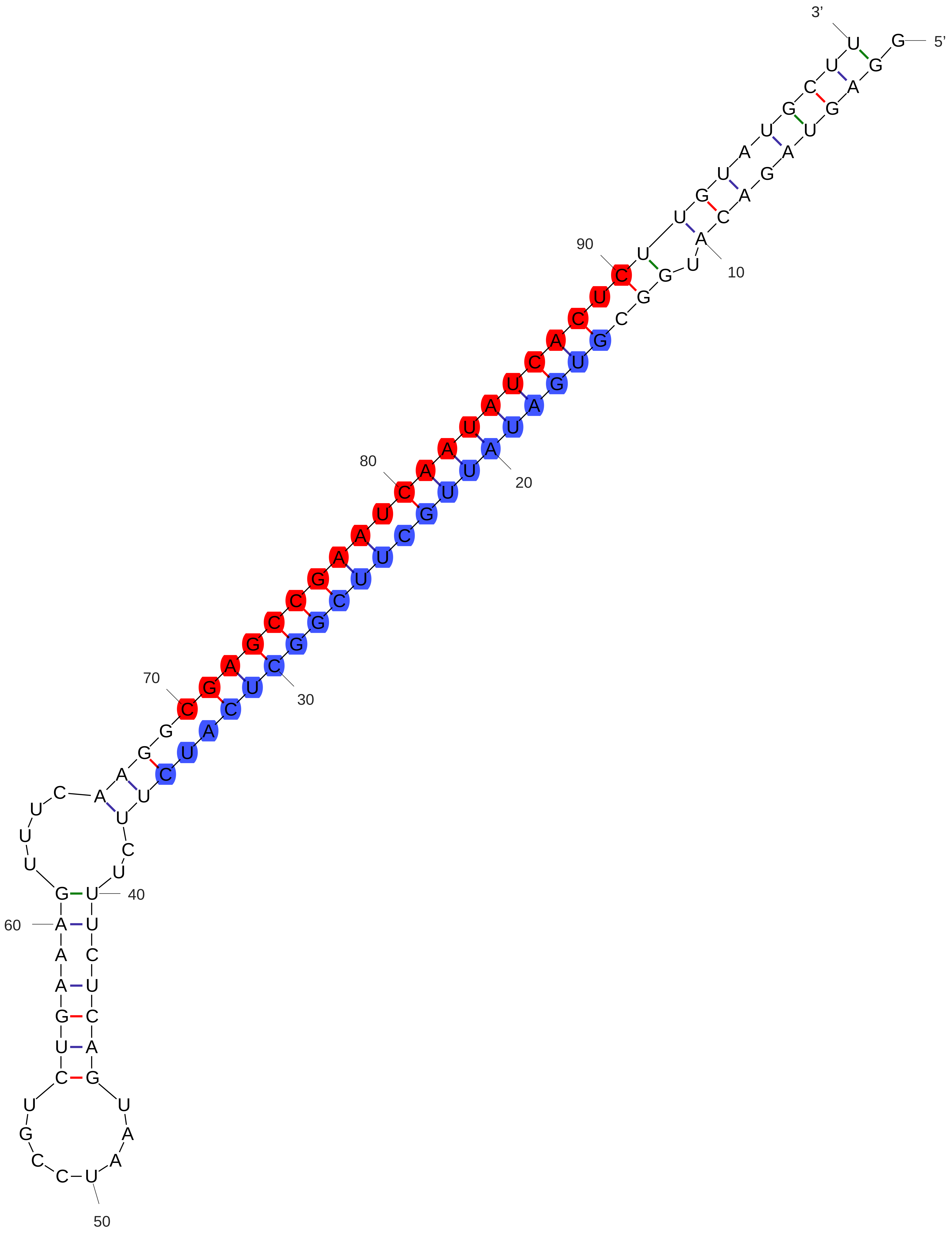

dG = -38.60 [Initially -38.60] 77-MIR171_2-[tcc-MIR172e MI0017501]

Output of sir_graph (©)
mfold_util 4.6

Created Fri Jan 18 10:49:21 2013

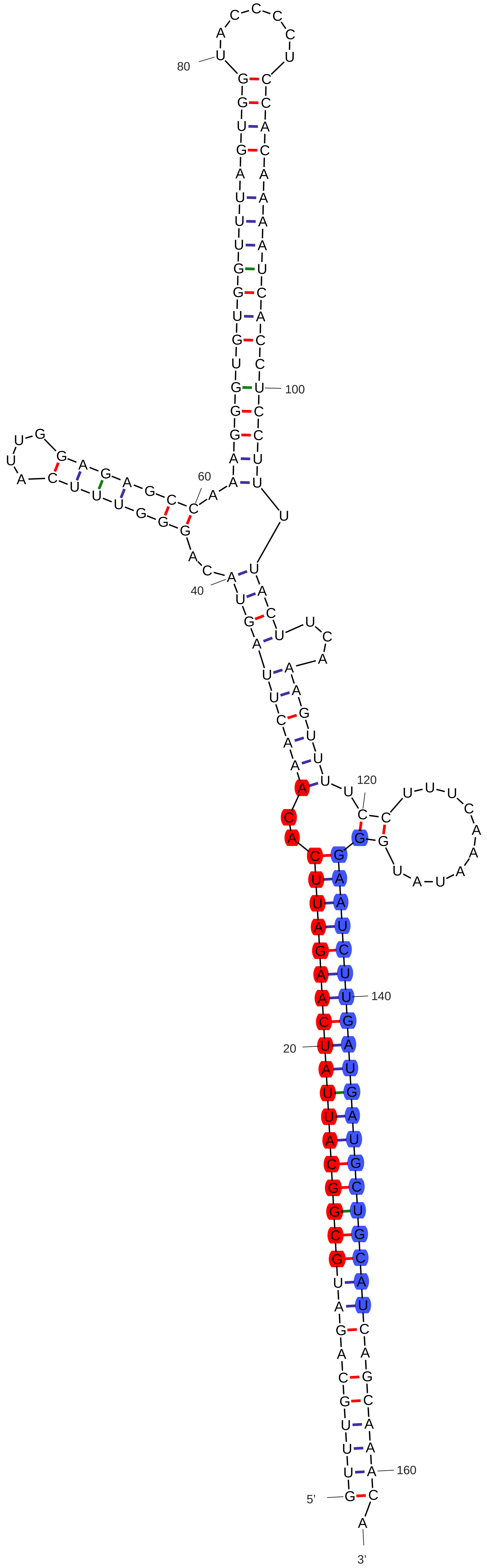

dG = -67.60 [Initially -70.30] 78_MIR172

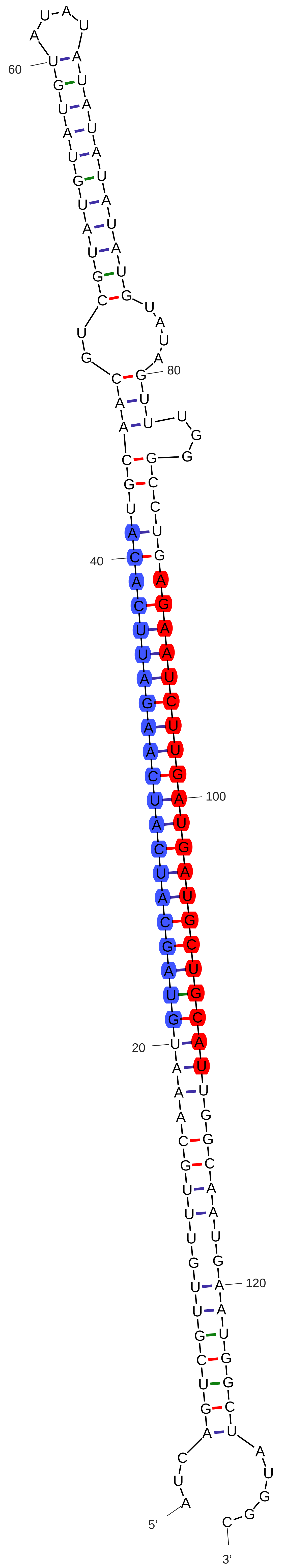

dG = -59.30 [Initially -59.30] 79-MIR172

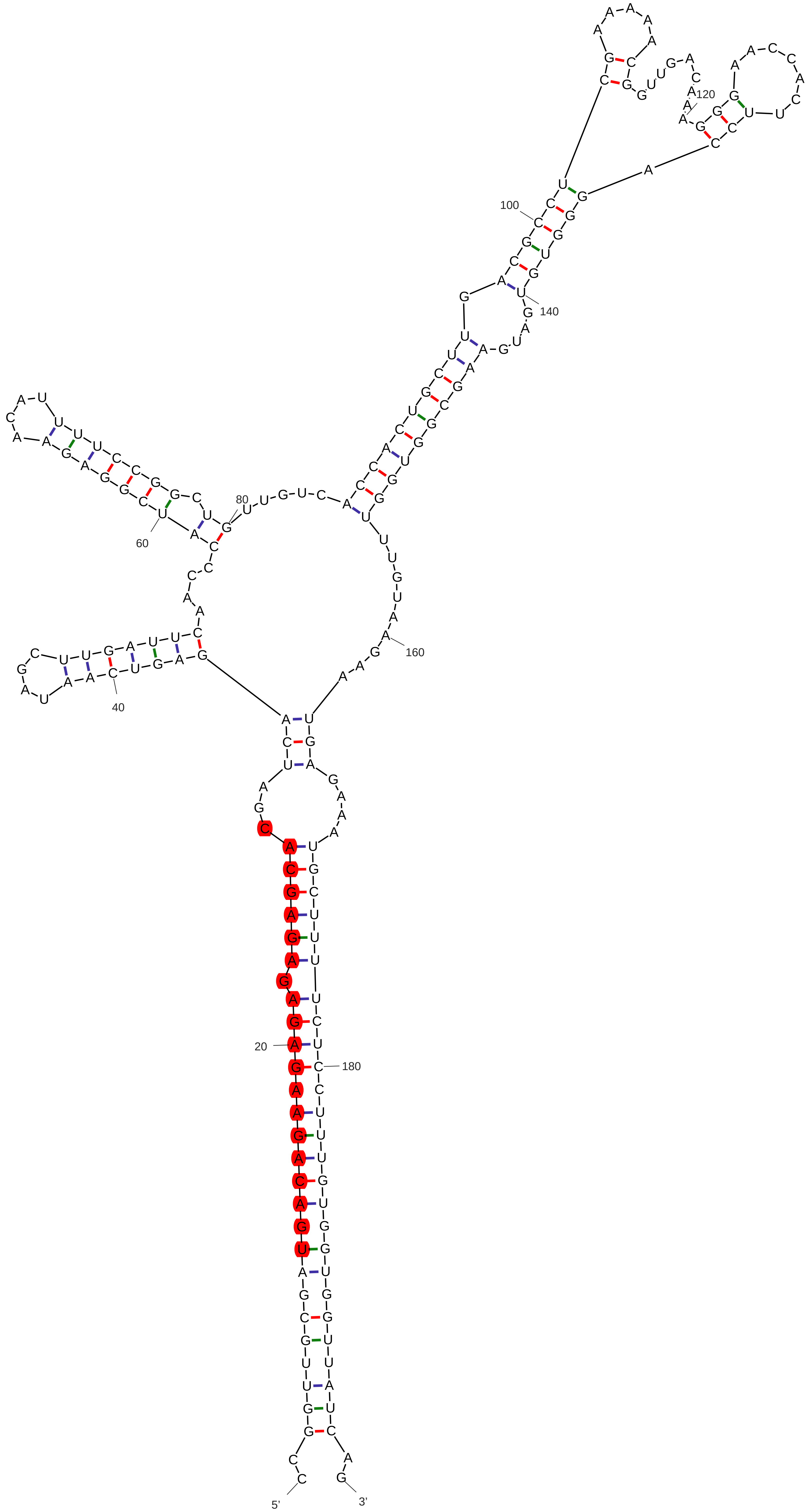

dG = -55.03 [Initially -60.20] 7-MIR156

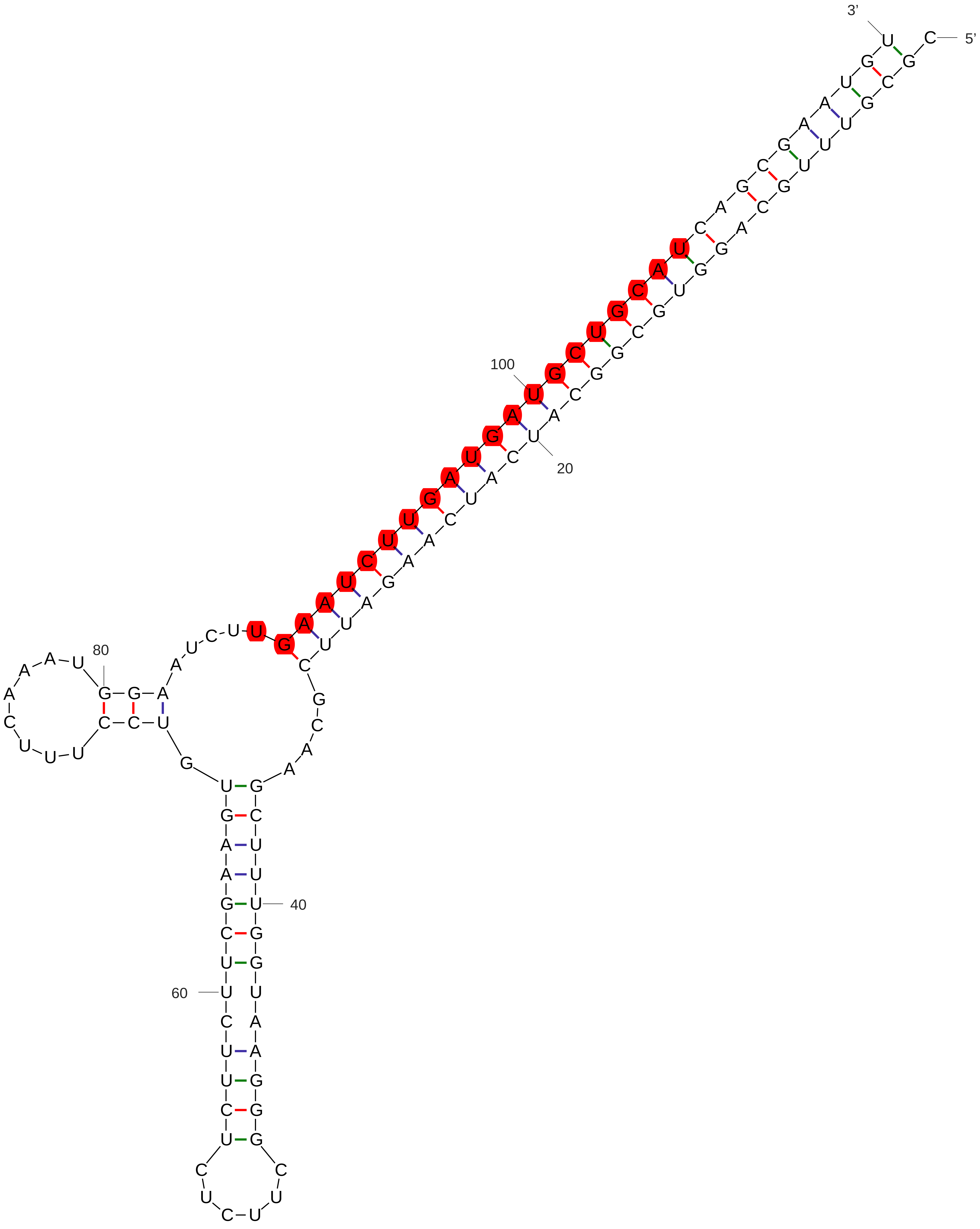

dG = -57.24 [Initially -59.10] 80-MIR172

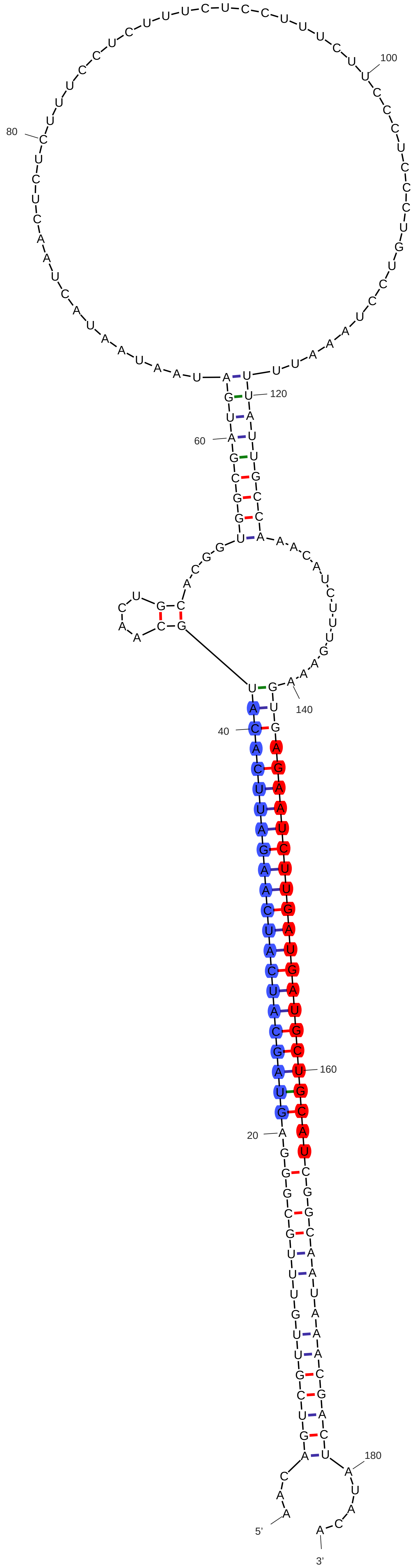

dG = -54.80 [Initially -58.45] 81-MIR172

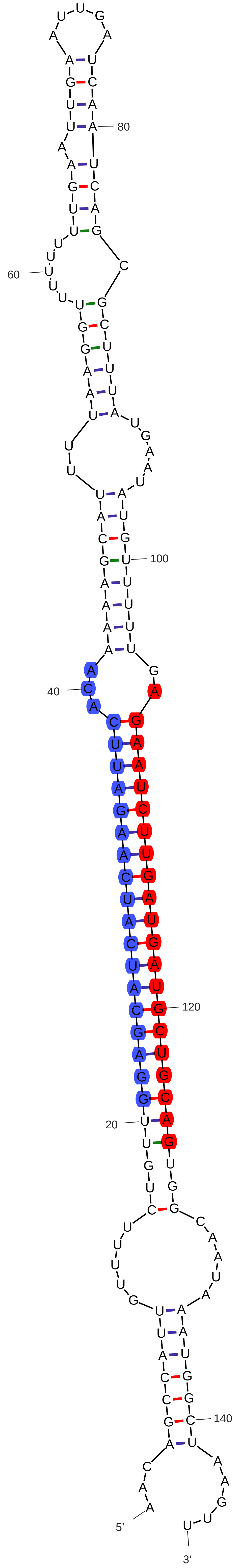

dG = -54.60 [Initially -54.60] 82-MIR172

Output of sir_graph (©)
mfold_util 4.6

Created Thu Jan 24 20:53:32 2013

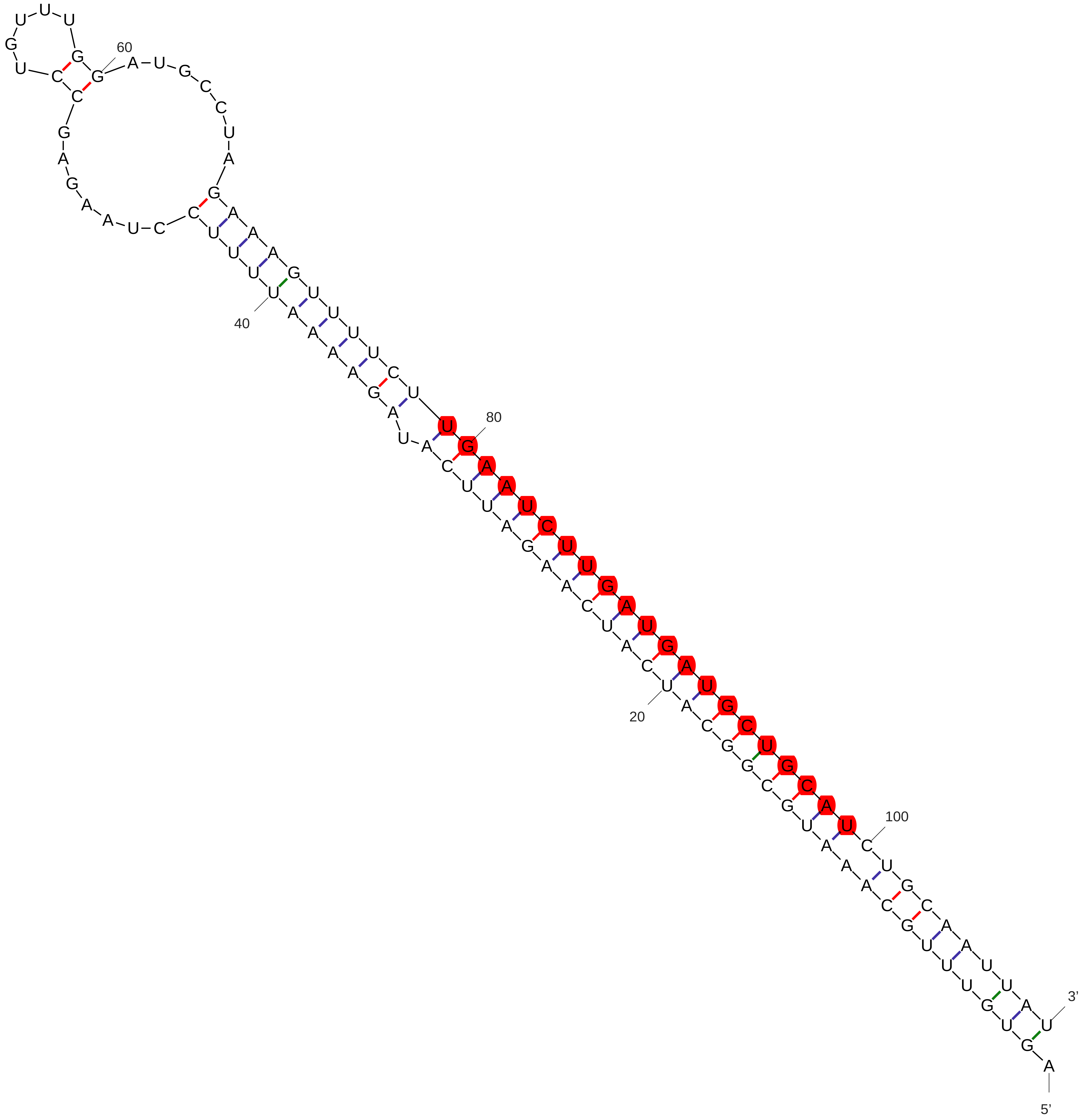

dG = -54.40 [Initially -54.40] 85-MIR172

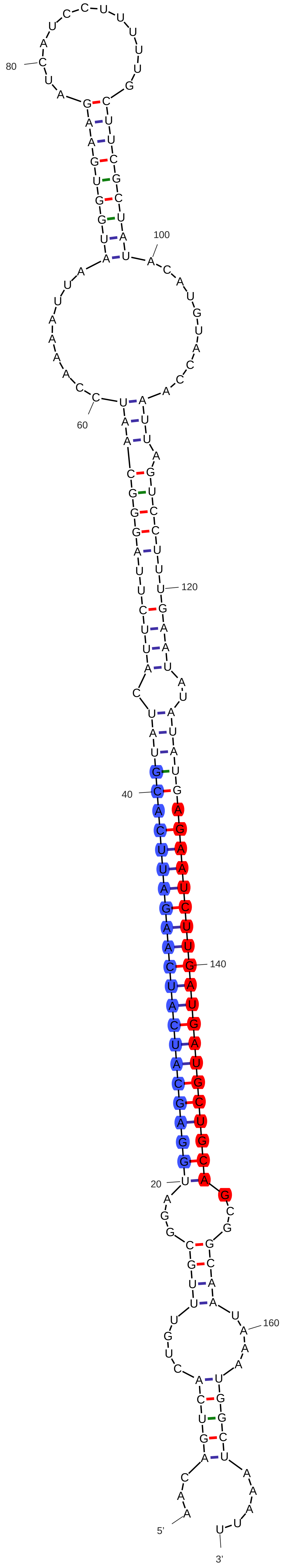

dG = -60.00 [Initially -60.00] 86-MIR172

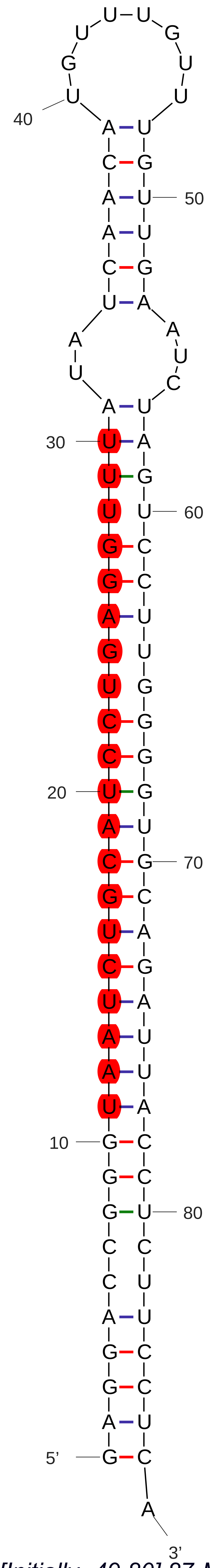

dG = -49.80 [Initially -49.80] 87-MIR2111

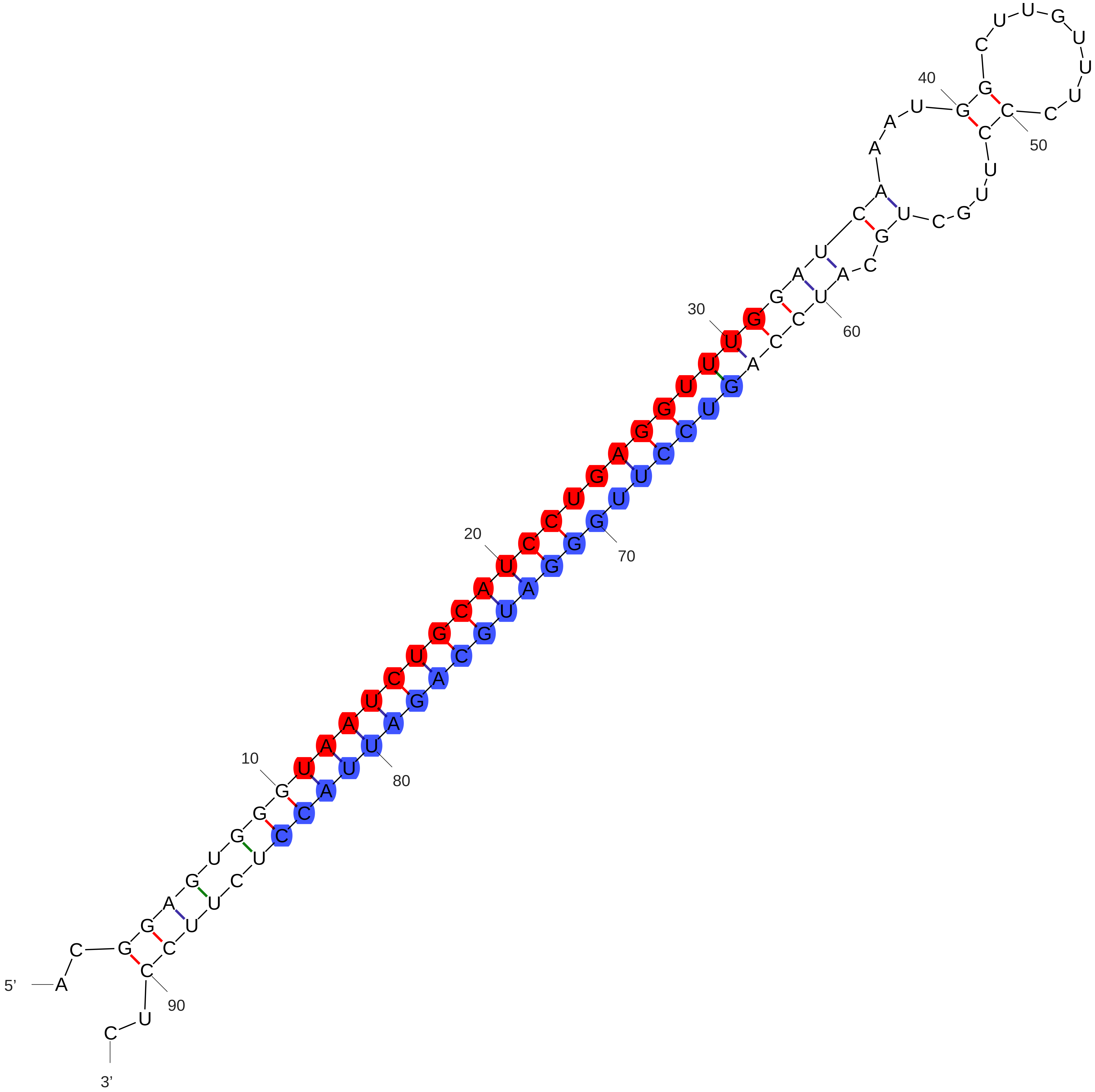

dG = -49.60 [Initially -49.60] 88-MIR2111

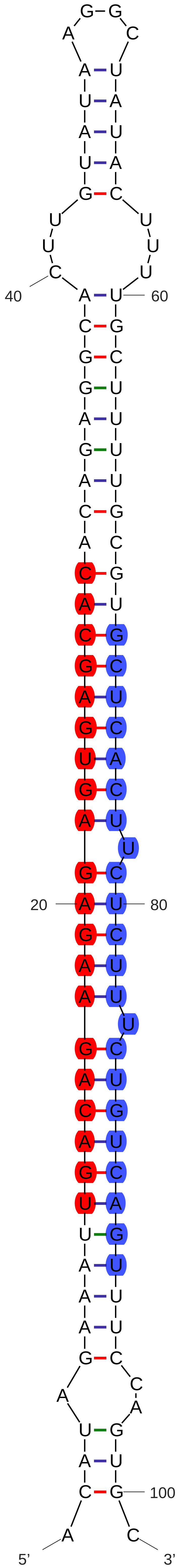

dG = -53.30 [Initially -53.30] 8-MIR156-[tcc-MIR156g MI0017459]

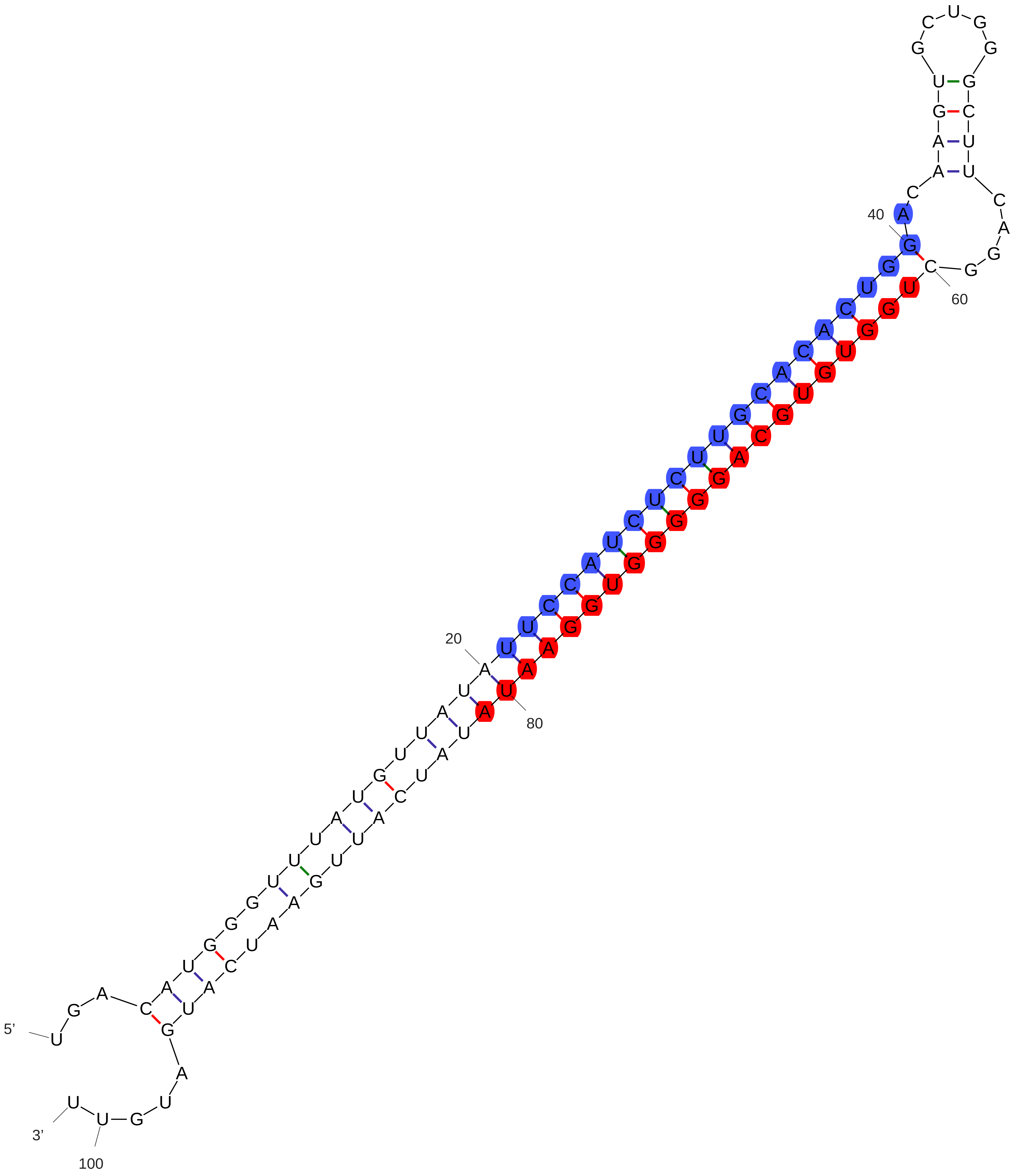

dG = -47.40 [Initially -47.40] 90-MIR2950

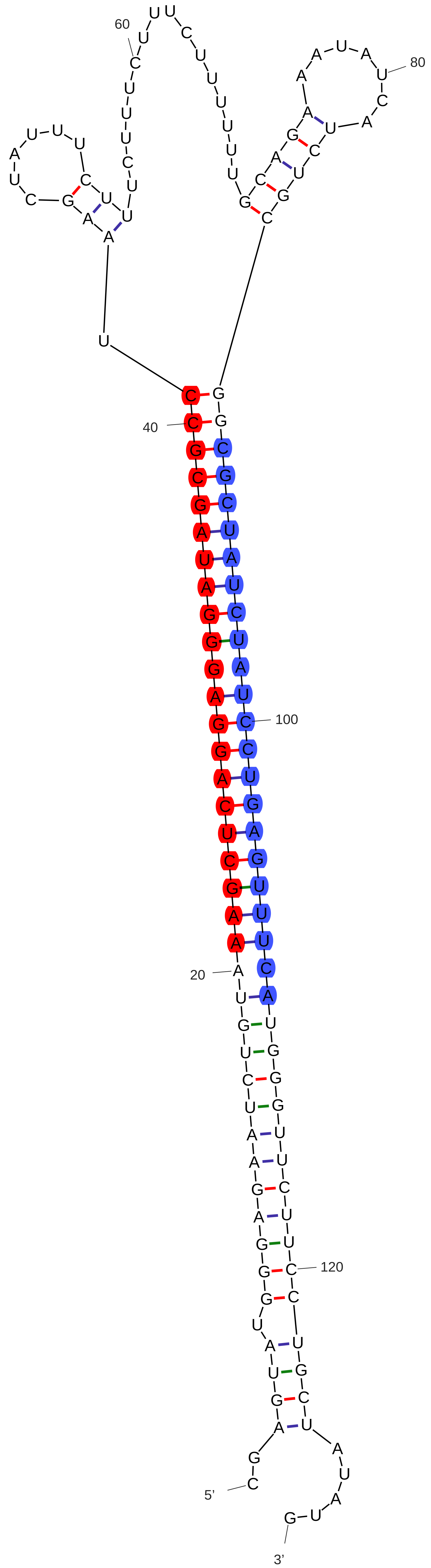

dG = -54.95 [Initially -57.30] 94-MIR390

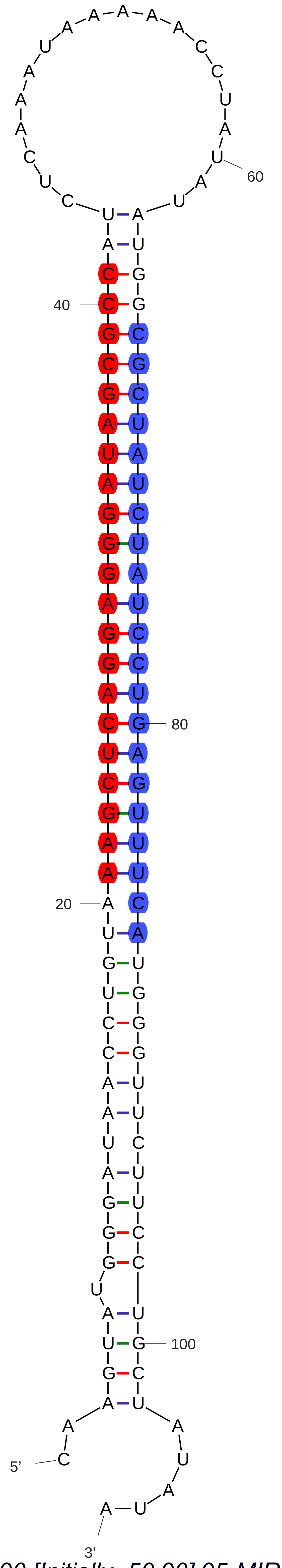

dG = -50.90 [Initially -50.90] 95-MIR390

Output of sir_graph (©)
mfold_util 4.6

Created Thu Jan 24 21:08:00 2013

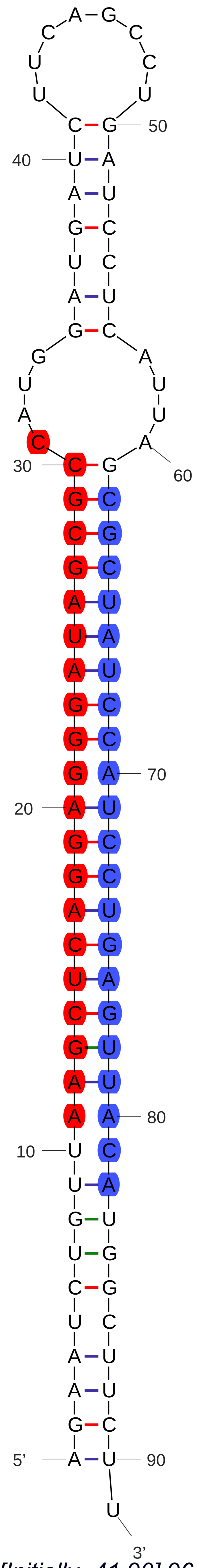

dG = -41.90 [Initially -41.90] 96-MIR390

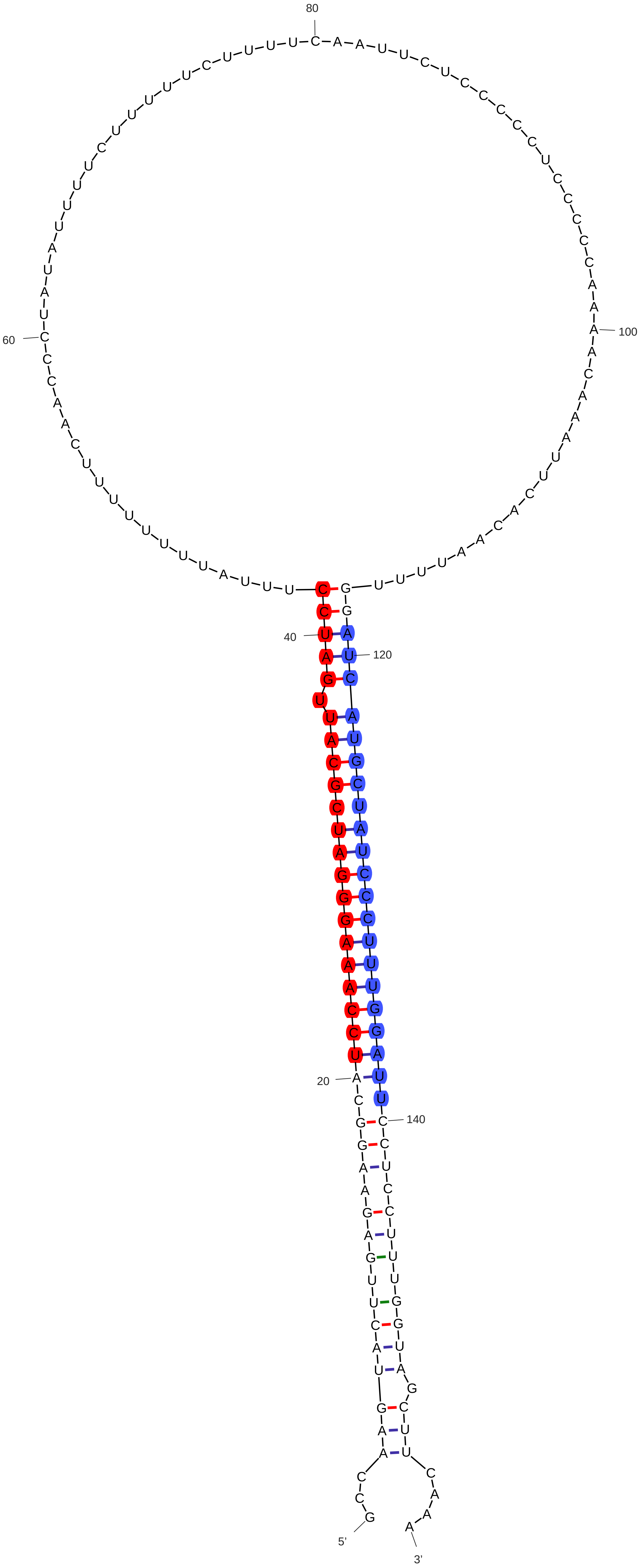

dG = -41.03 [Initially -41.02] 97-MIR393

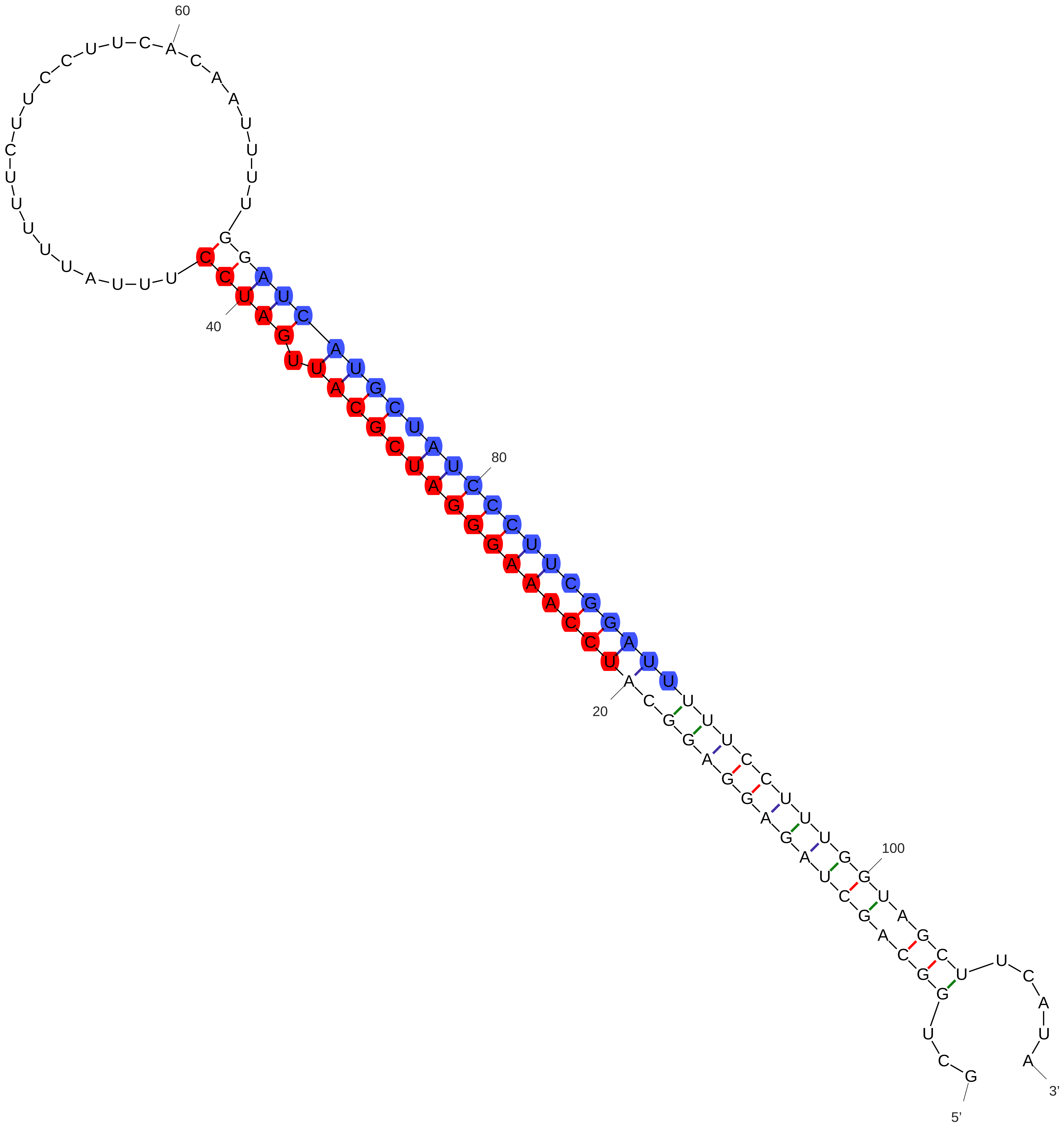

dG = -45.20 [Initially -45.20] 99-MIR393
