# Supplementary material for: Genome-wide analysis of small RNAs reveals eight fiber elongation-related and 257 novel microRNAs in elongating cotton fiber cells
Source: BMC Genomics. 2013 Sep 17;14:629. doi: 10.1186/1471-2164-14-629 (PMC3849097; doi:10.1186/1471-2164-14-629)
Supplement: Additional file 4: Table S3 — The 257 novel miRNAs in cotton fibers. [file 1471-2164-14-629-S4.docx]

**Additional Table S3：**

**The 257 novel miRNAs in cotton fibers**

| **Name** | **Sequence** | **Length (nt)** | **Precursor number** | **Counts (dpa)^a^** | | | | **miRNA*^b^** | **Annotation^c^** |
| --- | --- | --- | --- | --- | --- | --- | --- | --- | --- |
|  |  |  |  | **5** | **10** | **15** | **20** |  |  |
| GhmiRnA | ACAGCUUUAGAAAUCAUCCCU | 21 | 2 | 1822 | 6096 | 3901 | 4144 | Y |  |
| GhmiRnB | AUGAGCUAGAAGUUGGAACUC | 21 | 1 | 340 | 1062 | 851 | 600 | Y |  |
| GhmiRnC | UAAGUGAAGAAAGAGGUAGGUU | 22 | 1 | 337 | 1210 | 1012 | 593 | Y |  |
| GhmiRnD | UCUGUCGCAGGGGAGAUGGCUG | 22 | 1 | 951 | 2093 | 1648 | 2936 |  |  |
| GhmiRnE | UUGGUAUGGAGGAUGGAAAAG | 21 | 1 | 112 | 347 | 273 | 196 |  |  |
| GhmiRnF | UCGGACUGGAUUUGUUGACAA | 21 | 1 | 165 | 238 | 572 | 251 | Y |  |
| GhmiRnH | UCAGAUCAUCUUGCAGCUUCA | 21 | 2 | 67 | 207 | 97 | 146 |  |  |
| GhmiRnJ | UCGGAUCUUCAAACGGUGGAG | 21 | 2 | 374 | 378 | 230 | 120 | Y |  |
| GhmiRnK | CGGACCCUCUAACAGUGGAGG | 21 | 1 | 67 | 75 | 168 | 369 | Y |  |
| novel_mir_1000 | UUUGGAAAGUUAUAAAAUGGUCAU | 24 | 1 | m | - | 7 | m |  |  |
| novel_mir_1003 | AGGAGGAAAUCUGAUUUGUCAUUC | 24 | 1 | m | 5 | 0 | m |  |  |
| novel_mir_1004 | UUUCCAAUAGAAGAAUGACA | 20 | 1 | 8 | m | 0 | m |  |  |
| novel_mir_1006 | UAUUGAAUUAUUUAGAACUAGGAU | 24 | 1 | 0 | - | 0 | 6 |  |  |
| novel_mir_1050 | UCAUGGACUUUAGCGGCGUU | 20 | 1 | - | 13 | 8 | m |  |  |
| novel_mir_1071 | UUAACGUUUGUUAACUUUGUUGAU | 24 | 2 | m | 14 | 8 | m |  |  |
| novel_mir_1073 | UUAUUUUGGAAUUAGAAAAGUCGU | 24 | 1 | m | 17 | 8 | m |  |  |
| novel_mir_1085 | CGACUUGCUGACGUGGAAGGAAAU | 24 | 1 | 22 | 43 | 18 | 35 |  | L |
| novel_mir_1087 | UCGUGAUCUUUAGCGGUGUUU | 21 | 2 | 9 | 24 | 30 | m |  | L |
| novel_mir_111 | AGUAGUCUAAUUGGUAUAGCUGAA | 24 | 3 | m | 7 | 0 | m |  |  |
| novel_mir_1179 | CUUAAGGUGGGUUUGGAUGGGCGA | 24 | 1 | - | 16 | 16 | 20 |  |  |
| novel_mir_1200 | AGGAGAAAAAAAUCUGAUUUGUCA | 24 | 2 | 11 | - | - | m |  |  |
| novel_mir_1205 | AGAGGGAGAAGCAGAAGAGAAUA | 23 | 1 | - | 8 | - | 7 |  |  |
| novel_mir_1227 | UAGUGAGGAUGGGAAAUUUGU | 21 | 1 | 10 | 16 | 16 | 9 | Y |  |
| novel_mir_1251 | AUACAUGAACUUCGAUUUAA | 20 | 1 | m | 10 | 9 | m |  |  |
| novel_mir_1258 | GAUCAUAUCUCGUACGUUAGGACA | 24 | 1 | 11 | m | 0 | m |  |  |
| novel_mir_1290 | UUUGAUCUAACGUACAGAGACUA | 23 | 1 | 10 | m | 6 | 0 |  |  |
| novel_mir_133 | AGGAUAAAAUUACUGAUGUGGCAU | 24 | 1 | 46 | 15 | - | - |  |  |
| novel_mir_1378 | UCAAAAAAUGGGCAAAGUAGUCAU | 24 | 1 | m | 6 | 6 | m |  |  |
| novel_mir_1398 | GGAAUGUUGUCUGGACCGGGGACA | 24 | 1 | 119 | 19 | 0 | 18 |  |  |
| novel_mir_1437 | UUCCCAAAACCUCCAAUUCCAA | 22 | 1 | 13 | 88 | 51 | - | Y |  |
| novel_mir_147 | UCUGACAGUGCACUGAAAACG | 21 | 1 | 28 | 68 | 37 | 19 |  | L |
| novel_mir_153 | UUUAUACAUUAGAUCAAAGAGCAA | 24 | 1 | 16 | 20 | 23 | m |  |  |
| novel_mir_1773 | AGAUGAUGAGAAAGGAAAGUCAAG | 24 | 1 | 20 | m | - | 12 |  |  |
| novel_mir_1974 | AAAGUUGGGCCCCUGUUGGUGCGG | 24 | 2 | 9 | m | 5 | m |  |  |
| novel_mir_22 | UGUUCCAAGGAGAUGGGAUGG | 21 | 1 | 0 | 18 | 8 | 0 |  |  |
| novel_mir_2214 | UGGGCCGAGCUUGGACAAGCAUA | 23 | 1 | 26 | 14 | 8 | - |  |  |
| novel_mir_225 | UAUAUGAUCUCGGACCAGGCU | 21 | 1 | - | 635 | - | - |  |  |
| novel_mir_24 | UUAGAUUGCAUUUUACCCCUU | 21 | 1 | 9 | - | 13 | 8 |  |  |
| novel_mir_2454 | UGUAGUAAUUGUAGAAGUUCAGGG | 24 | 5 | 6 | 6 | 0 | 0 |  |  |
| novel_mir_2455 | AUAUGAAACUGAGAUACCAUG | 21 | 3 | 6 | 14 | - | 7 |  | L |
| novel_mir_2467 | AUUGGUUGUUCUGAUUCGAGGCUA | 24 | 1 | 5 | 0 | 0 | m |  |  |
| novel_mir_2470 | AGGGGCUUAGAAAGAUGGGAC | 21 | 1 | 685 | - | - | - | Y |  |
| novel_mir_2473 | GCAGCAUUAUCAAGAUUCACA | 21 | 1 | 19 | 15 | 10 | m |  |  |
| novel_mir_2474 | UCUCGGACCCUCAACGGAAGG | 21 | 1 | 12 | 28 | - | - |  |  |
| novel_mir_2477 | CCAUGAUCUUUAGCGGCGUUU | 21 | 2 | 8 | m | 0 | m |  |  |
| novel_mir_2478 | UCCCAGUUGUAGUUGGUCGUUCGG | 24 | 1 | 6 | m | 0 | 0 |  |  |
| novel_mir_2480 | UUCAAACUUAUUUUACGGCCA | 21 | 1 | 9 | - | - | m |  |  |
| novel_mir_2484 | CUAAGAGAUUGGGAUUUGGUAGGA | 24 | 1 | 8 | m | 5 | 8 |  |  |
| novel_mir_2497 | CCUGACAUGUCAGUAGAAAGCUC | 23 | 1 | 35 | 15 | 21 | m |  |  |
| novel_mir_2498 | UGUAACAGUAAGCUGACGUGACA | 23 | 1 | 23 | m | 0 | m |  |  |
| novel_mir_2501 | UAGGGAGGUAACGAAGCUUAACGG | 24 | 1 | 48 | - | - | - |  |  |
| novel_mir_2504 | CGUGAUCUUUAGCGGCGUUUG | 21 | 5 | 12 | m | 0 | m |  |  |
| novel_mir_2507 | AGAACUCAUAACACAUUUAGAUAA | 24 | 1 | 9 | 58 | - | m |  |  |
| novel_mir_2518 | CUAAAGAUCUGAGCAUUAGUGGCG | 24 | 1 | 9 | m | 0 | m |  |  |
| novel_mir_2521 | UUUGAACUUGGCAACUUUUUUCAC | 24 | 1 | 8 | 21 | 18 | 6 |  |  |
| novel_mir_2522 | AUGUGUCAAAAUGUGAGGCUGUCA | 24 | 1 | 5 | m | 0 | m |  |  |
| novel_mir_2523 | AGAAGUCGAAUUGCAUUUUG | 20 | 1 | 6 | - | 0 | m |  |  |
| novel_mir_2528 | UAUAGAUCAGGGUCUUUAGCGGCG | 24 | 1 | 25 | m | 5 | m |  |  |
| novel_mir_2529 | UGCCAAAUCAGGGAAGCGAAA | 21 | 1 | 13 | 16 | 0 | - |  |  |
| novel_mir_2531 | AAUUUUUUAAGGUUGUUUGUGGAA | 24 | 2 | 5 | 0 | 0 | 0 |  |  |
| novel_mir_2533 | AGGUGAUGAUGUGGUACAAUCUCA | 24 | 1 | 6 | m | 0 | m |  |  |
| novel_mir_2537 | AGGGAAAUUGAUUCAAGUGAGCAU | 24 | 1 | 8 | 9 | - | 6 |  |  |
| novel_mir_2538 | UGUUGACGUUGCAUACAUGUGGAU | 24 | 1 | 9 | m | 0 | m |  |  |
| novel_mir_2541 | UGAAAUUUGUAGAGAGAUAACGCU | 24 | 1 | 7 | m | 9 | m |  |  |
| novel_mir_2542 | AUAUGUUCUGAUUAUAUCUGU | 21 | 1 | 5 | 7 | 0 | 0 |  |  |
| novel_mir_2543**^e^** | UUUGGUGUUGAAGGGGAAUAA | 21 | 2 | 8 | 30 | - | m |  |  |
| novel_mir_2546 | AACCGAUGGUUGAACAUGUUAGAC | 24 | 1 | 6 | m | 0 | m |  |  |
| novel_mir_2552 | AUGGAGGAAAACAGAGGGAGAAGC | 24 | 1 | 11 | m | 0 | m |  |  |
| novel_mir_2554**^e^** | ACAUAUUAAGAAGUCGAAUUG | 21 | 2 | 7 | 22 | 16 | 9 |  |  |
| novel_mir_2555 | UUGGUACCUGAACUUGACGUCU | 22 | 1 | 9 | 18 | 9 | 5 |  |  |
| novel_mir_2557 | UGAAAUUUGUAGAGACAAAACGCU | 24 | 1 | 5 | m | 0 | m |  |  |
| novel_mir_2558 | AUGACUGUUUUAAUGAAGAUUGCG | 24 | 1 | 23 | - | 0 | m |  |  |
| novel_mir_2561 | UGUGGUUGUGACACGUGGUGGCUU | 24 | 1 | 11 | m | 0 | m |  |  |
| novel_mir_2563 | AGGGACAAUUAACUUUAACGGUCA | 24 | 1 | 21 | 6 | - | - |  |  |
| novel_mir_2564 | AGACUUGUUCAUGGGUCGGGC | 21 | 1 | 12 | 8 | 9 | 8 |  |  |
| novel_mir_2565 | CAUGUUUUUCCUGUUCAUCUUC | 22 | 1 | 22 | 92 | 91 | - | Y |  |
| novel_mir_2588 | UUUGUCCACGUGAACAGAAAACGC | 24 | 1 | 9 | 5 | 0 | m |  |  |
| novel_mir_2589 | GGCGUACUUUCAGAGAACAUUUUG | 24 | 1 | 5 | 0 | 0 | 0 |  |  |
| novel_mir_2592 | AGGAAAAAAAAUCUGAUUUGUCAU | 24 | 1 | 10 | m | 0 | m |  |  |
| novel_mir_2595 | GUGUAUCUCCUGAAAACGACGACA | 24 | 1 | 8 | m | 0 | 0 |  |  |
| novel_mir_2609 | CUAAAAUCGAGCAUAGACAUC | 21 | 1 | 8 | 18 | 13 | m |  |  |
| novel_mir_2618 | AGGUGCAGGUGCAGGCGCAGC | 21 | 1 | 33 | 42 | 0 | m |  |  |
| novel_mir_2621 | UUCUUAUAUGUUAGAUCAAAGAGC | 24 | 1 | 13 | - | - | m |  |  |
| novel_mir_2625 | AGUCAGUUCAGGCAGUCAGACCGU | 24 | 1 | 9 | m | 0 | m |  |  |
| novel_mir_2626 | GUUAACUUUAACGGUCAACGGUU | 23 | 2 | 5 | 0 | 0 | m |  |  |
| novel_mir_2630 | UUUACUCUUUGAUCUAACGUACA | 23 | 1 | 7 | m | 6 | m |  |  |
| novel_mir_2634 | UGGACGCGCUUUGCUGACGUGGCA | 24 | 1 | 7 | m | 0 | m |  |  |
| novel_mir_2635 | GAUUUAACGUAUAGGGACUAA | 21 | 1 | 5 | 10 | - | 24 |  |  |
| novel_mir_2661 | UAUCUAACGUGUAGGGACUAA | 21 | 1 | 18 | - | 0 | - |  |  |
| novel_mir_267 | AUACAAUGCUUAGAACUAUCCAUA | 24 | 1 | - | 102 | - | 21 |  |  |
| novel_mir_2715 | ACGCAAAAUGUCCGGACUGGUCGG | 24 | 1 | 8 | m | 0 | 0 |  |  |
| novel_mir_2721 | GCUGACGUGGAAGGAAAUCGCUA | 23 | 1 | 9 | m | 0 | m |  |  |
| novel_mir_2733 | AAGAGAAAUGAUUGUAUGAAACAG | 24 | 1 | 16 | - | - | - |  |  |
| novel_mir_2741 | UGGGCUUAGAUUUUUUGCGGCGUU | 24 | 1 | 8 | 0 | 0 | 0 |  |  |
| novel_mir_2743 | CAGUGUAGUUCUAAACCCGUCGGG | 24 | 1 | 17 | - | 0 | 11 |  |  |
| novel_mir_2744 | UUGGAUGAACGGUGCGUUUACUU | 23 | 1 | 12 | - | 8 | 7 |  |  |
| novel_mir_2748**^e^** | AGGUCAUGGUCUAUAGCGGCGCUU | 24 | 1 | 9 | m | 7 | m |  |  |
| novel_mir_2765 | UUAACGGUAGAAAUGGAUGAA | 21 | 1 | 5 | m | 0 | m |  |  |
| novel_mir_2888 | ACAGUGGAGGUAUUGUGCCUG | 21 | 1 | 5 | - | - | m |  |  |
| novel_mir_2894 | UUGGACAUCCAAGUUAGCAUUUA | 23 | 1 | 8 | 9 | 0 | m |  |  |
| novel_mir_2902 | ACGAAAAAUUAAAUGGAGGGCUA | 23 | 1 | 5 | 0 | 0 | 0 |  |  |
| novel_mir_291 | UUUUUAACUUUACUGACAUGGCAU | 24 | 1 | m | 12 | 8 | 0 |  |  |
| novel_mir_2943 | UUGAACUUUGACCGGAUCUAGGGA | 24 | 1 | 10 | - | 0 | 7 | Y |  |
| novel_mir_2999 | AGGCUCUAAUGUGGGAACGACUGC | 24 | 1 | 9 | m | 7 | m |  |  |
| novel_mir_30 | UCAACGGAGUUGGGAGACAAA | 21 | 1 | 12 | 22 | 10 | m |  |  |
| novel_mir_3001 | UUGUUAACUUUGAUGAUGUGGCAU | 24 | 1 | 9 | m | 0 | m |  |  |
| novel_mir_3032 | AGGUGAUGAUGUGGUACAAUCUUA | 24 | 1 | 9 | m | 8 | m |  |  |
| novel_mir_3049 | AAGAUAUGUAAACCUUGAGGGCUA | 24 | 1 | 11 | m | - | m |  |  |
| novel_mir_3369 | CUUUUAACAGUAGAAUUUGAUGGA | 24 | 1 | 37 | - | - | 28 |  |  |
| novel_mir_3622 | AUGGACGAAAUGAAAGUAGAG | 21 | 1 | 6 | 11 | 0 | - |  |  |
| novel_mir_3635 | AGAGACAAAAGAAACACGUUCUAC | 24 | 1 | 6 | m | 6 | m |  |  |
| novel_mir_3992 | UAUGAAAAGUUACAAAAUGGUCAU | 24 | 3 | m | 9 | 0 | 0 |  |  |
| novel_mir_3994 | UUUGAAAAGUACAGGGACUAU | 21 | 1 | 0 | 6 | 0 | 0 |  |  |
| novel_mir_4000 | AGGAUGUAAAAGAAUAGGUGA | 21 | 1 | m | 7 | 0 | m |  |  |
| novel_mir_4003 | AUAAAAACUUUUGAAUAAUUCAGU | 24 | 1 | 0 | 8 | 0 | m |  |  |
| novel_mir_4004 | CGAUGGAGUCUGGAGACAAAA | 21 | 1 | m | 10 | 0 | m |  |  |
| novel_mir_4008 | AAUGAAUCUAGGUUCUCUCUU | 21 | 1 | 0 | 5 | 0 | m |  |  |
| novel_mir_4009 | AAUGAAUCUAGUUUCUCUCUU | 21 | 1 | m | 80 | 53 | - |  |  |
| novel_mir_4016 | UAUCUUAUUCAUCUUGGACUG | 21 | 2 | m | 6 | 0 | m |  |  |
| novel_mir_4020 | UAAAUCUACGUAUGGAAAUUC | 21 | 1 | 0 | 9 | 0 | 0 |  |  |
| novel_mir_4037 | UGGAAAAGUGGUAAUAAGGGG | 21 | 2 | 0 | 7 | 0 | 0 |  |  |
| novel_mir_4038 | UGCCAUGUAGGAUUGUCGUUA | 21 | 1 | - | 179 | - | - |  |  |
| novel_mir_4040 | CGCUUUGCUGACGUGGAAACAAAU | 24 | 2 | 0 | 12 | 0 | m |  |  |
| novel_mir_4044 | AAGGGAUACAGGAUGAUGUGGCAU | 24 | 1 | - | 17 | - | 32 |  |  |
| novel_mir_4047 | CGGACUCUCAAACAGUGGAGGUA | 23 | 1 | m | 9 | 56 | 36 |  |  |
| novel_mir_4051 | UUGUUAAAAGUUUCAUCCAUU | 21 | 2 | 0 | 9 | 0 | 0 |  |  |
| novel_mir_4053 | UUAGUGGAGCUCAUGCUAGAA | 21 | 1 | m | 6 | 0 | m |  |  |
| novel_mir_4056 | UUAAUUAUUAUAUAGAUCAAGGAU | 24 | 1 | 0 | 5 | 0 | 0 |  |  |
| novel_mir_4061^e^ | UUUUACAGCAGCUACAUCCAU | 21 | 1 | m | 14 | 11 | 6 |  |  |
| novel_mir_4065 | AUAUCAUUGGUGAUGUAUCGUCUU | 24 | 1 | 0 | 5 | 0 | m |  |  |
| novel_mir_4067 | AGAAUGACCGGUUUGCUCUUU | 21 | 1 | m | 6 | 9 | m |  |  |
| novel_mir_4068 | UUUGAUUAGGAAGUUUGAGGAUCA | 24 | 1 | - | 34 | - | 11 |  |  |
| novel_mir_4069 | UAAUGAUGUGGCACAAUAUUA | 21 | 1 | m | 7 | 0 | 0 |  |  |
| novel_mir_4070 | UGGGGGCGCGCUUUGCUUACG | 21 | 1 | 0 | 5 | 0 | 0 |  |  |
| novel_mir_4071 | UCGGUGGAGAUGGAUAAAAUGAAU | 24 | 1 | m | 9 | 10 | - |  |  |
| novel_mir_4072 | AGGAUGCACUGUCAGCAAAAGUAU | 24 | 2 | m | 7 | 0 | m |  |  |
| novel_mir_4073 | CUGGGACAUGGCGUUGGCAA | 20 | 1 | m | 5 | 0 | m |  |  |
| novel_mir_4080 | CUAUAAACAGUCGAUGGUAUC | 21 | 1 | m | 5 | 0 | 0 |  |  |
| novel_mir_4084 | UAGUAUAAGGACUAAAUUGGU | 21 | 1 | 0 | 10 | 9 | 6 | Y |  |
| novel_mir_4085 | UUGGUGAUCAUAAGUACAUAA | 21 | 1 | 0 | 5 | 0 | 0 |  |  |
| novel_mir_4086 | UUAACAUUUGUUAACUUUGCUGAC | 24 | 2 | m | 8 | 7 | m |  |  |
| novel_mir_4110 | UGAGGCAUGAACUUGGCAAUUUU | 23 | 1 | 0 | 5 | 0 | 0 |  |  |
| novel_mir_4112 | GGGCACCUCUCACUUAGGCAGG | 22 | 1 | m | 10 | 0 | m |  |  |
| novel_mir_4127 | CAUCAGCGCGAUGUCAGGGGACGU | 24 | 1 | - | 8 | 6 | 7 |  |  |
| novel_mir_4154 | ACUAGCUCAGGCUCUCGGCAG | 21 | 1 | m | 5 | 0 | m |  |  |
| novel_mir_4168**^e^** | CAUGUGCCUUGGCUCUCCAUC | 21 | 1 | m | 14 | 7 | 6 |  | L |
| novel_mir_4175 | CAGCAACUCGCGCUGACGUGGACA | 24 | 1 | - | 6 | 0 | 9 |  |  |
| novel_mir_4192 | AUGGGCUGGCACAUGGGCGUGUGG | 24 | 1 | m | 12 | 0 | 0 |  |  |
| novel_mir_4199 | UUUUGGAAUCAAGACUACAU | 20 | 1 | 0 | 5 | 0 | 0 |  |  |
| novel_mir_4245 | UUGGAUUGUUAAAAGGUUAAUUGU | 24 | 1 | m | 5 | 0 | 0 |  |  |
| novel_mir_4246 | UUUUCAACUCUGCCAAGCAAU | 21 | 1 | m | 11 | 0 | m |  |  |
| novel_mir_4249 | UGAUGGGAAUGUUGUUUGGCU | 21 | 1 | - | 235 | 87 | - |  |  |
| novel_mir_4254 | AAUUUGGACUGUCACGUAGGA | 21 | 1 | 0 | 10 | 0 | m |  |  |
| novel_mir_4261 | UUUGGUUAGAUAGUUAGAUAAUU | 23 | 1 | m | 21 | 12 | m |  |  |
| novel_mir_4268 | CAUUACUUUUUCAUUCAUUAA | 21 | 1 | 0 | 5 | 0 | 0 |  |  |
| novel_mir_4278 | AAAGACGAAAGACAAAAUCUCAAU | 24 | 1 | m | 7 | 5 | m |  |  |
| novel_mir_43 | AGGCCCCUGUAUUGAGAGUCGGAU | 24 | 1 | 284 | 317 | 295 | 340 |  |  |
| novel_mir_4300 | UUAAUACUGUUAAAUUUGUUGGU | 23 | 1 | m | 6 | 0 | 0 |  |  |
| novel_mir_4309 | AGUUCAUACUAGUUCGUGGGUCA | 23 | 1 | 0 | 5 | 0 | m |  |  |
| novel_mir_4315 | AACUGUUCGUGAUAAGAUGUCGGU | 24 | 1 | m | 13 | 0 | m |  |  |
| novel_mir_4322 | UGGAGUCAACGGUGGGACGGAGA | 23 | 1 | 0 | 5 | 0 | 0 |  |  |
| novel_mir_4329 | UGGUGGAAGUAUUGUGCCCGG | 21 | 1 | m | 7 | 0 | m |  |  |
| novel_mir_4337 | AUACUACACUAAGGGCACUAGAUC | 24 | 1 | m | 7 | 0 | m |  |  |
| novel_mir_4342 | UUUAGCAGUAGAAAUAGAUGA | 21 | 1 | m | 21 | - | - |  |  |
| novel_mir_4352 | AUAUUAAAACUAUACAUGAACUUU | 24 | 1 | 0 | 8 | 0 | 0 |  |  |
| novel_mir_4414 | UGCCGCCUGACACACUGACGACAC | 24 | 1 | 0 | 5 | - | m |  |  |
| novel_mir_4419 | AGGAGUUAGAUUGUAUUUUAU | 21 | 1 | 0 | 9 | 0 | m |  |  |
| novel_mir_4535 | GUGGAACGUGUUAAGAGAGGAAUA | 24 | 1 | - | 12 | 10 | 12 |  |  |
| novel_mir_4977 | UGGACUGUUAAAAUUUUAAUGGCA | 24 | 1 | m | m | 10 | m |  |  |
| novel_mir_4978 | AGCAGGUCGCGCUGACGUGGACA | 23 | 1 | - | m | 16 | - |  |  |
| novel_mir_4986 | AGGAUGAAAAUAUUGAUGUAGCAU | 24 | 1 | - | - | 16 | - |  | L |
| novel_mir_4987 | UUUUAGUGAUGUGGCAGAAAGAUG | 24 | 2 | m | m | 7 | 0 |  |  |
| novel_mir_4988 | UUUUCUUUUAAUUGGACGAGAUA | 23 | 1 | 0 | m | 6 | 0 |  |  |
| novel_mir_4989 | UUUCUCCUGAUUUUUAGCAUUUUU | 24 | 3 | 0 | m | 5 | 0 |  |  |
| novel_mir_4990 | UUUGUAUGUUAGAUCGAAGAG | 21 | 1 | 0 | - | 16 | 8 |  |  |
| novel_mir_4991 | ACACUGUGGAAGUGGAUCUCUCUC | 24 | 1 | 0 | 0 | 10 | 0 |  |  |
| novel_mir_4998 | UUAGAAAGGGACCAUGGAUGAUGU | 24 | 1 | - | - | 19 | - |  |  |
| novel_mir_4999 | ACAAGCGAGUUAAGGAUGUGACAA | 24 | 1 | - | m | 15 | 8 |  |  |
| novel_mir_50 | UCCAUAUUUCACUAUCUCUUA | 21 | 1 | - | 176 | 85 | - | Y |  |
| novel_mir_5000 | AGAAUGACUGGUUUACUCUUU | 21 | 1 | m | m | 5 | 0 |  |  |
| novel_mir_5004 | AGAGGAUGCUUUAUAAAACUCAUA | 24 | 1 | m | - | 7 | m |  |  |
| novel_mir_5016 | ACCGAUUUGCUCUUUGAUCUA | 21 | 1 | 0 | 0 | 13 | 0 |  |  |
| novel_mir_5018 | CCUGACUAGAGACAAUACCAACCU | 24 | 1 | m | 0 | 5 | m |  |  |
| novel_mir_5019 | AUUAUGUGUAUGAAACUUUAGUU | 23 | 1 | 0 | 0 | 9 | 0 |  |  |
| novel_mir_5023 | UAACUAAAUAGUGACACGUGGCAU | 24 | 1 | - | - | 6 | m |  |  |
| novel_mir_5031 | CUAGUUUGCUCUUUGAUCUAAUGU | 24 | 1 | m | m | 5 | 0 |  |  |
| novel_mir_5039 | UCAUGUAUAAUUUUGAGAUUUGUC | 24 | 1 | 0 | m | 8 | 0 |  |  |
| novel_mir_5040 | UCUUAGUUGGCAUAUACUCAAGGA | 24 | 1 | - | m | 7 | 8 |  |  |
| novel_mir_5042**^e^** | AACUCUCUCCCUCAAAGGCUA | 21 | 1 | m | - | 11 | 6 | Y |  |
| novel_mir_5045 | AGACGACACUUCAAAGAUUUCUCC | 24 | 1 | 0 | - | 5 | m |  |  |
| novel_mir_5046**^e^** | UGGGCUUCUUGCAAGAUGAAGGUA | 24 | 1 | m | m | 5 | m |  |  |
| novel_mir_5047 | AGAGAAUGUCAGUACUAGAGGCAG | 24 | 1 | m | m | 6 | m |  |  |
| novel_mir_5062 | AGUUUUAGGAUUGAUUUGAUGAAA | 24 | 2 | m | m | 6 | 0 |  |  |
| novel_mir_5106**^e^** | CGGGCUUGGGCAAAAUUUUAGGCU | 24 | 1 | m | m | 5 | m |  |  |
| novel_mir_5142 | AAGAGAAAAAAUCGGAUUUAUCAU | 24 | 1 | 0 | 0 | 5 | 0 |  |  |
| novel_mir_5145 | UUUCCAUAUUAGGGUUUGAACUUU | 24 | 1 | 0 | m | 8 | 0 |  |  |
| novel_mir_5150 | UUUGAUCUAAUGUAUGGAGACUA | 23 | 1 | m | 0 | 6 | m |  |  |
| novel_mir_5170 | CCAAAGUGAUUGGCGGCACCA | 21 | 1 | m | m | 6 | m |  |  |
| novel_mir_5179 | UUGGAUUUUGAUUCAUAGAUUCGU | 24 | 1 | m | m | 7 | m |  |  |
| novel_mir_5235 | GAGUCUUGUAGGUACCACCAAU | 22 | 1 | 0 | 0 | 13 | 0 |  |  |
| novel_mir_539 | AGGGCGACAACGGUUACUGUGAUU | 24 | 1 | 63 | - | - | 27 |  |  |
| novel_mir_5668 | ACAUUGUUGAGGGUCUAAUCGG | 22 | 1 | 0 | m | 0 | 174 |  |  |
| novel_mir_5669 | UUUCAGCGCAGUAGAAGGAUU | 21 | 1 | 0 | 0 | 0 | 12 |  |  |
| novel_mir_5671 | UUUCCAAUAGUUUUAUGCCACAUC | 24 | 1 | 0 | 0 | 0 | 5 |  |  |
| novel_mir_5687 | GGAUUGUCGUUAGGGAGGUAA | 21 | 2 | - | - | - | 359 |  |  |
| novel_mir_5690 | UUGUGAGAUUGAAGCUGAUGG | 21 | 1 | - | - | - | 8 |  |  |
| novel_mir_5694 | AUUUAAUGUAUAGGGACUAAU | 21 | 1 | 0 | m | 0 | 6 |  |  |
| novel_mir_5708 | AUUUUGGAAGAAUUUCAGCUG | 21 | 1 | - | - | - | 397 |  |  |
| novel_mir_5712 | GGCAUUUAAAACACAUUUGGACUG | 24 | 1 | - | m | - | 14 |  |  |
| novel_mir_5716 | AGGAGGAAUAAGUCUGAUUUGUCA | 24 | 1 | m | - | 0 | 8 |  |  |
| novel_mir_5754 | AGGGCCAUUUUGACAAAACAUGCA | 24 | 1 | - | - | - | 6 |  |  |
| novel_mir_5769 | UUUGUACGUGGCGGGAGAUAU | 21 | 1 | m | - | 0 | 6 |  |  |
| novel_mir_5776 | UGUGACAUCGUCAAAUUCGGCCAU | 24 | 1 | m | 0 | 0 | 8 |  |  |
| novel_mir_5818 | CCAUUAACGGUGUAACAGUAAGCU | 24 | 1 | m | - | 0 | 6 |  |  |
| novel_mir_5867 | UUUGUCGACAUGUCAGGAAAGCGC | 24 | 1 | m | m | 0 | 6 |  |  |
| novel_mir_65 | UUUUUACUUUGGGACACUGAUGGC | 24 | 1 | 45 | 27 | 21 | 26 |  |  |
| novel_mir_67 | CUAUAGAACAUGACCUUUAGCAGC | 24 | 2 | - | 31 | 18 | 20 |  | L |
| novel_mir_68 | UUAGAAAUGGACCAUGGAUGAUGU | 24 | 1 | 12 | - | 11 | 11 |  |  |
| novel_mir_69 | UUUCCUAUGCCCCCCAUUCCAC | 22 | 1 | - | 219 | 125 | 84 | Y |  |
| novel_mir_70 | AGAGUGACUACUUCGUAACAAAAC | 24 | 1 | 13 | 29 | - | 9 |  |  |
| novel_mir_76 | UUAAUGGAUCAUAACAGCAGGUAU | 24 | 1 | 5 | 10 | 7 | 9 |  |  |
| novel_mir_819 | AGUGGAAGGACAAAAUGCAAUCUG | 24 | 1 | 10 | 5 | 8 | 5 |  |  |
| novel_mir_823 | UUUGAUGAUGUGGCAACAAAGCGC | 24 | 1 | 8 | m | 0 | m |  |  |
| novel_mir_824 | UAAACAUAAGUAGAAUUAAACAAG | 24 | 9 | 14 | 78 | 37 | 11 |  |  |
| novel_mir_830 | AGGUAUUGUCUCUGGGGAAGGGUU | 24 | 1 | - | 24 | - | 13 |  |  |
| novel_mir_832 | GAGCUUGGAAGUGCAUCCGGC | 21 | 1 | - | - | 27 | 19 | Y |  |
| novel_mir_84 | UUCCAUGUCACAGAGAUGUUG | 21 | 1 | m | 11 | 0 | m |  |  |
| novel_mir_841 | AGAGGUGCUCAUGGGCUGGGUCGG | 24 | 1 | m | m | 0 | 8 |  |  |
| novel_mir_844 | AUACAUGAACUUUGGUCCAA | 20 | 2 | 7 | m | 9 | m |  |  |
| novel_mir_848 | UUUGUUUAUGGUCAUCUAAGC | 21 | 1 | m | 8 | 0 | m |  |  |
| novel_mir_849 | CAUCGAUAGUUUGAGGAUGUA | 21 | 2 | 43 | - | 53 | 27 |  |  |
| novel_mir_860 | UGAUGUGGCAUUAAACUAUUGAAA | 24 | 2 | 8 | 0 | 0 | m |  |  |
| novel_mir_862 | UUGGACUGUGGCUACAUAUAG | 21 | 1 | m | 11 | 5 | 5 |  |  |
| novel_mir_884 | AGGUGCAGAUGCAGUUGCAGG | 21 | 1 | 0 | 5 | 0 | m |  |  |
| novel_mir_885 | UUUCAAAGUCCUUGCAUACUAUUU | 24 | 4 | 0 | 22 | 6 | m |  |  |
| novel_mir_886 | UUAAACUCCAGAAGUAGGGCU | 21 | 1 | 16 | 17 | 18 | 12 |  |  |
| novel_mir_887 | AAGGAUACUACUUUGAUGGAGAAA | 24 | 4 | 6 | 7 | 0 | 0 |  |  |
| novel_mir_890 | UGAAUACAGGAAUGGCUCUCU | 21 | 1 | 17 | 79 | 81 | 56 |  |  |
| novel_mir_892 | UGGGUCGAGCUUAGGCAAGCAUA | 23 | 1 | 22 | 10 | 10 | m |  |  |
| novel_mir_90 | UGAUGGAGAUAGGUAUCUGCA | 21 | 1 | 9 | 9 | 21 | m |  |  |
| novel_mir_905 | UUGAUGGUGGUAAGAAAUGUGCAU | 24 | 1 | - | - | 17 | 19 |  |  |
| novel_mir_92 | UAGCACUGAAGAUGAUGAUGG | 21 | 1 | - | - | 342 | - |  |  |
| novel_mir_925 | CAAAUUGGAUAACUGGACAGGUAA | 24 | 1 | 12 | m | 16 | 13 |  | L |
| novel_mir_928 | UGUUAGGCAGUCAGAGAAUGG | 21 | 1 | m | m | 9 | m |  |  |
| novel_mir_936 | UAUGAAAAGUUAUAAAAUGGUCAU | 24 | 1 | 0 | 7 | 6 | m |  |  |
| novel_mir_941 | AAGCGCCUCUAAAGAACAUGGUCU | 24 | 1 | 6 | 15 | 0 | m |  |  |
| novel_mir_949 | AAGGUGAUGACCUGCUACAAUUUU | 24 | 1 | m | 7 | 0 | 0 |  |  |
| novel_mir_95 | UCAGAUGAAGCUGCCAGCAUGA | 22 | 1 | 31 | - | 46 | 24 | Y |  |
| novel_mir_950 | UGAUCAAAACAGGAACGAAUUCAA | 24 | 1 | 17 | 28 | 22 | 9 |  |  |
| novel_mir_960 | UCAUUUUGAACAAAACGACGUCGU | 24 | 1 | 37 | - | 24 | 27 |  |  |
| novel_mir_968 | UUUAACCGUAGAAAUGGAUGA | 21 | 2 | m | 13 | 8 | m |  |  |
| novel_mir_974 | GCGGCAUUAUCAAGAUUCACA | 21 | 1 | 38 | 69 | 73 | 97 |  |  |
| novel_mir_977 | GAAGAGUAUAGGGACUUAUGGCAU | 24 | 1 | 13 | m | - | 23 |  |  |
| novel_mir_986 | CGAAGUCUUGGAAGAGAGUAA | 21 | 1 | 145 | 392 | 272 | - | Y | L |
| novel_mir_987 | UUCAACUCUGCCAAGCAAUUG | 21 | 2 | m | 36 | 26 | - |  |  |
| novel_mir_995 | GGGGACAAUACCUUCGAUUGUUGG | 24 | 1 | - | 8 | - | - |  |  |
| novel_mir_999 | AAGGUUGAUGGUUAAAUUUGACU | 23 | 1 | m | - | 11 | m |  |  |

a: ”-” indicates sequences for which the set of reads of candidate miRNA loci accounts for less than 95% of all the precursor-mapped small RNA reads, i.e., small RNAs that are not considered miRNAs because they did not meet the precise excision criteria for miRNA prediction; ”m” indicate small RNAs with abundances too low (less than 5 reads) to be predicted as candidate miRNAs; red highlighting indicates the small RNAs with reads accounting for less than 75% of the corresponding set of reads, which are excluded because they could not meet the precise excision criteria for miRNA prediction.

b^:^ Y indicates the precise miRNA* sequence (two-nucleotide 3’ overhangs).

c: Novel miRNAs identified by Li et al. [1].

d: Novel miRNAs that may be expressed specifically in the cotton fiber.

**References:**

1. Li Q, Jin X, Zhu YX: **Identification and analyses of miRNA genes in allotetraploid Gossypium hirsutum fiber cells based on the sequenced diploid G. raimondii genome**. *J Genet Genomics* 2012, **39**(7):351-360.
