# Supplementary material for: Genome-wide analysis of small RNAs reveals eight fiber elongation-related and 257 novel microRNAs in elongating cotton fiber cells
Source: BMC Genomics. 2013 Sep 17;14:629. doi: 10.1186/1471-2164-14-629 (PMC3849097; doi:10.1186/1471-2164-14-629)
Supplement: Additional file 6: Figure S3 — The 310 stem-loop structures of novel miRNA precursors in cotton fibers. The stem-loop structure shows the miRNA* sequence, mismatches between the miRNA and the other arm of the hairpin, and the size and frequency of asymmetric bulges. There were 314 novel miRNA gene loci, as shown in Additional file 5. The two miRNA gene loci each for novel_mir_2455, GhmiRnJ, novel_mir_4037 and novel_mir_860 had the same precursor sequences, so there were 310 distinct stem-loop structures. [file 1471-2164-14-629-S6.pdf]

Additional Figure S3:

The 310 stem-loop stru ctures of n ovel miRNA precursors in cotton fibers. The stem-loop structure shows the miRNA* sequence, mismatches between the miRNA and the other arm of the hairpin, and the size and frequency of asymmetric bulges. There were 314 novel miRNA gene loci, as shown in additional file 5. The two miRNA gene loci each for novel_mir_2455, GhmiRnJ, novel_mir_4037 and novel_mir_860 had the same precursor sequences, so there were 310 distinct stem-loop structures.

Additional Figure S6:

310 stem-loop structures of novel miRNA precursors in cotton fibers. Stem-loop structure showed that miRNA* sequence, mismatch between the miRNA and the other arm of the hairpin, and size and frequency asymmetric bulges. There were a total of 314 novel miRNA gene loci as shown in the additional file 5. The two miRNA gene loci of the novel_mir_2455, GhmiRnJ, novel_mir_4037 and novel_mir_860 had the same precursor sequences, so there were 310 distinct stem-loop structures.

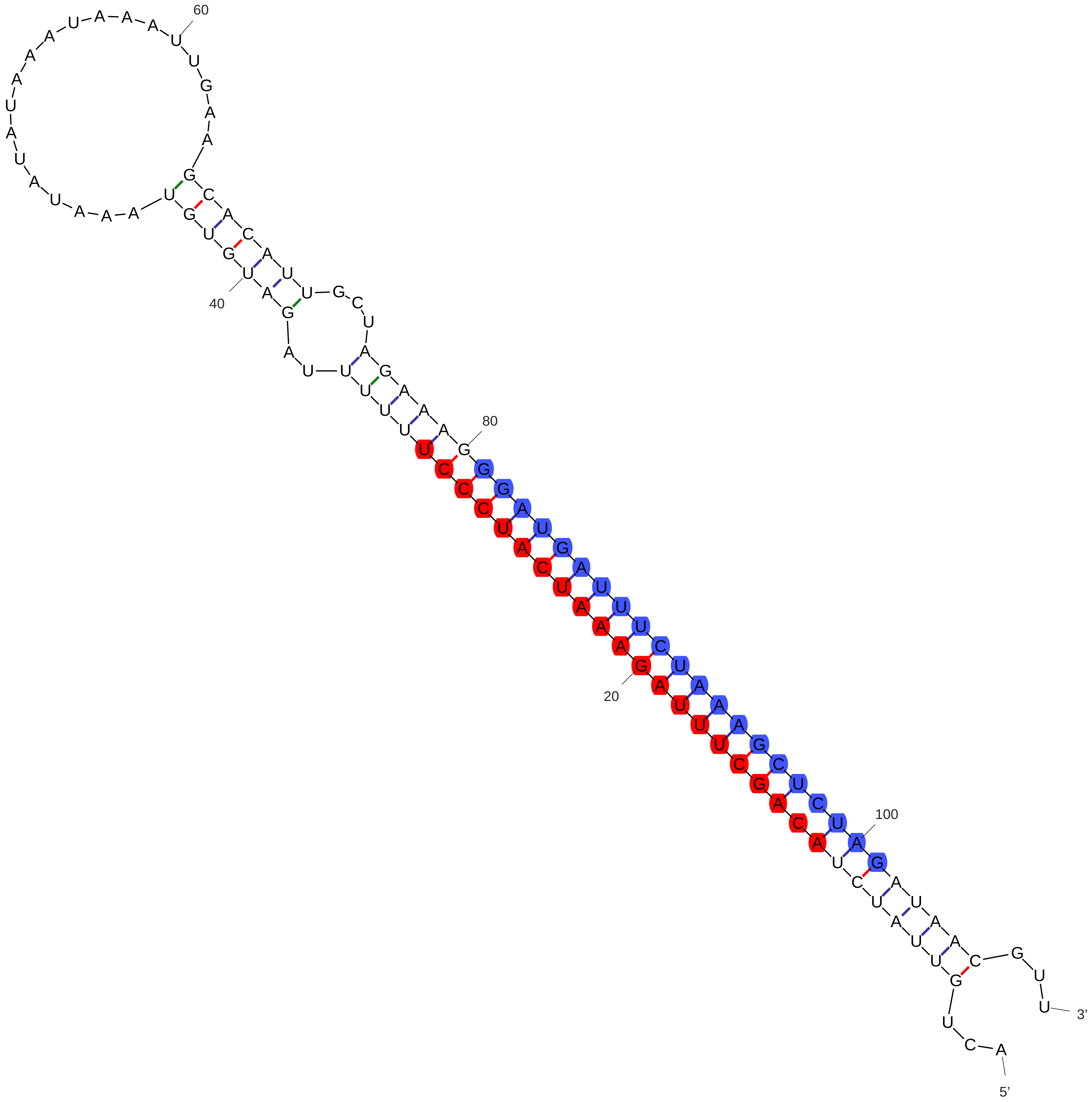

dG = -52.50 [Initially -52.50] GhmiRnA_1

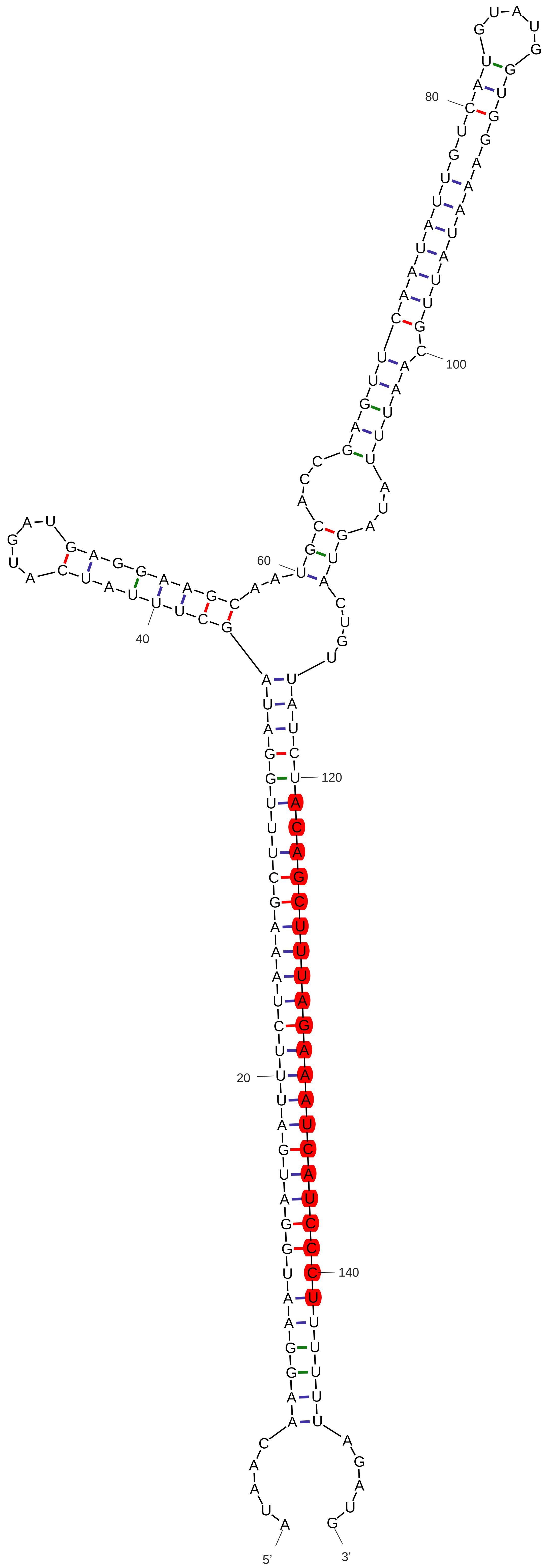

dG = -47.40 [Initially -50.00] GhmiRnA-2

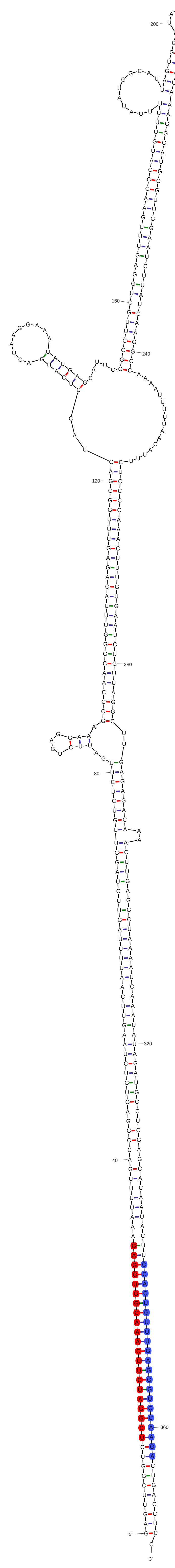

dG = -165.58 [Initially -173.90] GhmiRnJ_novel_mir_843

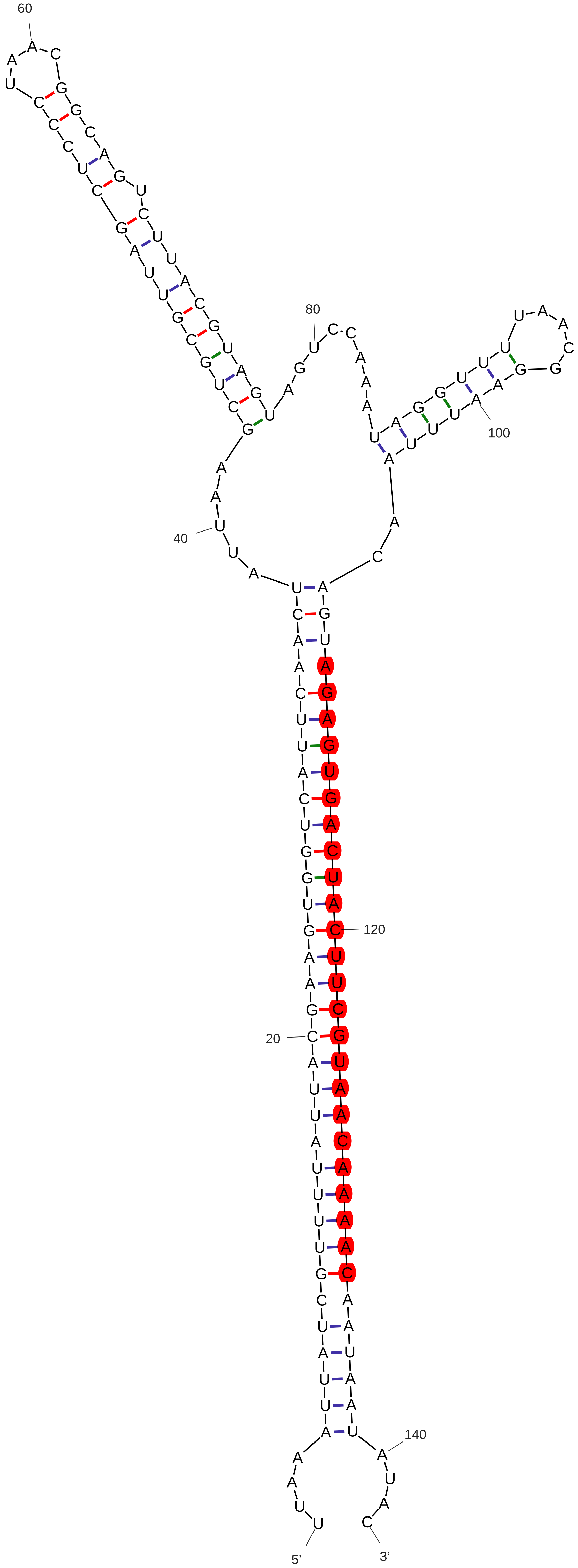

dG = -47.69 [Initially -51.50] novel_mir_70

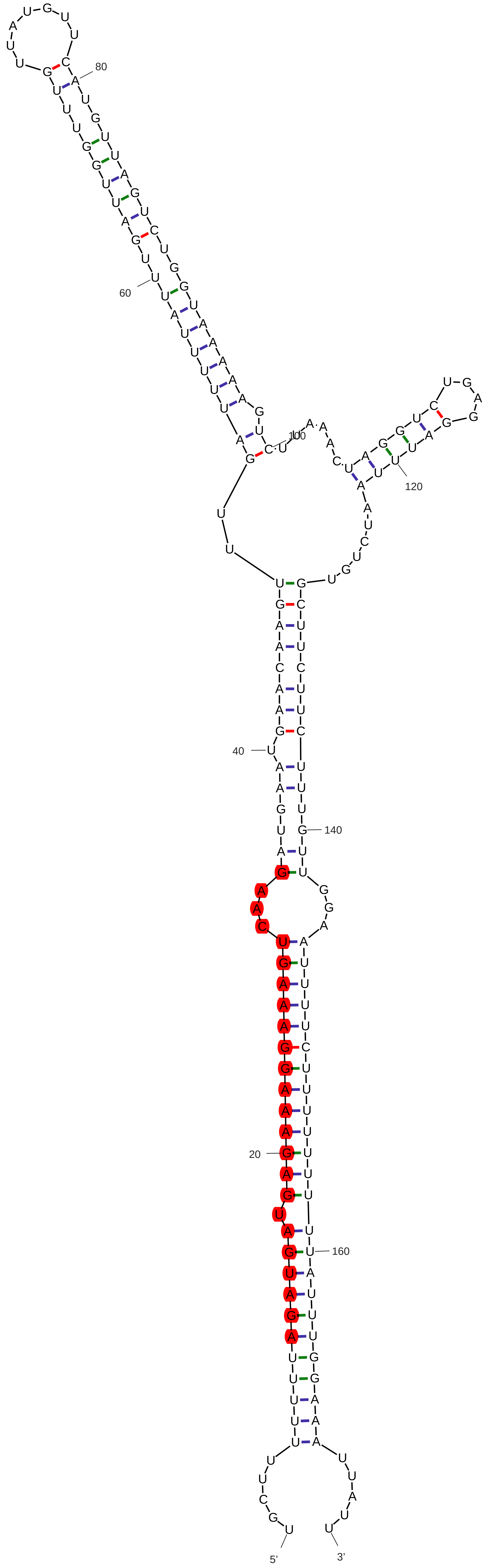

dG = -33.07 [Initially -36.20] novel_mir_1773

Output of sir_graph (©)
mfold_util 4.6

Created Sun Jan 13 08:06:05 2013

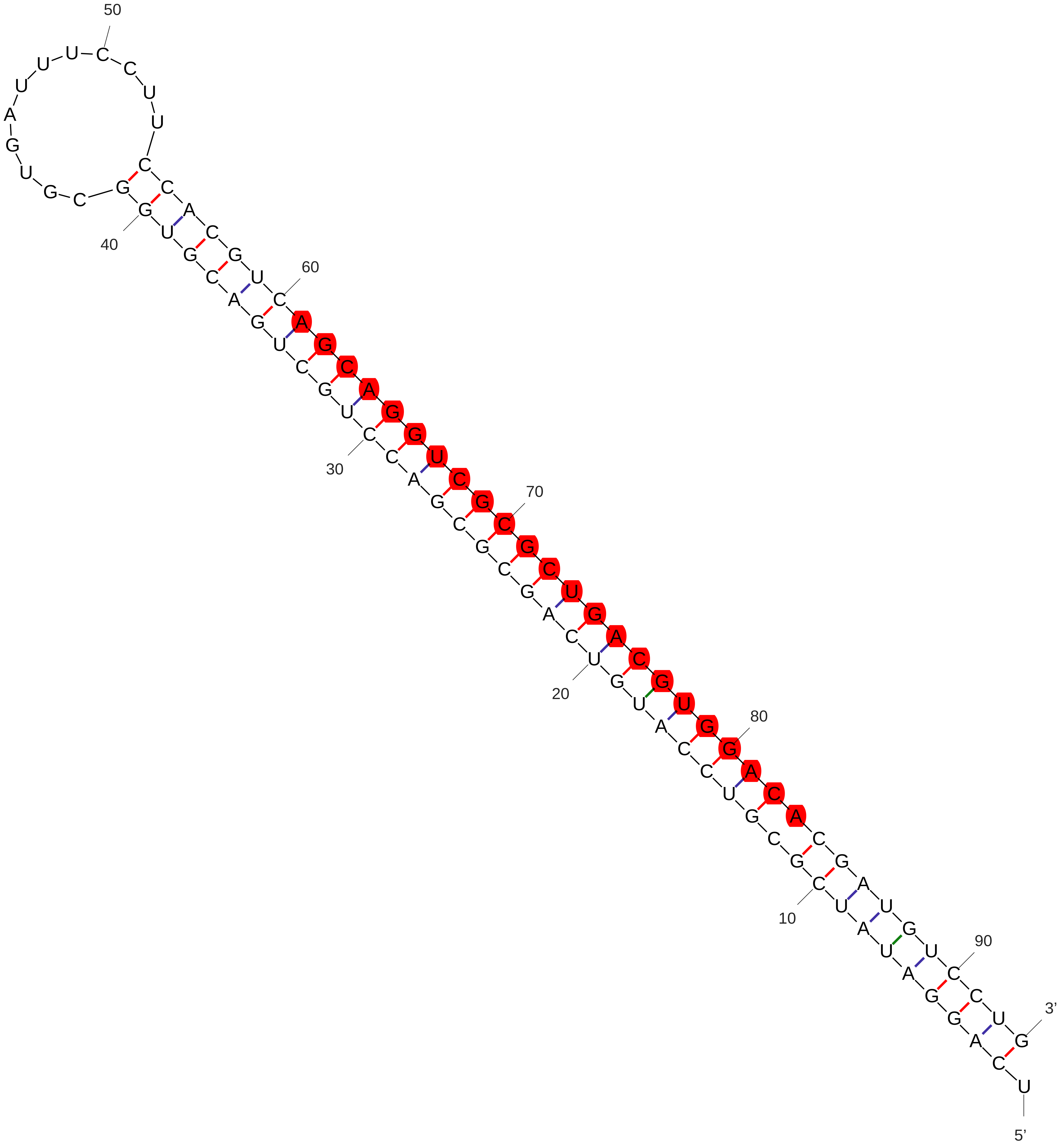

dG = -80.00 [Initially -80.00] novel_mir_4978

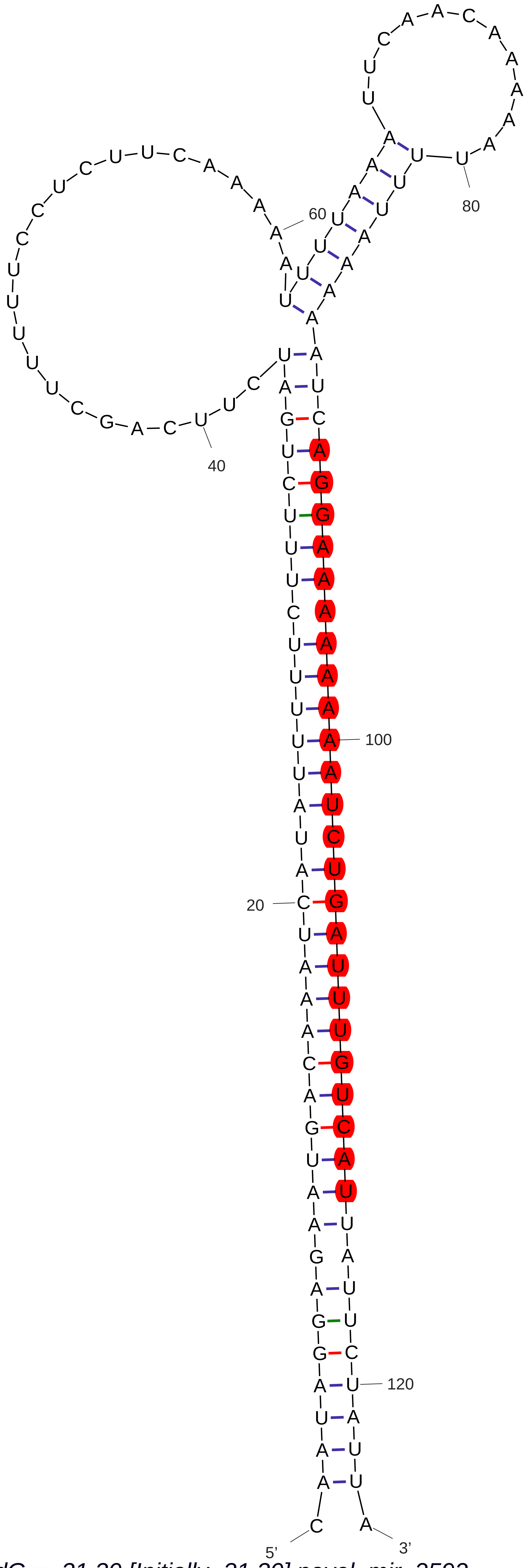

dG = -31.30 [Initially -31.30] novel_mir_2592

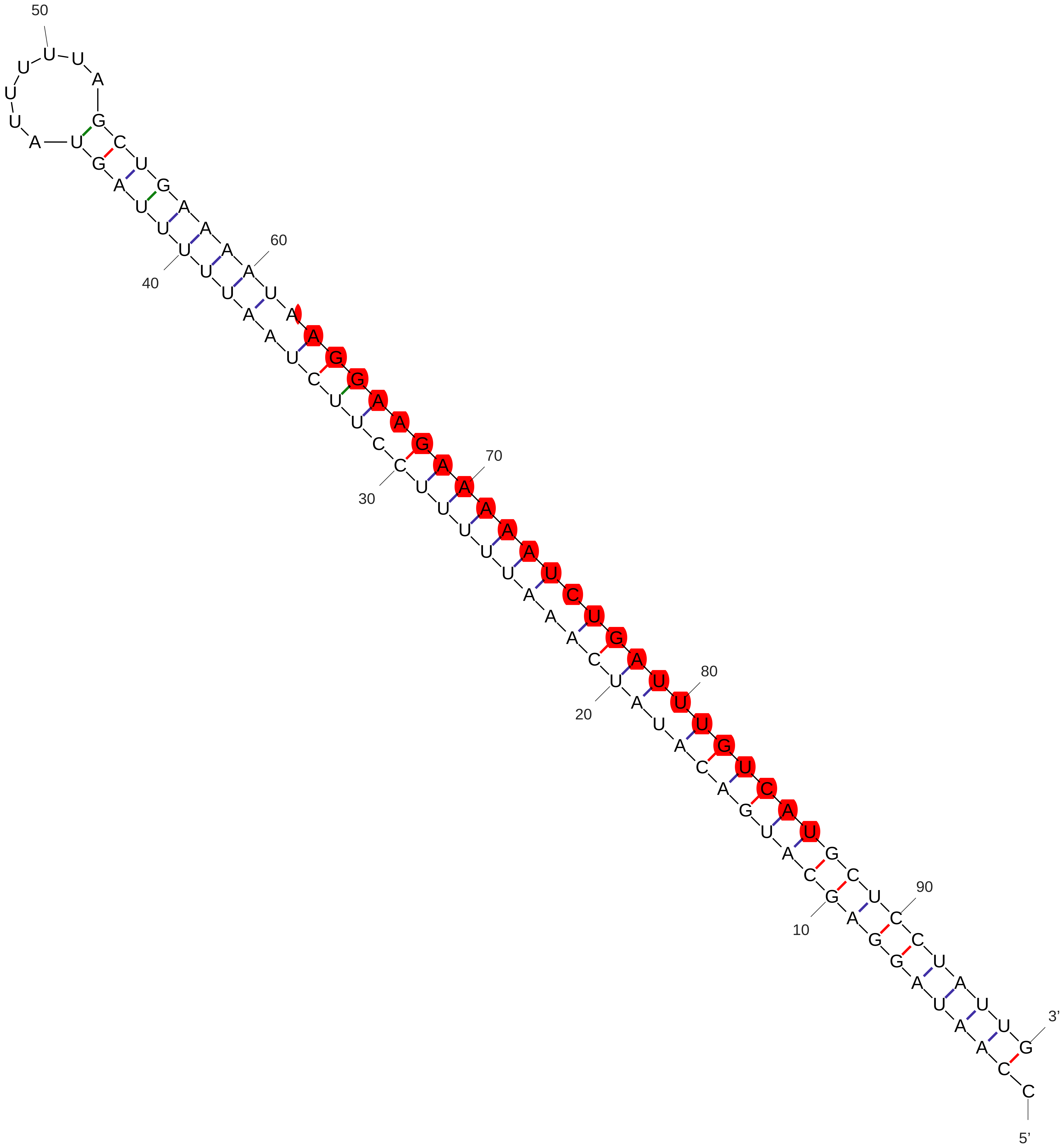

dG = -47.90 [Initially -47.90] novel_mir_5025_2

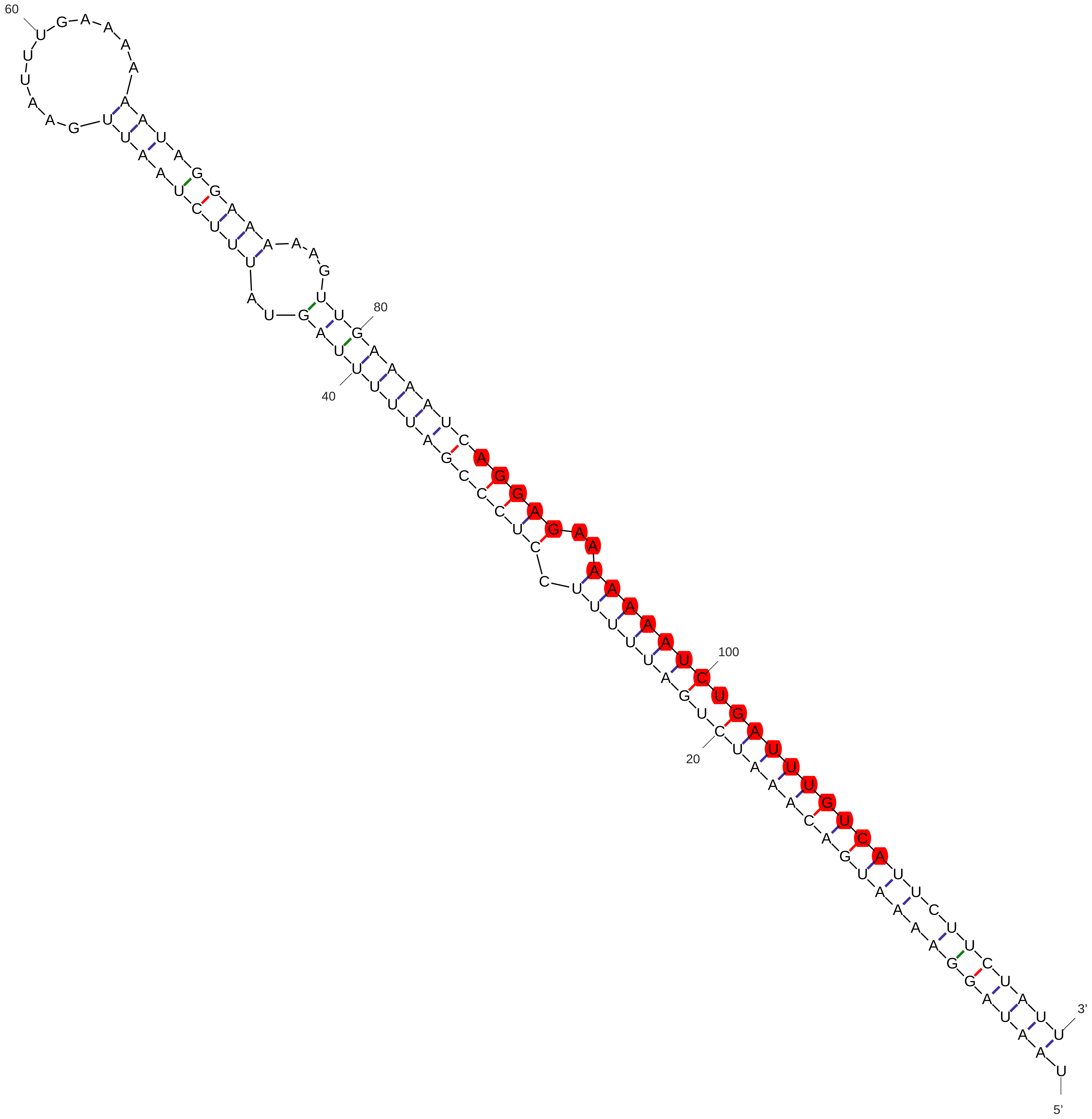

dG = -40.10 [Initially -40.10] novel_mir_1200_1

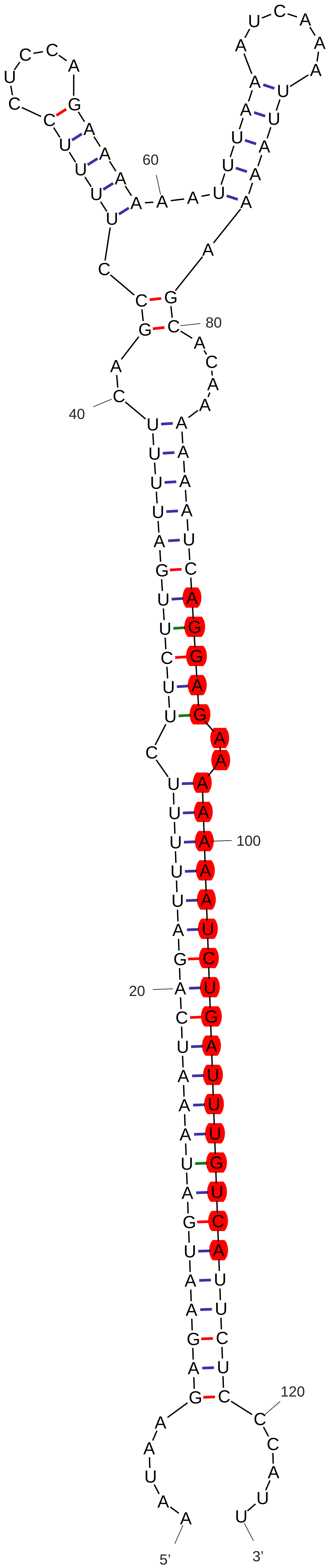

dG = -37.40 [Initially -39.80] novel_mir_1200_2

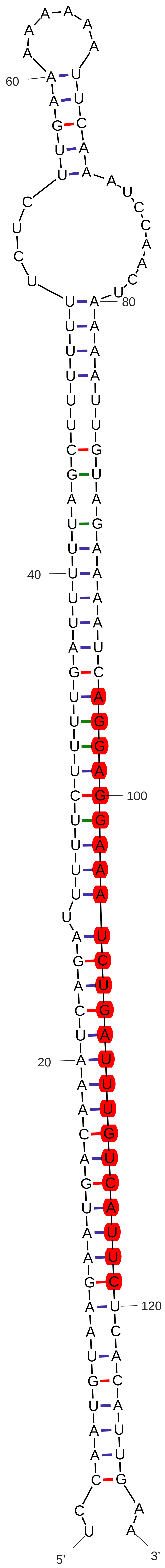

dG = -50.50 [Initially -50.50] novel_mir_1003

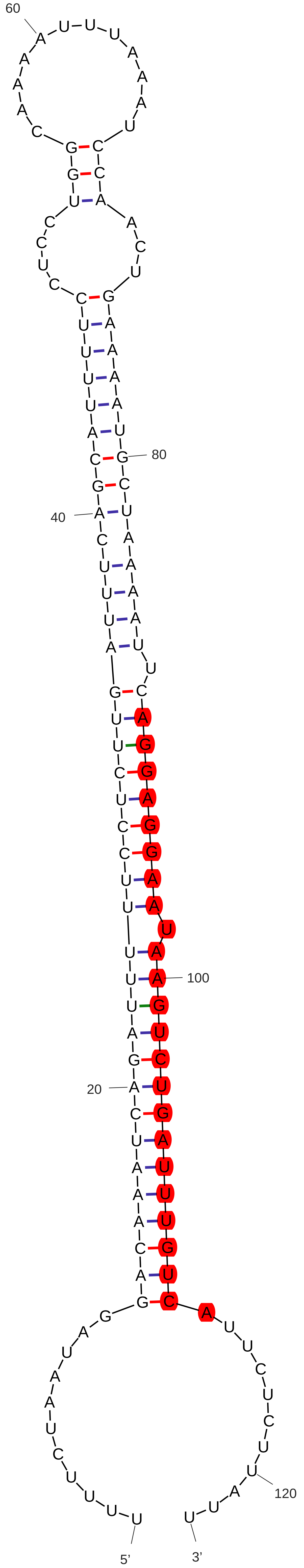

dG = -45.60 [Initially -45.60] novel_mir_5716

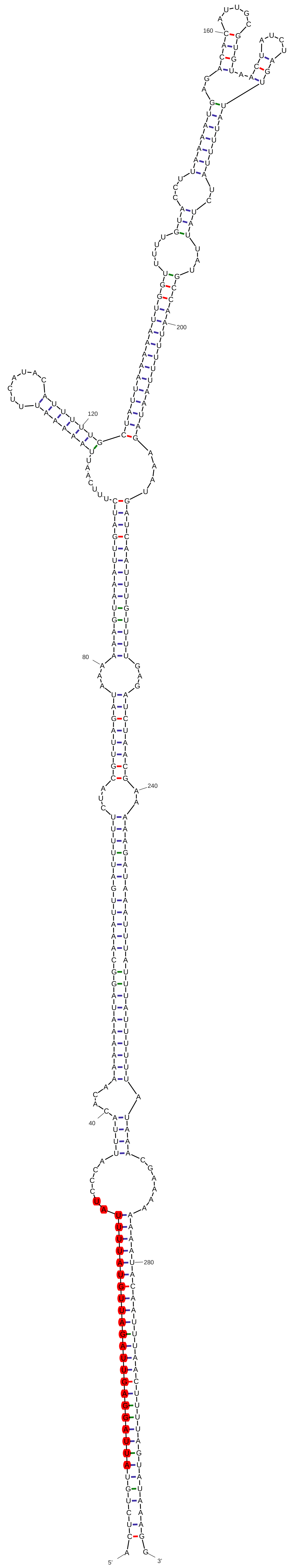

dG = -74.03 [Initially -79.00] novel_mir_4419

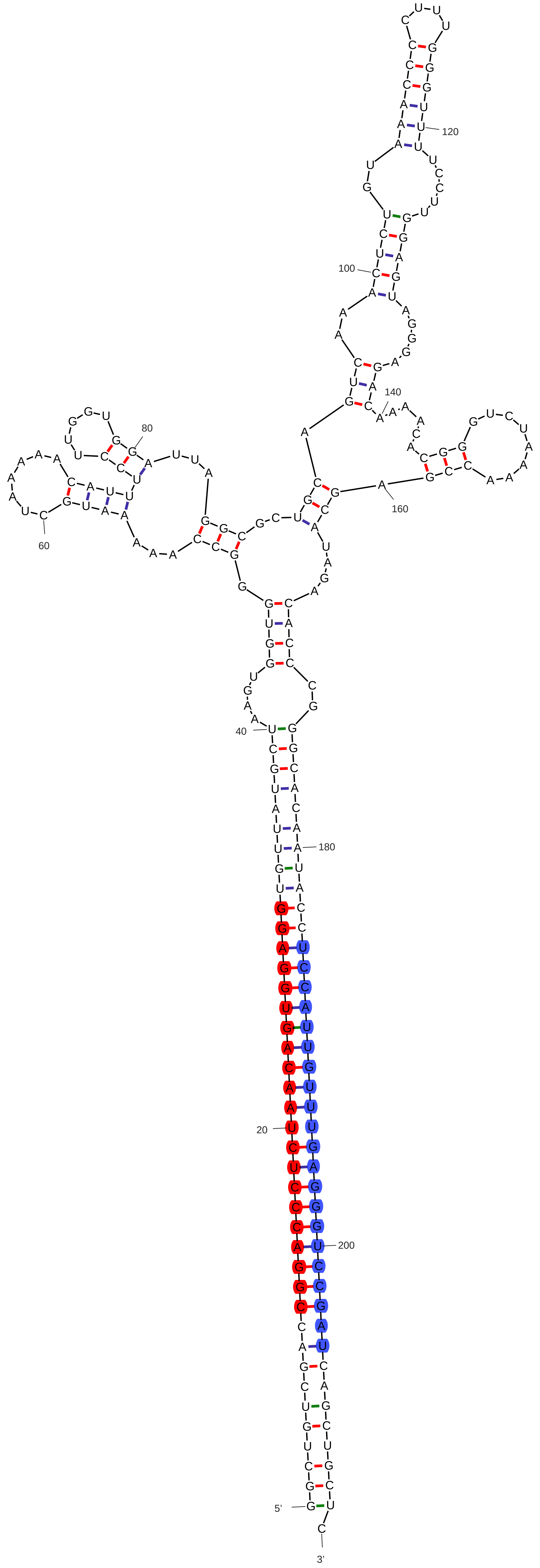

dG = -78.31 [Initially -87.70] GhmiRnK_novel_mir_839

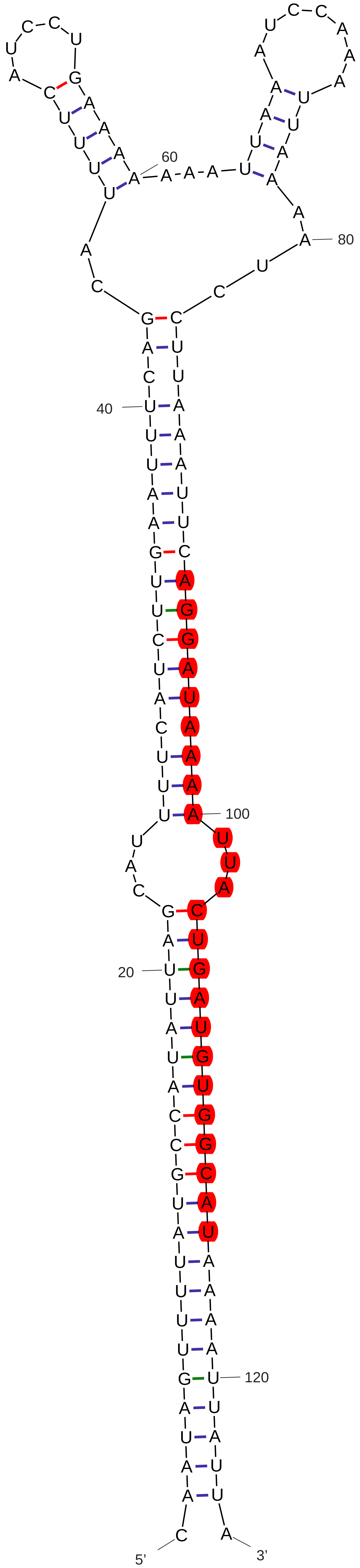

dG = -34.95 [Initially -38.80] novel_mir_133

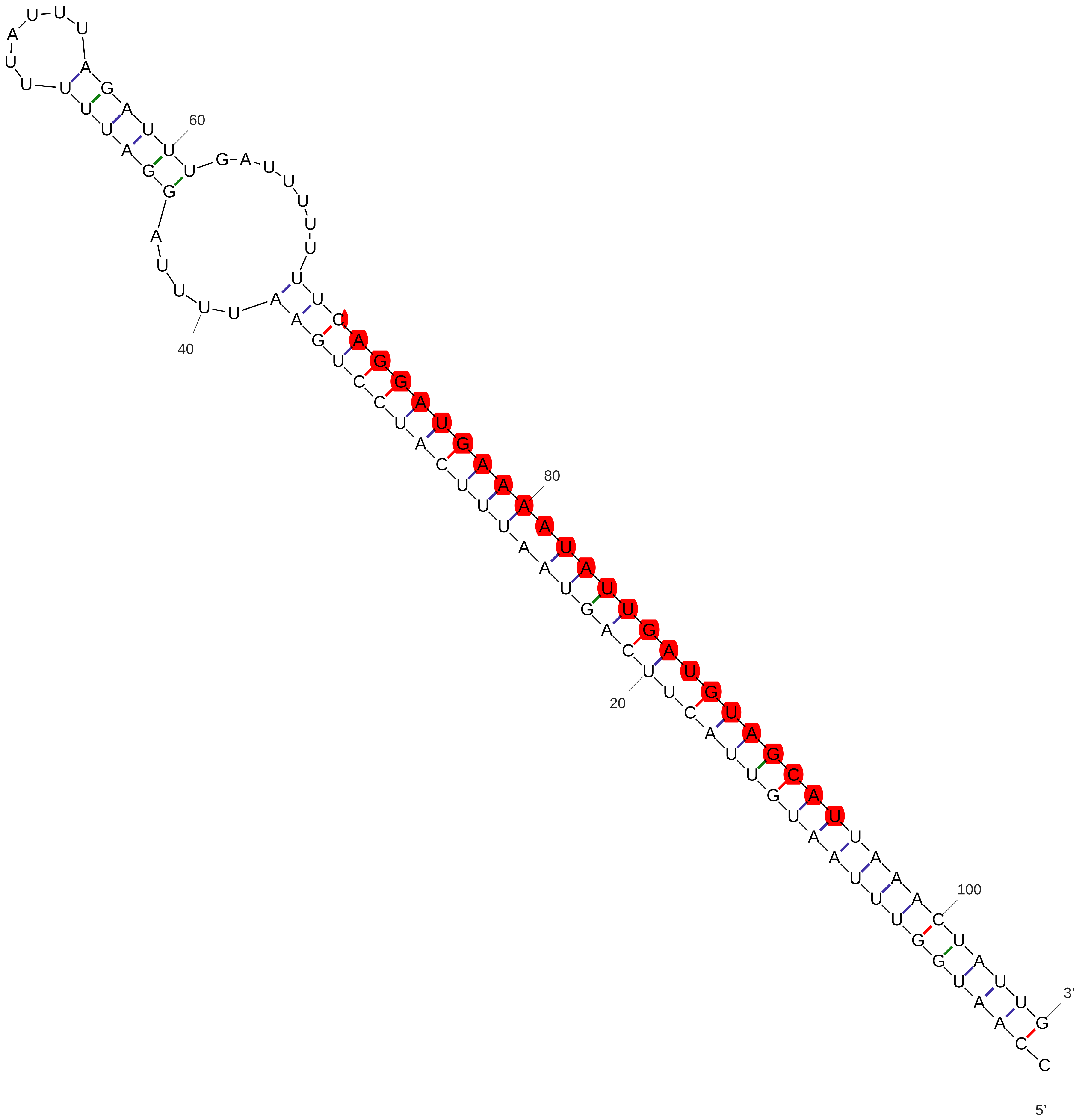

dG = -46.00 [Initially -46.00] novel_mir_4986

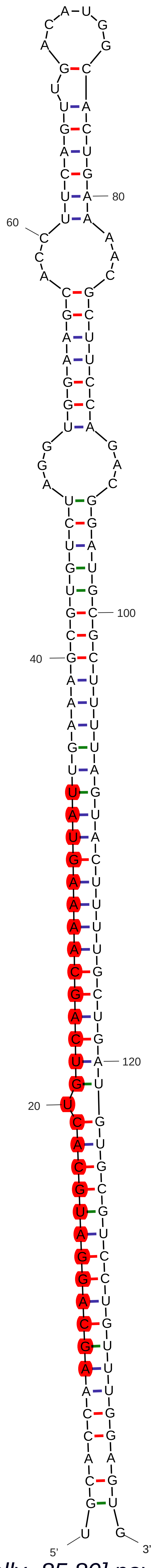

dG = -85.80 [Initially -85.80] novel_mir_4072

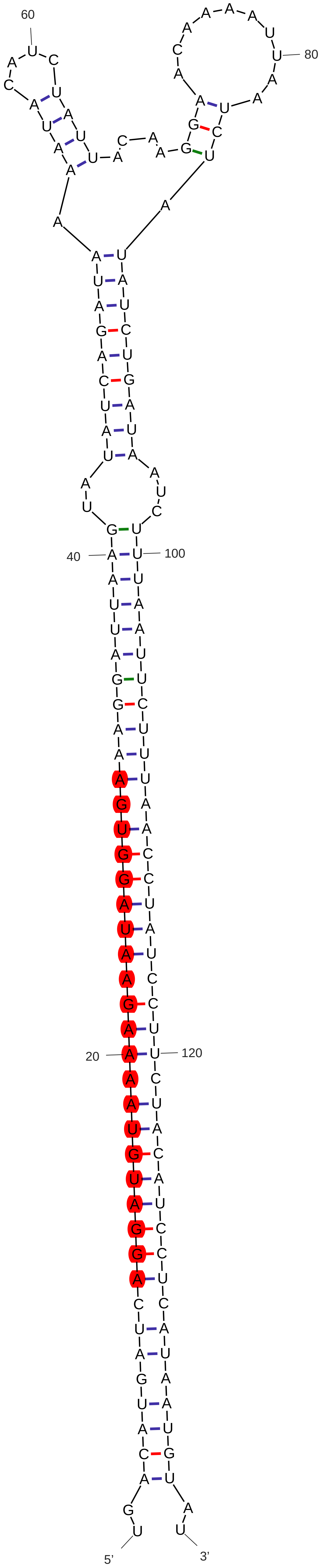

dG = -39.50 [Initially -40.90] novel_mir_4000

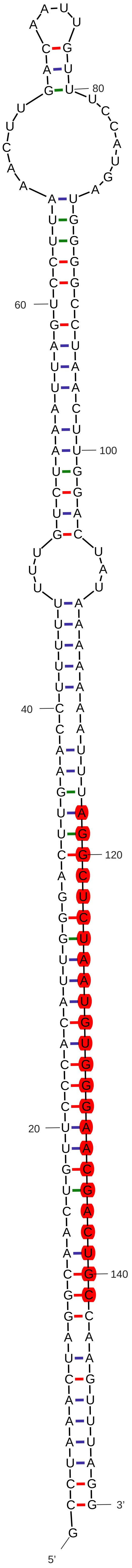

dG = -63.50 [Initially -63.50] novel_mir_2999

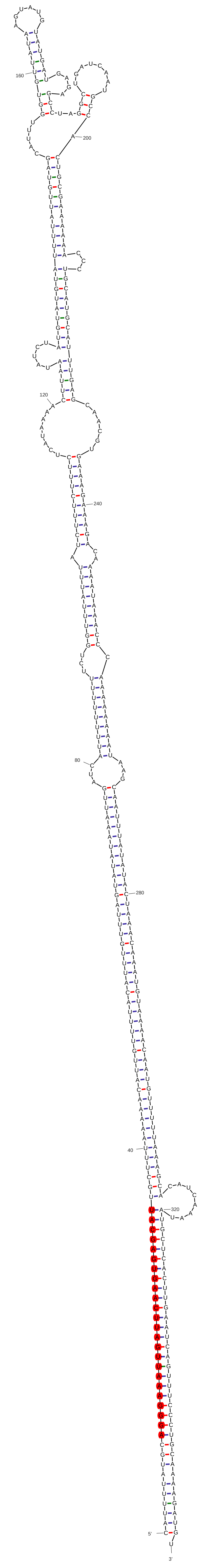

dG = -153.38 [Initially -156.00] novel_mir_2537

Output of sir_graph (©)
mfold_util 4.6

Created Sun Jan 13 08:12:16 2013

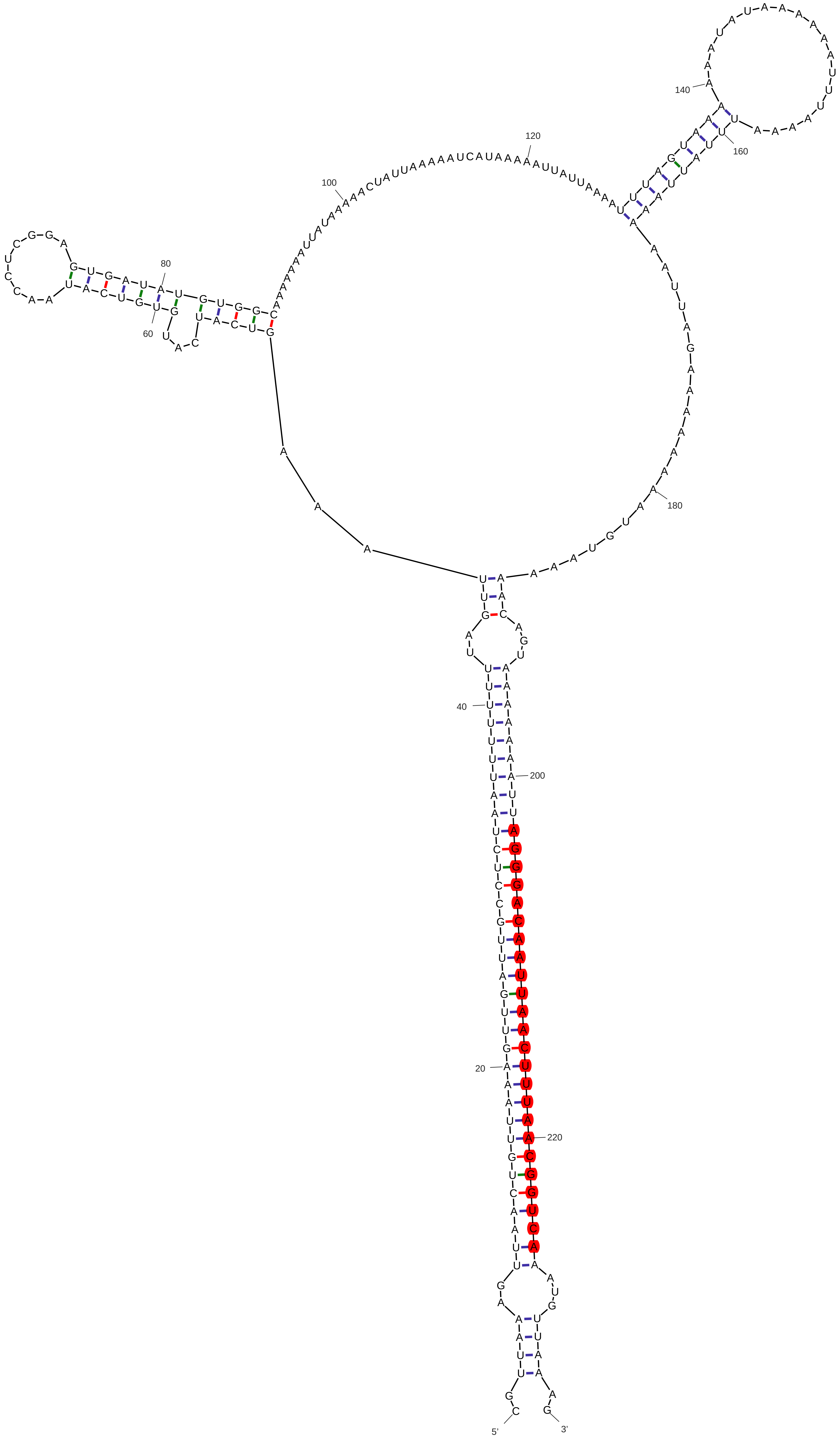

dG = -40.95 [Initially -46.40] novel_mir_2563

Output of sir_graph (©)
mfold_util 4.6

Created Sun Jan 13 08:12:25 2013

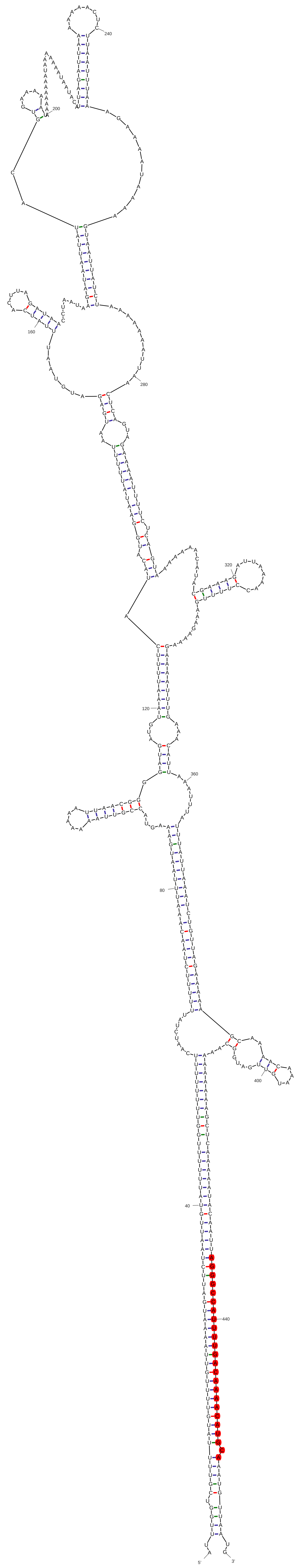

dG = -95.37 [Initially -112.70] novel_mir_5754

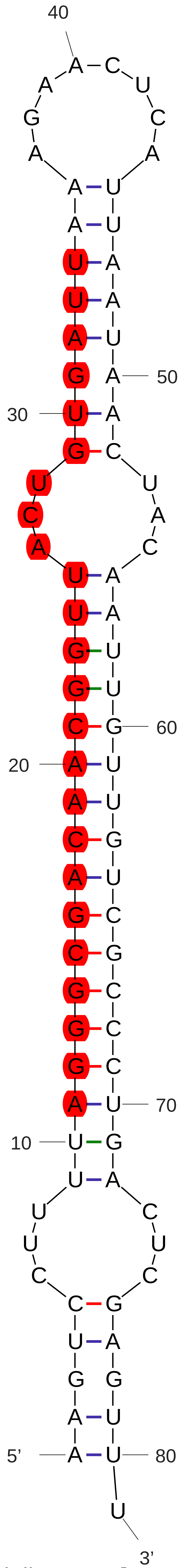

dG = -28.80 [Initially -28.80] novel_mir_539

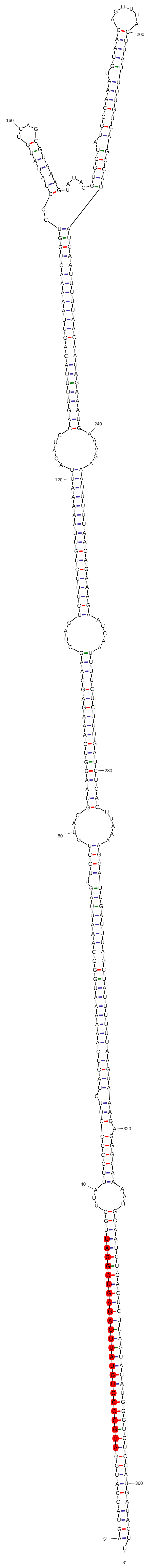

dG = -146.03 [Initially -147.80] novel_mir_43

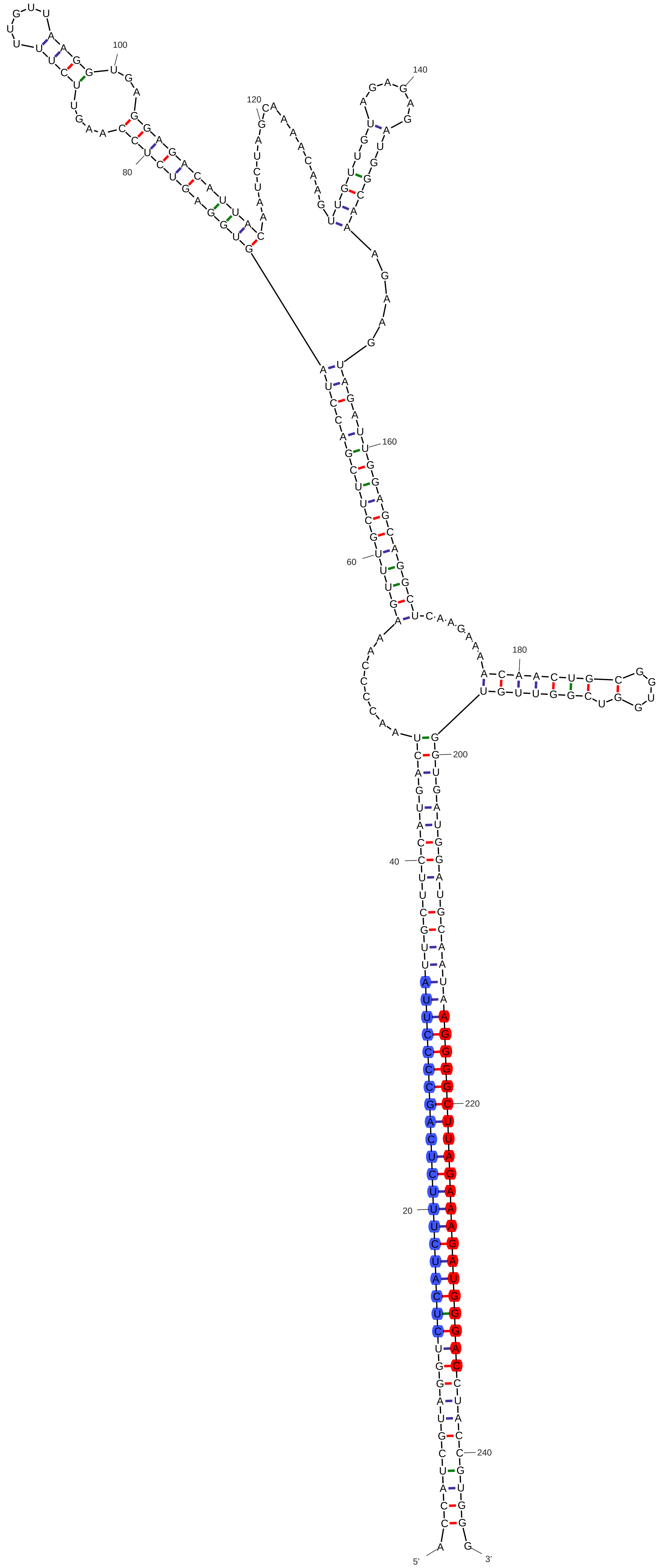

dG = -105.31 [Initially -112.00] novel_mir_2470

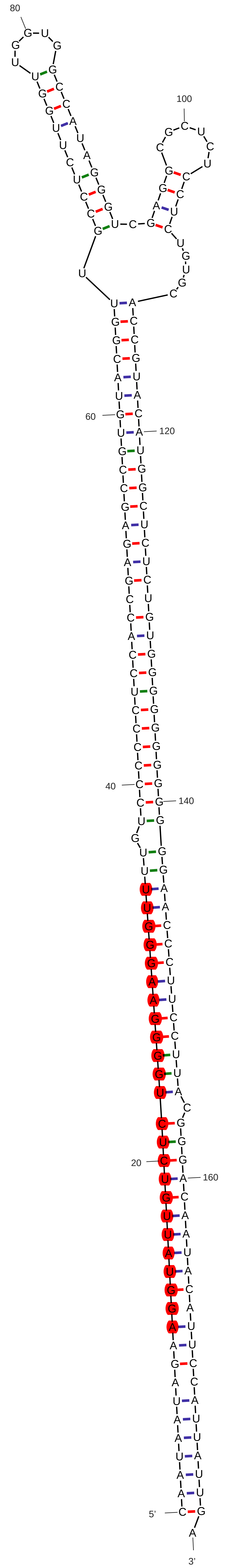

dG = -116.23 [Initially -116.10] novel_mir_830

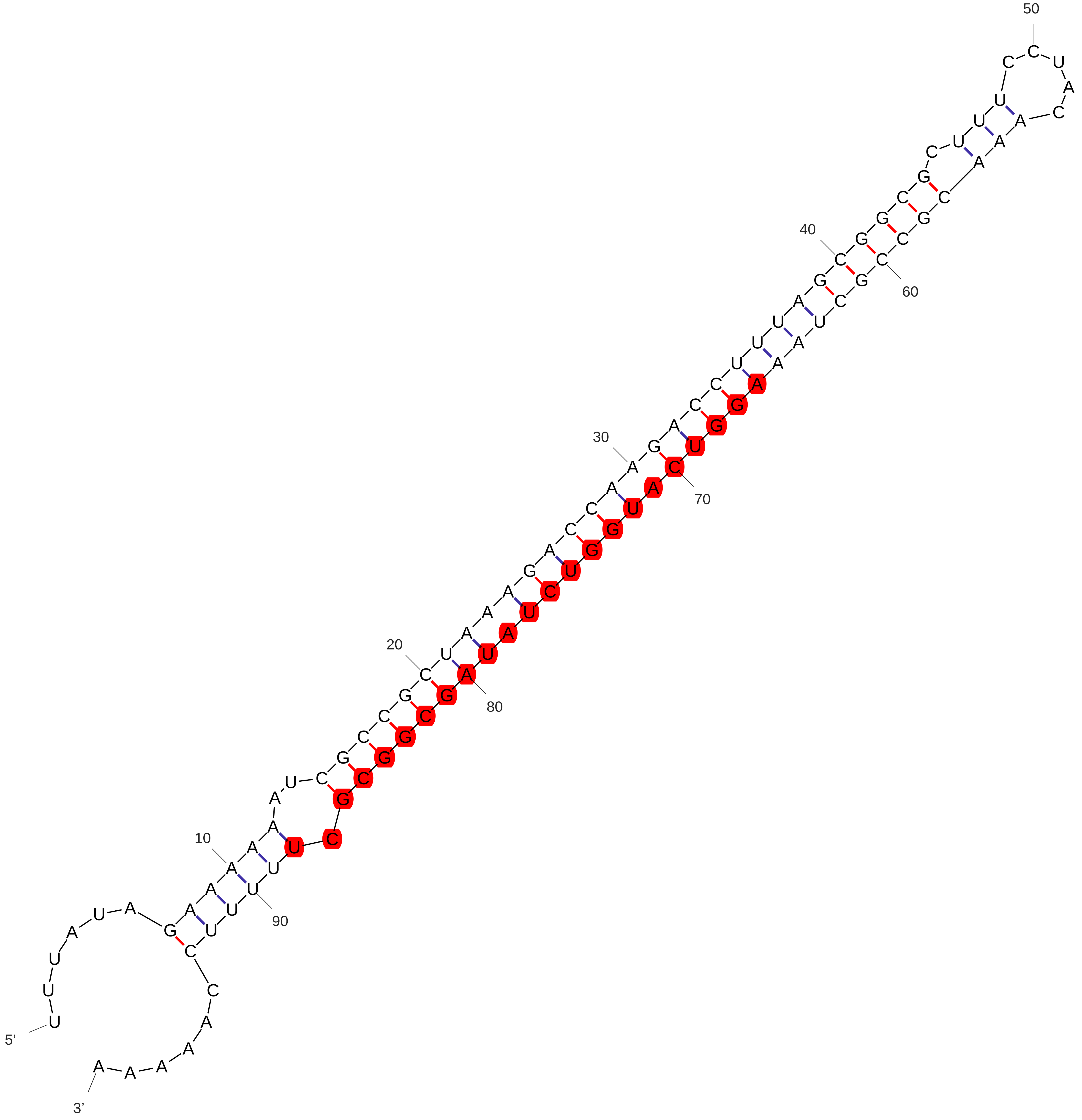

dG = -56.40 [Initially -56.40] novel_mir_2748

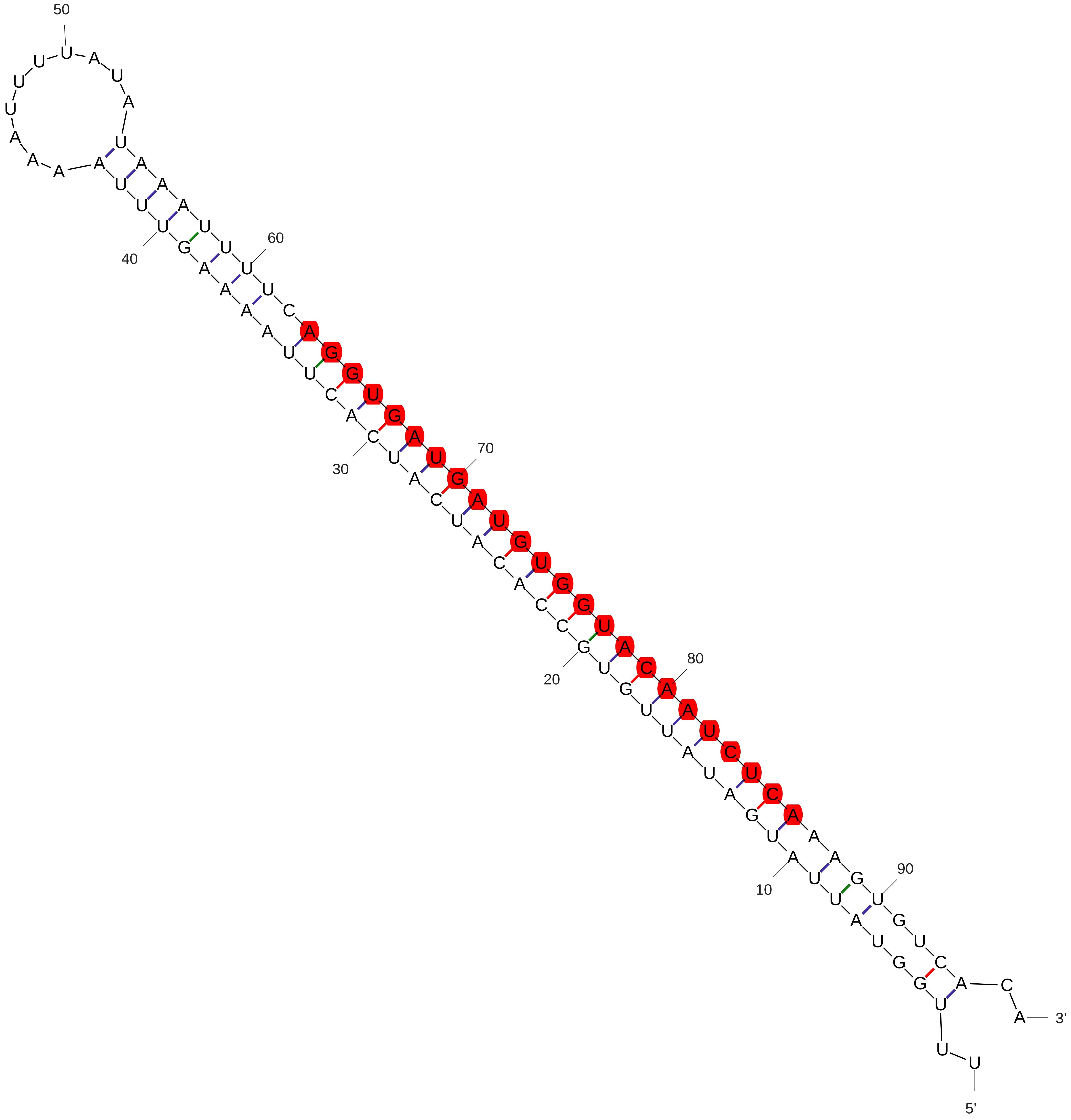

dG = -42.20 [Initially -42.20] novel_mir_2533

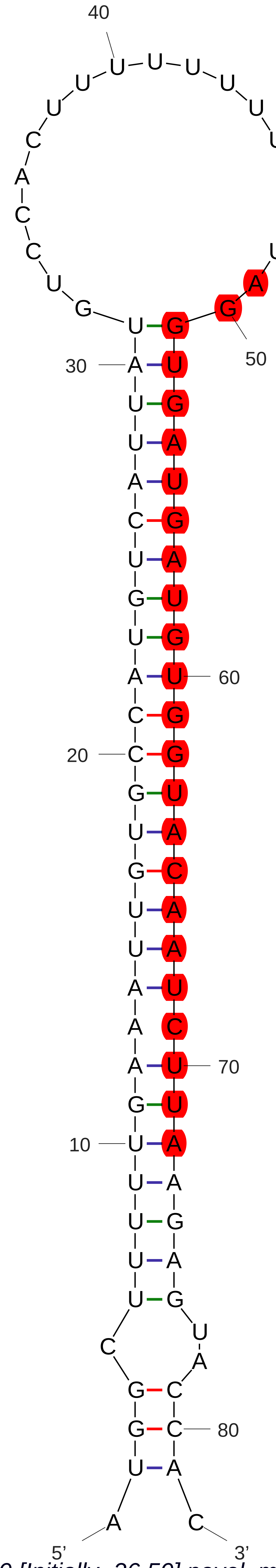

dG = -26.50 [Initially -26.50] novel_mir_3032

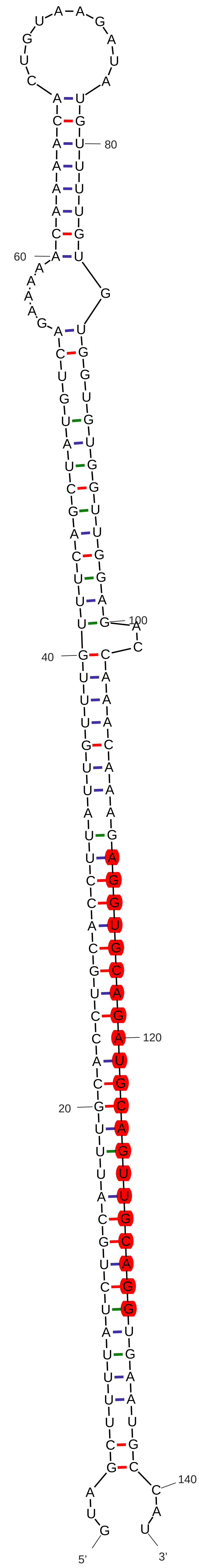

dG = -64.10 [Initially -64.10] novel_mir_884

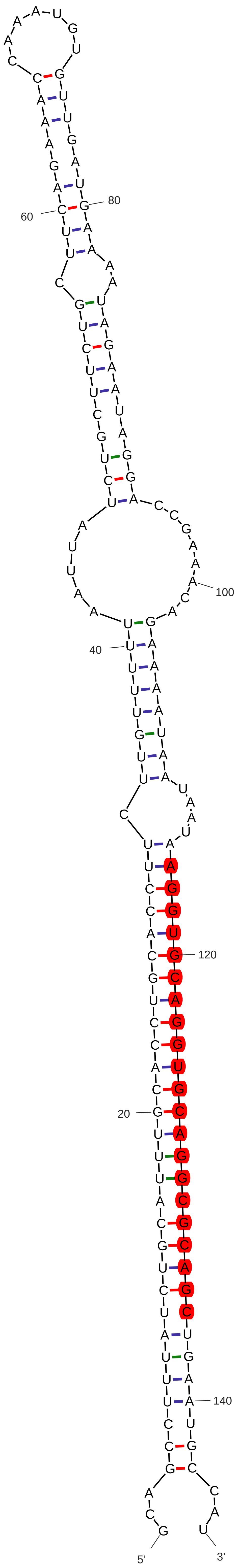

dG = -48.80 [Initially -48.80] novel_mir_2618

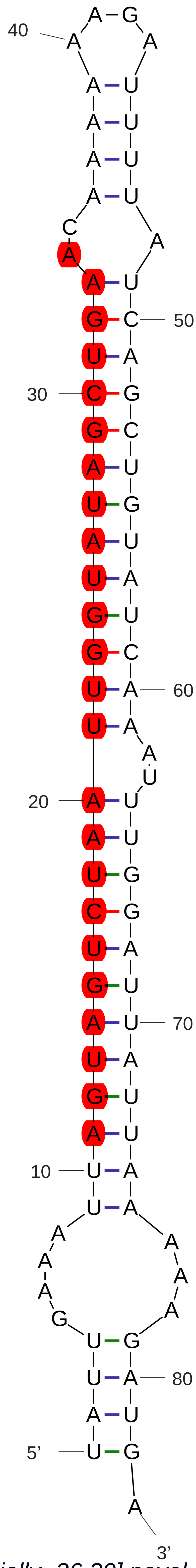

dG = -26.20 [Initially -26.20] novel_mir_111_1

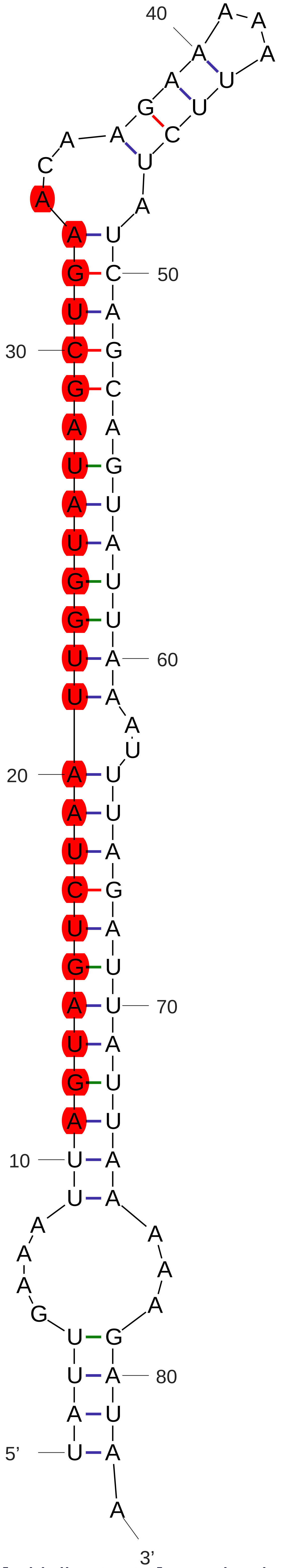

dG = -21.90 [Initially -21.90] novel_mir_111_2

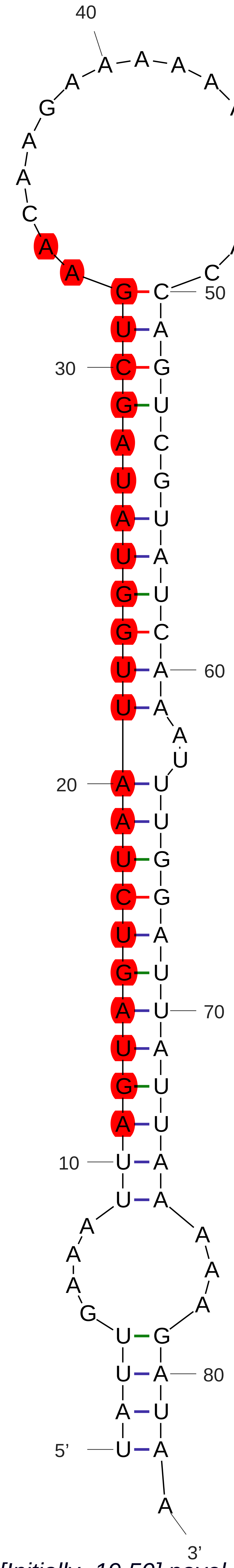

dG = -19.50 [Initially -19.50] novel_mir_111_3

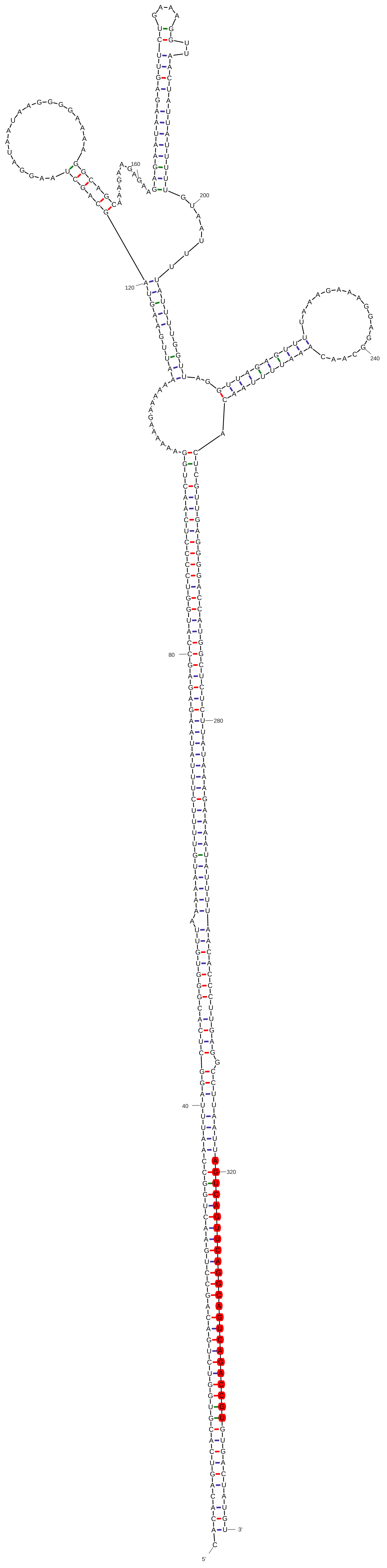

dG = -157.58 [Initially -165.00] novel_mir_2625

Output of sir_graph (©)
mfold_util 4.6

Created Sun Jan 13 08:17:19 2013

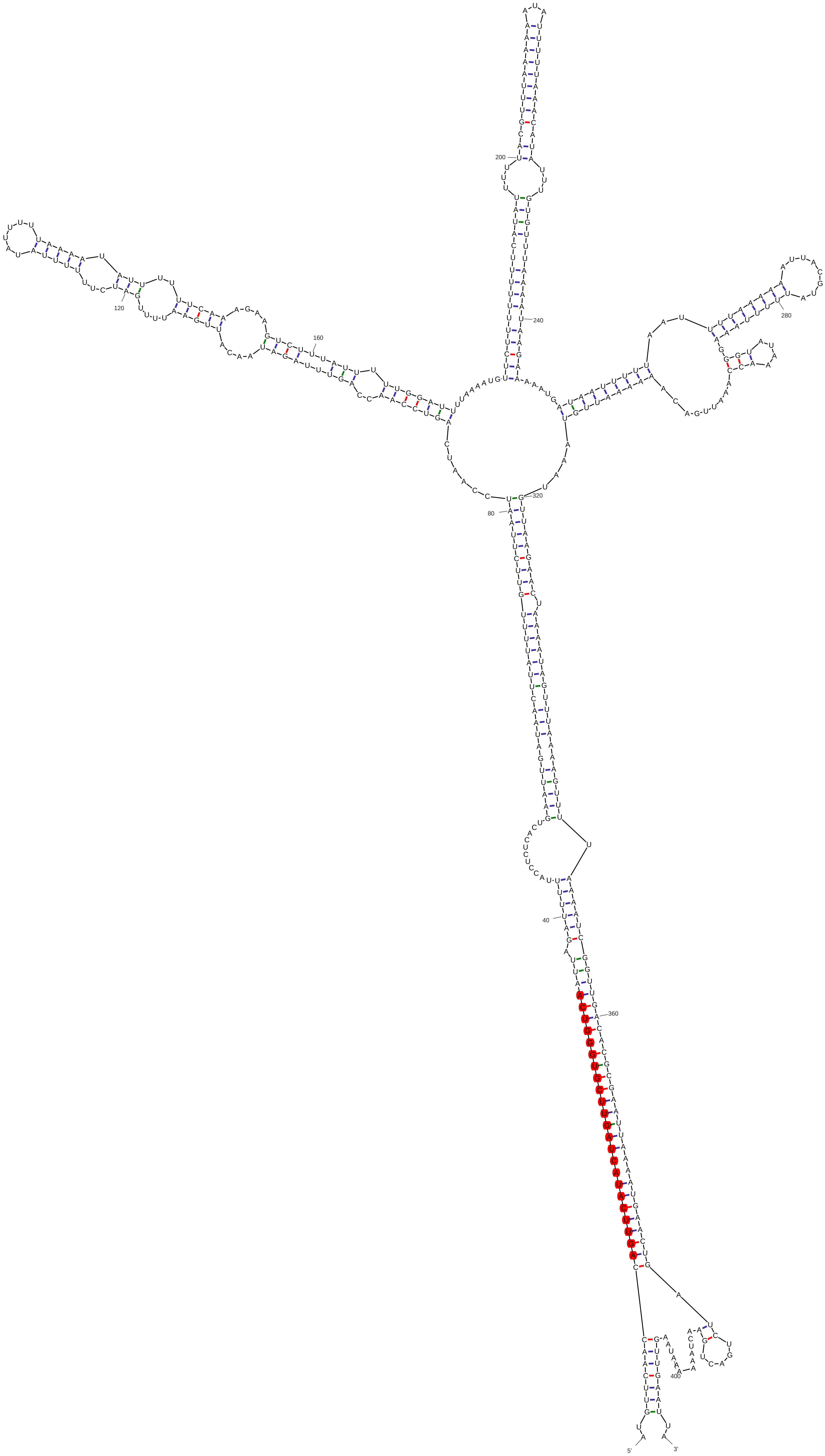

dG = -72.22 [Initially -79.00] novel_mir_4309

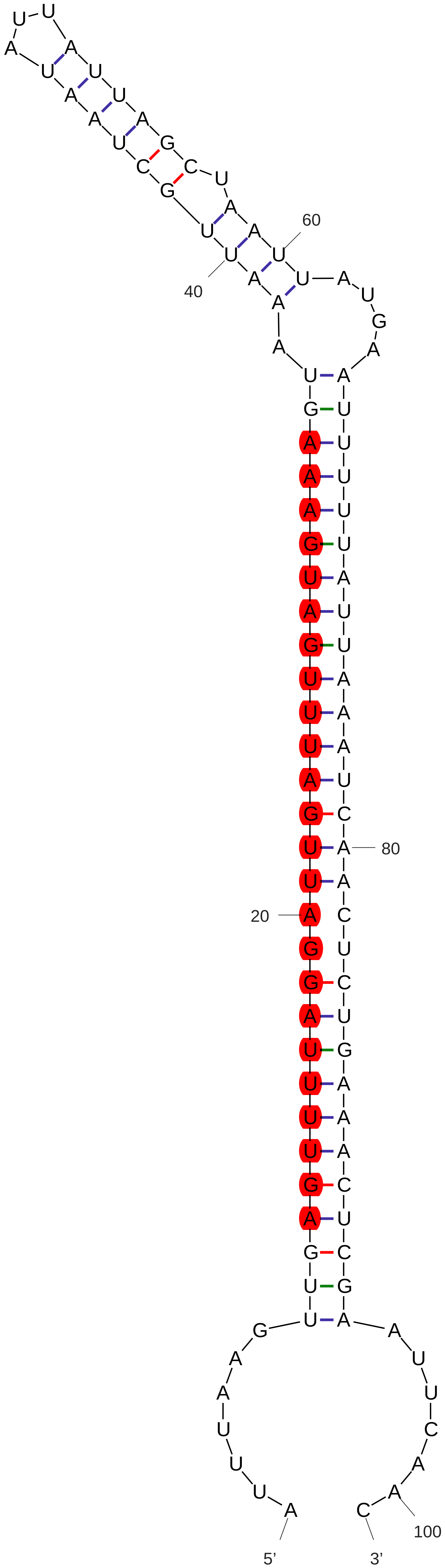

dG = -33.90 [Initially -33.90] novel_mir_5062_1

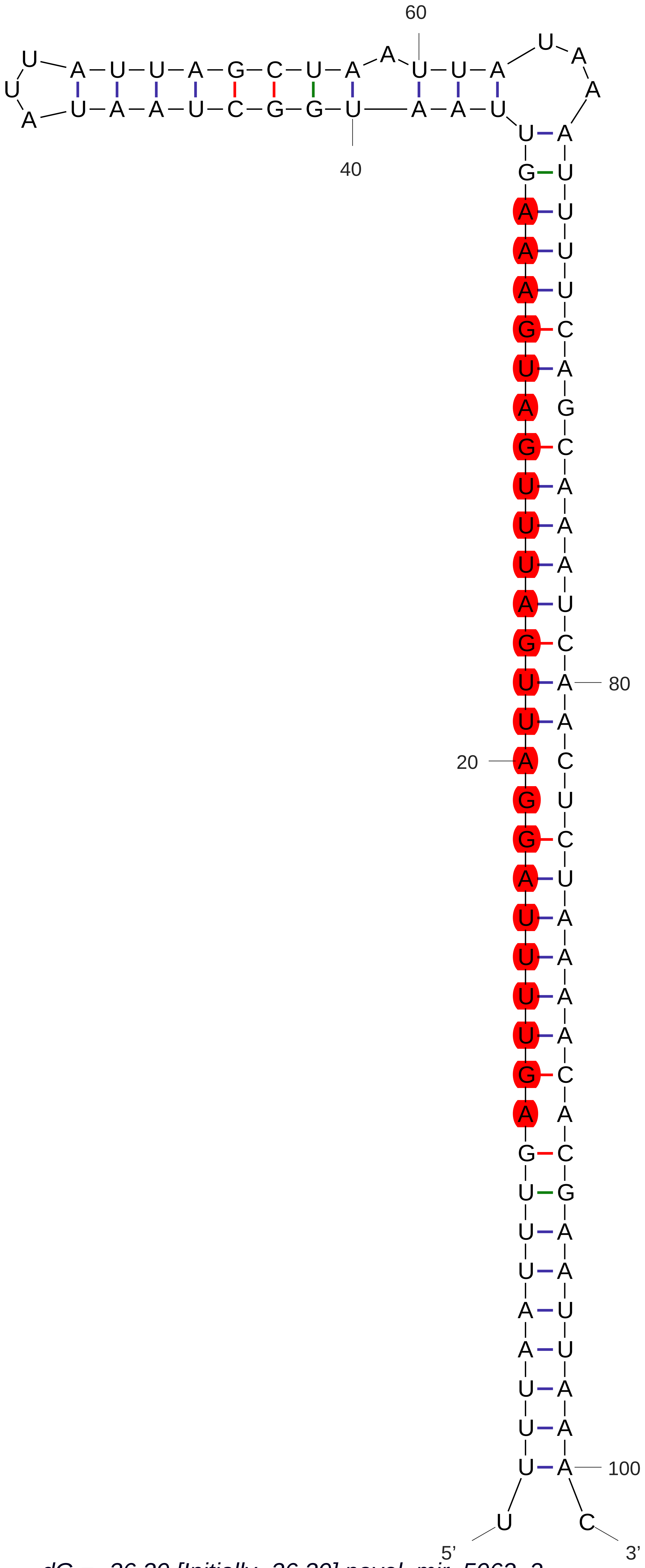

dG = -36.30 [Initially -36.30] novel_mir_5062_2

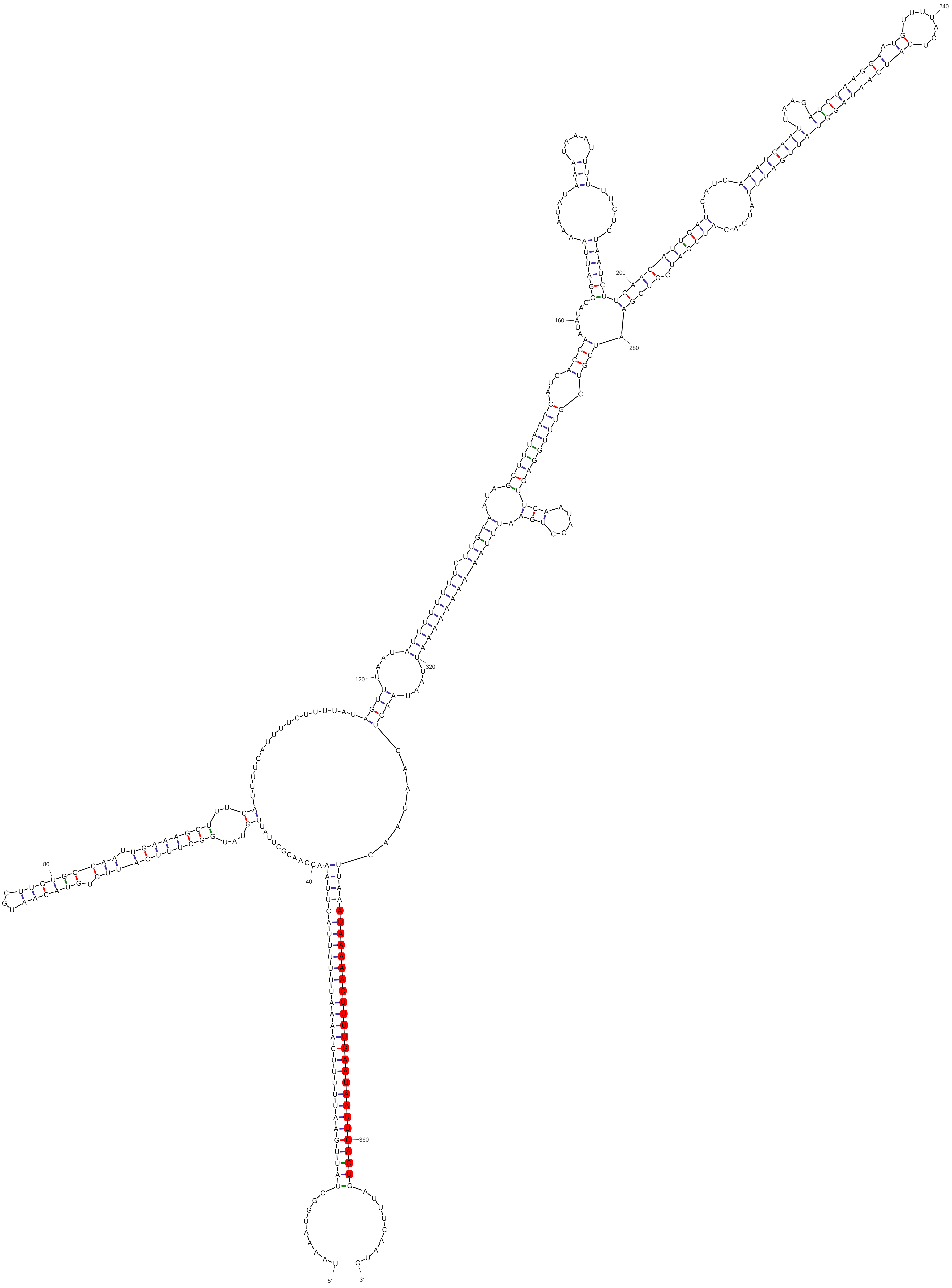

dG = -57.96 [Initially -65.70] novel_mir_4003

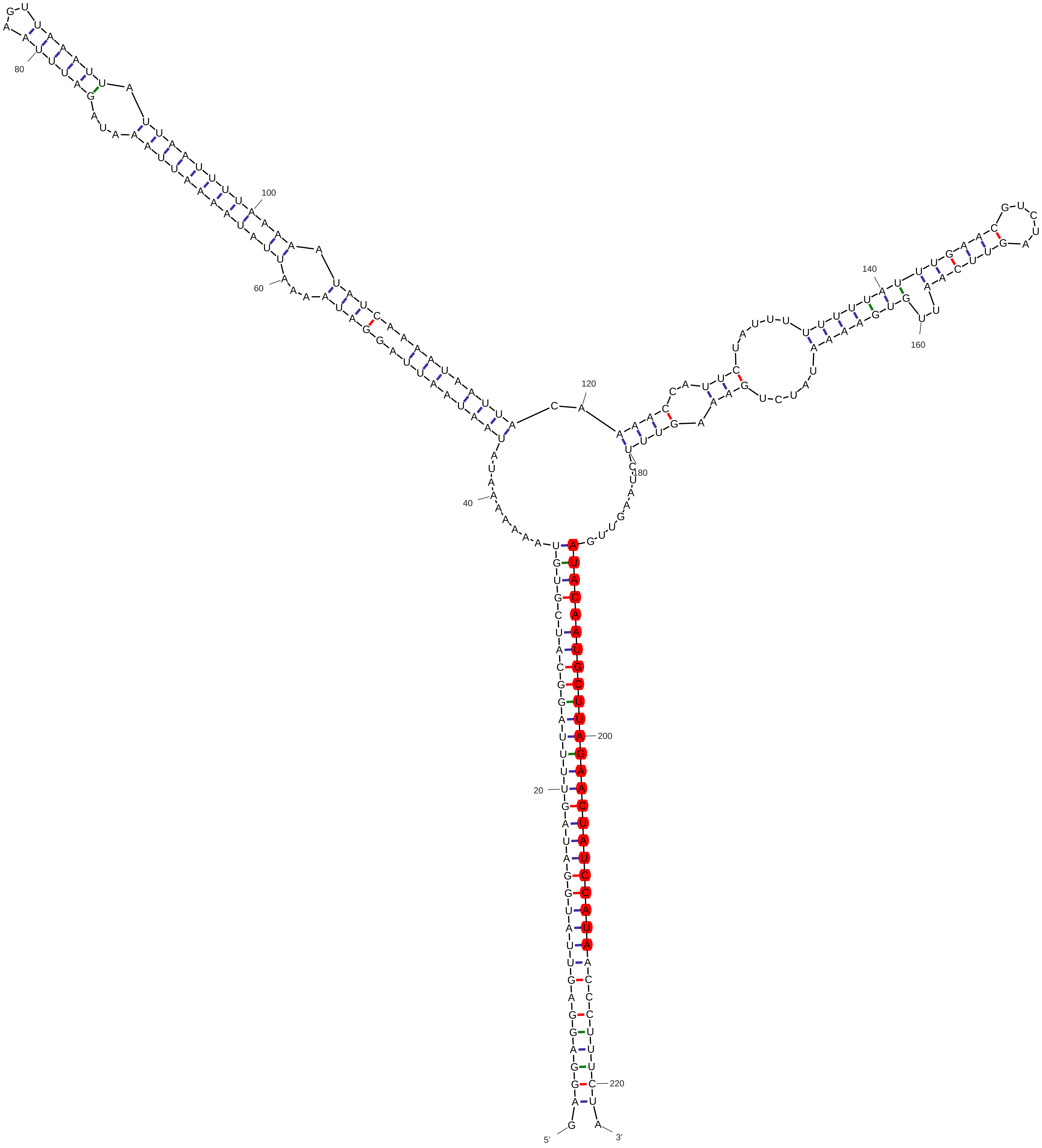

dG = -53.73 [Initially -57.20] novel_mir_267

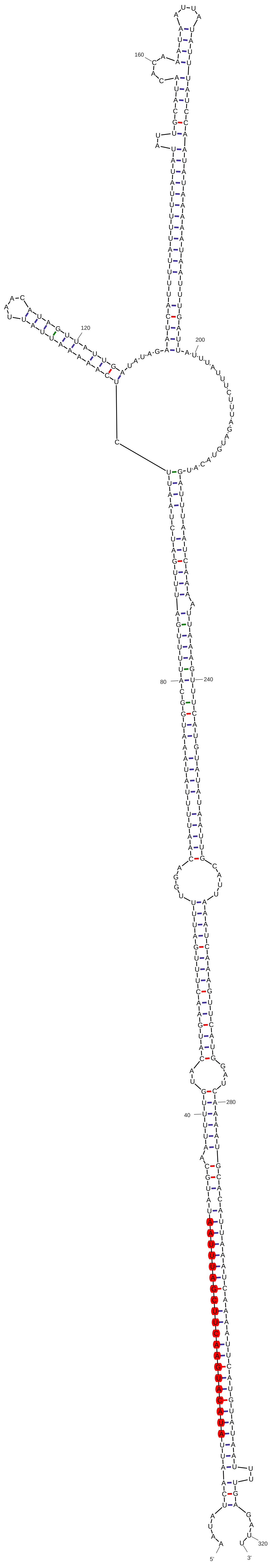

dG = -79.31 [Initially -82.90] novel_mir_1251

Output of sir_graph (©)
mfold_util 4.6

Created Sun Jan 13 08:21:35 2013

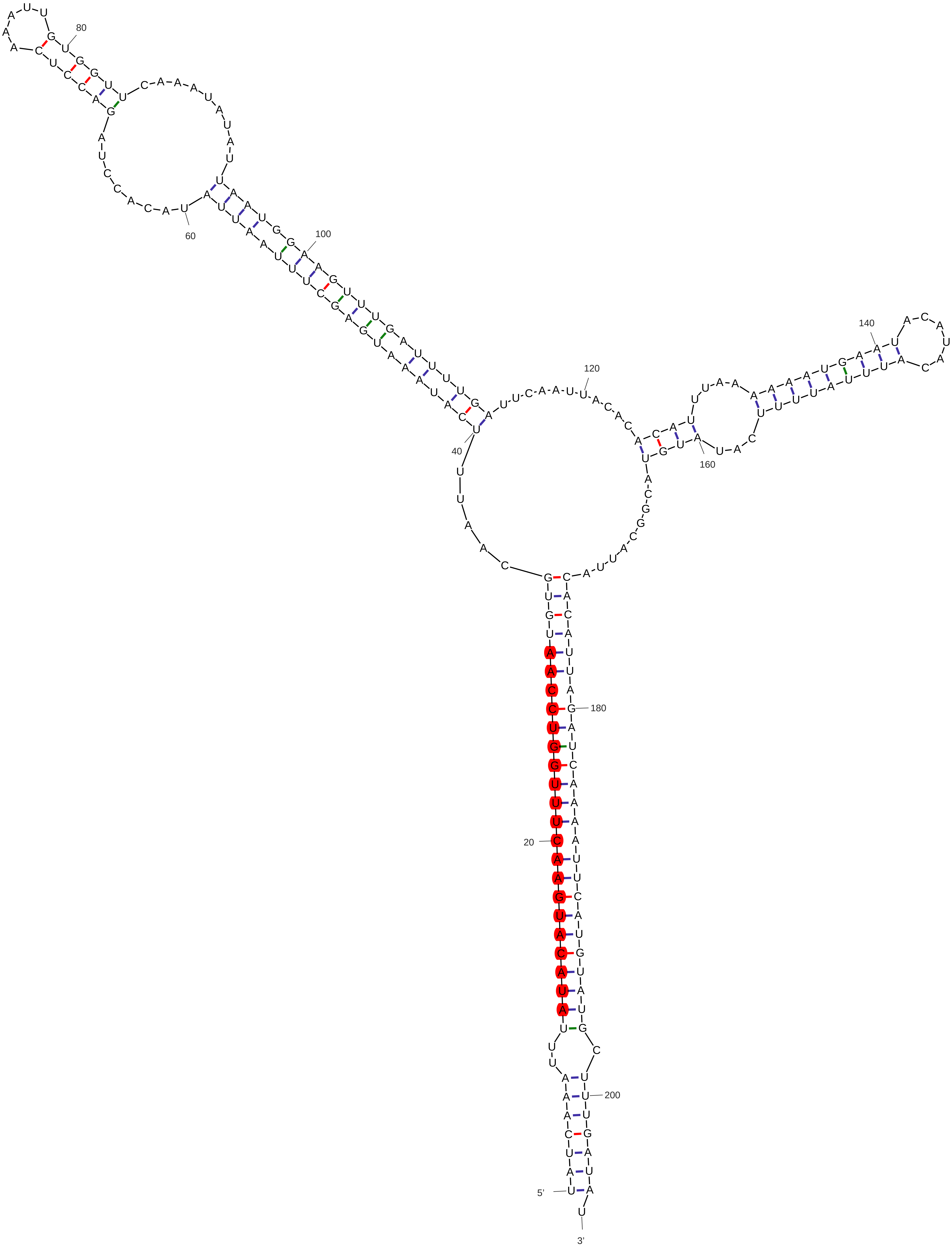

dG = -43.03 [Initially -47.40] novel_mir_844_1

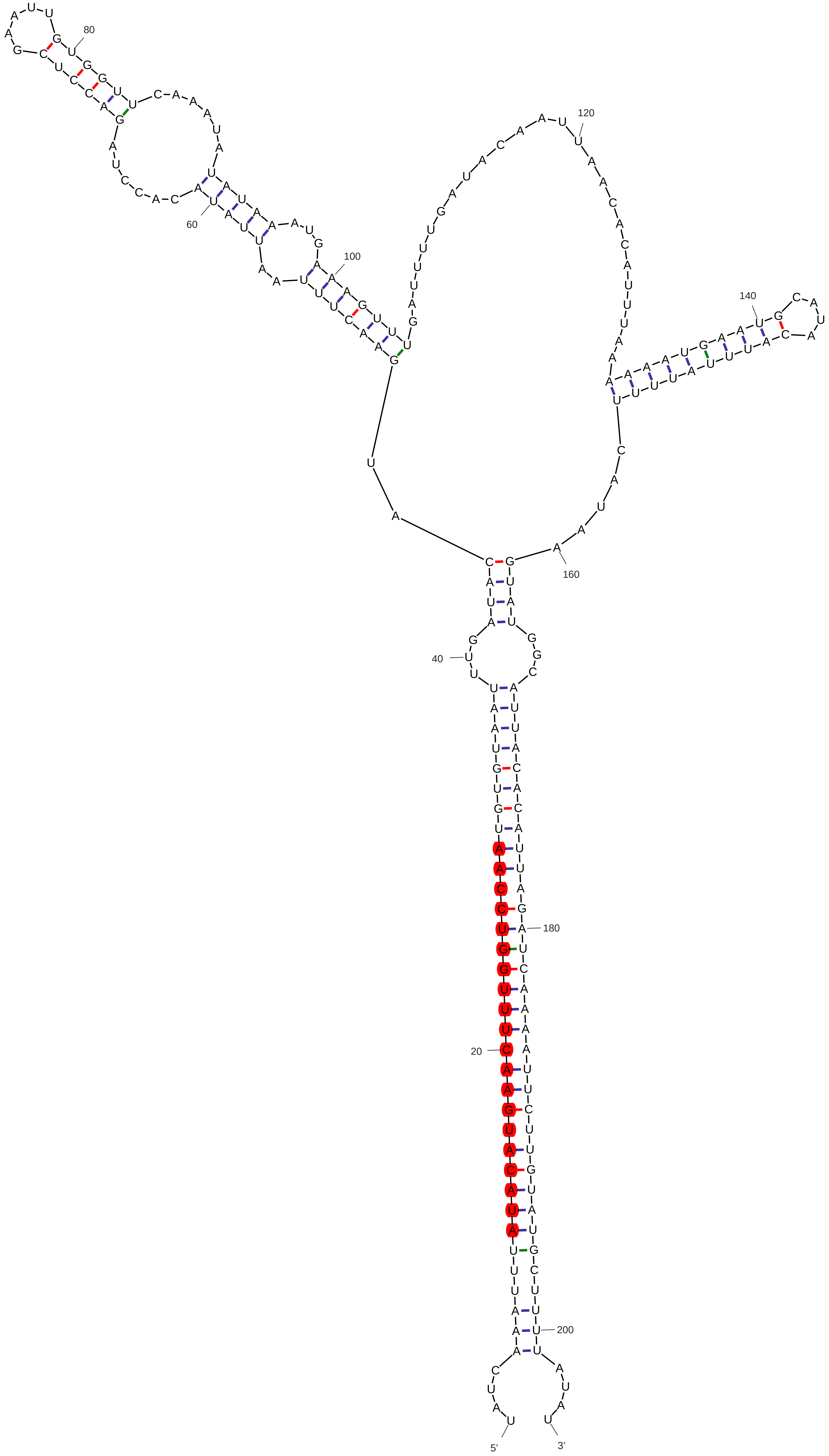

dG = -38.62 [Initially -44.40] novel_mir_844_2

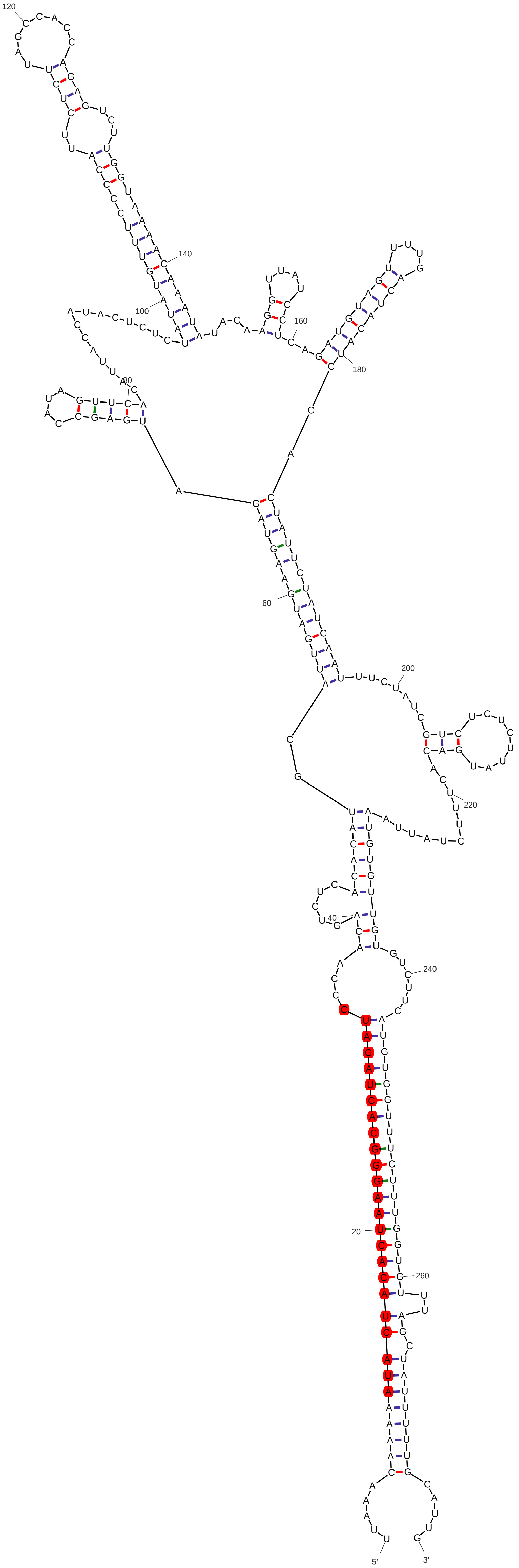

dG = -56.96 [Initially -63.00] novel_mir_4337

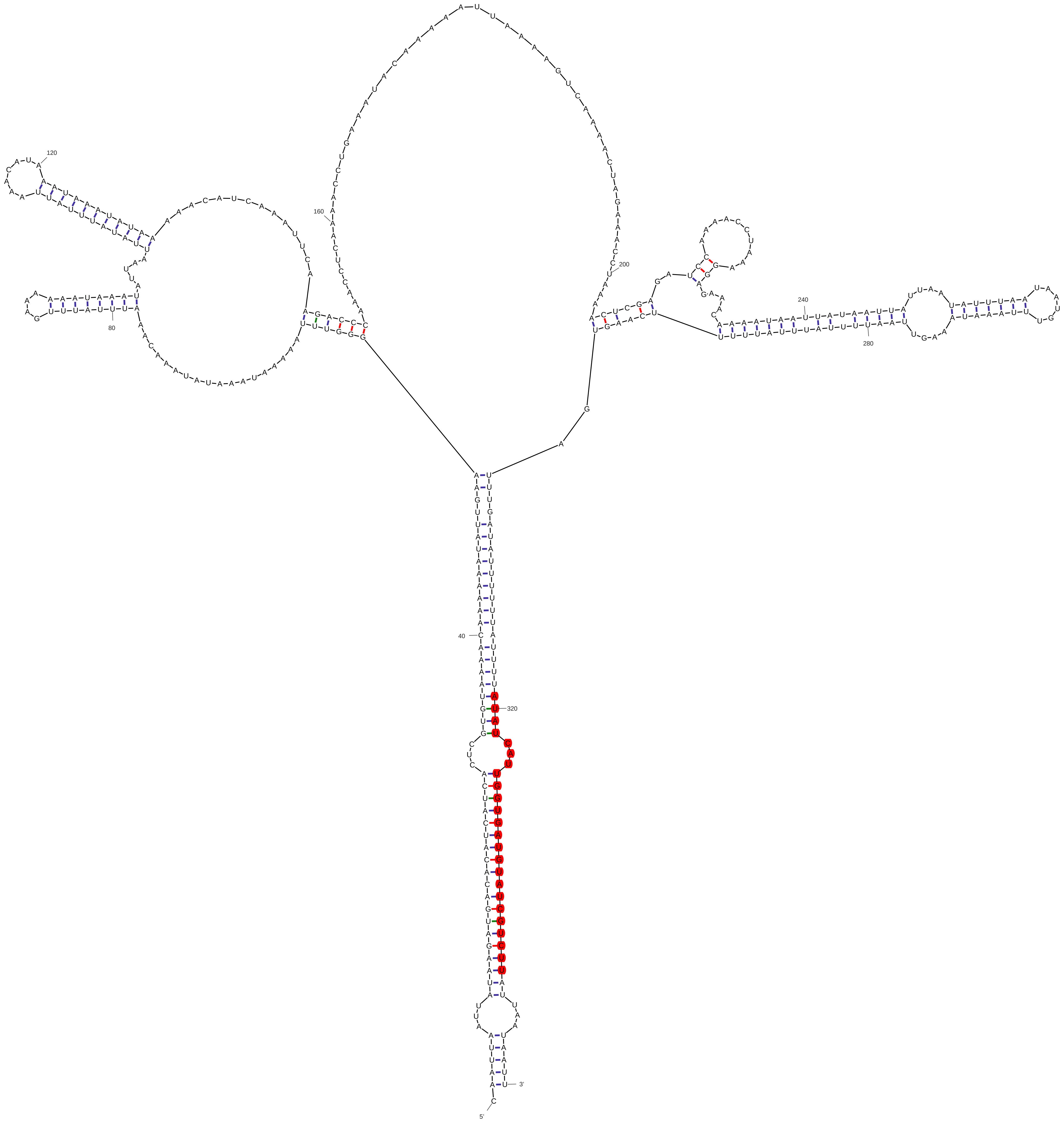

dG = -55.31 [Initially -67.20] novel_mir_4065

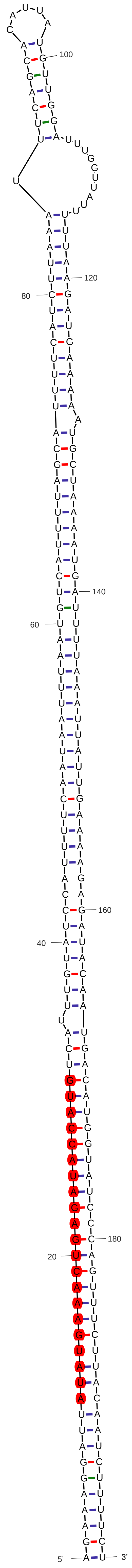

dG = -96.20 [Initially -95.70] novel_mir_2455_3_2_1

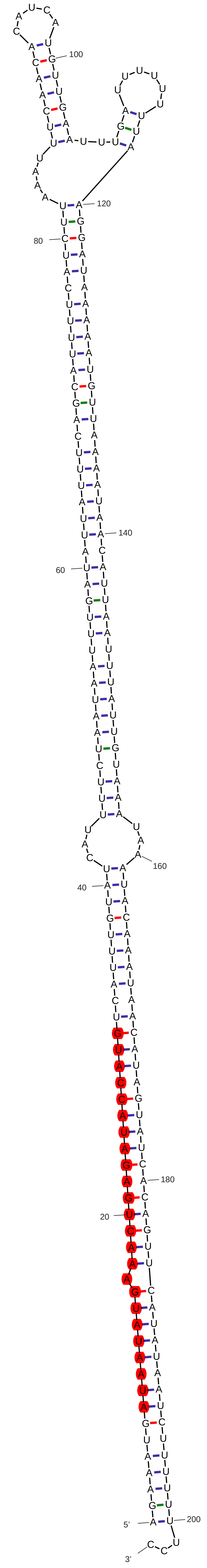

dG = -51.60 [Initially -53.90] novel_mir_2455_3_2_2

Output of sir_graph (©)
mfold_util 4.6

Created Sun Jan 13 07:08:44 2013

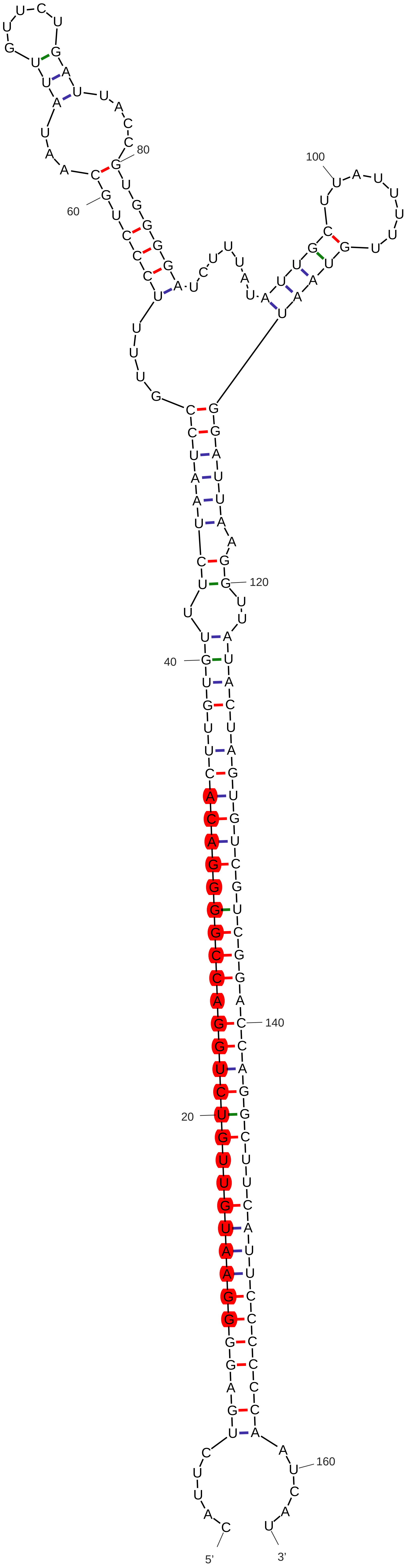

dG = -61.33 [Initially -64.70] novel_mir_1398

Output of sir_graph (©)
mfold_util 4.6

Created Sun Jan 13 08:26:25 2013

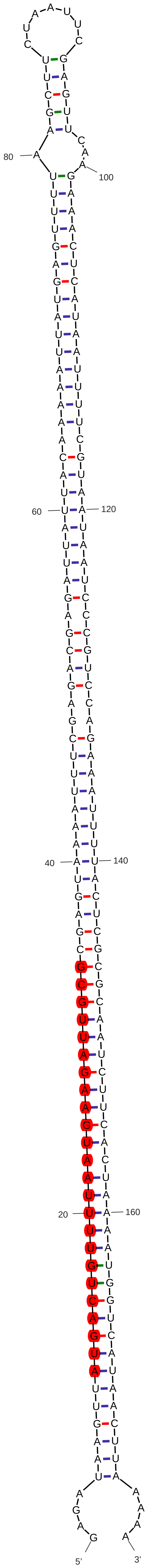

dG = -96.40 [Initially -96.40] novel_mir_2558

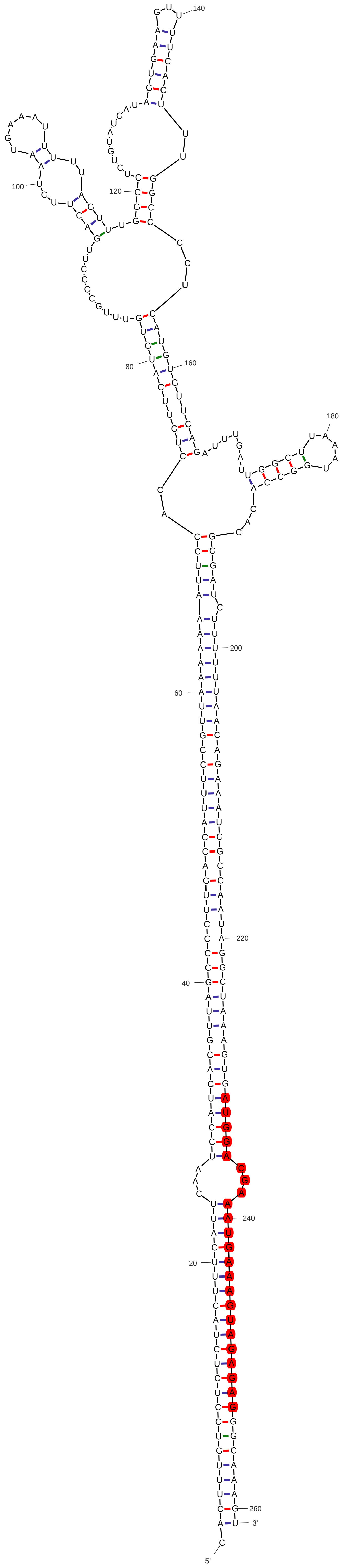

dG = -102.71 [Initially -108.50] novel_mir_3622

Output of sir_graph (©)
mfold_util 4.6

Created Sun Jan 13 08:26:42 2013

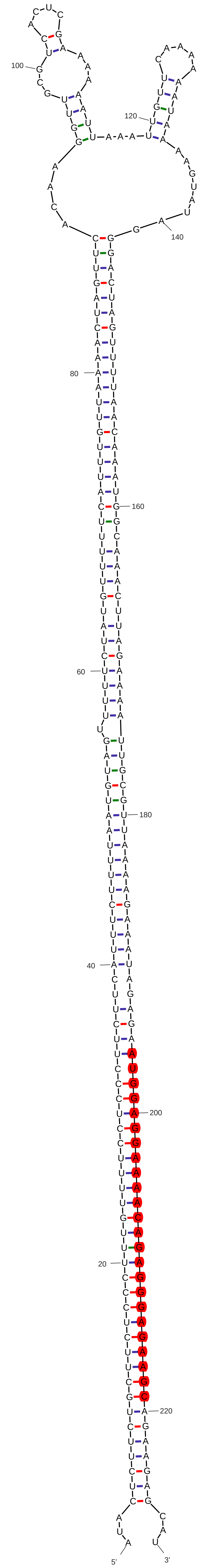

dG = -111.39 [Initially -115.50] novel_mir_2552

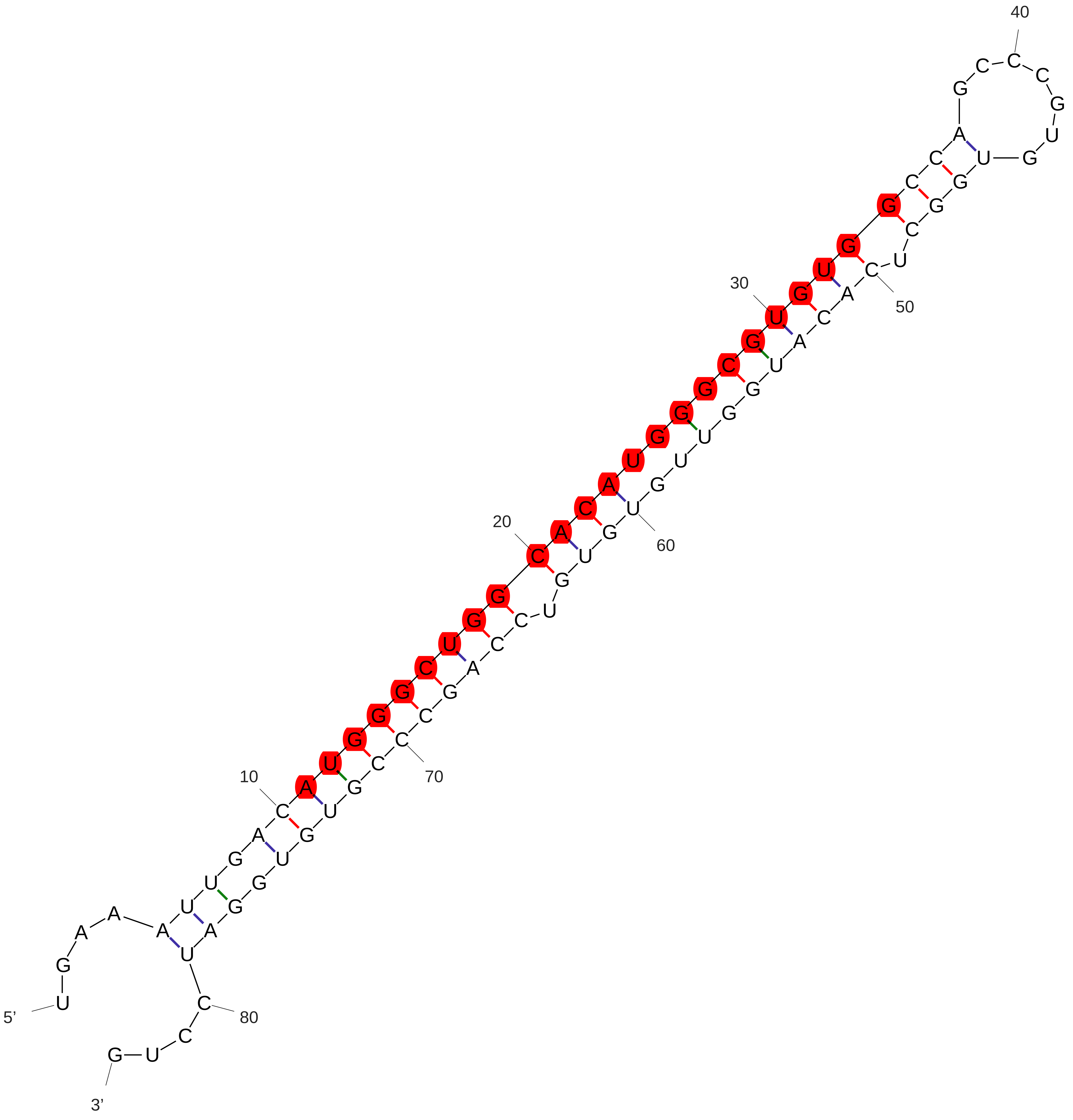

dG = -48.60 [Initially -48.60] novel_mir_4192

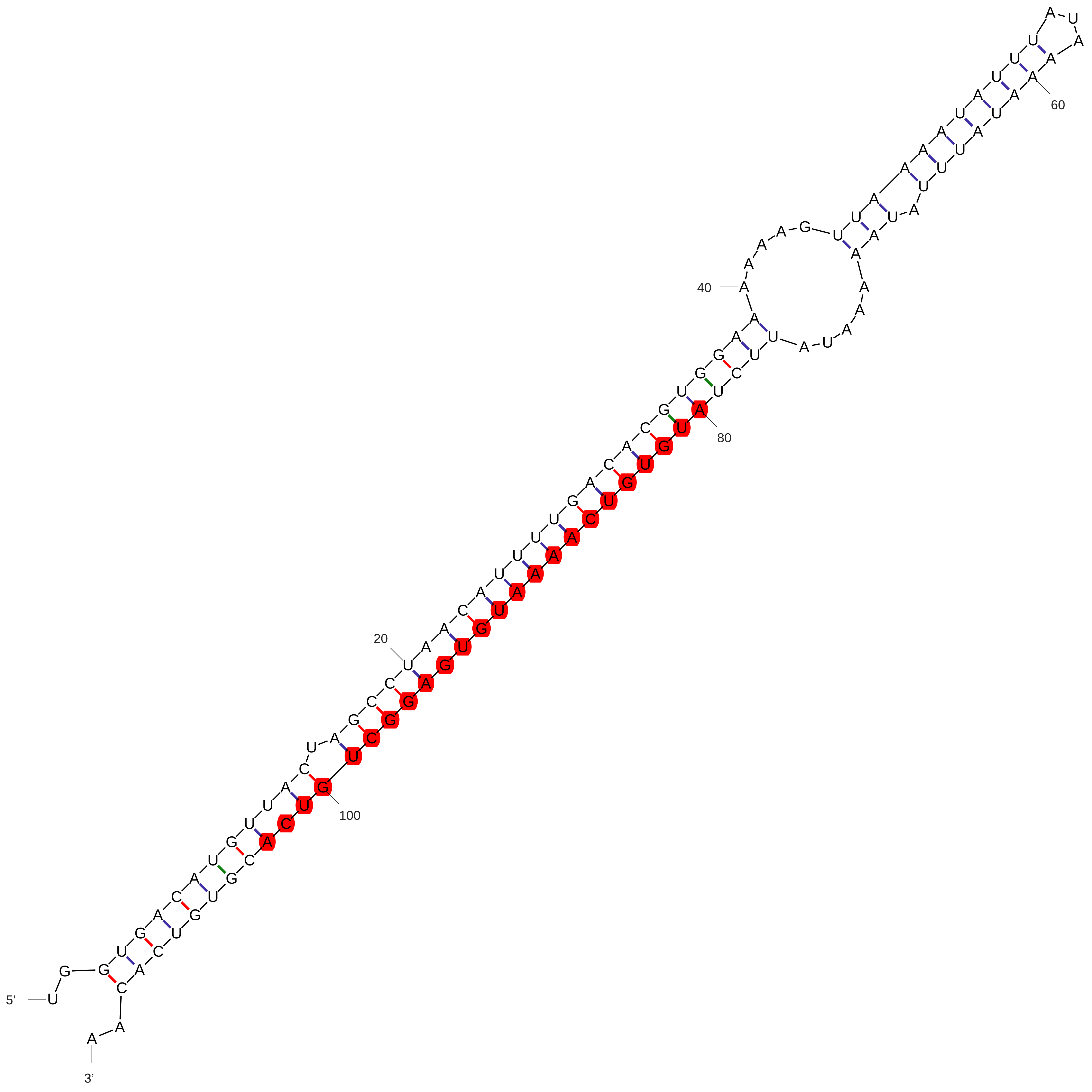

dG = -51.40 [Initially -51.40] novel_mir_2522

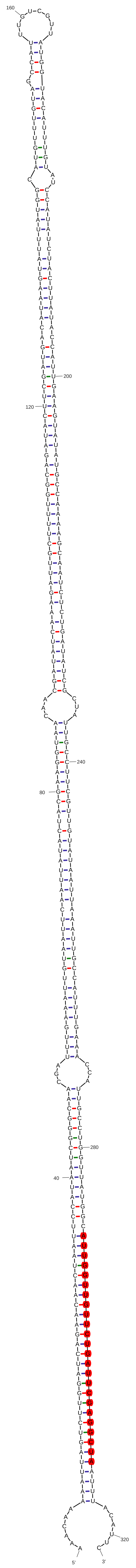

dG = -164.20 [Initially -164.20] novel_mir_2467

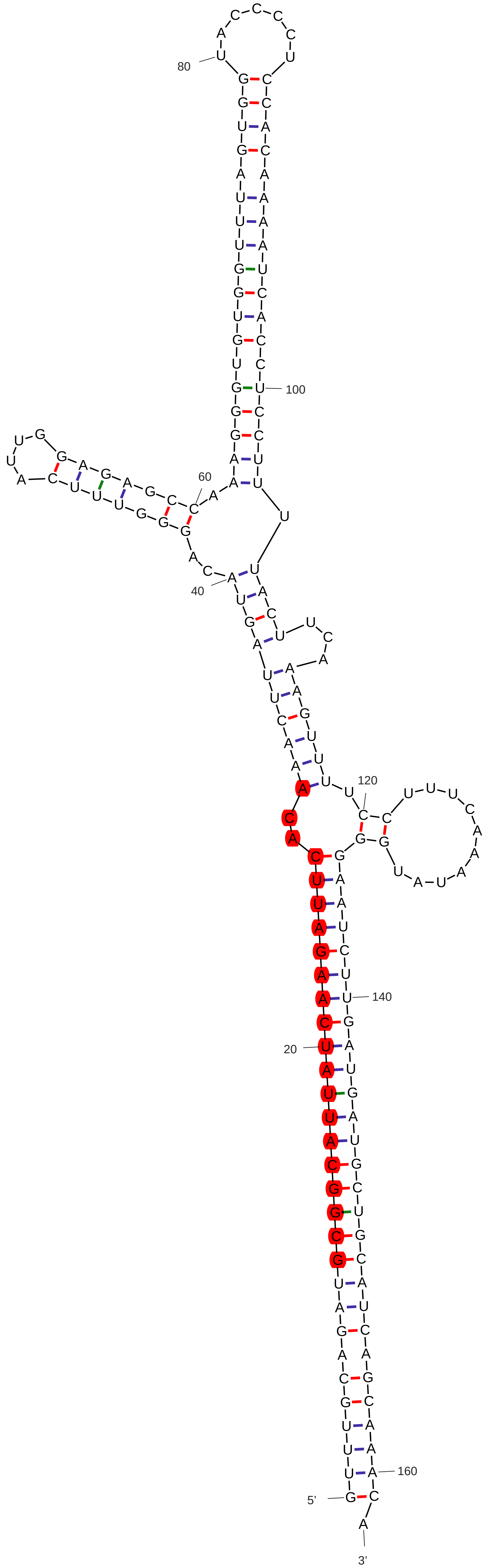

dG = -67.60 [Initially -70.30] novel_mir_974

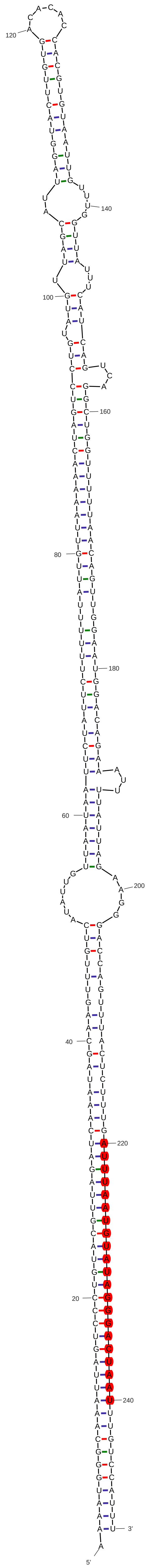

dG = -94.80 [Initially -94.80] 150_novel_mir_5694

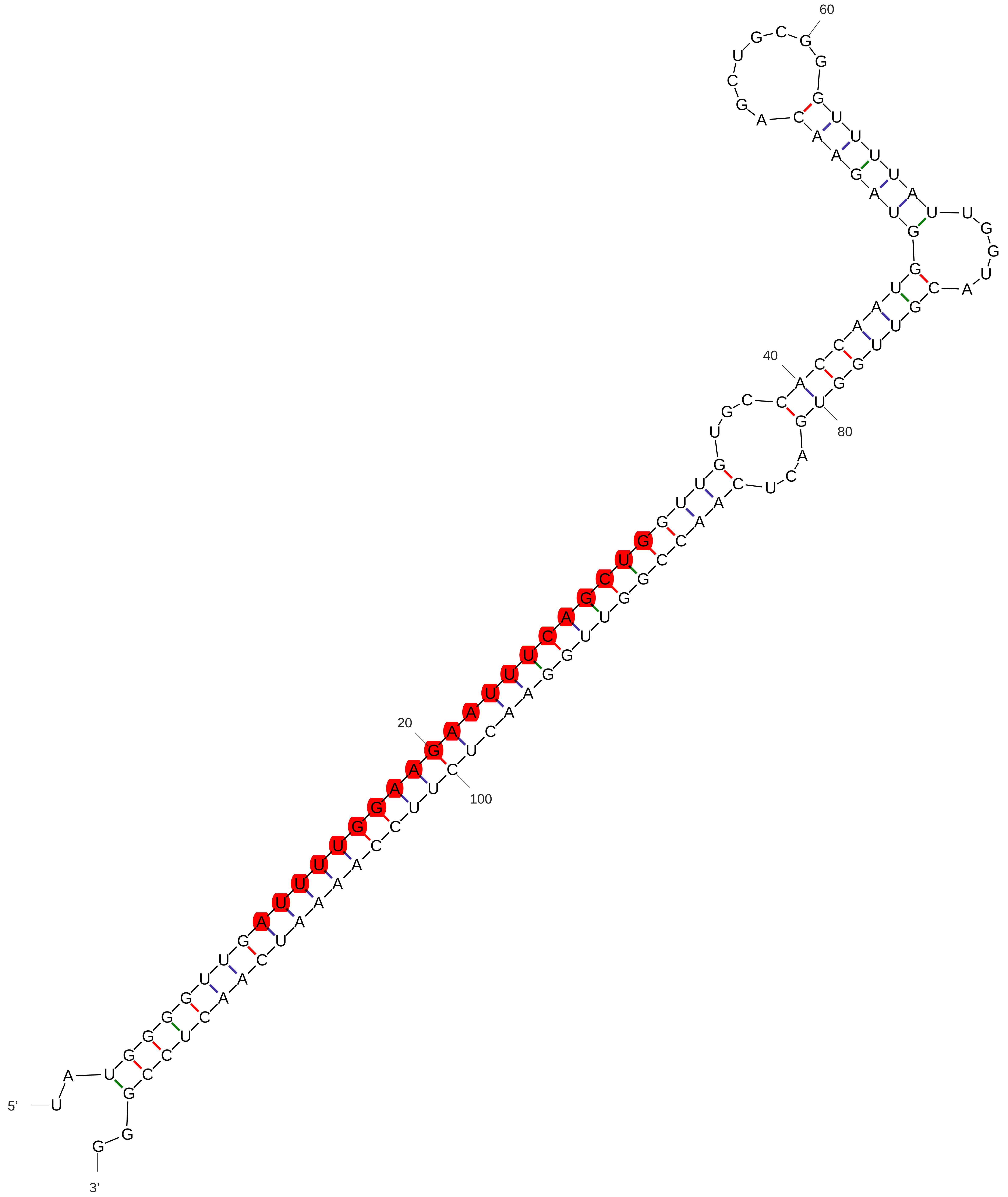

dG = -63.80 [Initially -63.80] novel_mir_5708

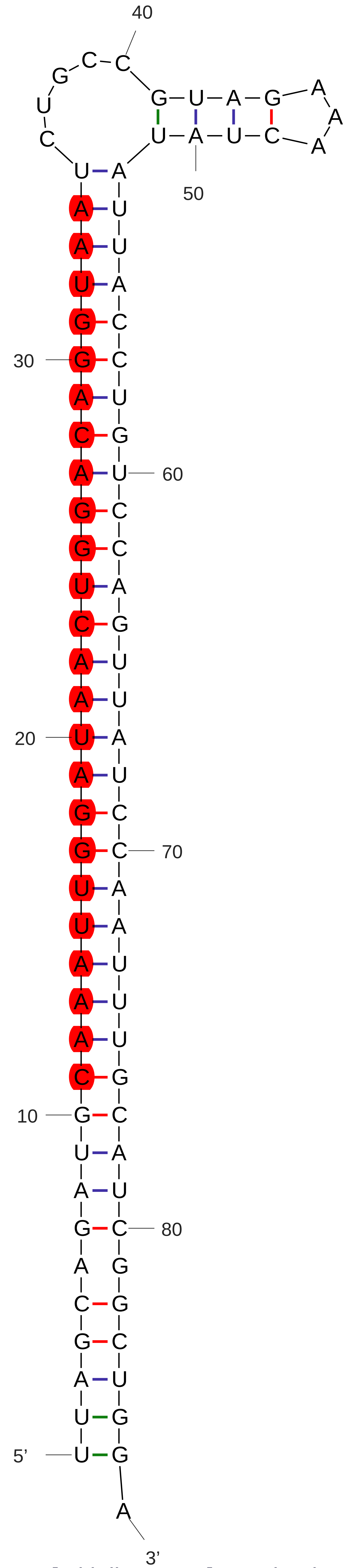

dG = -54.30 [Initially -54.30] novel_mir_925

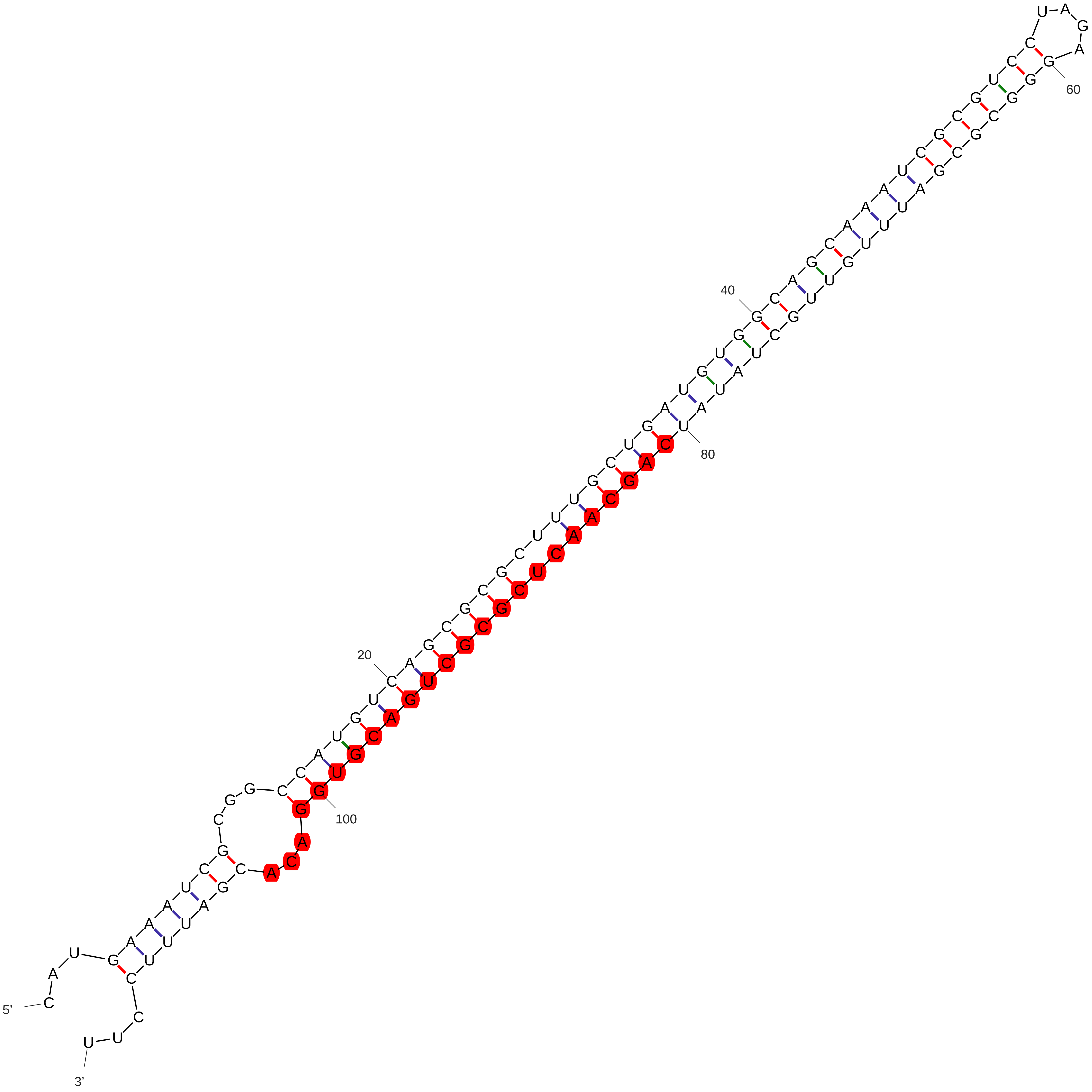

dG = -84.20 [Initially -84.20] novel_mir_4175

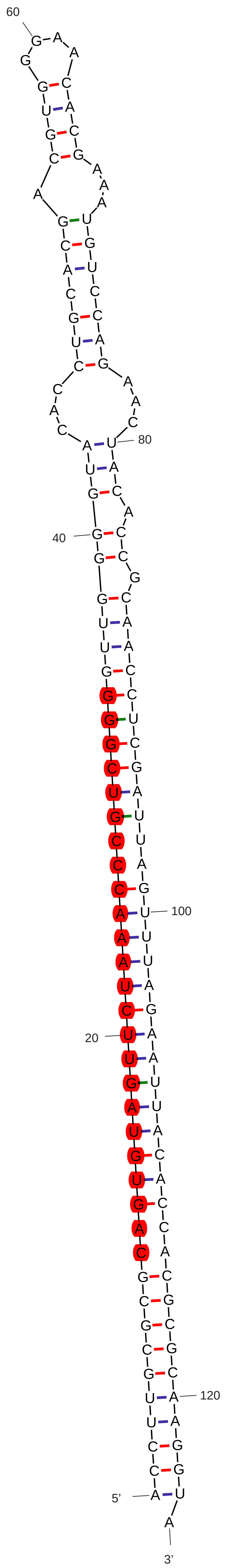

dG = -69.70 [Initially -69.70] novel_mir_2743

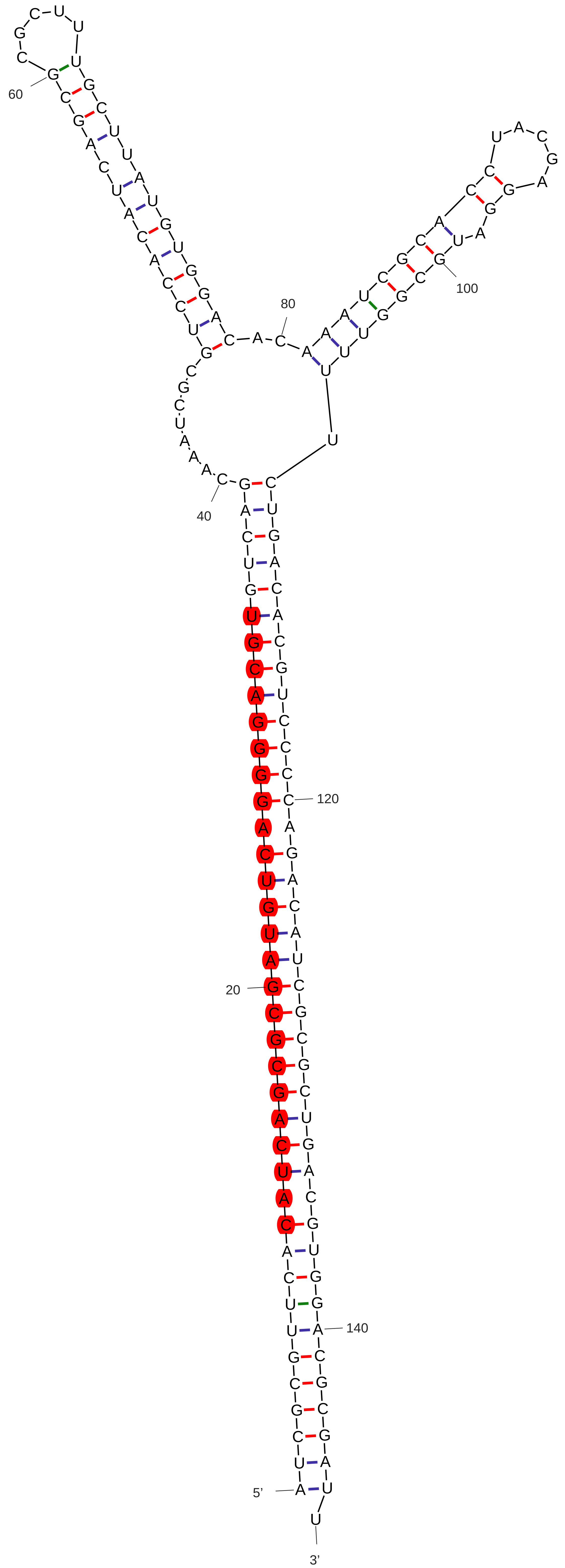

dG = -98.53 [Initially -100.10] novel_mir_4127

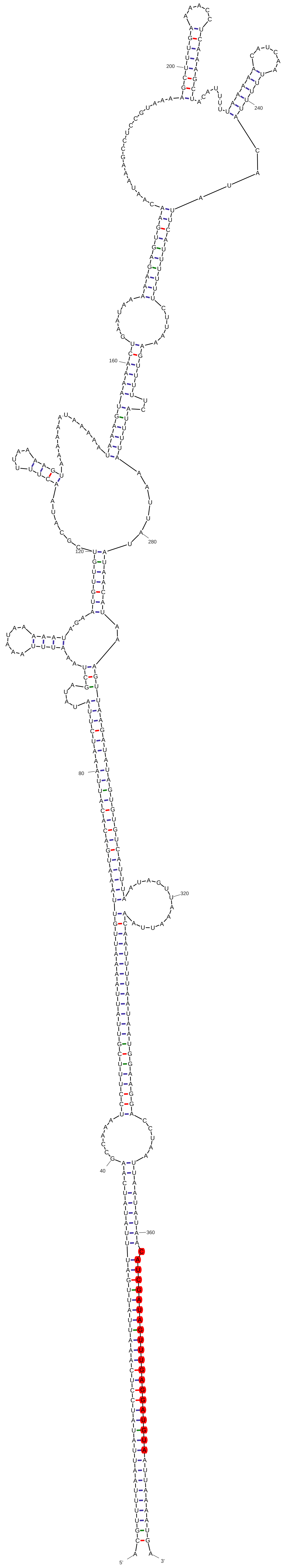

dG = -98.78 [Initially -109.40] novel_mir_849_1

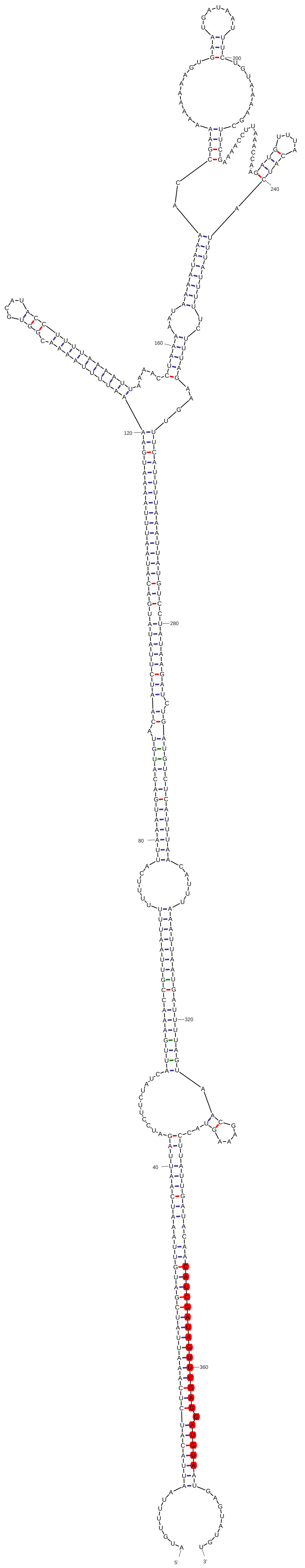

dG = -94.31 [Initially -104.10] novel_mir_849_2

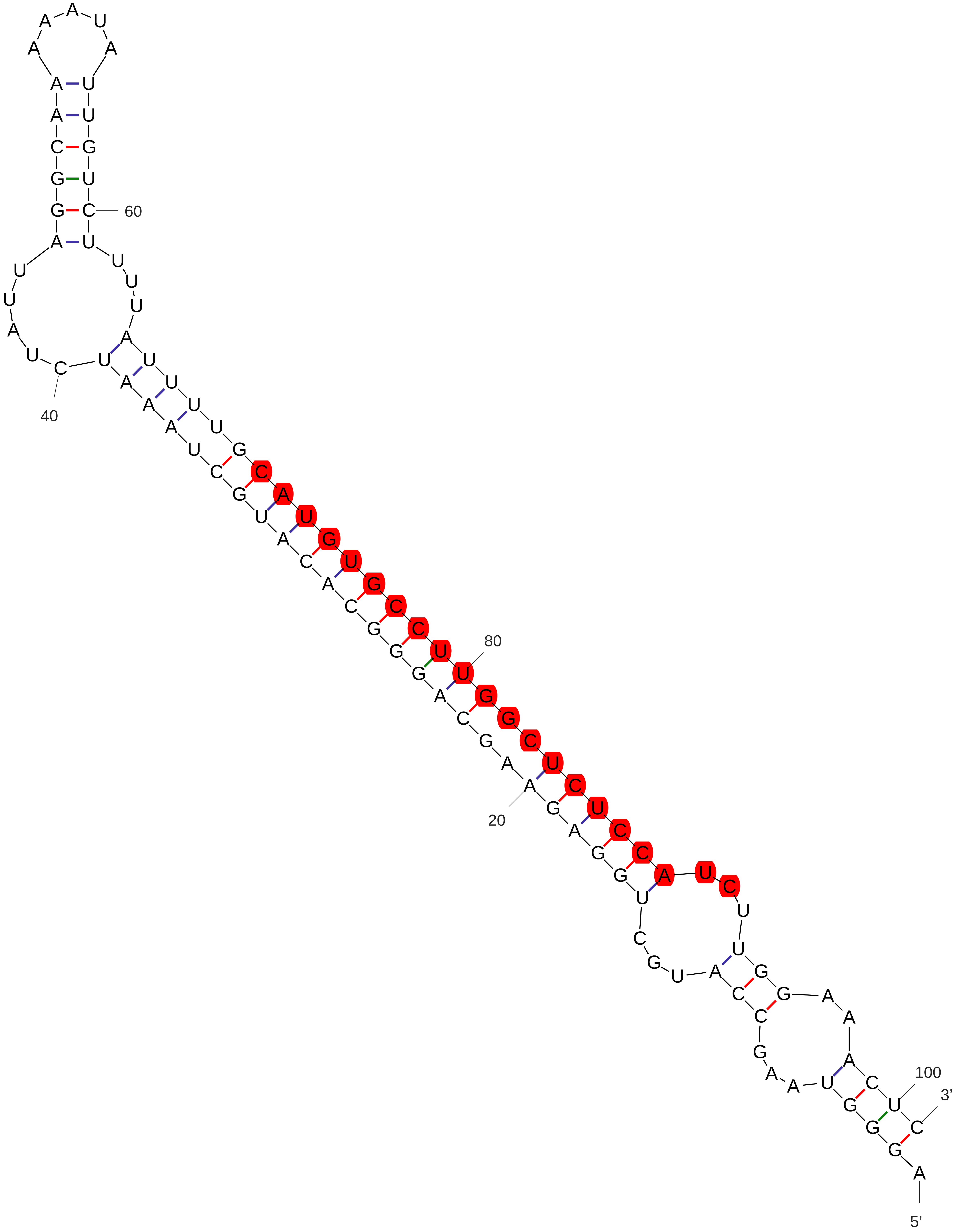

dG = -43.60 [Initially -43.60] novel_mir_4168

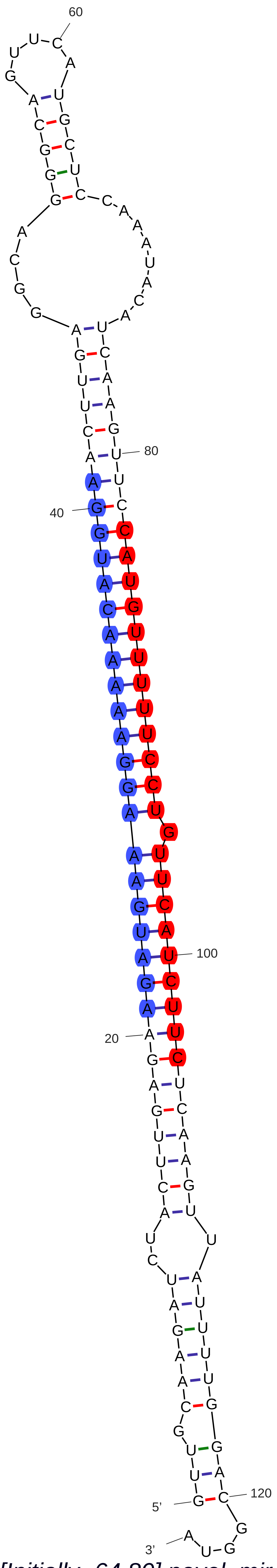

dG = -64.80 [Initially -64.80] novel_mir_2565

Output of sir_graph (©)
mfold_util 4.6

Created Sun Jan 13 08:38:53 2013

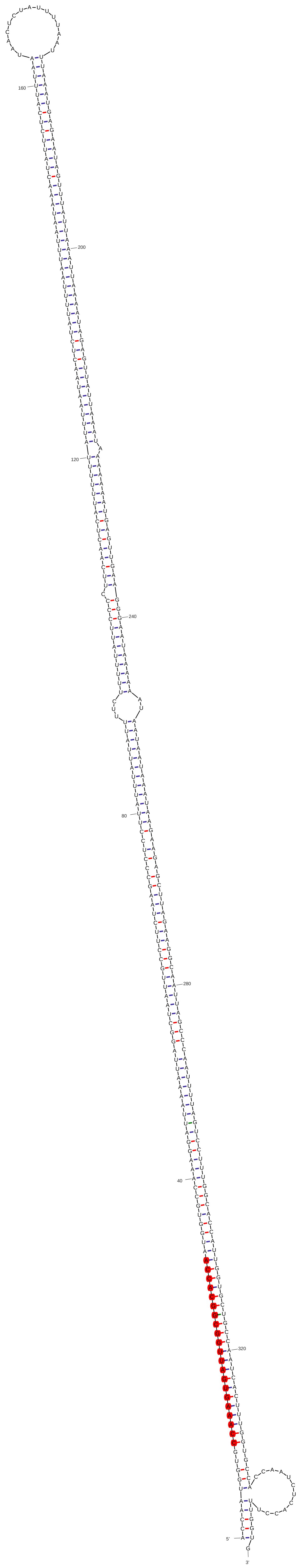

dG = -223.90 [Initially -223.90] novel_mir_5170

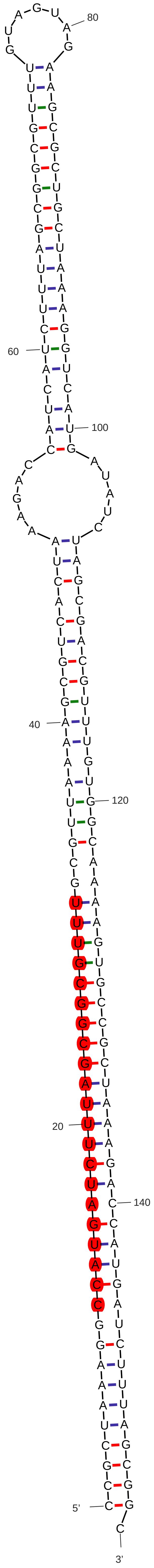

dG = -82.20 [Initially -82.20] novel_mir_2477_1

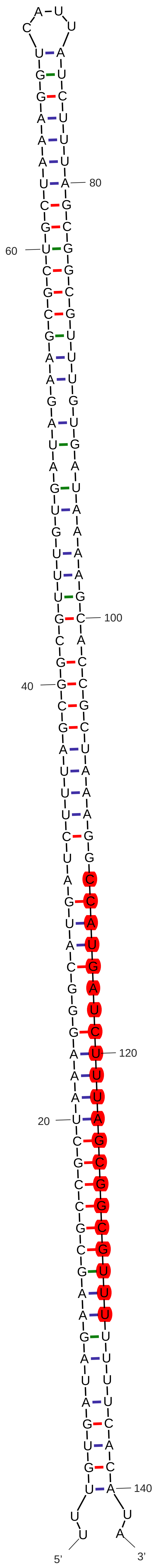

dG = -84.60 [Initially -84.60] novel_mir_2477_2

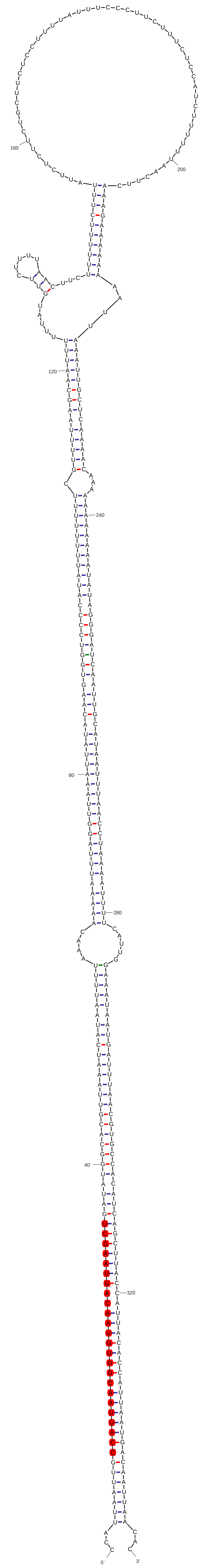

dG = -114.71 [Initially -117.47] novel_mir_5818

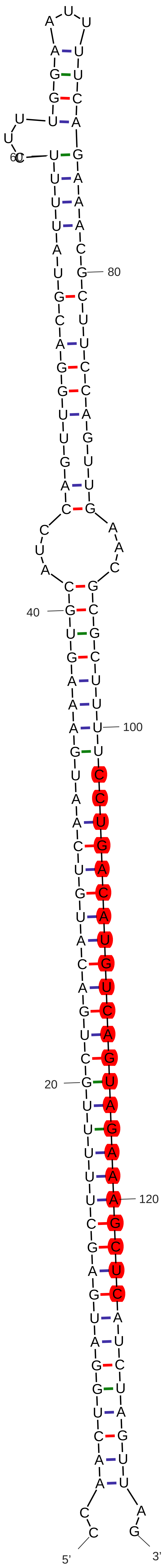

dG = -68.40 [Initially -68.40] novel_mir_2497

Output of sir_graph (©)
mfold_util 4.6

Created Sun Jan 13 08:43:28 2013

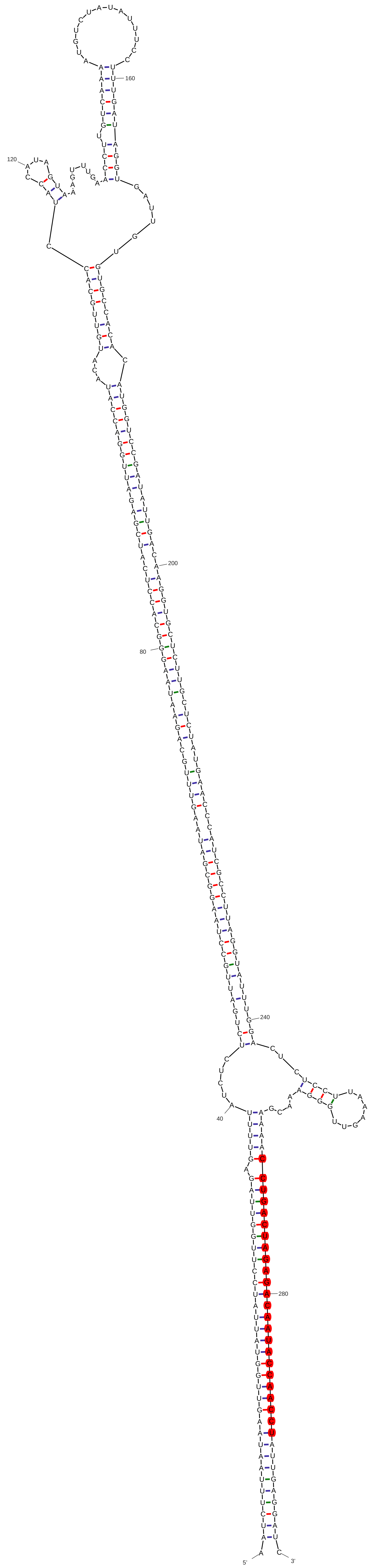

dG = -116.63 [Initially -121.70] novel_mir_5018

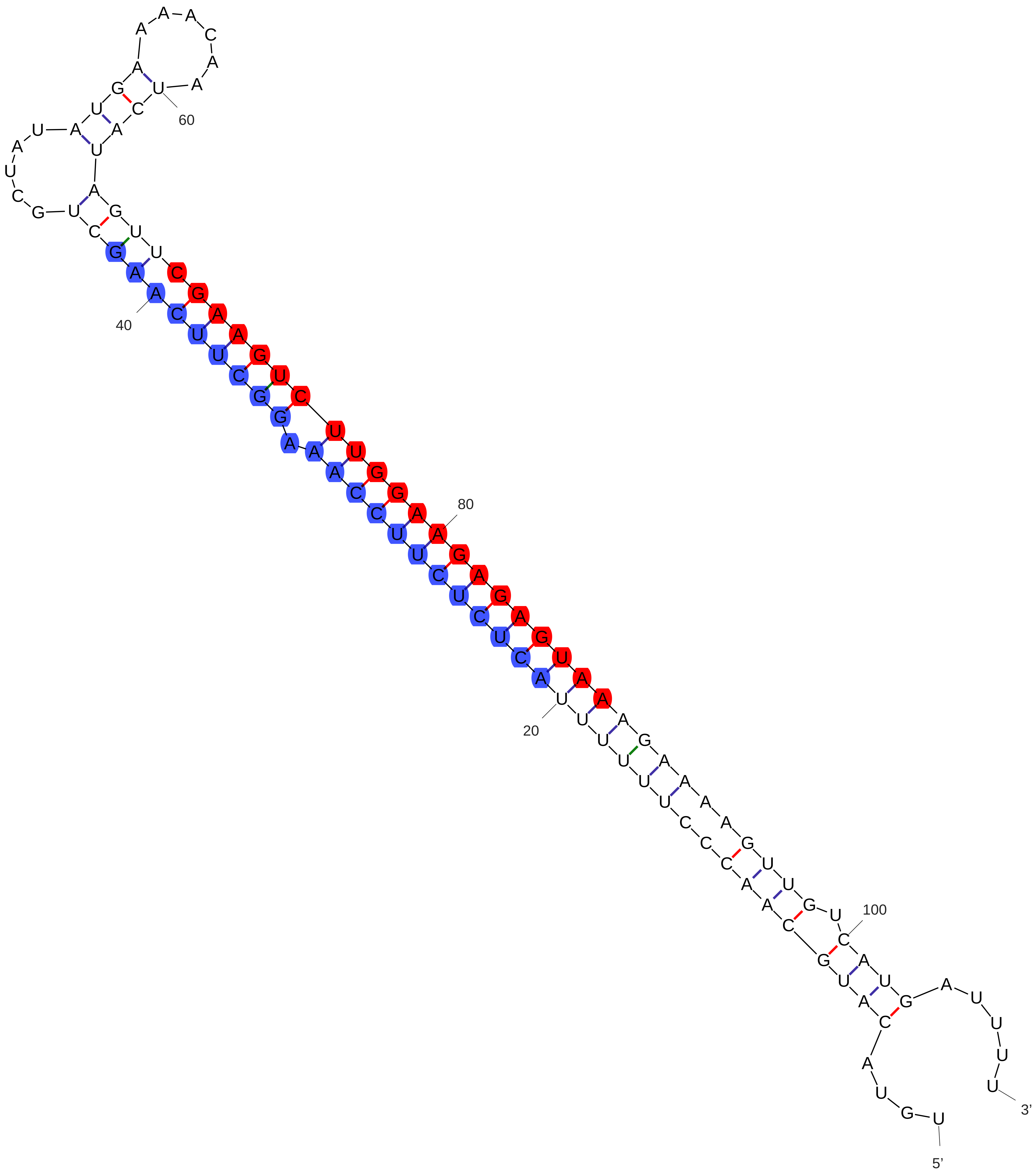

dG = -46.00 [Initially -46.00] novel_mir_986

Output of sir_graph (©)
mfold_util 4.6

Created Sun Jan 13 08:43:33 2013

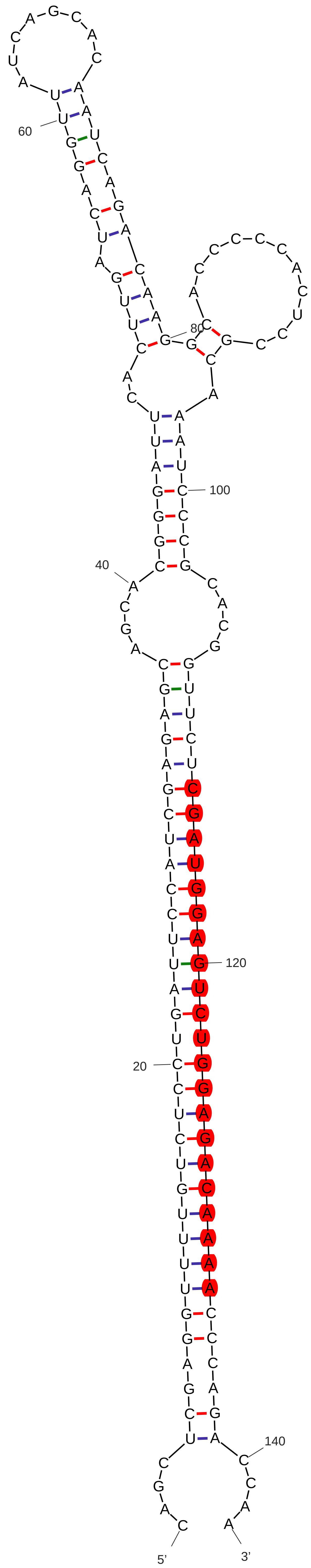

dG = -62.20 [Initially -63.70] novel_mir_4004

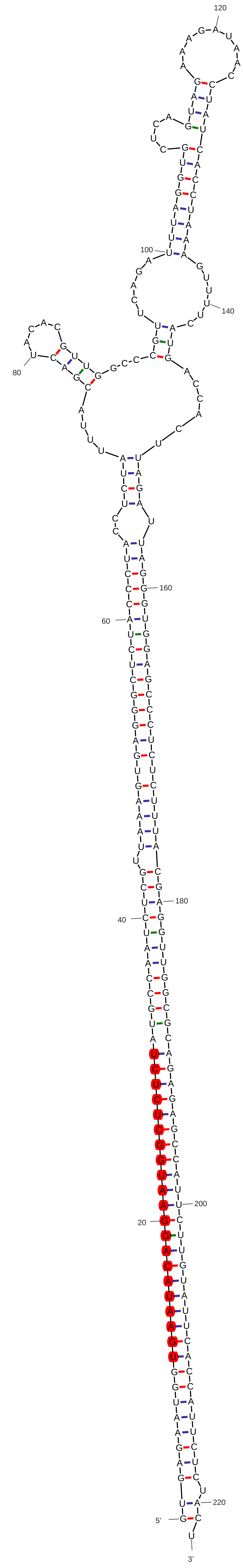

dG = -121.15 [Initially -124.40] novel_mir_890

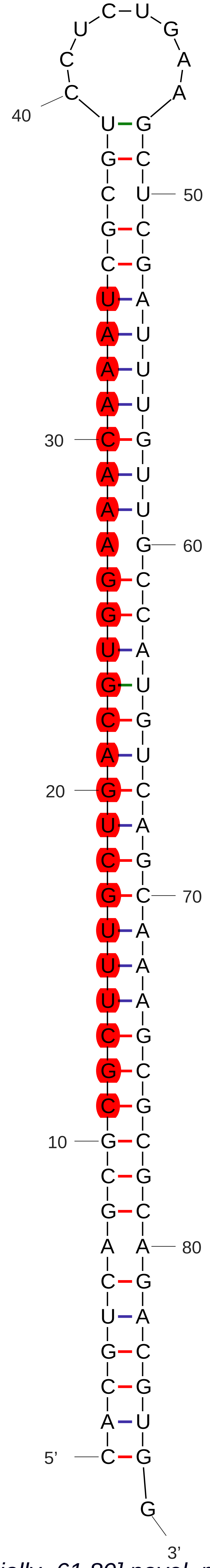

dG = -61.80 [Initially -61.80] novel_mir_4040_1

Output of sir_graph (©)
mfold_util 4.6

Created Sun Jan 13 08:43:40 2013

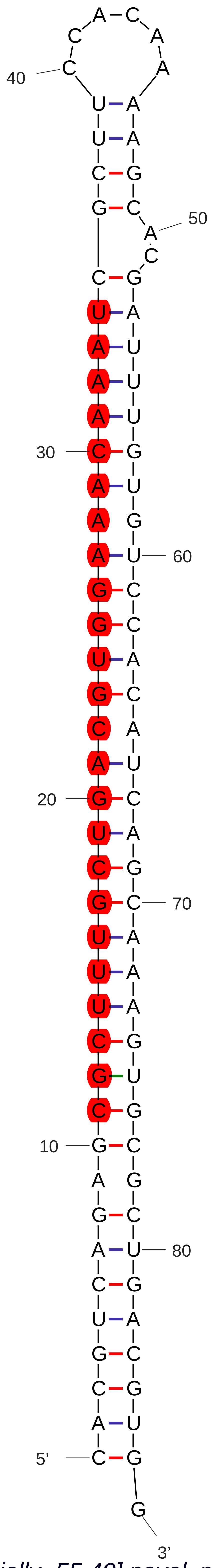

dG = -55.40 [Initially -55.40] novel_mir_4040_2

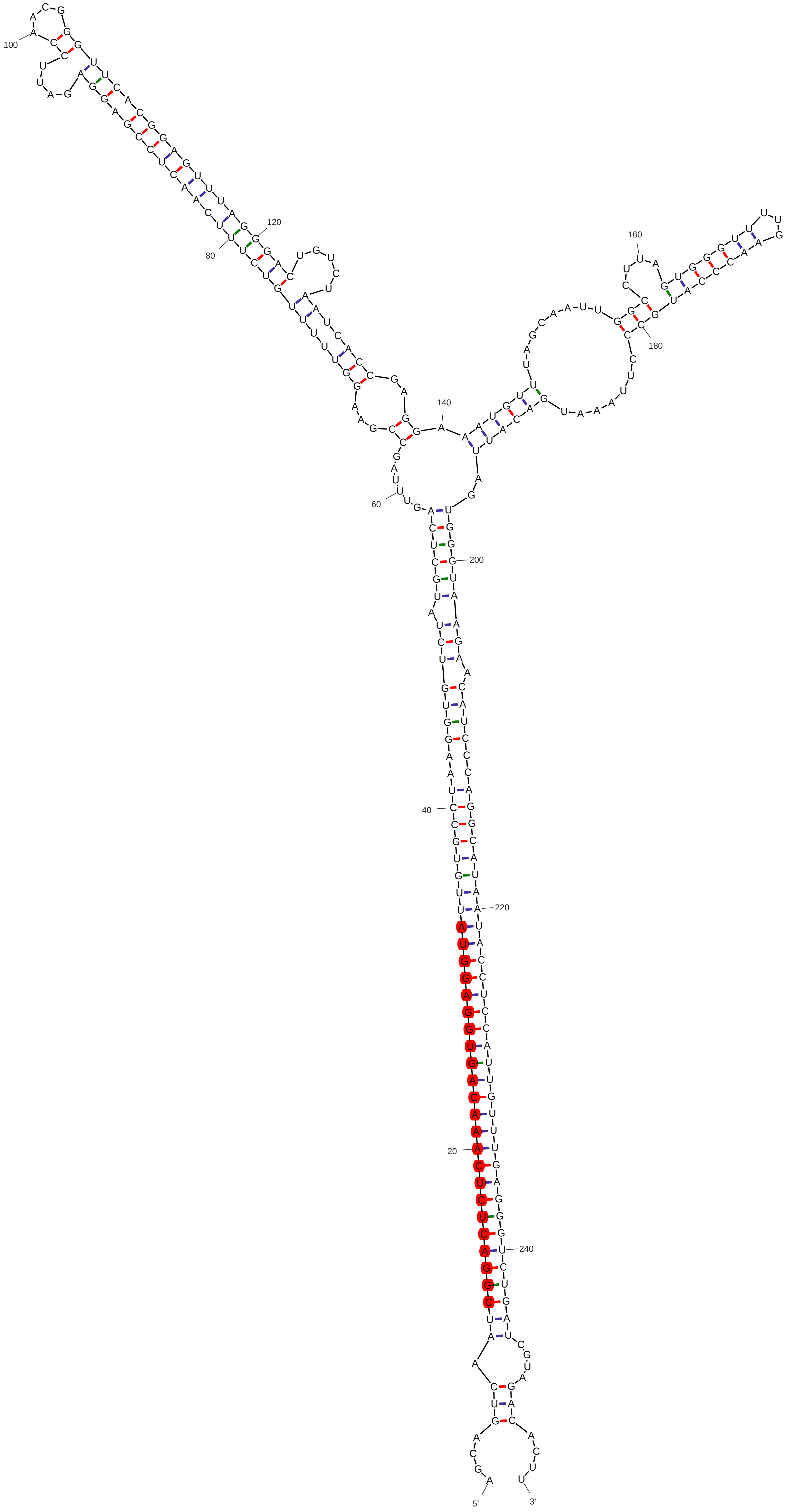

dG = -105.35 [Initially -108.00] novel_mir_4047

Output of sir_graph (©)
mfold_util 4.6

Created Sun Jan 13 08:43:43 2013

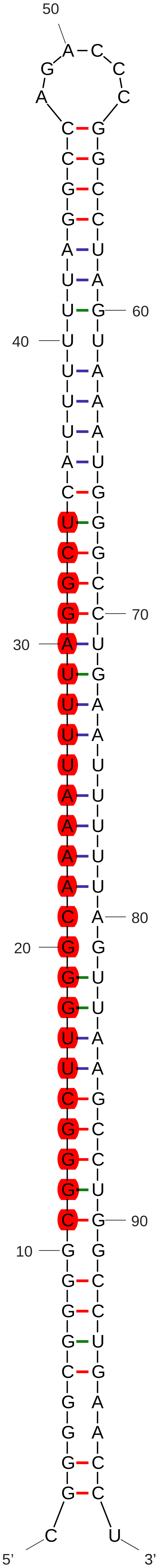

dG = -58.70 [Initially -58.70] novel_mir_5106

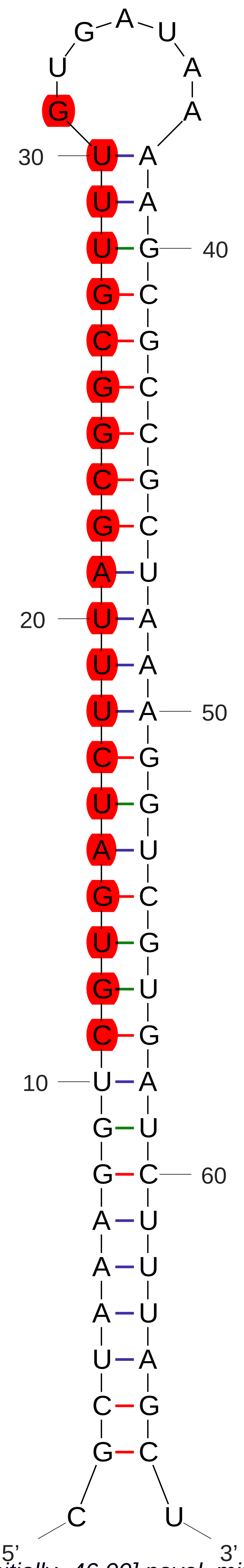

dG = -46.00 [Initially -46.00] novel_mir_2504_1

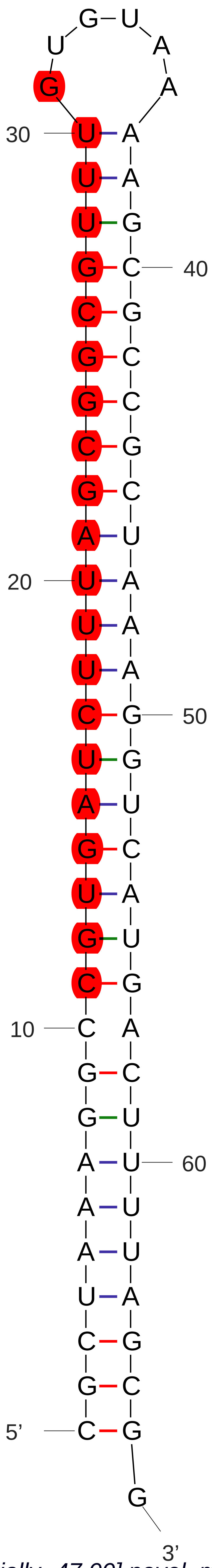

dG = -47.00 [Initially -47.00] novel_mir_2504_2

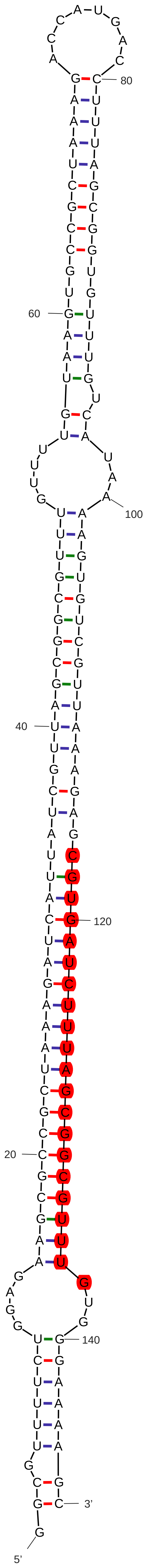

dG = -69.30 [Initially -69.30] novel_mir_2504_3

Output of sir_graph (©)
mfold_util 4.6

Created Sun Jan 13 08:48:29 2013

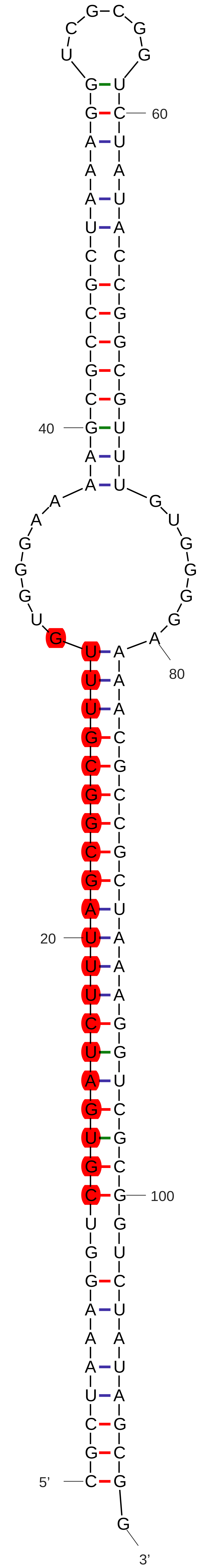

dG = -63.30 [Initially -63.30] novel_mir_2504_4

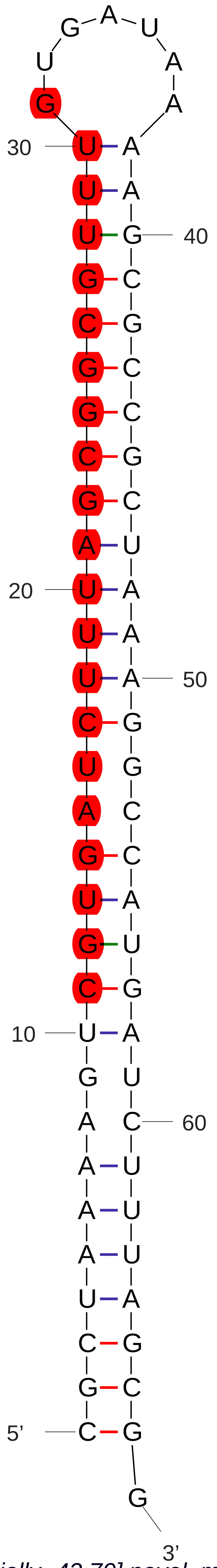

dG = -42.70 [Initially -42.70] novel_mir_2504_5

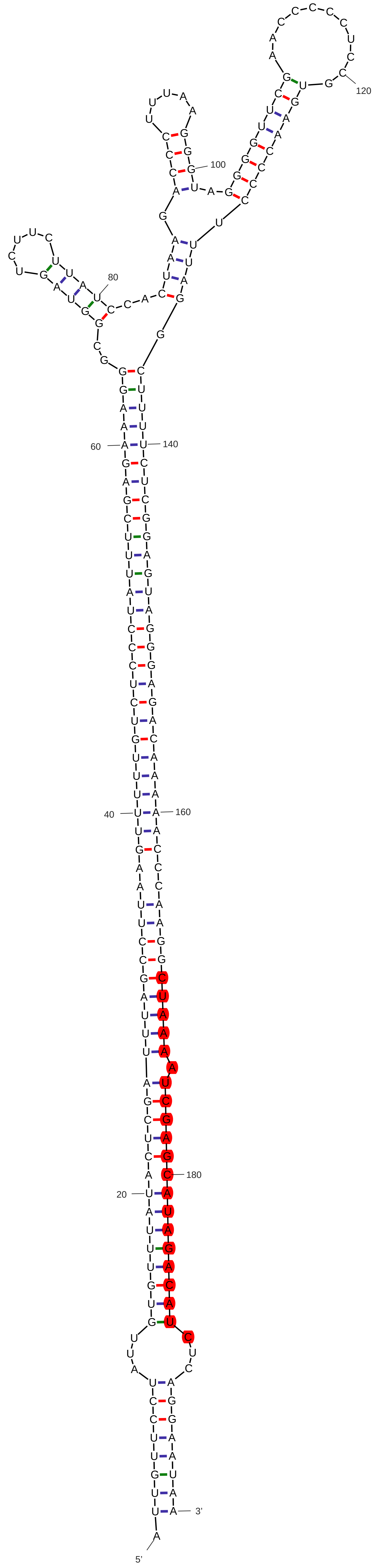

dG = -94.90 [Initially -98.40] novel_mir_2609

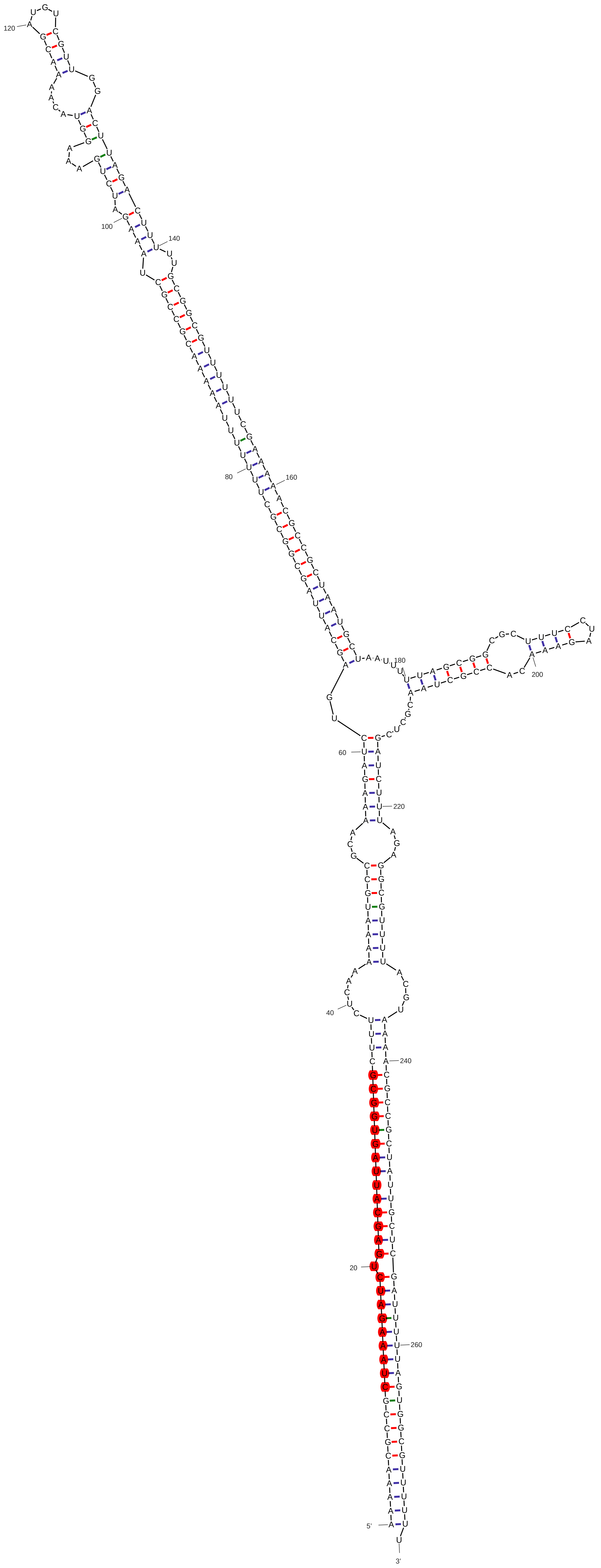

dG = -127.84 [Initially -131.50] novel_mir_2518

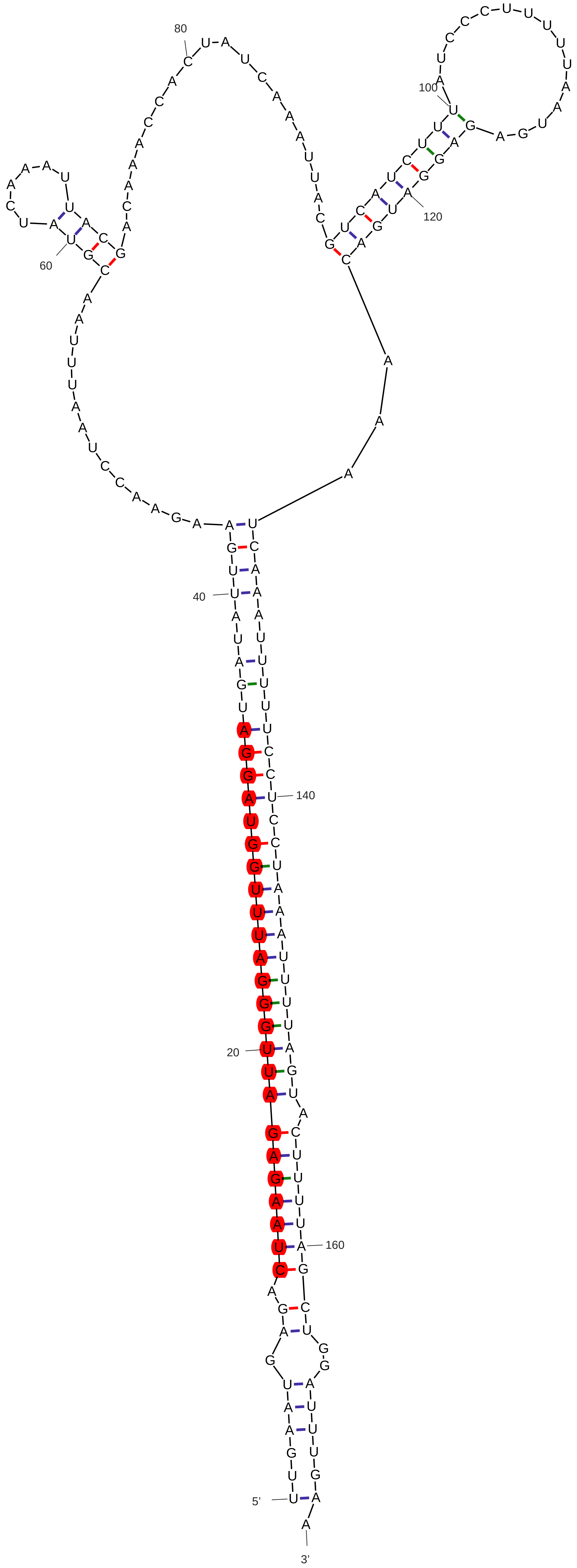

dG = -34.00 [Initially -39.40] novel_mir_2484

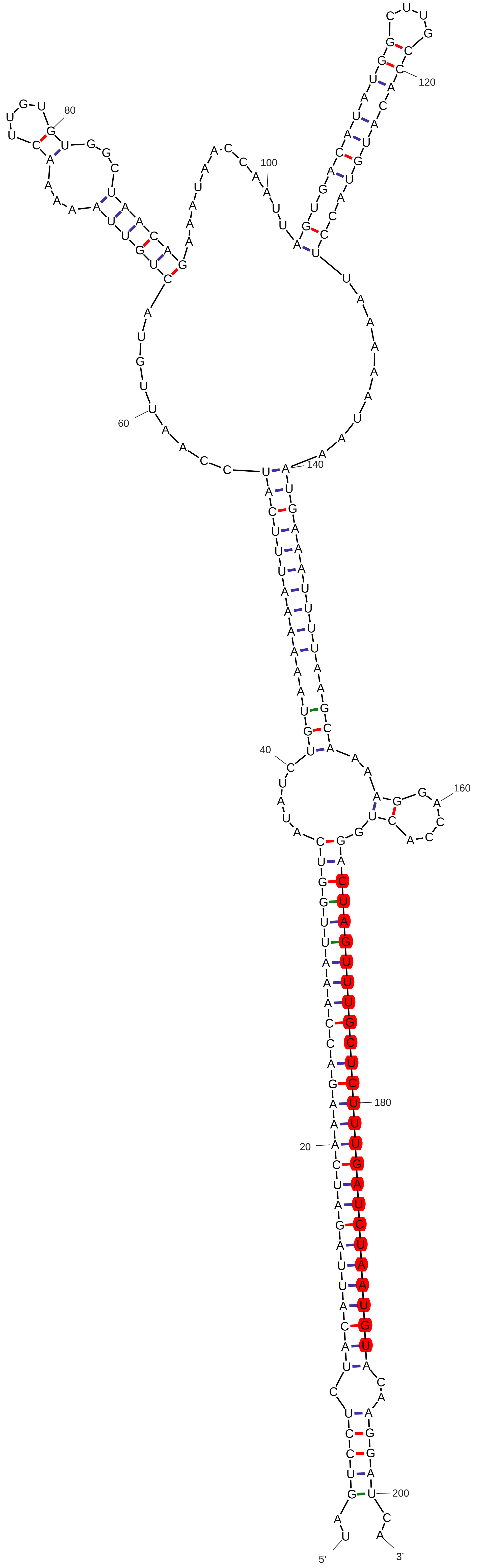

dG = -55.61 [Initially -61.80] novel_mir_5031

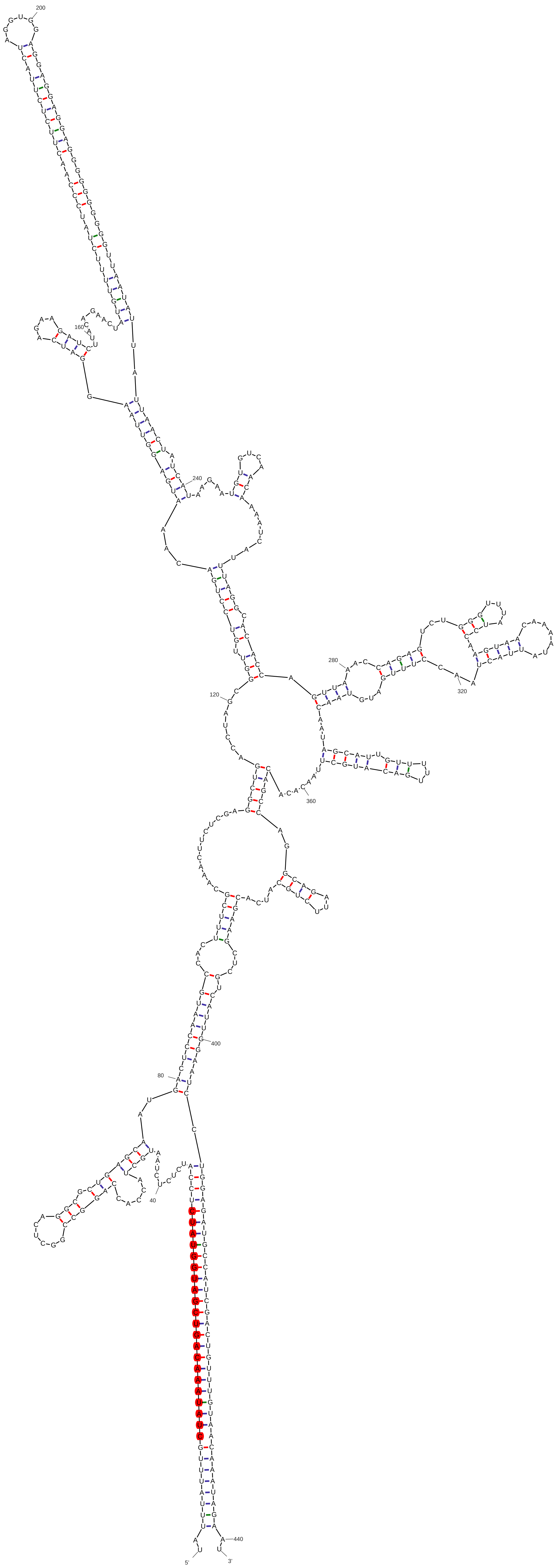

dG = -122.19 [Initially -142.30] novel_mir_4080

Output of sir_graph (©)
mfold_util 4.6

Created Sun Jan 13 07:13:37 2013

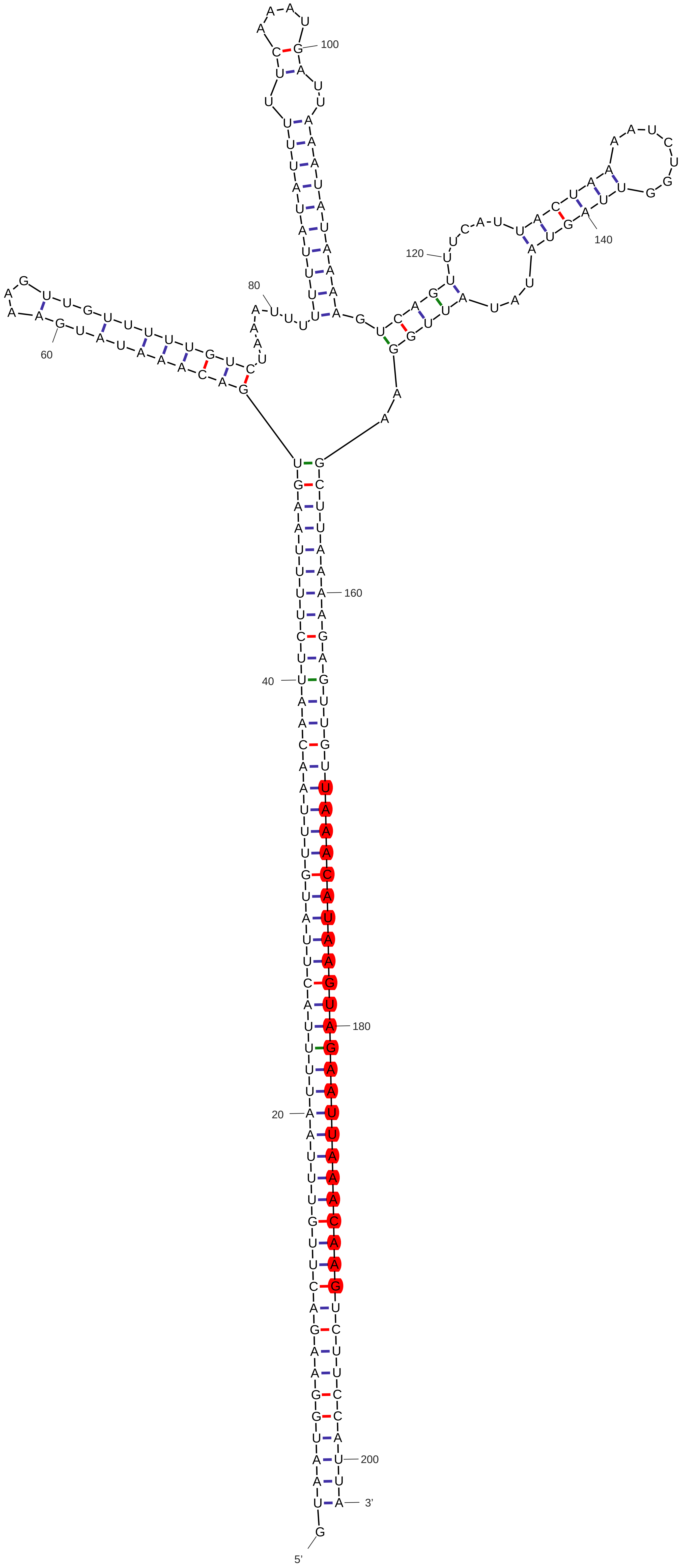

dG = -82.04 [Initially -81.80] novel_mir_824_1

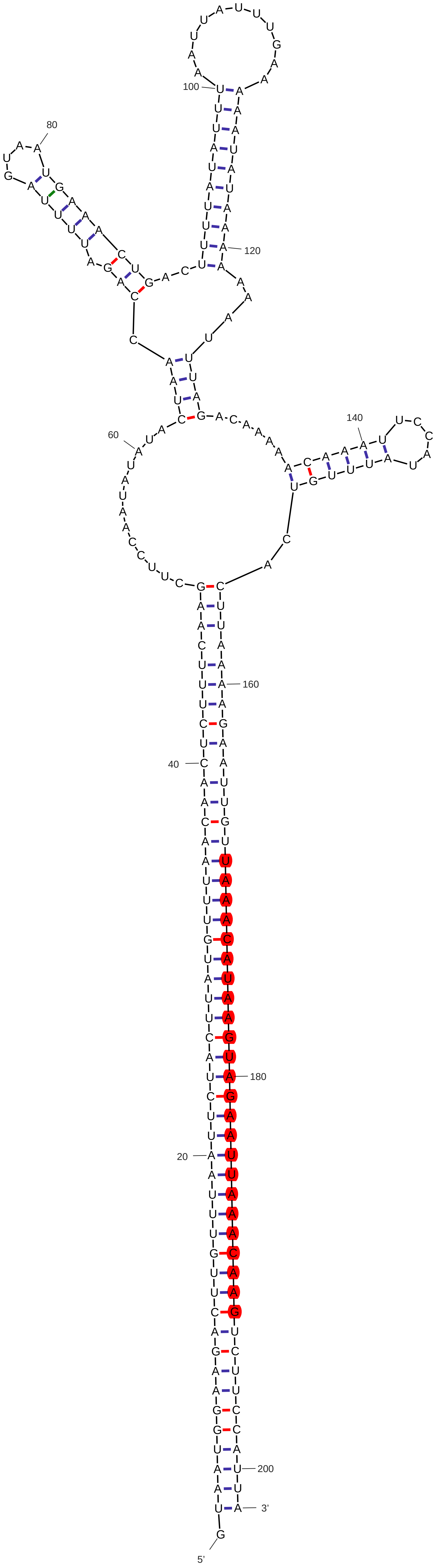

dG = -66.85 [Initially -73.00] novel_mir_824_2

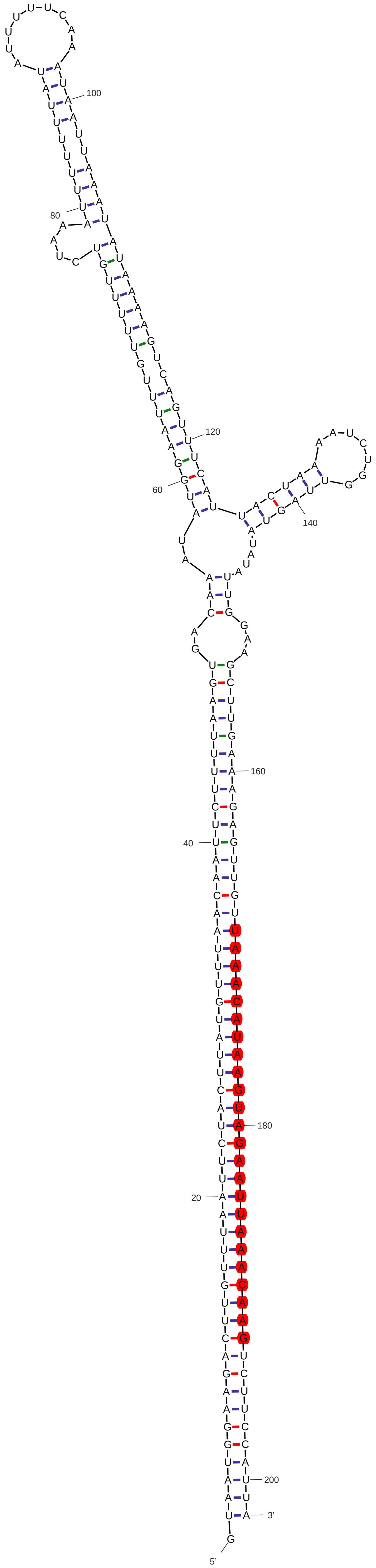

dG = -80.60 [Initially -83.10] novel_mir_824_3

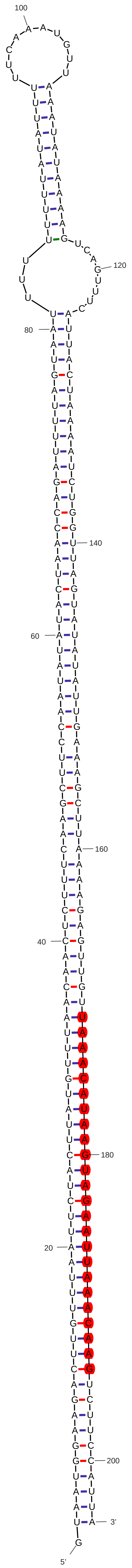

dG = -115.70 [Initially -115.70] novel_mir_824_4

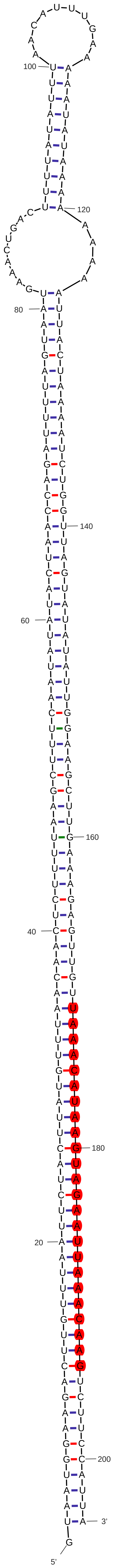

dG = -122.90 [Initially -122.90] novel_mir_824_5

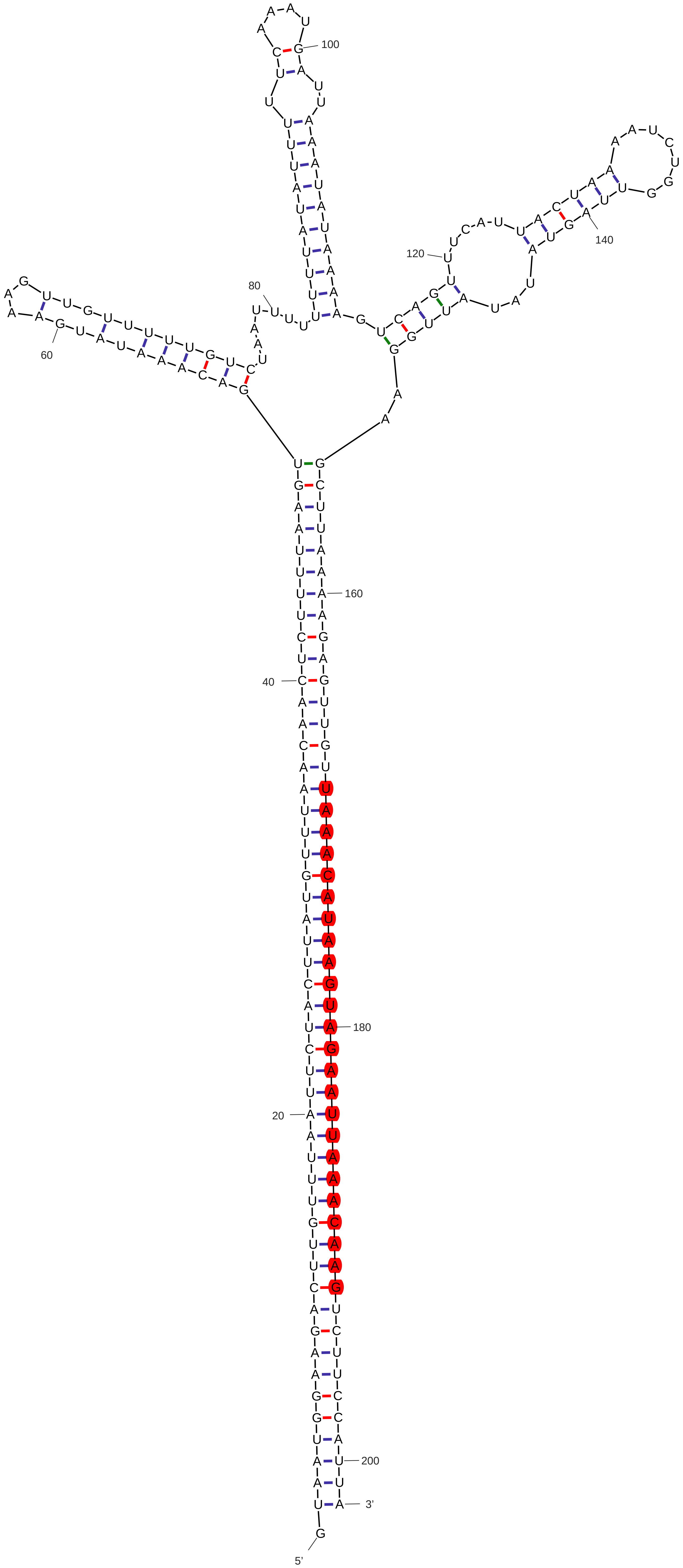

dG = -86.94 [Initially -86.70] novel_mir_824_6

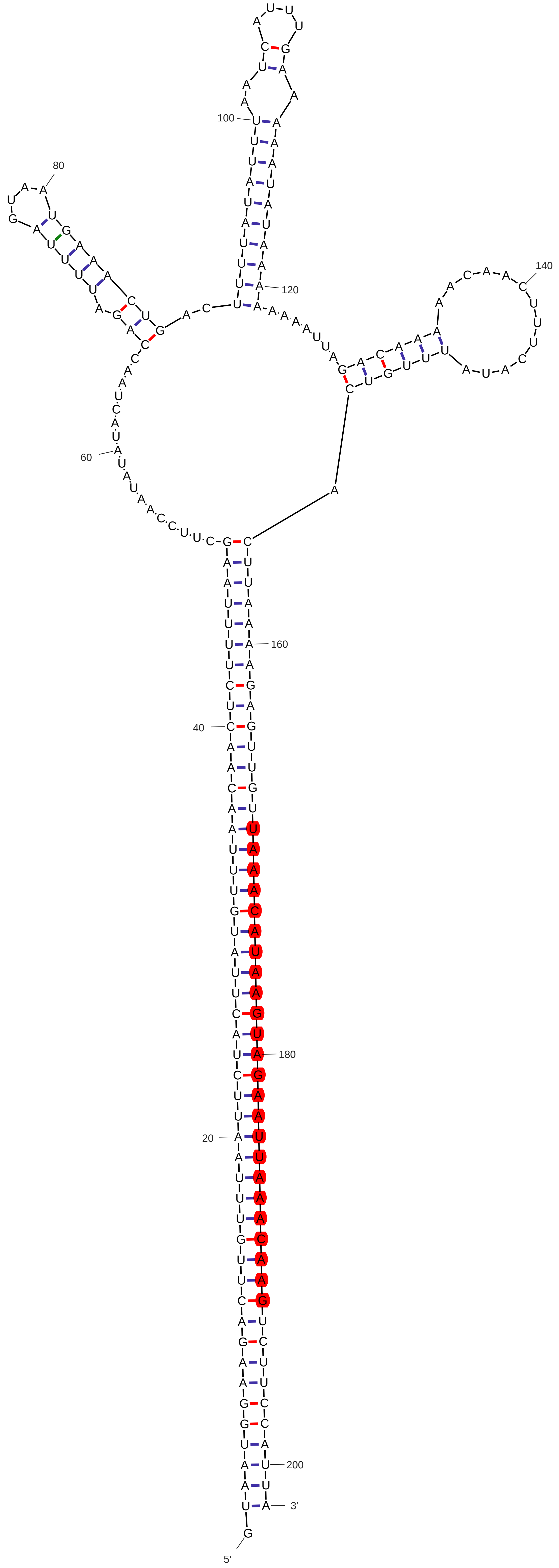

dG = -80.11 [Initially -83.20] novel_mir_824_7

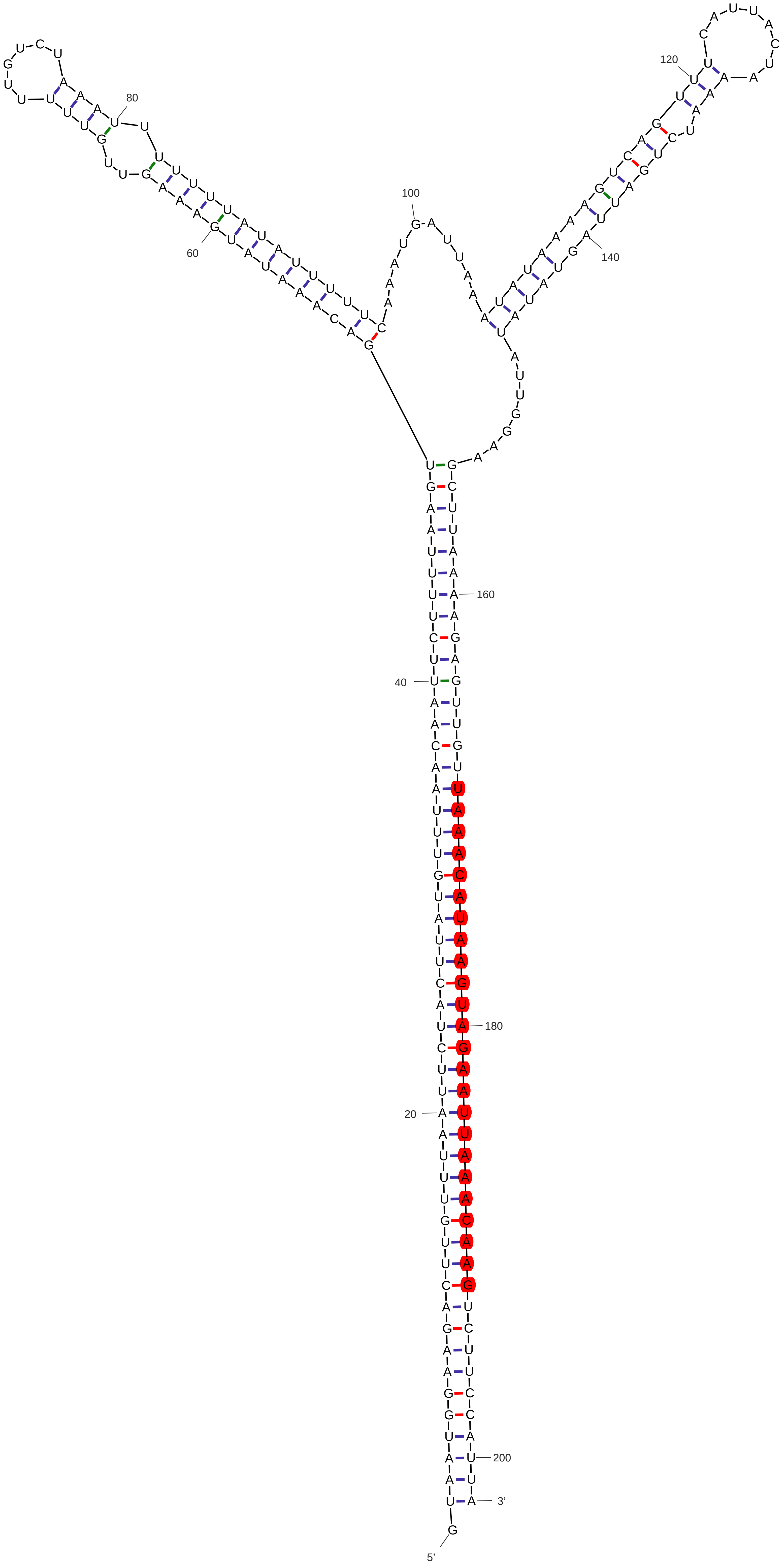

dG = -81.85 [Initially -85.30] novel_mir_824_8

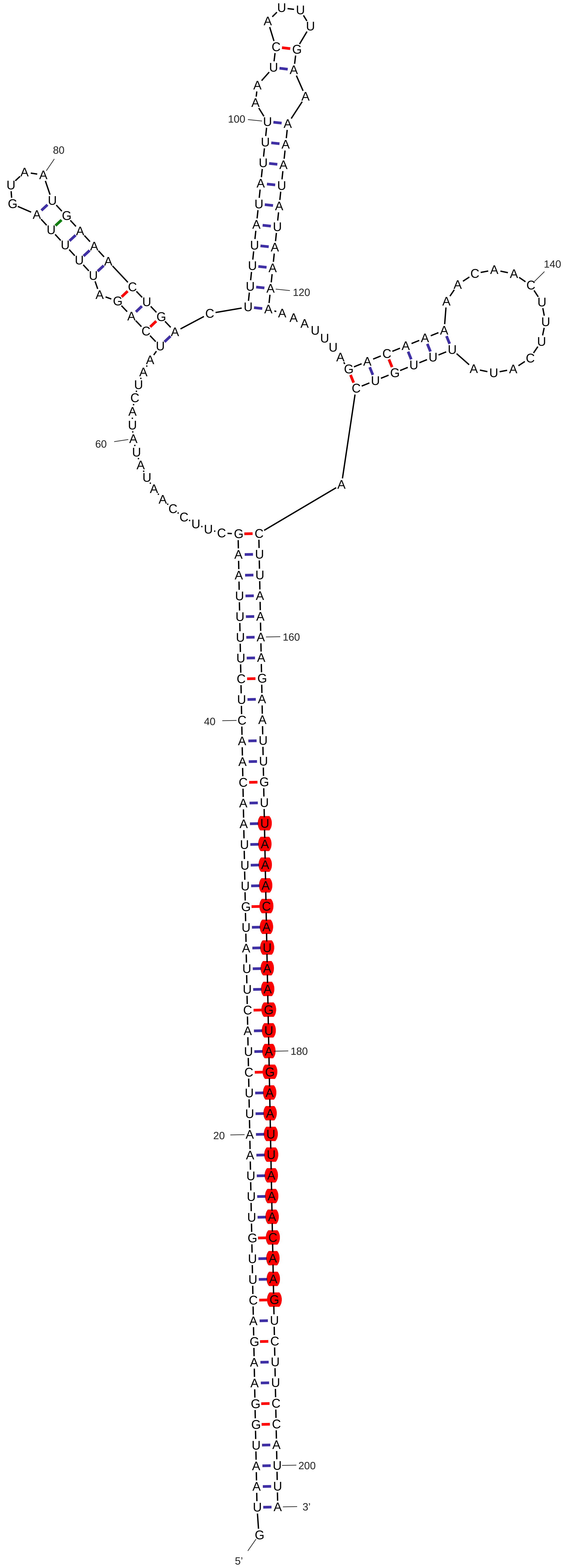

dG = -76.69 [Initially -78.40] novel_mir_824_9

Output of sir_graph (©)
mfold_util 4.6

Created Sun Jan 13 08:51:38 2013

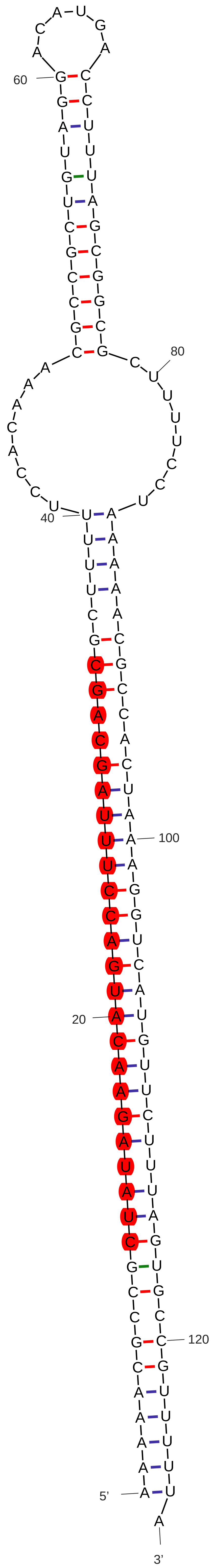

dG = -62.70 [Initially -62.70] novel_mir_67_1

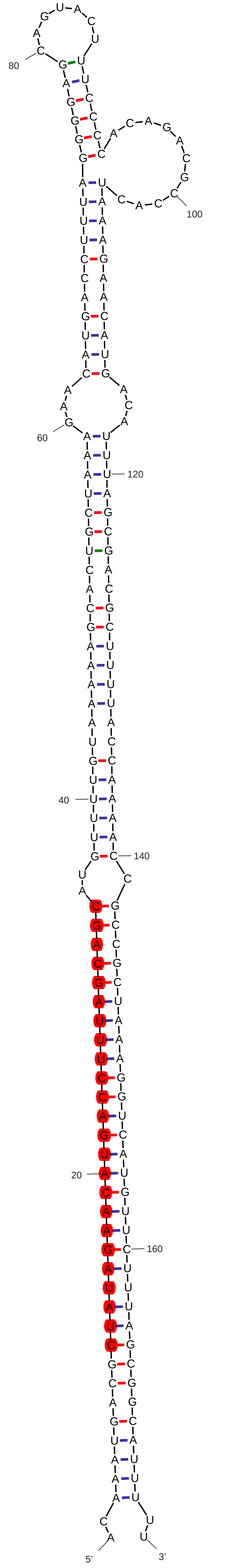

dG = -75.40 [Initially -75.40] novel_mir_67_2

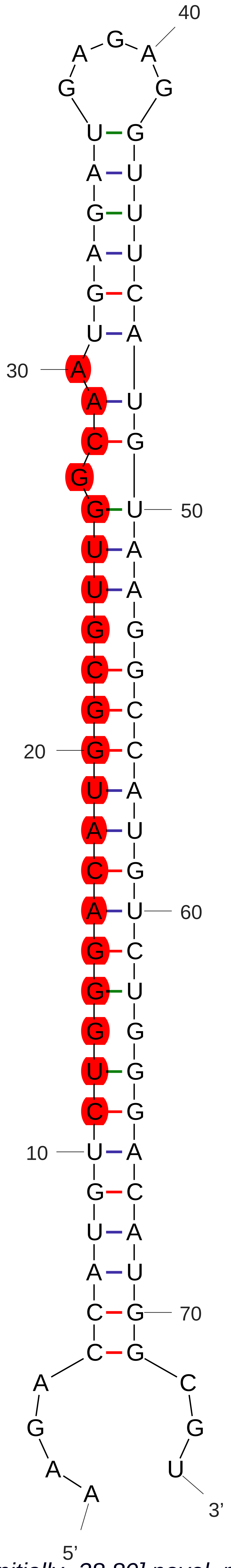

dG = -38.80 [Initially -38.80] novel_mir_4073

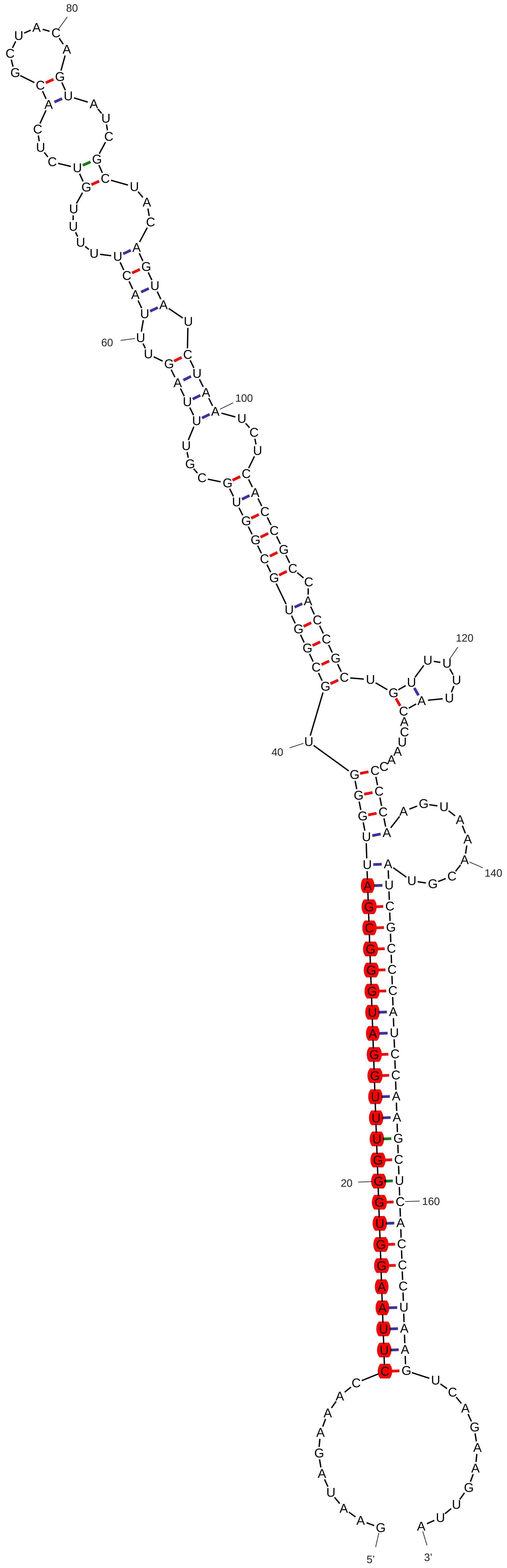

dG = -66.98 [Initially -70.00] novel_mir_1179

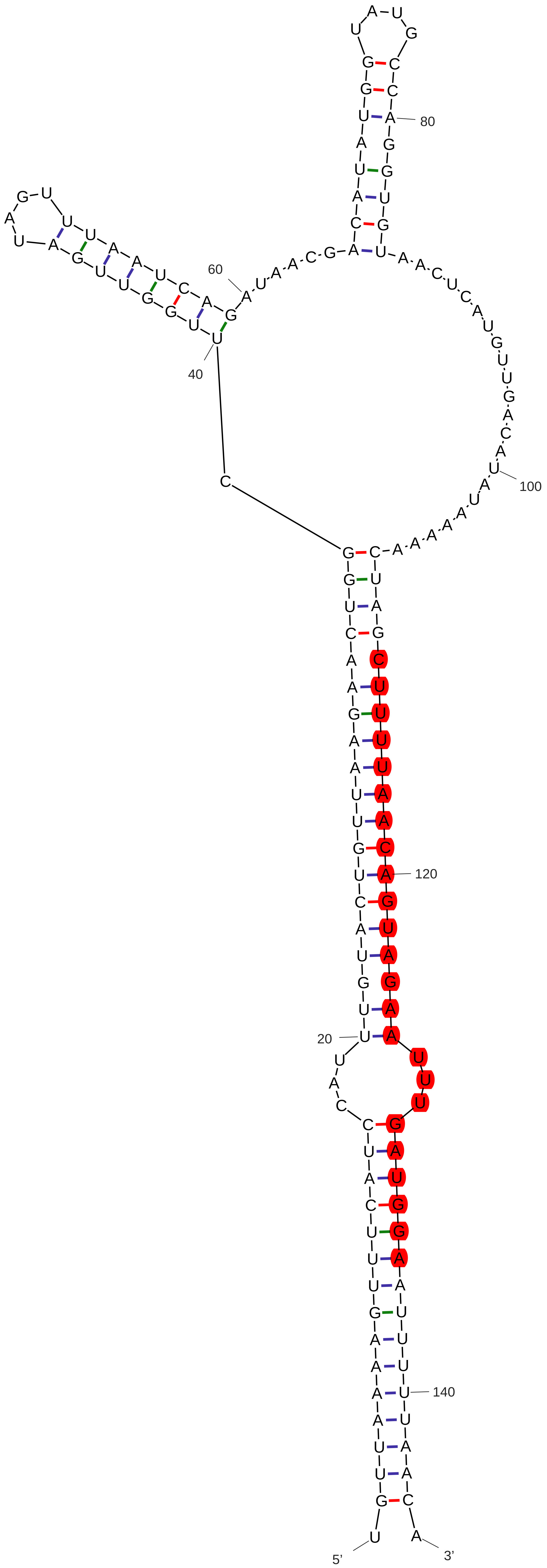

dG = -42.47 [Initially -45.90] novel_mir_3369

Output of sir_graph (©)
mfold_util 4.6

Created Sun Jan 13 08:52:27 2013

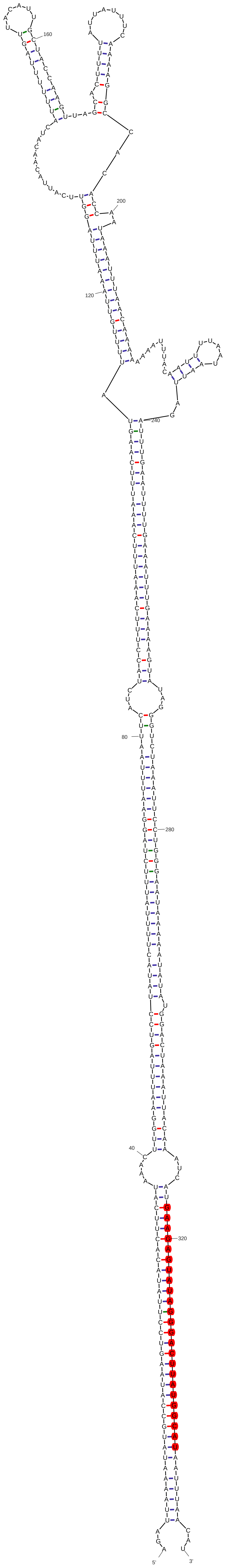

dG = -115.93 [Initially -121.80] novel_mir_977

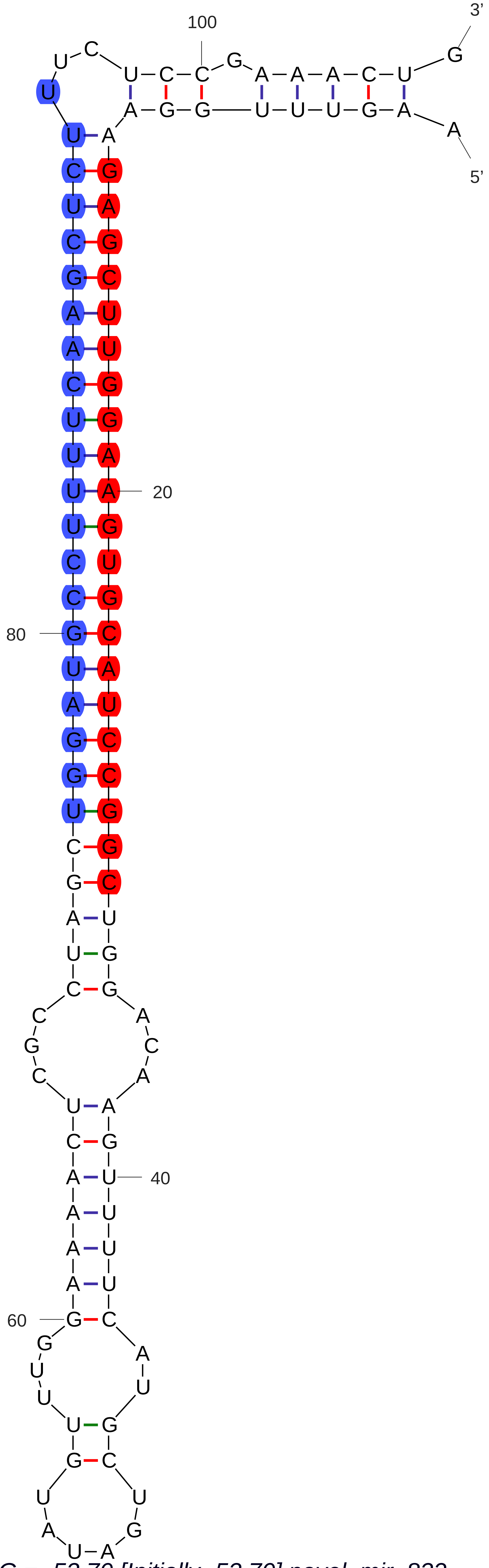

dG = -53.70 [Initially -53.70] novel_mir_832

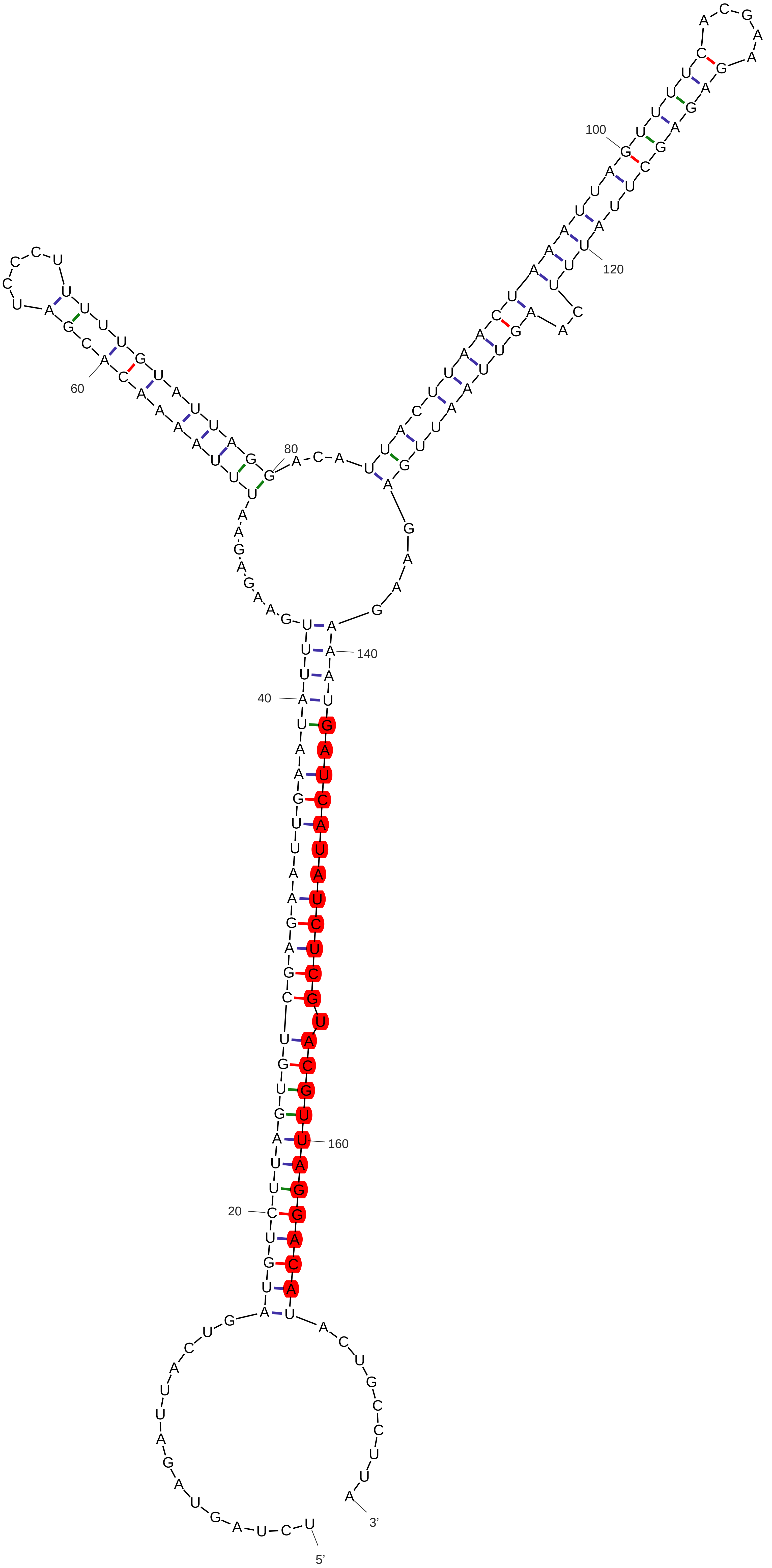

dG = -32.29 [Initially -36.10] novel_mir_1258

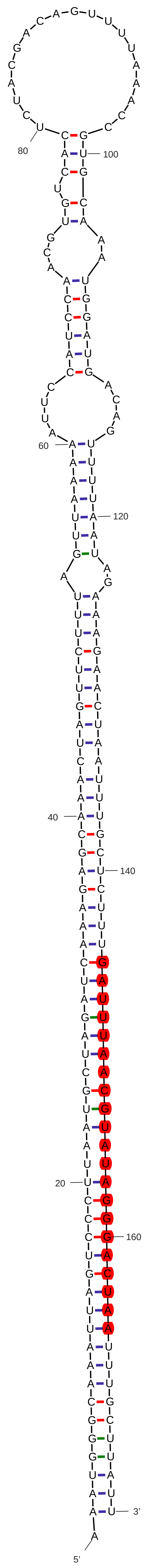

dG = -75.70 [Initially -75.70] novel_mir_2635

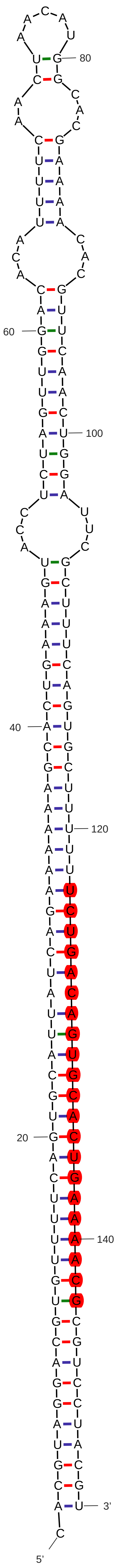

dG = -93.60 [Initially -93.60] novel_mir_147

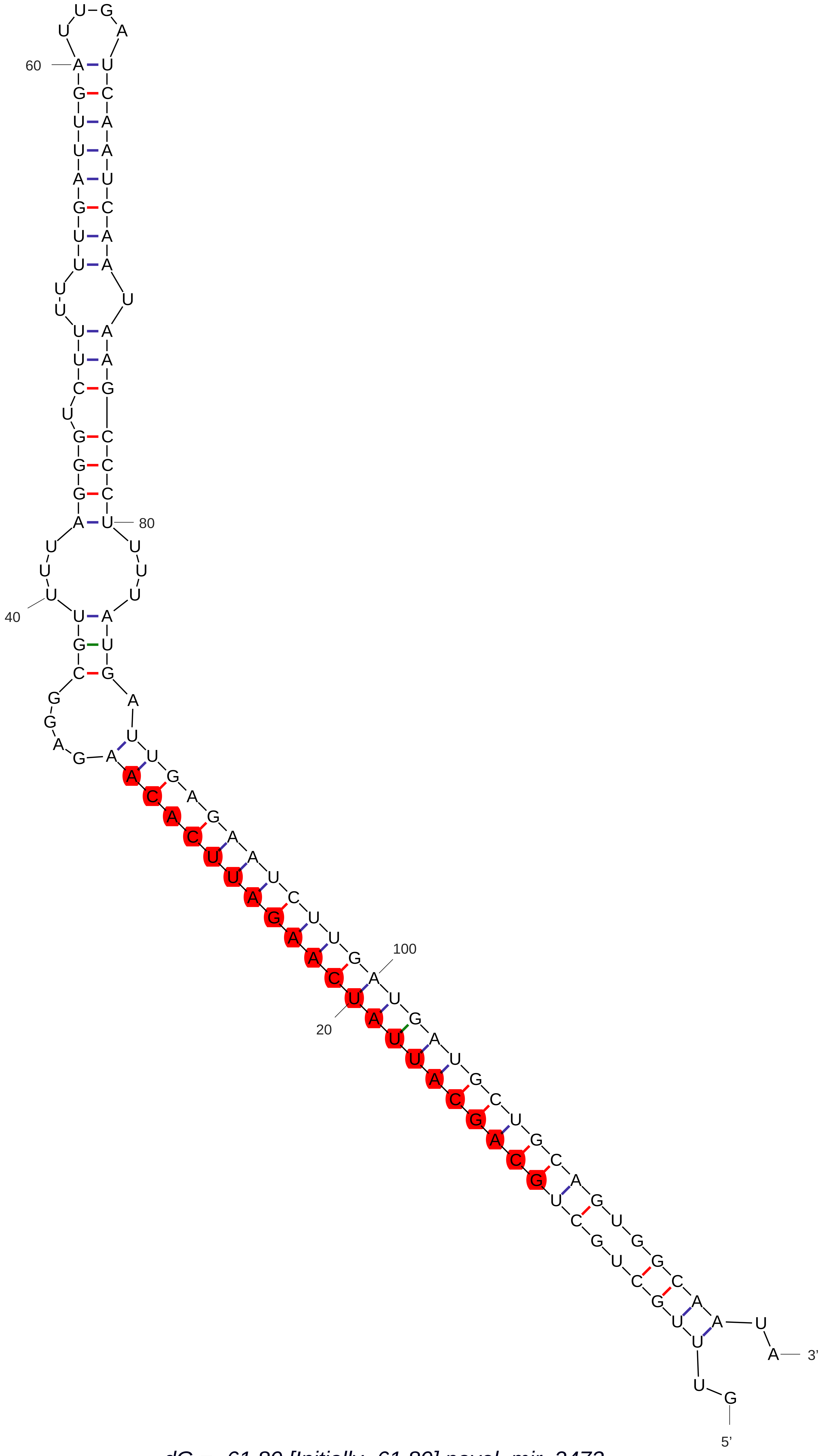

dG = -61.80 [Initially -61.80] novel_mir_2473

Output of sir_graph (©)
mfold_util 4.6

Created Sun Jan 13 09:31:22 2013

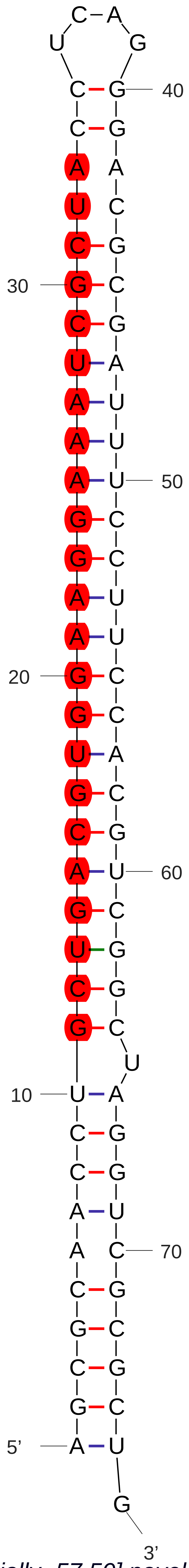

dG = -57.50 [Initially -57.50] novel_mir_2721

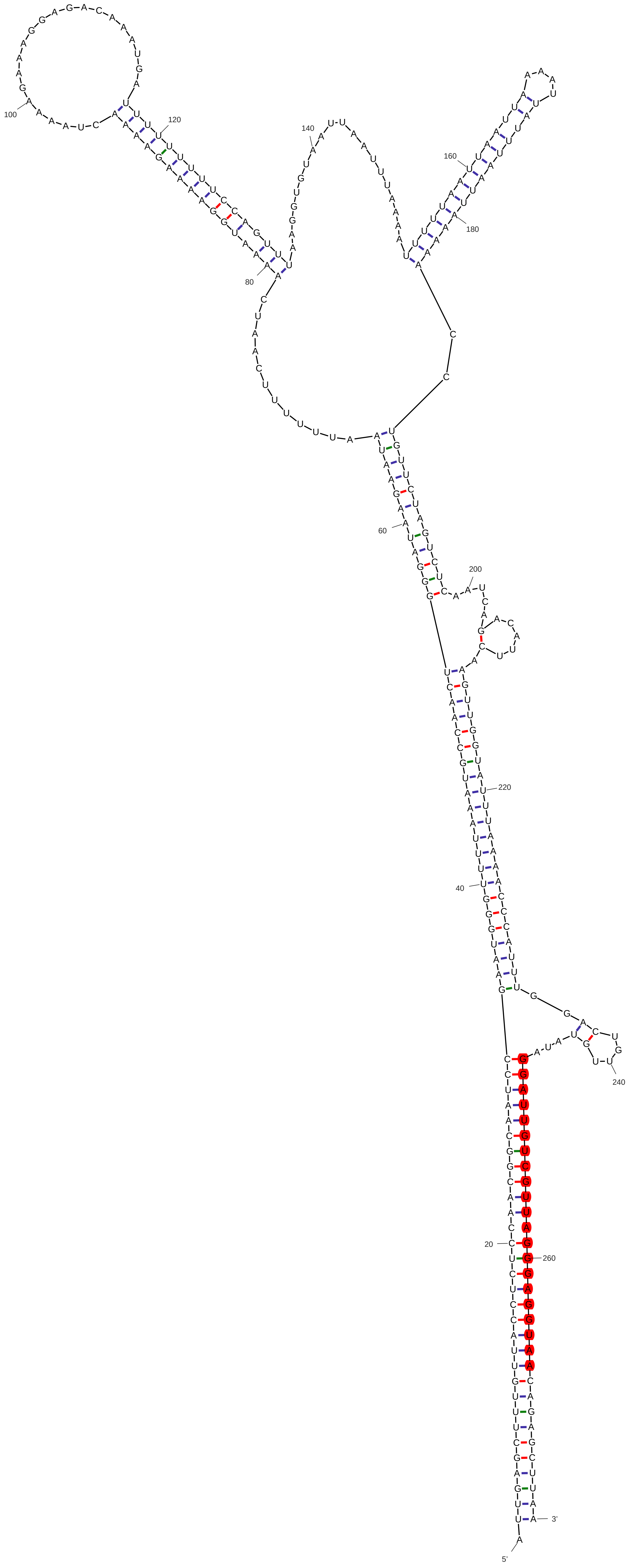

dG = -89.69 [Initially -98.90] novel_mir_5687_1

Output of sir_graph (©)
mfold_util 4.6

Created Sun Jan 13 09:31:37 2013

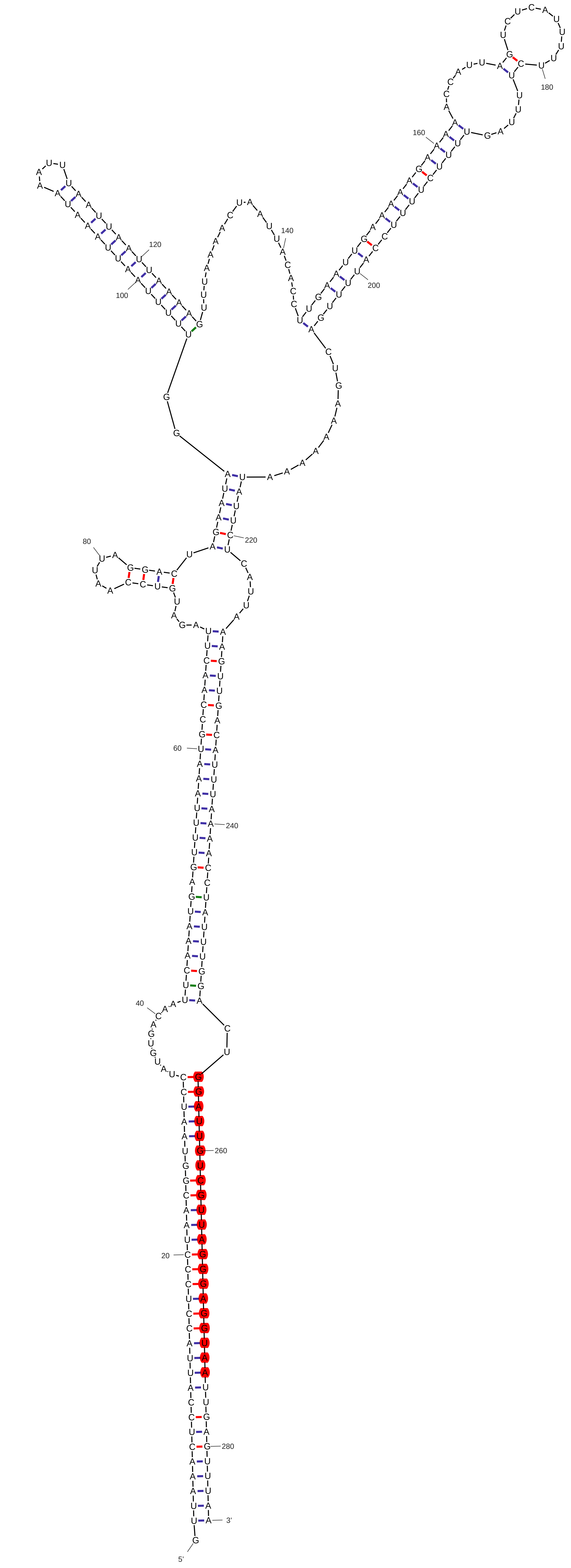

dG = -78.17 [Initially -85.60] novel_mir_5687_2

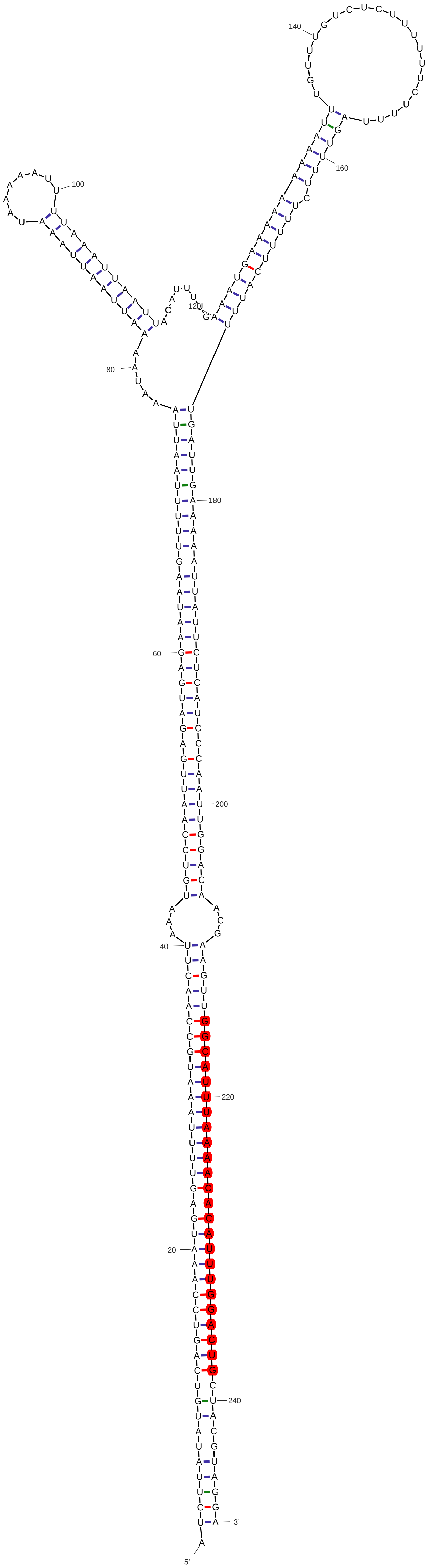

dG = -90.75 [Initially -93.90] novel_mir_5712

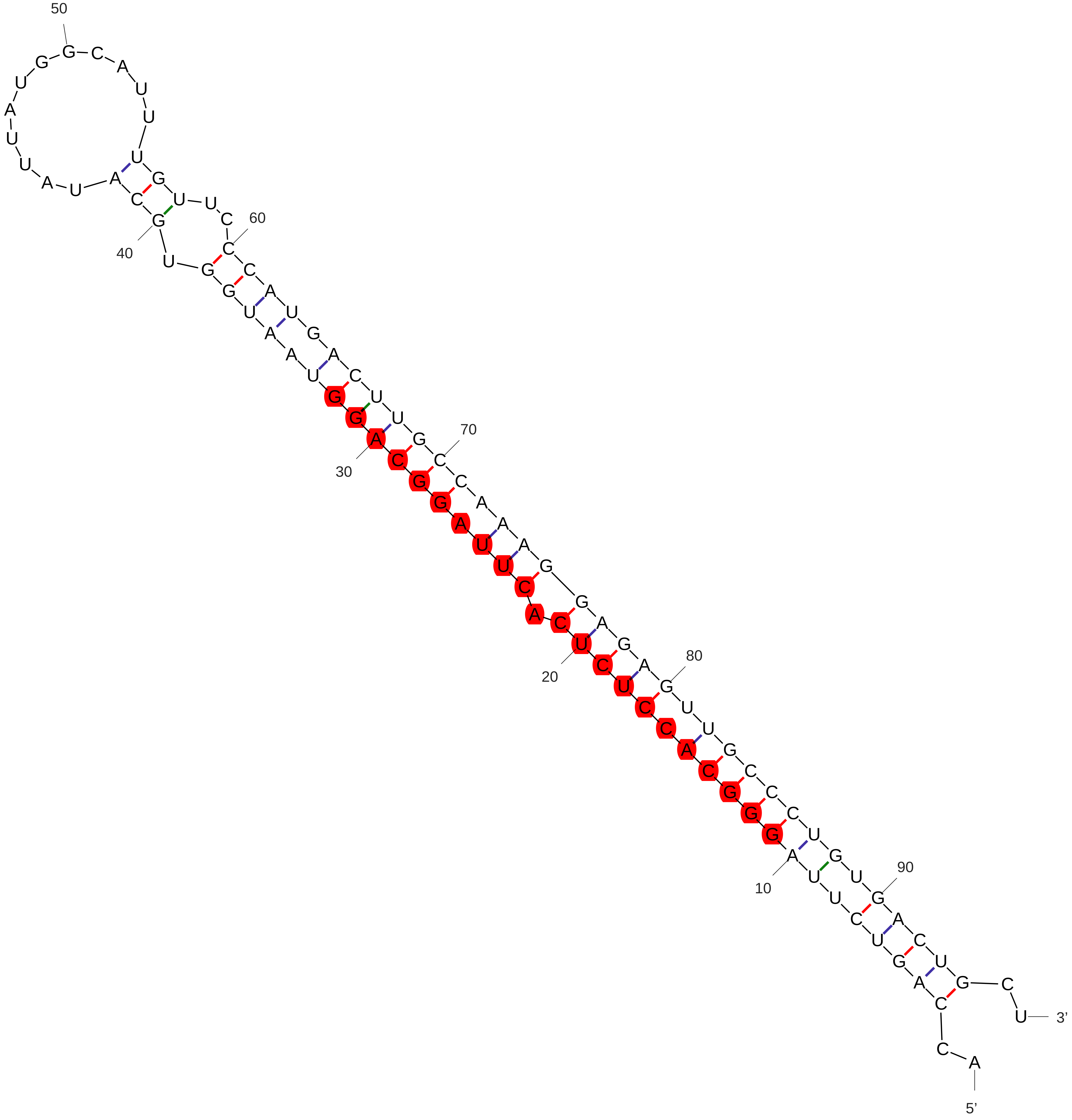

dG = -47.90 [Initially -47.90] novel_mir_4112

Output of sir_graph (©)
mfold_util 4.6

Created Sun Jan 13 09:35:36 2013

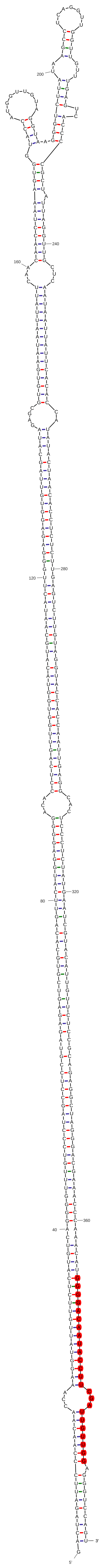

dG = -221.40 [Initially -223.40] novel_mir_995

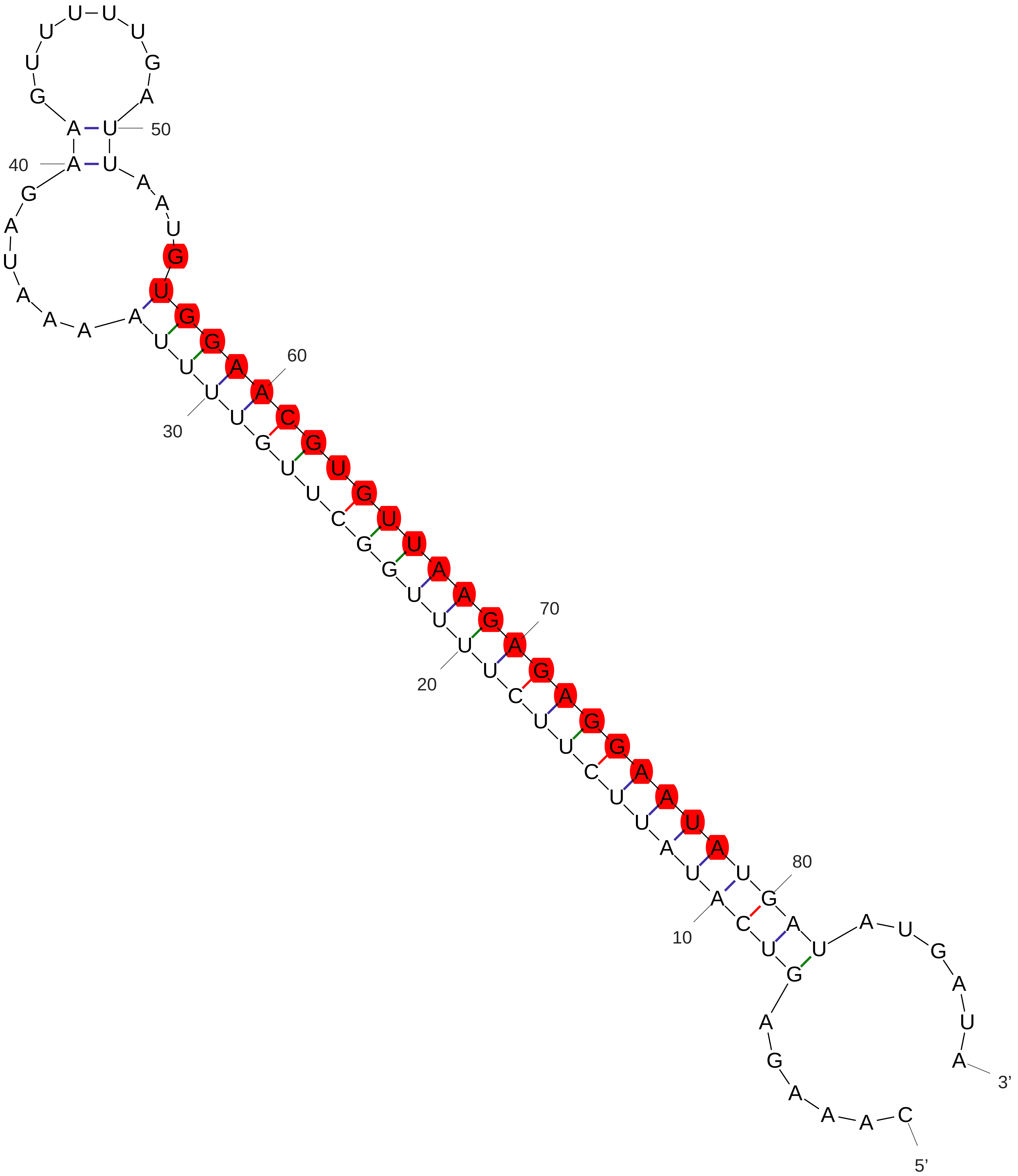

dG = -27.10 [Initially -27.10] novel_mir_4535

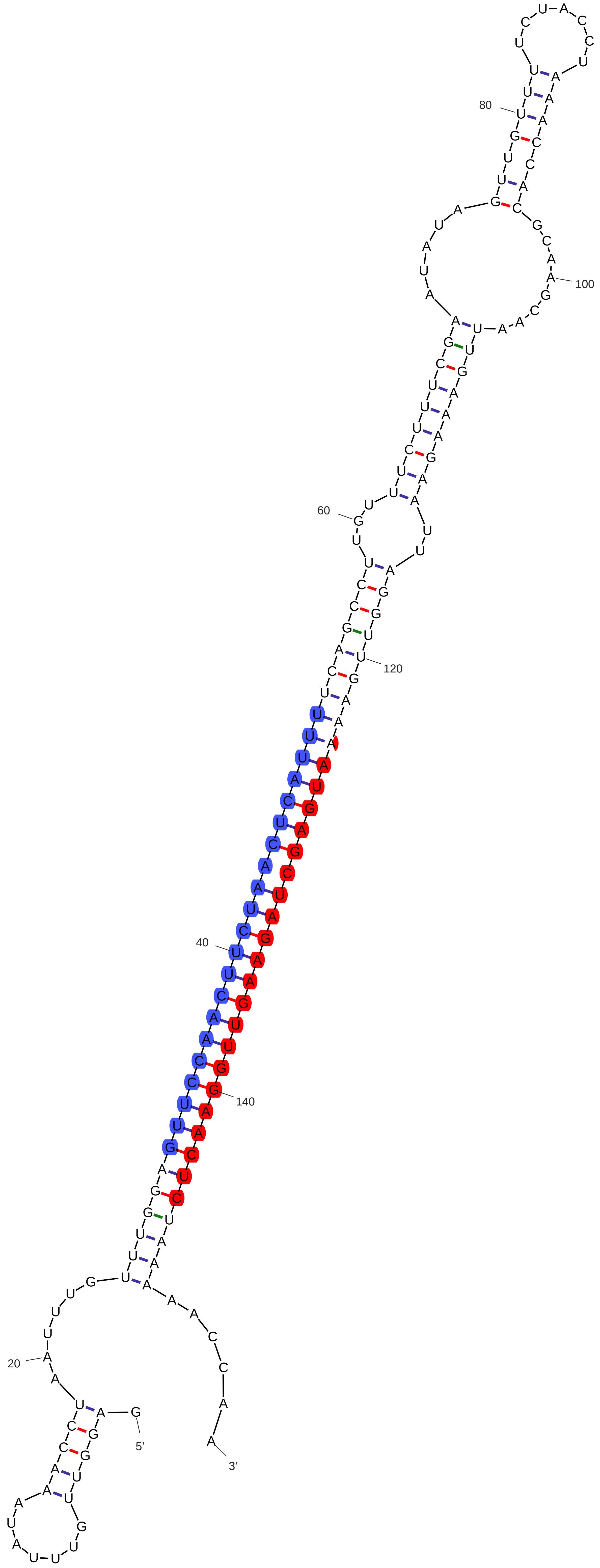

dG = -65.70 [Initially -65.70] GhmiRnB

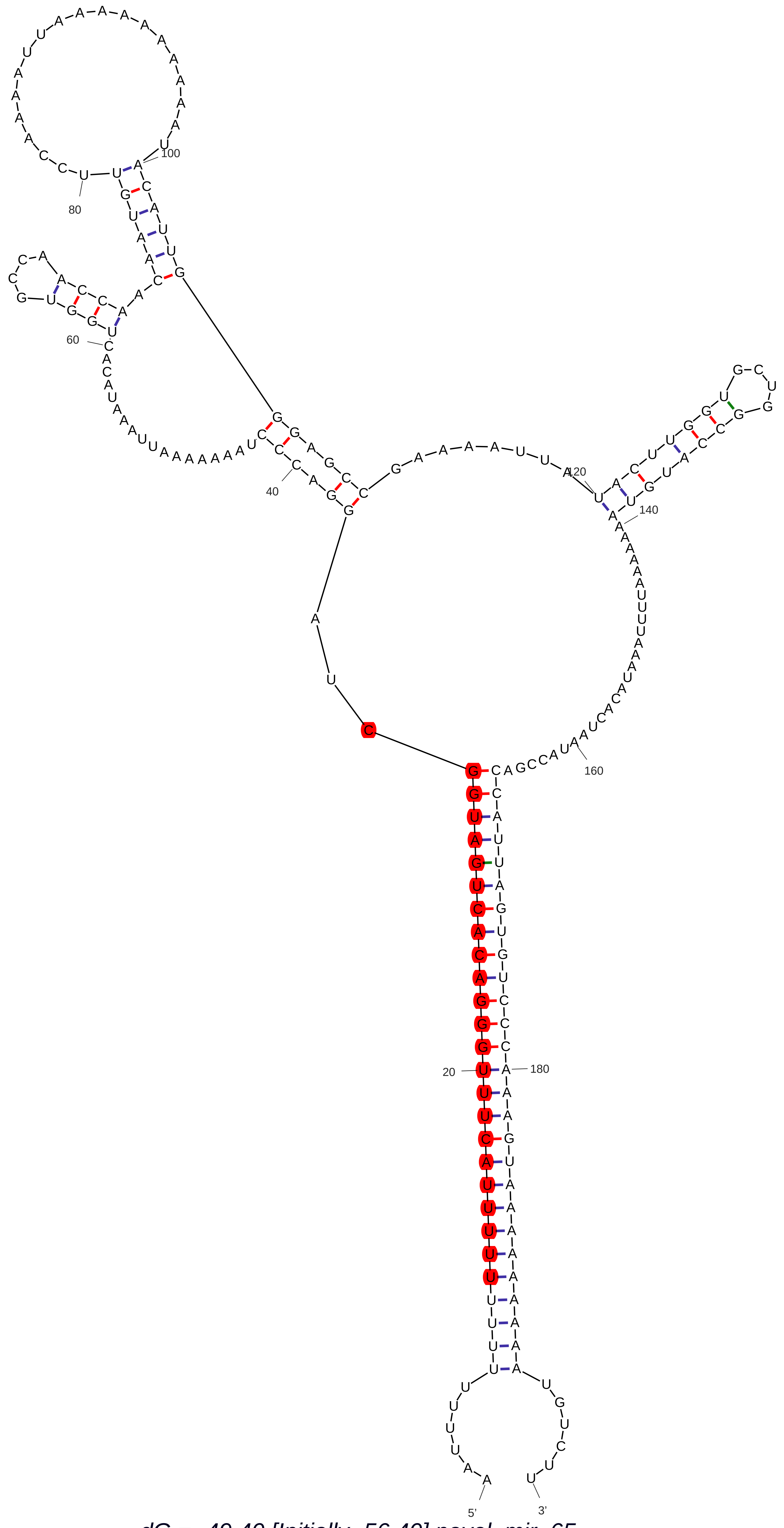

dG = -49.40 [Initially -56.40] novel_mir_65

Output of sir_graph (©)
mfold_util 4.6

Created Sun Jan 13 09:35:49 2013

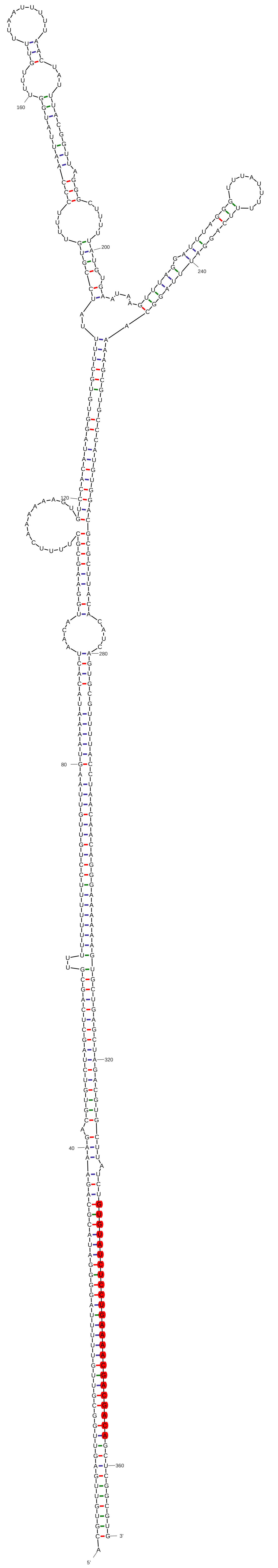

dG = -159.73 [Initially -161.90] novel_mir_2595

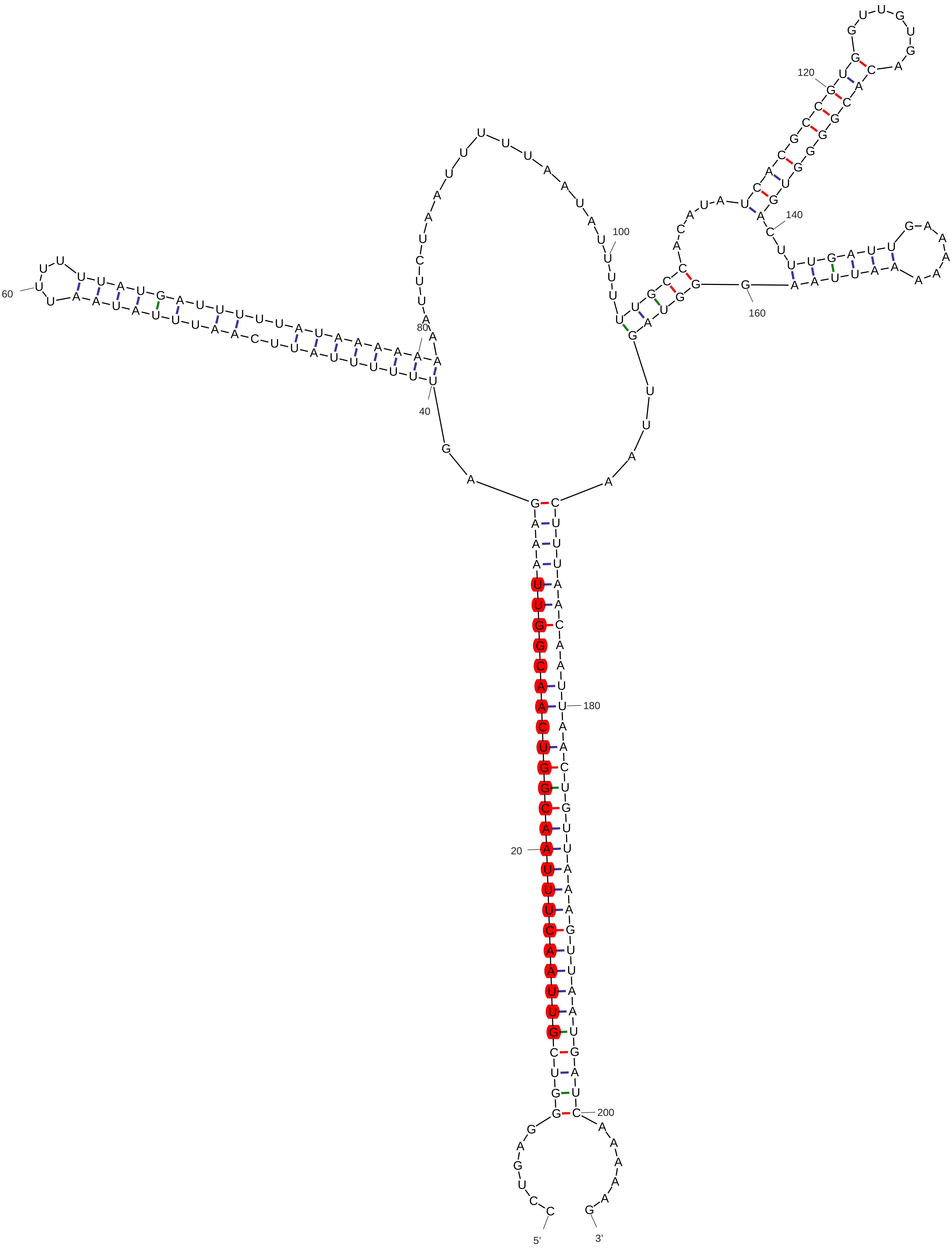

dG = -53.23 [Initially -60.80] novel_mir_2626_1

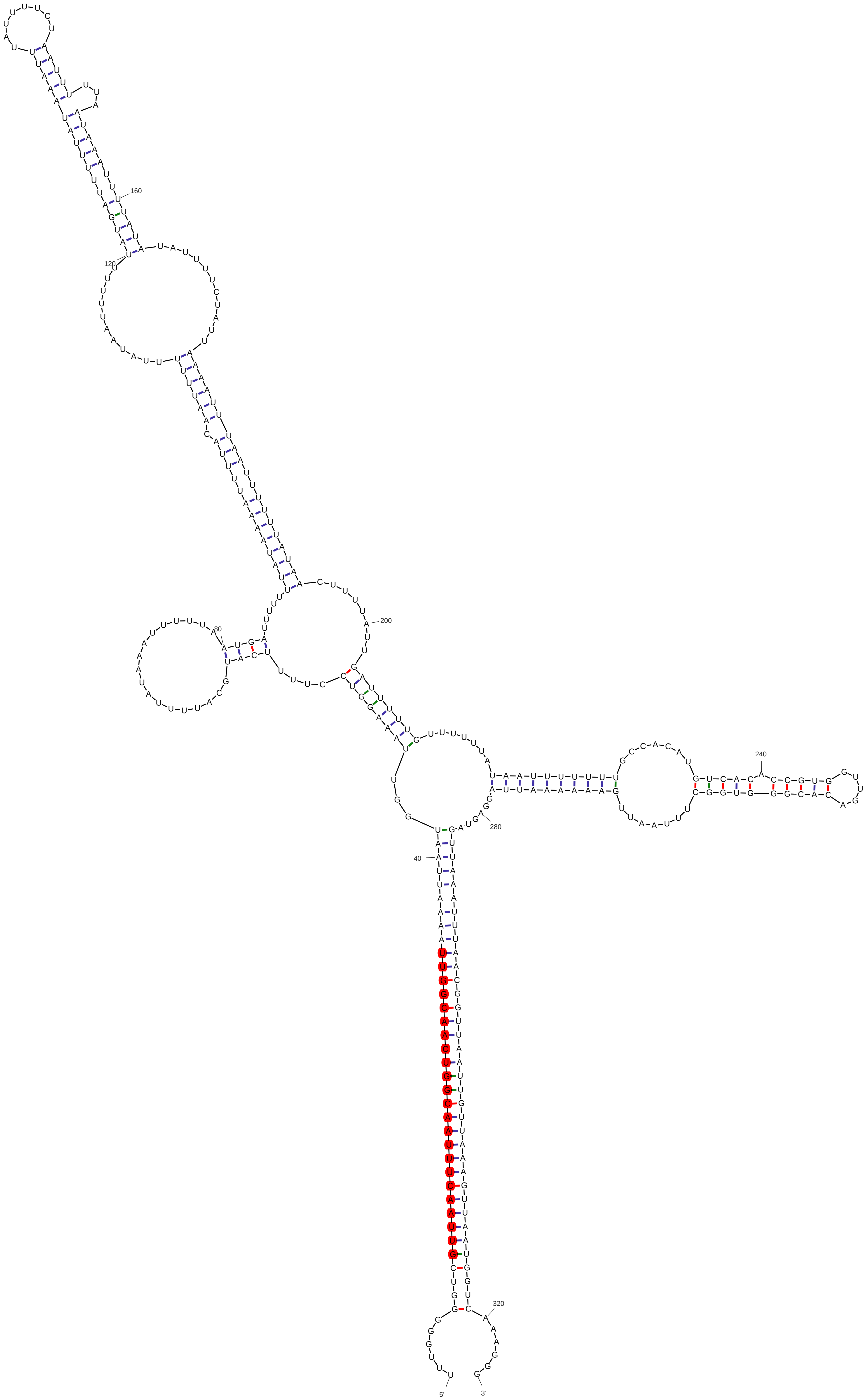

dG = -61.67 [Initially -69.60] novel_mir_2626_2

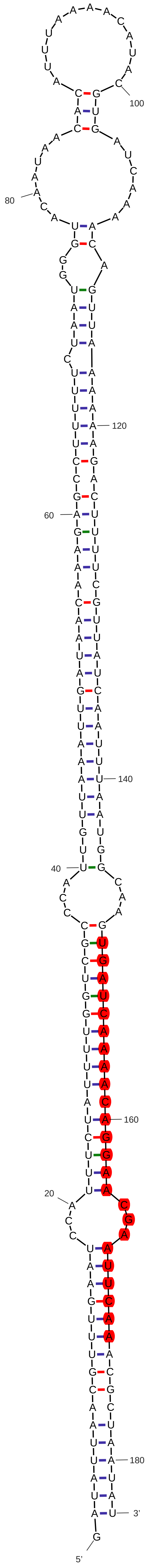

dG = -53.70 [Initially -53.70] novel_mir_950

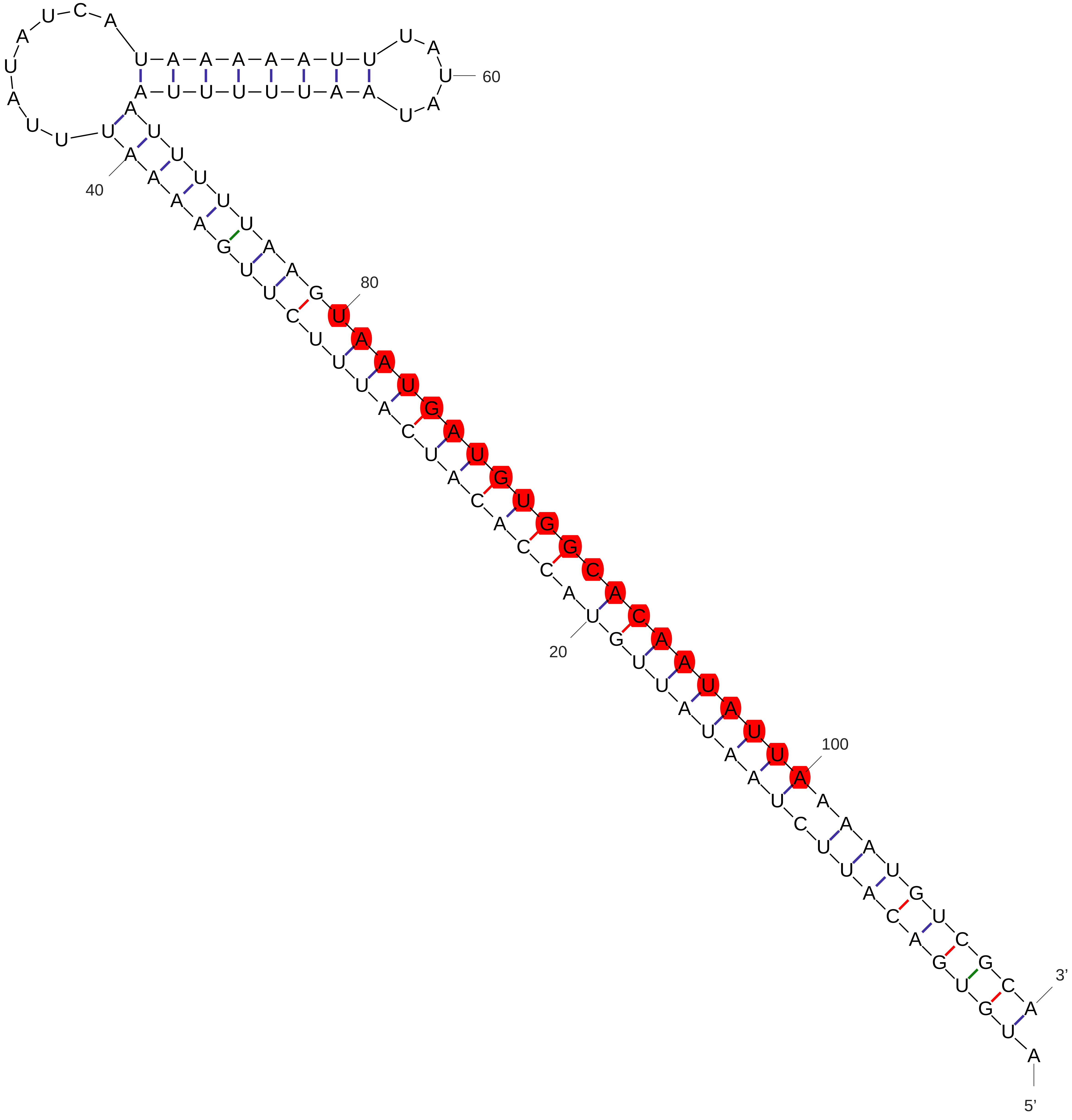

dG = -44.30 [Initially -44.30] novel_mir_4069

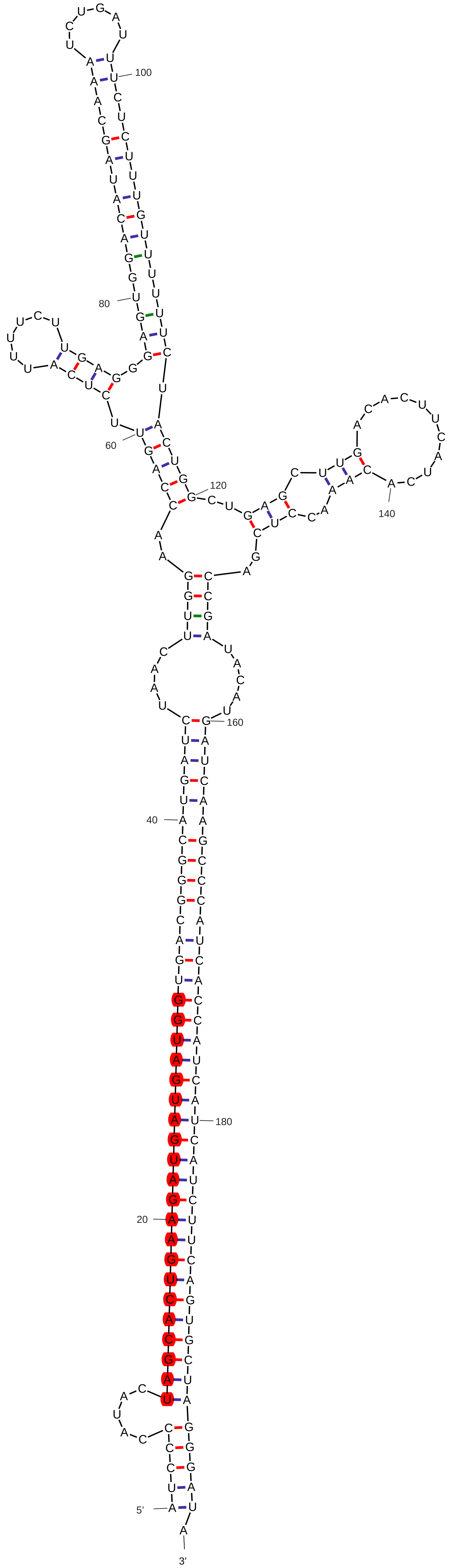

dG = -79.20 [Initially -83.90] novel_mir_92

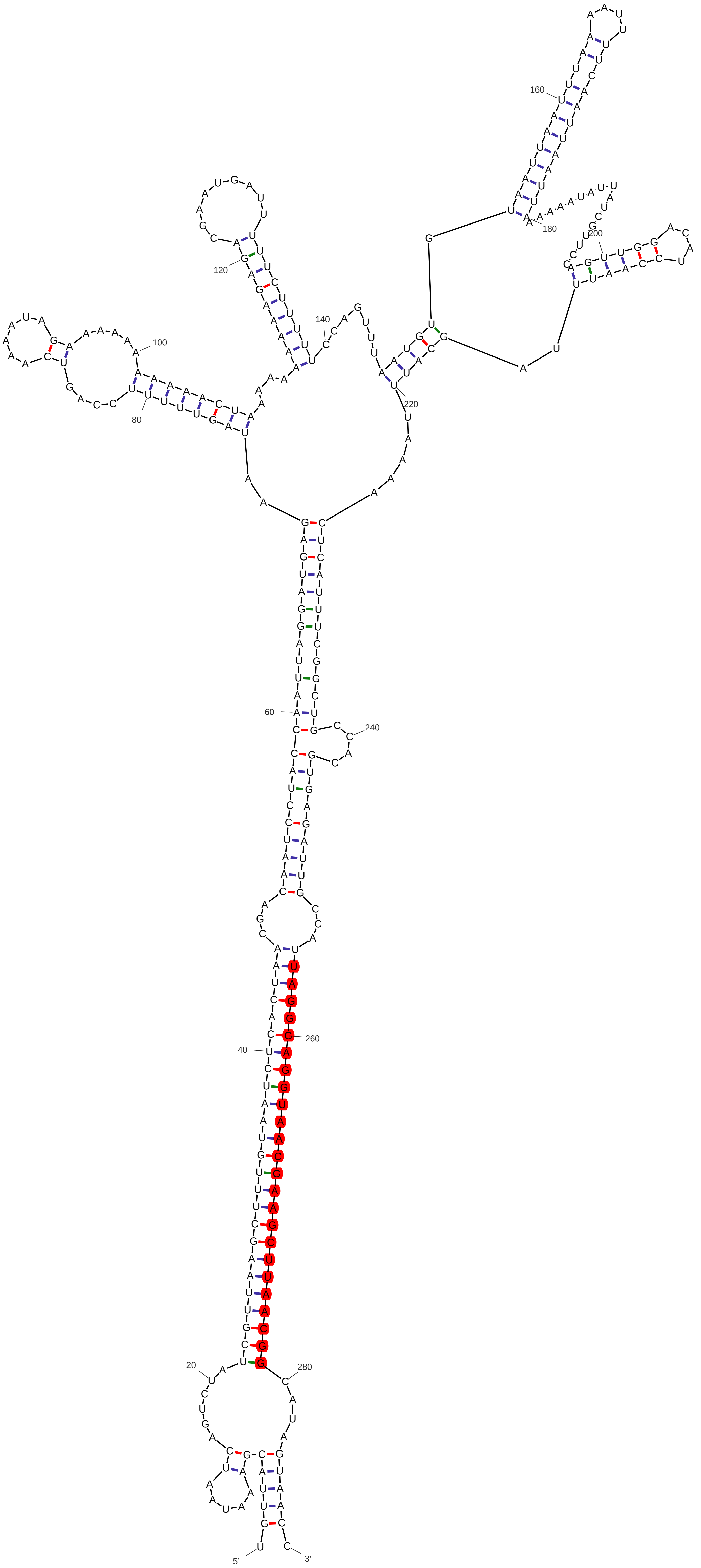

dG = -53.41 [Initially -62.00] novel_mir_2501

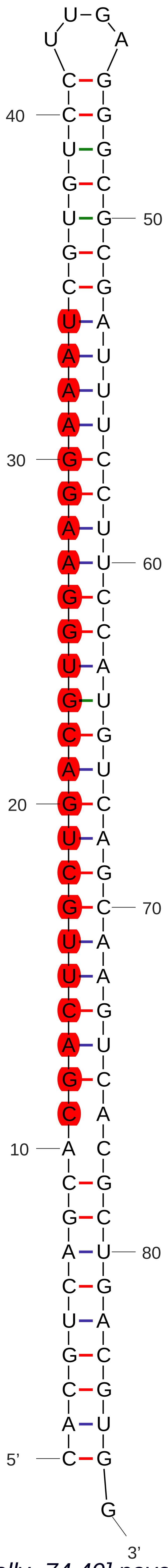

dG = -74.40 [Initially -74.40] novel_mir_1085

Output of sir_graph (©)
mfold_util 4.6

Created Sun Jan 13 09:43:11 2013

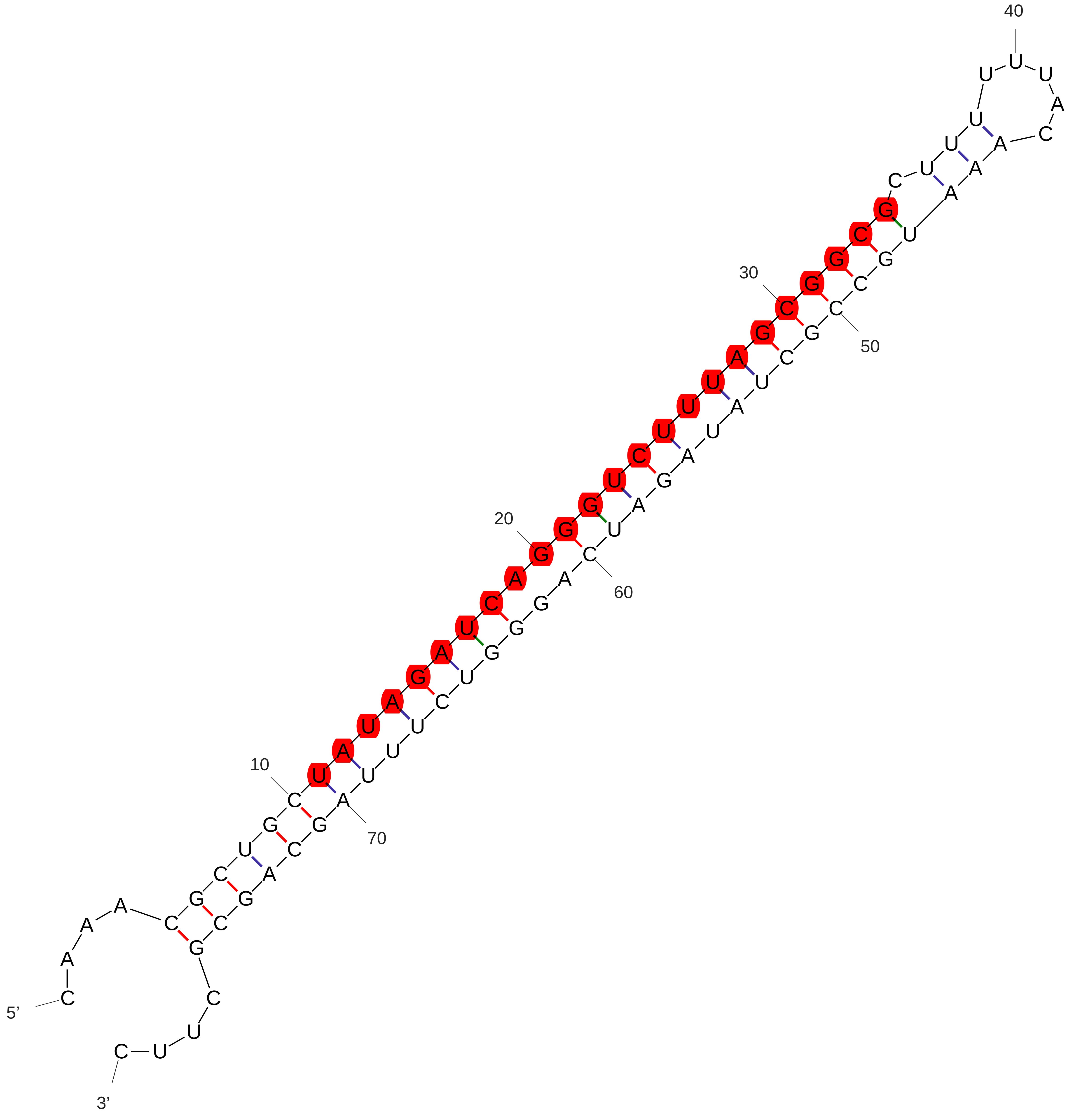

dG = -41.40 [Initially -41.40] novel_mir_2528

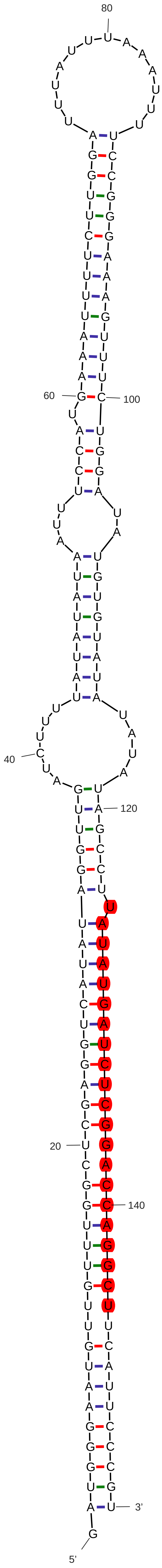

dG = -72.30 [Initially -72.30] novel_mir_225

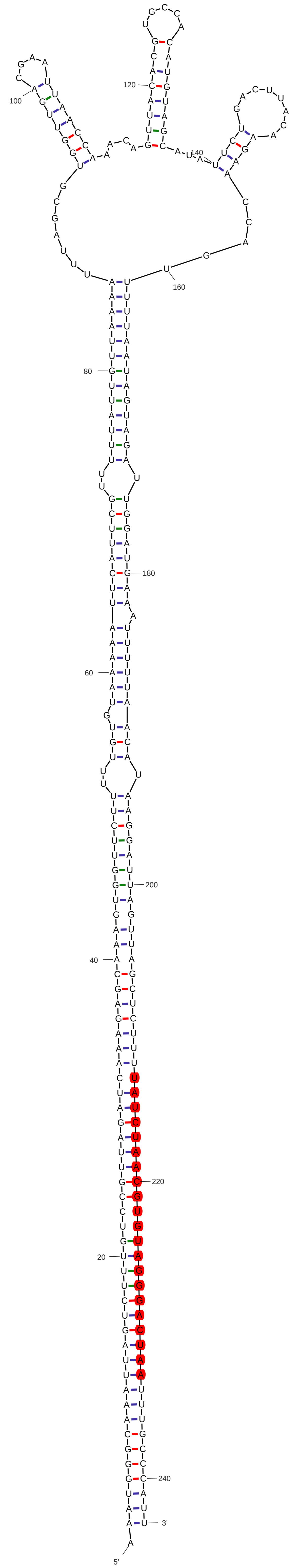

dG = -90.23 [Initially -93.70] novel_mir_2661

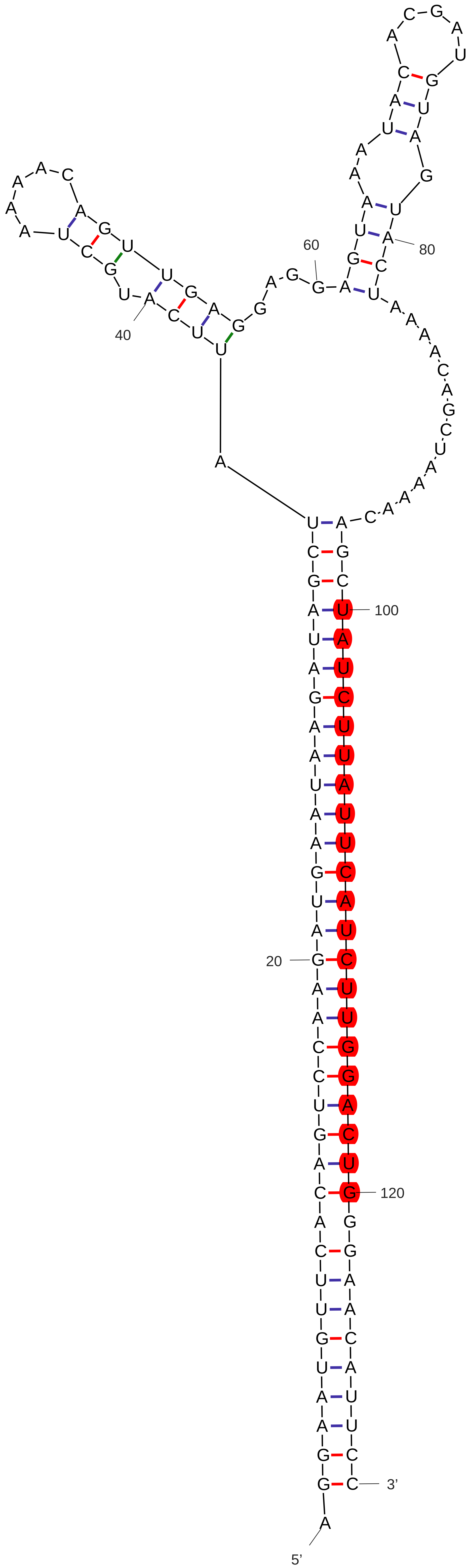

dG = -56.83 [Initially -59.10] novel_mir_4016_1

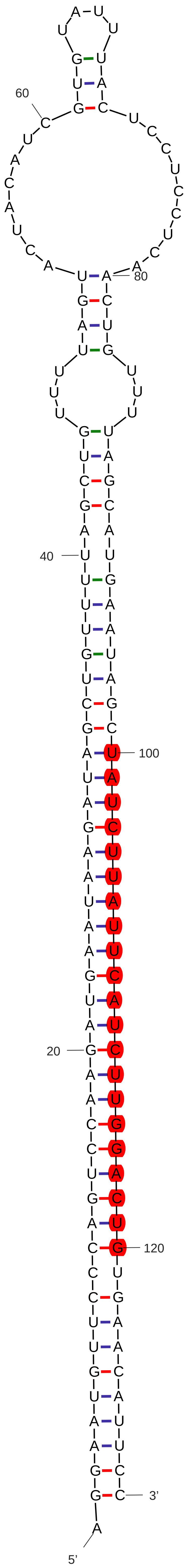

dG = -65.50 [Initially -65.50] novel_mir_4016_2

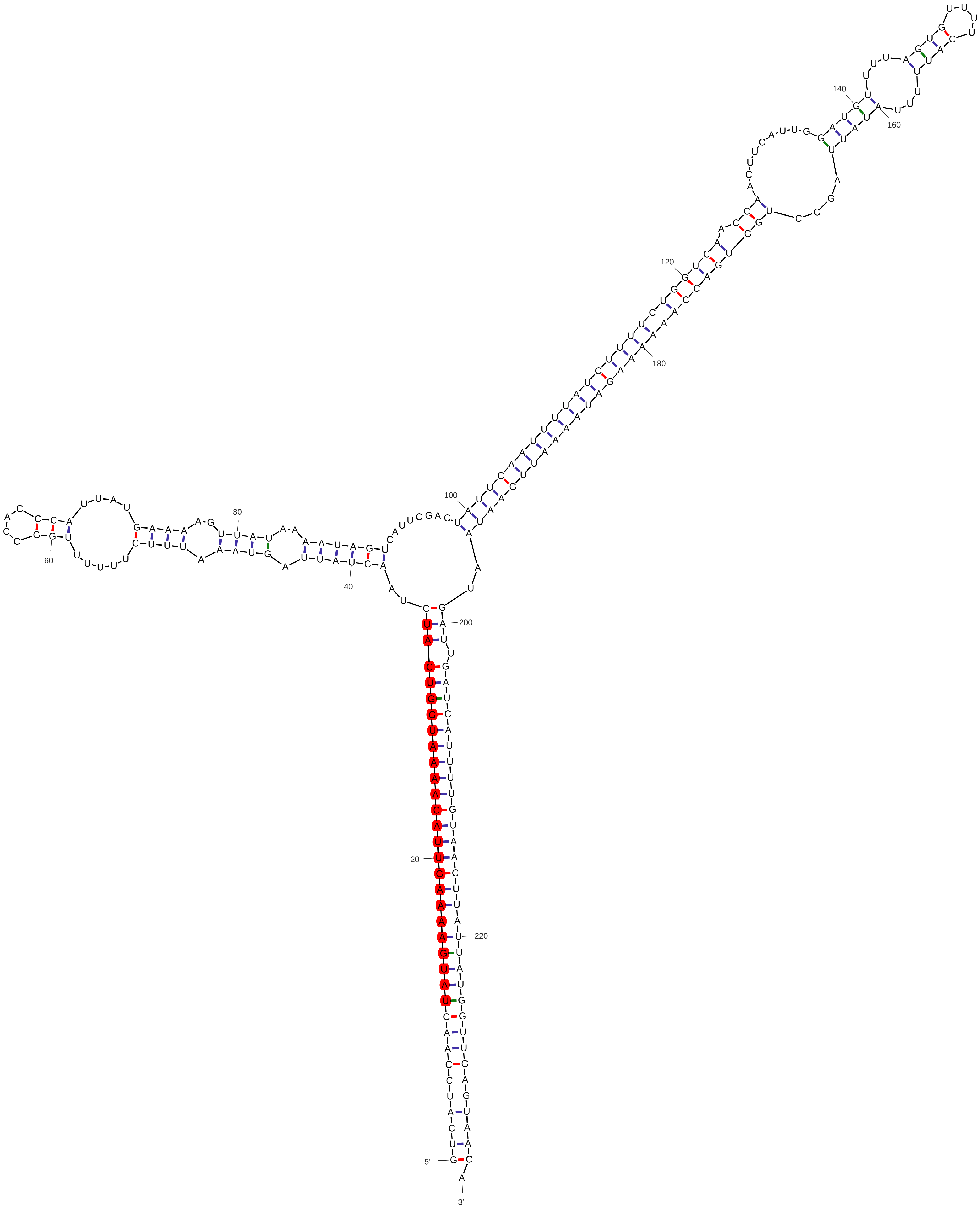

dG = -71.44 [Initially -74.60] novel_mir_3992_1

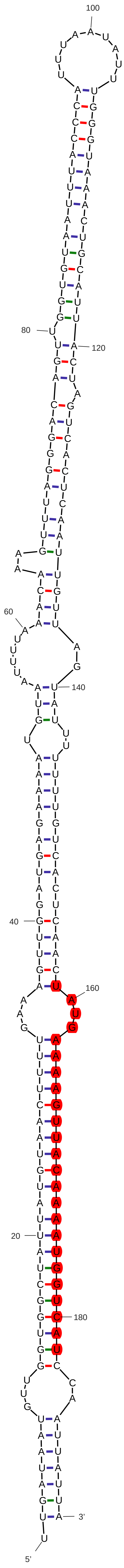

dG = -60.80 [Initially -60.80] novel_mir_3992_2

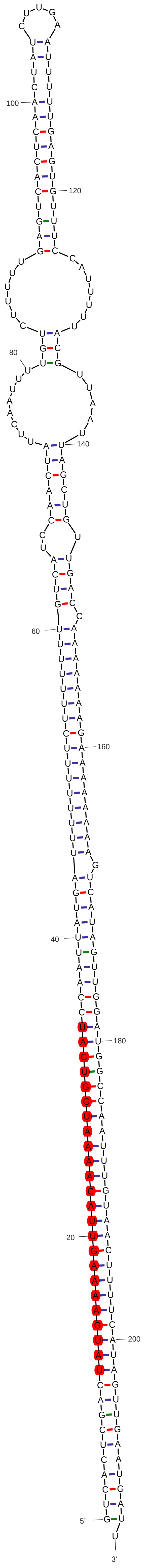

dG = -87.40 [Initially -87.40] novel_mir_3992_3

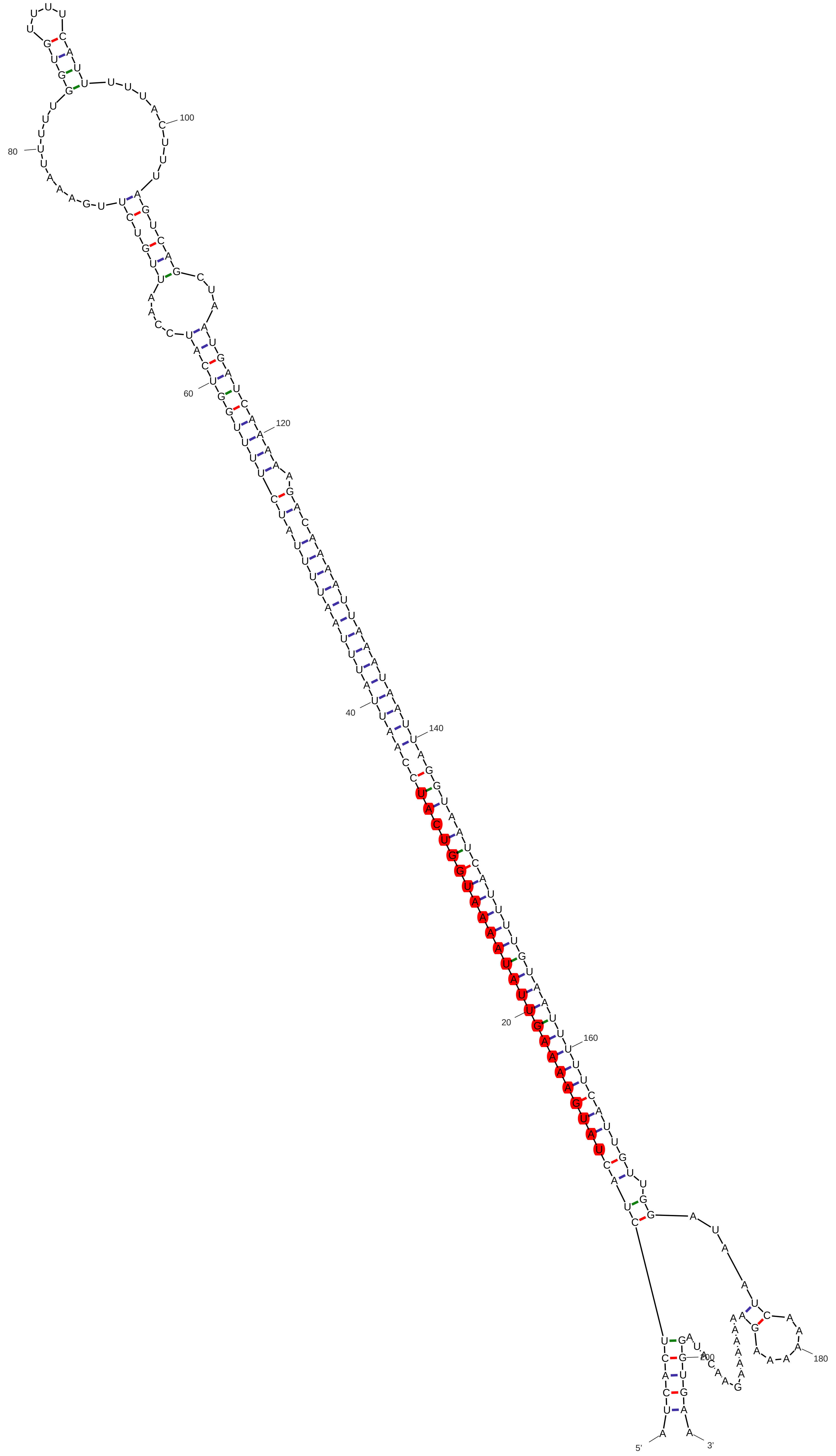

dG = -47.95 [Initially -51.60] novel_mir_936

Output of sir_graph (©)
mfold_util 4.6

Created Sun Jan 13 09:49:31 2013

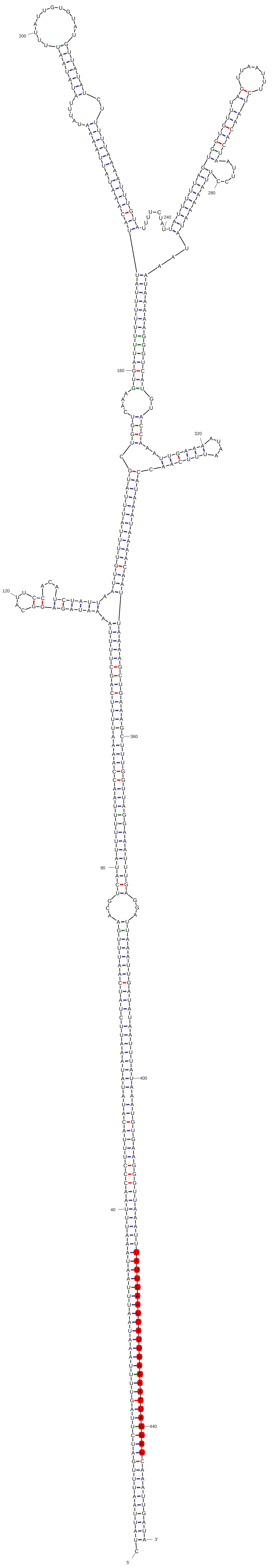

dG = -156.83 [Initially -163.60] novel_mir_1006

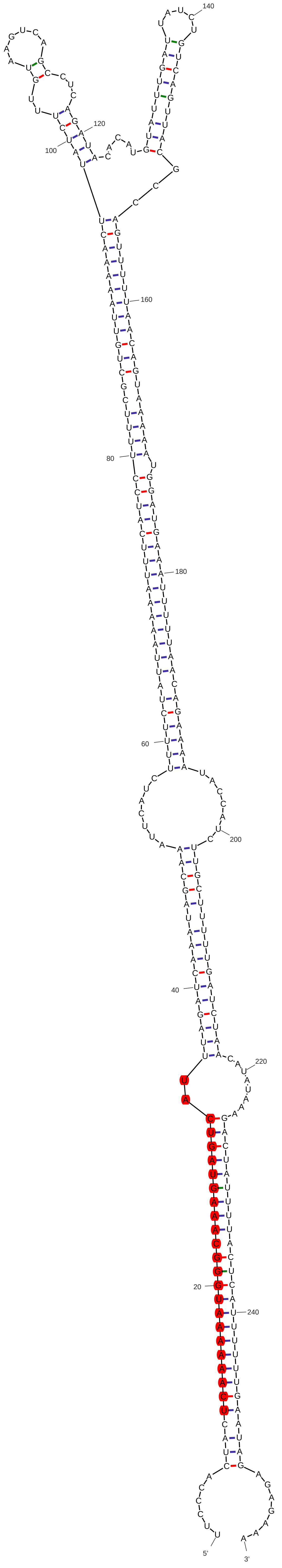

dG = -84.28 [Initially -86.80] novel_mir_1378

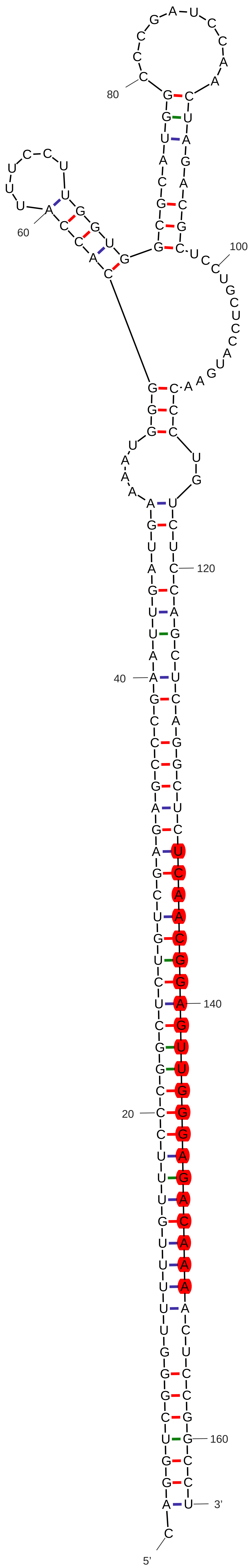

dG = -76.27 [Initially -77.10] novel_mir_30

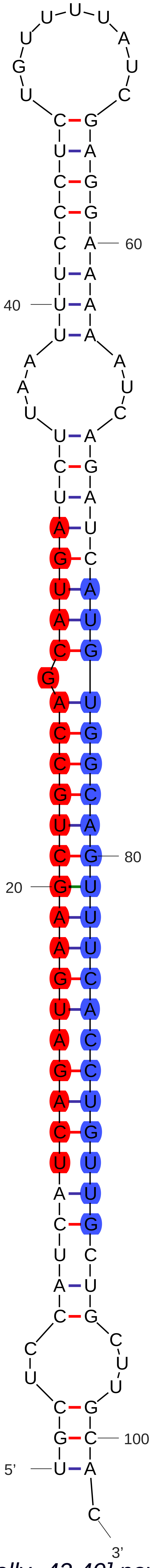

dG = -42.40 [Initially -42.40] novel_mir_95

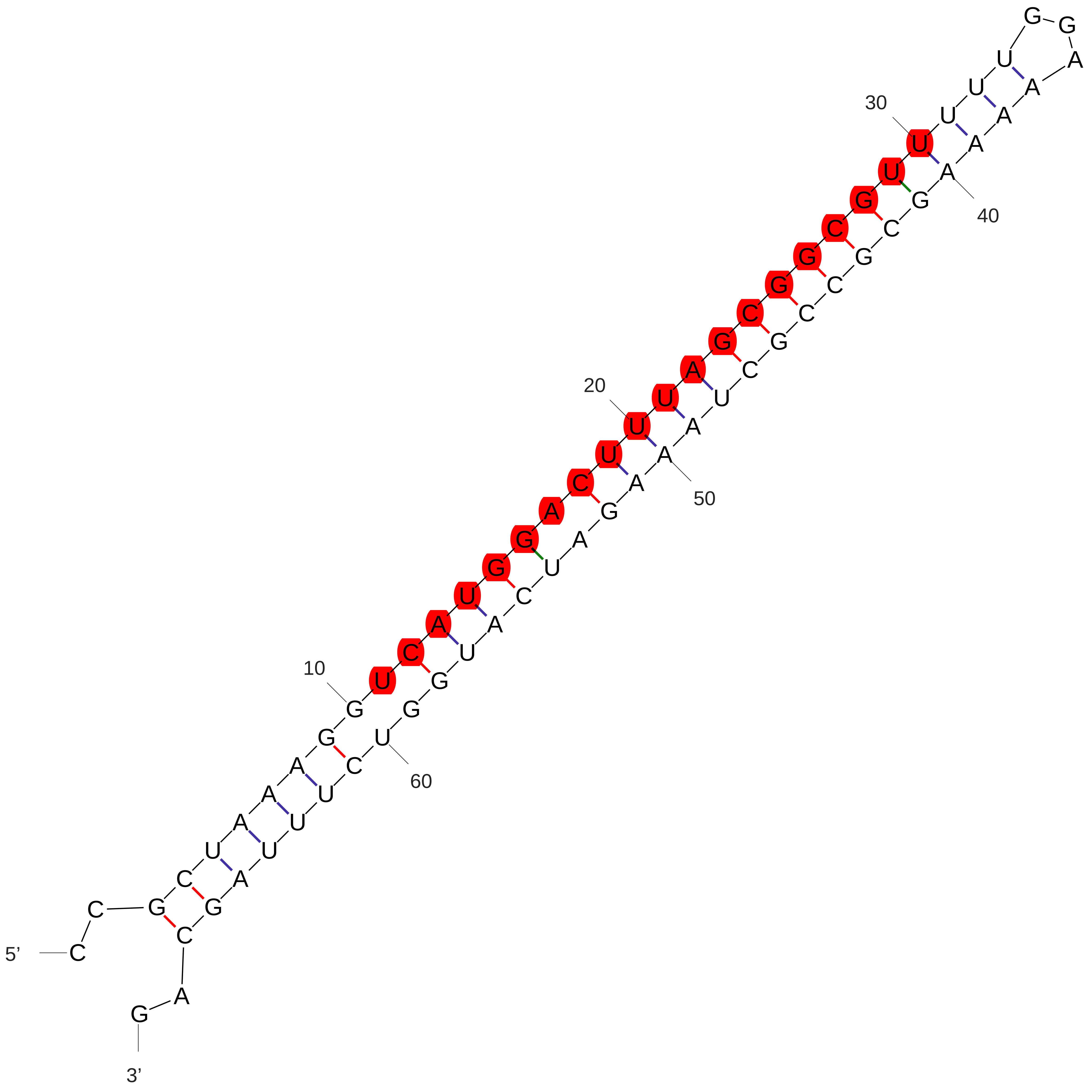

dG = -44.30 [Initially -44.30] novel_mir_1050

Output of sir_graph (©)
mfold_util 4.6

Created Sun Jan 13 09:49:50 2013

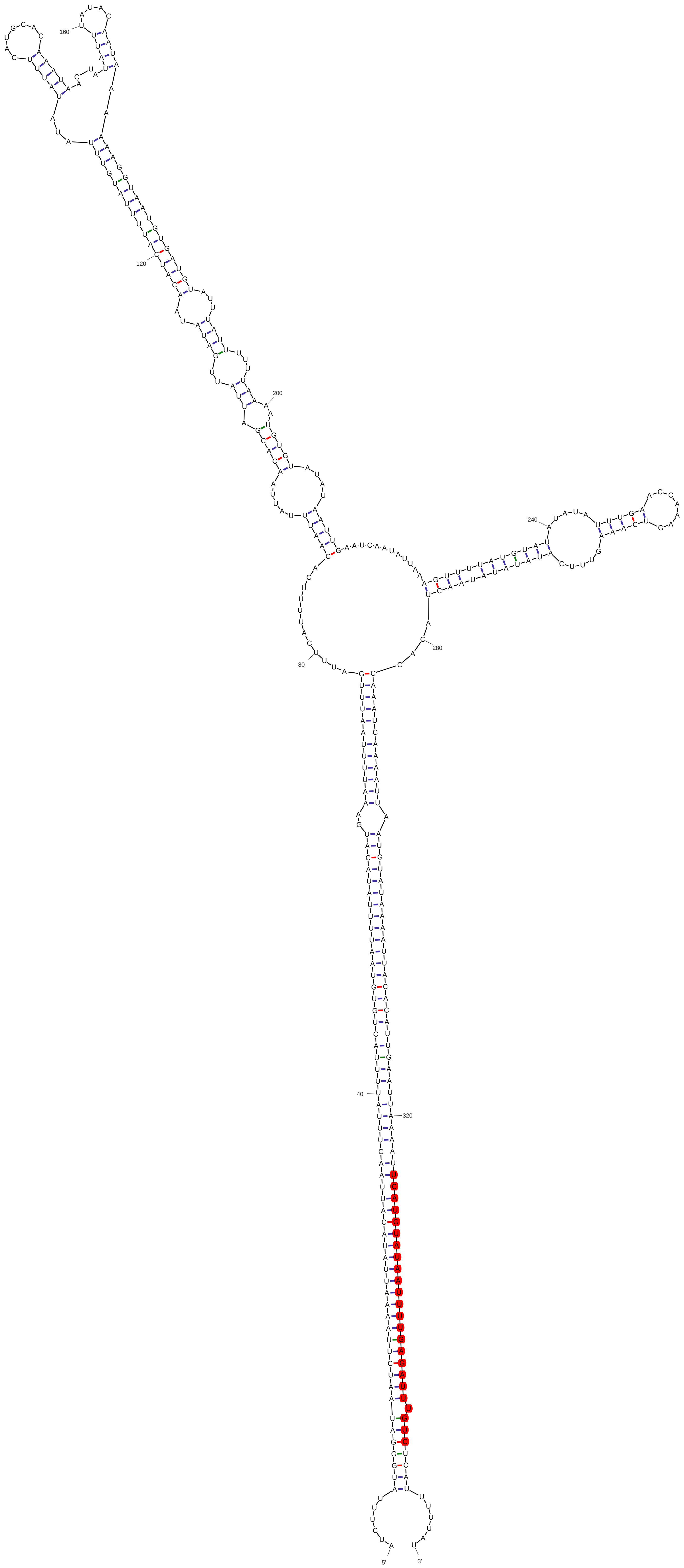

dG = -74.46 [Initially -82.70] novel_mir_5039

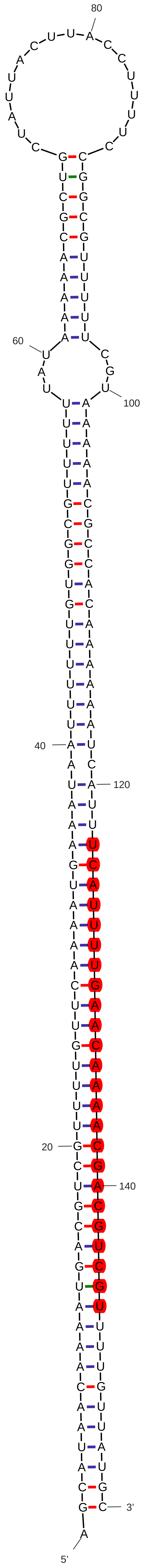

dG = -89.80 [Initially -89.80] novel_mir_960

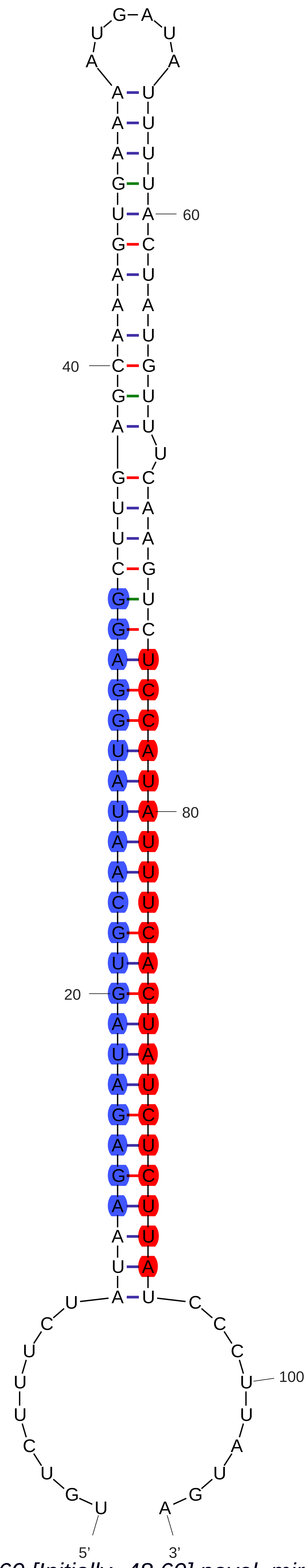

dG = -48.60 [Initially -48.60] novel_mir_50

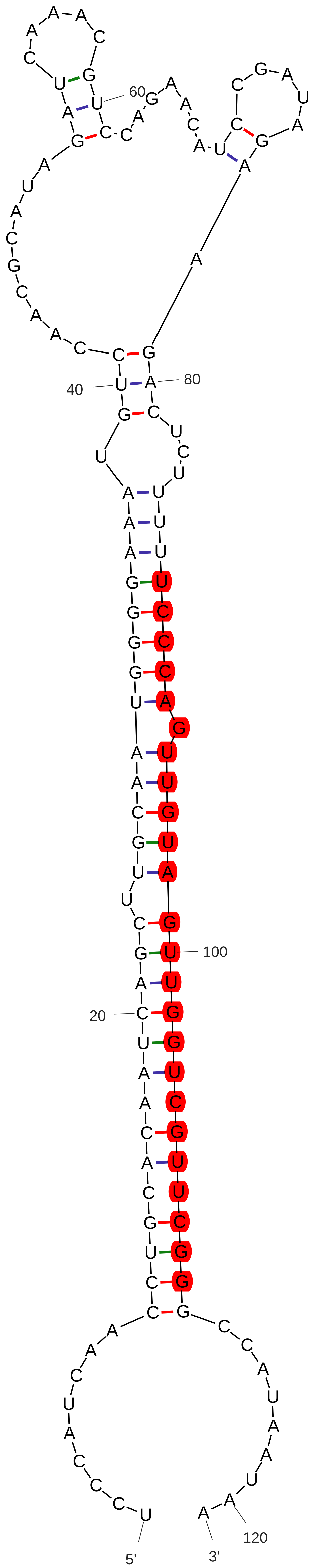

dG = -23.25 [Initially -26.30] novel_mir_2478

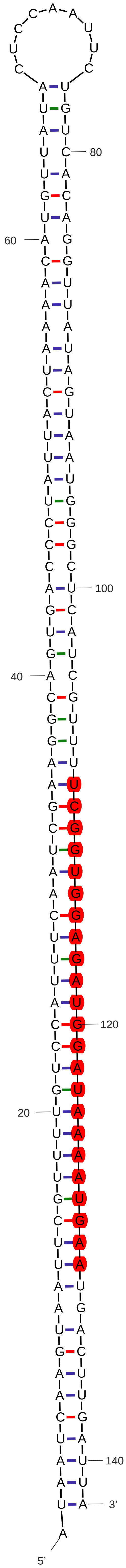

dG = -65.80 [Initially -65.80] novel_mir_4071

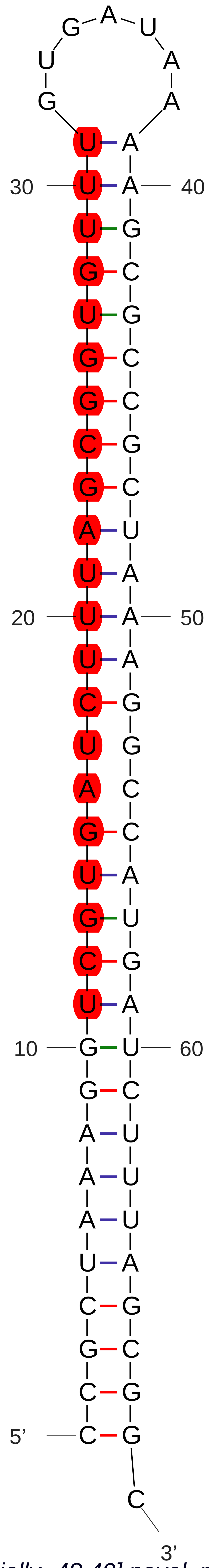

dG = -48.40 [Initially -48.40] novel_mir_1087_1

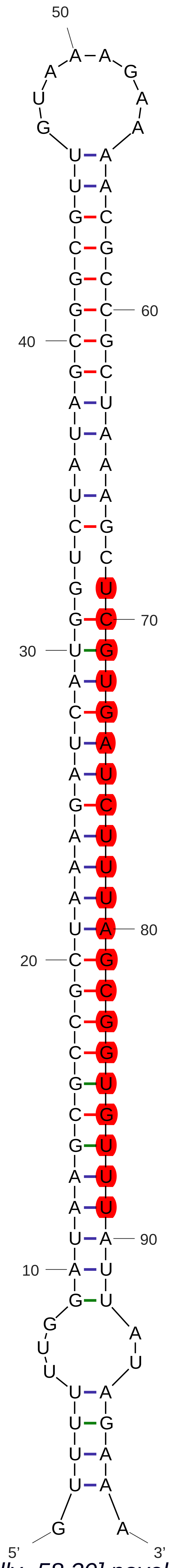

dG = -58.30 [Initially -58.30] novel_mir_1087_2

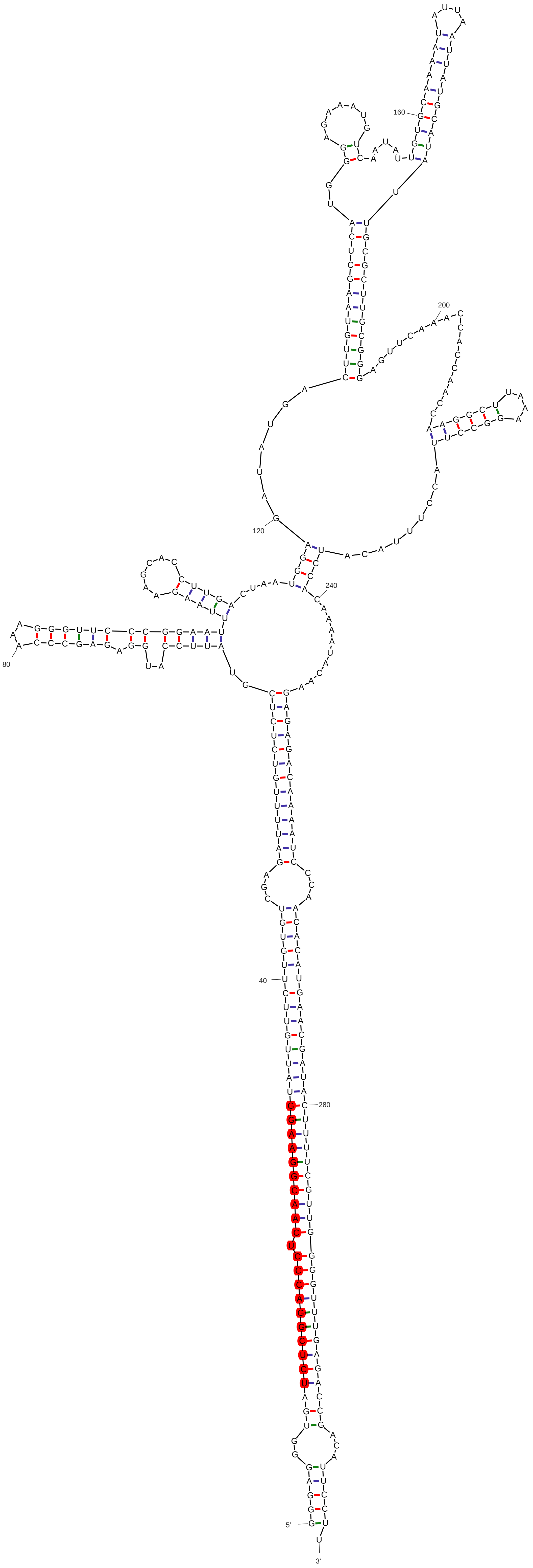

dG = -109.62 [Initially -120.60] novel_mir_2474

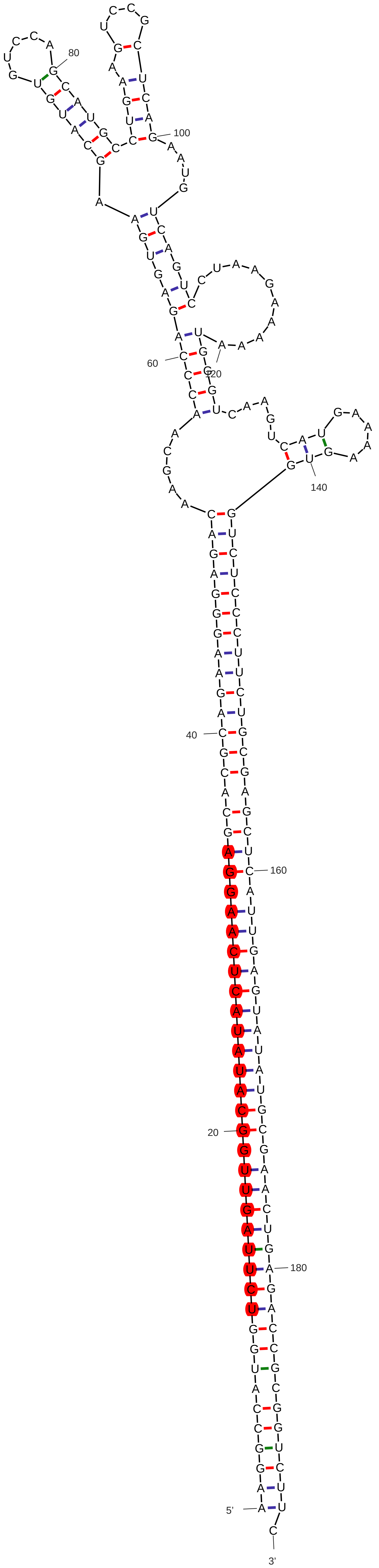

dG = -97.54 [Initially -99.70] novel_mir_5040

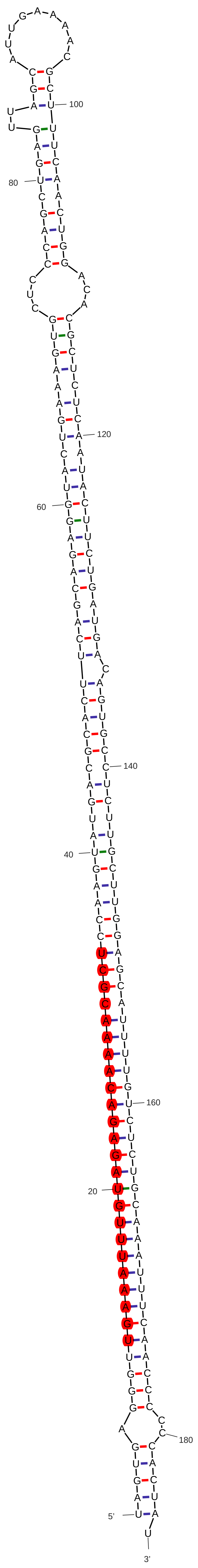

dG = -102.00 [Initially -102.00] novel_mir_2557

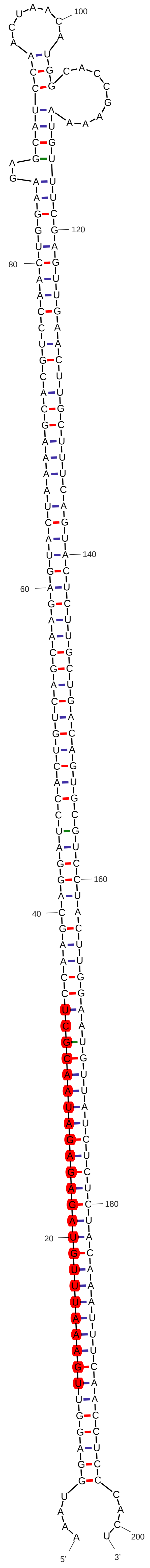

dG = -120.20 [Initially -120.20] novel_mir_2541

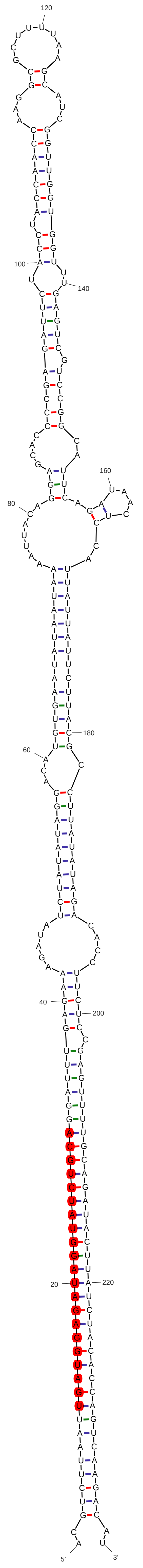

dG = -83.84 [Initially -86.90] novel_mir_90

Output of sir_graph (©)
mfold_util 4.6

Created Sun Jan 13 09:55:52 2013

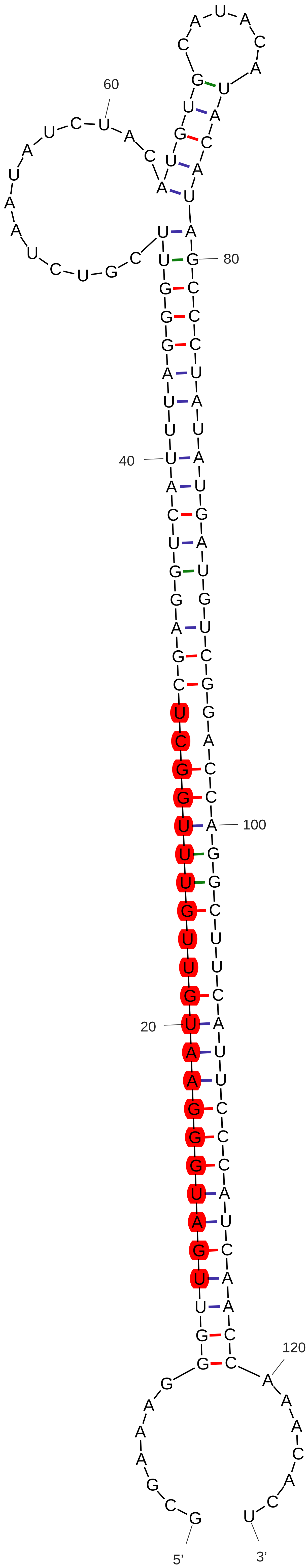

dG = -57.40 [Initially -57.40] novel_mir_4249

Output of sir_graph (©)
mfold_util 4.6

Created Thu Jan 17 20:55:55 2013

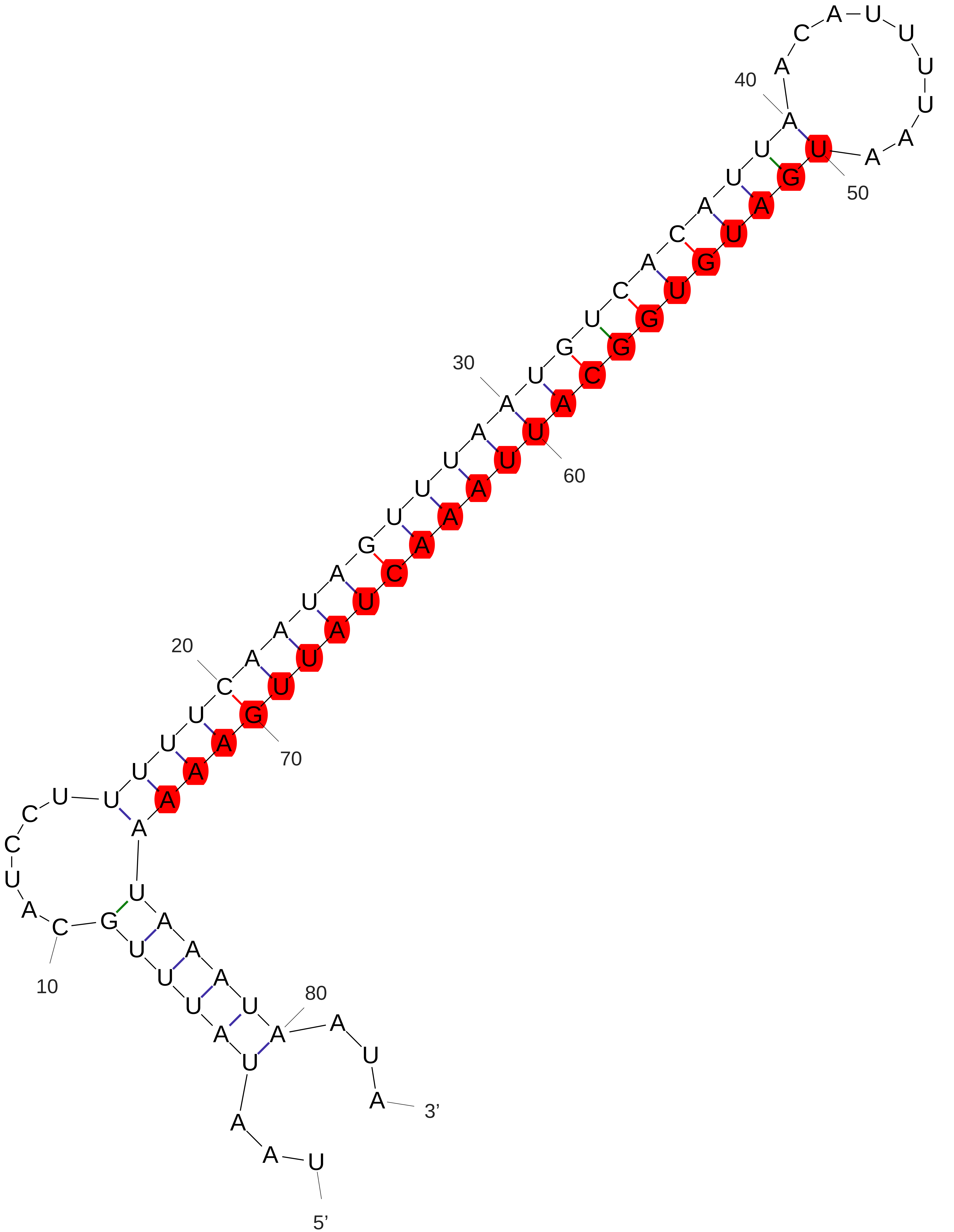

dG = -30.10 [Initially -30.10] novel_mir_860

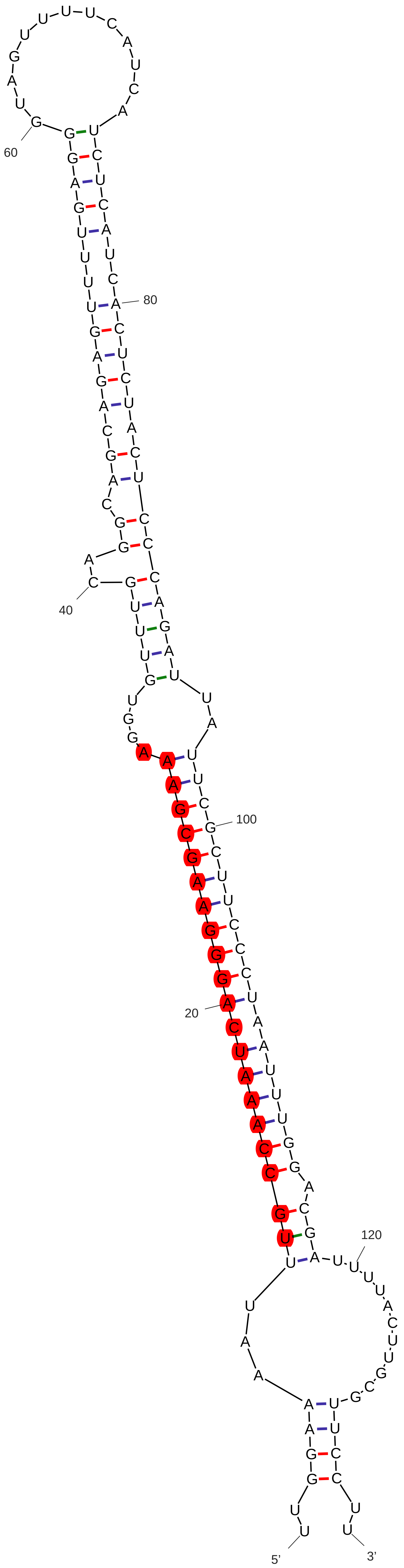

dG = -45.50 [Initially -45.50] novel_mir_2529

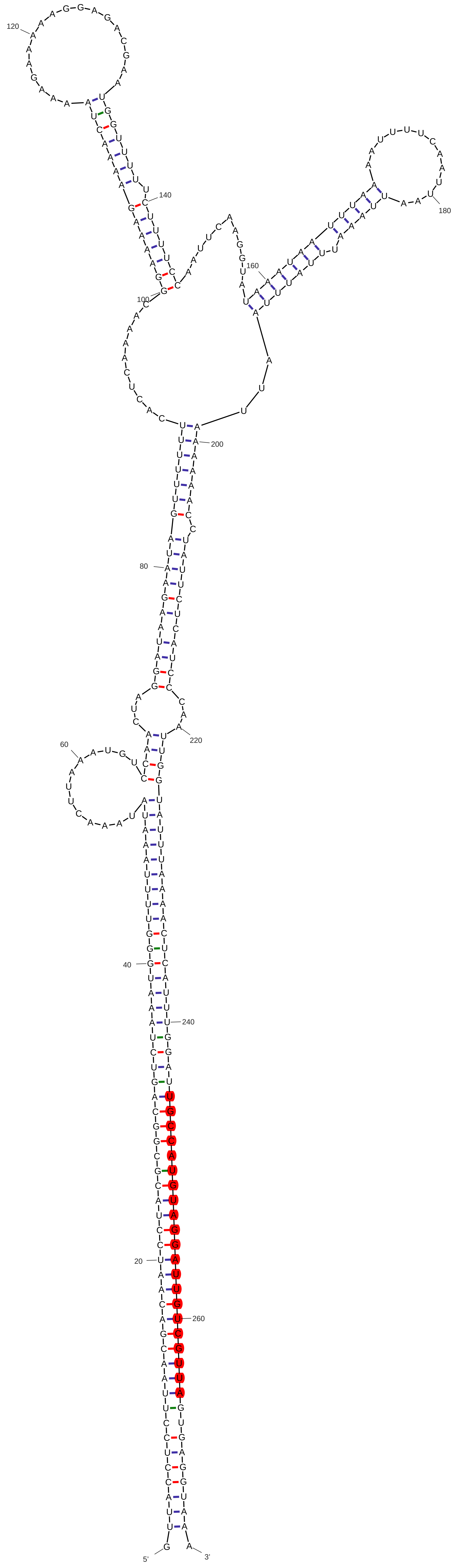

dG = -95.48 [Initially -99.70] novel_mir_4038

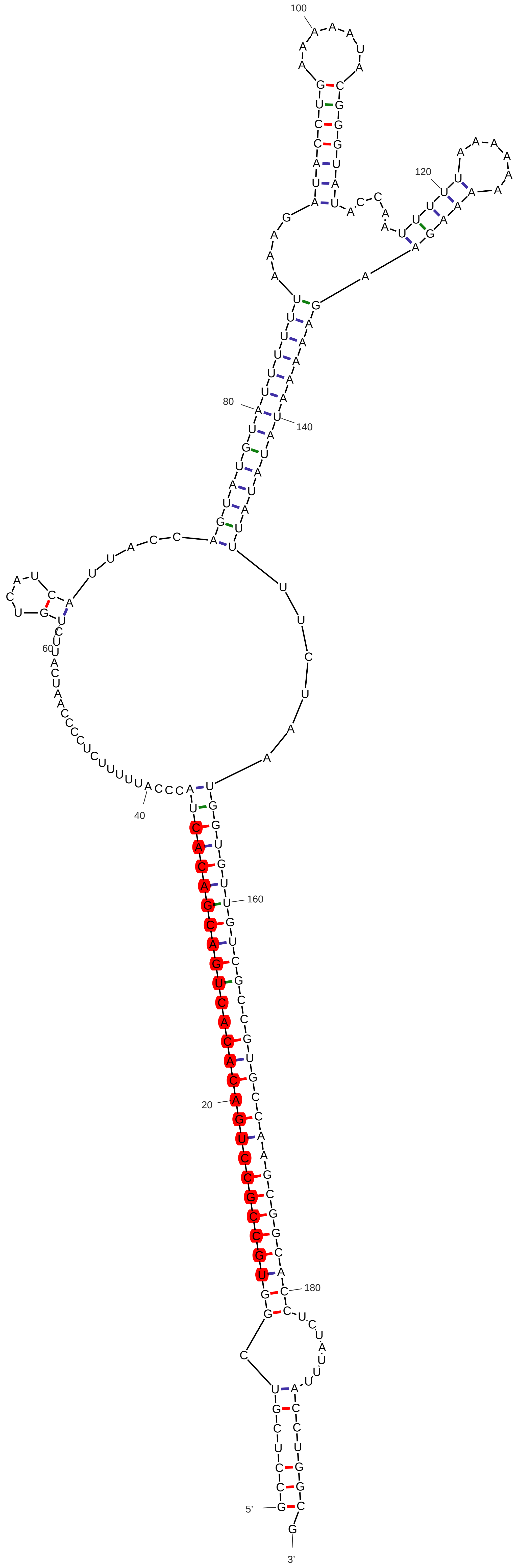

dG = -49.63 [Initially -55.30] novel_mir_4414

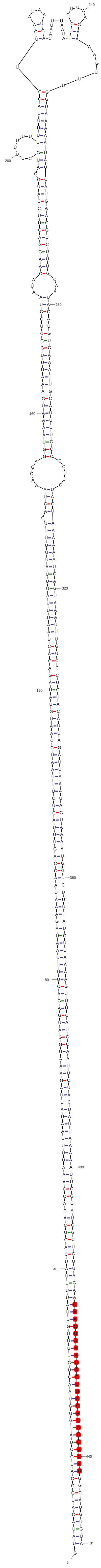

dG = -183.05 [Initially -186.00] novel_mir_5023

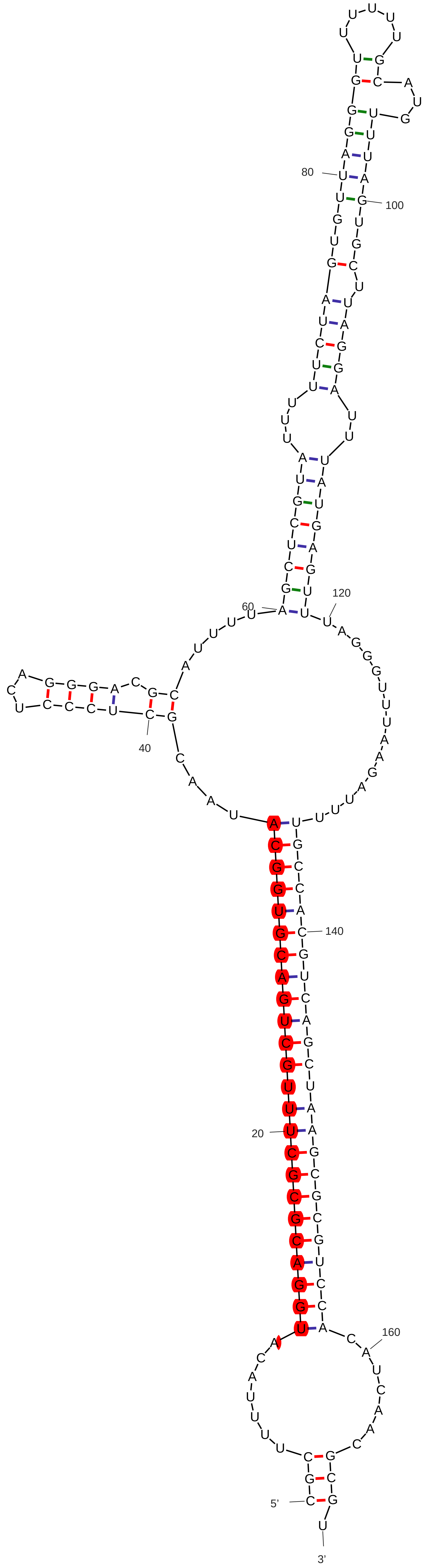

dG = -66.98 [Initially -70.90] novel_mir_2634

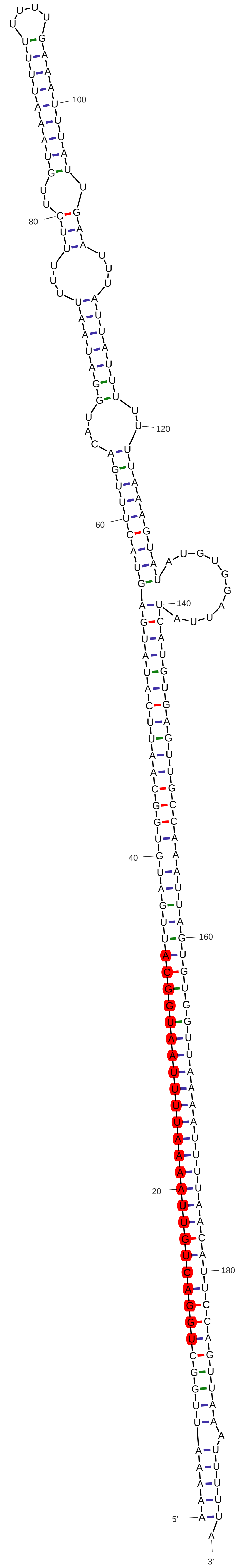

dG = -77.40 [Initially -77.40] novel_mir_4977

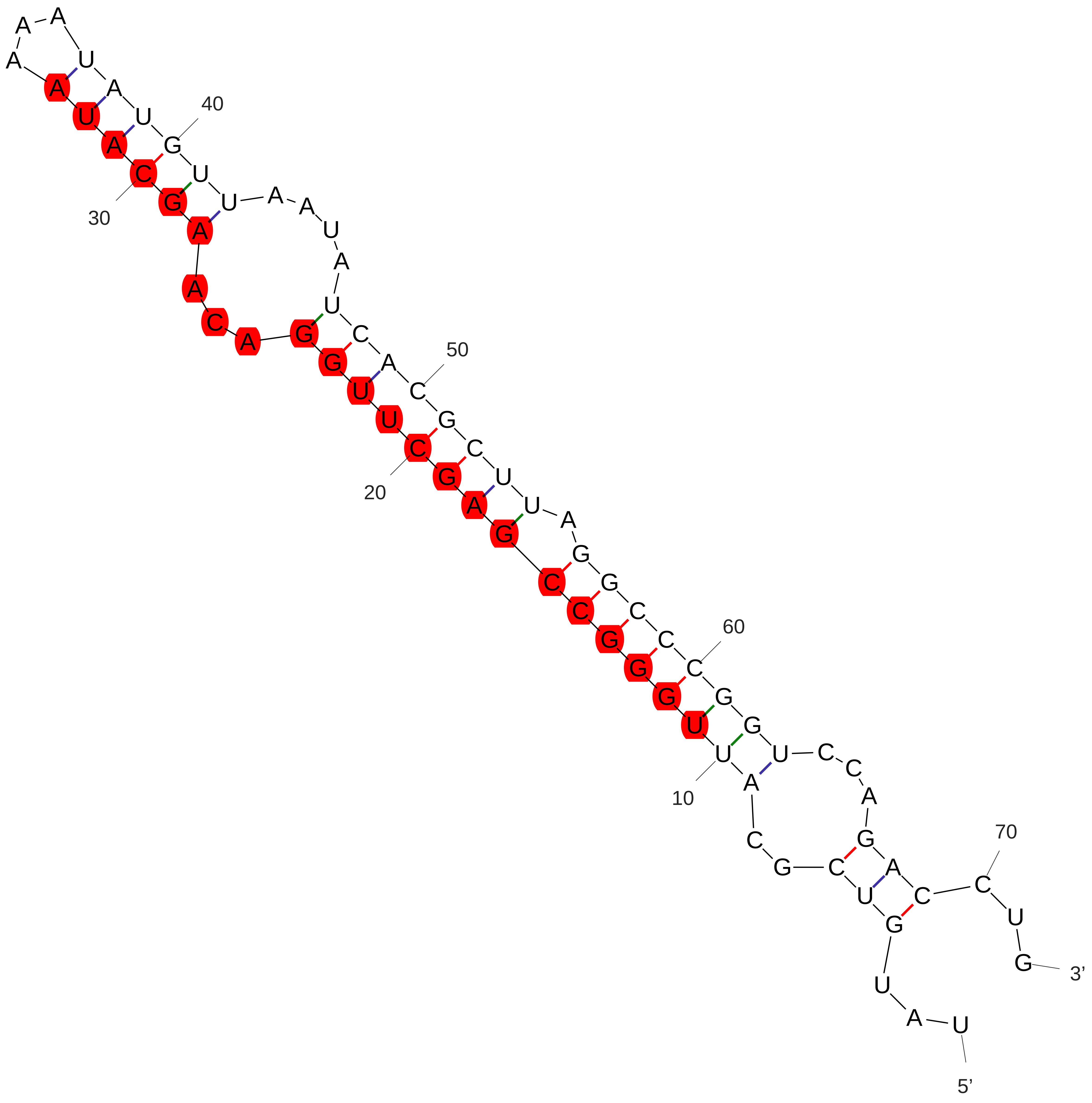

dG = -24.30 [Initially -24.30] novel_mir_2214

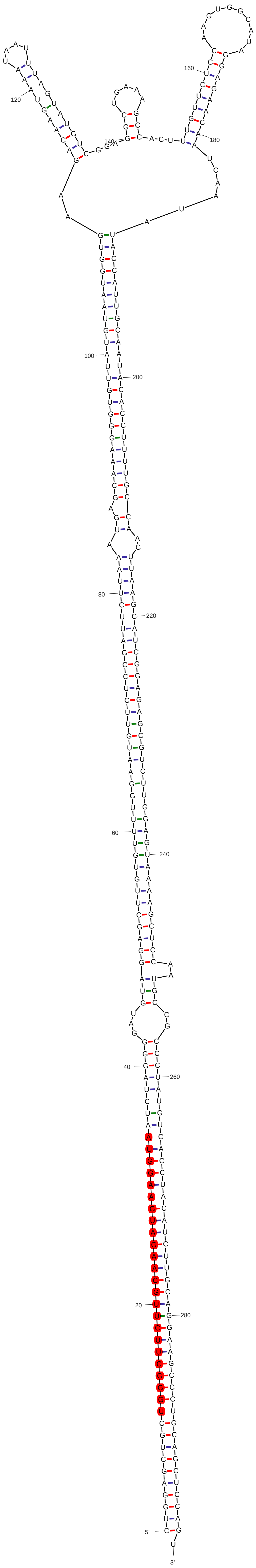

dG = -152.97 [Initially -156.20] novel_mir_5046

Output of sir_graph (©)
mfold_util 4.6

Created Sun Jan 13 10:00:20 2013

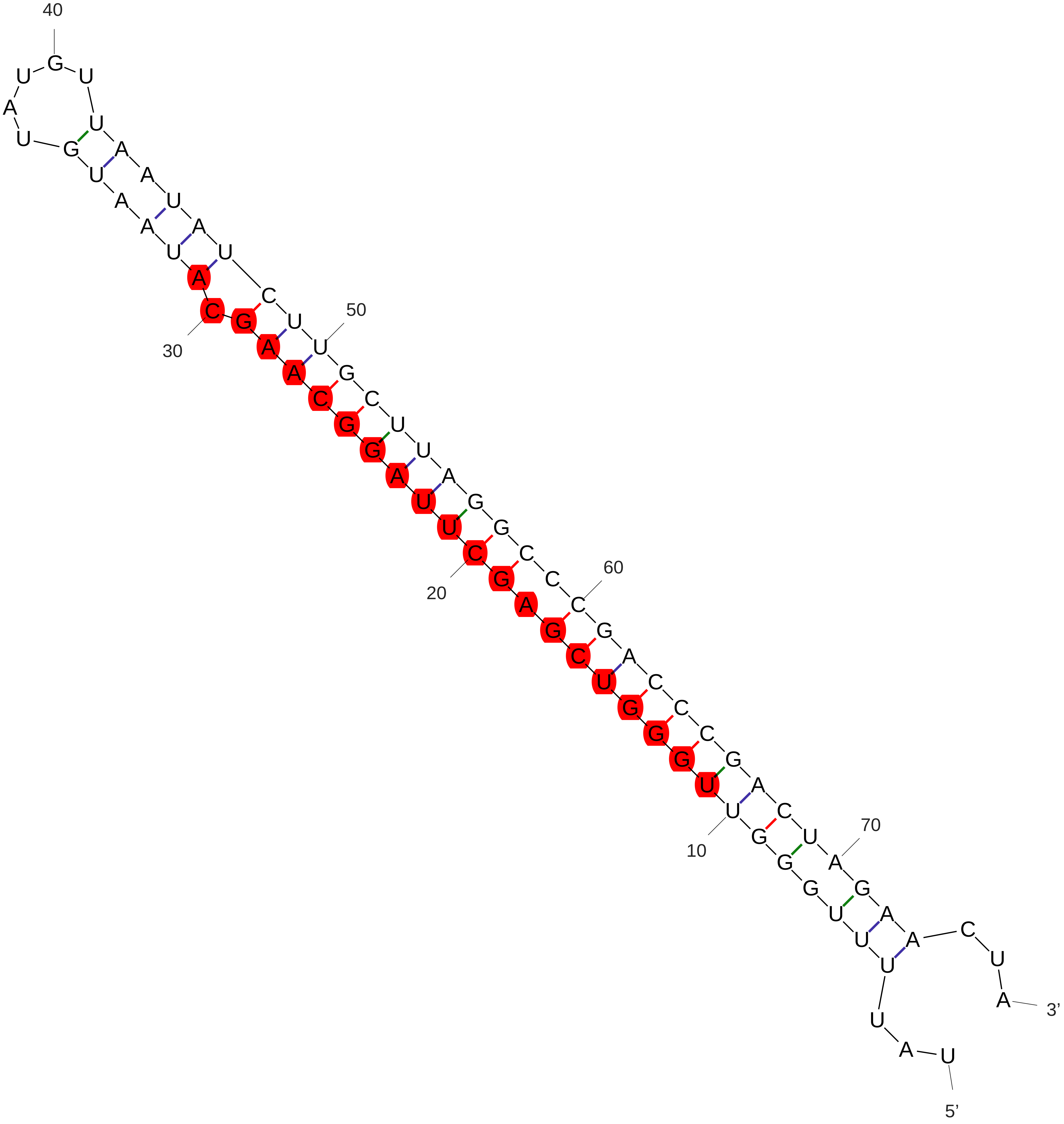

dG = -35.40 [Initially -35.40] novel_mir_892

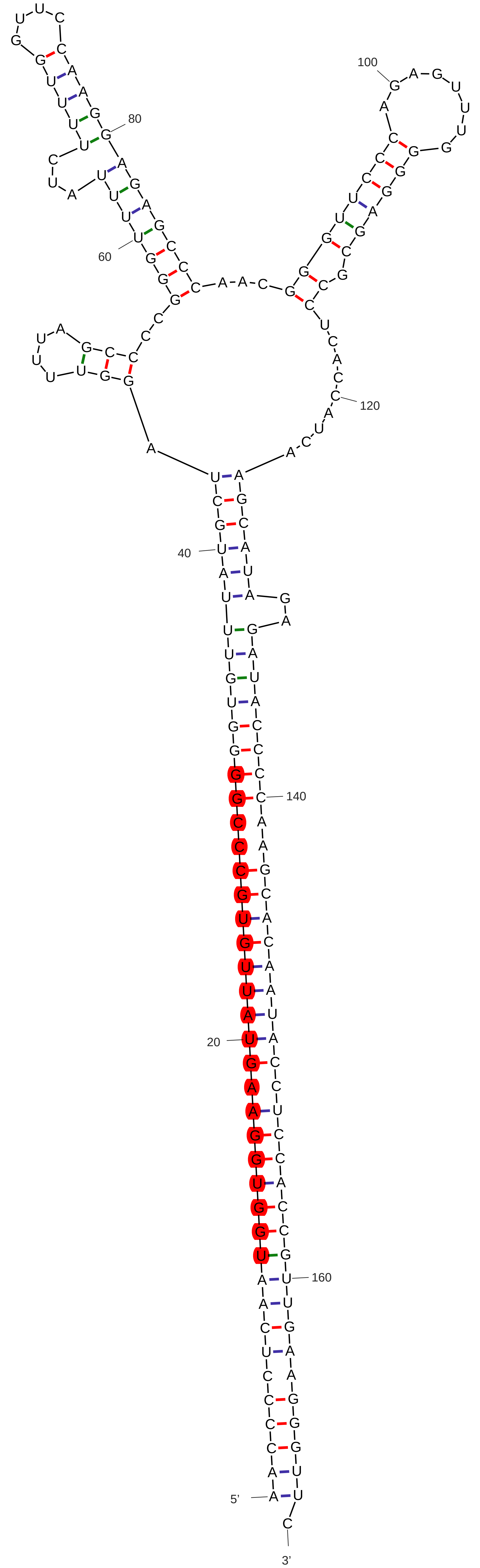

dG = -82.49 [Initially -84.50] novel_mir_4329

Output of sir_graph (©)
mfold_util 4.6

Created Sun Jan 13 10:00:36 2013

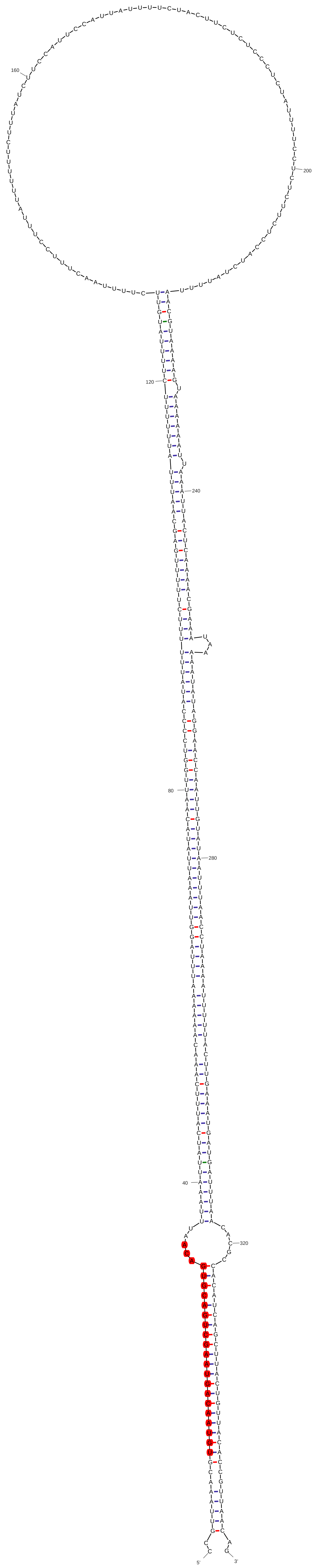

dG = -114.83 [Initially -114.83] novel_mir_2498

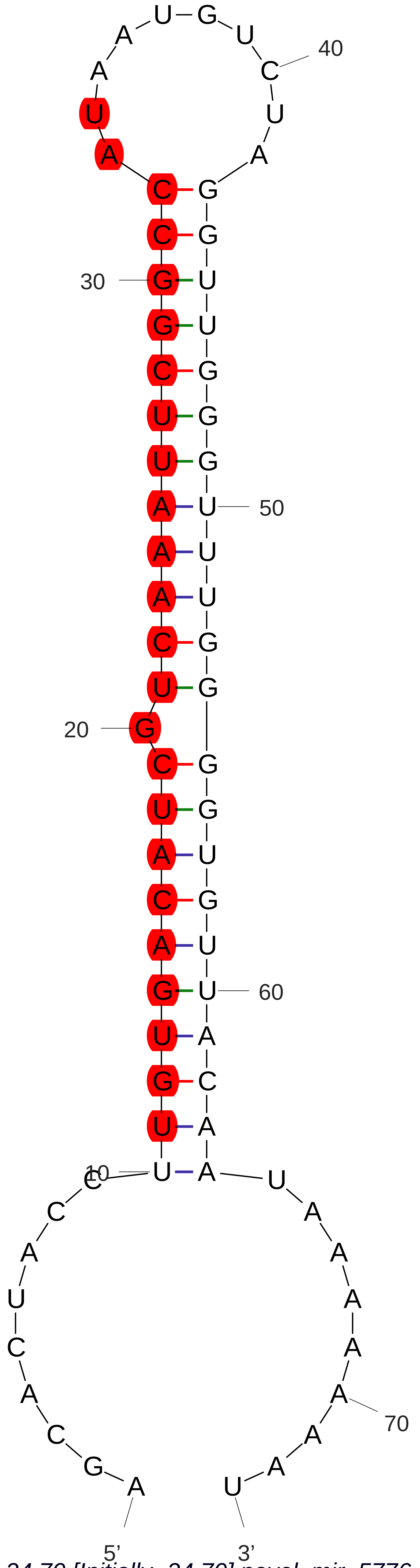

dG = -24.70 [Initially -24.70] novel_mir_5776

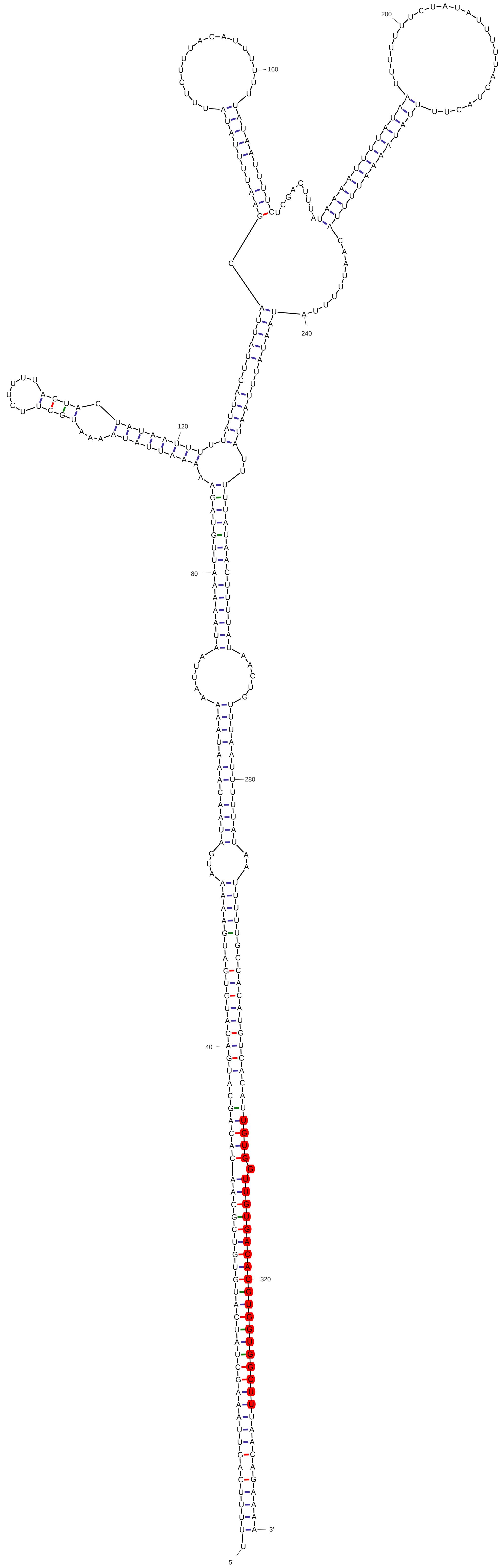

dG = -81.53 [Initially -86.20] novel_mir_2561

Output of sir_graph (©)
mfold_util 4.6

Created Sun Jan 13 07:18:07 2013

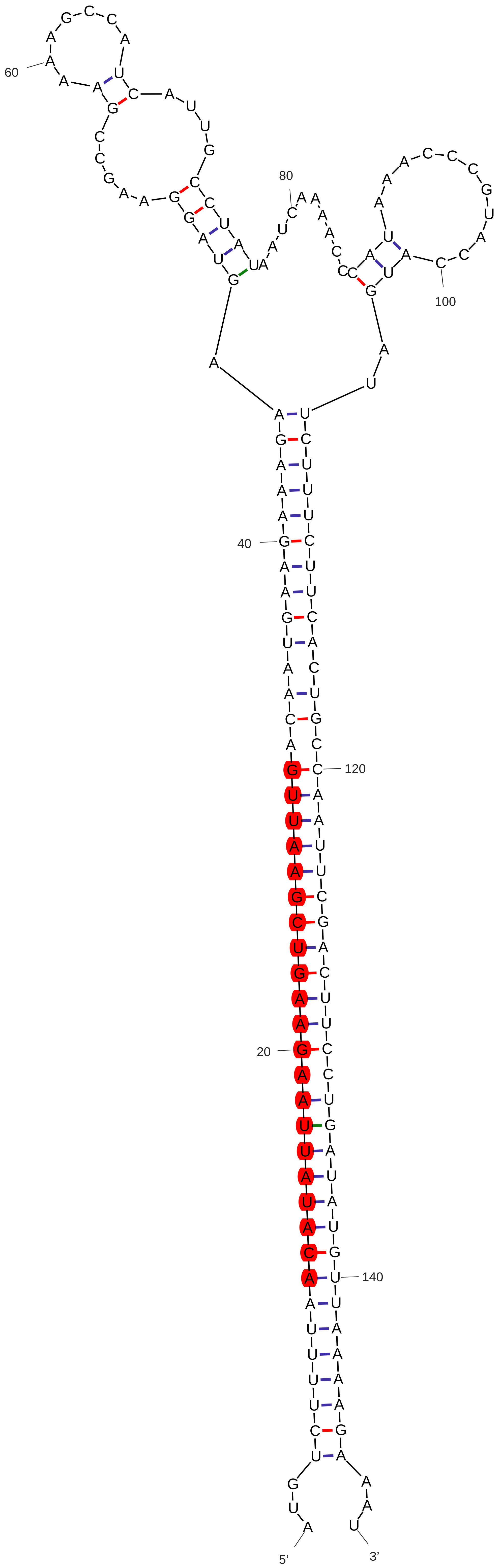

dG = -50.75 [Initially -52.40] novel_mir_2554_1

Output of sir_graph (©)
mfold_util 4.6

Created Sun Jan 13 07:18:07 2013

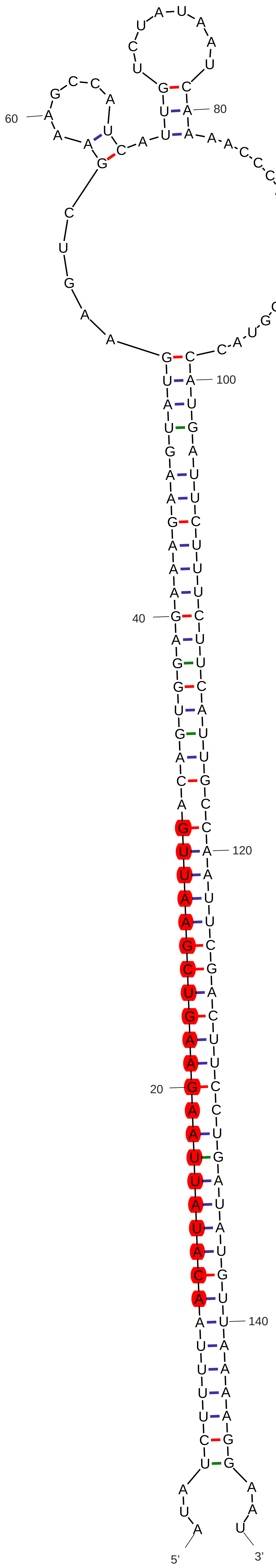

dG = -51.52 [Initially -56.00] novel_mir_2554_2

Output of sir_graph (©)
mfold_util 4.6

Created Sun Jan 13 10:00:55 2013

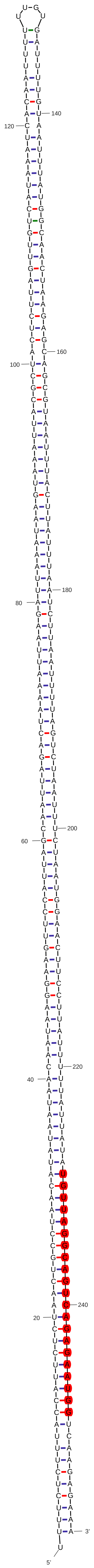

dG = -166.50 [Initially -166.50] novel_mir_928

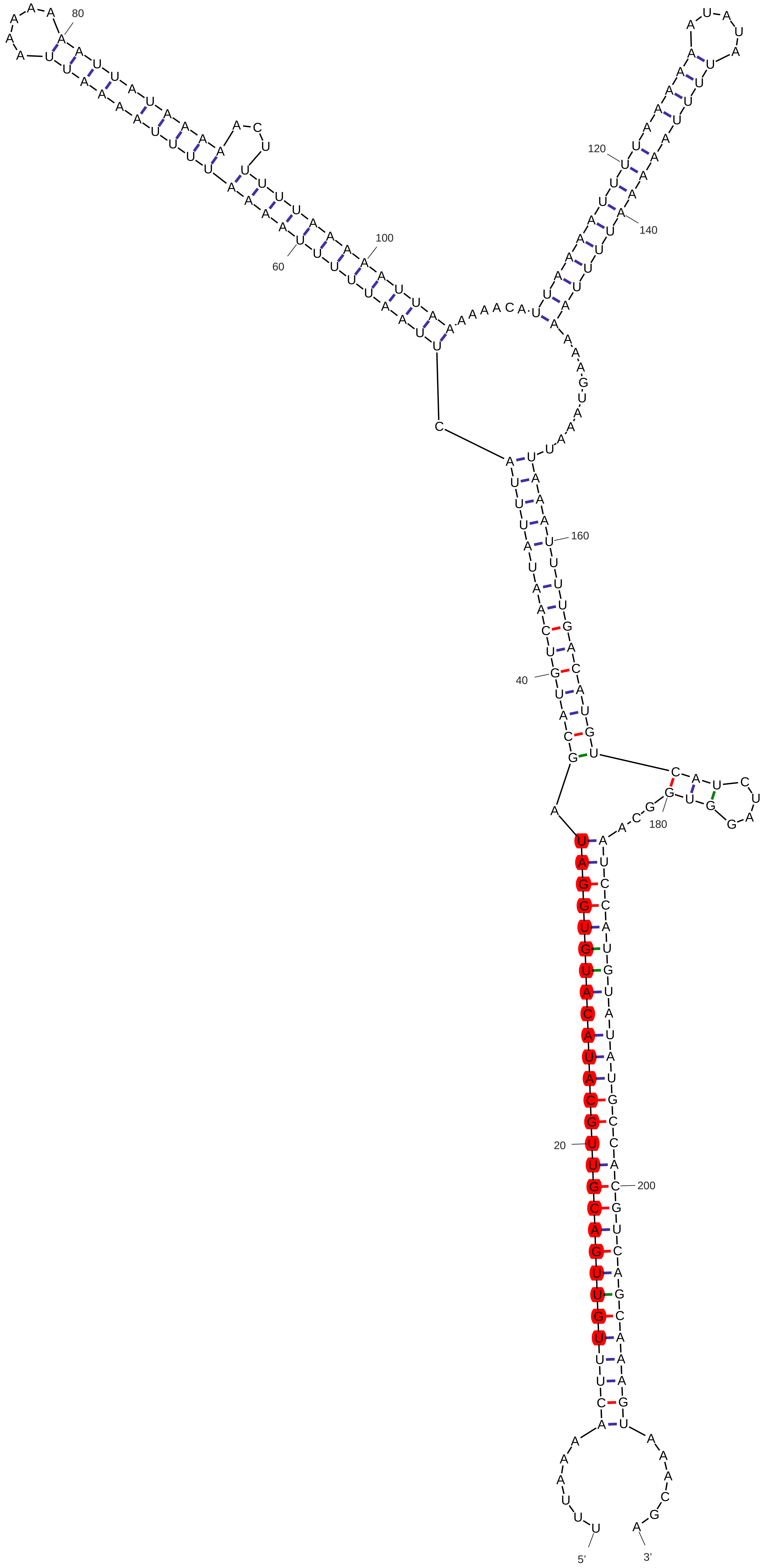

dG = -57.92 [Initially -62.60] novel_mir_2538

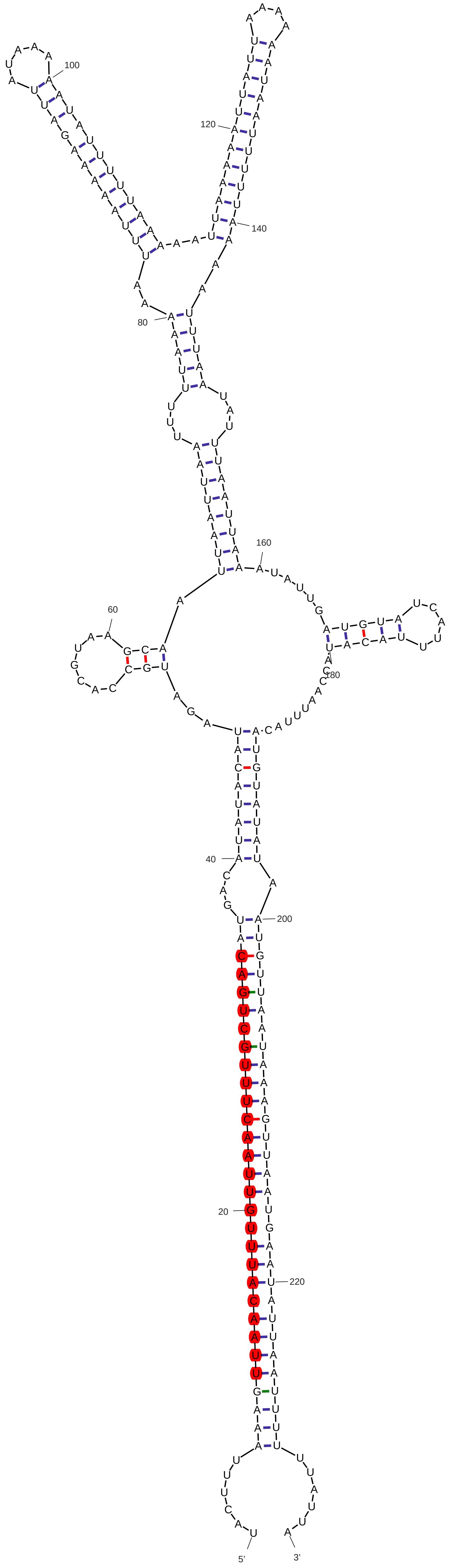

dG = -40.18 [Initially -46.00] novel_mir_4086_1

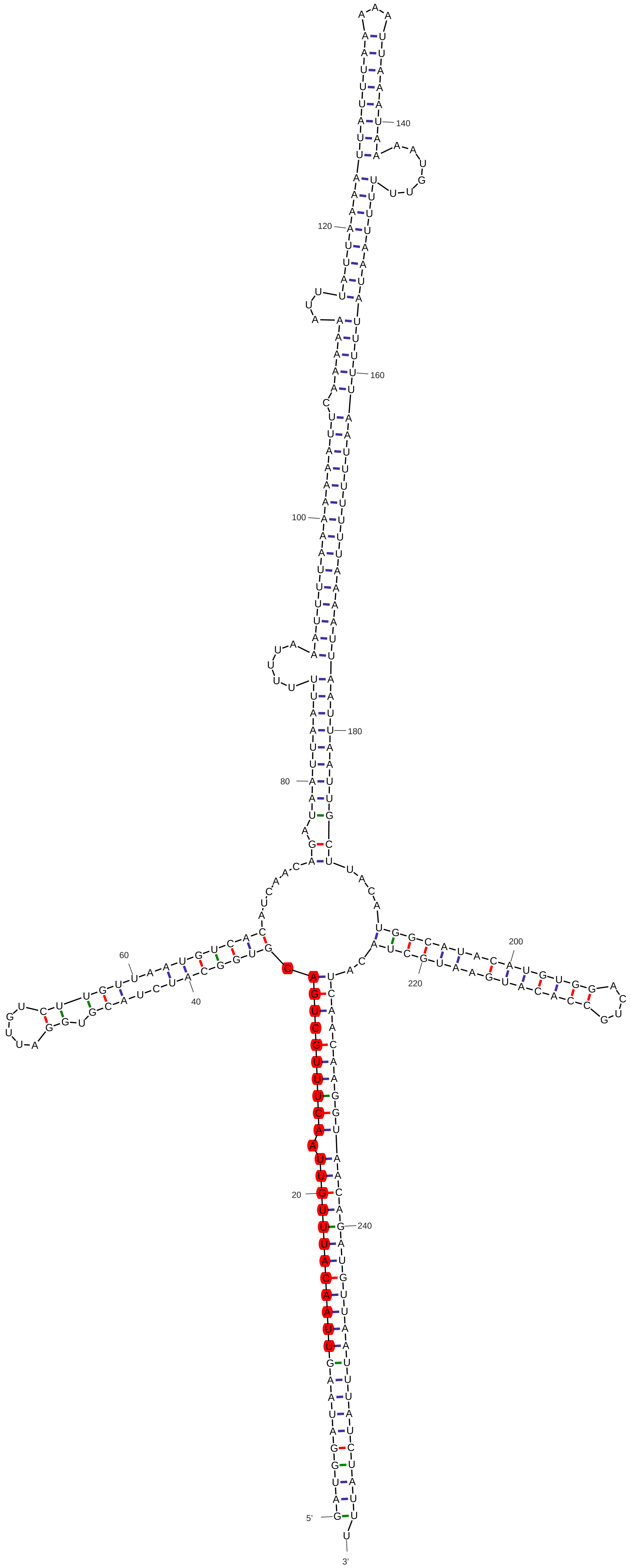

dG = -75.65 [Initially -77.70] novel_mir_4086_2

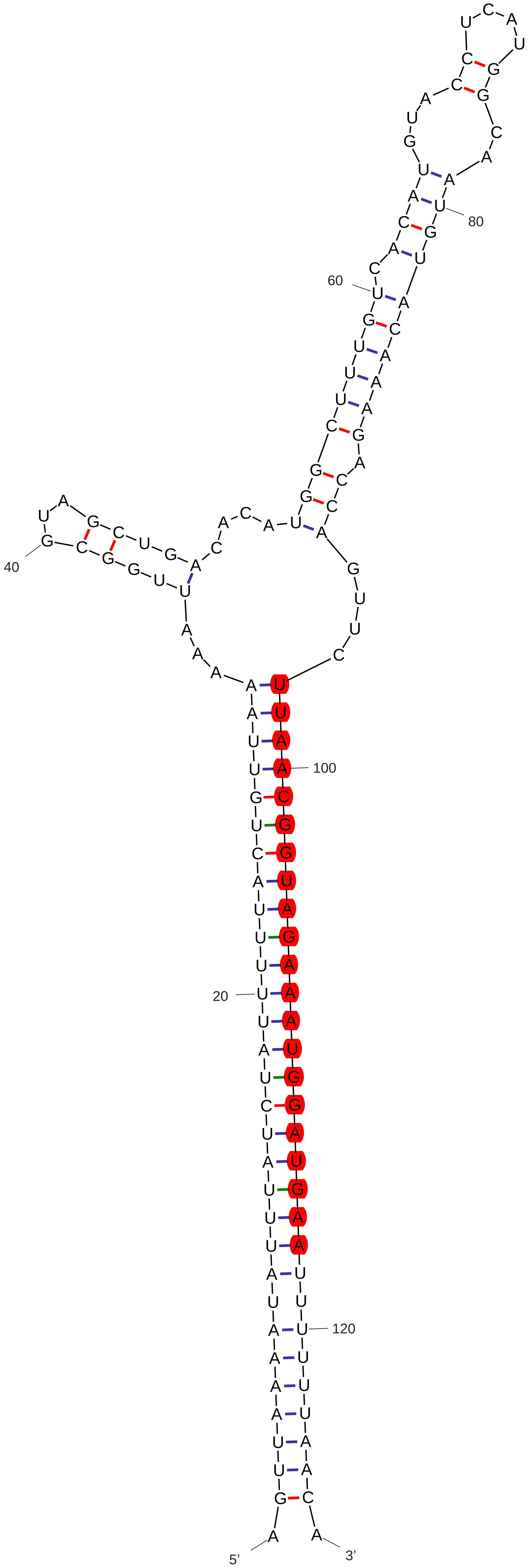

dG = -43.93 [Initially -47.30] novel_mir_2765

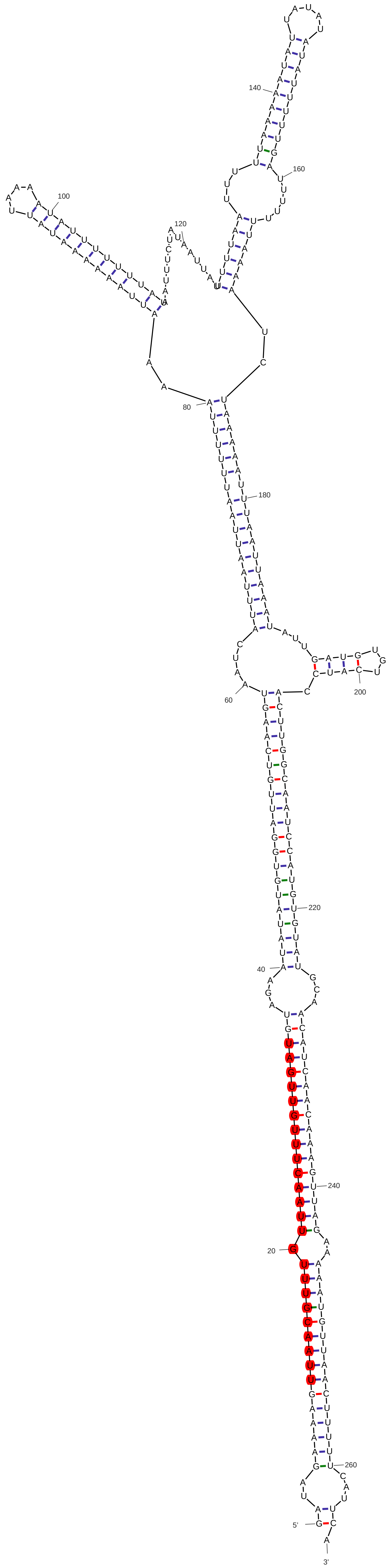

dG = -76.71 [Initially -82.90] novel_mir_1071_1

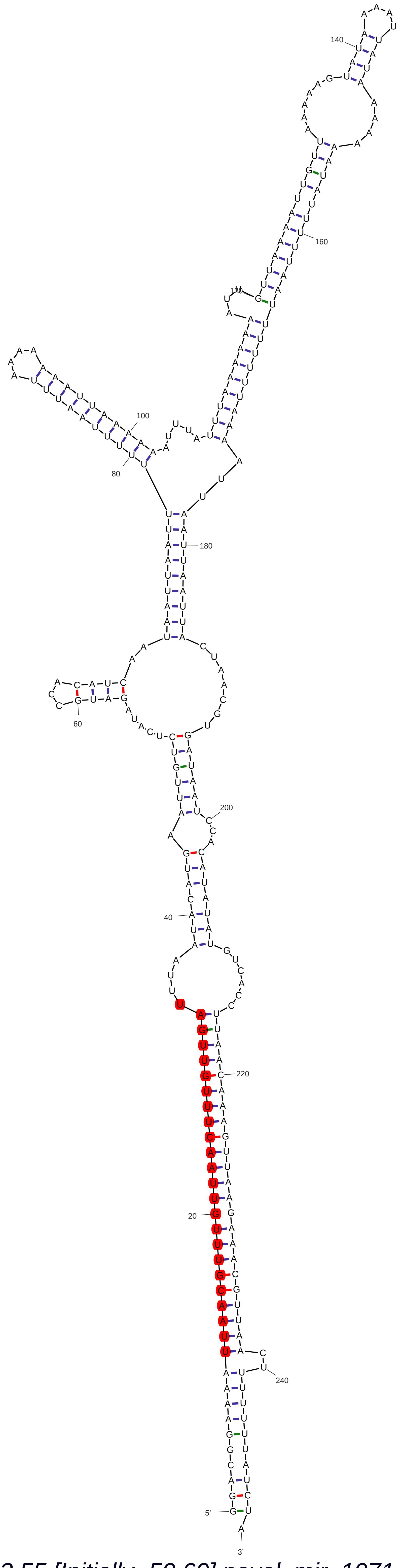

dG = -43.55 [Initially -50.60] novel_mir_1071_2

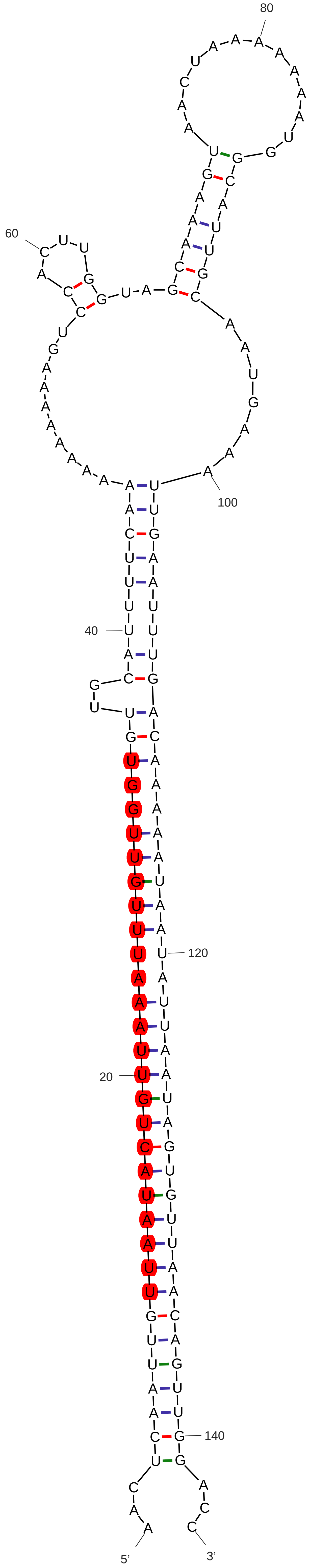

dG = -31.93 [Initially -36.00] novel_mir_4300

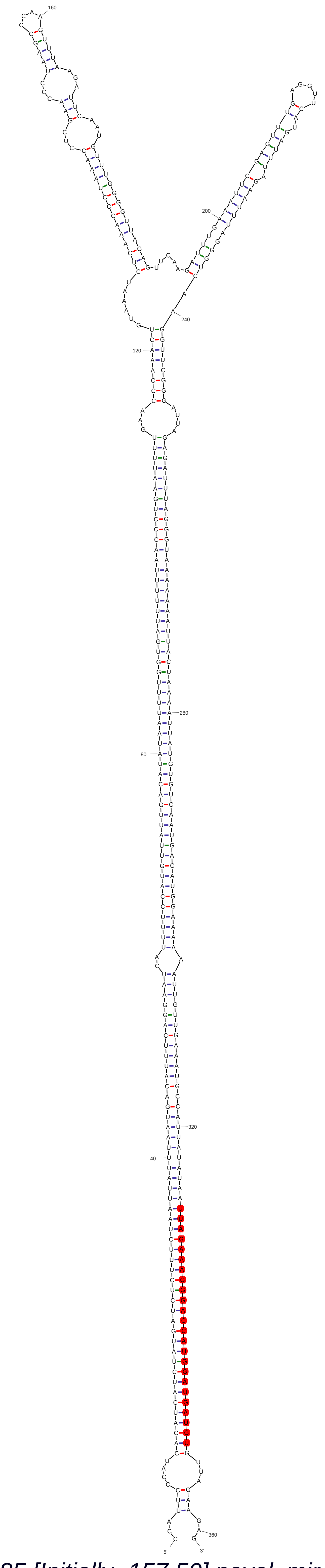

dG = -153.85 [Initially -157.50] novel_mir_4998

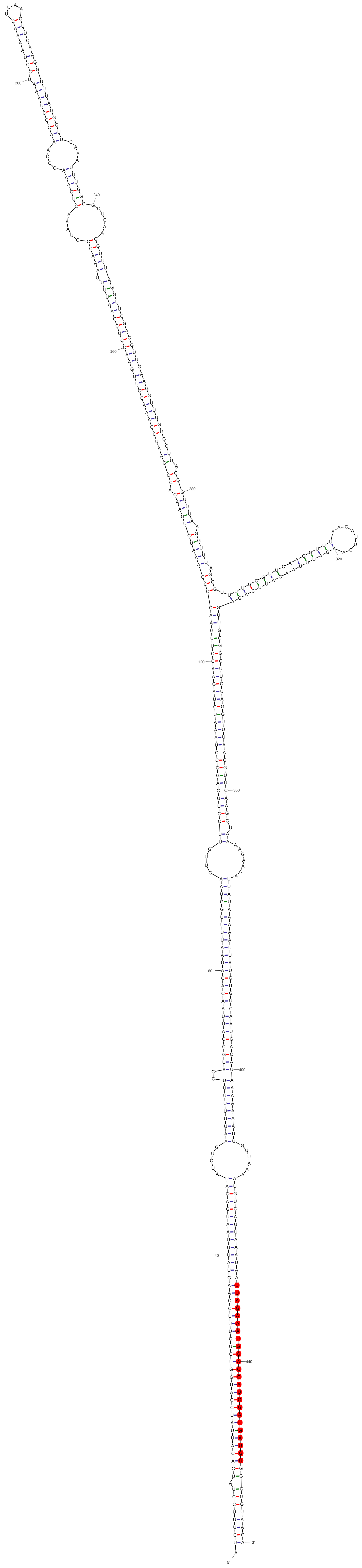

dG = -185.00 [Initially -186.40] novel_mir_68

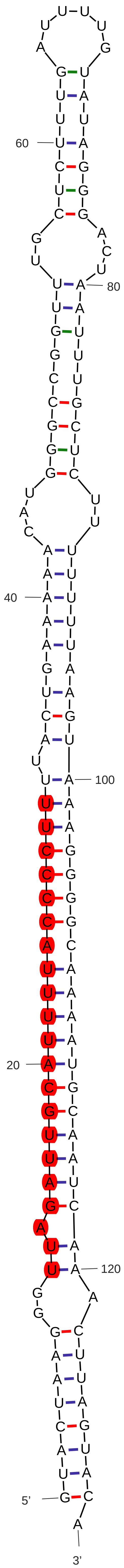

dG = -49.60 [Initially -49.60] novel_mir_24

Output of sir_graph (©)
mfold_util 4.6

Created Sun Jan 13 10:07:28 2013

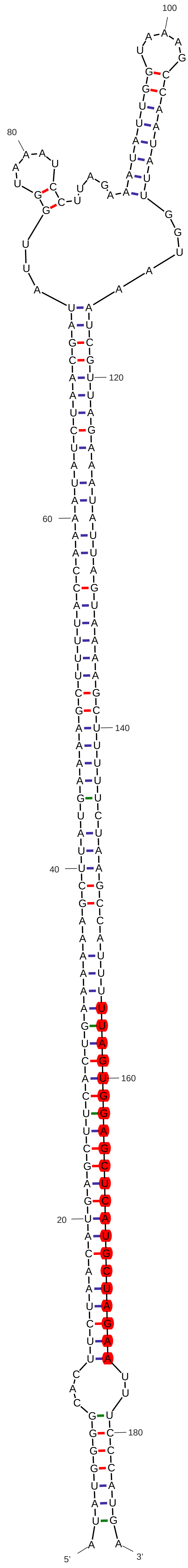

dG = -79.55 [Initially -82.60] novel_mir_4053

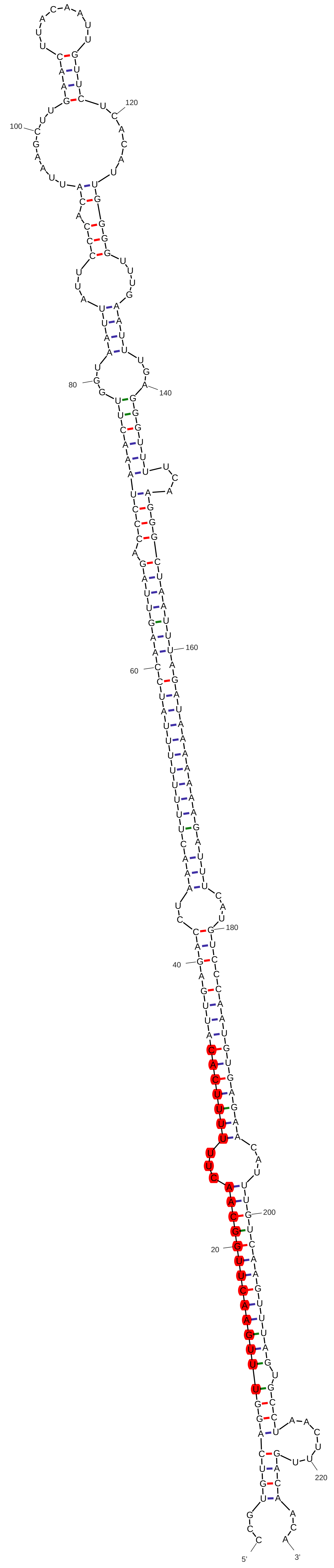

dG = -72.90 [Initially -72.90] novel_mir_2521

Output of sir_graph (©)
mfold_util 4.6

Created Sun Jan 13 10:07:25 2013

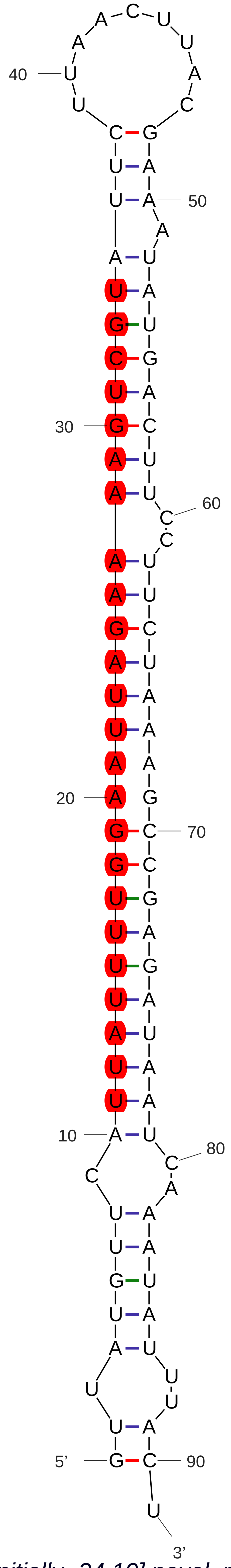

dG = -24.10 [Initially -24.10] novel_mir_1073

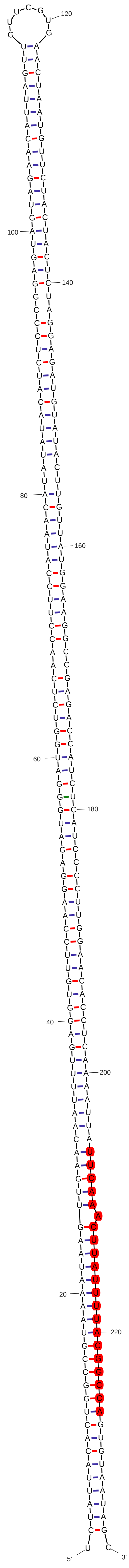

dG = -174.10 [Initially -174.10] novel_mir_2480

Output of sir_graph (©)
mfold_util 4.6

Created Sun Jan 13 10:15:56 2013

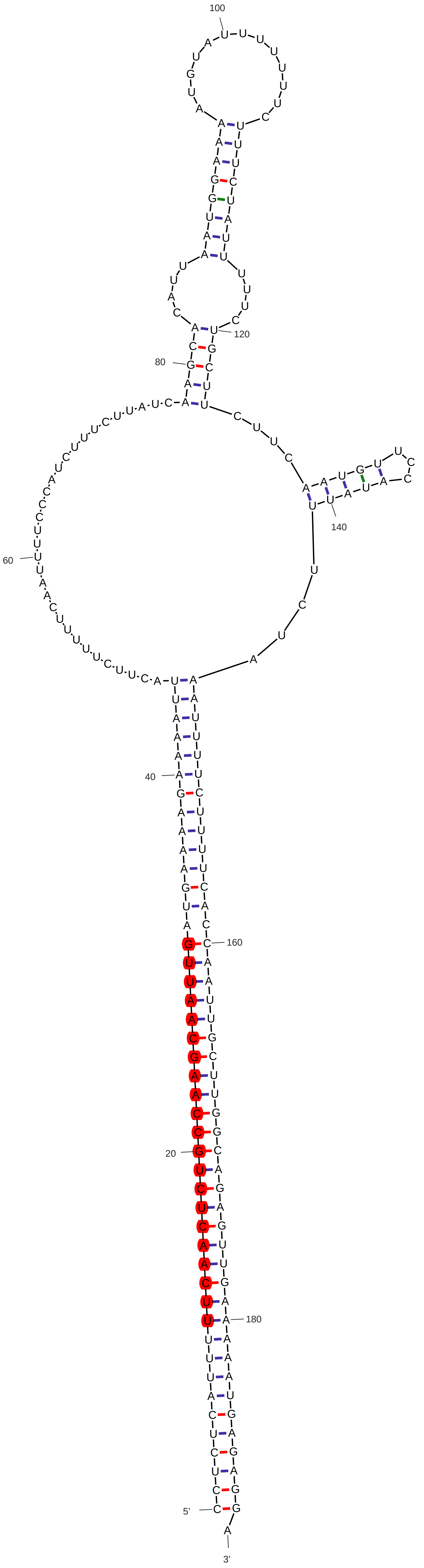

dG = -72.51 [Initially -76.50] novel_mir_987_1

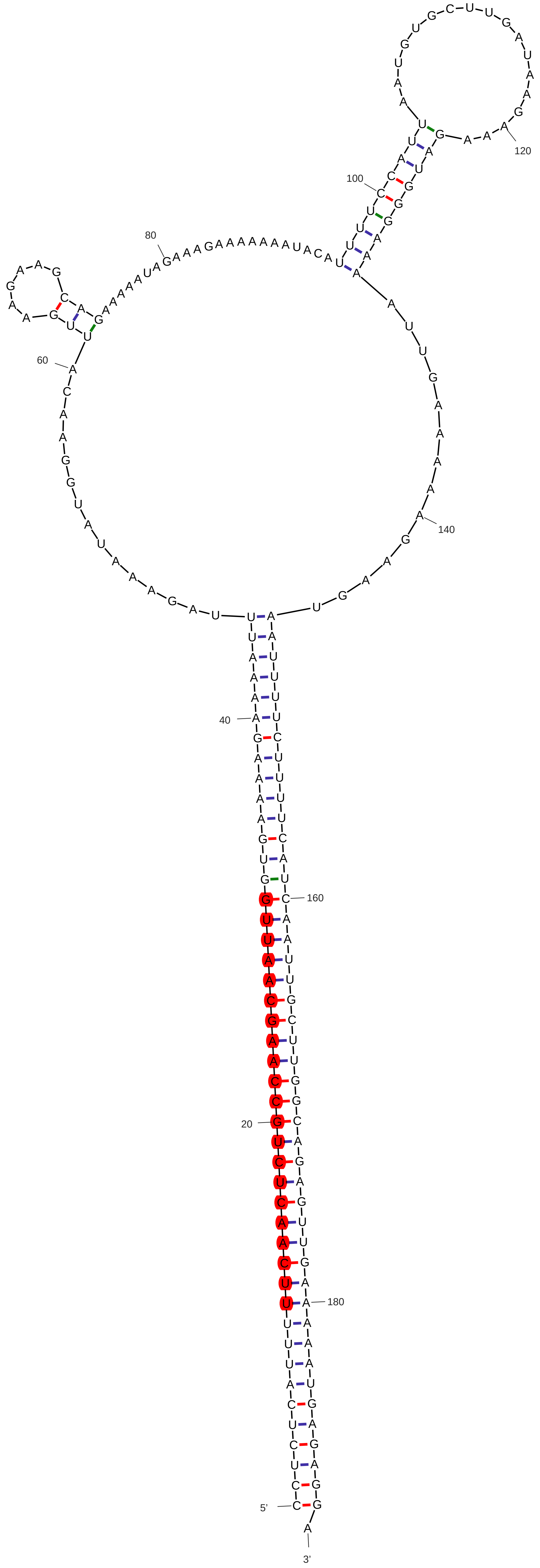

dG = -73.82 [Initially -78.90] novel_mir_987_2

Output of sir_graph (©)
mfold_util 4.6

Created Sun Jan 13 10:16:00 2013

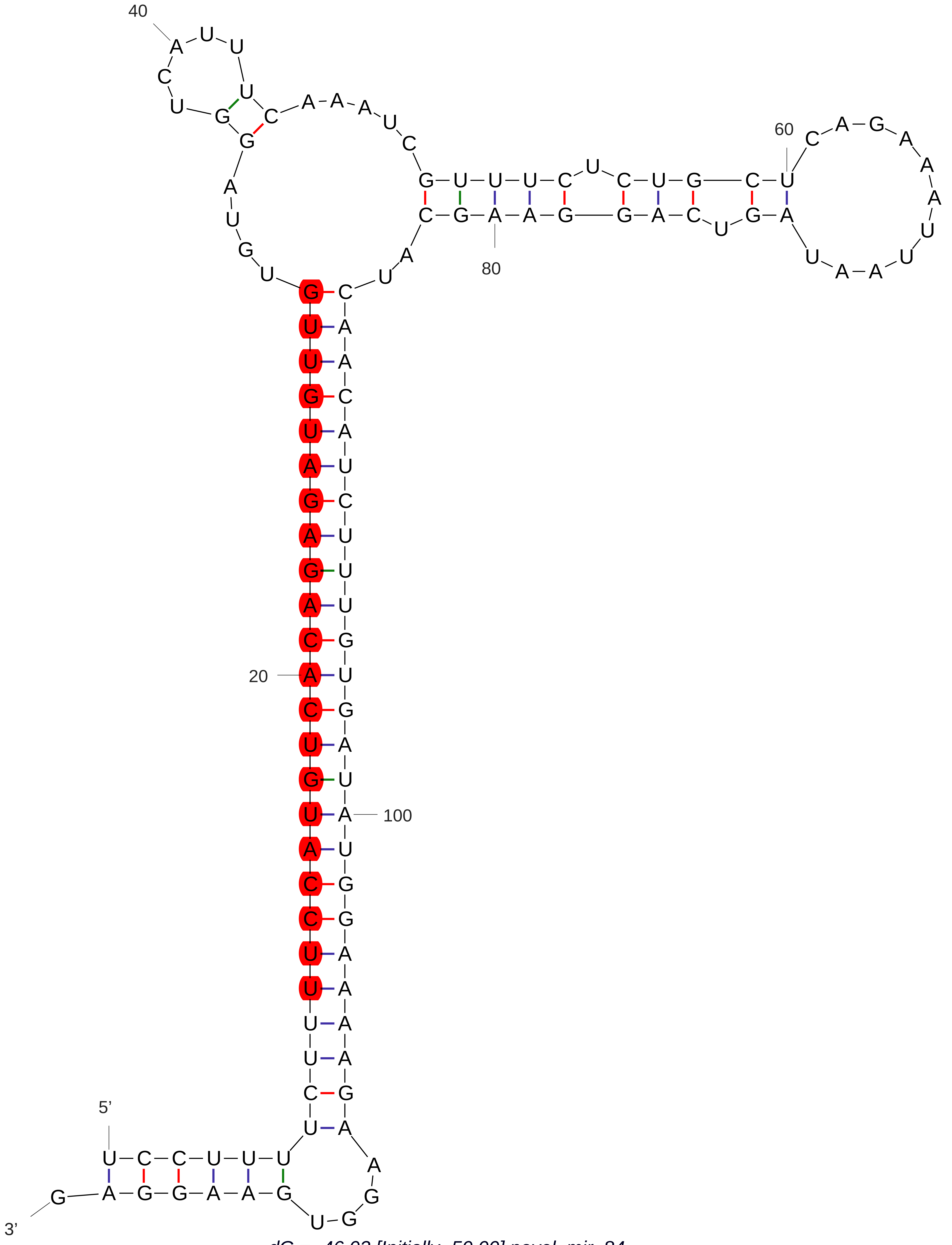

dG = -46.03 [Initially -50.00] novel_mir_84

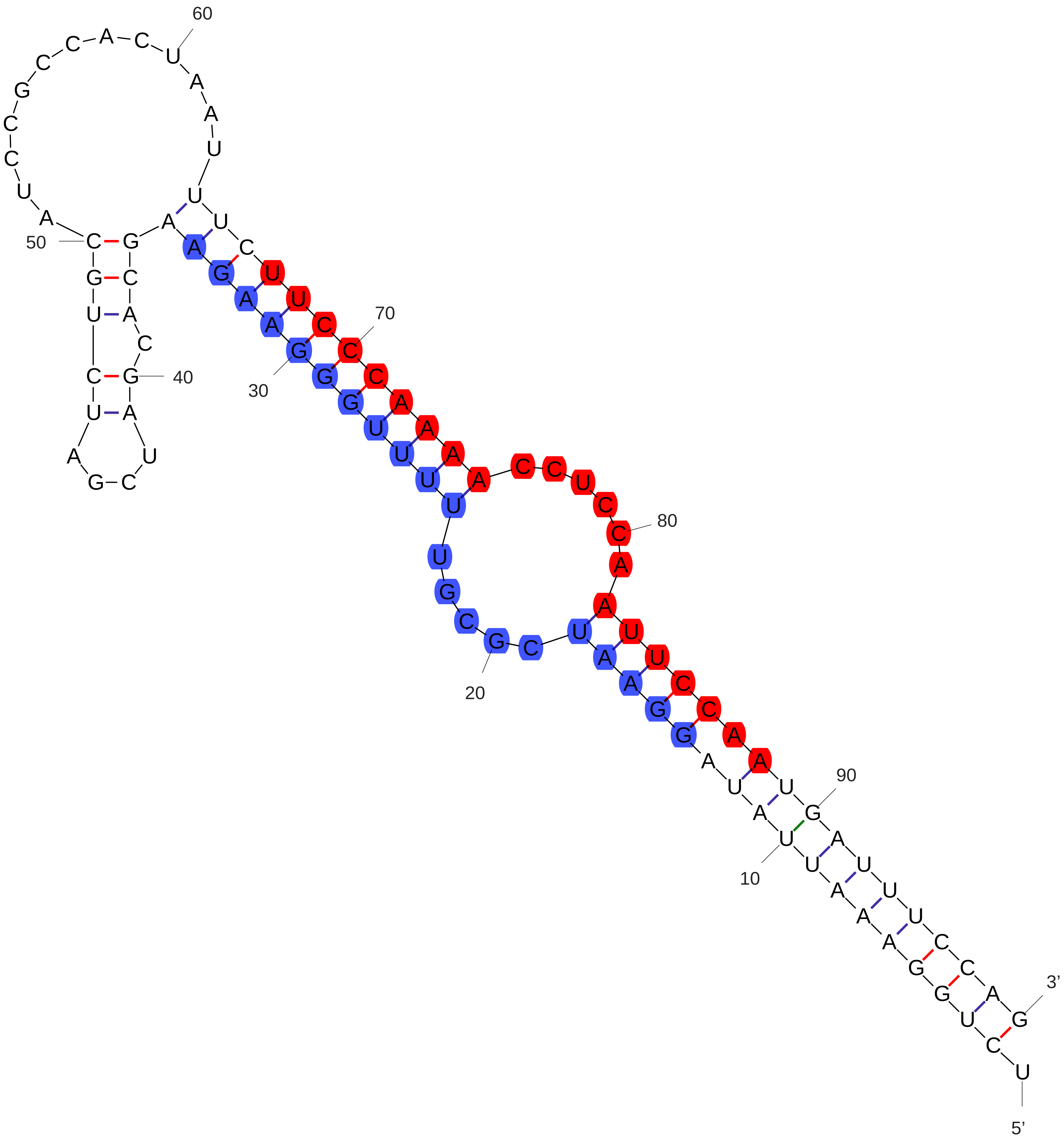

dG = -33.70 [Initially -33.70] novel_mir_1437

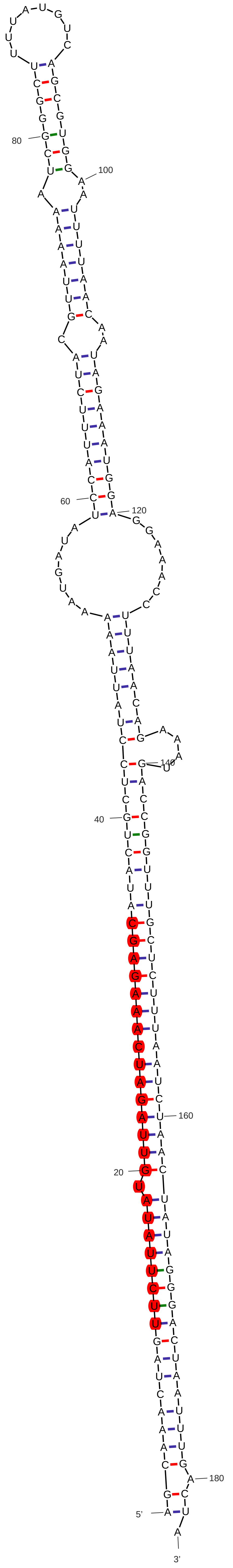

dG = -63.00 [Initially -63.00] novel_mir_2621

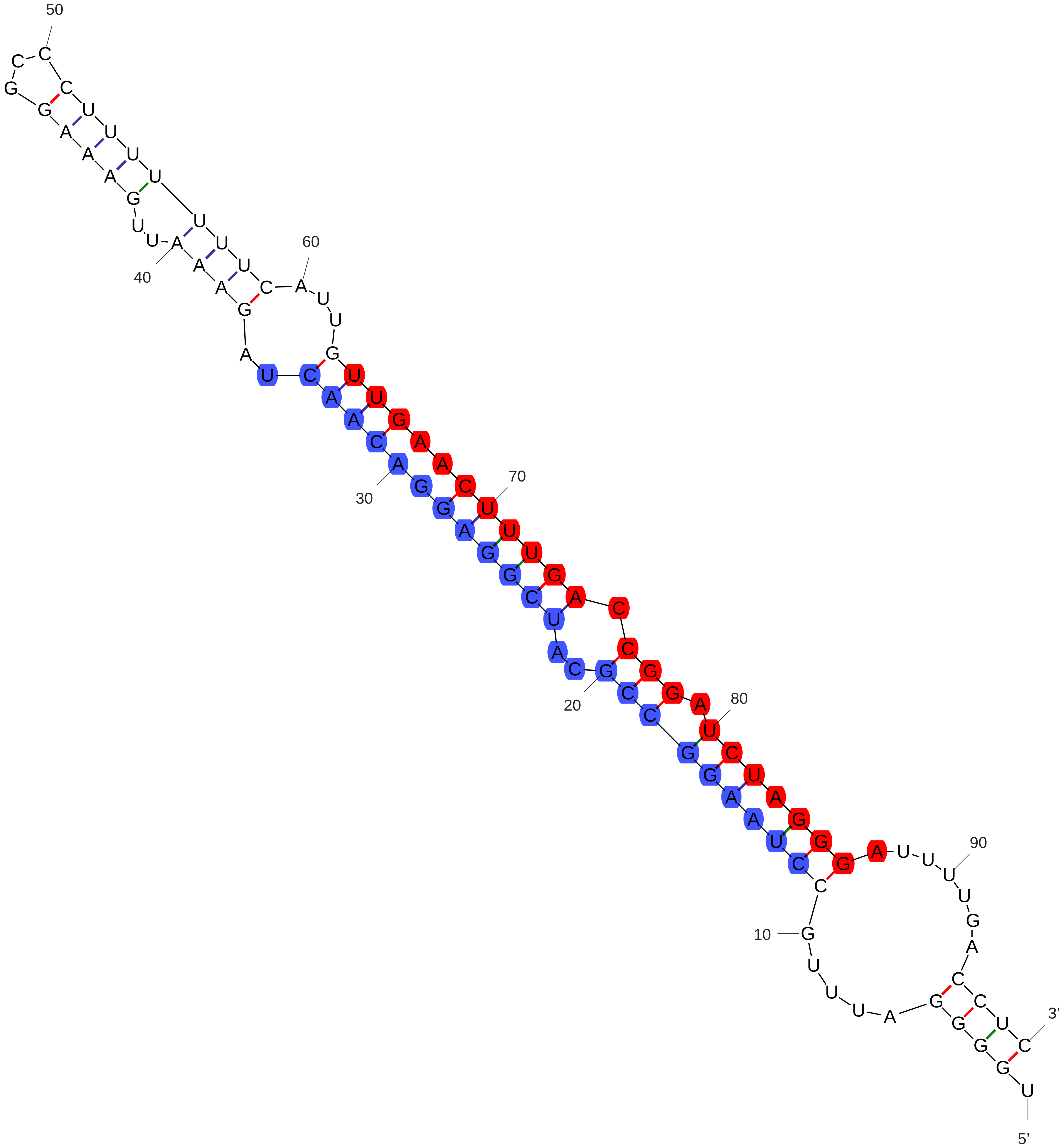

dG = -24.70 [Initially -24.70] novel_mir_2943

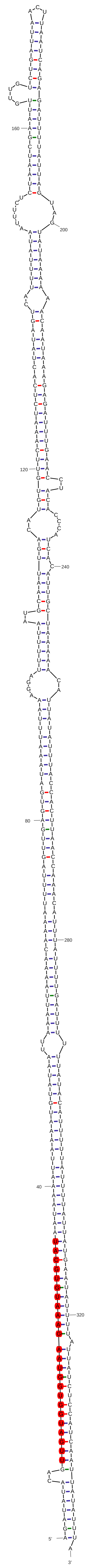

dG = -96.80 [Initially -96.80] novel_mir_905

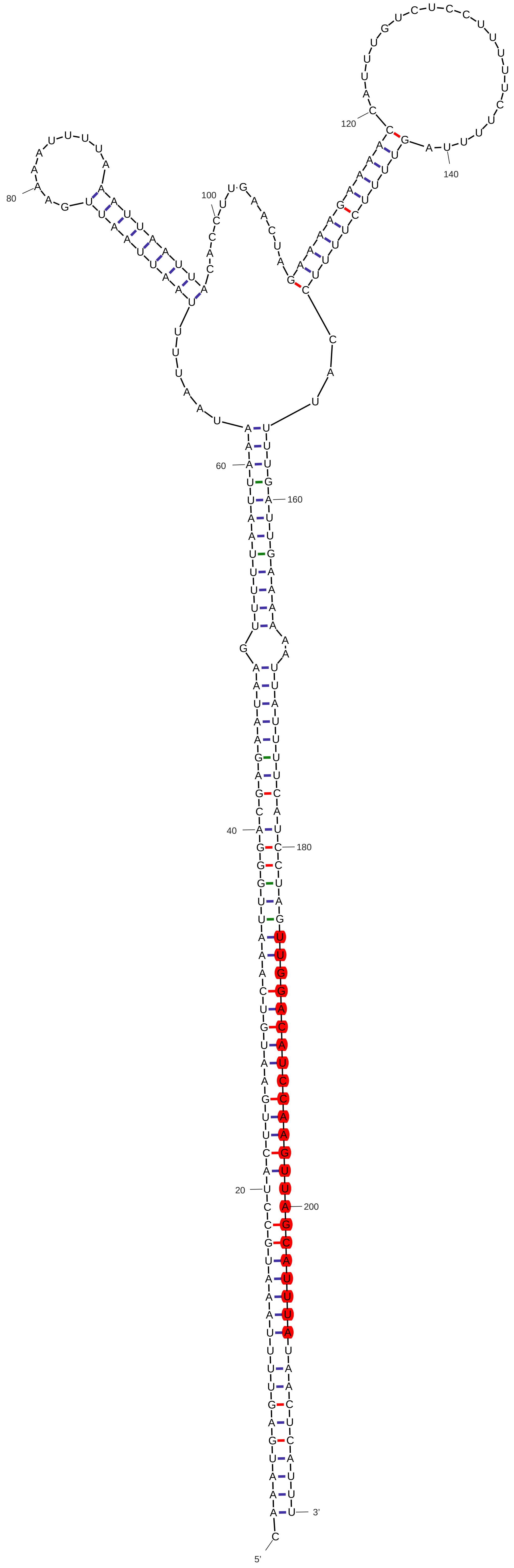

dG = -64.12 [Initially -67.80] novel_mir_2894

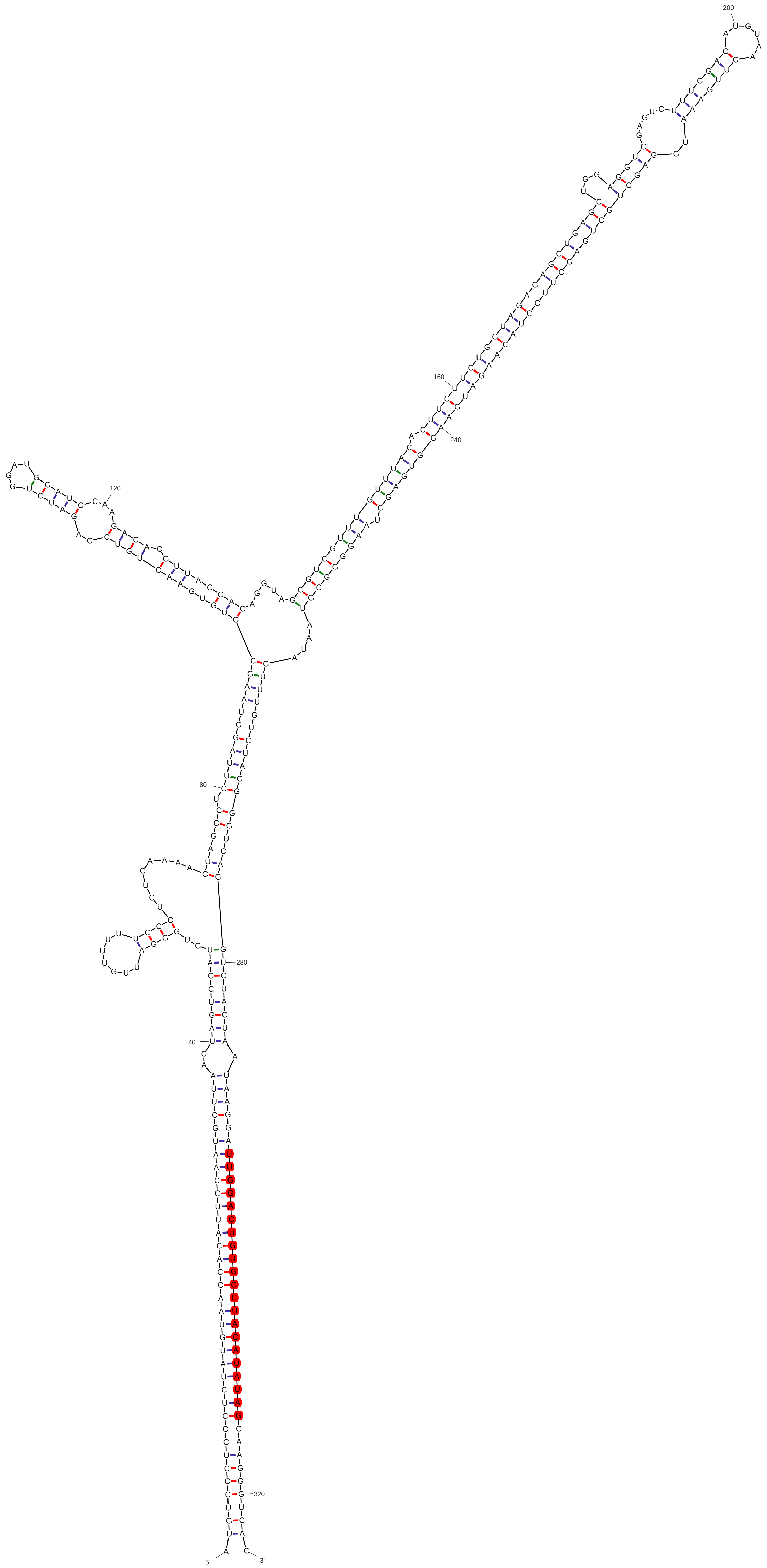

dG = -114.79 [Initially -120.10] novel_mir_862

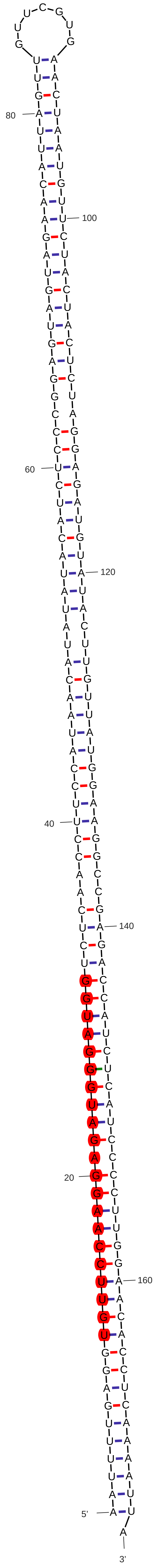

dG = -129.40 [Initially -129.40] novel_mir_22

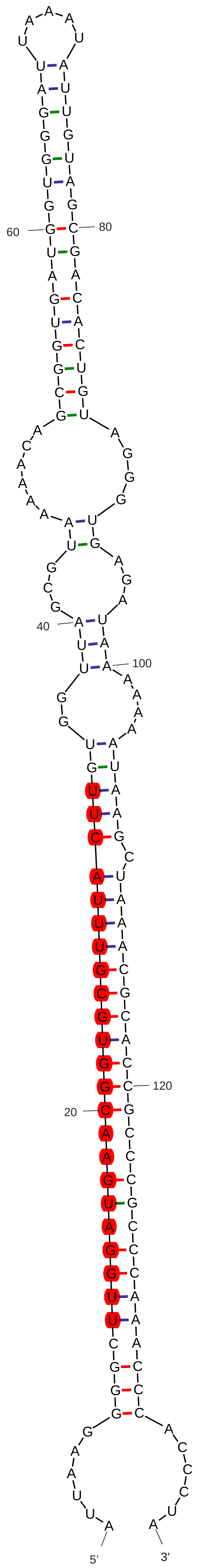

dG = -44.40 [Initially -44.40] novel_mir_2744

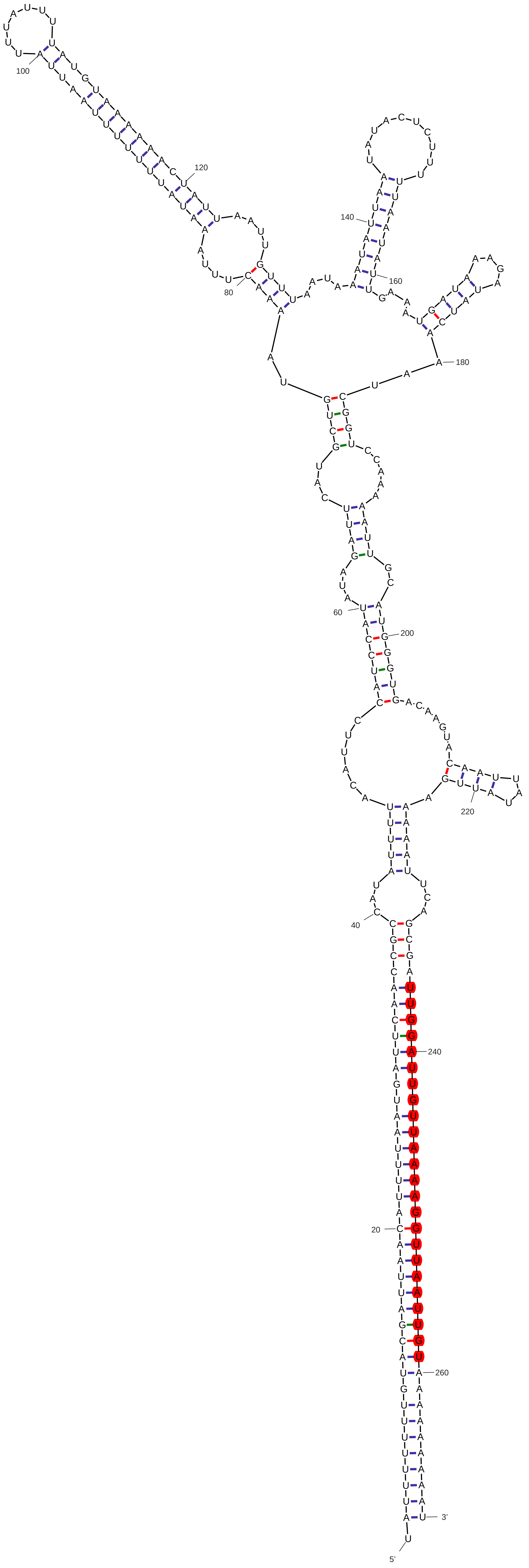

dG = -44.72 [Initially -51.20] novel_mir_4245

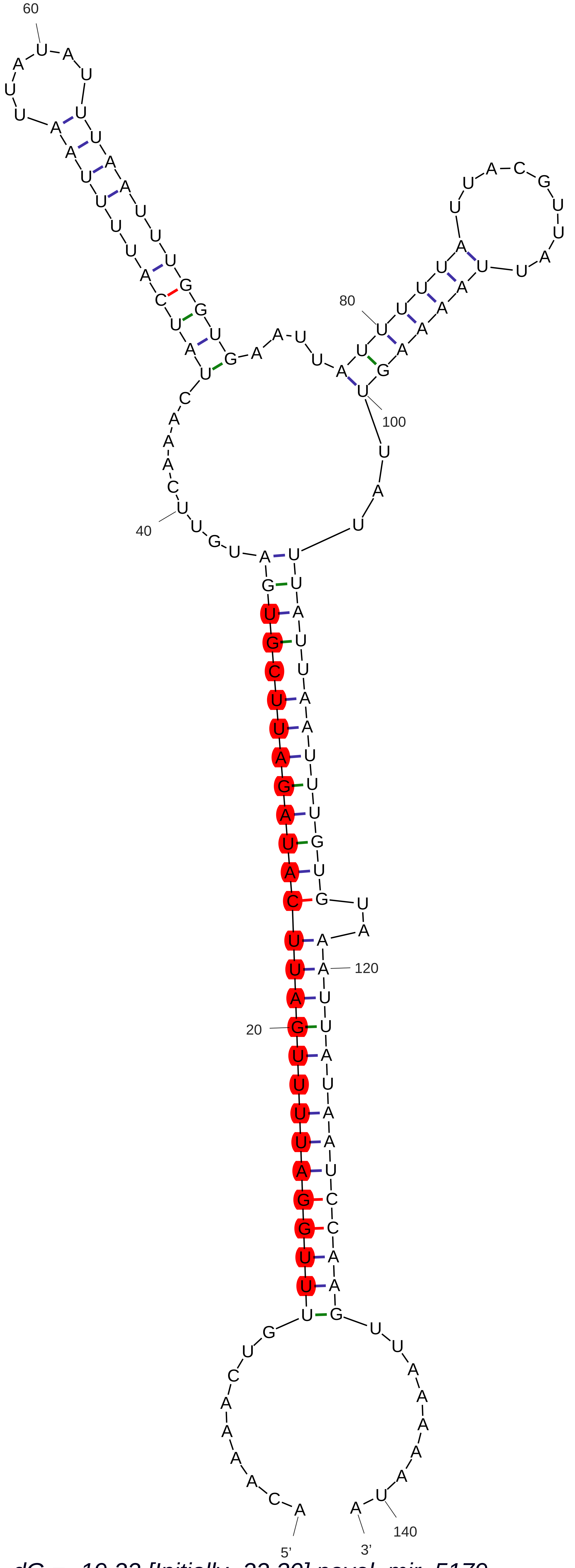

dG = -19.22 [Initially -22.30] novel_mir_5179

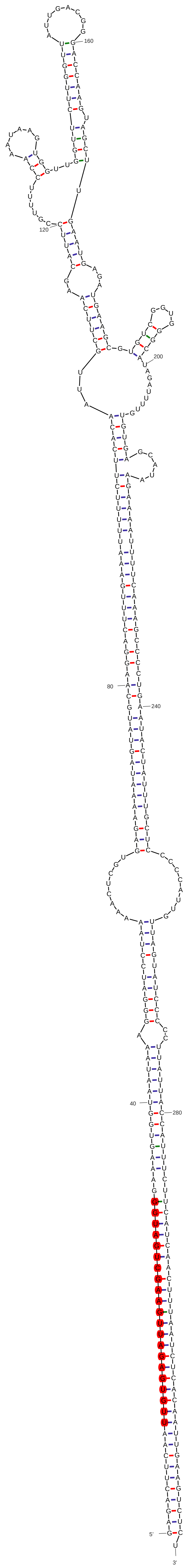

dG = -129.10 [Initially -132.80] novel_mir_5690

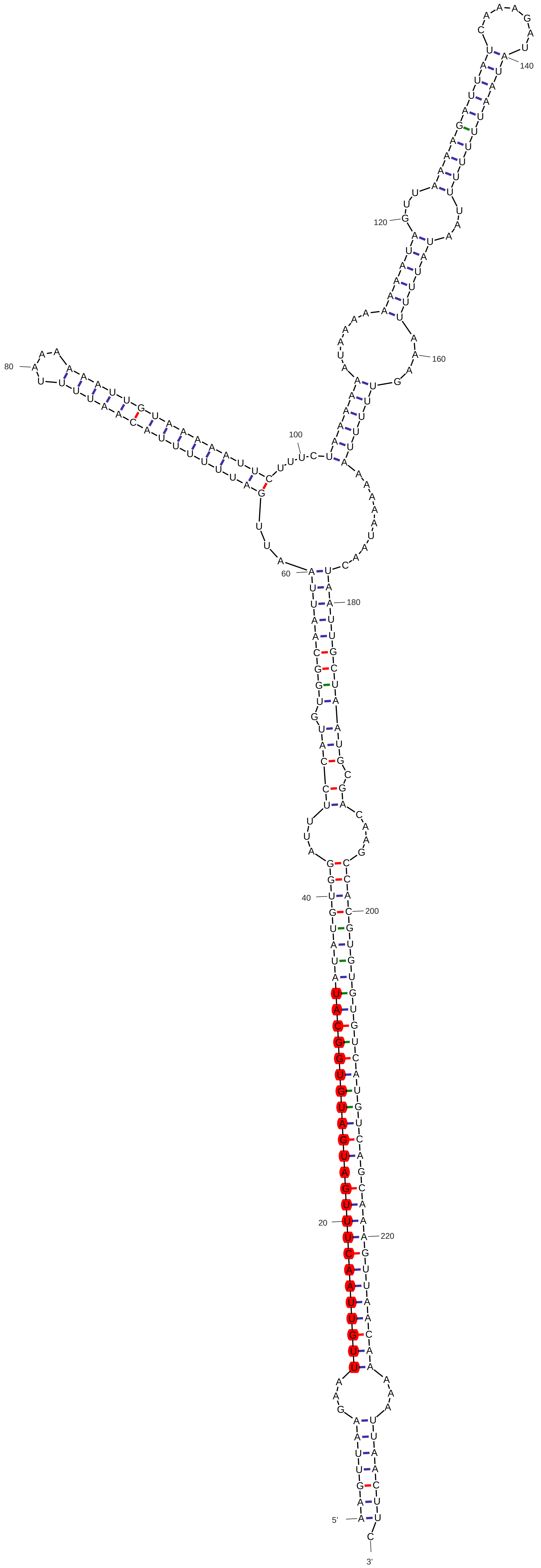

dG = -74.92 [Initially -78.10] novel_mir_3001

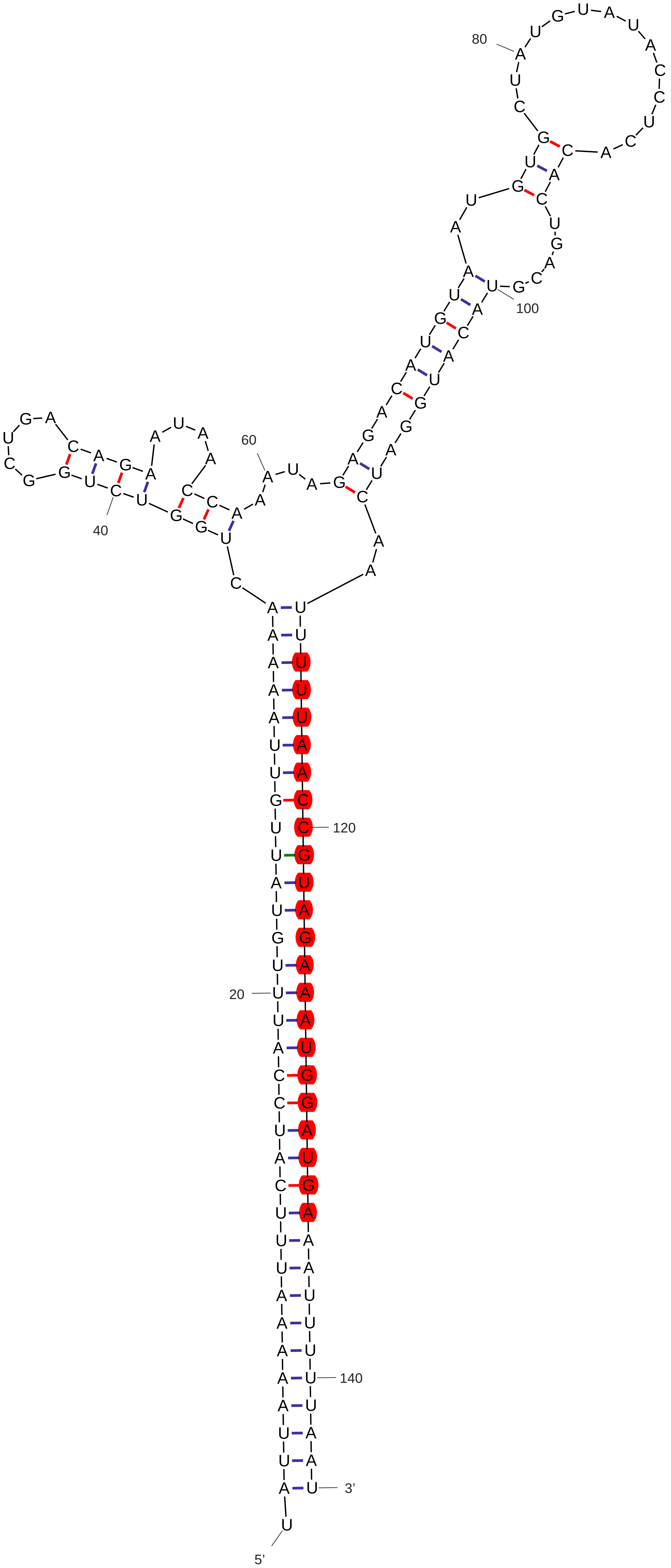

dG = -45.43 [Initially -46.60] novel_mir_968_1

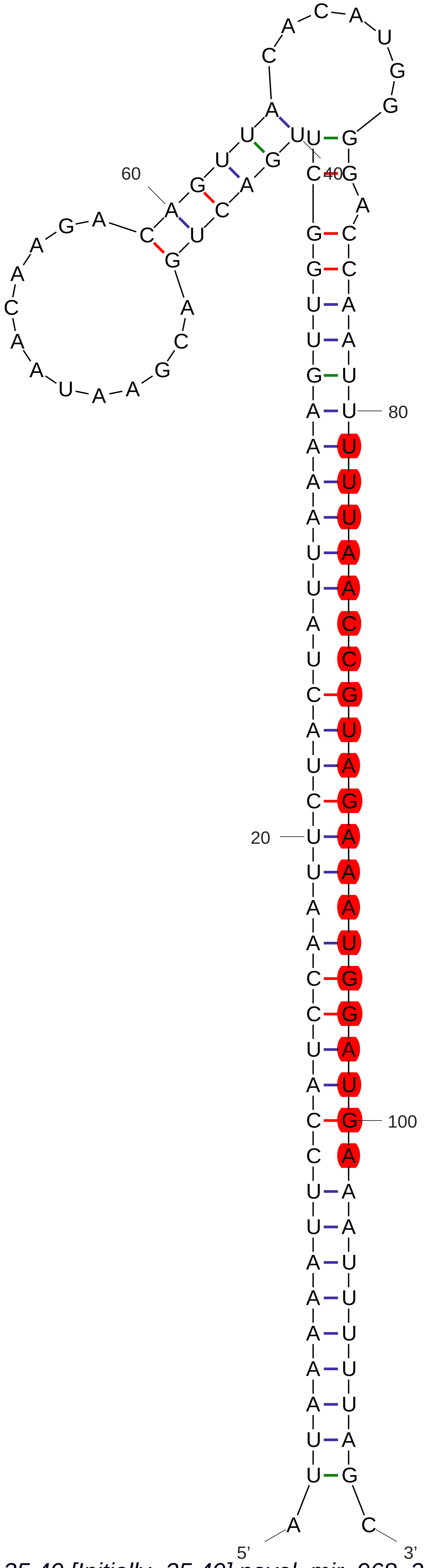

dG = -35.40 [Initially -35.40] novel_mir_968_2

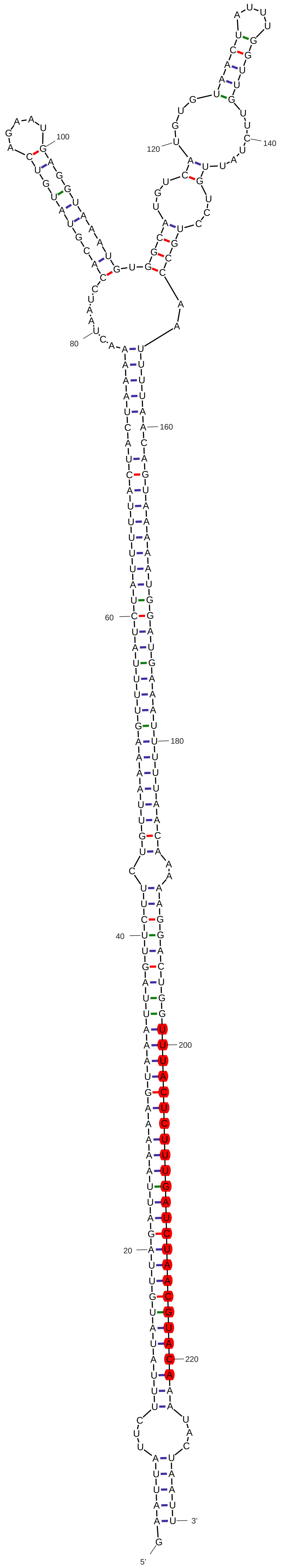

dG = -71.34 [Initially -74.40] novel_mir_2630

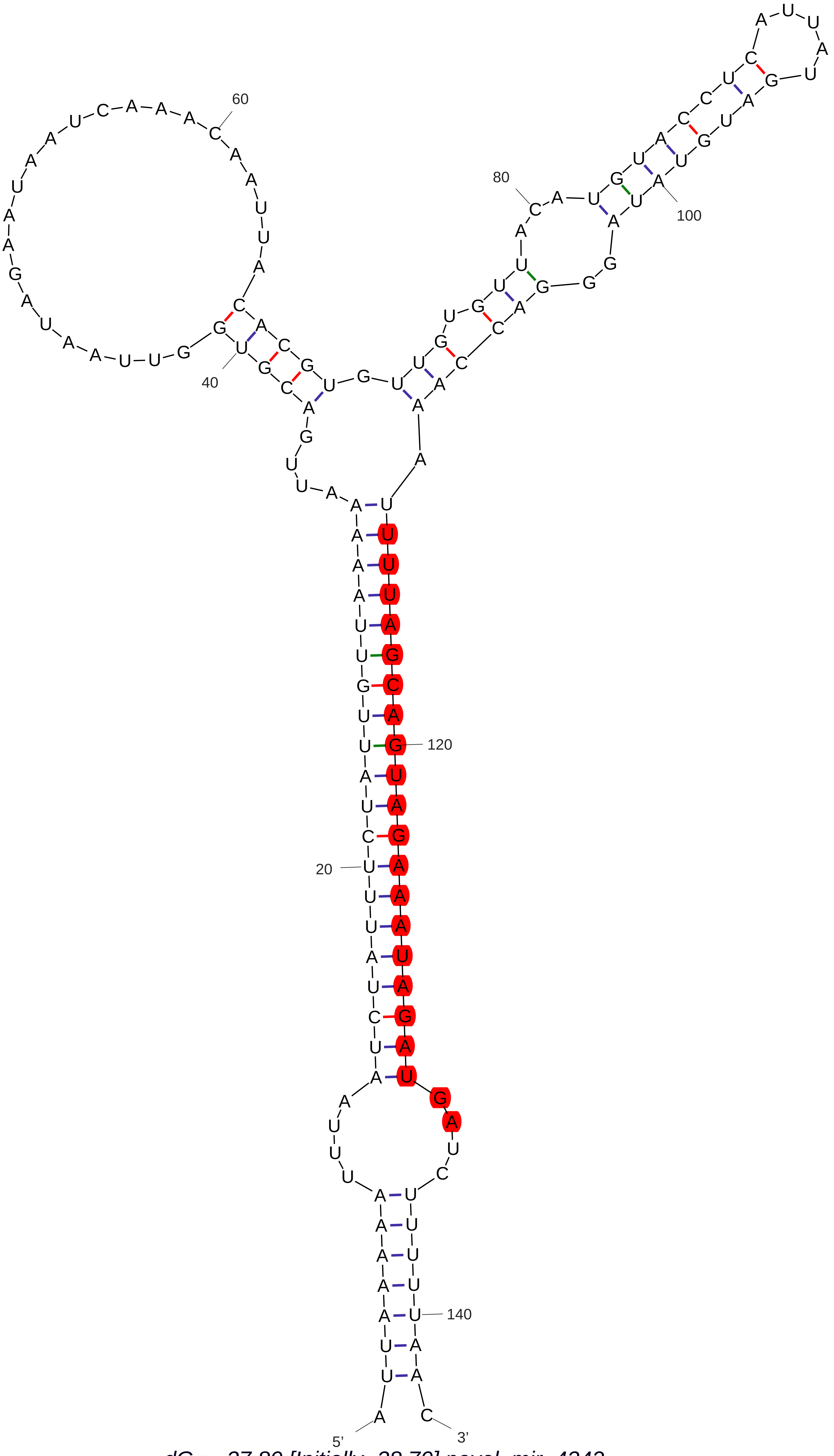

dG = -37.80 [Initially -38.70] novel_mir_4342

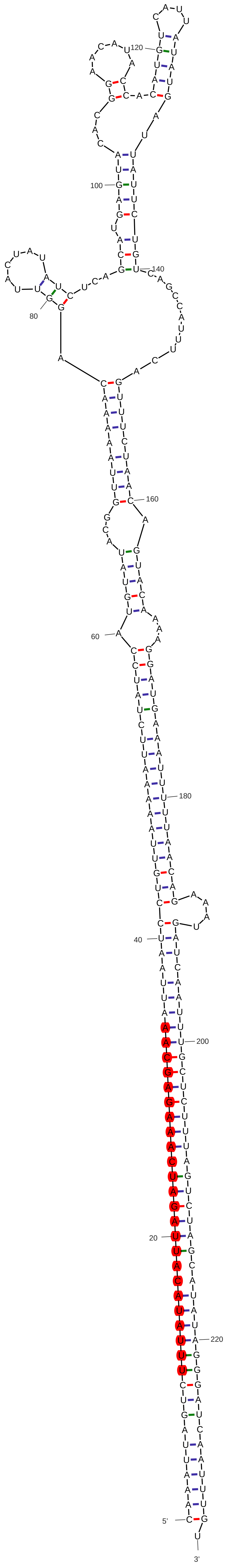

dG = -59.49 [Initially -65.40] novel_mir_153

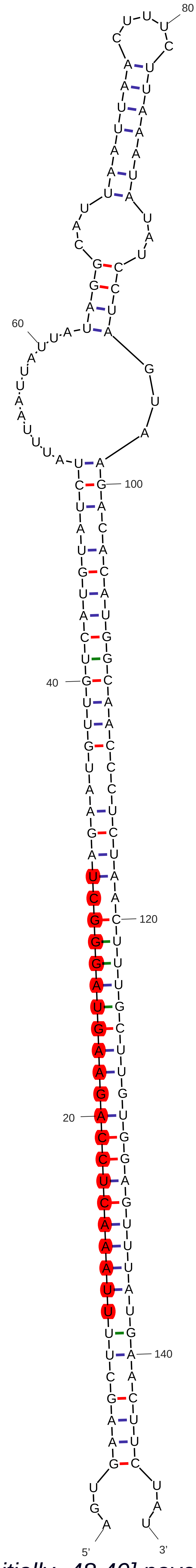

dG = -48.40 [Initially -48.40] novel_mir_886

Output of sir_graph (©)
mfold_util 4.6

Created Sun Jan 13 23:05:12 2013

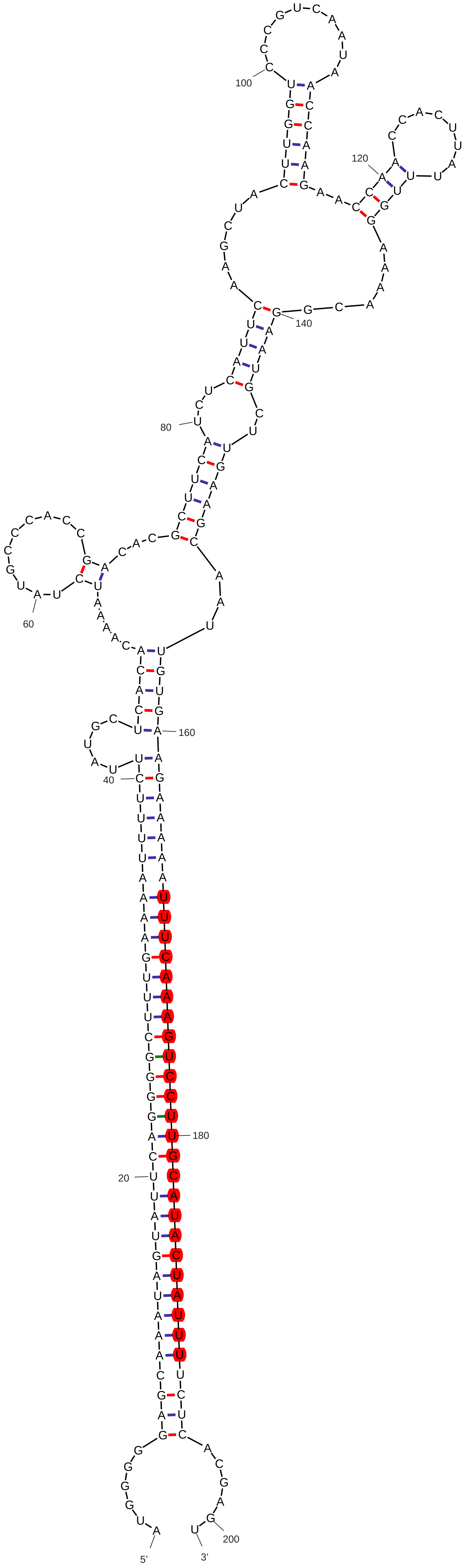

dG = -54.10 [Initially -62.30] novel_mir_885_1

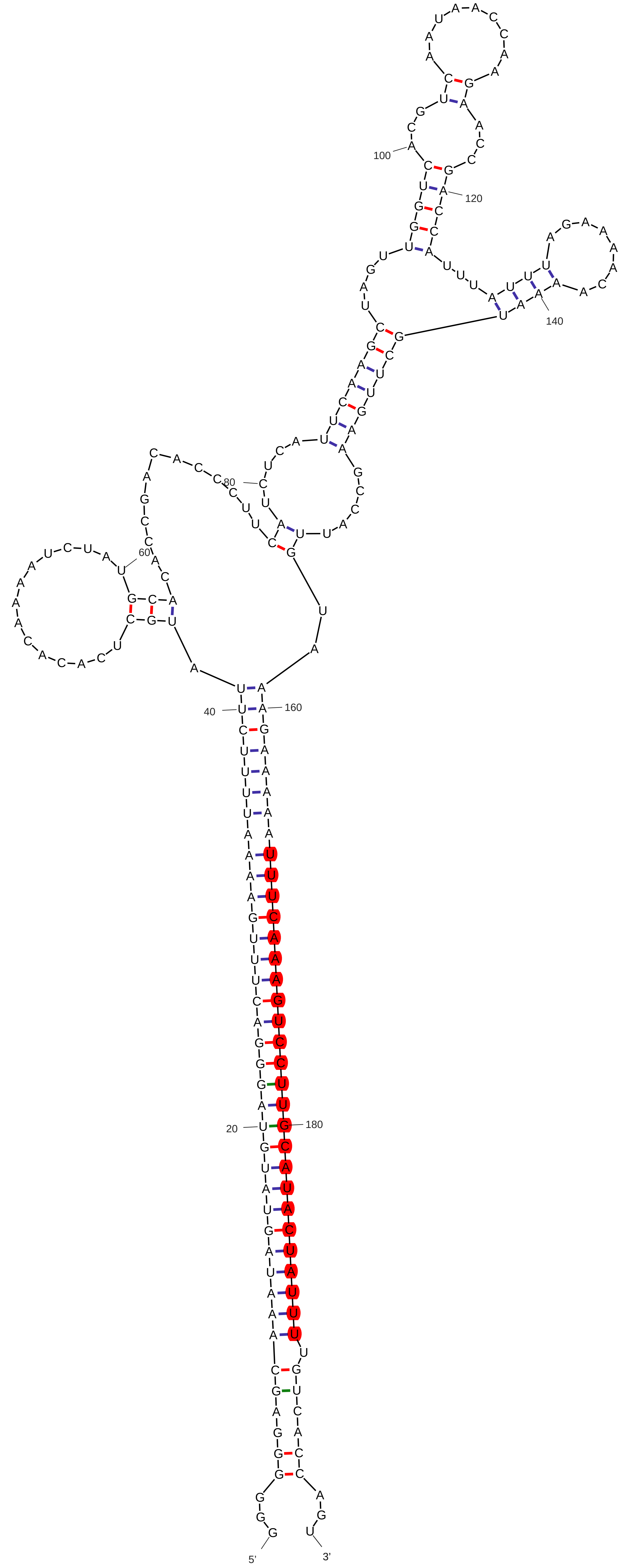

dG = -54.65 [Initially -59.10] novel_mir_885_2

Output of sir_graph (©)
mfold_util 4.6

Created Sun Jan 13 23:05:22 2013

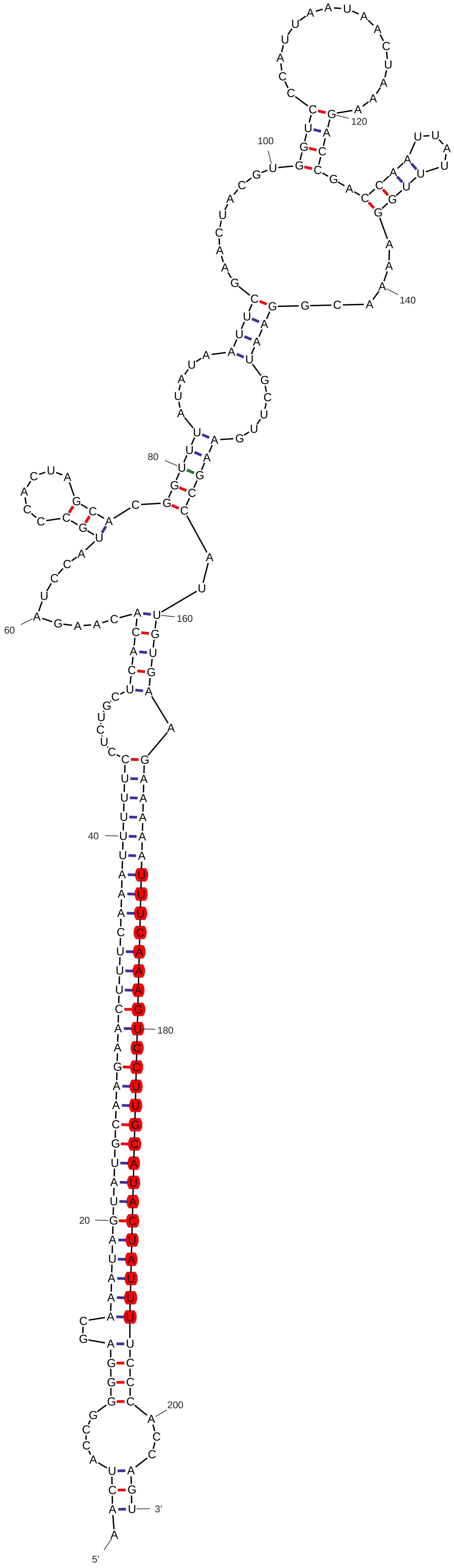

dG = -49.89 [Initially -57.00] novel_mir_885_3

Output of sir_graph (©)
mfold_util 4.6

Created Sun Jan 13 23:05:28 2013

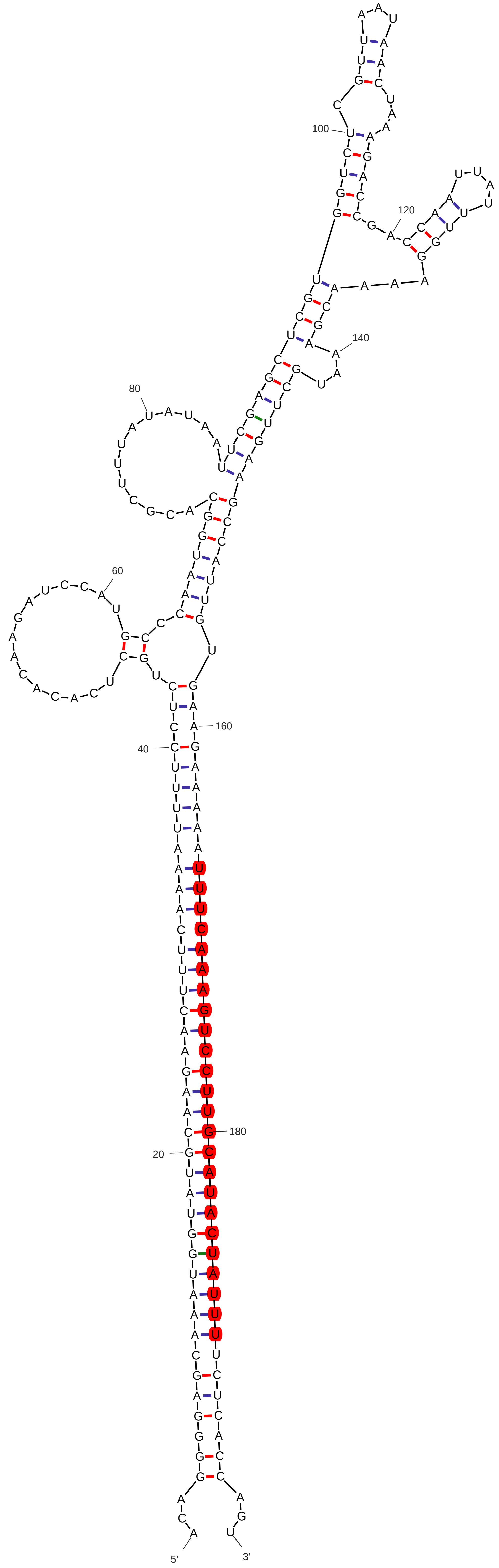

dG = -56.00 [Initially -61.50] novel_mir_885_4

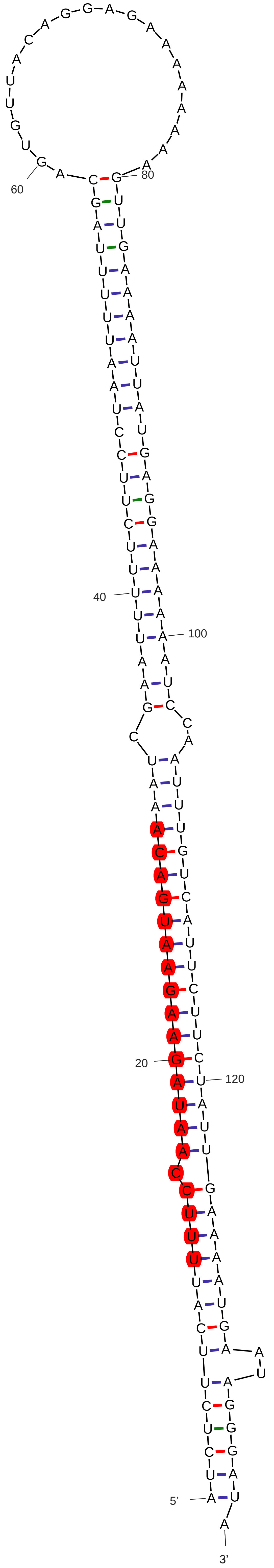

dG = -54.80 [Initially -54.80] novel_mir_1004

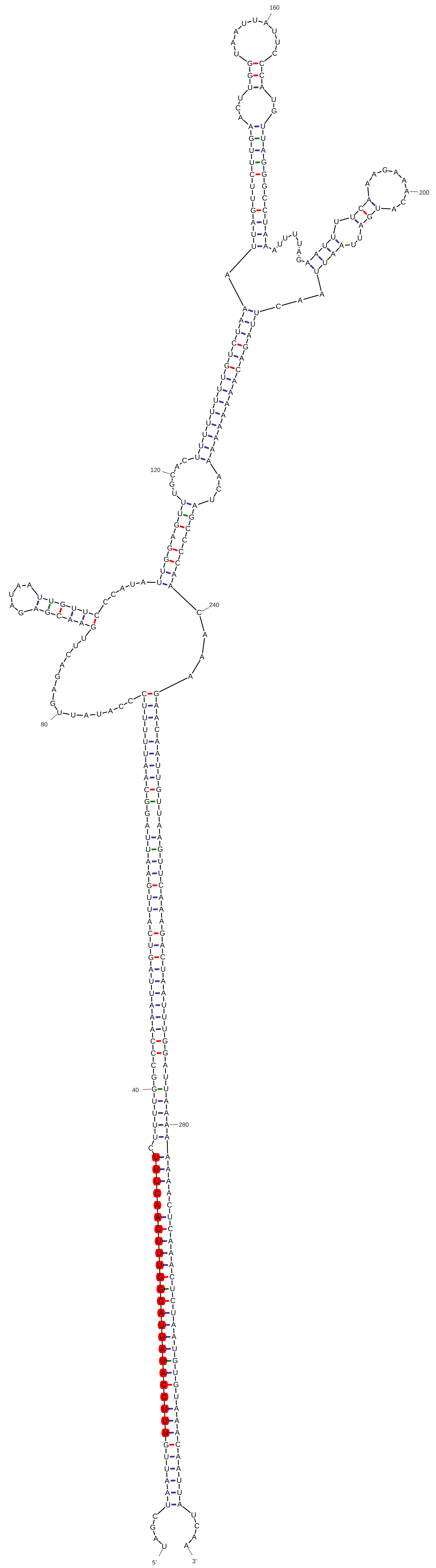

dG = -85.45 [Initially -92.20] novel_mir_5145

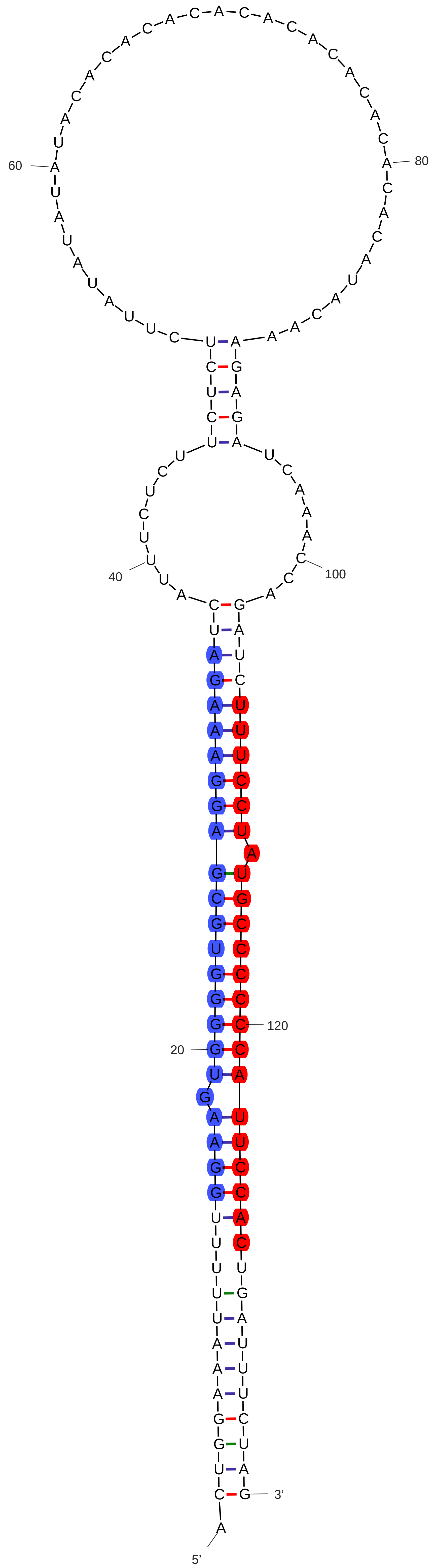

dG = -46.32 [Initially -46.32] novel_mir_69

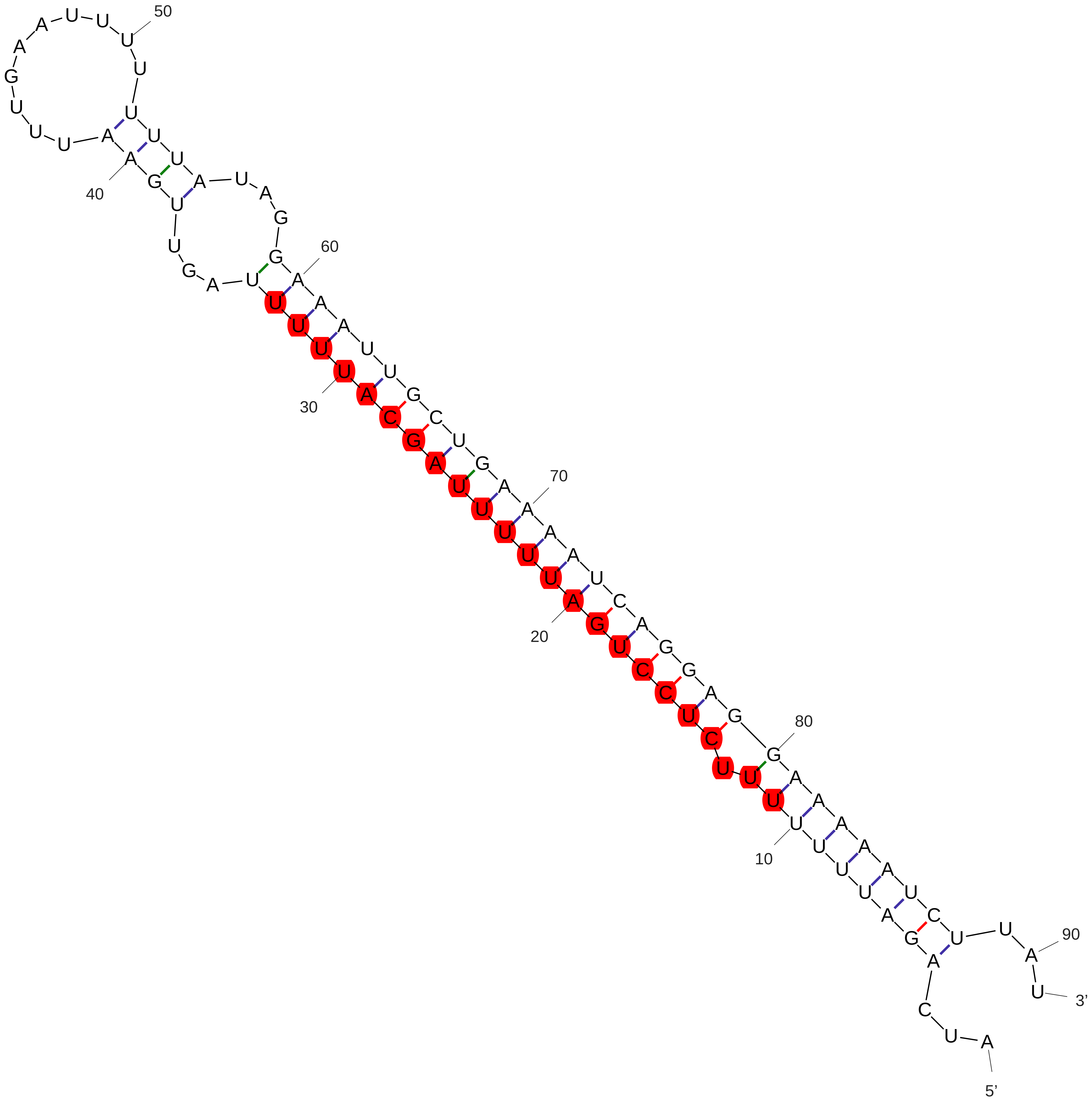

dG = -34.30 [Initially -34.30] novel_mir_4989_1

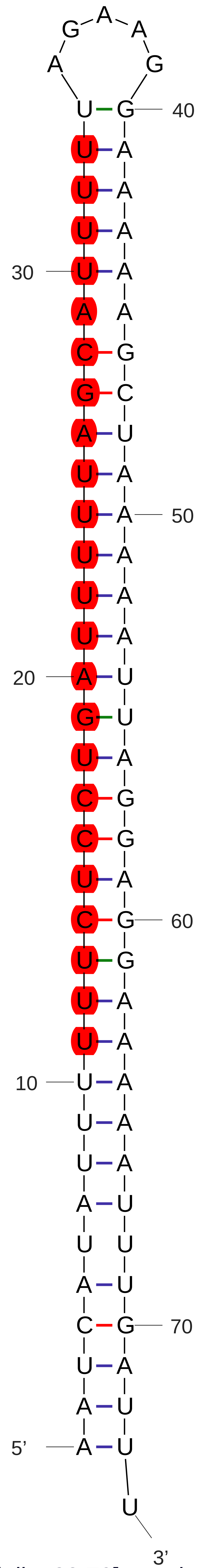

dG = -33.70 [Initially -33.70] novel_mir_4989_2

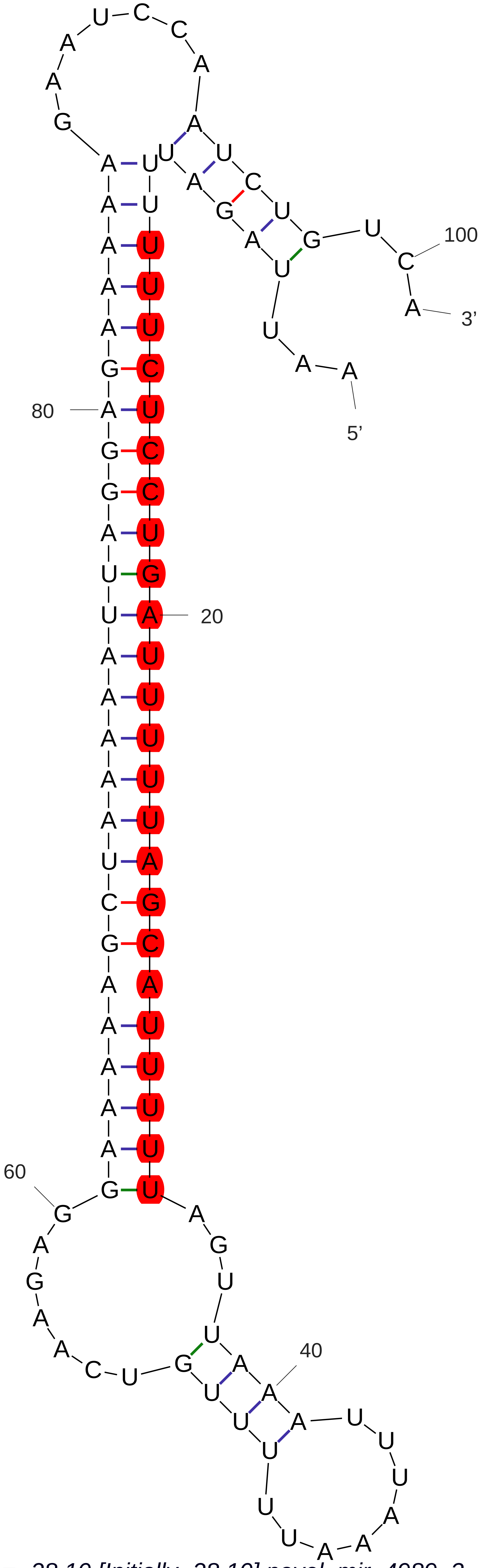

dG = -28.10 [Initially -28.10] novel_mir_4989_3

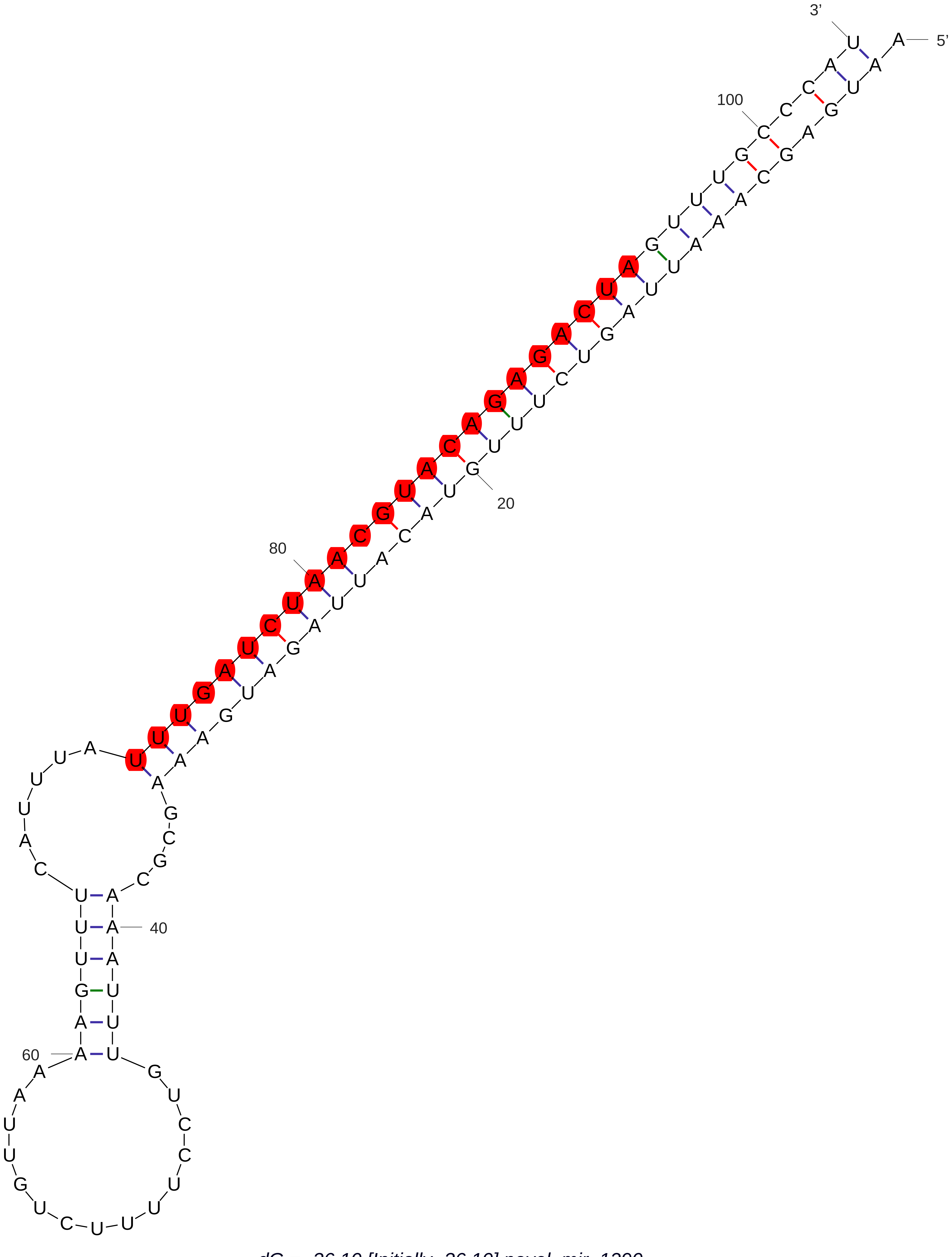

dG = -36.10 [Initially -36.10] novel_mir_1290

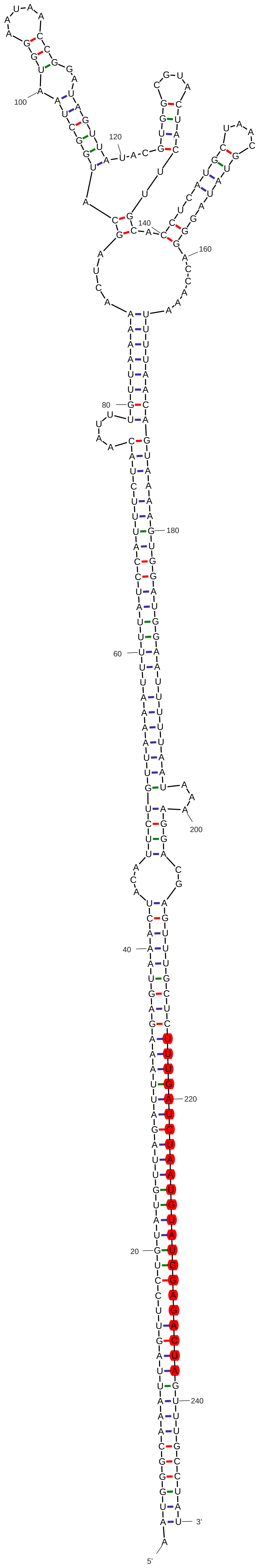

dG = -91.43 [Initially -97.30] novel_mir_5150

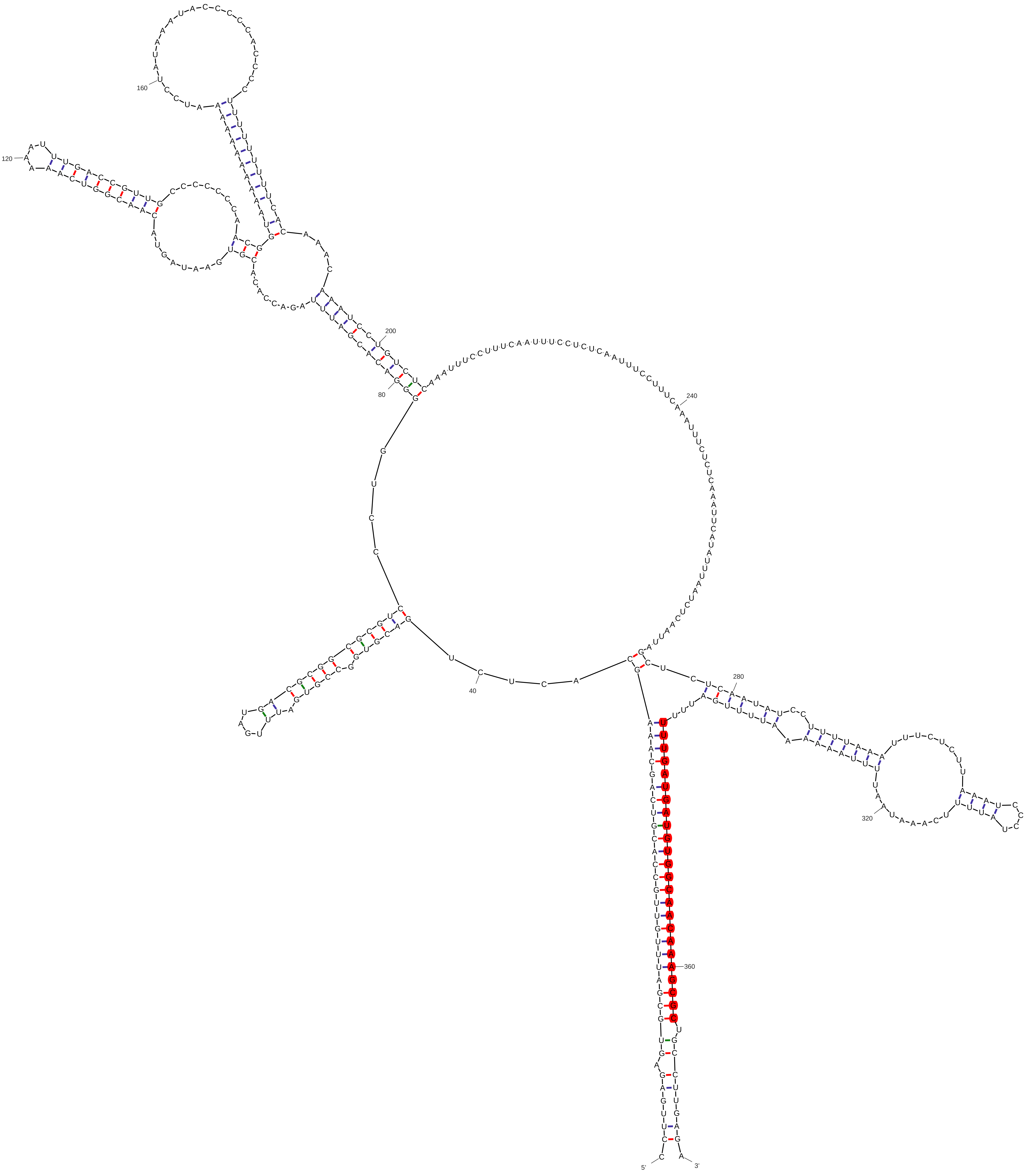

dG = -77.93 [Initially -87.70] novel_mir_823

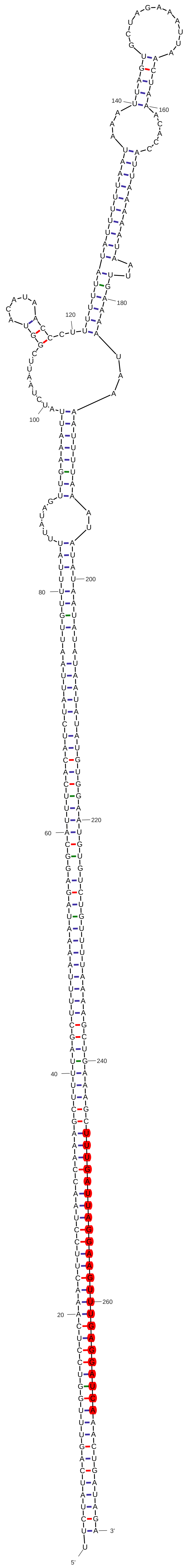

dG = -113.57 [Initially -116.80] novel_mir_4068

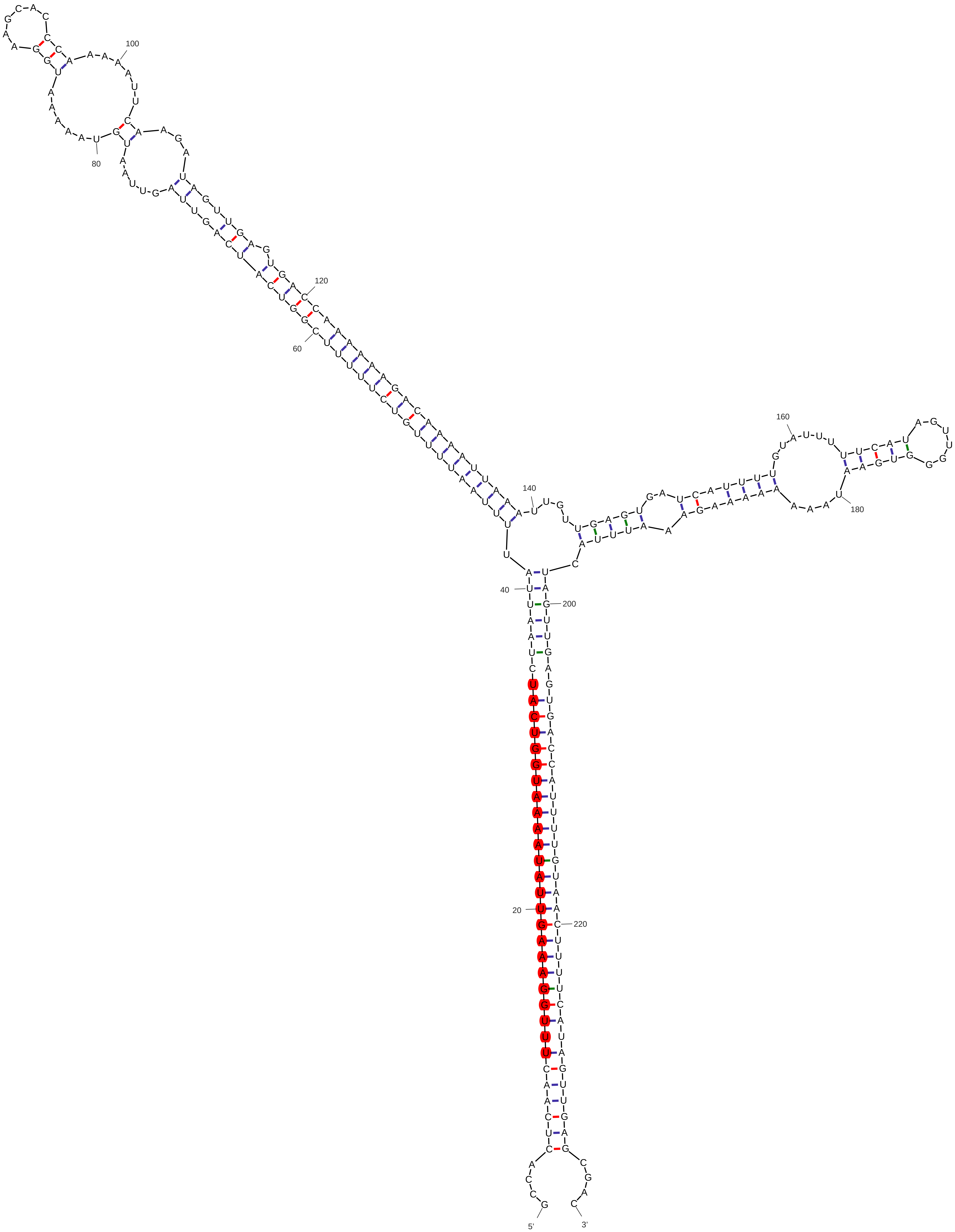

dG = -75.90 [Initially -77.80] novel_mir_1000

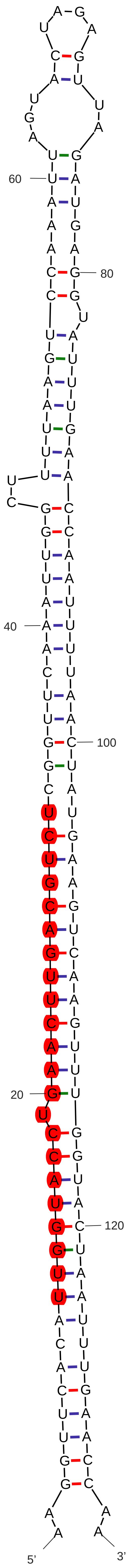

dG = -51.10 [Initially -51.10] novel_mir_2555

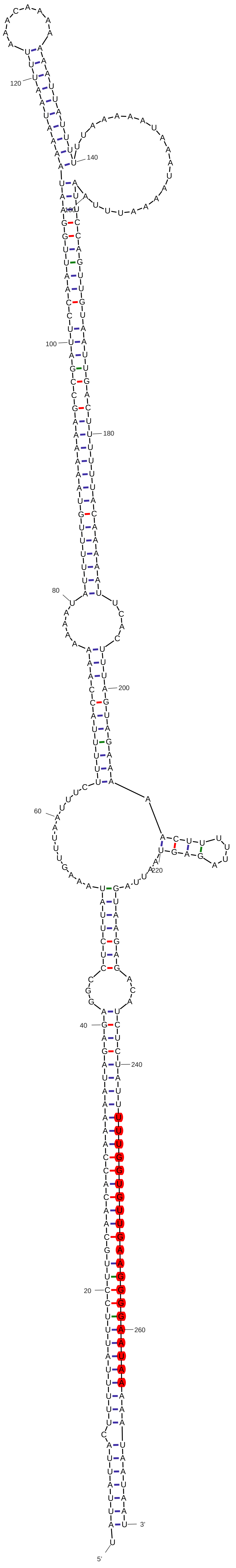

dG = -86.93 [Initially -90.10] novel_mir_2543_1

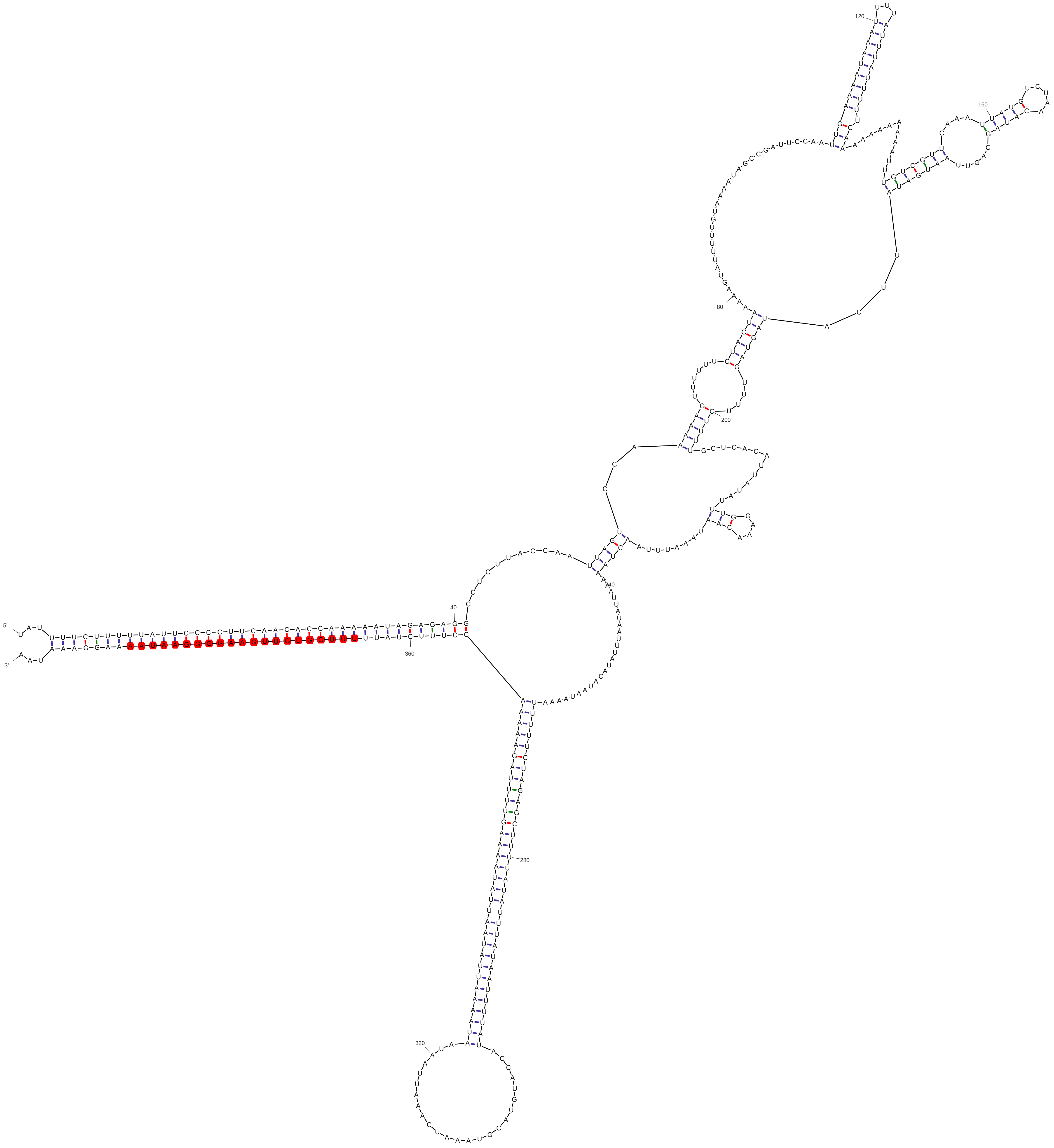

dG = -97.07 [Initially -109.20] novel_mir_2543_2

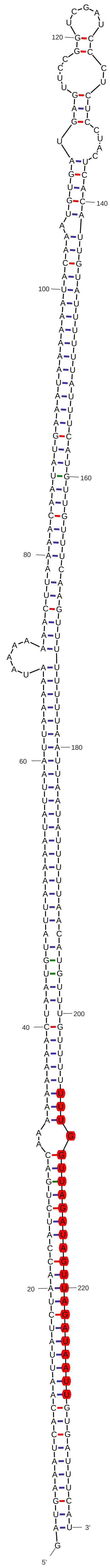

dG = -100.50 [Initially -100.50] novel_mir_4261

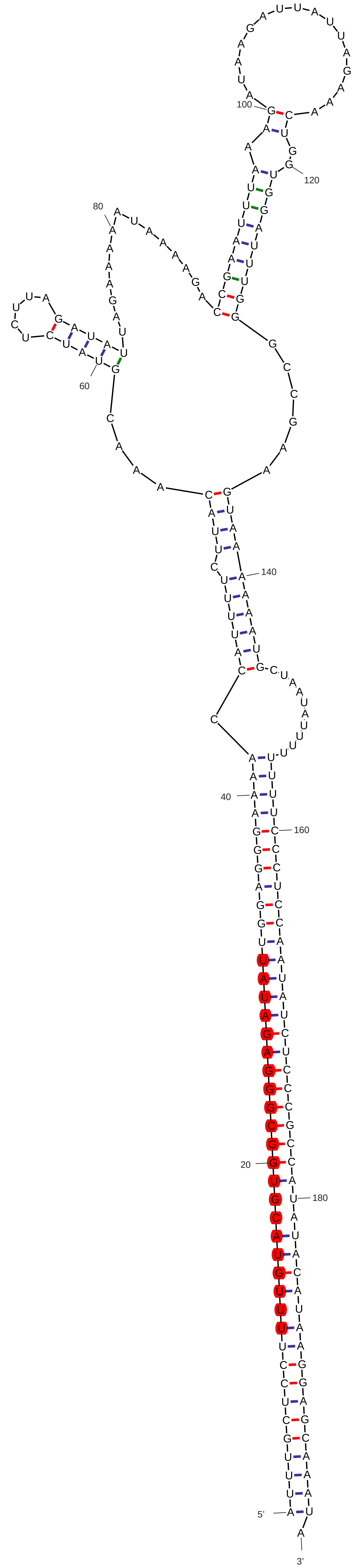

dG = -77.03 [Initially -81.60] novel_mir_5769

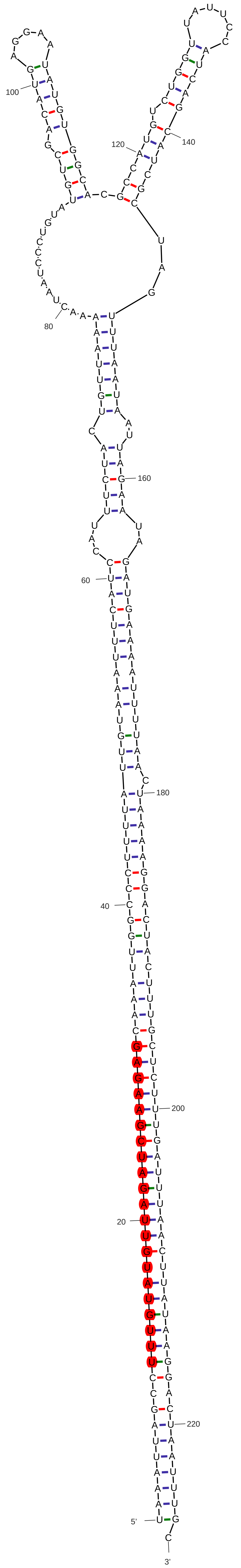

dG = -70.89 [Initially -74.60] novel_mir_4990

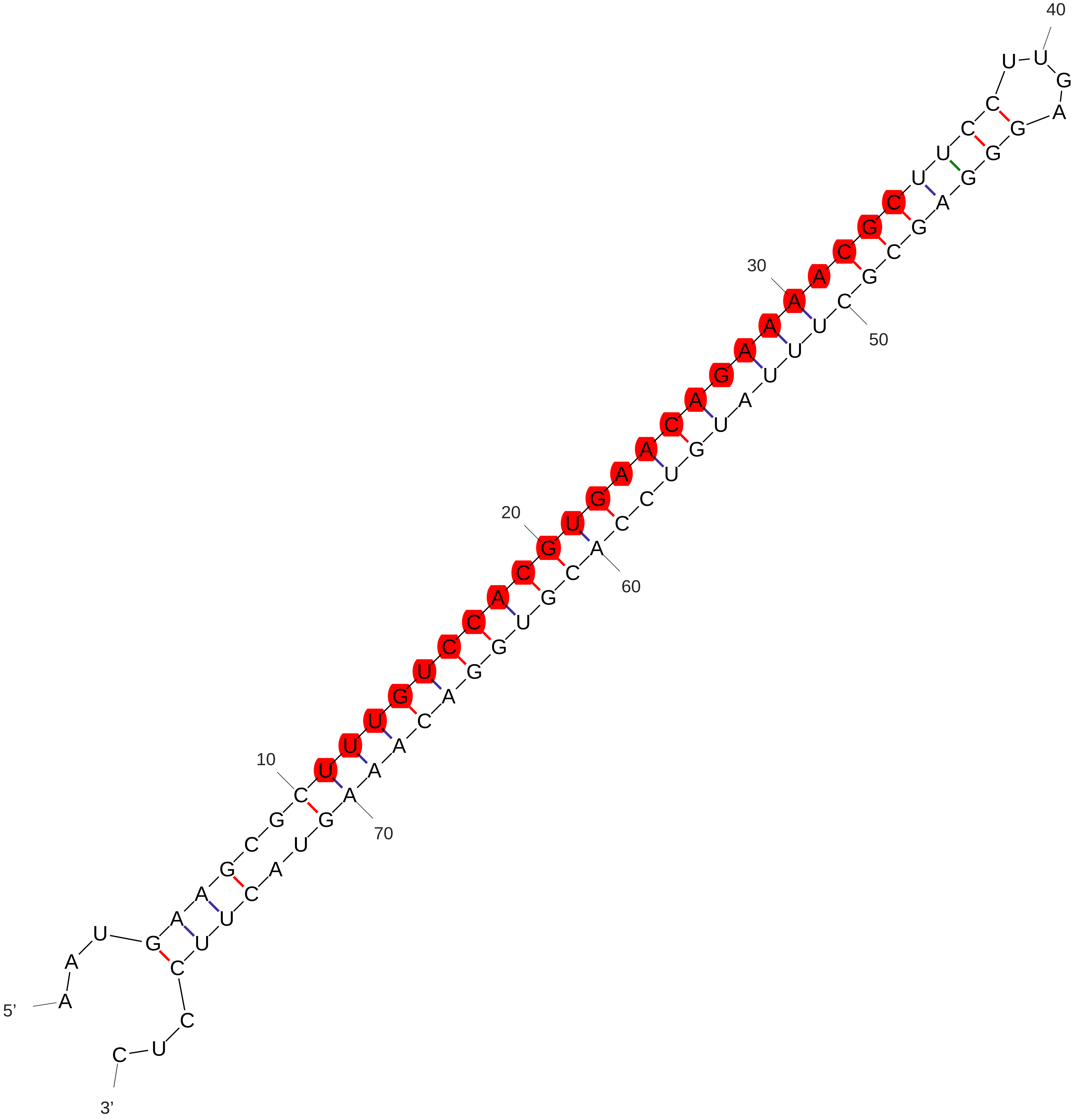

dG = -45.30 [Initially -45.30] novel_mir_2588

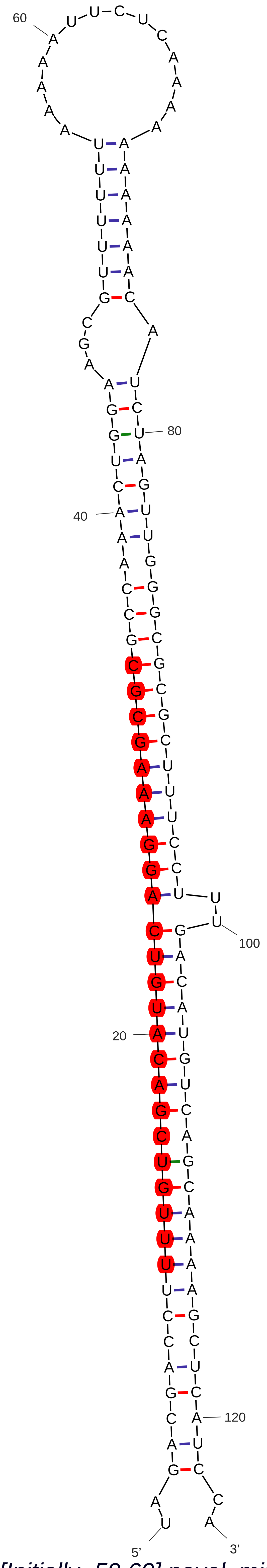

dG = -59.60 [Initially -59.60] novel_mir_5867

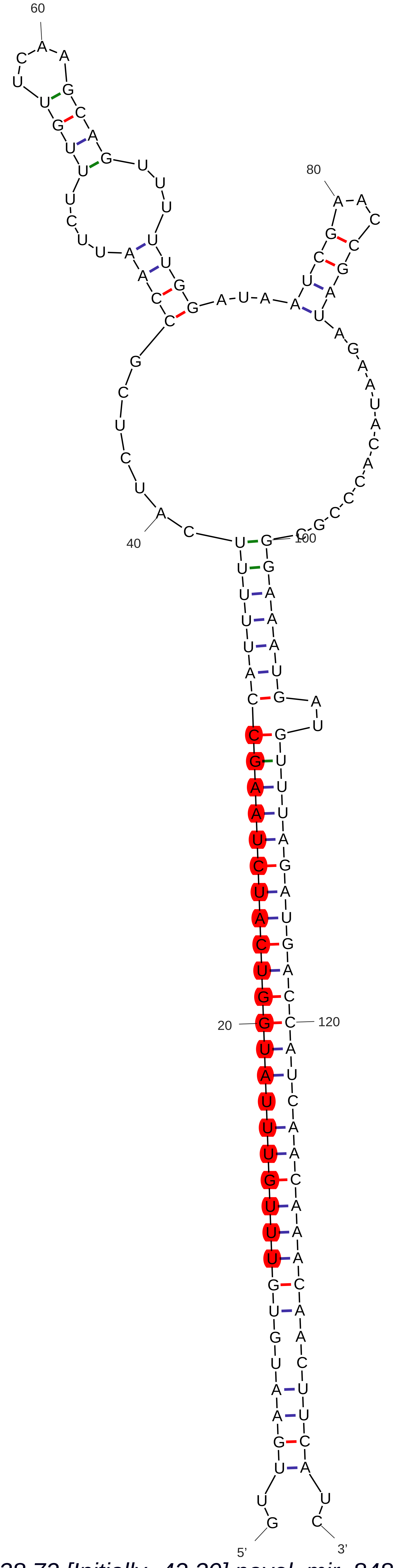

dG = -38.72 [Initially -42.30] novel_mir_848

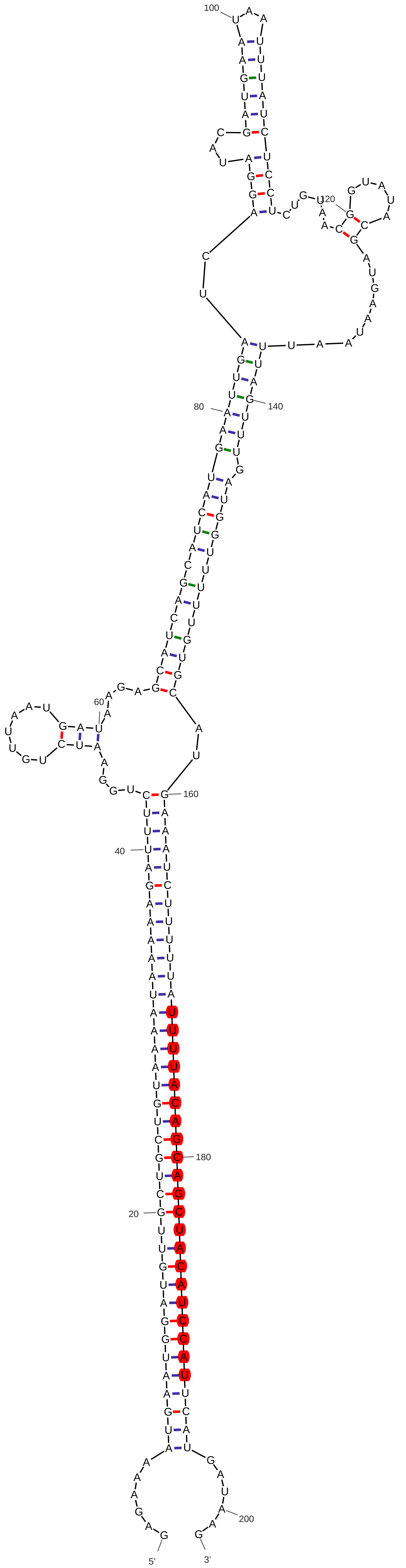

dG = -63.59 [Initially -70.90] novel_mir_4061

Output of sir_graph (©)
mfold_util 4.6

Created Sun Jan 13 23:16:51 2013

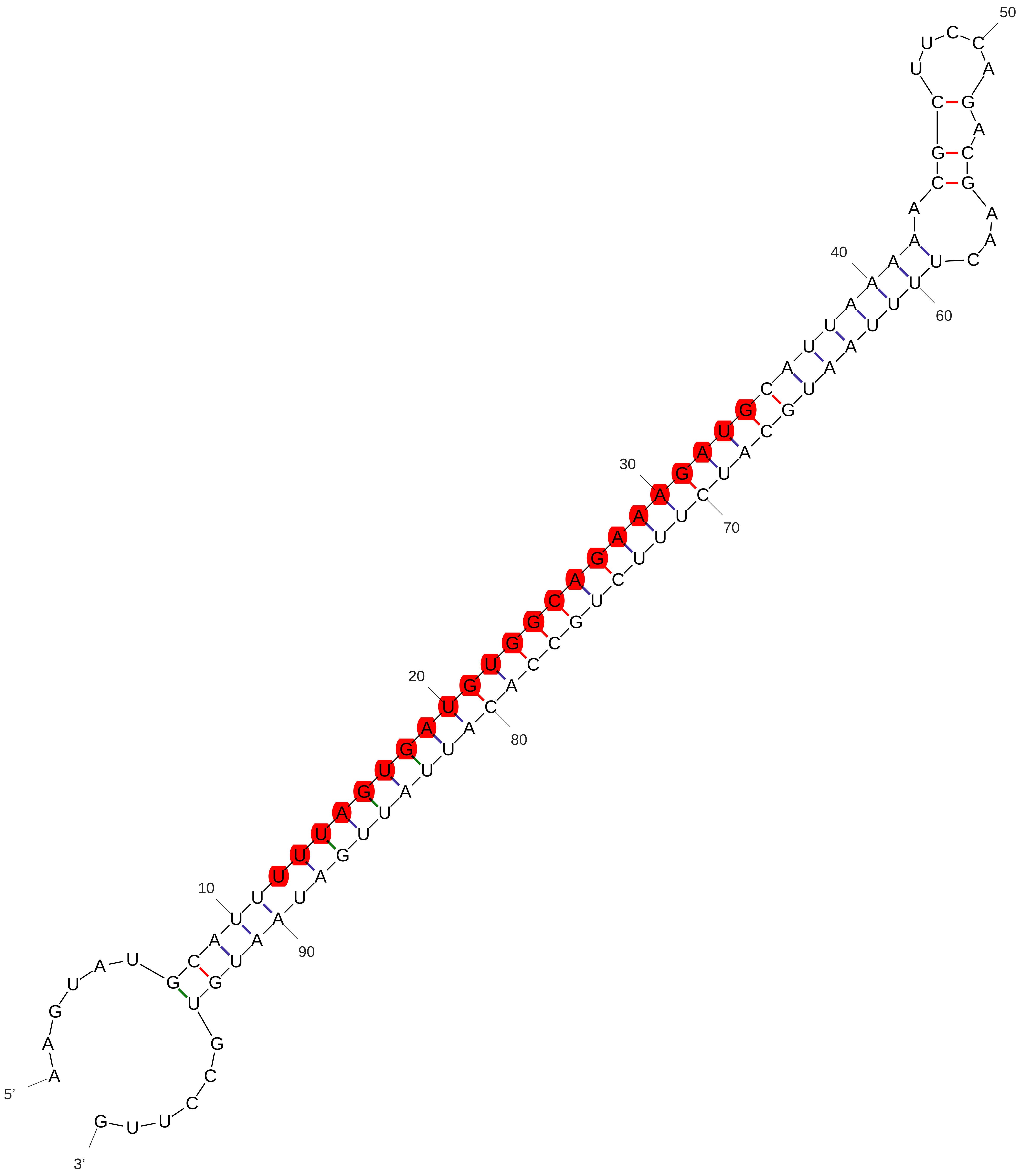

dG = -48.60 [Initially -48.60] novel_mir_4987_1

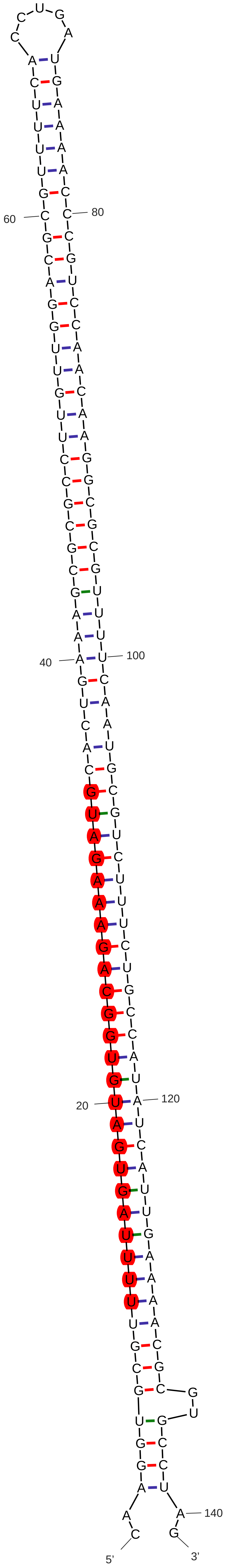

dG = -104.70 [Initially -104.70] novel_mir_4987_2

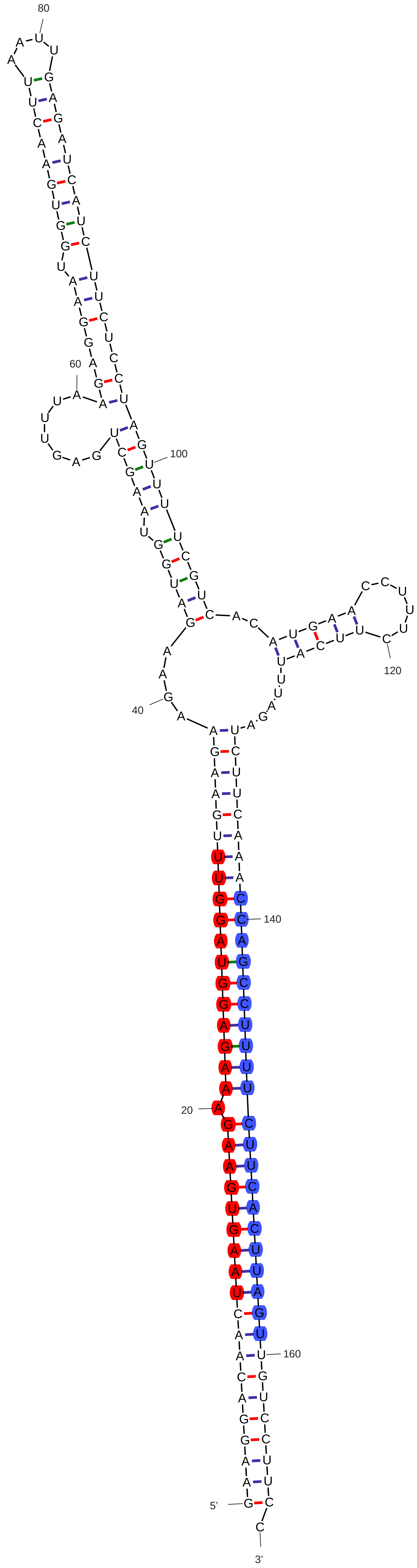

dG = -71.23 [Initially -74.50] GhmiRnC_novel_mir_5

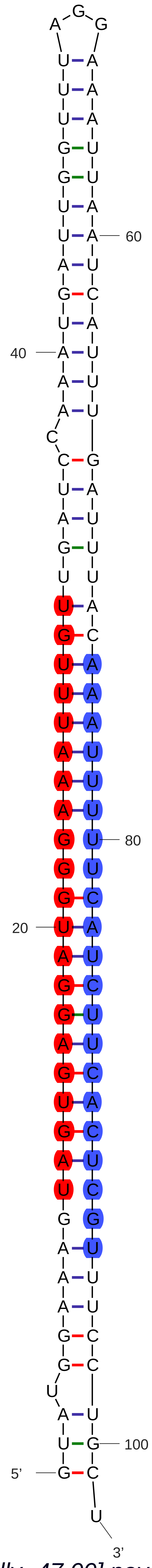

dG = -47.00 [Initially -47.00] novel_mir_1227

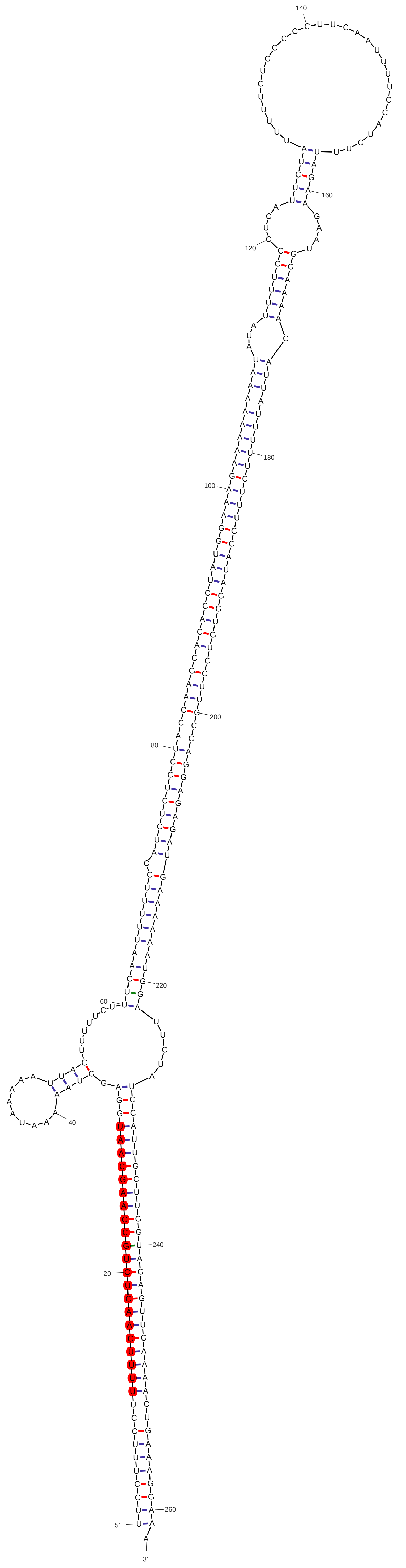

dG = -110.65 [Initially -113.70] novel_mir_4246

Output of sir_graph (©)
mfold_util 4.6

Created Sun Jan 13 23:16:56 2013

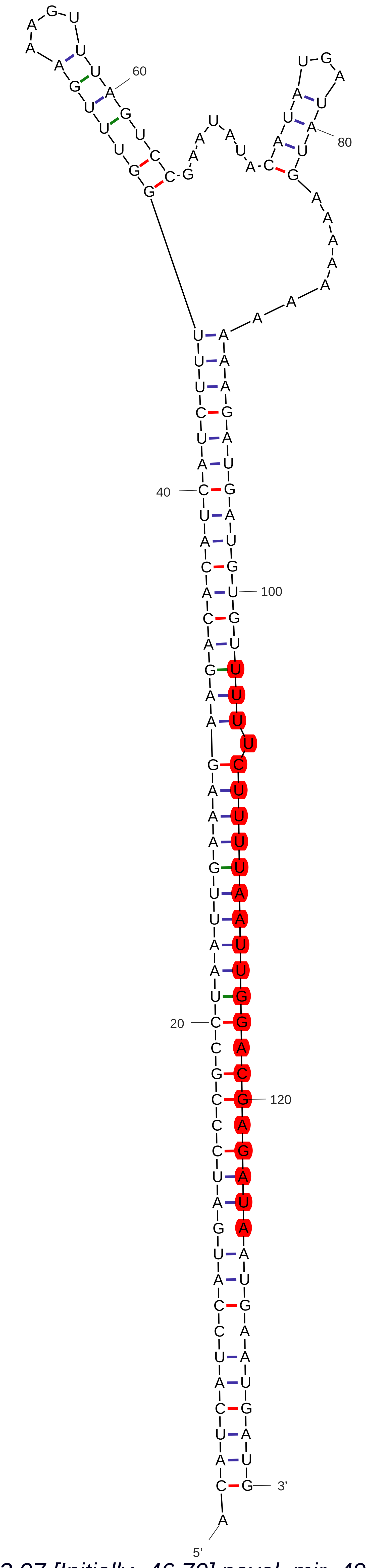

dG = -43.07 [Initially -46.70] novel_mir_4988

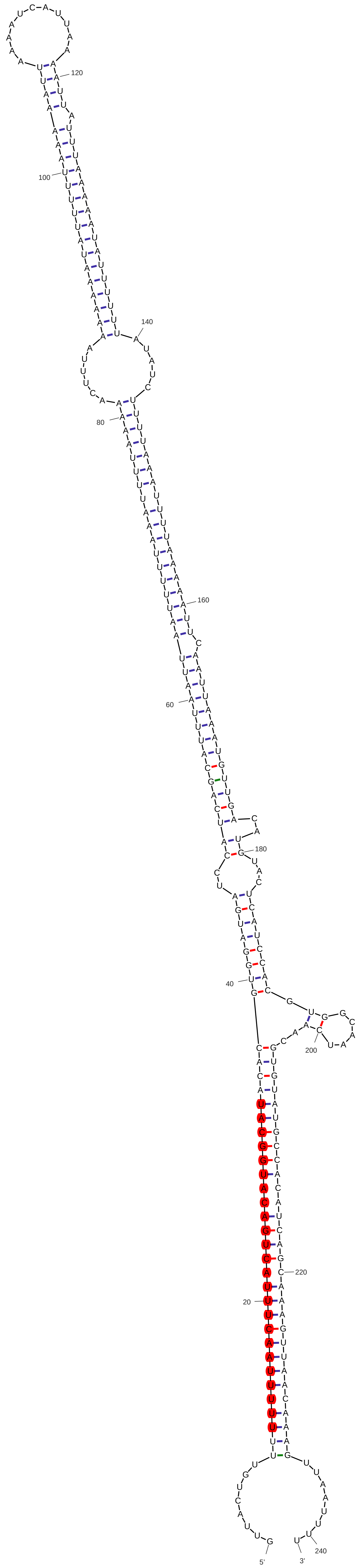

dG = -68.30 [Initially -71.20] novel_mir_291

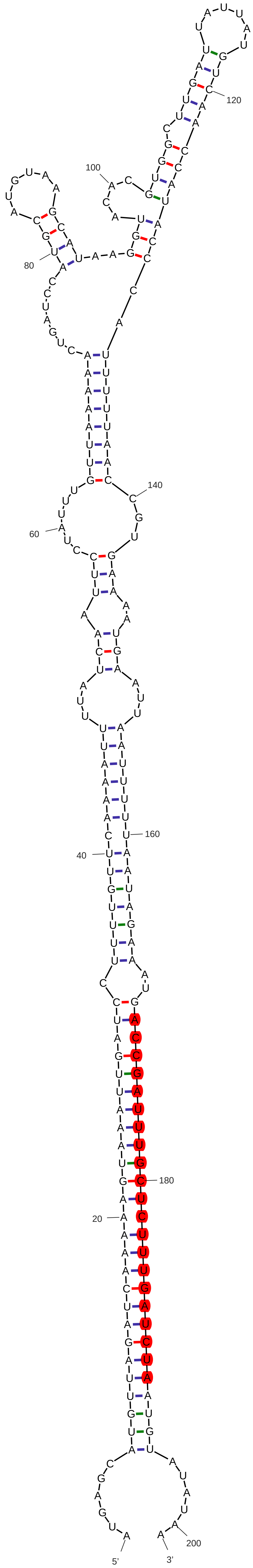

dG = -44.13 [Initially -47.50] novel_mir_5016

Output of sir_graph (©)
mfold_util 4.6

Created Sun Jan 13 07:24:16 2013

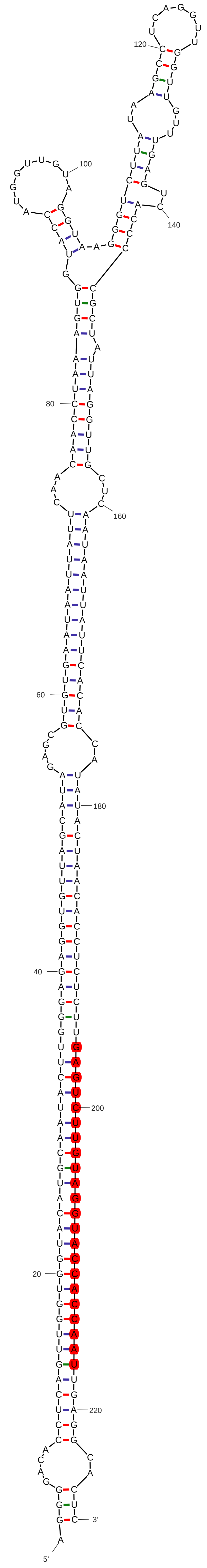

dG = -109.30 [Initially -111.30] novel_mir_5235

Output of sir_graph (©)
mfold_util 4.6

Created Sun Jan 13 07:24:24 2013

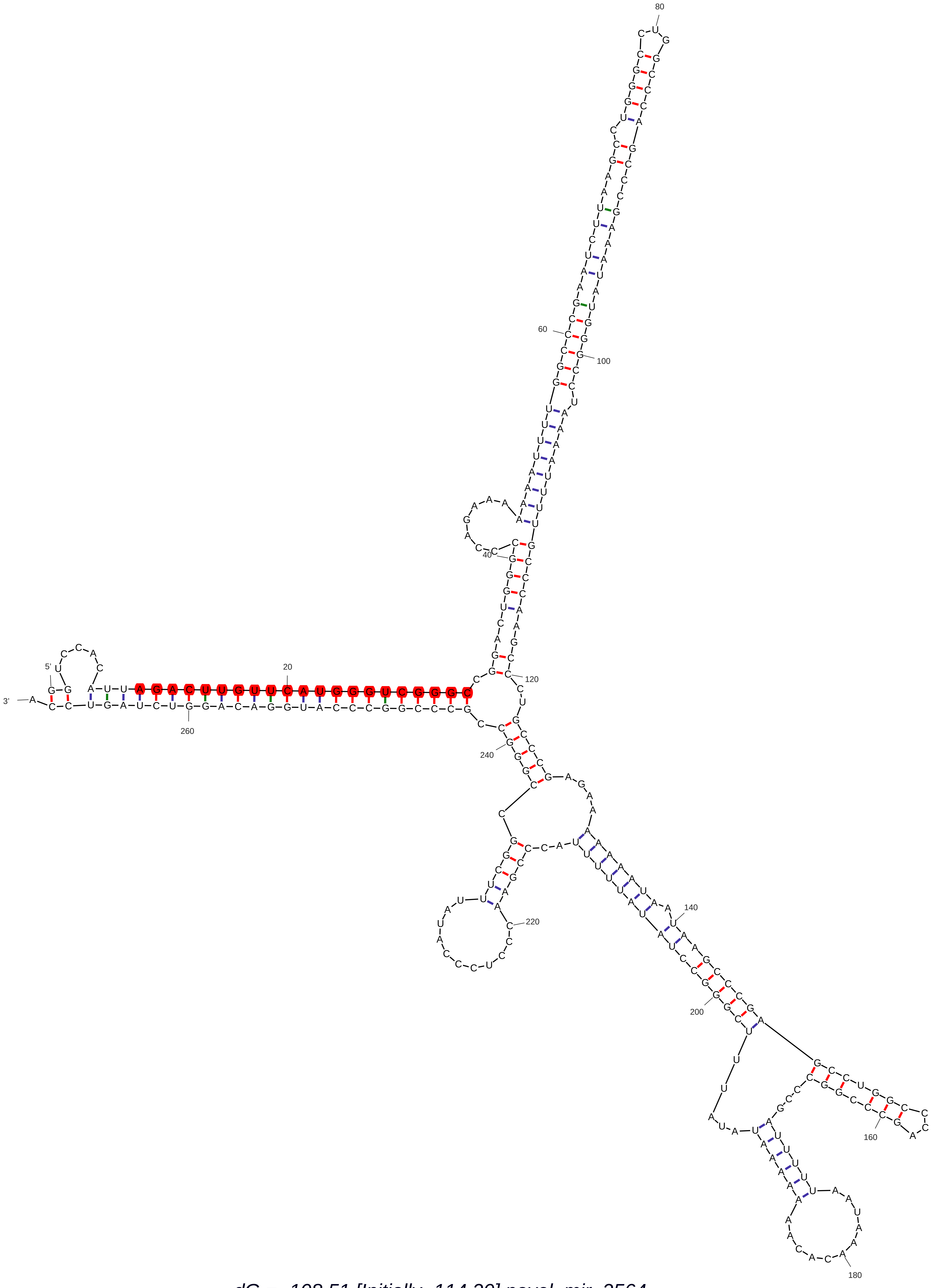

dG = -108.51 [Initially -114.20] novel_mir_2564

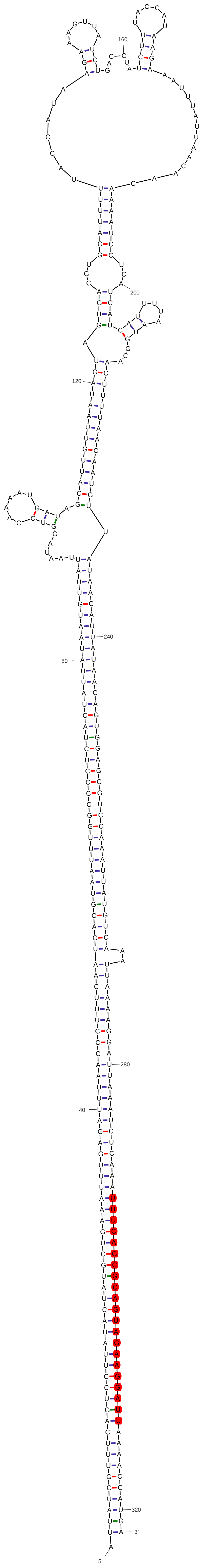

dG = -113.53 [Initially -122.00] novel_mir_5669

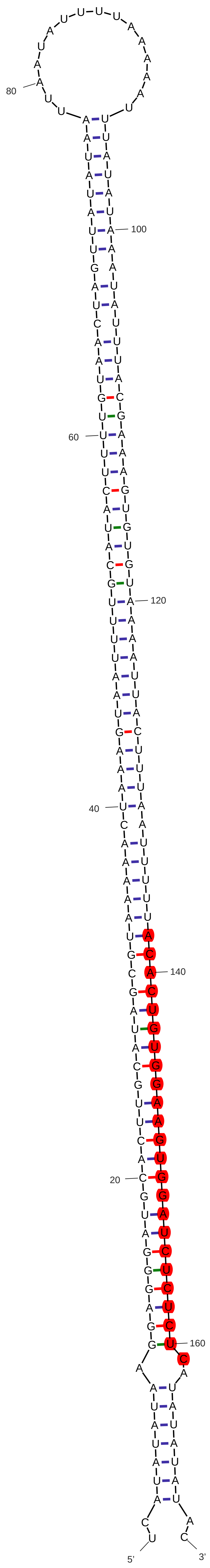

dG = -74.00 [Initially -74.00] novel_mir_4991

Output of sir_graph (©)
mfold_util 4.6

Created Sun Jan 13 07:29:30 2013

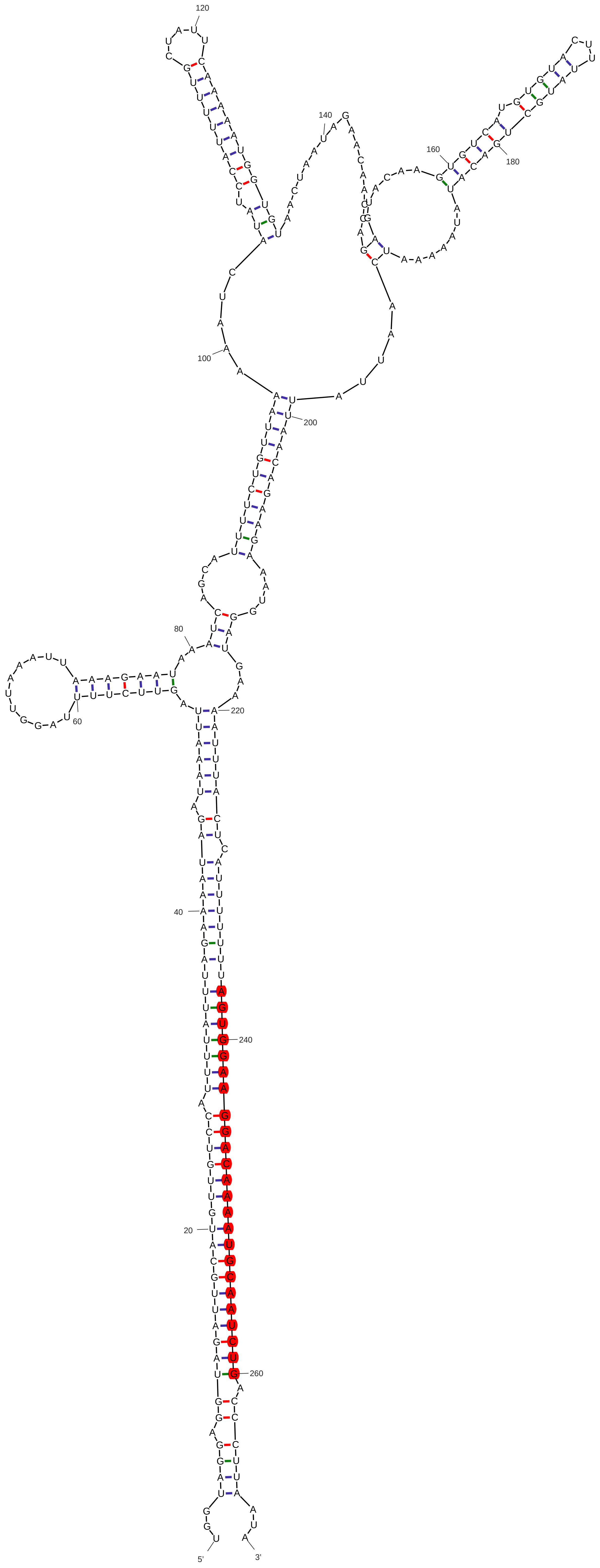

dG = -64.95 [Initially -70.40] novel_mir_819

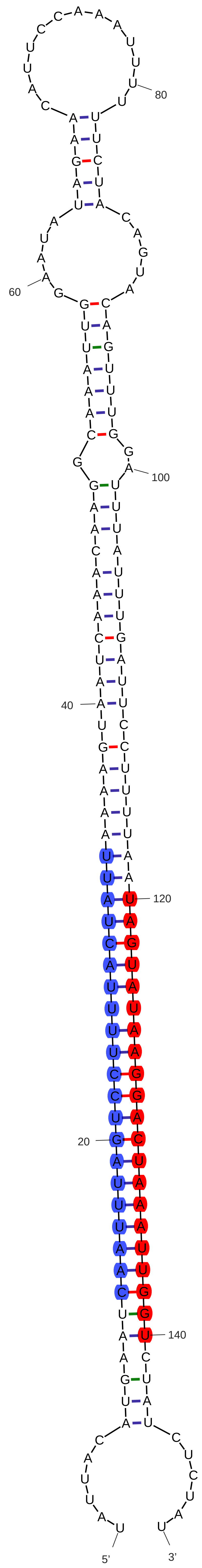

dG = -48.30 [Initially -48.30] novel_mir_4084

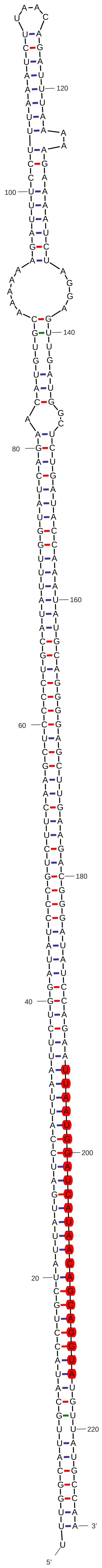

dG = -155.50 [Initially -155.50] novel_mir_76

Output of sir_graph (©)
mfold_util 4.6

Created Sun Jan 13 07:29:47 2013

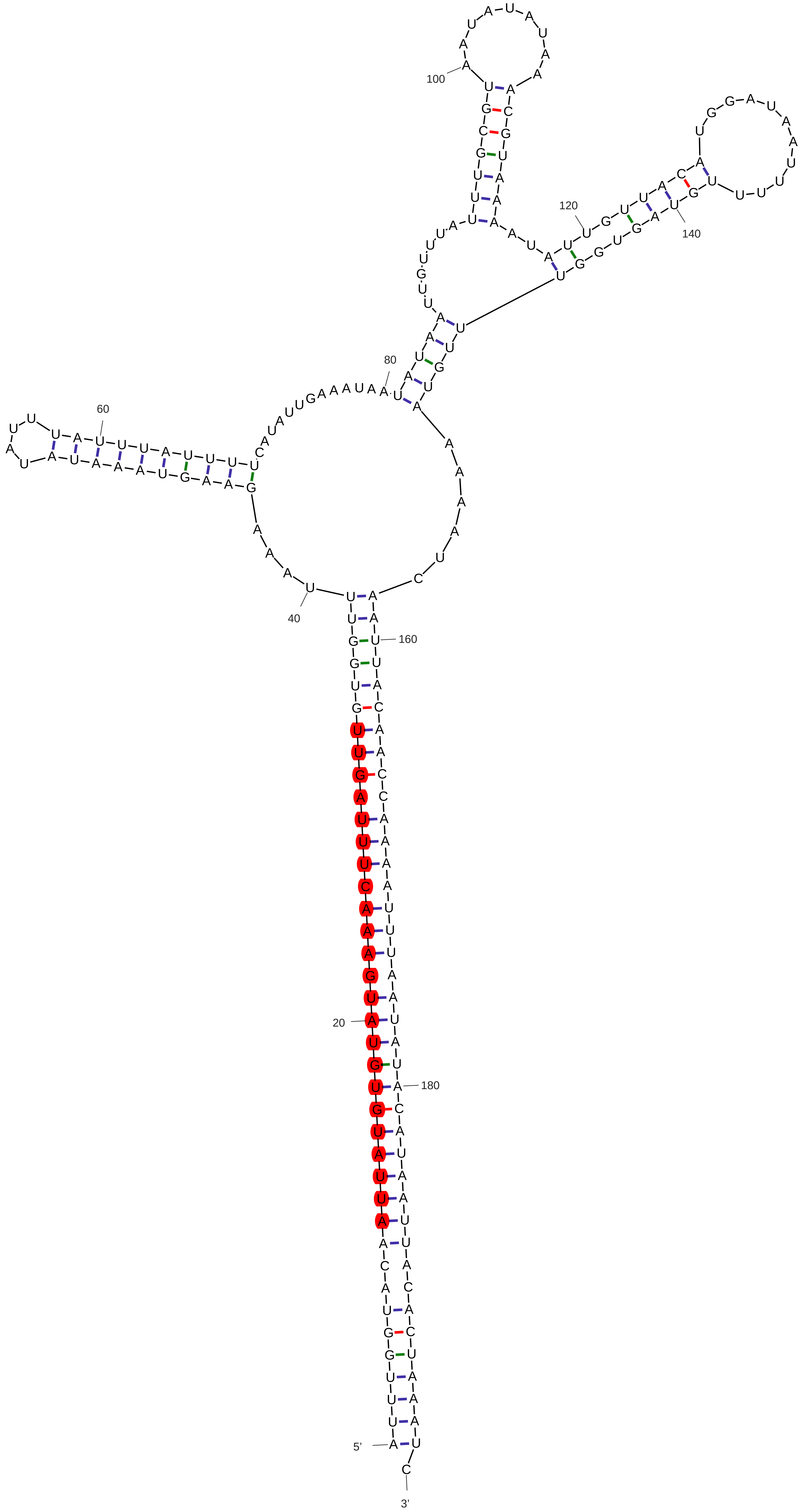

dG = -33.17 [Initially -39.70] novel_mir_5019

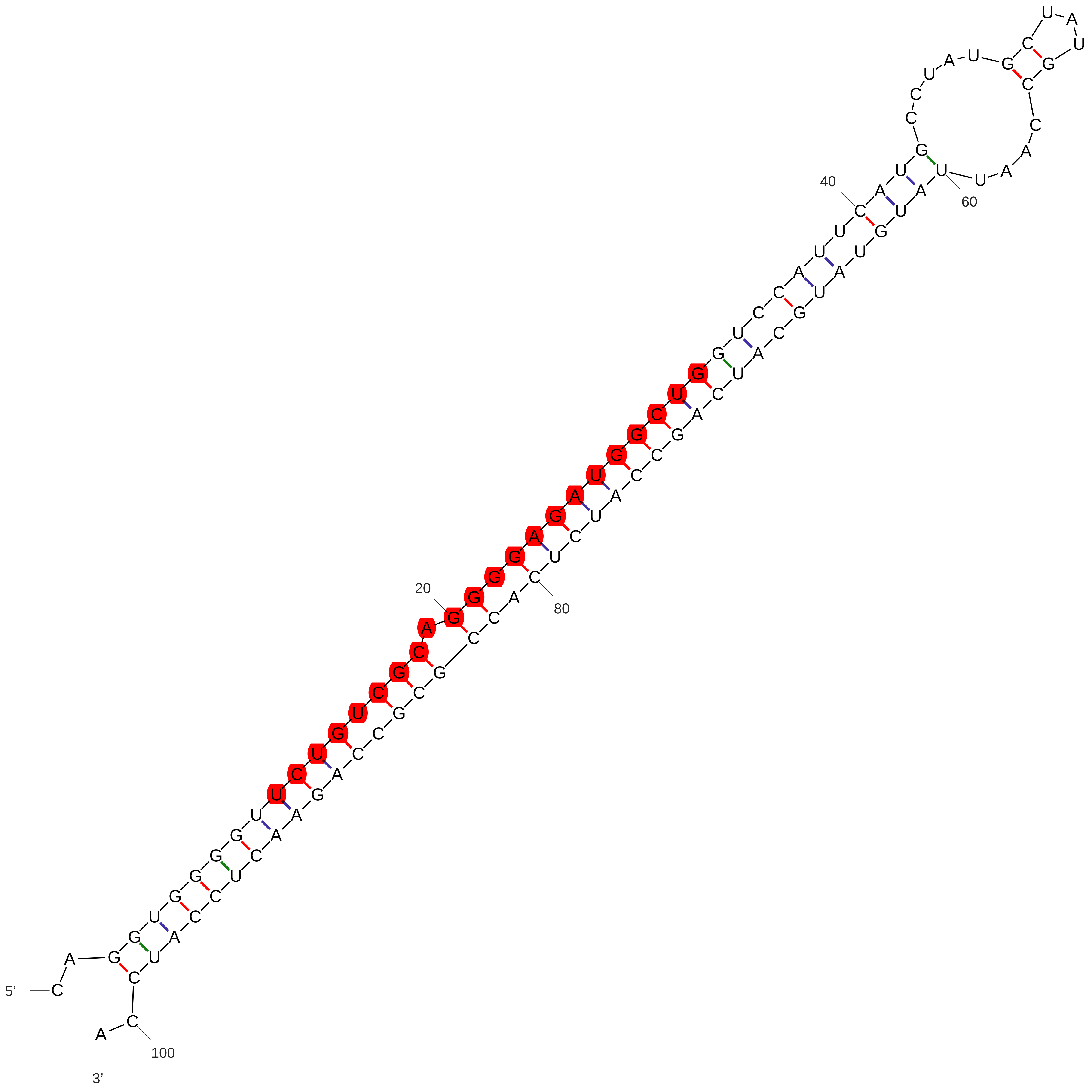

dG = -52.70 [Initially -52.70] GhmiRnD

Output of sir_graph (©)
mfold_util 4.6

Created Wed Jan 16 02:45:12 2013

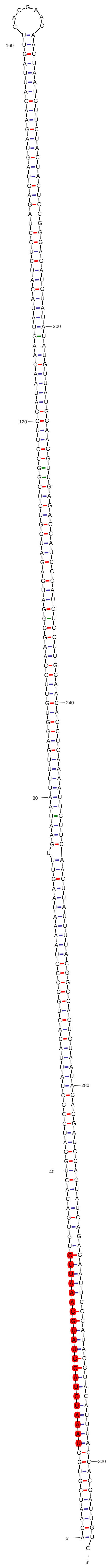

dG = -251.50 [Initially -251.50] novel_mir_4020

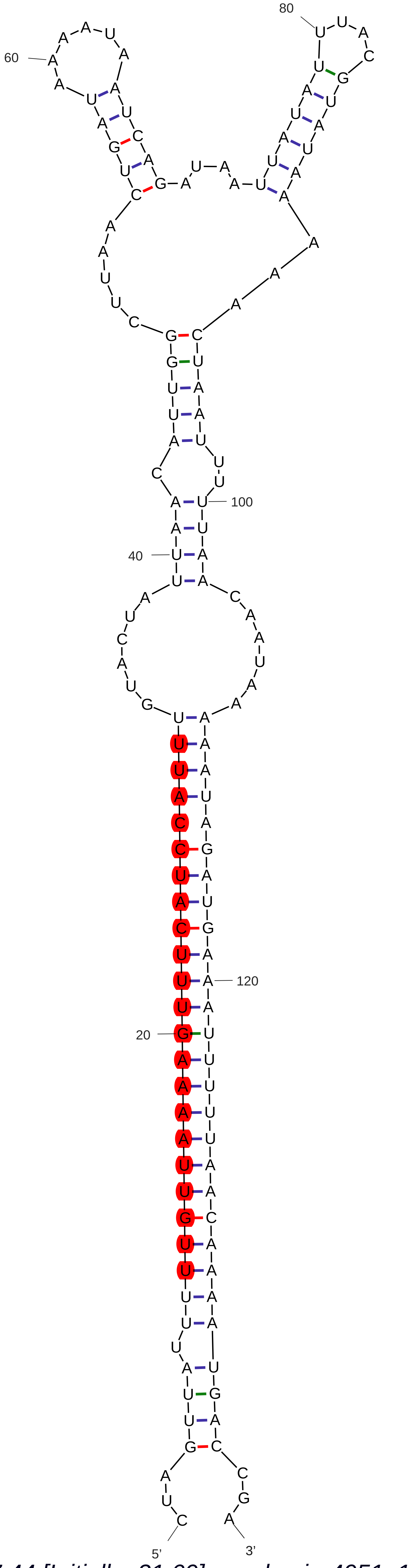

dG = -27.44 [Initially -31.60] novel_mir_4051_1

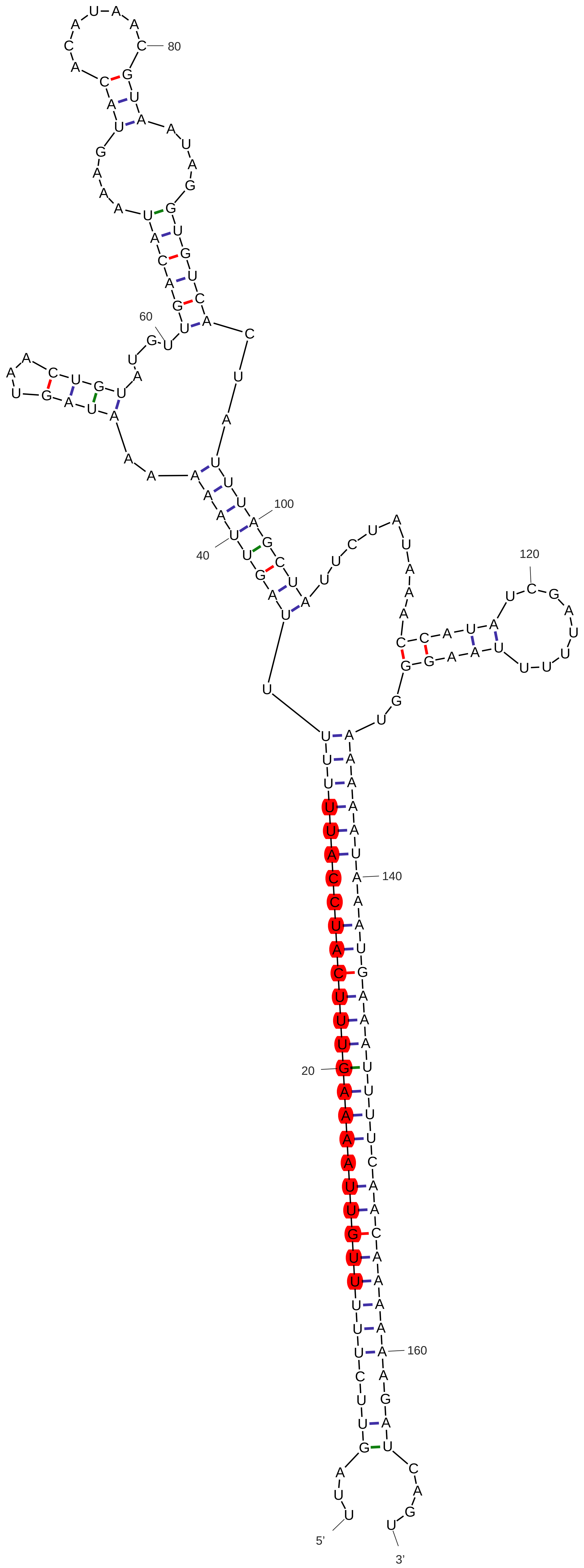

dG = -25.29 [Initially -31.30] novel_mir_4051_2

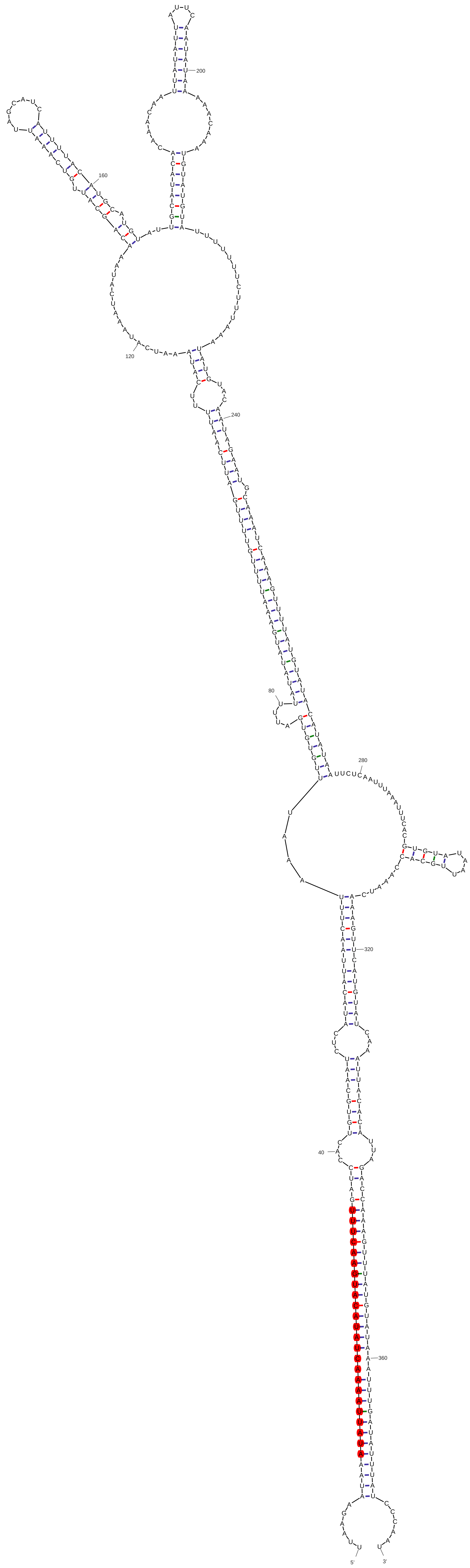

dG = -78.26 [Initially -86.90] novel_mir_4352

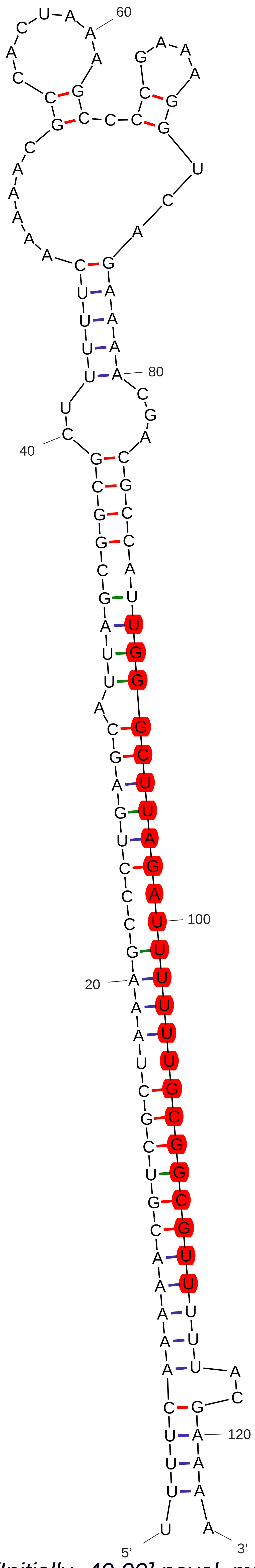

dG = -36.84 [Initially -40.00] novel_mir_2741

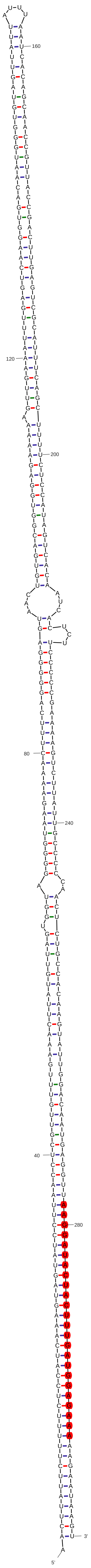

dG = -164.90 [Initially -164.90] novel_mir_887_1

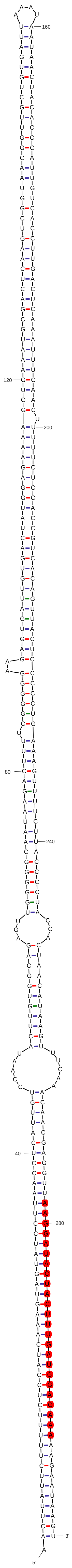

dG = -158.00 [Initially -158.00] novel_mir_887_2

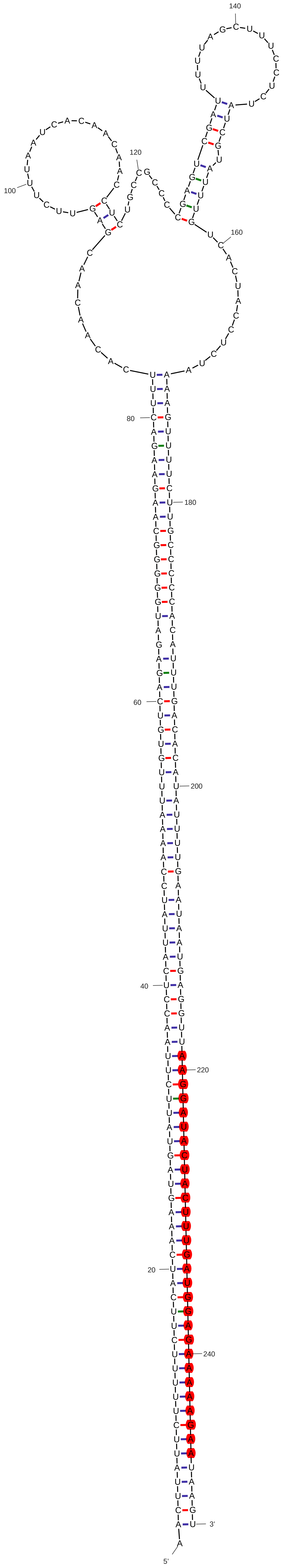

dG = -117.57 [Initially -121.30] novel_mir_887_3

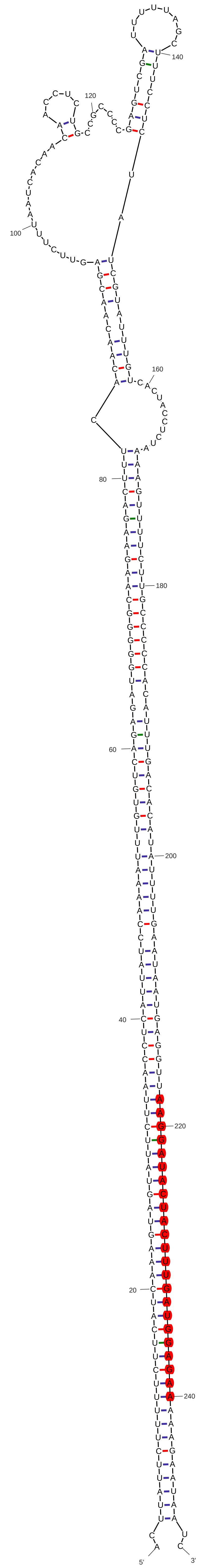

dG = -111.18 [Initially -115.20] novel_mir_887_4

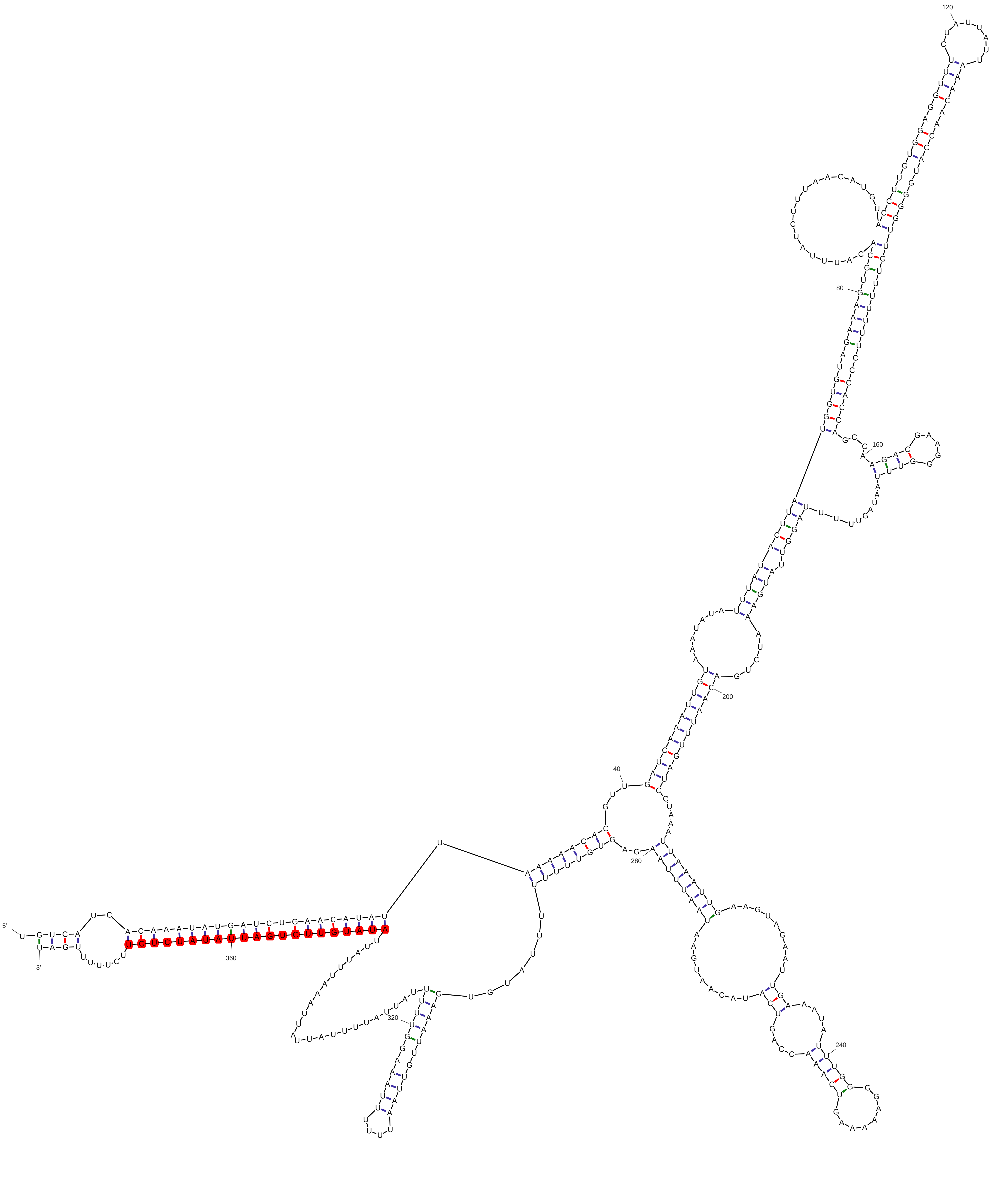

dG = -64.65 [Initially -73.90] novel_mir_2542

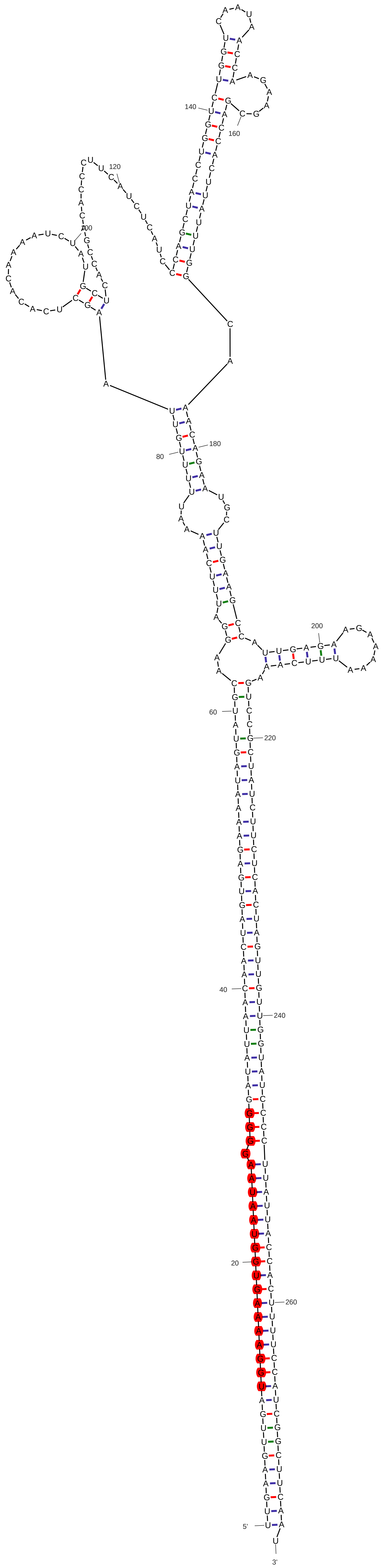

dG = -107.83 [Initially -114.10] novel_mir_4037

Output of sir_graph (©)
mfold_util 4.6

Created Sun Jan 13 07:33:55 2013

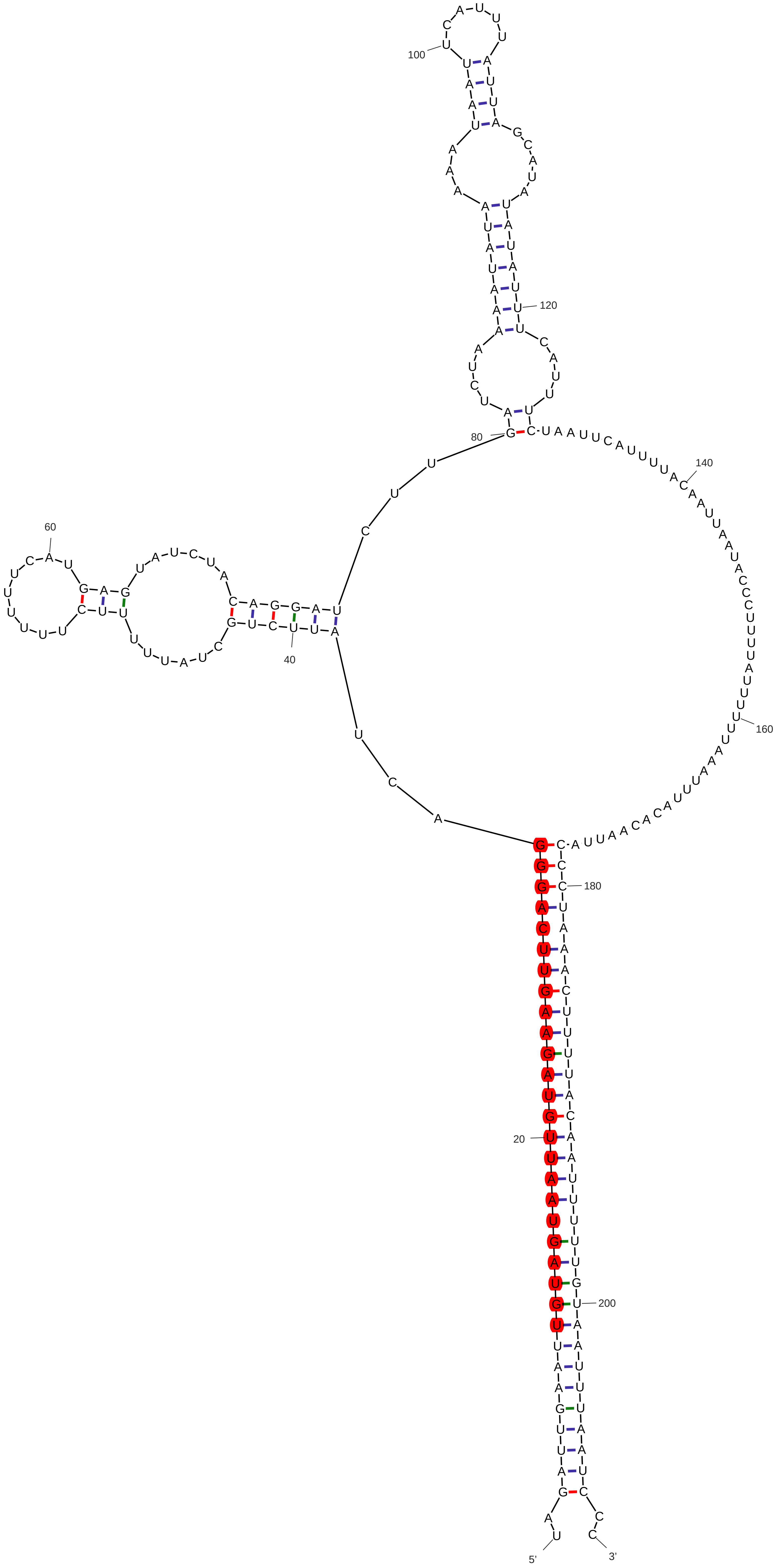

dG = -31.24 [Initially -36.80] novel_mir_2454_1

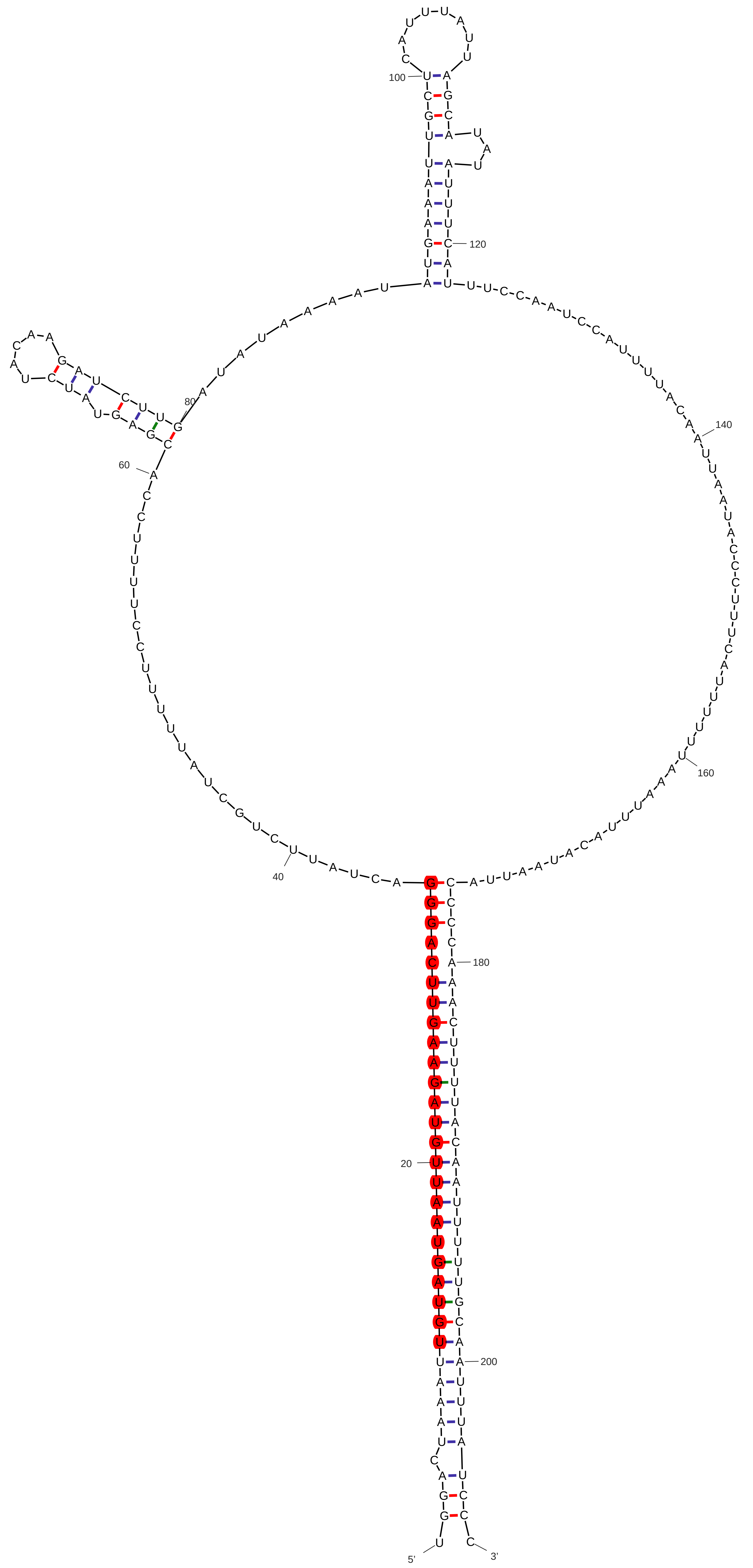

dG = -36.15 [Initially -42.70] novel_mir_2454_2

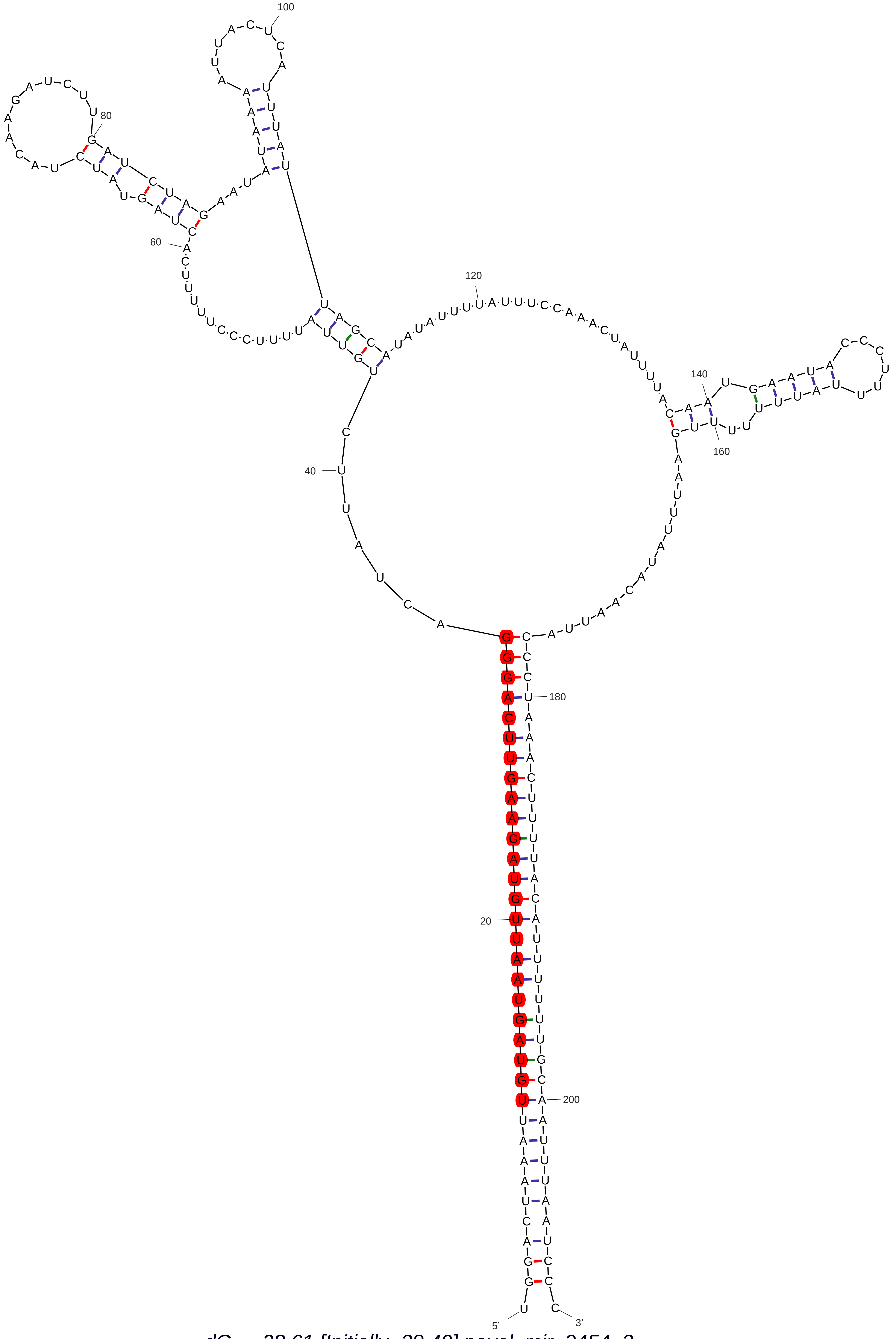

dG = -28.61 [Initially -38.40] novel_mir_2454_3

Created Sun Jan 13 07:37:16 2013

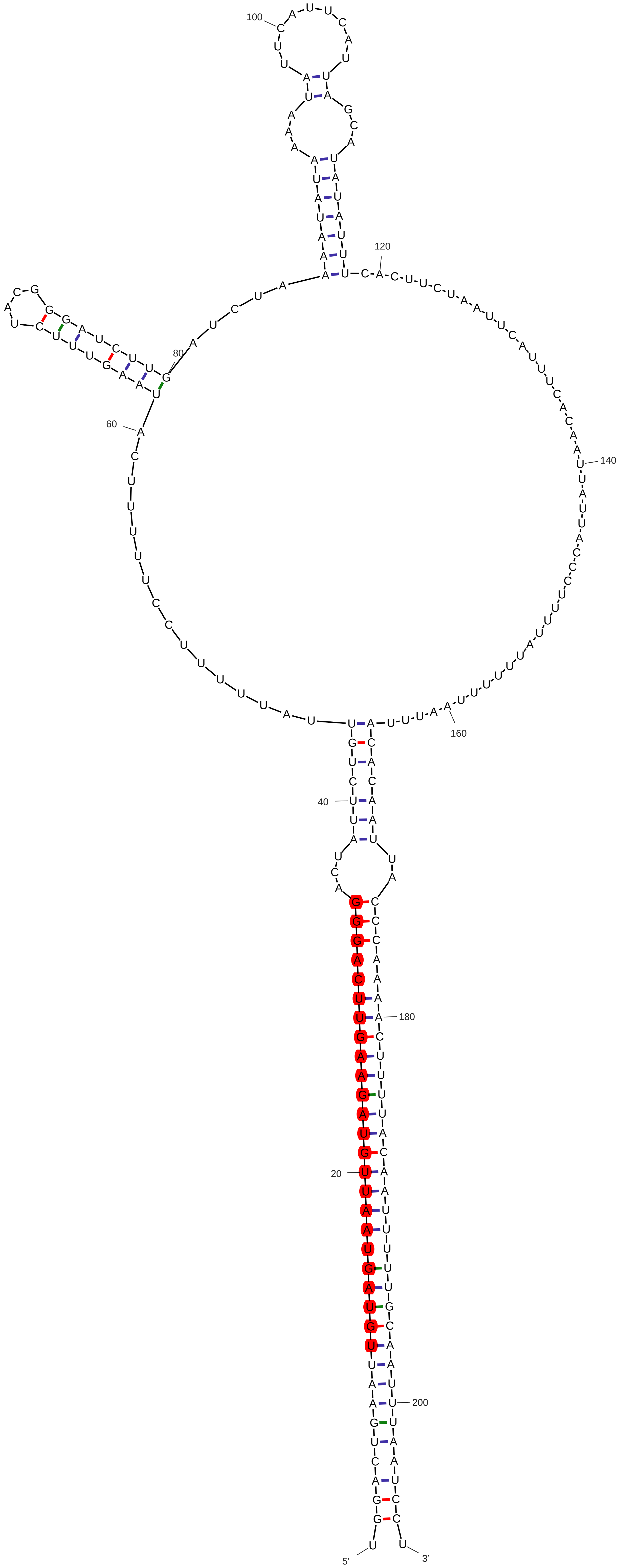

dG = -34.45 [Initially -39.20] novel_mir_2454_4

Output of sir_graph (©)
mfold_util 4.6

Created Sun Jan 13 07:37:22 2013

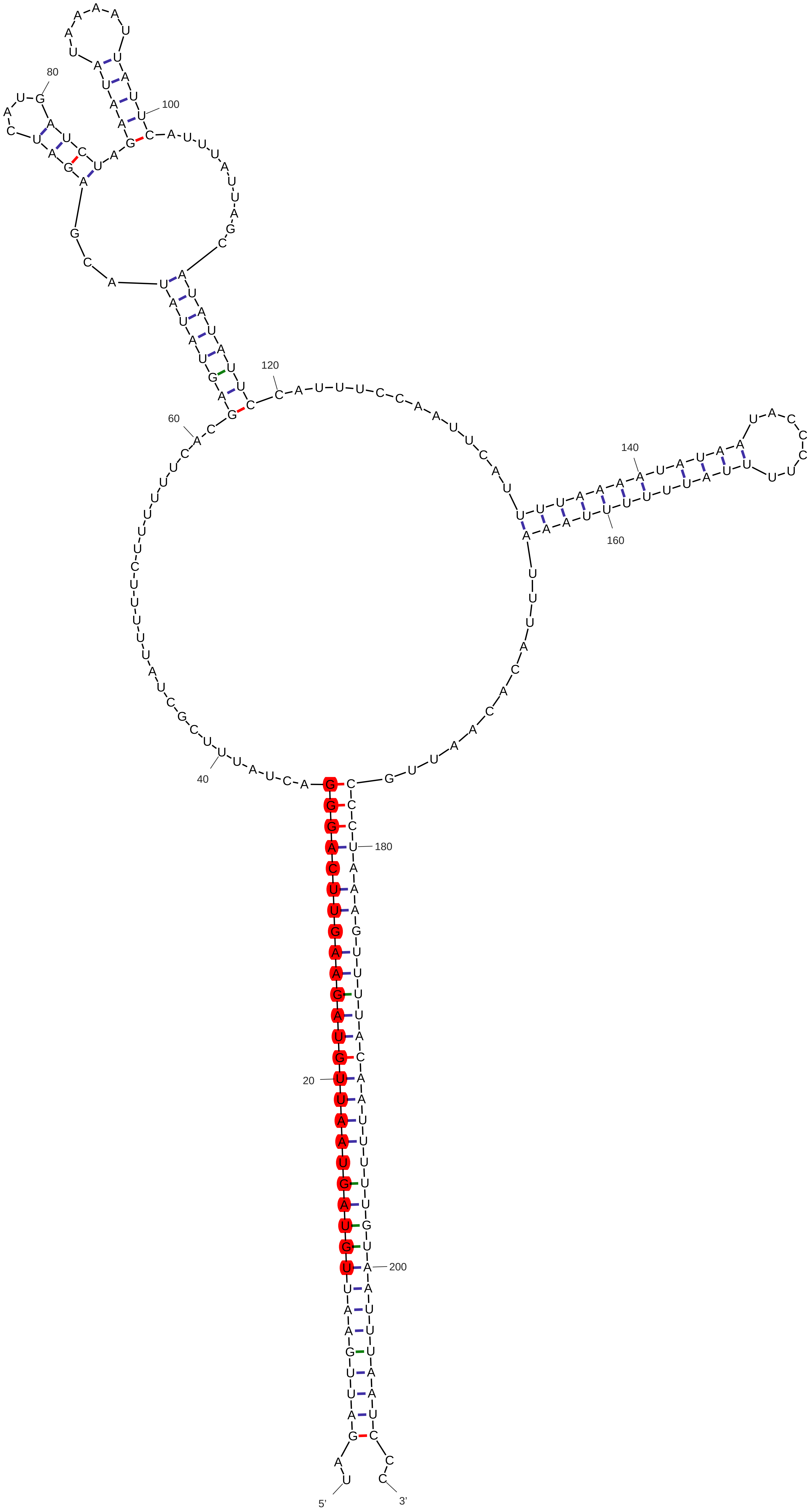

dG = -32.37 [Initially -40.70] novel_mir_2454_5

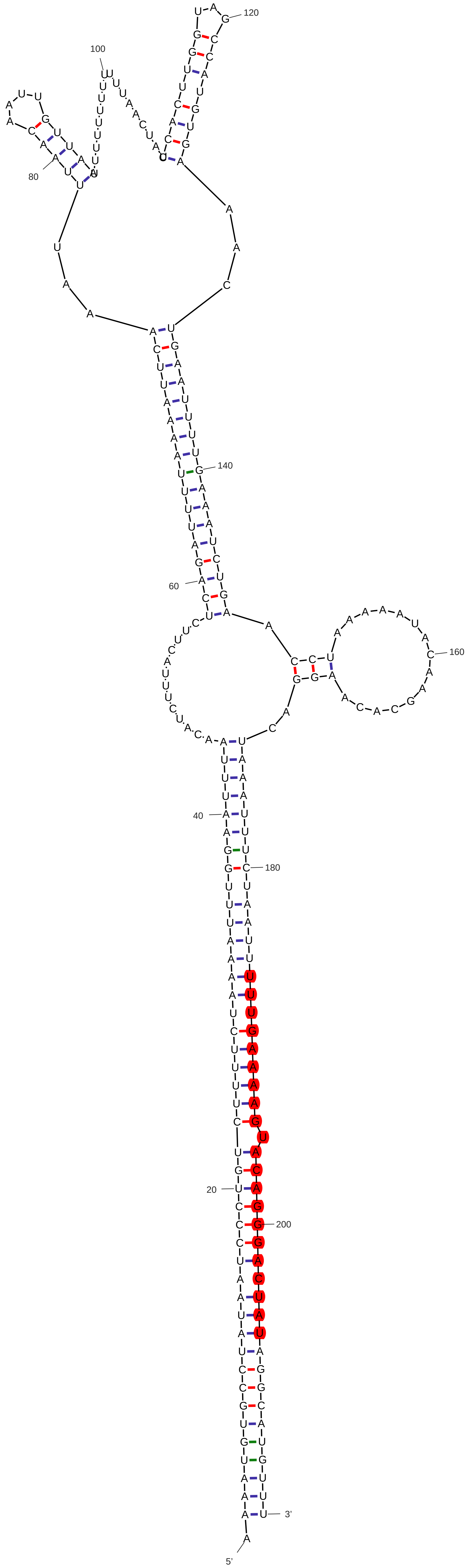

dG = -64.80 [Initially -71.60] novel_mir_3994

Output of sir_graph (©)
mfold_util 4.6

Created Sun Jan 13 07:37:31 2013

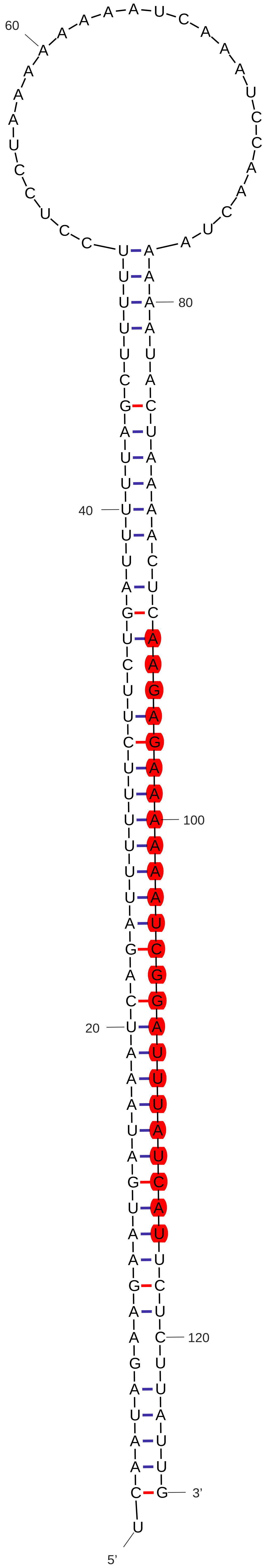

dG = -39.10 [Initially -39.10] novel_mir_5142

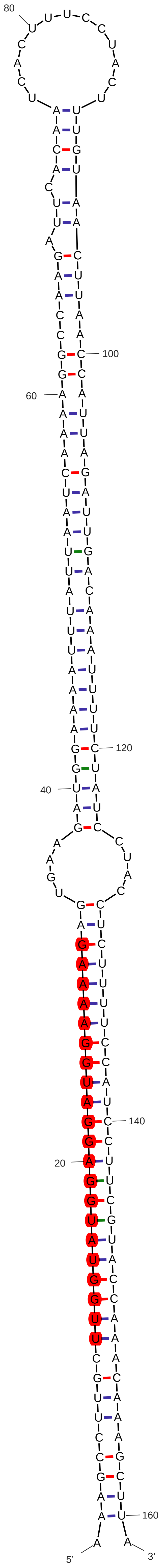

dG = -65.80 [Initially -65.80] GhmiRnE_novel_mir_868

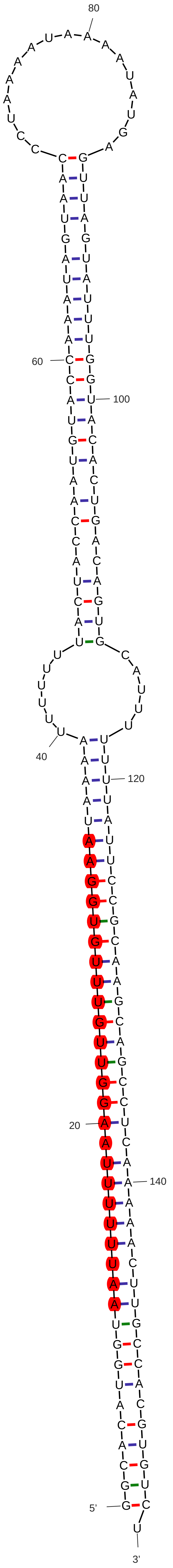

dG = -66.90 [Initially -66.90] novel_mir_2531_1

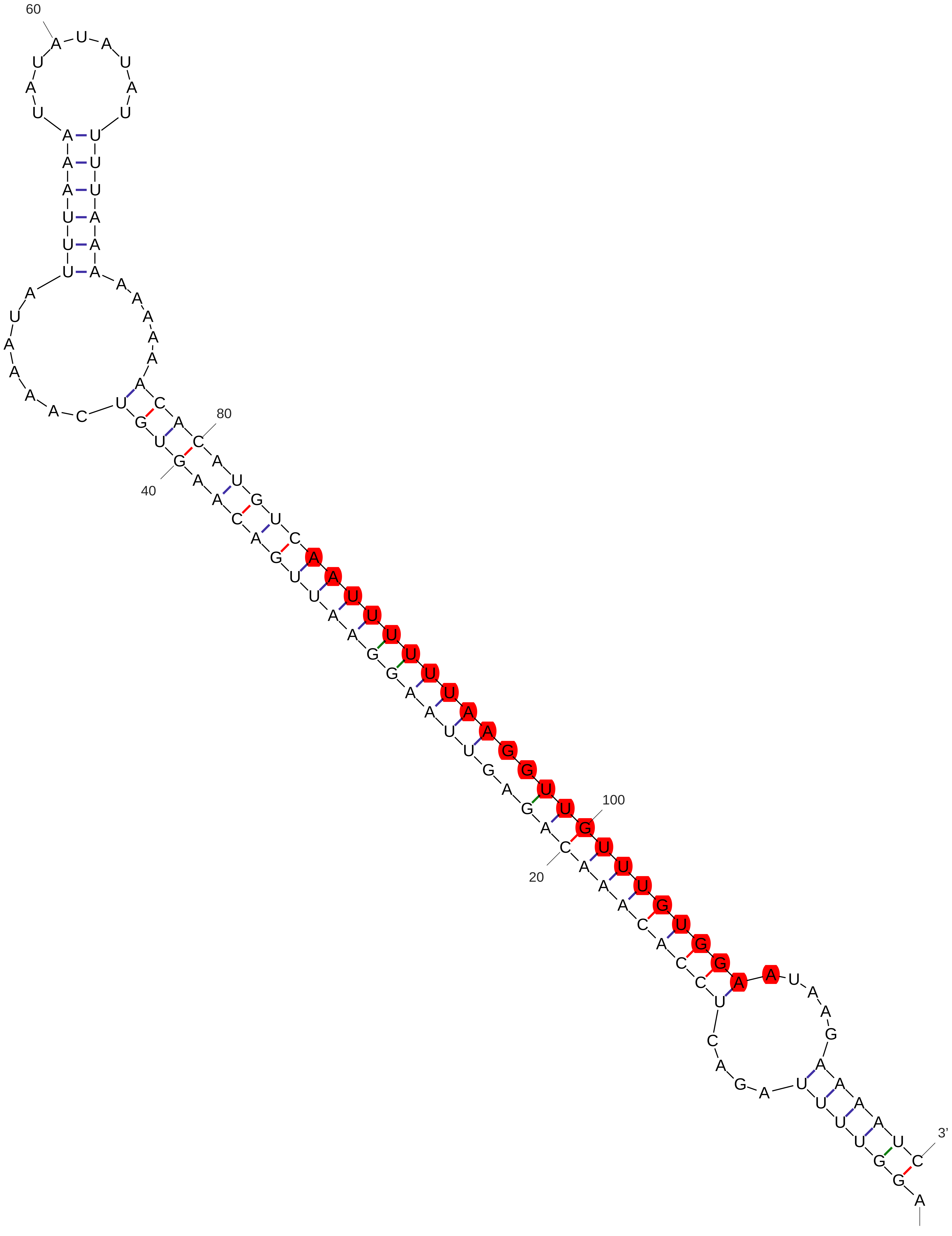

dG = -37.70 [Initially -37.70] novel_mir_2531_2

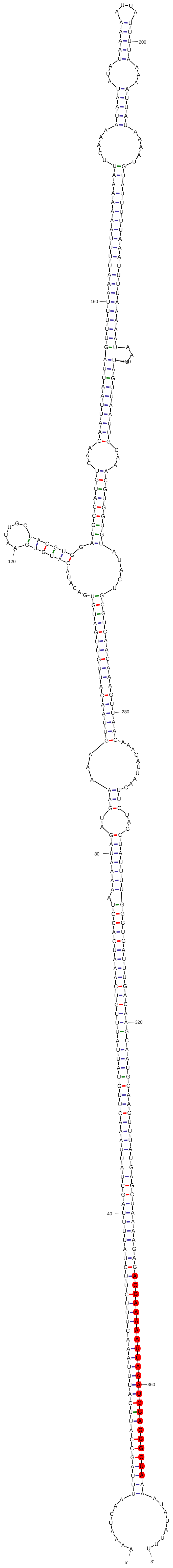

dG = -122.04 [Initially -124.80] novel_mir_2902

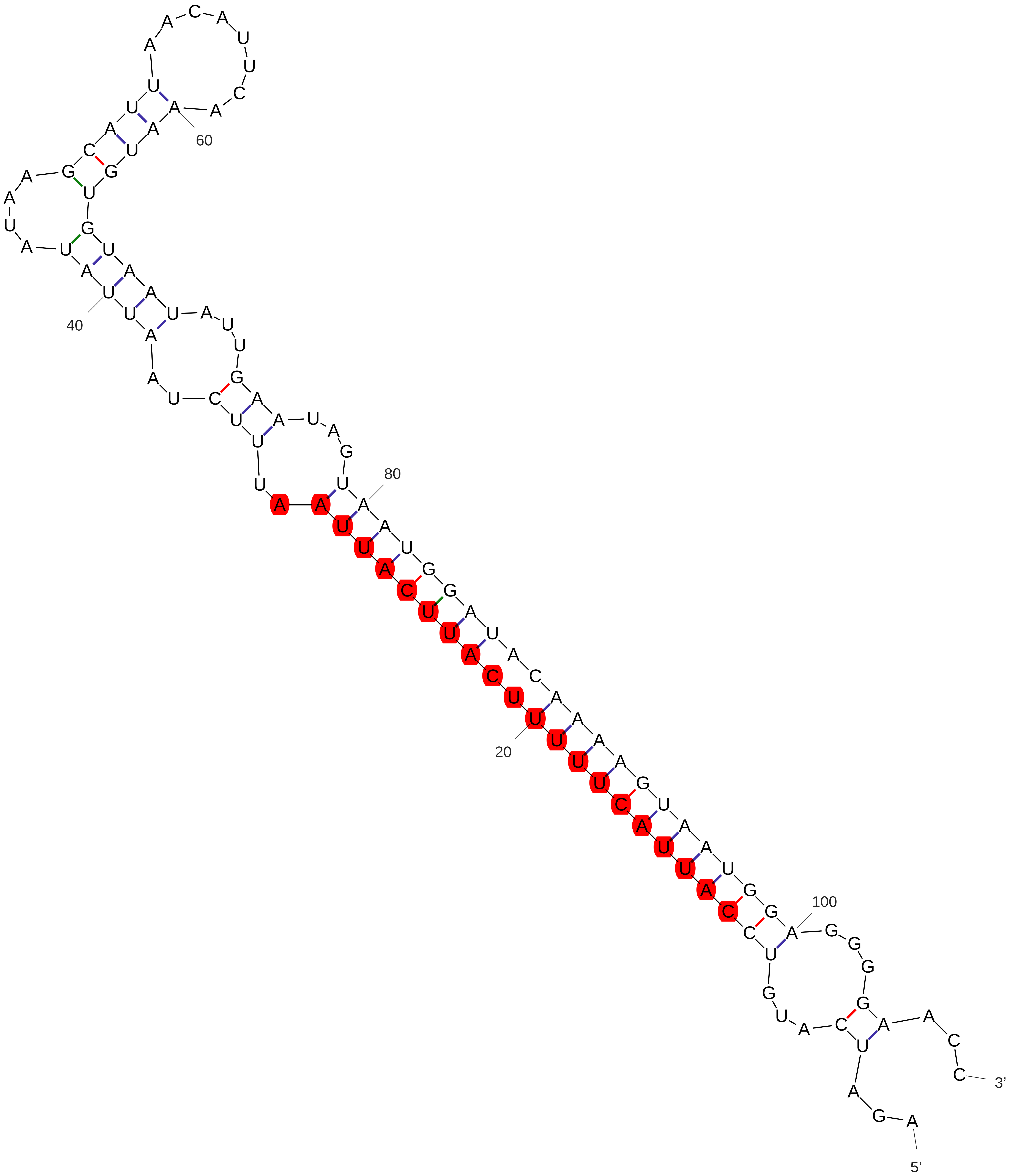

dG = -26.70 [Initially -26.70] novel_mir_4268

Output of sir_graph (©)
mfold_util 4.6

Created Sun Jan 13 07:38:04 2013

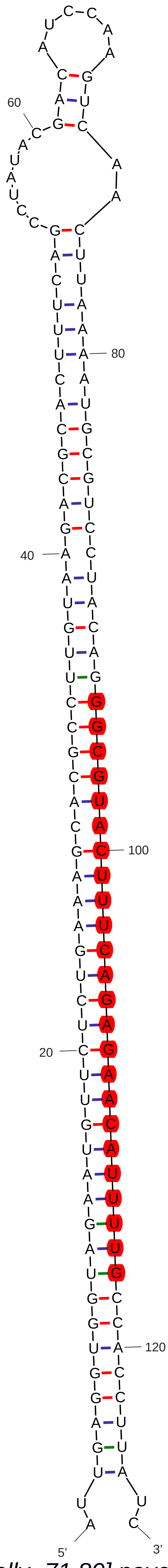

dG = -71.80 [Initially -71.80] novel_mir_2589

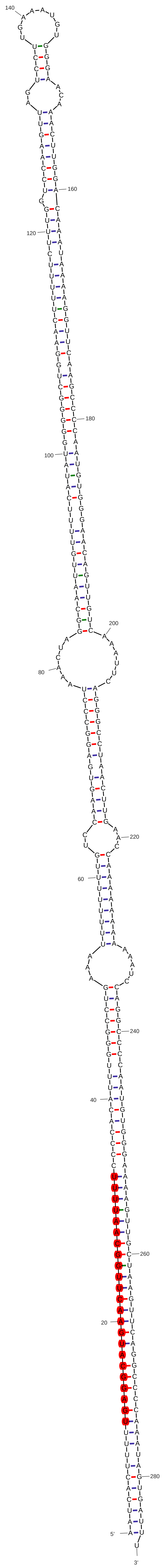

dG = -156.40 [Initially -156.40] novel_mir_4110

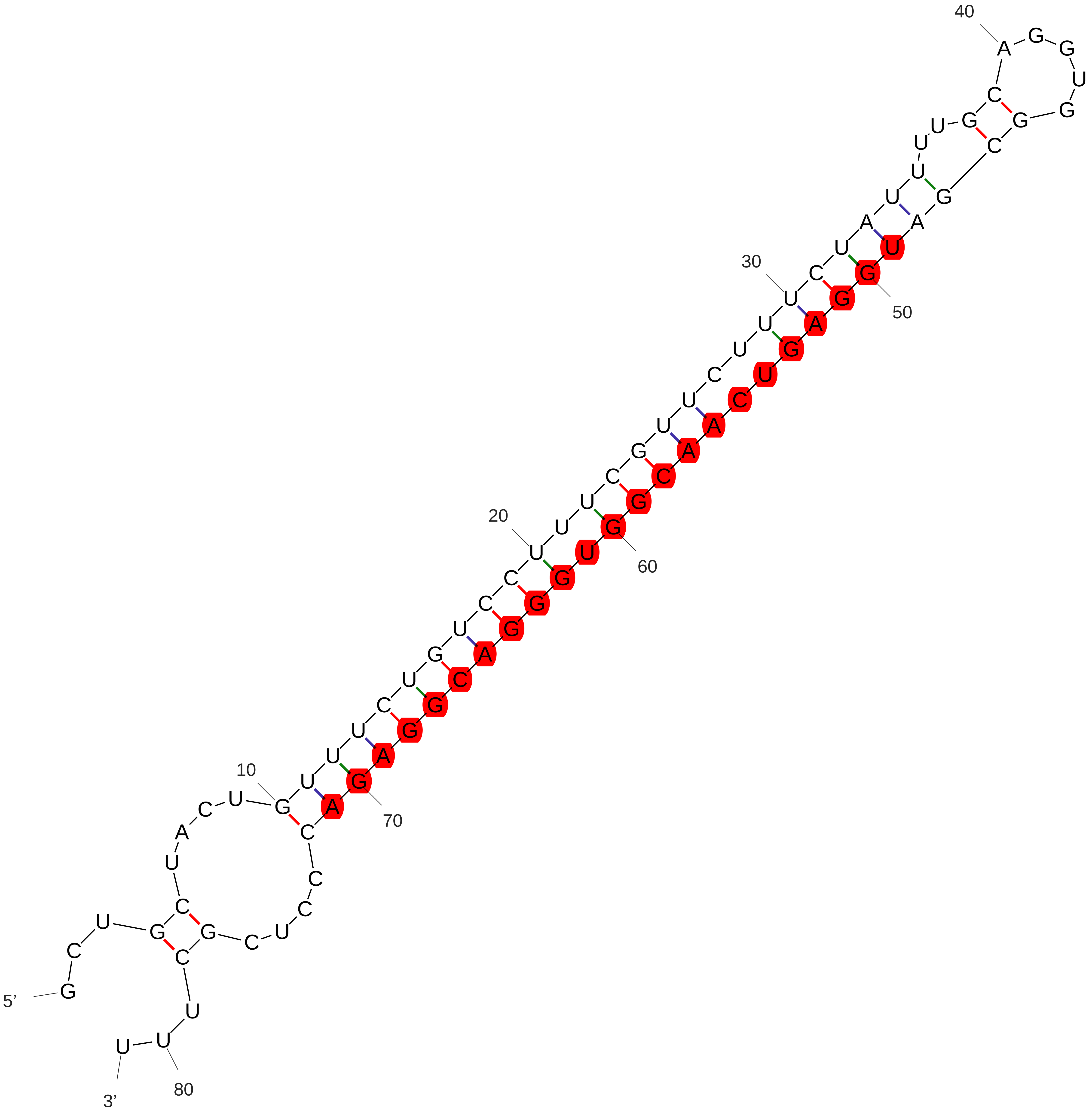

dG = -30.30 [Initially -30.30] novel_mir_4322

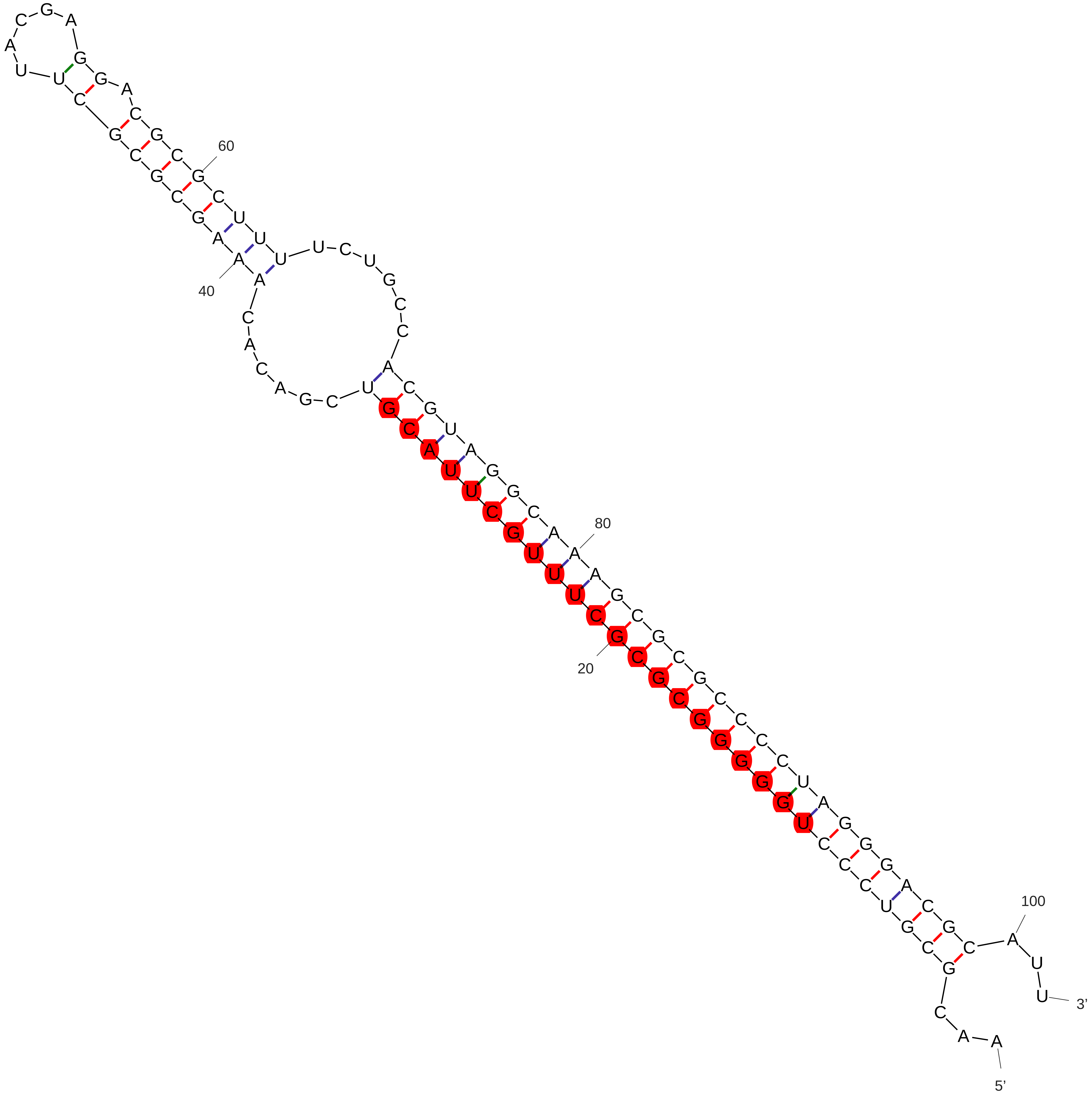

dG = -77.40 [Initially -77.40] novel_mir_4070

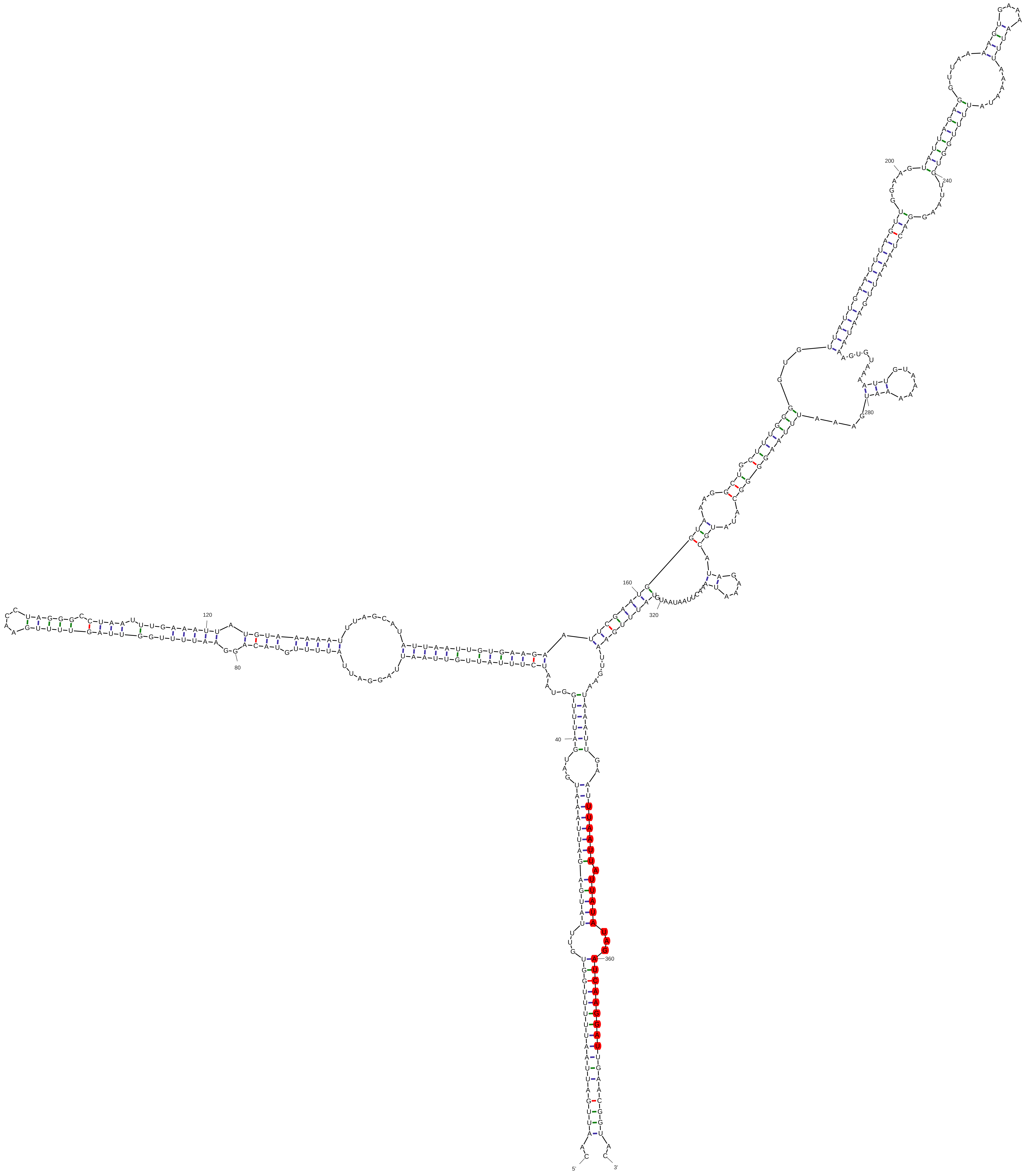

dG = -55.98 [Initially -65.60] novel_mir_4056

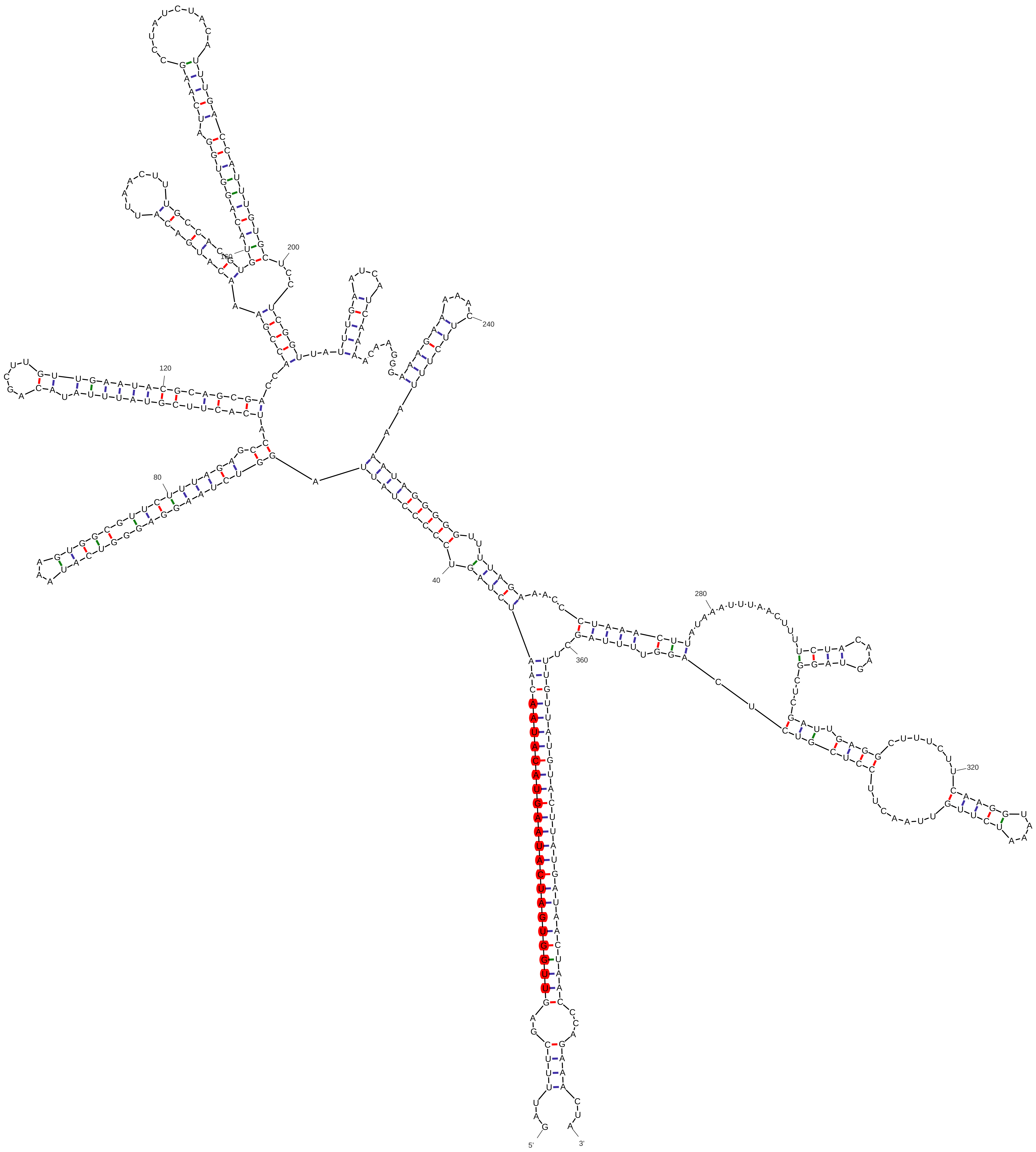

dG = -107.62 [Initially -116.40] novel_mir_4085

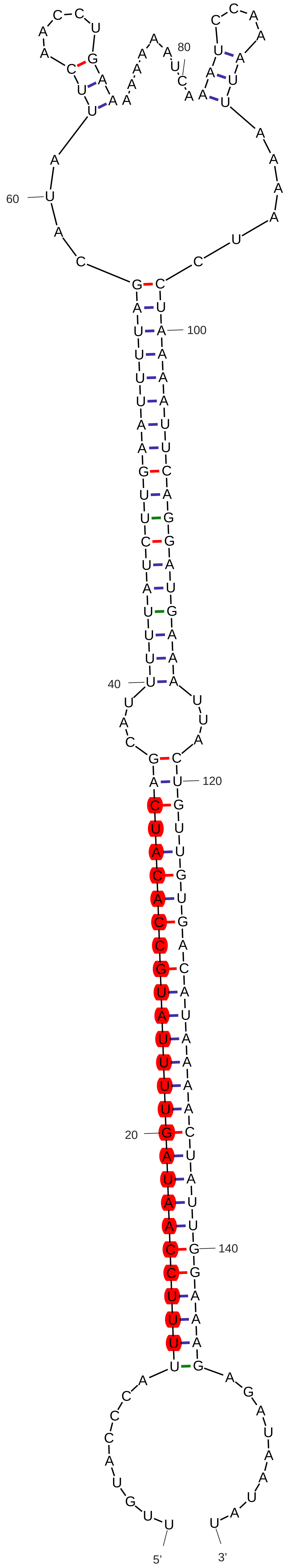

dG = -44.63 [Initially -49.00] novel_mir_5671

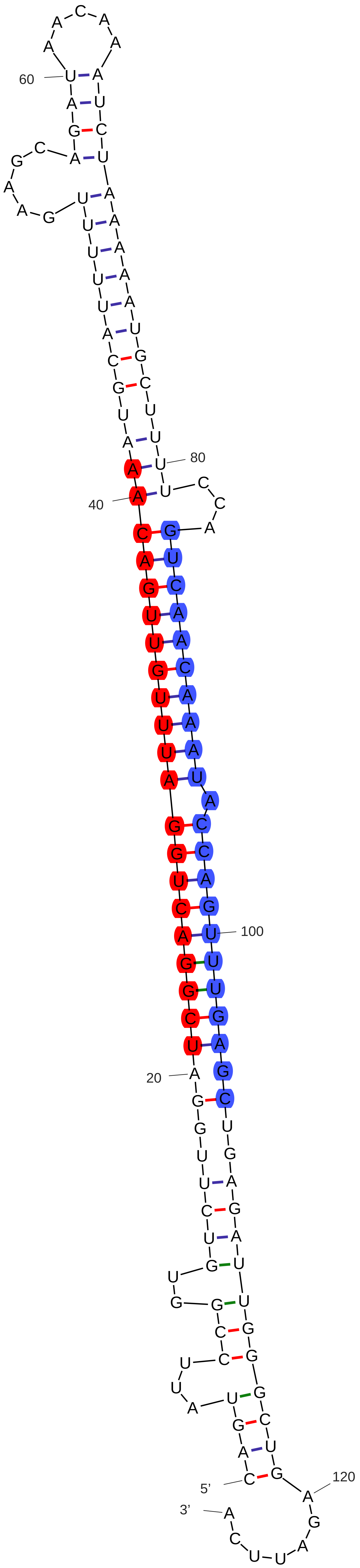

dG = -44.70 [Initially -44.70] GhmiRnF_novel_mir_1738

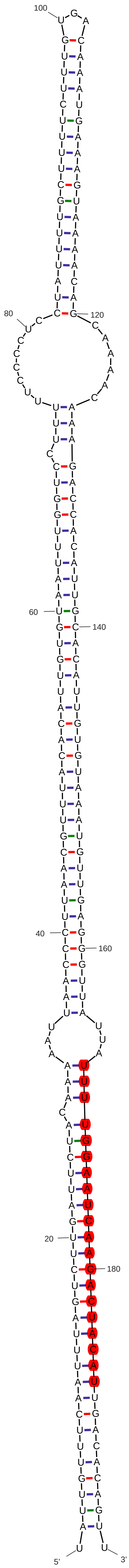

dG = -84.20 [Initially -84.20] novel_mir_4199

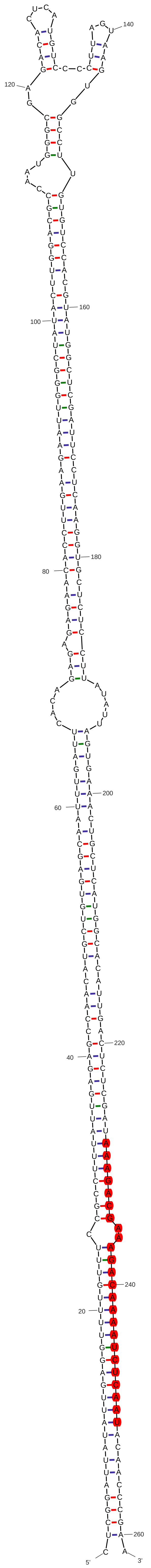

dG = -120.90 [Initially -123.60] novel_mir_4278

Output of sir_graph (©)
mfold_util 4.6

Created Sun Jan 13 07:47:28 2013

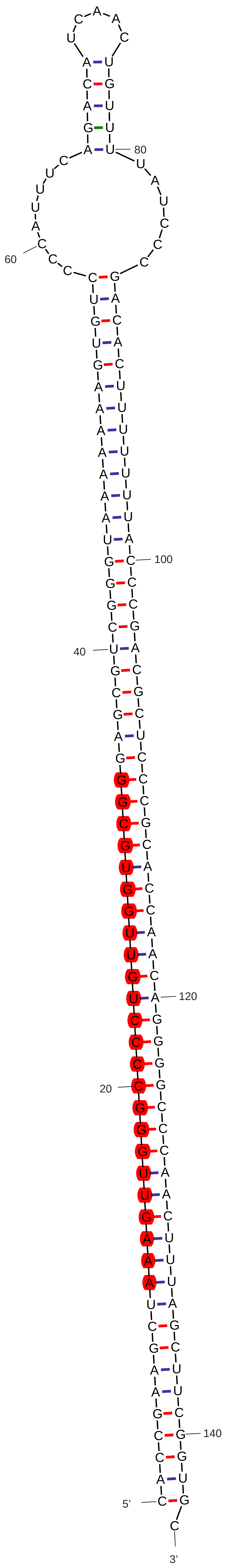

dG = -121.10 [Initially -121.10] novel_mir_1974_1

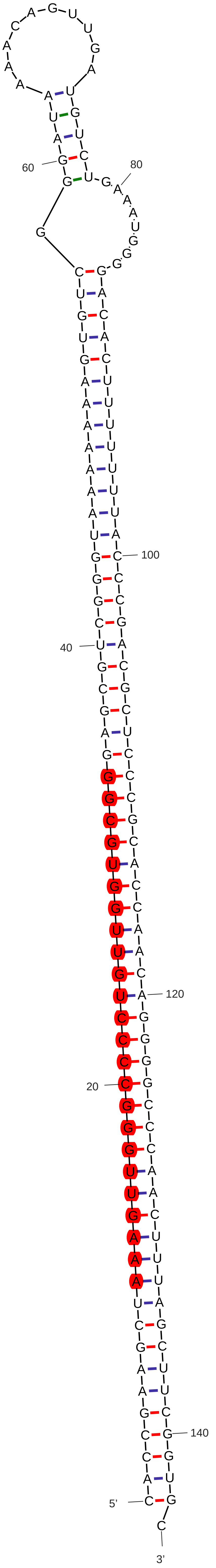

dG = -119.40 [Initially -119.40] novel_mir_1974_2

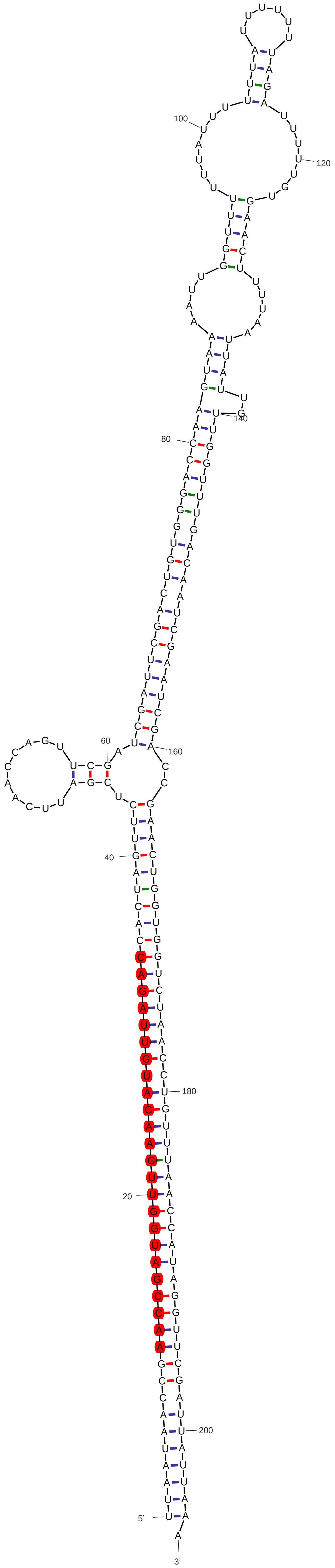

dG = -84.50 [Initially -86.90] novel_mir_2546

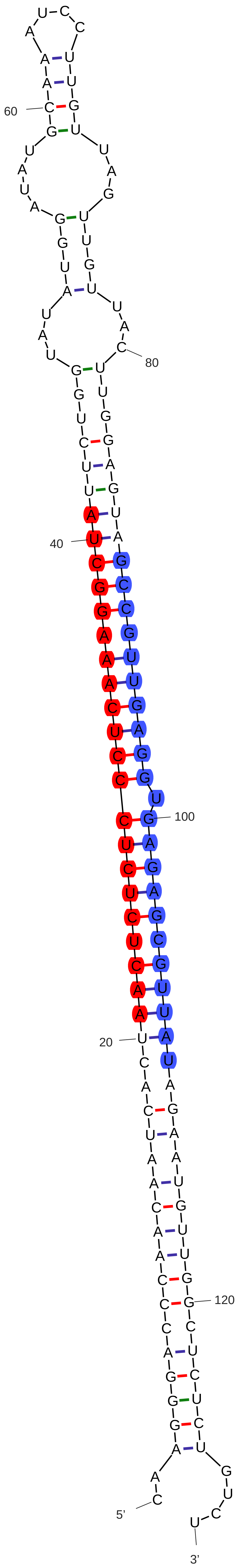

dG = -55.00 [Initially -55.00] novel_mir_5042

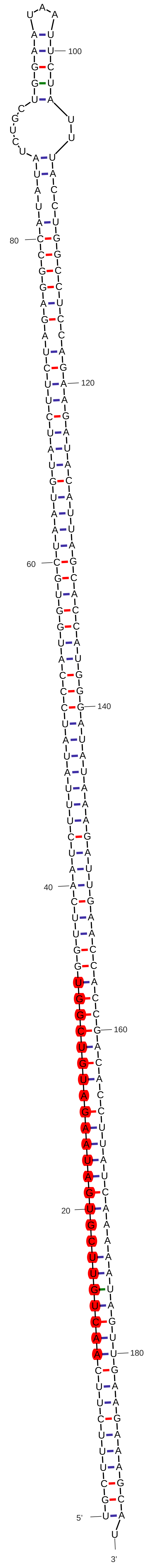

dG = -134.00 [Initially -134.00] novel_mir_4315

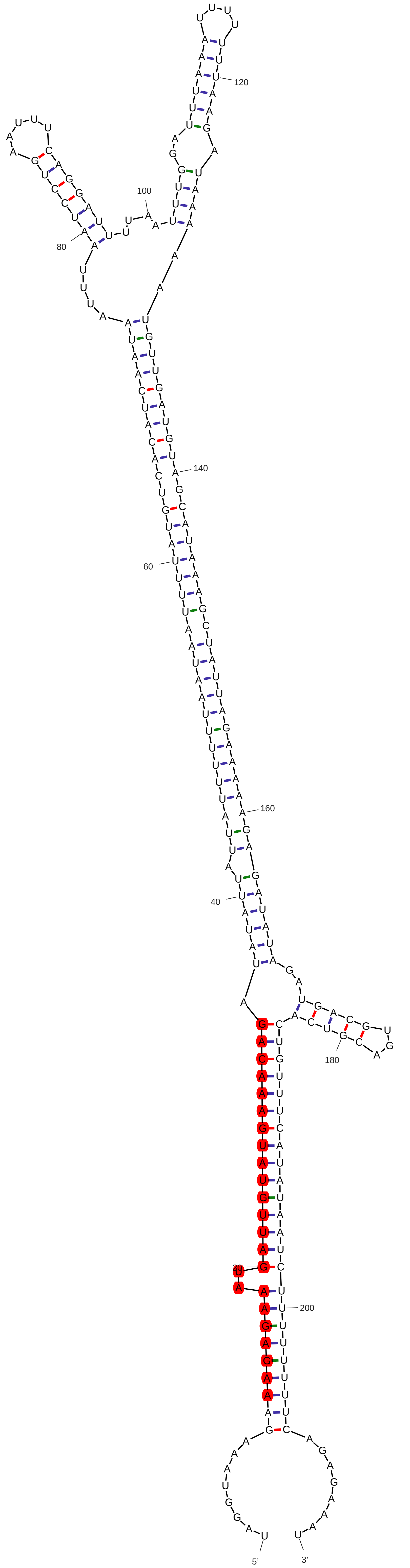

dG = -59.94 [Initially -65.70] novel_mir_2733

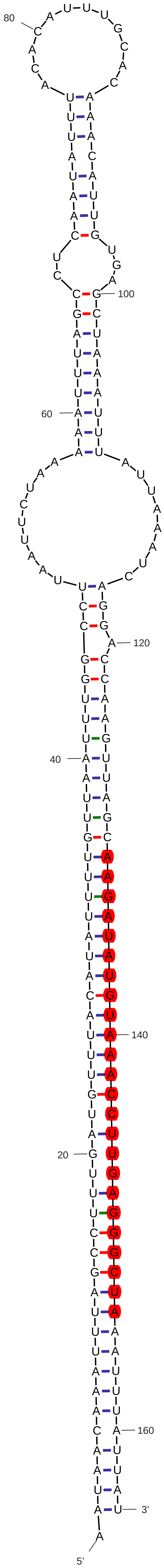

dG = -60.00 [Initially -60.00] novel_mir_3049

Output of sir_graph (©)
mfold_util 4.6

Created Sun Jan 13 07:52:28 2013

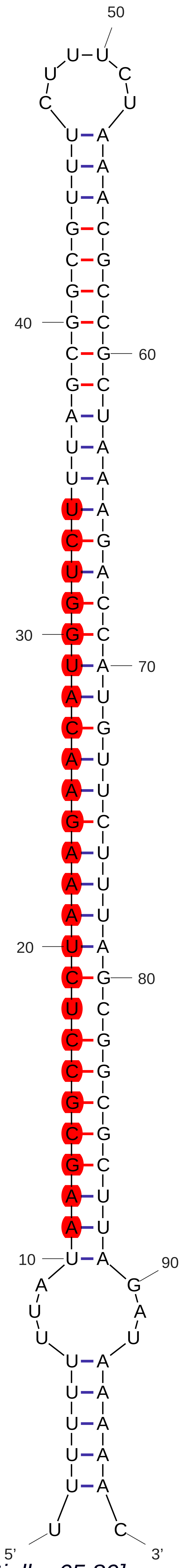

dG = -65.80 [Initially -65.80] novel_mir_941

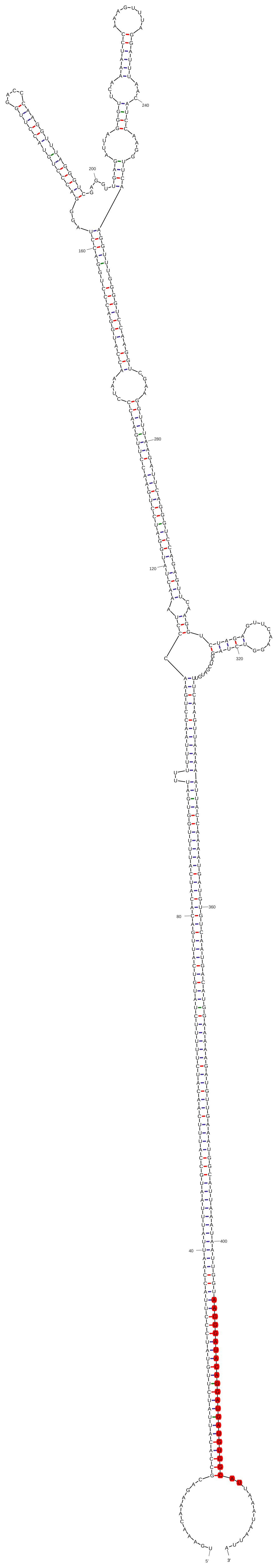

dG = -244.51 [Initially -249.70] novel_mir_4044

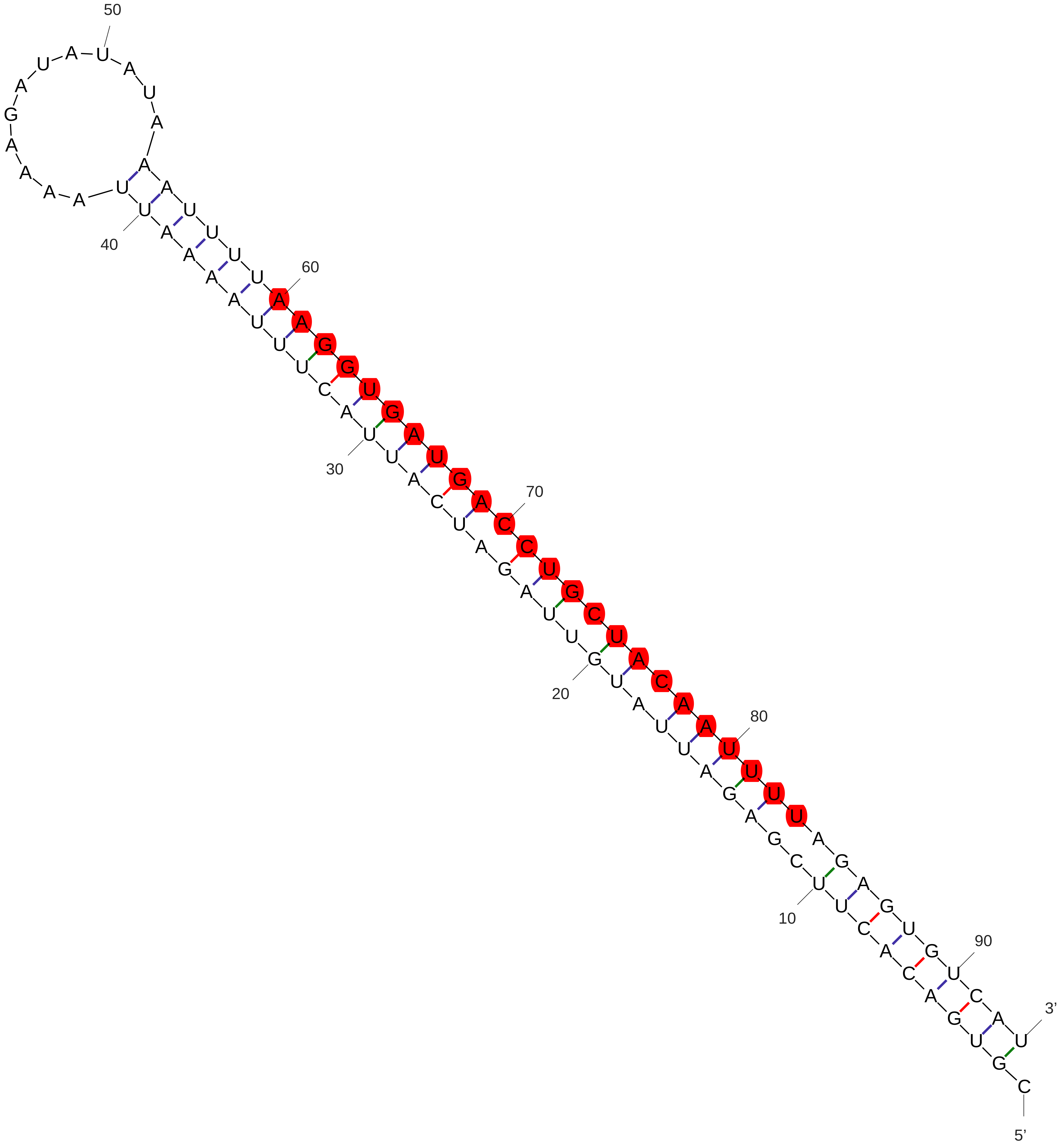

dG = -33.10 [Initially -33.10] novel_mir_949

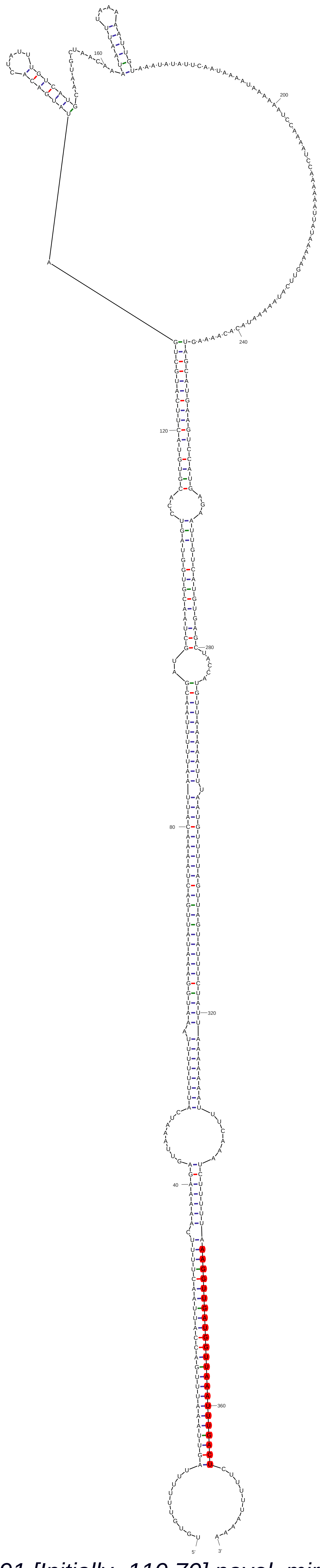

dG = -106.91 [Initially -110.70] novel_mir_999

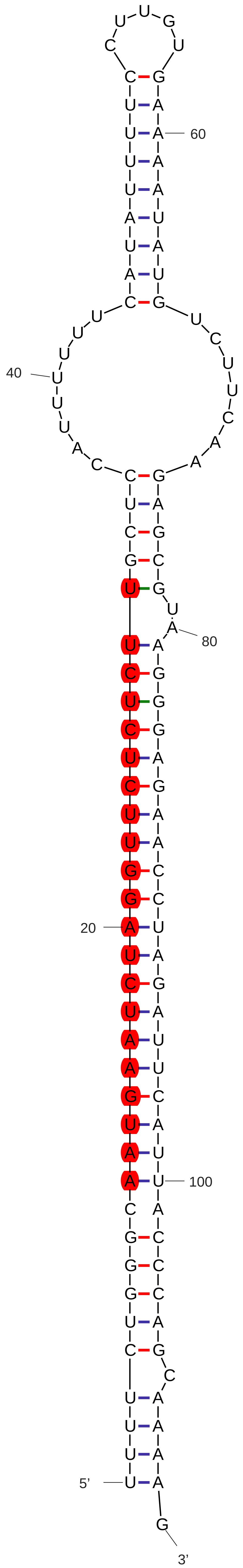

dG = -55.30 [Initially -55.30] novel_mir_4008

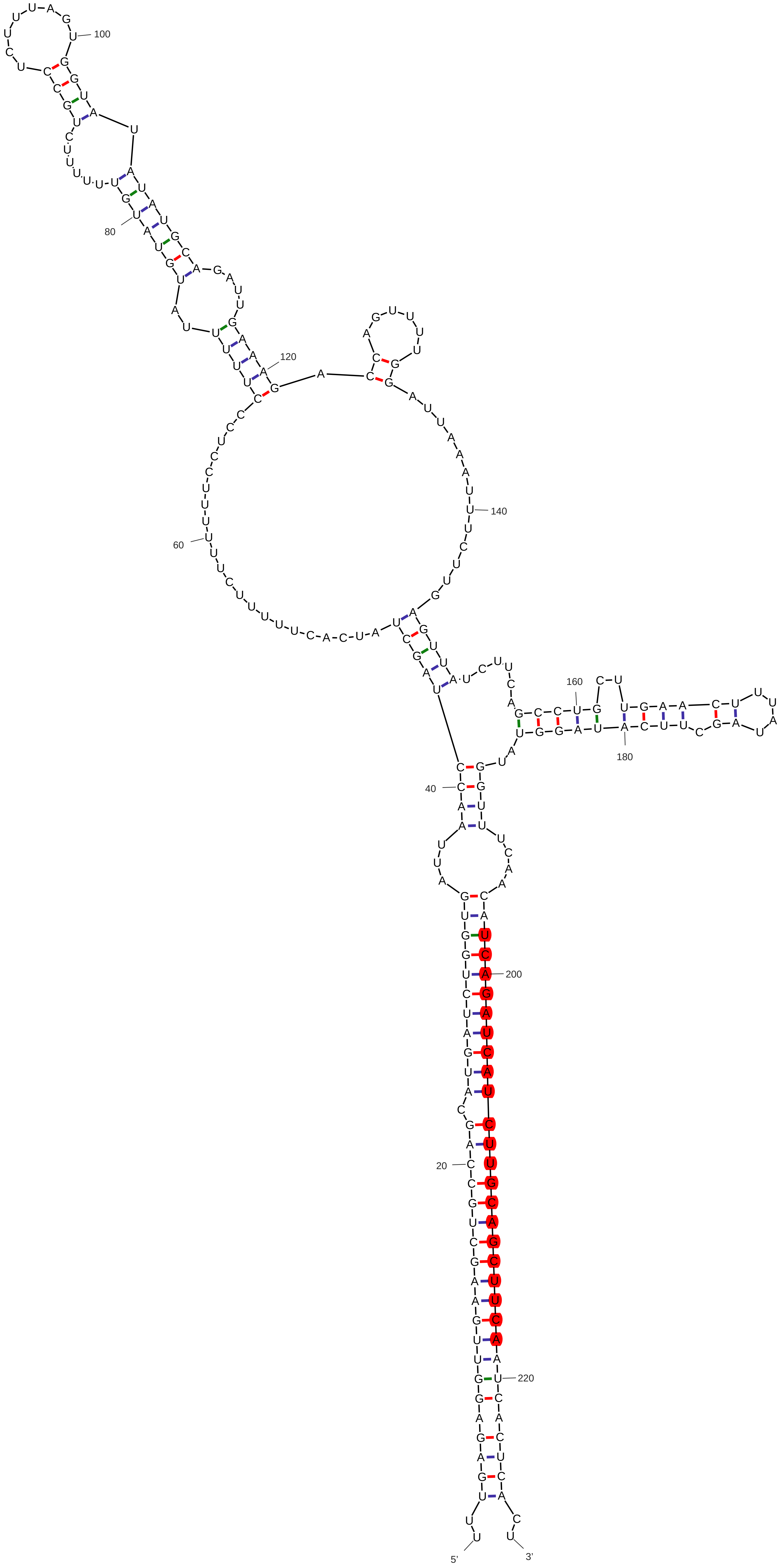

dG = -60.21 [Initially -66.00] GhmiRnH_novel_mir_853_1

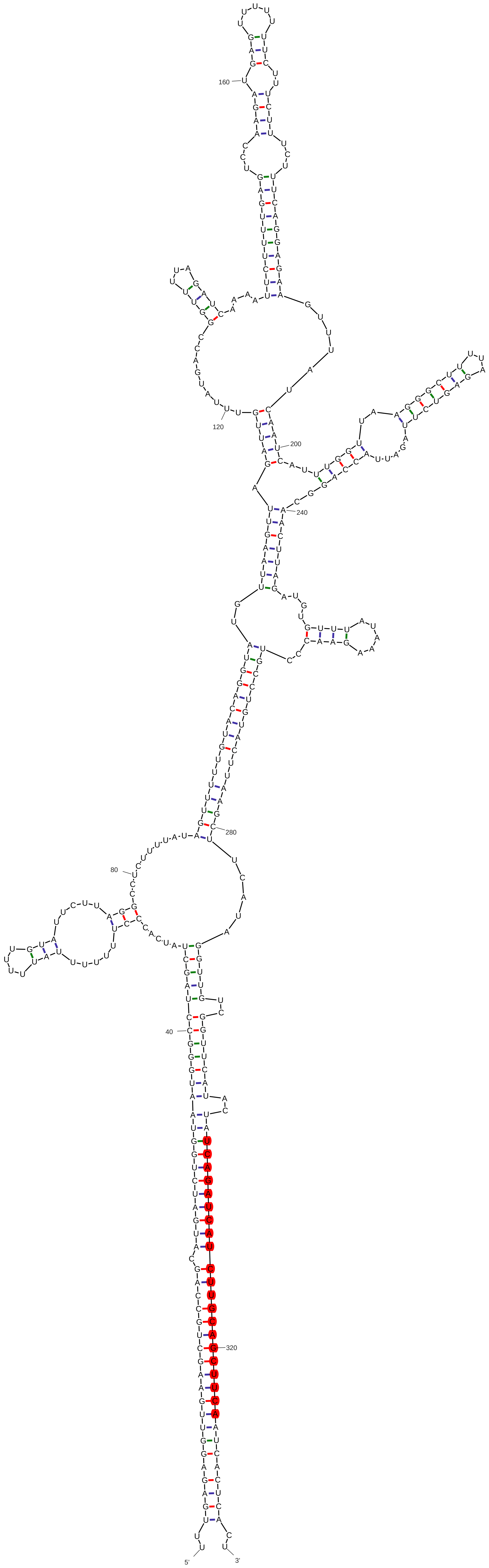

dG = -87.69 [Initially -100.90] GhmiRnH_novel_mir_853_2

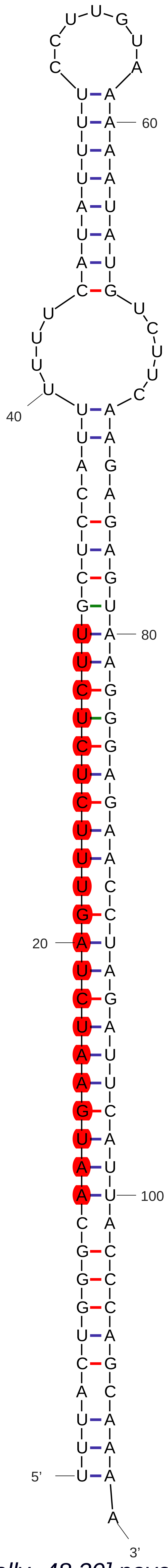

dG = -48.30 [Initially -48.30] novel_mir_4009

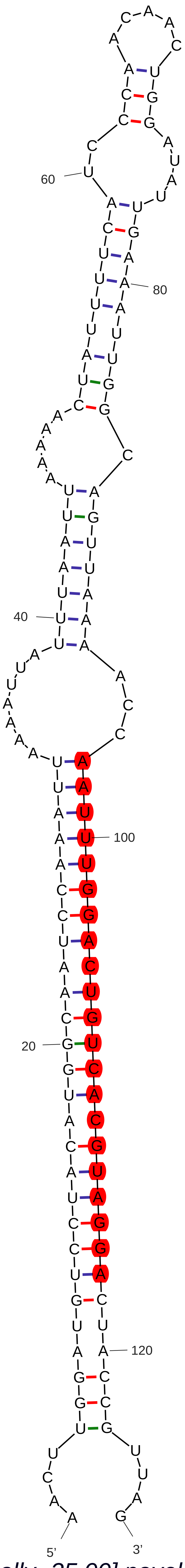

dG = -35.00 [Initially -35.00] novel_mir_4254

Output of sir_graph (©)
mfold_util 4.6

Created Thu Jan 17 22:01:24 2013

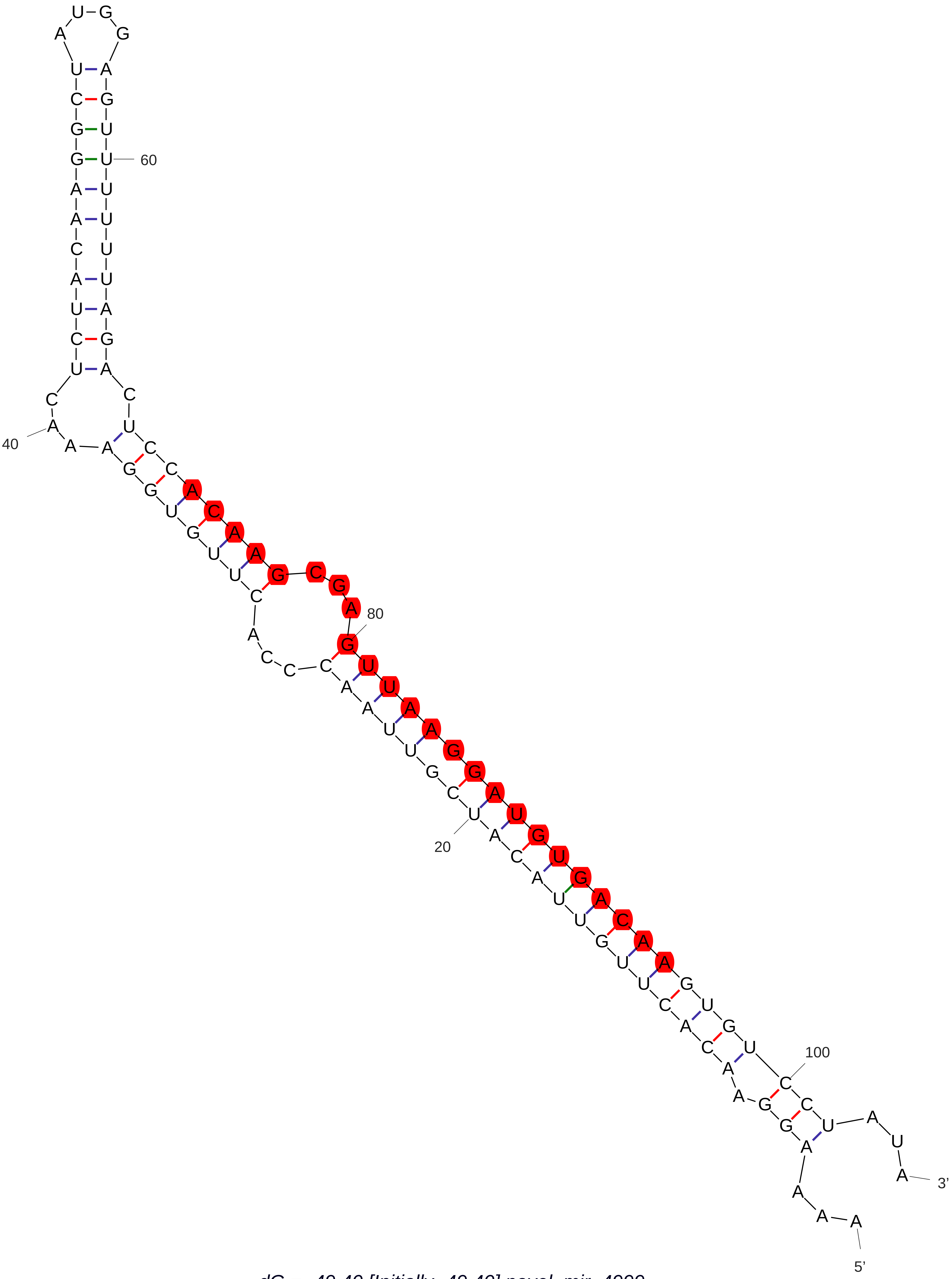

dG = -49.40 [Initially -49.40] novel_mir_4999

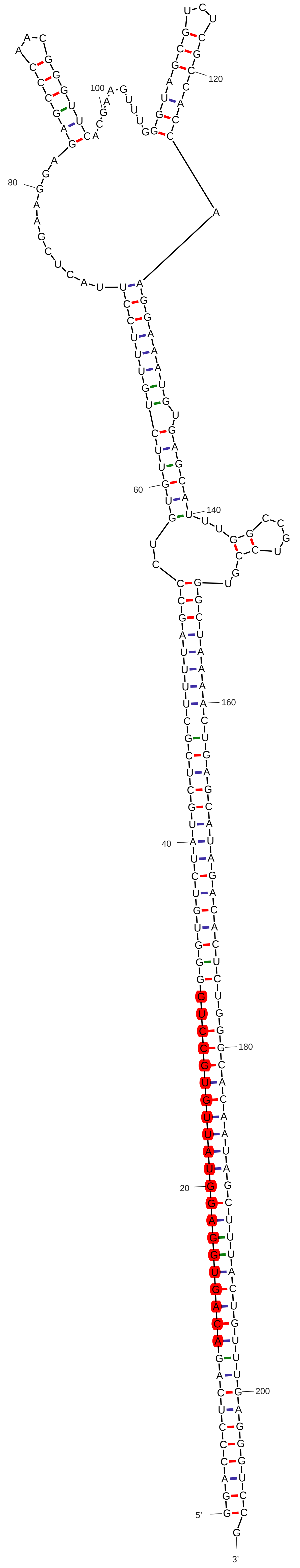

dG = -116.97 [Initially -122.70] novel_mir_2888

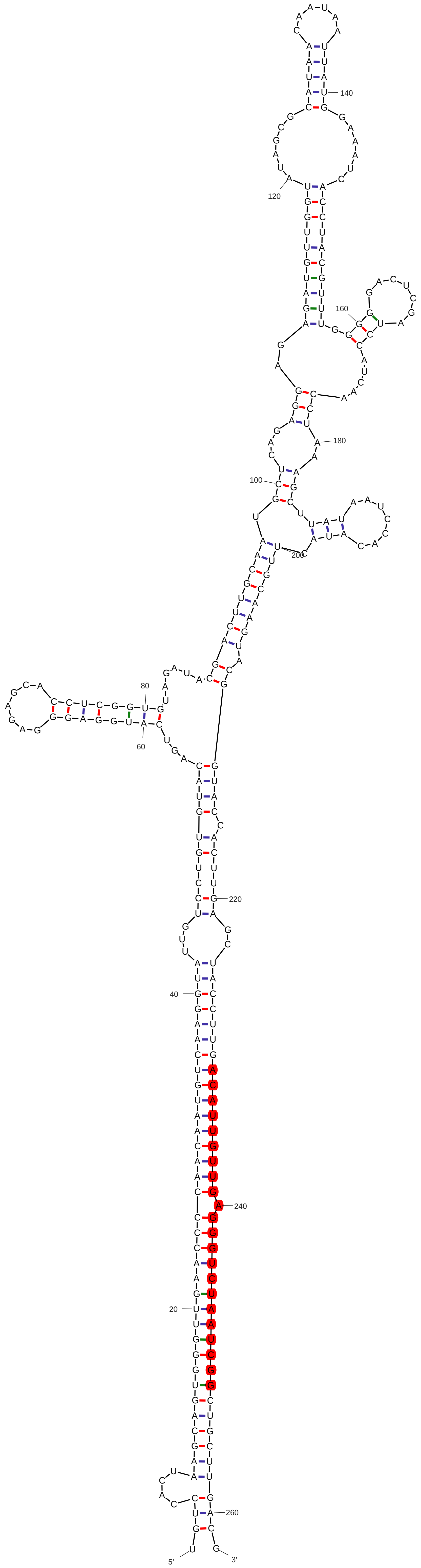

dG = -86.23 [Initially -92.90] novel_mir_5668

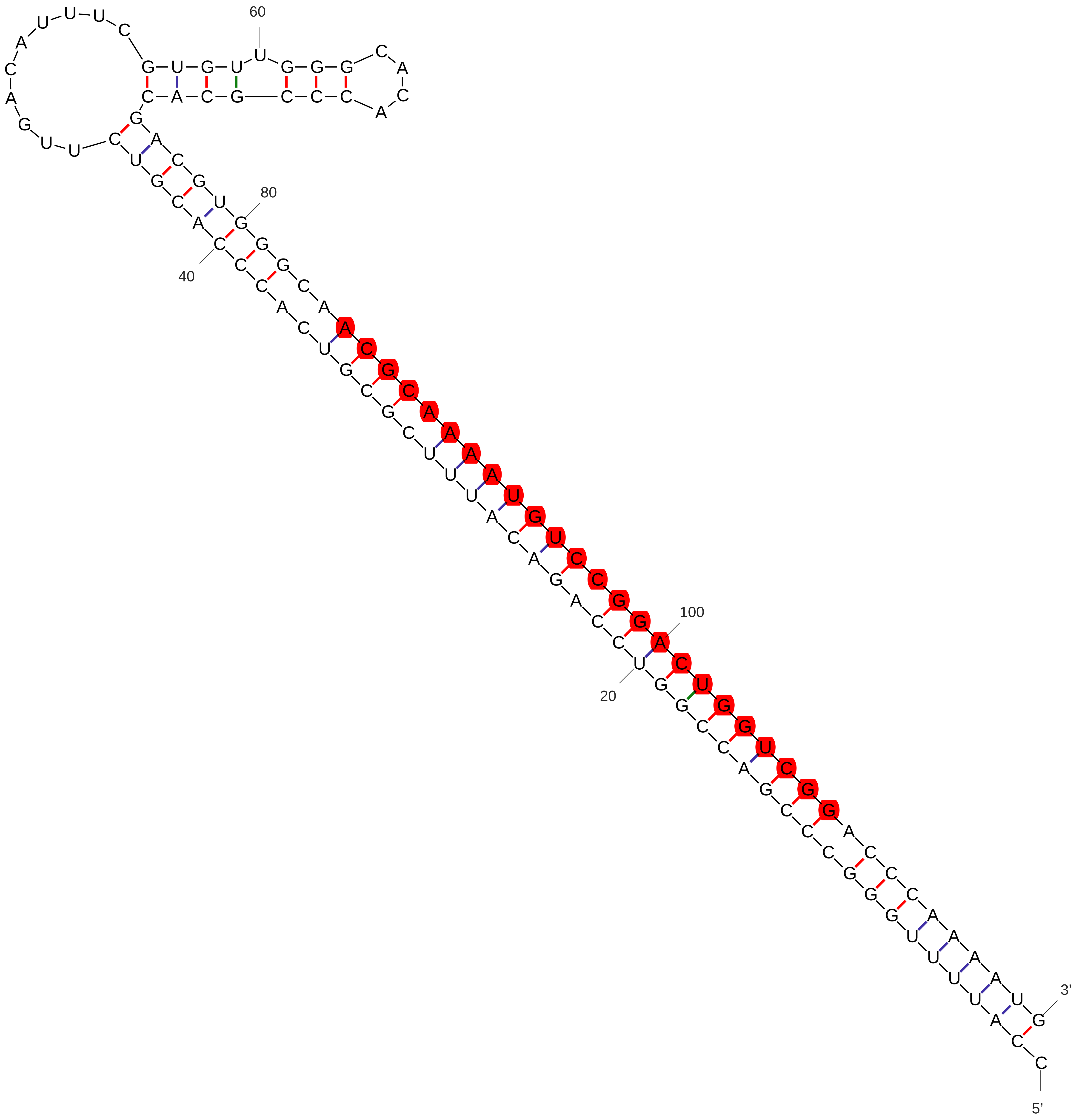

dG = -73.70 [Initially -73.70] novel_mir_2715

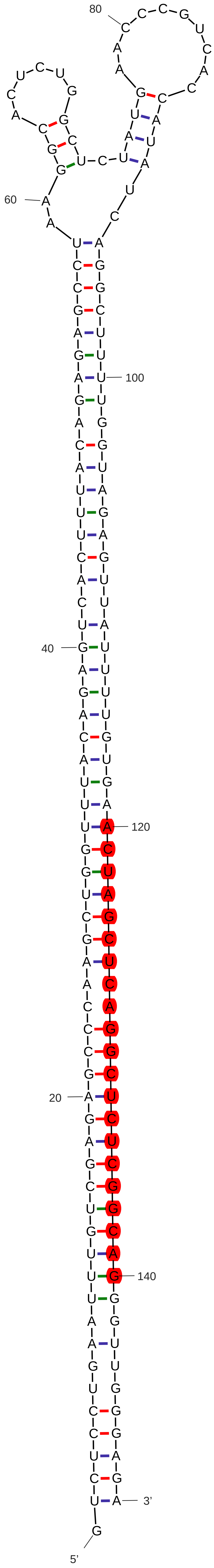

dG = -75.40 [Initially -77.70] novel_mir_4154

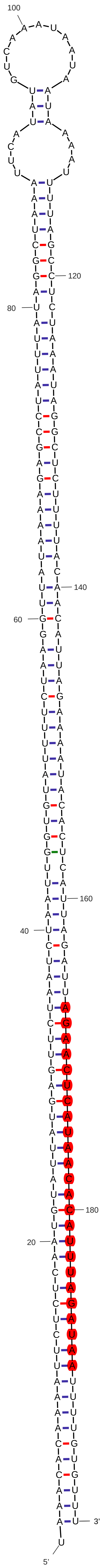

dG = -99.50 [Initially -99.50] novel_mir_2507

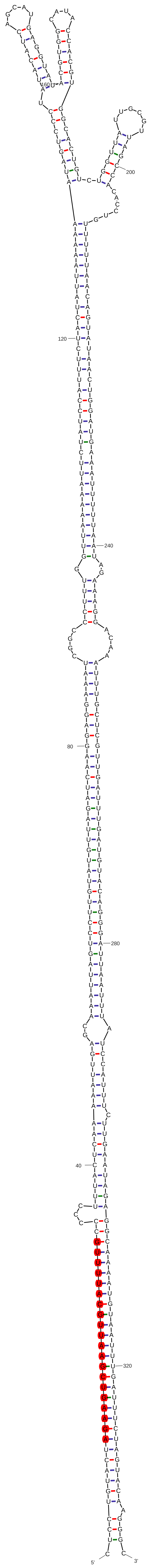

dG = -132.23 [Initially -135.10] novel_mir_2523

Output of sir_graph (©)
mfold_util 4.6

Created Sun Jan 13 08:01:07 2013

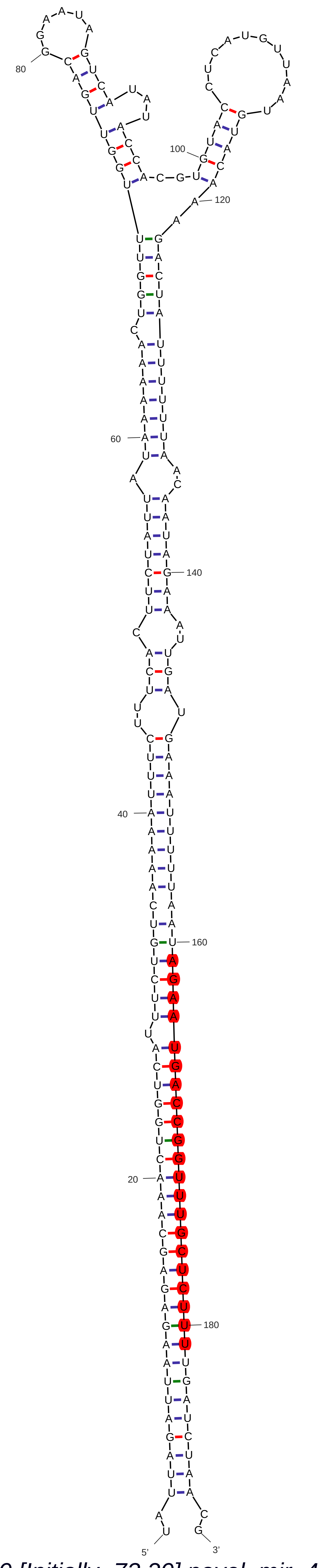

dG = -70.10 [Initially -73.20] novel_mir_4067

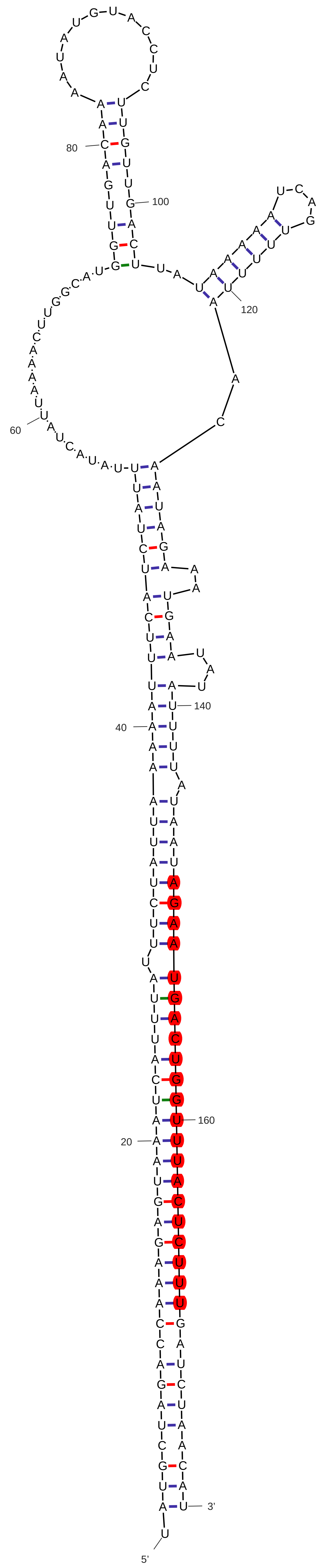

dG = -39.33 [Initially -43.10] novel_mir_5000

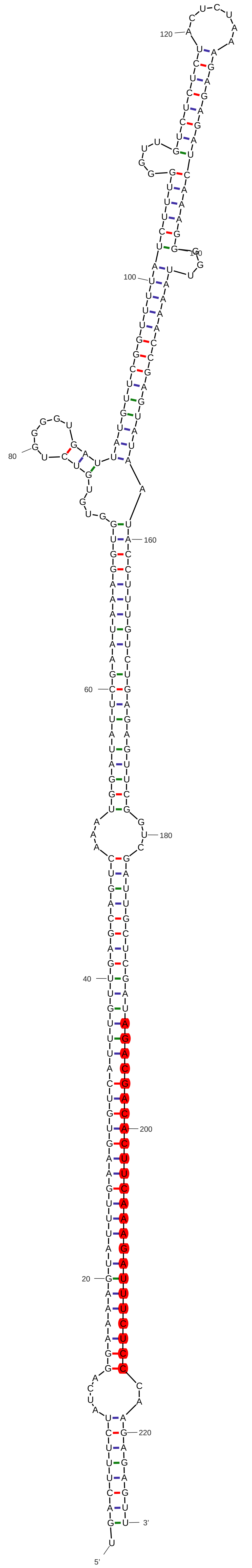

dG = -90.20 [Initially -91.30] novel_mir_5045

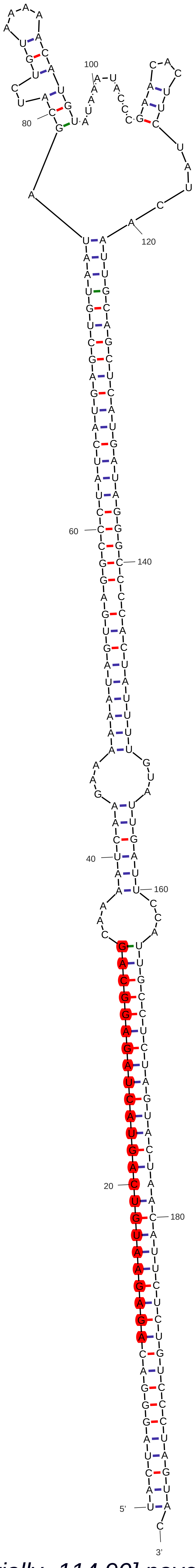

dG = -112.82 [Initially -114.90] novel_mir_5047

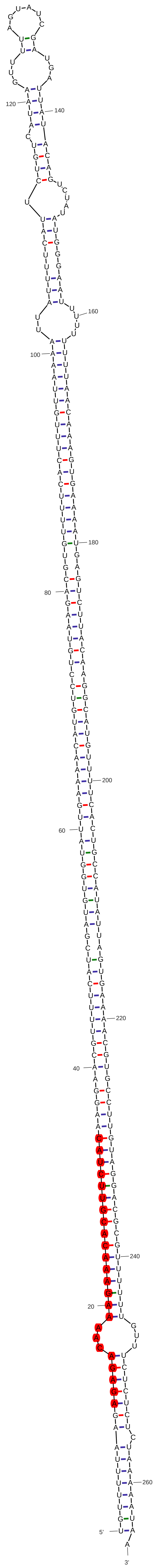

dG = -126.20 [Initially -126.20] novel_mir_3635

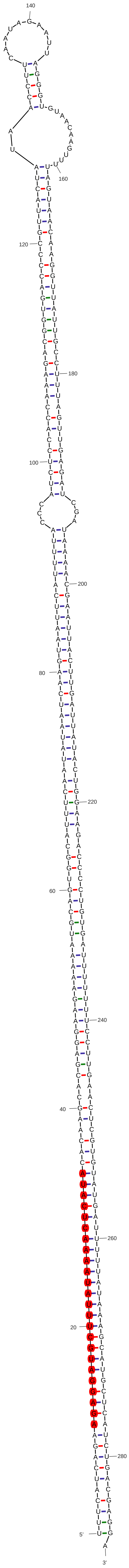

dG = -120.40 [Initially -120.40] novel_mir_5004

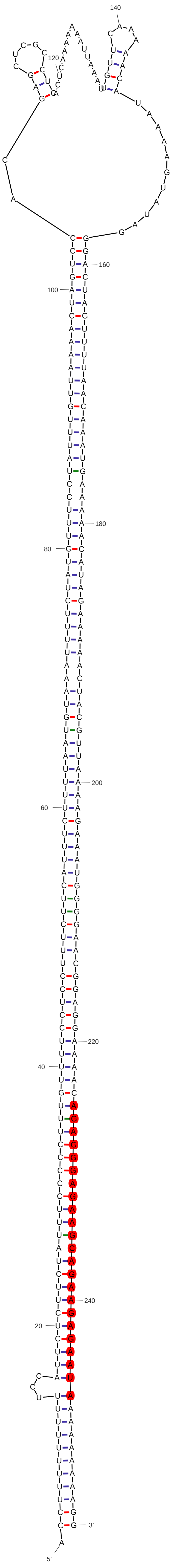

dG = -118.83 [Initially -123.70] novel_mir_1205

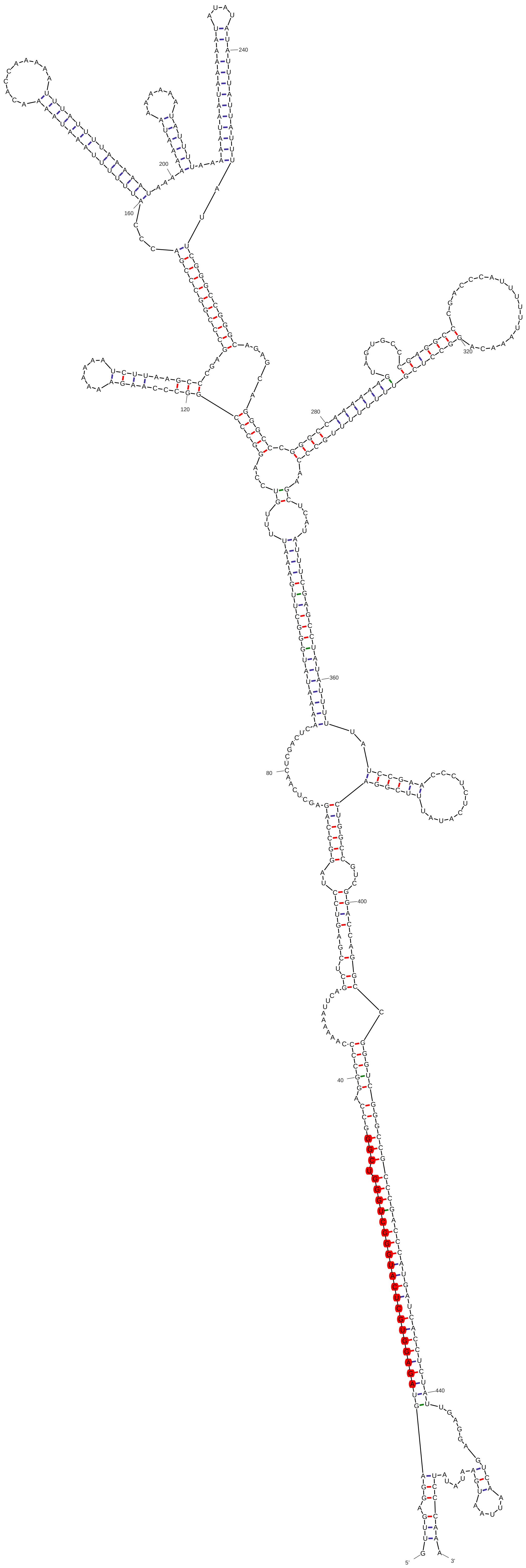

dG = -161.48 [Initially -173.70] novel_mir_841
