# Supplementary material for: Genome-wide analysis of small RNAs reveals eight fiber elongation-related and 257 novel microRNAs in elongating cotton fiber cells
Source: BMC Genomics. 2013 Sep 17;14:629. doi: 10.1186/1471-2164-14-629 (PMC3849097; doi:10.1186/1471-2164-14-629)
Supplement: Additional file 7: Figure S4 — Genomic overview of 174 known and 311 novel GhmiRNA gene loci on the 13 G. raimondii chromosomes. There were 314 novel miRNA gene loci, as shown in Additional file 5. The three miRNA gene loci of GhmiRnA and GhmiRnB were not anchored on any of the 13 chromosomes of the G. raimondii genome, so 311 novel miRNA gene loci were mapped on the 13 G. raimondii chromosomes. [file 1471-2164-14-629-S7.docx]

**Additional Figure S4:**

**Genomic overview of 174 known and 311 novel GhmiRNA gene loci on the 13 *G. raimondii* chromosomes.** There were 314 novel miRNA gene loci, as shown in Additional file 5. The three miRNA gene loci of GhmiRnA and GhmiRnB were not anchored on any of the 13 chromosomes of the G. raimondii genome, so 311 novel miRNA gene loci were mapped on the 13 G. raimondii chromosomes.

**
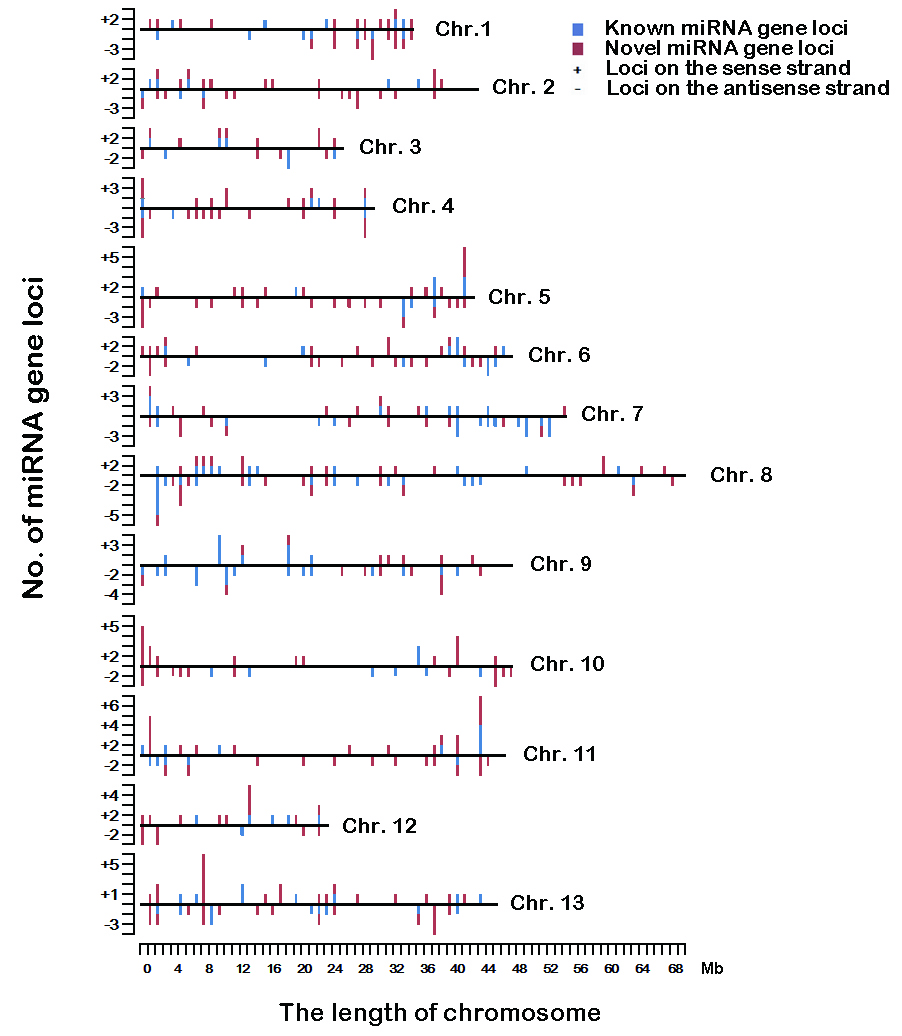
**
