# Supplementary material for: Genome-wide analysis of small RNAs reveals eight fiber elongation-related and 257 novel microRNAs in elongating cotton fiber cells
Source: BMC Genomics. 2013 Sep 17;14:629. doi: 10.1186/1471-2164-14-629 (PMC3849097; doi:10.1186/1471-2164-14-629)

**Additional Figure S5:**

**Quantitative RT-PCR analysis of GhmiR156, GhmiR167 and GhmiR168.** The data represent the mean values ± SD of three replicates. U6 was used as a reference gene. Types A, B, and C are shown in red, yellow, and blue, respectively, in Figure 2A.


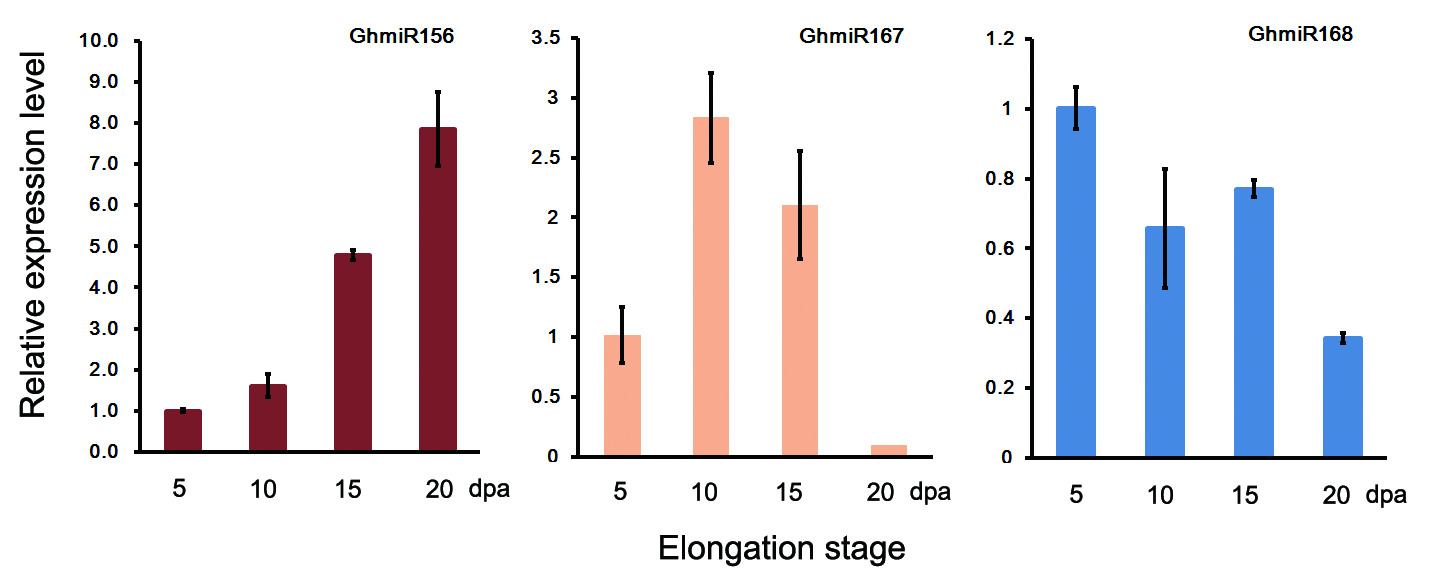

Supplement: Additional file 8: Figure S5 — Quantitative RT-PCR analysis of GhmiR156, GhmiR167 and GhmiR168. The data represent the mean values ± SD of three replicates. U6 was used as a reference gene. Types A, B and C are shown in red, yellow, and blue, respectively, in Figure 2A. [file 1471-2164-14-629-S8.docx]
