# Supplementary figures and images for: Genome-wide analysis of small RNAs reveals eight fiber elongation-related and 257 novel microRNAs in elongating cotton fiber cells
Source: BMC Genomics. 2013 Sep 17;14:629. doi: 10.1186/1471-2164-14-629 (PMC3849097; doi:10.1186/1471-2164-14-629)

**Additional Figure S6:**

**Size distribution of cotton ta-siRNAs generated from the DW503626 gene.**


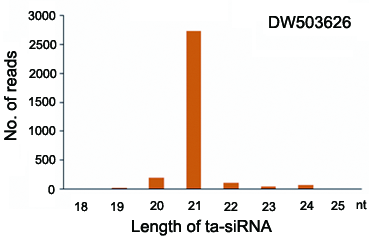

Supplement: Additional file 9: Figure S6 — Size distribution of cotton ta-siRNA generated from the DW503626 gene. [file 1471-2164-14-629-S9.docx]
