# Supplementary material for: Genome-wide analysis of small RNAs reveals eight fiber elongation-related and 257 novel microRNAs in elongating cotton fiber cells
Source: BMC Genomics. 2013 Sep 17;14:629. doi: 10.1186/1471-2164-14-629 (PMC3849097; doi:10.1186/1471-2164-14-629)
Supplement: Additional file 10: Table S4 — Target prediction for novel cotton fiber elongation-related miRNAs. [file 1471-2164-14-629-S10.docx]

**Additional Table S4:**

**Target prediction for novel cotton fiber elongation-related miRNAs**

| **miRNA** | **Predicted target** | **Mismatch** | **UPE** | **Inhibition** | **E-value** | **Target gene annotation** |
| --- | --- | --- | --- | --- | --- | --- |
| GhmiRnA | GR708796 | 2.5 | 16.38 | Cleavage | 7.00E-57 | pentatricopeptide repeat-containing protein [Raphanus sativus] |
|  |  |  |  |  | 7.00E-57 | non restoring pentatricopeptide repeat [Raphanus sativus] |
|  |  |  |  |  | 5.00E-56 | pentatricopeptide repeat-containing protein [Arabidopsis lyrata subsp. lyrata]. |
| GhmiRnA | TC269108 | 2.5 | 15.22 | Cleavage | 4.00E-83 | predicted protein [Populus trichocarpa] |
|  |  |  |  |  | 3.00E-81 | predicted protein [Populus trichocarpa] |
|  |  |  |  |  | 4.00E-81 | predicted protein [Populus trichocarpa] |
| GhmiRnA | ES809349 | 2.0 | 17.74 | Cleavage | 4.00E-46 | predicted protein [Populus trichocarpa] |
|  |  |  |  |  | 2.00E-45 | predicted protein [Populus trichocarpa] |
|  |  |  |  |  | 6.00E-43 | predicted protein [Populus trichocarpa] |
| GhmiRnA | AI727415 | 3.0 | 18.44 | Cleavage | 2.00E-61 | PREDICTED: hypothetical protein [Vitis vinifera] |
|  |  |  |  |  | 3.00E-60 | PREDICTED: hypothetical protein [Vitis vinifera] |
|  |  |  |  |  | 5.00E-60 | PREDICTED: hypothetical protein [Vitis vinifera] |
| GhmiRnA | TC266681 | 3.0 | 21.14 | Cleavage | 5.00E-90 | predicted protein [Populus trichocarpa] |
|  |  |  |  |  | 8.00E-90 | predicted protein [Populus trichocarpa] |
|  |  |  |  |  | 1.00E-88 | PREDICTED: hypothetical protein [Vitis vinifera] |
| GhmiRnA | CO077591 | 2.5 | 12.66 | Translation | 2.00E-08 | hypothetical protein VITISV_041503 [Vitis vinifera] |
|  |  |  |  |  | 3.00E-08 | PREDICTED: hypothetical protein [Vitis vinifera] |
|  |  |  |  |  | 1.00E-07 | N-6 Adenine-specific DNA methylase; Homeodomain-related [Medicago |
| GhmiRnA | TC262358 | 3.0 | 19.64 | Cleavage | 4.00E-100 | predicted protein [Populus trichocarpa] |
|  |  |  |  |  | 4.00E-100 | predicted protein [Populus trichocarpa] |
|  |  |  |  |  | 6.00E-100 | PREDICTED: hypothetical protein [Vitis vinifera] |
| GhmiRnA | TC259860 | 3.0 | 16.46 | Cleavage | 6.00E-51 | pentatricopeptide repeat-containing protein, putative [Ricinus communis]. |
|  |  |  |  |  | 8.00E-50 | pentatricopeptide repeat-containing protein [Arabidopsis thaliana] |
|  |  |  |  |  | 1.00E-49 | unnamed protein product [Arabidopsis thaliana] |
| GhmiRnA | ES801805 | 3.0 | 14.03 | Cleavage | 2.00E-55 | pentatricopeptide repeat-containing protein, putative [Ricinus communis]. |
|  |  |  |  |  | 1.00E-52 | predicted protein [Populus trichocarpa] |
|  |  |  |  |  | 2.00E-52 | predicted protein [Populus trichocarpa] |
| GhmiRnA | DT527363 | 3.0 | 17.06 | Cleavage | 1.00E-35 | sterol delta-7 reductase DWF5 [Gossypium hirsutum] |
|  |  |  |  |  | 2.00E-34 | sterol delta-7 reductase [Arabidopsis thaliana] |
|  |  |  |  |  | 2.00E-34 | 7-dehydrocholesterol reductase [Arabidopsis thaliana] |
| GhmiRnB | ES832131 | 2.5 | 18.00 | Cleavage | 1.00E-95 | PREDICTED: hypothetical protein [Vitis vinifera] |
|  |  |  |  |  | 1.00E-95 | unnamed protein product [Vitis vinifera] |
|  |  |  |  |  | 6.00E-94 | auxin-induced protein [Arabidopsis thaliana] |
| GhmiRnB | TC257014 | 3.0 | 7.55 | Cleavage | 1.00E-59 | PREDICTED: hypothetical protein [Vitis vinifera] |
|  |  |  |  |  | 2.00E-58 | unnamed protein product [Vitis vinifera] |
|  |  |  |  |  | 7.00E-55 | alpha-galactosidase/alpha-n-acetylgalactosaminidase, putative [Ricinus communis]. |
| GhmiRnB | DR452627 | 3.0 | 14.22 | Cleavage | 2.00E-40 | PREDICTED: hypothetical protein isoform 1 [Vitis vinifera] |
|  |  |  |  |  | 3.00E-40 | RecName: Full=60S ribosomal protein L38 |
|  |  |  |  |  | 4.00E-40 | unnamed protein product [Vitis vinifera] |
| GhmiRnB | BG440189 | 3.0 | 15.88 | Cleavage | 5.00E-52 | unknown [Glycine max] |
|  |  |  |  |  | 2.00E-51 | predicted protein [Populus trichocarpa] |
|  |  |  |  |  | 6.00E-51 | unknown [Glycine max] |
| GhmiRnB | EV485538 | 3.0 | 15.92 | Cleavage | 5.00E-52 | unknown [Glycine max] |
|  |  |  |  |  | 2.00E-51 | predicted protein [Populus trichocarpa] |
|  |  |  |  |  | 6.00E-51 | unknown [Glycine max] |
| GhmiRnB | DR452595 | 3.0 | 17.11 | Cleavage | 1.00E-35 | predicted protein [Populus trichocarpa] |
|  |  |  |  |  | 2.00E-35 | unknown [Glycine max] |
|  |  |  |  |  | 4.00E-35 | predicted protein [Populus trichocarpa] |
| GhmiRnB | TC255909 | 3.0 | 17.45 | Cleavage | 5.00E-165 | predicted protein [Populus trichocarpa] |
|  |  |  |  |  | 3.00E-162 | predicted protein [Populus trichocarpa] |
|  |  |  |  |  | 1.00E-158 | PREDICTED: transaldolase-like [Glycine max] |
| GhmiRnB | TC252473 | 3.0 | 16.78 | Cleavage | 9.00E-139 | predicted protein [Populus trichocarpa] |
|  |  |  |  |  | 3.00E-138 | PREDICTED: transaldolase-like [Glycine max] |
|  |  |  |  |  | 3.00E-138 | predicted protein [Populus trichocarpa] |
| GhmiRnB | TC246805 | 3.0 | 17.56 | Cleavage | 3.00E-114 | predicted protein [Populus trichocarpa] |
|  |  |  |  |  | 2.00E-110 | predicted protein [Populus trichocarpa] |
|  |  |  |  |  | 2.00E-107 | PREDICTED: transaldolase-like [Glycine max] |
| GhmiRnC | AW726550 | 3.0 | 8.36 | Cleavage | 1.00E-05 | predicted protein [Populus trichocarpa] |
|  |  |  |  |  | 1.00E-05 | unnamed protein product [Vitis vinifera] |
|  |  |  |  |  | 6.00E-05 | PREDICTED: hypothetical protein [Vitis vinifera] |
| GhmiRnC | TC275688 | 2.0 | 0.05 | Cleavage | 3.00E-09 | hypothetical protein VITISV_009914 [Vitis vinifera] |
|  |  |  |  |  | 1.00E-08 | endo-1,4-beta-glucancase precursor [Glycine max] |
|  |  |  |  |  | 1.00E-08 | membrane-anchored endo-1,4-beta-glucanase [Gossypium hirsutum] |
| GhmiRnC | EX168214 | 3.0 | 3.47 | Cleavage | 1.00E-65 | membrane-anchored endo-1,4-beta-glucanase [Gossypium herbaceum subsp. africanum] |
|  |  |  |  |  | 3.00E-60 | villin 1-4, putative [Ricinus communis] |
|  |  |  |  |  | 1.00E-59 | PREDICTED: hypothetical protein [Vitis vinifera] |
| GhmiRnC | DR453997 | 3.0 | 6.52 | Cleavage | 3.00E-06 | unknown [Populus trichocarpa] |
| GhmiRnC | TC246803 | 3.0 | 20.86 | Cleavage | 2.00E-143 | predicted protein [Populus trichocarpa] |
|  |  |  |  |  | 8.00E-138 | PREDICTED: hypothetical protein [Vitis vinifera] |
|  |  |  |  |  | 5.00E-137 | predicted protein [Populus trichocarpa] |
| GhmiRnC | TC245877 | 3.0 | 5.17 | Cleavage | 7.00E-27 | uncharacterized protein [Arabidopsis thaliana] |
|  |  |  |  |  | 4.00E-22 | predicted protein [Populus trichocarpa] |
|  |  |  |  |  | 5.00E-22 | predicted protein [Populus trichocarpa] |
| GhmiRnC | DW518192 | 3.0 | 8.34 | Translation | 5.00E-77 | predicted protein [Populus trichocarpa] |
|  |  |  |  |  | 1.00E-75 | H/ACA ribonucleoprotein complex subunit, putative [Ricinus communis]. |
|  |  |  |  |  | 2.00E-75 | JHL10I11.6 [Jatropha curcas] |
| GhmiRnC | DW518567 | 3.0 | 5.09 | Cleavage | 3.00E-12 | predicted protein [Populus trichocarpa] |
|  |  |  |  |  | 2.00E-09 | translin associated factor X, putative [Ricinus communis] |
|  |  |  |  |  | 2.00E-09 | unnamed protein product [Vitis vinifera] |
| GhmiRnC | TC259543 | 2.5 | 17.93 | Cleavage | 3.00E-73 | PREDICTED: hypothetical protein [Vitis vinifera] |
|  |  |  |  |  | 2.00E-72 | PREDICTED: hypothetical protein isoform 2 [Vitis vinifera] |
|  |  |  |  |  | 3.00E-72 | calmodulin binding protein, putative [Ricinus communis] |
| GhmiRnC | EX168945 | 3.0 | 19.42 | Cleavage | 1.00E-38 | unnamed protein product [Vitis vinifera] |
|  |  |  |  |  | 1.00E-38 | integral membrane family protein [Arabidopsis lyrata subsp. lyrata] |
|  |  |  |  |  | 1.00E-38 | unknown [Arabidopsis thaliana] |
| GhmiRnC | TC270208 | 2.5 | 12.19 | Cleavage | 6.00E-75 | uncharacterized protein [Arabidopsis thaliana] |
|  |  |  |  |  | 1.00E-74 | predicted protein [Populus trichocarpa] |
|  |  |  |  |  | 3.00E-73 | calmodulin binding protein, putative [Ricinus communis] |
| GhmiRnC | EV482668 | 2.0 | 11.87 | Cleavage | 2.00E-10 | predicted protein [Populus trichocarpa] |
|  |  |  |  |  | 8.00E-10 | predicted protein [Populus trichocarpa] |
|  |  |  |  |  | 1.00E-09 | glucose-6-phosphate transporter 1 [Arabidopsis lyrata subsp.lyrata]. |
| GhmiRnC | BQ409431 | 3.0 | 8.42 | Cleavage | 2.00E-18 | Glucose-6-phosphate/phosphate translocator 1, chloroplast precursor, putative [Ricinus communis]. |
|  |  |  |  |  | 1.00E-17 | pyruvate dehydrogenase alpha subunit [Gossypium hirsutum] |
|  |  |  |  |  | 1.00E-17 | putative pyruvate dehydrogenase E1 component alpha subunit [Triticum aestivum]. |
| GhmiRnC | ES837150 | 3.0 | 9.80 | Translation | 5.00E-35 | Os02g0739600 [Oryza sativa Japonica Group] |
|  |  |  |  |  | 9.00E-35 | hypothetical protein POPTRDRAFT_836962 [Populus trichocarpa] |
|  |  |  |  |  | 8.00E-34 | Beta-expansin 3 precursor, putative [Ricinus communis] |
| GhmiRnC | DR456714 | 3.0 | 11.45 | Cleavage | 8.00E-17 | predicted protein [Populus trichocarpa] |
|  |  |  |  |  | 3.00E-16 | predicted protein [Populus trichocarpa] |
|  |  |  |  |  | 5.00E-16 | type I inositol polyphosphate 5-phosphatase, putative [Ricinus communis]. |
| GhmiRnE | ES826978 | 1.5 | 7.08 | Cleavage | 1.00E-65 | predicted protein [Populus trichocarpa] |
|  |  |  |  |  | 1.00E-65 | predicted protein [Populus trichocarpa] |
|  |  |  |  |  | 2.00E-64 | PREDICTED: similar to cysteine proteinase inhibitor [Vitis vinifera] |
| GhmiRnE | TC248714 | 1.5 | 7.07 | Cleavage | 1.00E-56 | predicted protein [Populus trichocarpa] |
|  |  |  |  |  | 1.00E-56 | predicted protein [Populus trichocarpa] |
|  |  |  |  |  | 4.00E-56 | PREDICTED: similar to cysteine proteinase inhibitor [Vitis vinifera] |
| GhmiRnE | TC234336 | 2.0 | 20.67 | Cleavage | 0.00E+00 | unnamed protein product [Vitis vinifera] |
|  |  |  |  |  | 0.00E+00 | PREDICTED: hypothetical protein [Vitis vinifera] |
|  |  |  |  |  | 0.00E+00 | kinase family protein [Arabidopsis lyrata subsp. lyrata] |
| GhmiRnE | TC244386 | 3.0 | 10.73 | Cleavage | 5.00E-144 | PREDICTED: hypothetical protein [Vitis vinifera] |
|  |  |  |  |  | 4.00E-142 | uncharacterized protein [Arabidopsis thaliana] |
|  |  |  |  |  | 4.00E-142 | hypothetical protein ARALYDRAFT_484344 [Arabidopsis lyrata subsp.lyrata]. |
| GhmiRnE | TC229767 | 3.0 | 14.04 | Translation | 0.00E+00 | Acetyle-CoA carboxylase beta subunit [Gossypium barbadense] |
|  |  |  |  |  | 0.00E+00 | acetyl-CoA carboxylase carboxyltransferase beta subunit [Gossypium thurberi]. |
|  |  |  |  |  | 0.00E+00 | acetyl-CoA carboxylase carboxyltransferase beta subunit [Gossypium thurberi]. |
| GhmiRnE | TC252382 | 3.0 | 13.42 | Cleavage | 2.00E-138 | conserved hypothetical protein [Ricinus communis] |
|  |  |  |  |  | 2.00E-135 | predicted protein [Populus trichocarpa] |
|  |  |  |  |  | 4.00E-134 | unnamed protein product [Vitis vinifera] |
| GhmiRnE | TC237889 | 3.0 | 14.04 | Translation | 3.00E-20 | ribulose-1,5-bisphosphate carboxylase/oxygenase large subunit [Phoenix canariensis]. |
|  |  |  |  |  | 5.00E-20 | ribulose-1,5-bisphosphate carboxylase/oxygenase large subunit [Cajanus cajan]. |
|  |  |  |  |  | 5.00E-20 | ribulose-1,5-bisphosphate carboxylase/oxygenase large subunit [Arachis hypogaea]. |
| GhmiRnE | AI729515 | 2.5 | 12.38 | Translation | 3.00E-43 | Cyclic nucleotide-gated ion channel, putative [Ricinus communis] |
|  |  |  |  |  | 3.00E-40 | predicted protein [Populus trichocarpa] |
|  |  |  |  |  | 1.00E-32 | unnamed protein product [Vitis vinifera] |
| GhmiRnE | BQ407568 | 2.5 | 11.34 | Translation | 2.00E-76 | Cyclic nucleotide-gated ion channel, putative [Ricinus communis] |
|  |  |  |  |  | 6.00E-73 | predicted protein [Populus trichocarpa] |
|  |  |  |  |  | 1.00E-64 | unnamed protein product [Vitis vinifera] |
| GhmiRnE | CO124962 | 3.0 | 15.25 | Cleavage | 3.00E-24 | predicted protein [Populus trichocarpa] |
|  |  |  |  |  | 6.00E-24 | cancer-associatedprotein protein, putative [Ricinus communis] |
|  |  |  |  |  | 1.00E-23 | PREDICTED: hypothetical protein [Vitis vinifera] |
| GhmiRnE | ES820530 | 3.0 | 12.88 | Cleavage | 1.00E-62 | phosphoprotein phosphatase, putative [Ricinus communis] |
|  |  |  |  |  | 2.00E-60 | Disease resistance protein RPS5, putative [Ricinus communis] |
|  |  |  |  |  | 1.00E-57 | cc-nbs-lrr resistance protein [Populus trichocarpa] |
| GhmiRnE | TC254895 | 2.5 | 19.08 | Cleavage | 2.00E-136 | NAC domain protein, IPR003441 [Populus trichocarpa] |
|  |  |  |  |  | 2.00E-133 | PREDICTED: hypothetical protein [Vitis vinifera] |
|  |  |  |  |  | 4.00E-133 | unnamed protein product [Vitis vinifera] |
| GhmiRnE | DW504606 | 3.0 | 15.74 | Cleavage | 3.00E-67 | WD-repeat protein, putative [Ricinus communis] |
|  |  |  |  |  | 6.00E-67 | PREDICTED: hypothetical protein [Vitis vinifera] |
|  |  |  |  |  | 8.00E-67 | unnamed protein product [Vitis vinifera] |
| GhmiRnE | DR452640 | 3.0 | 15.74 | Cleavage | 4.00E-66 | PREDICTED: hypothetical protein [Vitis vinifera] |
|  |  |  |  |  | 5.00E-66 | unnamed protein product [Vitis vinifera] |
|  |  |  |  |  | 9.00E-66 | WD-repeat protein, putative [Ricinus communis] |
| GhmiRnE | TC253320 | 3.0 | 8.62 | Cleavage | 4.00E-28 | predicted protein [Populus trichocarpa] |
|  |  |  |  |  | 5.00E-28 | unnamed protein product [Vitis vinifera] |
|  |  |  |  |  | 5.00E-28 | PREDICTED: hypothetical protein [Vitis vinifera] |
| GhmiRnE | ES837344 | 3.0 | 6.14 | Cleavage | 1.00E-28 | predicted protein [Populus trichocarpa] |
|  |  |  |  |  | 2.00E-28 | unnamed protein product [Vitis vinifera] |
|  |  |  |  |  | 2.00E-28 | PREDICTED: hypothetical protein [Vitis vinifera] |
| GhmiRnE | TC236427 | 3.0 | 9.62 | Translation | 3.00E-54 | HB03p [Malus floribunda] |
|  |  |  |  |  | 3.00E-54 | serine/threonine-protein kinase bri1, putative [Ricinus communis] |
|  |  |  |  |  | 1.00E-53 | HB09p [Malus floribunda] |
| GhmiRnF | TC265567 | 0.0 | 24.99 | Cleavage | 5.70E-01 | F23N19.5 [Arabidopsis thaliana] |
|  |  |  |  |  |  |  |
|  |  |  |  |  |  |  |
| GhmiRnF | TC243156 | 3.0 | 14.20 | Cleavage | 2.00E-86 | PREDICTED: uncharacterized protein LOC100499984 [Glycine max] |
|  |  |  |  |  | 3.00E-85 | hypothetical protein MTR_5g043680 [Medicago truncatula] |
|  |  |  |  |  | 4.00E-84 | PREDICTED: uncharacterized protein LOC100796991 [Glycine max] |
| GhmiRnF | TC232571 | 3.0 | 14.79 | Cleavage | 2.00E-110 | PREDICTED: uncharacterized protein LOC100255052 [Vitis vinifera] |
|  |  |  |  |  | 2.00E-99 | unnamed protein product [Vitis vinifera] |
|  |  |  |  |  | 3.00E-90 | PREDICTED: uncharacterized protein LOC100784670 [Glycine max] |
| GhmiRnH | TC231288 | 3.0 | 15.00 | Cleavage | 1.00E-151 | unnamed protein product [Vitis vinifera] |
|  |  |  |  |  | 2.00E-151 | PREDICTED: serine/threonine-protein kinase atg-1 [Vitis vinifera] |
|  |  |  |  |  | 3.00E-150 | predicted protein [Populus trichocarpa] |
| GhmiRnJ | TC256245 | 3.0 | 12.31 | Cleavage | 1.00E-36 | protein transporter, putative [Ricinus communis] |
|  |  |  |  |  | 3.00E-34 | predicted protein [Populus trichocarpa] |
|  |  |  |  |  | 9.00E-32 | unnamed protein product [Vitis vinifera] |
| GhmiRnJ | DW502333 | 3.0 | 12.31 | Cleavage | 3.00E-33 | protein transporter, putative [Ricinus communis] |
|  |  |  |  |  | 5.00E-29 | predicted protein [Populus trichocarpa] |
|  |  |  |  |  | 3.00E-28 | unnamed protein product [Vitis vinifera] |
| GhmiRnK | DR457633 | 3.0 | 12.41 | Cleavage | 6.00E-63 | hydrolase, putative [Ricinus communis] |
|  |  |  |  |  | 1.00E-59 | PREDICTED: uncharacterized protein LOC100242968 [Vitis vinifera] |
|  |  |  |  |  | 8.00E-58 | predicted protein [Populus trichocarpa] |
| GhmiRnK | TC242069 | 3.0 | 21.78 | Cleavage | 4.00E-26 | predicted protein [Populus trichocarpa] > |
|  |  |  |  |  | 4.00E-23 | hypothetical protein RCOM_1341690 [Ricinus communis] > |
|  |  |  |  |  | 3.00E-21 | predicted protein [Populus trichocarpa] |
| GhmiRnL | BF279166 | 0.5 | 16.76 | Cleavage | 3.60E-01 | RNA polymerase beta chain [Galium aparine] |
|  |  |  |  |  | 1.20E+00 | hypothetical protein OsI_36128 [Oryza sativa Indica Group] |
|  |  |  |  |  | 4.40E+00 | WRKY6-1 transcription factor [Brassica napus] |
| GhmiRnL | ES794311 | 0.5 | 19.25 | Cleavage | 1.60E+00 | maturase K (chloroplast) [Megastachya mucronata] |
| GhmiRnL | TC280043 | 2.5 | 16.25 | Cleavage | 0.00E+00 | Protein SE, putative [Ricinus communis] |
|  |  |  |  |  | 1.00E-175 | predicted protein [Populus trichocarpa] |
|  |  |  |  |  | 1.00E-171 | root hair defective 3 GTP-binding family protein [Arabidopsis lyrata subsp. lyrata] |
| GhmiRnL | TC271586 | 2.0 | 24.55 | Cleavage | 0.00E+00 | vacuolar H+-translocating inorganic pyrophosphatase [Populus trichocarpa] |
|  |  |  |  |  | 0.00E+00 | PREDICTED: pyrophosphate-energized vacuolar membrane proton pump-like [Glycine max] |
|  |  |  |  |  | 0.00E+00 | proton-translocating inorganic pyrophosphatase [Cucurbita moschata] |
| GhmiRnL | BF271848 | 2.5 | 24.14 | Cleavage | 2.00E-37 | HyPRP2 [Gossypium hirsutum] |
|  |  |  |  |  | 6.00E-30 | predicted protein [Populus trichocarpa] |
|  |  |  |  |  | 2.00E-29 | bifunctional inhibitor/lipid-transfer protein/seed storage 2S albumin-like protein [Arabidopsis thaliana] |
| GhmiRnL | ES815397 | 2.5 | 19.72 | Cleavage | 5.00E-41 | unnamed protein product [Vitis vinifera] |
|  |  |  |  |  | 2.00E-39 | PREDICTED: uncharacterized protein At5g08430-like [Glycine max] |
|  |  |  |  |  | 9.00E-38 | hypothetical protein ZEAMMB73_317180 [Zea mays] |
| GhmiRnL | TC276845 | 3.0 | 15.05 | Cleavage | 0.00E+00 | Protein SE, putative [Ricinus communis] |
|  |  |  |  |  | 0.00E+00 | predicted protein [Populus trichocarpa] |
|  |  |  |  |  | 0.00E+00 | predicted protein [Populus trichocarpa] |
| GhmiRnL | TC251681 | 2.5 | 12.00 | Cleavage | 7.00E-65 | hypothetical protein VITISV_041989 [Vitis vinifera] |
|  |  |  |  |  | 7.00E-65 | PREDICTED: DEAD-box ATP-dependent RNA helicase 24 [Vitis vinifera] |
|  |  |  |  |  | 3.00E-60 | DEAD-box ATP-dependent RNA helicase [Medicago truncatula] |
| GhmiRnL | TC248315 | 2.5 | 7.87 | Cleavage | 5.00E-108 | predicted protein [Populus trichocarpa] |
|  |  |  |  |  | 2.00E-102 | PREDICTED: trihelix transcription factor GTL2-like [Vitis vinifera] |
|  |  |  |  |  | 3.00E-96 | transcription factor, putative [Ricinus communis] |
| GhmiRnL | TC242672 | 2.5 | 11.81 | Cleavage | 6.00E-115 | hypothetical protein VITISV_041989 [Vitis vinifera] |
|  |  |  |  |  | 7.00E-115 | PREDICTED: DEAD-box ATP-dependent RNA helicase 24 [Vitis vinifera] |
|  |  |  |  |  | 3.00E-108 | PREDICTED: DEAD-box ATP-dependent RNA helicase 24-like [Glycine max] |
| GhmiRnL | TC251004 | 2.5 | 7.88 | Cleavage | 5.00E-85 | predicted protein [Populus trichocarpa] |
|  |  |  |  |  | 6.00E-85 | uncharacterized protein LOC100305764 [Glycine max] |
|  |  |  |  |  | 2.00E-83 | unknown [Populus trichocarpa] |
| GhmiRnL | TC256329 | 2.5 | 21.69 | Cleavage | 0.00E+00 | unnamed protein product [Vitis vinifera] |
|  |  |  |  |  | 0.00E+00 | PREDICTED: tricalbin-1 [Vitis vinifera] |
|  |  |  |  |  | 0.00E+00 | PREDICTED: extended synaptotagmin-2-like [Glycine max] |
| GhmiRnL | TC278338 | 3.0 | 9.76 | Cleavage | 4.00E-06 | unnamed protein product [Vitis vinifera] |
|  |  |  |  |  | 5.00E-06 | PREDICTED: long-chain-fatty-acid--CoA ligase FadD15-like [Vitis vinifera] |
|  |  |  |  |  | 9.00E-06 | long-chain-fatty-acid CoA ligase, putative [Ricinus communis] |
| GhmiRnL | ES804250 | 2.5 | 11.63 | Translation | 1.00E-78 | gcn4-complementing protein, putative [Ricinus communis] |
|  |  |  |  |  | 8.00E-75 | PREDICTED: ADP-ribosylation factor GTPase-activating protein AGD3-like [Vitis vinifera] |
|  |  |  |  |  | 8.00E-75 | unnamed protein product [Vitis vinifera] |
| GhmiRnL | ES837476 | 3.0 | 12.48 | Cleavage | 5.00E-95 | PREDICTED: LOW QUALITY PROTEIN: activator of 90 kDa heat shock protein ATPase homolog 1-like [Glycine max] |
|  |  |  |  |  | 2.00E-94 | PREDICTED: activator of 90 kDa heat shock protein ATPase homolog 1 isoform 1 [Vitis vinifera] |
|  |  |  |  |  | 4.00E-94 | PREDICTED: activator of 90 kDa heat shock protein ATPase homolog |
| GhmiRnL | TC234906 | 3.0 | 21.55 | Cleavage | 1.00E-96 | suppressor of actin, putative [Ricinus communis] |
|  |  |  |  |  | 3.00E-95 | PREDICTED: phosphatidylinositide phosphatase SAC1-like isoform 1 [Vitis vinifera] |
|  |  |  |  |  | 4.00E-95 | hypothetical protein VITISV_027268 [Vitis vinifera] |
| GhmiRnL | ES805372 | 3.0 | 19.91 | Cleavage | 1.00E-151 | PREDICTED: phosphatidylinositide phosphatase SAC1-like isoform 1 [Vitis vinifera] |
|  |  |  |  |  | 4.00E-147 | unnamed protein product [Vitis vinifera] |
|  |  |  |  |  | 8.00E-146 | AT3G51830 [Arabidopsis thaliana] |
| GhmiRnL | DW479618 | 3.0 | 22.89 | Cleavage | 3.30E+00 | hypothetical protein ZEAMMB73_305630, partial [Zea mays] |
|  |  |  |  |  | 5.10E+00 | hypothetical protein LOC_Os03g12750 [Oryza sativa Japonica Group] |
| GhmiRnL | TC236134 | 2.0 | 24.28 | Translation | 6.00E-13 | RRM-containing protein [Citrus unshiu] |
|  |  |  |  |  | 6.00E-10 | predicted protein [Populus trichocarpa] |
|  |  |  |  |  | 4.00E-09 | PREDICTED: uncharacterized protein LOC100257637 isoform 2 [Vitis vinifera] |
| GhmiRnL | ES817982 | 3.0 | 22.89 | Cleavage | 6.00E+00 | hypothetical protein RCOM_0447050 [Ricinus communis] |
