# Supplementary material for: Genome-wide analysis of small RNAs reveals eight fiber elongation-related and 257 novel microRNAs in elongating cotton fiber cells
Source: BMC Genomics. 2013 Sep 17;14:629. doi: 10.1186/1471-2164-14-629 (PMC3849097; doi:10.1186/1471-2164-14-629)
Supplement: Additional file 11: Table S5 — Target prediction for known cotton fiber elongation-related miRNAs. [file 1471-2164-14-629-S11.docx]

**Additional Table S5**:

**Target prediction for known cotton fiber elongation-related miRNAs**

| **miRNA** | **Predicted target** | **Mismatch** | **UPE** | **Inhibition** | **E-value** | **Target gene annotation** |
| --- | --- | --- | --- | --- | --- | --- |
| miR1310 | TC270887 | 0.0 | 24.03 | Cleavage | 3.00E-31 | hypothetical protein SORBIDRAFT_1292s002010 [Sorghum bicolor] |
|  |  |  |  |  | 4.00E-30 | hypothetical protein MTR_5g051120 [Medicago truncatula] |
|  |  |  |  |  | 2.00E-29 | ATP synthase subunit beta [Medicago truncatula] |
| miR1310 | TC274430 | 0.0 | 21.90 | Cleavage | 3.00E-32 | hypothetical protein MTR_5g051030 [Medicago truncatula] |
|  |  |  |  |  | 4.00E-32 | hypothetical protein MTR_5g050970 [Medicago truncatula] |
|  |  |  |  |  | 5.00E-32 | hypothetical protein MTR_132s0010, partial [Medicago truncatula] |
| miR1310 | TC267000 | 0.0 | 24.03 | Cleavage | 5.00E-46 | hypothetical protein MTR_5g051170 [Medicago truncatula] |
|  |  |  |  |  | 2.00E-35 | hypothetical protein MTR_5g051030 [Medicago truncatula] |
|  |  |  |  |  | 2.00E-35 | hypothetical protein MTR_5g051130 [Medicago truncatula] |
| miR1310 | TC235341 | 0.0 | 24.03 | Cleavage | 6.00E-89 | hypothetical protein MTR_5g051170 [Medicago truncatula] |
|  |  |  |  |  | 2.00E-62 | hypothetical protein MTR_5g050970 [Medicago truncatula] |
|  |  |  |  |  | 2.00E-61 | hypothetical protein MTR_132s0010, partial [Medicago truncatula] |
| miR1310 | TC251692 | 0.0 | 24.03 | Cleavage | 1.00E-90 | hypothetical protein MTR_5g051170 [Medicago truncatula] |
|  |  |  |  |  | 4.00E-53 | hypothetical protein MTR_132s0010, partial [Medicago truncatula] |
|  |  |  |  |  | 7.00E-53 | hypothetical protein MTR_5g051140 [Medicago truncatula] |
| miR1310 | TC275327 | 0.0 | 24.87 | Cleavage | 2.00E-41 | hypothetical protein SORBIDRAFT_1368s002010 [Sorghum bicolor] |
|  |  |  |  |  | 3.00E-40 | hypothetical protein MTR_5g051170 [Medicago truncatula] |
|  |  |  |  |  | 8.00E-37 | hypothetical protein SORBIDRAFT_0070s002020 [Sorghum bicolor] |
| miR156 | DR462234 | 1.0 | 16.56 | Cleavage | 2.00E-56 | LIGULELESS1 protein, putative [Ricinus communis] |
|  |  |  |  |  | 1.00E-50 | PREDICTED: hypothetical protein [Vitis vinifera] |
|  |  |  |  |  | 1.00E-50 | promoter-binding protein SPL9 [Vitis vinifera] |
| miR156 | CO097437 | 1.0 | 17.96 | Cleavage | 1.00E-64 | LIGULELESS1 protein, putative [Ricinus communis] |
|  |  |  |  |  | 2.00E-64 | PREDICTED: hypothetical protein [Vitis vinifera] |
|  |  |  |  |  | 3.00E-64 | promoter-binding protein SPL9 [Vitis vinifera] |
| miR156 | TC272934 | 1.0 | 21.32 | Cleavage | 4.00E-66 | unnamed protein product [Vitis vinifera] |
|  |  |  |  |  | 8.00E-66 | promoter-binding protein SPL9 [Vitis vinifera] |
|  |  |  |  |  | 8.00E-66 | PREDICTED: promoter-binding protein SPL9 [Vitis vinifera] |
| miR156 | TC253516 | 1.0 | 17.56 | Cleavage | 6.00E-82 | LIGULELESS1 protein, putative [Ricinus communis] |
|  |  |  |  |  | 1.00E-80 | unnamed protein product [Vitis vinifera] |
|  |  |  |  |  | 3.00E-80 | promoter-binding protein SPL9 [Vitis vinifera] |
| miR156 | TC232339 | 1.0 | 13.53 | Cleavage | 3.00E-108 | LIGULELESS1 protein, putative [Ricinus communis] |
|  |  |  |  |  | 8.00E-102 | unnamed protein product [Vitis vinifera] |
|  |  |  |  |  | 3.00E-101 | PREDICTED: squamosa promoter-binding-like protein 12-like [Vitis vinifera] |
| miR156 | TC266384 | 1.0 | 9.68 | Cleavage | 1.00E-40 | hypothetical protein VITISV_001736 [Vitis vinifera] |
|  |  |  |  |  | 1.00E-40 | PREDICTED: squamosa promoter-binding-like protein 12-like [Vitis vinifera] |
|  |  |  |  |  | 1.00E-40 | hypothetical protein VITISV_001736 [Vitis vinifera] |
| miR156 | TC273000 | 1.5 | 17.02 | Cleavage | 3.00E-90 | LIGULELESS1 protein, putative [Ricinus communis] |
|  |  |  |  |  | 7.00E-87 | unnamed protein product [Vitis vinifera] |
|  |  |  |  |  | 1.00E-85 | PREDICTED: squamosa promoter-binding-like protein 6-like [Vitis vinifera] |
| miR156 | TC272692 | 1.5 | 21.04 | Cleavage | 2.00E-55 | unnamed protein product [Vitis vinifera] |
|  |  |  |  |  | 1.00E-54 | PREDICTED: squamosa promoter-binding-like protein 6-like [Vitis vinifera] |
|  |  |  |  |  | 8.00E-54 | LIGULELESS1 protein, putative [Ricinus communis] |
| miR156 | TC239557 | 1.0 | 5.59 | Cleavage | 7.00E-56 | Squamosa promoter-binding protein, putative [Ricinus communis] |
|  |  |  |  |  | 3.00E-53 | PREDICTED: squamosa promoter-binding protein 1 [Vitis vinifera] |
|  |  |  |  |  | 3.00E-52 | SPL3-like protein [Eucalyptus globulus] |
| miR156 | CO092899 | 2.0 | 9.98 | Cleavage | 2.00E-63 | conserved hypothetical protein [Ricinus communis] |
|  |  |  |  |  | 7.00E-62 | PREDICTED: hypothetical protein [Vitis vinifera] |
|  |  |  |  |  | 5.00E-56 | unnamed protein product [Vitis vinifera] |
| miR156 | TC252495 | 2.0 | 17.99 | Cleavage | 1.00E-10 | hypothetical protein POPTRDRAFT_733659 [Populus trichocarpa] |
|  |  |  |  |  | 5.00E-09 | squamosa promoter-binding protein [Citrus trifoliata] |
|  |  |  |  |  | 2.00E-08 | SPL domain class transcription factor [Malus x domestica] |
| miR156 | TC276834 | 2.0 | 15.33 | Cleavage | 2.00E-111 | hypothetical protein POPTRDRAFT_755123 [Populus trichocarpa] |
|  |  |  |  |  | 5.00E-110 | unnamed protein product [Vitis vinifera] |
|  |  |  |  |  | 3.00E-109 | PREDICTED: squamosa promoter-binding-like protein 7-like [Vitis vinifera] |
| miR156 | TC246550 | 2.0 | 13.74 | Cleavage | 4.00E-91 | PREDICTED: squamosa promoter-binding-like protein 6-like [Vitis vinifera] |
|  |  |  |  |  | 8.00E-77 | PREDICTED: uncharacterized protein LOC100777766 [Glycine max] |
|  |  |  |  |  | 3.00E-76 | LIGULELESS1 protein, putative [Ricinus communis] |
| miR156 | TC264623 | 1.0 | 5.77 | Cleavage | 5.00E-54 | Squamosa promoter-binding protein, putative [Ricinus communis] |
|  |  |  |  |  | 2.00E-50 | PREDICTED: squamosa promoter-binding protein 1 [Vitis vinifera] |
|  |  |  |  |  | 2.00E-49 | SPL3-like protein [Eucalyptus globulus] |
| miR156 | DW511492 | 2.0 | 21.38 | Cleavage | 5.00E-54 | predicted protein [Populus trichocarpa] |
|  |  |  |  |  | 6.00E-52 | predicted protein [Populus trichocarpa] |
|  |  |  |  |  | 2.00E-48 | protein phosphatase pp2a regulatory subunit B, putative [Ricinus communis] |
| miR156 | GR708165 | 2.5 | 22.50 | Cleavage | 4.00E-130 | predicted protein [Populus trichocarpa] |
|  |  |  |  |  | 2.00E-126 | predicted protein [Populus trichocarpa] |
|  |  |  |  |  | 5.00E-126 | predicted protein [Populus trichocarpa] |
| miR156 | TC240338 | 2.5 | 15.58 | Cleavage | 3.00E-78 | LIGULELESS1 protein, putative [Ricinus communis] |
|  |  |  |  |  | 8.00E-78 | unnamed protein product [Vitis vinifera] |
|  |  |  |  |  | 2.00E-77 | promoter-binding protein SPL9 [Vitis vinifera] |
| miR156 | DW499966 | 2.0 | 23.83 | Cleavage | 3.00E-43 | squamosa promoter-binding protein [Citrus trifoliata] |
|  |  |  |  |  | 5.00E-41 | hypothetical protein POPTRDRAFT_733659 [Populus trichocarpa] |
|  |  |  |  |  | 7.00E-40 | SPL domain class transcription factor [Malus x domestica] |
| miR156 | TC278149 | 3.0 | 10.08 | Cleavage | 1.00E-105 | uncharacterized protein LOC100789838 [Glycine max] |
|  |  |  |  |  | 2.00E-105 | PREDICTED: basic leucine zipper and W2 domain-containing protein 2-like [Glycine max] |
|  |  |  |  |  | 7.00E-105 | Basic leucine zipper and W2 domain-containing protein [Medicago truncatula] |
| miR156 | TC245204 | 2.5 | 16.40 | Translation | 7.00E-145 | predicted protein [Populus trichocarpa] |
|  |  |  |  |  | 6.00E-142 | Triacylglycerol lipase 1 precursor, putative [Ricinus communis] |
|  |  |  |  |  | 2.00E-140 | ATLIP1 [Arabidopsis lyrata subsp. lyrata] |
| miR159/319 | TC279858 | 1.0 | 8.60 | Cleavage | 9.00E-06 | pentatricopeptide repeat-containing protein, putative [Ricinus communis] |
| miR159/319 | TC237076 | 2.0 | 8.10 | Cleavage | 4.00E-59 | PREDICTED: uncharacterized protein LOC100257710 [Vitis vinifera] |
|  |  |  |  |  | 5.00E-59 | conserved hypothetical protein [Ricinus communis] |
|  |  |  |  |  | 1.00E-58 | predicted protein [Populus trichocarpa] |
| miR159/319 | TC248481 | 3.0 | 10.60 | Cleavage | 2.00E-57 | hypothetical protein VITISV_040250 [Vitis vinifera] |
|  |  |  |  |  | 1.00E-56 | PREDICTED: pentatricopeptide repeat-containing protein At5g66520 [Vitis vinifera] |
|  |  |  |  |  | 1.00E-44 | unnamed protein product [Vitis vinifera] |
| miR159/319 | DT466704 | 2.5 | 9.79 | Translation | 4.00E-21 | uncharacterized protein LOC100381656 [Zea mays] |
|  |  |  |  |  | 5.00E-21 | PREDICTED: uncharacterized protein LOC100267308 [Vitis vinifera] |
|  |  |  |  |  | 5.00E-21 | conserved hypothetical protein [Ricinus communis] |
| miR159/319 | TC243661 | 2.5 | 17.04 | Translation | 4.00E-29 | predicted protein [Populus trichocarpa] |
|  |  |  |  |  | 1.00E-28 | f-box family protein [Populus trichocarpa] |
|  |  |  |  |  | 1.00E-25 | conserved hypothetical protein [Ricinus communis] |
| miR159/319 | CO121704 | 2.5 | 11.91 | Translation | 4.00E-23 | PREDICTED: uncharacterized protein LOC100267308 [Vitis vinifera] |
|  |  |  |  |  | 6.00E-22 | conserved hypothetical protein [Ricinus communis] |
|  |  |  |  |  | 2.00E-21 | unnamed protein product [Vitis vinifera] |
| miR159/319 | TC261353 | 3.0 | 7.32 | Cleavage | 3.00E-180 | Serine/threonine-protein kinase PBS1, putative [Ricinus communis] |
|  |  |  |  |  | 2.00E-172 | predicted protein [Populus trichocarpa] |
|  |  |  |  |  | 5.00E-168 | PREDICTED: protein STRUBBELIG-RECEPTOR FAMILY 8-like [Vitis vinifera] |
| miR159/319 | DN803059 | 3.0 | 12.95 | Cleavage | 4.00E-05 | conserved hypothetical protein [Ricinus communis] |
|  |  |  |  |  | 6.00E-05 | putative cullin-like 1 protein [Arabidopsis thaliana] |
|  |  |  |  |  | 6.00E-05 | cullin 1 [Arabidopsis thaliana] |
| miR159/319 | EV486340 | 2.5 | 17.30 | Cleavage | 5.00E-84 | PREDICTED: 3-ketoacyl-CoA synthase 11-like [Glycine max] |
|  |  |  |  |  | 9.00E-84 | PREDICTED: 3-ketoacyl-CoA synthase 11-like [Glycine max] |
|  |  |  |  |  | 8.00E-83 | 3-ketoacyl-CoA synthase 17 [Arabidopsis thaliana] |
| miR159/319 | TC231683 | 2.5 | 14.05 | Cleavage | 6.00E-130 | PREDICTED: uncharacterized protein ywbO [Vitis vinifera] |
|  |  |  |  |  | 2.00E-129 | unnamed protein product [Vitis vinifera] |
|  |  |  |  |  | 3.00E-128 | predicted protein [Populus trichocarpa] |
| miR159/319 | TC235328 | 3.0 | 15.35 | Cleavage | 6.00E-150 | PREDICTED: EID1-like F-box protein 2-like [Vitis vinifera] |
|  |  |  |  |  | 6.00E-150 | hypothetical protein VITISV_009953 [Vitis vinifera] |
|  |  |  |  |  | 1.00E-148 | conserved hypothetical protein [Ricinus communis] |
| miR164 | CO109521 | 1.5 | 13.37 | Cleavage | 4.00E-113 | NAC domain protein, IPR003441 [Populus trichocarpa] |
|  |  |  |  |  | 2.00E-112 | NAC domain protein [Medicago truncatula] |
|  |  |  |  |  | 5.00E-111 | transcriptional factor NAC35 [Glycine max] |
| miR164 | TC253804 | 3.0 | 12.89 | Cleavage | 5.00E-87 | Protein grpE, putative [Ricinus communis] |
|  |  |  |  |  | 1.00E-86 | PREDICTED: protein grpE-like [Vitis vinifera] |
|  |  |  |  |  | 4.00E-85 | hypothetical protein VITISV_018795 [Vitis vinifera] |
| miR164 | TC241215 | 3.0 | 11.71 | Cleavage | 3.00E-120 | Protein grpE, putative [Ricinus communis] |
|  |  |  |  |  | 5.00E-116 | PREDICTED: protein grpE-like [Vitis vinifera] |
|  |  |  |  |  | 2.00E-111 | hypothetical protein VITISV_018795 [Vitis vinifera] |
| miR164 | TC274274 | 3.0 | 12.23 | Cleavage | 2.00E-100 | PREDICTED: ubiquitin-conjugating enzyme E2 7-like [Glycine max] |
|  |  |  |  |  | 2.00E-100 | PREDICTED: ubiquitin-conjugating enzyme E2 7 isoform 1 [Vitis vinifera] |
|  |  |  |  |  | 3.00E-100 | uncharacterized protein LOC100500474 [Glycine max] |
| miR164 | DT465455 | 3.0 | 11.80 | Cleavage | 4.00E-156 | UDP-glucuronic acid decarboxylase 2 [Gossypium hirsutum] |
|  |  |  |  |  | 6.00E-115 | PREDICTED: UDP-glucuronic acid decarboxylase 1-like [Vitis vinifera] |
|  |  |  |  |  | 1.00E-109 | PREDICTED: UDP-glucuronic acid decarboxylase 1-like [Glycine max] |
| miR164 | TC240488 | 3.0 | 13.06 | Cleavage | 0.00E+00 | UDP-glucuronic acid decarboxylase 2 [Gossypium hirsutum] |
|  |  |  |  |  | 2.00E-164 | PREDICTED: UDP-glucuronic acid decarboxylase 1-like [Vitis vinifera] |
|  |  |  |  |  | 6.00E-163 | putative UDP-glucuronate decarboxylase 3 [Nicotiana tabacum] |
| miR164 | TC233169 | 3.0 | 13.06 | Cleavage | 0.00E+00 | UDP-glucuronic acid decarboxylase 2 [Gossypium hirsutum] |
|  |  |  |  |  | 0.00E+00 | PREDICTED: UDP-glucuronic acid decarboxylase 1-like [Glycine max] |
|  |  |  |  |  | 0.00E+00 | PREDICTED: UDP-glucuronic acid decarboxylase 1-like [Glycine max] |
| miR164 | TC236955 | 3.0 | 13.06 | Cleavage | 0.00E+00 | UDP-glucuronic acid decarboxylase 2 [Gossypium hirsutum] |
|  |  |  |  |  | 6.00E-146 | PREDICTED: UDP-glucuronic acid decarboxylase 1-like [Vitis vinifera] |
|  |  |  |  |  | 3.00E-141 | PREDICTED: UDP-glucuronic acid decarboxylase 1-like [Glycine max] |
| miR165/166 | ES808067 | 1.5 | 24.37 | Cleavage | 0.00E+00 | PREDICTED: homeobox-leucine zipper protein ATHB-15-like isoform 2 [Vitis vinifera] |
|  |  |  |  |  | 0.00E+00 | unnamed protein product [Vitis vinifera] |
|  |  |  |  |  | 0.00E+00 | PREDICTED: homeobox-leucine zipper protein ATHB-15-like isoform 1 [Vitis vinifera] |
| miR165/166 | ES810681 | 1.5 | 24.37 | Cleavage | 0.00E+00 | PREDICTED: homeobox-leucine zipper protein ATHB-15-like isoform 2 [Vitis vinifera] |
|  |  |  |  |  | 0.00E+00 | unnamed protein product [Vitis vinifera] |
|  |  |  |  |  | 0.00E+00 | PREDICTED: homeobox-leucine zipper protein ATHB-15-like isoform 1 [Vitis vinifera] |
| miR165/166 | TC279526 | 2.0 | 25.51 | Cleavage | 0.00E+00 | class III HD-Zip protein 8 [Prunus persica] |
|  |  |  |  |  | 0.00E+00 | putative HB8 HD-ZipIII [Malus x domestica] |
|  |  |  |  |  | 0.00E+00 | PREDICTED: homeobox-leucine zipper protein ATHB-15-like [Vitis vinifera] |
| miR165/166 | TC257490 | 2.0 | 22.46 | Cleavage | 7.00E-57 | hypothetical protein VITISV_018803 [Vitis vinifera] |
|  |  |  |  |  | 7.00E-57 | PREDICTED: homeobox-leucine zipper protein ATHB-15-like isoform 3 [Vitis vinifera] |
|  |  |  |  |  | 7.00E-57 | PREDICTED: homeobox-leucine zipper protein ATHB-15-like isoform 1 [Vitis vinifera] |
| miR165/166 | TC267202 | 2.0 | 19.39 | Cleavage | 0.00E+00 | conserved hypothetical protein [Ricinus communis] |
|  |  |  |  |  | 0.00E+00 | PREDICTED: homeobox-leucine zipper protein HOX32-like isoform 3 [Vitis vinifera] |
|  |  |  |  |  | 0.00E+00 | PREDICTED: homeobox-leucine zipper protein HOX32-like isoform 1 [Vitis vinifera] |
| miR165/166 | GR716471 | 2.5 | 16.24 | Cleavage |  | none |
| miR165/166 | TC240051 | 2.5 | 22.75 | Cleavage | 6.00E-110 | PREDICTED: homeobox-leucine zipper protein ATHB-15-like isoform 2 [Vitis vinifera] |
|  |  |  |  |  | 7.00E-110 | unnamed protein product [Vitis vinifera] |
|  |  |  |  |  | 7.00E-110 | PREDICTED: homeobox-leucine zipper protein ATHB-15-like isoform 1 [Vitis vinifera] |
| miR165/166 | ES832414 | 2.5 | 22.75 | Cleavage | 2.00E-98 | PREDICTED: homeobox-leucine zipper protein ATHB-15-like isoform 2 [Vitis vinifera] |
|  |  |  |  |  | 2.00E-98 | hypothetical protein VITISV_018803 [Vitis vinifera] |
|  |  |  |  |  | 2.00E-98 | unnamed protein product [Vitis vinifera] |
| miR165/166 | TC259656 | 3.0 | 21.75 | Cleavage | 2.00E-132 | PREDICTED: histone-lysine N-methyltransferase setd3 [Vitis vinifera] |
|  |  |  |  |  | 1.00E-129 | conserved hypothetical protein [Ricinus communis] |
|  |  |  |  |  | 2.00E-124 | PREDICTED: histone-lysine N-methyltransferase setd3-like [Glycine max] |
| miR165/166 | DR462038 | 3.0 | 20.73 | Cleavage | 5.00E-82 | PREDICTED: pentatricopeptide repeat-containing protein At1g71210-like [Vitis vinifera] |
|  |  |  |  |  | 8.00E-81 | hypothetical protein VITISV_028175 [Vitis vinifera] |
|  |  |  |  |  | 3.00E-78 | pentatricopeptide repeat-containing protein, putative [Ricinus communis] |
| miR165/166 | TC258729 | 3.0 | 19.29 | Cleavage | 1.00E-158 | PREDICTED: ribonucleoside-diphosphate reductase small chain A [Vitis vinifera] |
|  |  |  |  |  | 1.00E-157 | ribonucleoside-diphosphate reductase small chain, putative [Ricinus communis] |
|  |  |  |  |  | 5.00E-157 | predicted protein [Populus trichocarpa] |
| miR165/166 | TC245058 | 3.0 | 16.29 | Cleavage | 7.00E-180 | PREDICTED: histone-lysine N-methyltransferase setd3 [Vitis vinifera] |
|  |  |  |  |  | 9.00E-178 | conserved hypothetical protein [Ricinus communis] |
|  |  |  |  |  | 2.00E-170 | PREDICTED: LOW QUALITY PROTEIN: histone-lysine N-methyltransferase setd3-like [Glycine max] |
| miR165/166 | TC230556 | 3.0 | 19.29 | Cleavage | 0.00E+00 | ribonucleoside-diphosphate reductase small chain, putative [Ricinus communis] |
|  |  |  |  |  | 0.00E+00 | PREDICTED: ribonucleoside-diphosphate reductase small chain A [Vitis vinifera] |
|  |  |  |  |  | 0.00E+00 | predicted protein [Populus trichocarpa] |
| miR167 | AW730640 | 2.5 | 14.67 | Translation |  |  |
|  |  |  |  |  |  |  |
|  |  |  |  |  |  |  |
| miR167 | DW226555 | 3.0 | 12.39 | Cleavage | 6.00E-46 | LIM domain-containing protein [Medicago truncatula] |
|  |  |  |  |  | 2.00E-45 | LIM domain-containing protein [Medicago truncatula] |
|  |  |  |  |  | 5.00E-45 | uncharacterized protein LOC100809121 [Glycine max] |
| miR167 | DW478879 | 3.0 | 16.48 | Cleavage | 2.00E-136 | LIM domain protein [Gossypium hirsutum] |
|  |  |  |  |  | 6.00E-134 | LIM1 [Hevea brasiliensis] |
|  |  |  |  |  | 6.00E-134 | Pollen-specific protein SF3, putative [Ricinus communis] |
| miR167 | TC267958 | 3.0 | 17.22 | Cleavage | 2.00E-119 | Os07g0574800 [Oryza sativa Japonica Group] |
|  |  |  |  |  | 1.00E-116 | alpha-tubulin [Gossypium hirsutum] |
|  |  |  |  |  | 1.00E-116 | alpha-tubulin [Gossypium hirsutum] |
| miR167 | ES851036 | 3.0 | 15.35 | Translation | 2.00E-61 | unnamed protein product [Vitis vinifera] |
|  |  |  |  |  | 1.00E-60 | conserved hypothetical protein [Ricinus communis] |
|  |  |  |  |  | 2.00E-60 | PREDICTED: protein MID1-COMPLEMENTING ACTIVITY 1-like [Vitis vinifera] |
| miR167 | ES831296 | 3.5 | 23.13 | Cleavage | 1.00E-41 | predicted protein [Populus trichocarpa] |
|  |  |  |  |  | 3.00E-41 | PREDICTED: auxin response factor 6-like [Vitis vinifera] |
|  |  |  |  |  | 3.00E-40 | predicted protein [Populus trichocarpa] |
| miR167 | ES814902 | 3.5 | 19.59 | Cleavage | 2.00E-139 | PREDICTED: auxin response factor 6-like [Vitis vinifera] |
|  |  |  |  |  | 4.00E-134 | predicted protein [Populus trichocarpa] |
|  |  |  |  |  | 5.00E-134 | predicted protein [Populus trichocarpa] |
| miR167 | TC256045 | 3.5 | 24.66 | Cleavage | 4.00E-94 | predicted protein [Populus trichocarpa] |
|  |  |  |  |  | 3.00E-93 | predicted protein [Populus trichocarpa] |
|  |  |  |  |  | 2.00E-91 | unnamed protein product [Vitis vinifera] |
| miR167 | TC270505 | 3.5 | 24.66 | Cleavage | 2.00E-164 | PREDICTED: auxin response factor 6-like [Vitis vinifera] |
|  |  |  |  |  | 8.00E-164 | predicted protein [Populus trichocarpa] |
|  |  |  |  |  | 9.00E-157 | ARF domain class transcription factor [Malus x domestica] |
| miR167 | TC239291 | 3.5 | 22.22 | Cleavage | 6.00E-161 | predicted protein [Populus trichocarpa] |
|  |  |  |  |  | 2.00E-160 | PREDICTED: auxin response factor 6-like [Vitis vinifera] |
|  |  |  |  |  | 1.00E-156 | predicted protein [Populus trichocarpa] |
| miR167 | TC241907 | 3.5 | 24.95 | Cleavage | 5.00E-102 | PREDICTED: auxin response factor 8-like [Vitis vinifera] |
|  |  |  |  |  | 4.00E-100 | unknown [Populus trichocarpa] |
|  |  |  |  |  | 7.00E-100 | PREDICTED: auxin response factor 8-like [Glycine max] |
| miR167 | TC242629 | 3.5 | 20.24 | Cleavage | 9.00E-51 | PREDICTED: uncharacterized protein LOC100243457 [Vitis vinifera] |
|  |  |  |  |  | 4.00E-50 | hypothetical protein VITISV_017367 [Vitis vinifera] |
|  |  |  |  |  | 3.00E-49 | predicted protein [Populus trichocarpa] |
| miR167 | AI729255 | 3.5 | 20.23 | Cleavage | 5.00E-43 | PREDICTED: uncharacterized protein LOC100243457 [Vitis vinifera] |
|  |  |  |  |  | 2.00E-42 | predicted protein [Populus trichocarpa] |
|  |  |  |  |  | 2.00E-42 | hypothetical protein VITISV_017367 [Vitis vinifera] |
| miR167 | GR714818 | 3.5 | 20.24 | Cleavage | 3.00E-51 | PREDICTED: uncharacterized protein LOC100243457 [Vitis vinifera] |
|  |  |  |  |  | 1.00E-50 | hypothetical protein VITISV_017367 [Vitis vinifera] |
|  |  |  |  |  | 1.00E-49 | predicted protein [Populus trichocarpa] |
| miR167 | TC260930 | 3.5 | 20.03 | Cleavage | 6.00E-99 | PREDICTED: auxin response factor 6-like [Vitis vinifera] |
|  |  |  |  |  | 6.00E-94 | PREDICTED: auxin response factor 6-like [Glycine max] |
|  |  |  |  |  | 1.00E-93 | PREDICTED: auxin response factor 6-like [Glycine max] |
| miR167 | CO119630 | 3.5 | 19.00 | Cleavage | 1.00E-31 | conserved hypothetical protein [Ricinus communis] |
|  |  |  |  |  | 9.00E-31 | conserved hypothetical protein [Ricinus communis] |
|  |  |  |  |  | 2.00E-30 | uncharacterized protein [Arabidopsis thaliana] |
| miR168 | TC241943 | 3.0 | 13.49 | Translation | 2.00E-134 | glucose-methanol-choline (gmc) oxidoreductase, putative [Ricinus communis] |
|  |  |  |  |  | 1.00E-127 | predicted protein [Populus trichocarpa] |
|  |  |  |  |  | 6.00E-125 | unnamed protein product [Vitis vinifera] |
| miR168 | CO084924 | 2.5 | 8.76 | Cleavage | 3.00E-163 | eukaryotic translation initiation factor 2c, putative [Ricinus communis] |
|  |  |  |  |  | 1.00E-160 | AGO1-1 [Nicotiana benthamiana] |
|  |  |  |  |  | 1.00E-158 | argonaute protein group [Populus trichocarpa] |
| miR168 | TC280219 | 3.0 | 17.40 | Translation | 1.00E-119 | PREDICTED: DEAD-box ATP-dependent RNA helicase 16-like [Glycine max] |
|  |  |  |  |  | 1.00E-111 | dead box ATP-dependent RNA helicase, putative [Ricinus communis] |
|  |  |  |  |  | 7.00E-111 | hypothetical protein ARALYDRAFT_491167 [Arabidopsis lyrata subsp. lyrata] |
| miR172 | TC249346 | 0.0 | 19.45 | Cleavage | 4.00E-35 | Protein AINTEGUMENTA, putative [Ricinus communis] |
|  |  |  |  |  | 5.00E-35 | PREDICTED: ethylene-responsive transcription factor RAP2-7-like [Vitis vinifera] |
|  |  |  |  |  | 6.00E-35 | unnamed protein product [Vitis vinifera] |
| miR172 | TC275039 | 0.0 | 19.46 | Cleavage | 1.00E-81 | unnamed protein product [Vitis vinifera] |
|  |  |  |  |  | 2.00E-81 | PREDICTED: ethylene-responsive transcription factor RAP2-7-like [Vitis vinifera] |
|  |  |  |  |  | 1.00E-77 | Protein AINTEGUMENTA, putative [Ricinus communis] |
| miR172 | DW225324 | 0.0 | 11.50 | Cleavage | 7.00E-38 | PREDICTED: ethylene-responsive transcription factor RAP2-7-like [Vitis vinifera] |
|  |  |  |  |  | 7.00E-38 | Protein AINTEGUMENTA, putative [Ricinus communis] |
|  |  |  |  |  | 7.00E-38 | unnamed protein product [Vitis vinifera] |
| miR172 | TC276431 | 1.0 | 17.30 | Cleavage | 3.00E-68 | unnamed protein product [Vitis vinifera] |
|  |  |  |  |  | 9.00E-68 | APETALA2 [Betula platyphylla] |
|  |  |  |  |  | 1.00E-67 | Floral homeotic protein APETALA2, putative [Ricinus communis] |
| miR172 | TC266497 | 1.0 | 16.32 | Cleavage | 4.00E-39 | unnamed protein product [Vitis vinifera] |
|  |  |  |  |  | 3.00E-38 | PREDICTED: floral homeotic protein APETALA 2 [Vitis vinifera] |
|  |  |  |  |  | 1.00E-36 | transcription factor APETALA2 [Citrus trifoliata] |
| miR172 | TC248423 | 1.0 | 15.72 | Cleavage | 3.00E-100 | PREDICTED: floral homeotic protein APETALA 2 [Vitis vinifera] |
|  |  |  |  |  | 3.00E-96 | unnamed protein product [Vitis vinifera] |
|  |  |  |  |  | 2.00E-90 | Floral homeotic protein APETALA2, putative [Ricinus communis] |
| miR172 | TC270554 | 1.0 | 17.68 | Cleavage | 9.00E-125 | PREDICTED: floral homeotic protein APETALA 2 [Vitis vinifera] |
|  |  |  |  |  | 1.00E-120 | unnamed protein product [Vitis vinifera] |
|  |  |  |  |  | 3.00E-114 | Floral homeotic protein APETALA2, putative [Ricinus communis] |
| miR172 | TC259821 | 1.0 | 18.53 | Cleavage | 7.00E-54 | Floral homeotic protein APETALA2, putative [Ricinus communis] |
|  |  |  |  |  | 2.00E-51 | PREDICTED: floral homeotic protein APETALA 2 [Vitis vinifera] |
|  |  |  |  |  | 2.00E-50 | unnamed protein product [Vitis vinifera] |
| miR172 | TC276428 | 1.0 | 22.67 | Cleavage | 5.70E-01 | PREDICTED: ethylene-responsive transcription factor RAP2-7-like [Vitis vinifera] |
|  |  |  |  |  | 5.90E-01 | unnamed protein product [Vitis vinifera] |
|  |  |  |  |  | 8.20E+00 | TOE1 [Arabidopsis thaliana] |
| miR172 | TC276199 | 1.0 | 18.26 | Cleavage | 1.00E-78 | AP2 domain-containing transcription factor [Populus trichocarpa] |
|  |  |  |  |  | 2.00E-78 | AP2 domain-containing transcription factor [Populus trichocarpa] |
|  |  |  |  |  | 3.00E-75 | PREDICTED: ethylene-responsive transcription factor RAP2-7-like [Glycine max] |
| miR172 | TC251459 | 2.0 | 18.04 | Cleavage | 1.00E-133 | PREDICTED: floral homeotic protein APETALA 2 [Vitis vinifera] |
|  |  |  |  |  | 6.00E-133 | unnamed protein product [Vitis vinifera] |
|  |  |  |  |  | 5.00E-123 | Transcription factor APETALA2 [Medicago truncatula] |
| miR172 | TC267470 | 2.0 | 9.01 | Cleavage | 2.00E-61 | unnamed protein product [Vitis vinifera] |
|  |  |  |  |  | 4.00E-61 | PREDICTED: ethylene-responsive transcription factor RAP2-7-like [Vitis vinifera] |
|  |  |  |  |  | 6.00E-55 | hypothetical protein VITISV_001337 [Vitis vinifera] |
| miR172 | TC251161 | 2.0 | 6.81 | Cleavage | 1.00E-123 | PREDICTED: ethylene-responsive transcription factor RAP2-7-like [Vitis vinifera] |
|  |  |  |  |  | 2.00E-121 | unnamed protein product [Vitis vinifera] |
|  |  |  |  |  | 5.00E-111 | Protein AINTEGUMENTA, putative [Ricinus communis] |
| miR172 | TC231199 | 3.0 | 18.34 | Translation | 5.00E-166 | PREDICTED: mitochondrial-processing peptidase subunit alpha [Vitis vinifera] |
|  |  |  |  |  | 1.00E-165 | PREDICTED: mitochondrial-processing peptidase subunit alpha-like [Glycine max] |
|  |  |  |  |  | 1.00E-165 | PREDICTED: mitochondrial-processing peptidase subunit alpha-like isoform 1 [Glycine max] |
| miR172 | TC243398 | 3.0 | 16.82 | Translation | 2.00E-129 | PREDICTED: mitochondrial-processing peptidase subunit alpha [Vitis vinifera] |
|  |  |  |  |  | 4.00E-127 | PREDICTED: mitochondrial-processing peptidase subunit alpha-like [Glycine max] |
|  |  |  |  |  | 5.00E-127 | PREDICTED: mitochondrial-processing peptidase subunit alpha-like isoform 1 [Glycine max] |
| miR172 | TC257610 | 3.0 | 13.98 | Cleavage | 2.00E-30 | unnamed protein product [Vitis vinifera] |
|  |  |  |  |  | 6.00E-30 | PREDICTED: histone deacetylase 19-like [Vitis vinifera] |
|  |  |  |  |  | 7.00E-29 | PREDICTED: histone deacetylase 19-like [Vitis vinifera] |
| miR172 | DT053268 | 3.0 | 13.41 | Translation |  | None |
| miR172 | TC253864 | 3.0 | 14.79 | Cleavage | 5.00E-49 | hypothetical protein ARALYDRAFT_355402 [Arabidopsis lyrata subsp. lyrata] |
|  |  |  |  |  | 8.00E-49 | conserved hypothetical protein [Ricinus communis] |
|  |  |  |  |  | 2.00E-48 | uncharacterized protein [Arabidopsis thaliana] |
| miR172 | BE054528 | 3.0 | 9.13 | Cleavage | 8.00E-72 | conserved hypothetical protein [Ricinus communis] |
|  |  |  |  |  | 9.00E-66 | hypothetical protein ARALYDRAFT_355402 [Arabidopsis lyrata subsp. lyrata] |
|  |  |  |  |  | 9.00E-65 | predicted protein [Populus trichocarpa] |
| miR172 | TC247951 | 3.0 | 16.47 | Cleavage | 1.00E-71 | conserved hypothetical protein [Ricinus communis] |
|  |  |  |  |  | 5.00E-65 | hypothetical protein ARALYDRAFT_355402 [Arabidopsis lyrata subsp. lyrata] |
|  |  |  |  |  | 7.00E-64 | uncharacterized protein [Arabidopsis thaliana] |
| miR172 | TC268375 | 3.0 | 22.89 | Cleavage | 2.00E-71 | PREDICTED: protein ODORANT1 [Vitis vinifera] |
|  |  |  |  |  | 3.00E-64 | predicted protein [Populus trichocarpa] |
|  |  |  |  |  | 4.00E-63 | ODORANT1 protein, putative [Ricinus communis] |
| miR2118 | EV485900 | 3.0 | 20.28 | Cleavage | 1.00E-132 | cinnamate-4-hydroxylase [Gossypium arboreum] |
|  |  |  |  |  | 1.00E-126 | cinnamate-4-hydroxylate-like [Hibiscus cannabinus] |
|  |  |  |  |  | 4.00E-122 | cinnamate-4-hydroxylase [Parthenocissus henryana] |
| miR2118 | ES805590 | 3.0 | 22.87 | Cleavage | 8.00E-62 | predicted protein [Populus trichocarpa] |
|  |  |  |  |  | 7.00E-56 | conserved hypothetical protein [Ricinus communis] |
|  |  |  |  |  | 5.00E-52 | PREDICTED: protein SUPPRESSOR OF GENE SILENCING 3-like [Vitis vinifera] |
| miR2118 | TC277739 | 3.0 | 22.66 | Cleavage | 0.00E+00 | PREDICTED: protein SUPPRESSOR OF GENE SILENCING 3-like [Vitis vinifera] |
|  |  |  |  |  | 0.00E+00 | conserved hypothetical protein [Ricinus communis] |
|  |  |  |  |  | 0.00E+00 | PREDICTED: protein SUPPRESSOR OF GENE SILENCING 3-like [Glycine max] |
| miR2118 | ES807202 | 2.5 | 24.02 | Translation | 7.00E-85 | NBS-type resistance protein [Gossypium barbadense] |
|  |  |  |  |  | 2.00E-84 | NBS-type resistance protein [Gossypium barbadense] |
|  |  |  |  |  | 3.00E-83 | NBS-type resistance protein [Gossypium barbadense] |
| miR2118 | TC235376 | 2.5 | 21.97 | Translation | 6.00E-146 | TMV resistance protein N, putative [Ricinus communis] |
|  |  |  |  |  | 8.00E-141 | tir-nbs-lrr resistance protein [Populus trichocarpa] |
|  |  |  |  |  | 5.00E-138 | HD domain class transcription factor [Malus x domestica] |
| miR2911 | CO107746 | 2.0 | 17.13 | Translation |  | None |
| miR2911 | TC258771 | 2.5 | 6.88 | Translation | 2.00E-119 | beta-tubulin [Theobroma cacao] |
|  |  |  |  |  | 2.00E-119 | unknown [Zea mays] |
|  |  |  |  |  | 2.00E-119 | beta-tubulin [Avena sativa] |
| miR2911 | CO114056 | 3.0 | 15.57 | Translation | 2.00E-36 | sucrose synthase 1 [Gossypium arboreum] |
|  |  |  |  |  | 1.00E-35 | sucrose synthase isoform D [Gossypium hirsutum] |
|  |  |  |  |  | 1.00E-34 | unknown [Medicago truncatula] |
| miR2947 | CO105636 | 3.0 | 19.58 | Cleavage | 5.00E-26 | hypothetical protein VITISV_008825 [Vitis vinifera] |
|  |  |  |  |  | 4.00E-25 | REDICTED: serine/threonine-protein phosphatase 7 long form homolog [Glycine max] |
|  |  |  |  |  | 4.00E-24 | serine/threonine-protein phosphatase 7-like protein [Arabidopsis thaliana] |
| miR2948-5p | TC255591 | 2.5 | 12.81 | Cleavage | 4.00E-05 | predicted protein [Hordeum vulgare subsp. vulgare] |
|  |  |  |  |  | 5.00E-05 | uncharacterized protein [Arabidopsis thaliana] |
|  |  |  |  |  | 5.00E-05 | hypothetical protein OsI_25459 [Oryza sativa Indica Group] |
| miR2948-5p | CO126449 | 2.5 | 6.12 | Cleavage |  | None |
| miR2948-5p | TC250962 | 2.5 | 16.50 | Cleavage | 3.00E-34 | conserved hypothetical protein [Ricinus communis] |
|  |  |  |  |  | 3.00E-34 | redicted protein [Populus trichocarpa] |
|  |  |  |  |  | 1.00E-33 | PREDICTED: uncharacterized protein LOC100261066 isoform 1 [Vitis vinifera] |
| miR2948-5p | BE053057 | 2.5 | 6.54 | Cleavage | 8.00E-12 | predicted protein [Populus trichocarpa] |
|  |  |  |  |  | 3.00E-11 | hypothetical protein VITISV_032245 [Vitis vinifera] |
|  |  |  |  |  | 2.00E-10 | predicted protein [Populus trichocarpa] |
| miR2948-5p | TC265202 | 2.5 | 8.94 | Cleavage | 1.00E-32 | hypothetical protein VITISV_017049 [Vitis vinifera] |
|  |  |  |  |  | 1.00E-32 | unknown [Glycine max] |
|  |  |  |  |  | 1.00E-31 | Phosphoglycerate mutase-like protein [Medicago truncatula] |
| miR2948-5p | AW725440 | 2.5 | 21.41 | Cleavage | 5.00E-06 | PREDICTED: tyrosine N-monooxygenase-like [Brachypodium distachyon] |
| miR2948-5p | EV497230 | 2.0 | 14.26 | Cleavage | 1.00E-22 | unknown [Populus trichocarpa] |
|  |  |  |  |  | 2.00E-22 | cytochrome P450 [Populus trichocarpa] |
|  |  |  |  |  | 2.00E-22 | cytochrome P450 [Populus trichocarpa] |
| miR2948-5p | TC233607 | 2.0 | 14.42 | Cleavage | 5.00E-98 | cytochrome P450 [Citrus sinensis] |
|  |  |  |  |  | 8.00E-95 | cytochrome P450 [Populus trichocarpa] |
|  |  |  |  |  | 1.00E-94 | cytochrome P450 [Populus trichocarpa] |
| miR2948-5p | TC231731 | 2.0 | 14.42 | Cleavage | 4.00E-110 | cytochrome P450 [Citrus sinensis] |
|  |  |  |  |  | 1.00E-108 | cytochrome P450 [Populus trichocarpa] |
|  |  |  |  |  | 4.00E-108 | cytochrome P450 [Populus trichocarpa] |
| miR2948-5p | DT467322 | 2.5 | 4.46 | Cleavage | 6.00E-09 | conserved hypothetical protein [Ricinus communis] |
|  |  |  |  |  | 1.00E-07 | predicted protein [Populus trichocarpa] |
|  |  |  |  |  | 6.00E-07 | hypothetical protein SORBIDRAFT_10g024955 [Sorghum bicolor] |
| miR2948-5p | DW238255 | 3.0 | 9.52 | Cleavage | 9.00E-15 | predicted protein [Populus trichocarpa] |
|  |  |  |  |  | 1.00E-12 | unnamed protein product [Vitis vinifera] |
|  |  |  |  |  | 7.00E-10 | unknown [Populus trichocarpa] |
| miR2948-5p | ES798923 | 3.0 | 2.97 | Cleavage | 9.00E-18 | predicted protein [Populus trichocarpa] |
|  |  |  |  |  | 2.00E-17 | glucose-methanol-choline (gmc) oxidoreductase, putative [Ricinus communis] |
|  |  |  |  |  | 8.00E-17 | PREDICTED: protein HOTHEAD isoform 2 [Vitis vinifera] |
| miR2948-5p | TC251973 | 2.5 | 11.19 | Cleavage | 7.00E-93 | cytochrome P450, putative [Ricinus communis] |
|  |  |  |  |  | 4.00E-92 | unnamed protein product [Vitis vinifera] |
|  |  |  |  |  | 4.00E-92 | PREDICTED: 2-methylbutanal oxime monooxygenase-like [Vitis vinifera] |
| miR2948-5p | ES808717 | 2.5 | 11.20 | Cleavage | 5.00E-107 | cytochrome P450 [Citrus sinensis] |
|  |  |  |  |  | 2.00E-106 | cytochrome P450 [Populus trichocarpa] |
|  |  |  |  |  | 2.00E-106 | cytochrome P450 [Populus trichocarpa] |
| miR2949 | TC244560 | 1.0 | 20.81 | Cleavage | 2.00E-05 | NAC domain protein [Medicago truncatula] |
|  |  |  |  |  | 3.00E-05 | ubiquitin-protein ligase, putative [Ricinus communis] > |
| miR2949 | TC273521 | 1.0 | 23.22 | Cleavage | 1.00E-06 | hypothetical protein SORBIDRAFT_09g030046 [Sorghum bicolor] |
| miR2949 | CO129605 | 2.0 | 11.42 | Cleavage | 3.00E-06 | PREDICTED: probable inactive receptor kinase RLK902-like [Vitis vinifera] |
|  |  |  |  |  | 1.00E-05 | PREDICTED: SPX domain-containing protein 5-like [Brachypodium distachyon] |
|  |  |  |  |  | 1.00E-05 | unnamed protein product [Vitis vinifera] |
| miR2949 | GE653651 | 2.5 | 23.82 | Translation | 2.00E+00 | hypothetical protein ZEAMMB73_378250 [Zea mays] |
|  |  |  |  |  | 2.00E+00 | hypothetical protein ZEAMMB73_378250 [Zea mays] |
|  |  |  |  |  | 2.00E+00 | NOL1/NOP2/sun family [Zea mays] |
| miR2949 | CO075720 | 2.5 | 15.64 | Cleavage | 7.00E-07 | unknown [Zea mays] |
| miR2949 | TC244353 | 3.0 | 20.61 | Cleavage | 7.00E-93 | uncharacterized protein LOC100305910 [Glycine max] |
|  |  |  |  |  | 2.00E-92 | uncharacterized protein LOC100527513 [Glycine max] |
|  |  |  |  |  | 2.00E-91 | conserved hypothetical protein [Ricinus communis] |
| miR2949 | TC279101 | 3.0 | 24.25 | Cleavage | 3.00E-90 | uncharacterized protein LOC100305910 [Glycine max] |
|  |  |  |  |  | 9.00E-90 | uncharacterized protein LOC100527513 [Glycine max] |
|  |  |  |  |  | 9.00E-90 | conserved hypothetical protein [Ricinus communis] |
| miR2949 | TC243432 | 2.5 | 12.60 | Cleavage | 2.00E-104 | predicted protein [Populus trichocarpa] |
|  |  |  |  |  | 3.00E-100 | PREDICTED: protein SAMHD1 homolog [Glycine max] |
|  |  |  |  |  | 7.00E-100 | metal-dependent phosphohydrolase HD domain-containing protein [Arabidopsis thaliana] |
| miR2950 | AW729887 | 3.0 | 22.78 | Cleavage | 6.00E-06 | hypothetical protein [Oryza sativa Japonica Group] |
|  |  |  |  |  | 1.00E-05 | hypothetical protein OsI_04918 [Oryza sativa Indica Group] |
|  |  |  |  |  | 2.00E-05 | hypothetical protein SORBIDRAFT_01g050163 [Sorghum bicolor] |
| miR2950 | BM358931 | 3.0 | 22.60 | Translation | 3.00E-39 | cyclopropane fatty acid synthase [Gossypium hirsutum] |
|  |  |  |  |  | 1.00E-38 | cyclopropane fatty acid synthase [Gossypium hirsutum] |
|  |  |  |  |  | 2.00E-34 | cyclopropane synthase [Sterculia foetida] |
| miR2950 | TC260975 | 2.5 | 18.75 | Cleavage | 2.00E-136 | PREDICTED: uncharacterized oxidoreductase ykwC-like [Vitis vinifera] |
|  |  |  |  |  | 4.00E-133 | predicted protein [Populus trichocarpa] |
|  |  |  |  |  | 4.00E-131 | 3-hydroxyisobutyrate dehydrogenase, putative [Ricinus communis] |
| miR2950 | AI727192 | 2.5 | 18.44 | Cleavage | 9.00E-78 | unnamed protein product [Vitis vinifera] |
|  |  |  |  |  | 2.00E-76 | PREDICTED: uncharacterized oxidoreductase ykwC-like [Vitis vinifera] |
|  |  |  |  |  | 2.00E-69 | predicted protein [Populus trichocarpa] |
| miR2950 | BF271544 | 2.5 | 15.56 | Cleavage | 1.00E-49 | unnamed protein product [Vitis vinifera] |
|  |  |  |  |  | 1.00E-48 | PREDICTED: uncharacterized oxidoreductase ykwC-like [Vitis vinifera] |
|  |  |  |  |  | 1.00E-41 | predicted protein [Populus trichocarpa] |
| miR2950 | TC236767 | 3.0 | 21.24 | Cleavage | 3.00E-54 | PREDICTED: uncharacterized protein LOC100807371 isoform 1 [Glycine max] |
|  |  |  |  |  | 1.00E-53 | unknown [Glycine max] |
|  |  |  |  |  | 3.00E-53 | PREDICTED: uncharacterized protein LOC100261483 [Vitis vinifera] |
| miR2950 | TC242859 | 2.5 | 11.14 | Cleavage | 0.00E+00 | predicted protein [Populus trichocarpa] |
|  |  |  |  |  | 0.00E+00 | predicted protein [Populus trichocarpa] |
|  |  |  |  |  | 0.00E+00 | PREDICTED: subtilisin-like protease-like [Glycine max] |
| miR2950 | TC272803 | 3.0 | 17.39 | Cleavage | 3.00E-38 | actin [Cicer arietinum] |
|  |  |  |  |  | 4.00E-38 | actin [Ipomoea nil] |
|  |  |  |  |  | 4.00E-38 | actin [Striga asiatica] |
| miR2950 | ES813827 | 3.0 | 16.31 | Cleavage | 8.00E-18 | homogentisate phytyltransferase [Linum usitatissimum] |
|  |  |  |  |  | 1.00E-16 | homogentisate phytyltransferase [Solanum tuberosum] |
|  |  |  |  |  | 1.00E-16 | predicted protein [Arabidopsis lyrata subsp. lyrata] |
| miR3476 | TC234003 | 3.0 | 22.77 | Cleavage | 0.00E+00 | putative phosphatidylinositol 4-phosphate 5-kinase [Nicotiana rustica] |
|  |  |  |  |  | 0.00E+00 | predicted protein [Populus trichocarpa] |
|  |  |  |  |  | 3.00E-180 | putative phosphatidylinositol-4-phosphate-5-kinase [Arabidopsis thaliana] |
| miR3476 | TC244560 | 3.0 | 21.69 | Cleavage | 2.00E-05 | NAC domain protein [Medicago truncatula] |
|  |  |  |  |  | 3.00E-05 | ubiquitin-protein ligase, putative [Ricinus communis] |
| miR3476 | TC273521 | 3.0 | 23.50 | Cleavage | 1.00E-06 | hypothetical protein SORBIDRAFT_09g030046 [Sorghum bicolor] |
| miR3476 | TC276560 | 3.0 | 13.09 | Cleavage | 5.00E-14 | Snakin-1 [Solanum tuberosum] |
|  |  |  |  |  | 1.00E-13 | snakin-1 [Solanum chacoense] |
|  |  |  |  |  | 1.00E-13 | snakin-1 [Solanum bulbocastanum] |
| miR3476 | TC262254 | 3.0 | 17.28 | Cleavage | 3.00E-25 | conserved hypothetical protein [Ricinus communis] |
|  |  |  |  |  | 1.00E-23 | predicted protein [Populus trichocarpa] |
|  |  |  |  |  | 1.00E-14 | uncharacterized protein LOC100527815 [Glycine max] |
| miR3476 | TC238923 | 3.0 | 14.74 | Cleavage | 2.00E-24 | conserved hypothetical protein [Ricinus communis] |
|  |  |  |  |  | 3.00E-22 | predicted protein [Populus trichocarpa] |
|  |  |  |  |  | 7.00E-14 | uncharacterized protein LOC100527815 [Glycine max] |
| miR3711-3p | ES807560 | 2.5 | 22.44 | Cleavage | 2.00E-121 | Membrane protein PB1A10.07c, putative [Ricinus communis] |
|  |  |  |  |  | 3.00E-118 | predicted protein [Populus trichocarpa] |
|  |  |  |  |  | 4.00E-118 | PREDICTED: probable serine incorporator [Vitis vinifera] |
| miR3711-3p | TC246491 | 3.0 | 19.15 | Cleavage | 2.00E-117 | conserved hypothetical protein [Ricinus communis] |
|  |  |  |  |  | 6.00E-115 | unknown [Populus trichocarpa] |
|  |  |  |  |  | 2.00E-111 | PREDICTED: uncharacterized protein LOC100807088 [Glycine max] |
| miR3711-3p | CO107431 | 3.0 | 16.28 | Cleavage | 5.00E-31 | PREDICTED: uncharacterized protein LOC100854194 [Vitis vinifera] |
|  |  |  |  |  | 5.00E-31 | unnamed protein product [Vitis vinifera] |
|  |  |  |  |  | 6.00E-28 | predicted protein [Populus trichocarpa] |
| miR3711-3p | CO132657 | 3.0 | 19.04 | Cleavage | 7.00E-51 | PREDICTED: uncharacterized protein LOC100854194 [Vitis vinifera] |
|  |  |  |  |  | 7.00E-51 | unnamed protein product [Vitis vinifera] |
|  |  |  |  |  | 7.00E-46 | kinesin motor family protein [Arabidopsis lyrata subsp. lyrata] |
| miR390 | TC275010 | 1.5 | 15.45 | Translation | 3.00E-15 | TAS3^a^ |
| miR390 | TC242082 | 1.5 | 15.04 | Translation | 1.40E+00 | predicted protein [Populus trichocarpa] |
| miR390 | TC277595 | 2.0 | 9.52 | Translation | 1.00E-11 | TAS3 ^a^ |
| miR390 | TC272586 | 2.0 | 10.81 | Translation | 6.00E-06 | TAS3 ^a^ |
| miR390 | CO075666 | 2.0 | 9.07 | Translation | 2.00E-12 | TAS3 ^a^ |
| miR390 | TC276618 | 2.5 | 18.73 | Cleavage | 2.00E-49 | unknown [Populus trichocarpa] |
|  |  |  |  |  | 6.00E-49 | AP-4 complex subunit sigma-1, putative [Ricinus communis] |
|  |  |  |  |  | 9.00E-49 | predicted protein [Populus trichocarpa] |
| miR390 | ES828745 | 2.0 | 10.52 | Translation | 5.00E-04 | TAS3b ^a^ |
| miR390 | TC240667 | 2.5 | 22.07 | Cleavage | 1.00E-71 | protein kinase domain-containing protein [Arabidopsis thaliana] |
|  |  |  |  |  | 5.00E-69 | kinase family protein [Arabidopsis lyrata subsp. lyrata] |
|  |  |  |  |  | 4.00E-67 | PREDICTED: serine/threonine-protein kinase At3g07070 [Vitis vinifera] |
| miR390 | CO115013 | 2.5 | 17.14 | Translation | 2.00E-119 | predicted protein [Populus trichocarpa] |
|  |  |  |  |  | 4.00E-118 | leucine-rich repeat protein kinase-like protein [Arabidopsis thaliana] >sp\|Q9LVP0.1\|Y5639_ARATH RecName: Full=Probable leucine-rich repeat receptor-like protein kinase At5g63930; Flags: Precursor >dbj\|BAA96896.1\| receptor-like protein kinase [Arabidopsis thaliana] >gb\|ACN59405.1\| leucine-rich repeat receptor-like protein kinase [Arabidopsis thaliana] >gb\|AED97817.1\| leucine-rich repeat protein kinase-like protein [Arabidopsis thaliana] |
|  |  |  |  |  | 2.00E-117 | hypothetical protein ARALYDRAFT_496585 [Arabidopsis lyrata subsp. lyrata] |
| miR390 | ES818319 | 2.5 | 20.25 | Translation | 2.00E-124 | predicted protein [Populus trichocarpa] |
|  |  |  |  |  | 3.00E-123 | Leucine-rich repeat receptor protein kinase EXS precursor, putative[Ricinus communis] |
|  |  |  |  |  | 7.00E-122 | predicted protein [Populus trichocarpa] |
| miR390 | TC271473 | 3.0 | 8.14 | Translation | 1.00E-12 | TAS3 ^a^ |
| miR390 | DW502659 | 2.5 | 10.97 | Cleavage | 5.00E-05 | F7F22.5 [Arabidopsis thaliana] |
|  |  |  |  |  | 6.00E-05 | PREDICTED: phospholipase D p1-like [Vitis vinifera] |
|  |  |  |  |  | 6.00E-05 | unnamed protein product [Vitis vinifera] |
| miR390 | DW503626 | 2.5 | 13.94 | Translation | 3.00E-15 | TAS3 ^a^ |
| miR390 | TC266640 | 2.5 | 22.52 | Cleavage | 2.00E-105 | BRASSINOSTEROID INSENSITIVE 1 precursor, putative [Ricinus communis] |
|  |  |  |  |  | 5.00E-99 | PREDICTED: probable leucine-rich repeat receptor-like protein kinase At2g33170-like [Glycine max] |
|  |  |  |  |  | 9.00E-99 | PREDICTED: probable leucine-rich repeat receptor-like protein kinase At2g33170-like [Glycine max] |
| miR390 | TC250886 | 2.5 | 23.57 | Cleavage | 0.00E+00 | PREDICTED: DEAD-box ATP-dependent RNA helicase 21-like [Vitis vinifera] |
|  |  |  |  |  | 0.00E+00 | PREDICTED: DEAD-box ATP-dependent RNA helicase 21-like [Vitis vinifera] |
|  |  |  |  |  | 0.00E+00 | dead box ATP-dependent RNA helicase, putative [Ricinus communis] |
| miR390 | TC236002 | 3.0 | 12.98 | Cleavage | 2.00E-122 | unnamed protein product [Vitis vinifera] |
|  |  |  |  |  | 5.00E-121 | nucleic acid binding protein, putative [Ricinus communis] |
|  |  |  |  |  | 3.00E-120 | predicted protein [Populus trichocarpa] |
| miR390 | TC266622 | 3.0 | 16.74 | Translation | 6.00E-87 | predicted protein [Populus trichocarpa] |
|  |  |  |  |  | 1.00E-71 | conserved hypothetical protein [Ricinus communis] |
|  |  |  |  |  | 1.00E-68 | hypothetical protein VITISV_029171 [Vitis vinifera] |
| miR393-3p | TC262066 | 2.5 | 22.22 | Cleavage |  | None |
| miR395 | TC235159 | 3.0 | 17.67 | Cleavage | 7.00E-109 | predicted protein [Populus trichocarpa] |
|  |  |  |  |  | 1.00E-106 | unknown [Medicago truncatula] |
|  |  |  |  |  | 2.00E-106 | sulfate adenylyltransferase, putative [Ricinus communis] |
| miR395 | TC276235 | 3.0 | 17.67 | Cleavage | 8.00E-47 | predicted protein [Populus trichocarpa] |
|  |  |  |  |  | 3.00E-45 | ATP sulfurylase [Glycine max] |
|  |  |  |  |  | 3.00E-44 | ATP sulfurylase [Camellia sinensis] |
| miR395 | DT463008 | 3.0 | 18.44 | Cleavage | 7.00E-73 | predicted protein [Populus trichocarpa] |
|  |  |  |  |  | 9.00E-70 | predicted protein [Populus trichocarpa] |
|  |  |  |  |  | 2.00E-65 | sulfate adenylyltransferase [Solanum tuberosum] |
| miR395 | TC245081 | 3.0 | 17.36 | Cleavage | 8.00E-77 | predicted protein [Populus trichocarpa] |
|  |  |  |  |  | 4.00E-74 | predicted protein [Populus trichocarpa] |
|  |  |  |  |  | 1.00E-68 | ulfate adenylyltransferase [Solanum tuberosum] |
| miR395 | BG443152 | 3.0 | 21.07 | Cleavage | 1.00E-37 | predicted protein [Populus trichocarpa] |
|  |  |  |  |  | 7.00E-37 | unknown [Medicago truncatula] |
|  |  |  |  |  | 9.00E-37 | PREDICTED: uncharacterized protein LOC100267262 [Vitis vinifera] |
| miR395 | TC233433 | 3.0 | 15.79 | Cleavage | 4.00E-148 | transferase, putative [Ricinus communis] |
|  |  |  |  |  | 4.00E-147 | PREDICTED: cellulose synthase-like protein G2-like [Vitis vinifera] |
|  |  |  |  |  | 2.00E-145 | PREDICTED: cellulose synthase A catalytic subunit 4 [UDP-forming]-like, partial [Vitis vinifera] |
| miR395 | TC238357 | 2.5 | 19.35 | Cleavage | 3.00E-105 | predicted protein [Populus trichocarpa] |
|  |  |  |  |  | 3.00E-102 | predicted protein [Populus trichocarpa] |
|  |  |  |  |  | 8.00E-96 | PREDICTED: uncharacterized protein LOC100248615 [Vitis vinifera] |
| miR396-3p | TC275676 | 2.0 | 14.95 | Cleavage | 4.00E-06 | PREDICTED: uncharacterized protein LOC100527871 [Glycine max] |
|  |  |  |  |  | 4.00E-06 | unknown [Glycine max] |
|  |  |  |  |  | 4.00E-05 | hypothetical protein SORBIDRAFT_01g008700 [Sorghum bicolor] |
| miR396-3p | TC238611 | 2.5 | 9.02 | Cleavage | 4.00E-06 | PREDICTED: uncharacterized protein LOC100527871 [Glycine max] |
|  |  |  |  |  | 4.00E-06 | unknown [Glycine max] |
|  |  |  |  |  | 4.00E-05 | hypothetical protein SORBIDRAFT_01g008700 [Sorghum bicolor] |
| miR396-3p | ES850361 | 2.5 | 20.41 | Cleavage | 2.00E-24 | heat shock protein binding protein, putative [Ricinus communis] |
|  |  |  |  |  | 8.00E-22 | unknown [Glycine max] |
|  |  |  |  |  | 2.00E-21 | PREDICTED: uncharacterized protein LOC100779992 [Glycine max] |
| miR396-3p | TC280552 | 2.5 | 24.79 | Cleavage | 5.00E-116 | shaggy-like kinase [Ricinus communis] |
|  |  |  |  |  | 3.00E-115 | PREDICTED: shaggy-related protein kinase eta-like [Vitis vinifera] |
|  |  |  |  |  | 4.00E-115 | predicted protein [Populus trichocarpa] |
| miR396-3p | DT048786 | 3.0 | 12.61 | Cleavage | 8.00E-07 | unknown [Medicago truncatula] |
|  |  |  |  |  | 4.00E-06 | uncharacterized protein LOC100812783 [Glycine max] |
|  |  |  |  |  | 9.00E-06 | 26S protease regulatory subunit, putative [Ricinus communis] |
| miR396-3p | ES834637 | 3.0 | 21.31 | Cleavage | 3.00E-93 | flavonoid 3',5'-hydroxylase [Gossypium hirsutum] |
|  |  |  |  |  | 2.00E-92 | flavonoid 3'5'-hydroxylase [Gossypium hirsutum] |
|  |  |  |  |  | 9.00E-92 | flavonoid 3'5'-hydroxylase [Gossypium hirsutum] |
| miR396-5p | TC271568 | 1.5 | 14.71 | Cleavage | 1.00E-131 | hypothetical protein VITISV_012188 [Vitis vinifera] |
|  |  |  |  |  | 3.00E-126 | PREDICTED: transcription regulatory protein SNF2-like [Glycine max] |
|  |  |  |  |  | 3.00E-126 | PREDICTED: transcription regulatory protein SNF2-like [Glycine max] |
| miR396-5p | TC275676 | 2.0 | 22.94 | Cleavage | 4.00E-06 | PREDICTED: uncharacterized protein LOC100527871 [Glycine max] |
|  |  |  |  |  | 4.00E-06 | unknown [Glycine max] |
|  |  |  |  |  | 4.00E-05 | hypothetical protein SORBIDRAFT_01g008700 [Sorghum bicolor] |
| miR396-5p | TC246647 | 2.0 | 16.48 | Cleavage | 2.00E-75 | chromatin remodeling complex subunit [Populus trichocarpa] |
|  |  |  |  |  | 9.00E-75 | ATP binding protein, putative [Ricinus communis] |
|  |  |  |  |  | 1.00E-74 | hypothetical protein VITISV_012188 [Vitis vinifera] |
| miR396-5p | TC254882 | 2.0 | 16.48 | Cleavage | 9.00E-57 | chromatin remodeling complex subunit [Populus trichocarpa] |
|  |  |  |  |  | 2.00E-54 | ATP binding protein, putative [Ricinus communis] |
|  |  |  |  |  | 3.00E-53 | chromatin remodeling complex subunit [Populus trichocarpa] |
| miR396-5p | DR453736 | 2.5 | 21.43 | Cleavage | 1.00E-101 | ATP-dependent CLP protease [Solanum tuberosum] |
|  |  |  |  |  | 3.00E-99 | Os04g0397100 [Oryza sativa Japonica Group] |
|  |  |  |  |  | 1.00E-98 | ATP-dependent clp protease [Cucumis melo subsp. melo] |
| miR396-5p | ES832212 | 2.5 | 16.09 | Translation | 2.00E-90 | conserved hypothetical protein [Ricinus communis] |
|  |  |  |  |  | 6.00E-87 | predicted protein [Populus trichocarpa] |
|  |  |  |  |  | 2.00E-83 | hypothetical protein [Capsella rubella] |
| miR396-5p | AI055349 | 3.0 | 19.69 | Cleavage | 1.00E-66 | calcium ion binding protein, putative [Ricinus communis] |
|  |  |  |  |  | 3.00E-65 | predicted protein [Populus trichocarpa] |
|  |  |  |  |  | 9.00E-64 | predicted protein [Populus trichocarpa] |
| miR396-5p | TC240832 | 3.0 | 18.43 | Cleavage | 1.00E-118 | calcium ion binding protein, putative [Ricinus communis] |
|  |  |  |  |  | 1.00E-115 | predicted protein [Populus trichocarpa] |
|  |  |  |  |  | 1.00E-114 | predicted protein [Populus trichocarpa] |
| miR396-5p | TC251689 | 3.0 | 18.43 | Cleavage | 1.00E-145 | calcium ion binding protein, putative [Ricinus communis] |
|  |  |  |  |  | 3.00E-142 | predicted protein [Populus trichocarpa] |
|  |  |  |  |  | 8.00E-142 | predicted protein [Populus trichocarpa] |
| miR396-5p | TC276400 | 3.0 | 18.43 | Cleavage | 4.00E-145 | calcium ion binding protein, putative [Ricinus communis] |
|  |  |  |  |  | 1.00E-139 | predicted protein [Populus trichocarpa] |
|  |  |  |  |  | 1.00E-138 | PREDICTED: uncharacterized protein LOC100248610 [Vitis vinifera] |
| miR396-5p | TC231394 | 3.0 | 18.43 | Cleavage | 7.00E-151 | calcium ion binding protein, putative [Ricinus communis] |
|  |  |  |  |  | 1.00E-145 | predicted protein [Populus trichocarpa] |
|  |  |  |  |  | 7.00E-140 | PREDICTED: uncharacterized protein LOC100248610 [Vitis vinifera] |
| miR396-5p | BF274373 | 2.0 | 13.55 | Cleavage | 8.00E-47 | conserved hypothetical protein [Ricinus communis] |
|  |  |  |  |  | 2.00E-46 | predicted protein [Populus trichocarpa] |
|  |  |  |  |  | 5.00E-46 | GRL2 [Medicago truncatula] |
| miR396-5p | TC245894 | 3.0 | 10.86 | Cleavage | 4.00E-122 | hypothetical protein VITISV_035768 [Vitis vinifera] |
|  |  |  |  |  | 2.00E-109 | GTP binding protein, putative [Ricinus communis] |
|  |  |  |  |  | 2.00E-106 | unnamed protein product [Vitis vinifera] |
| miR396-5p | ES814410 | 3.0 | 12.29 | Cleavage | 4.00E-35 | skip-2, putative [Ricinus communis] |
|  |  |  |  |  | 2.00E-34 | f-box family protein [Populus trichocarpa] |
|  |  |  |  |  | 4.00E-34 | F-box protein [Medicago truncatula] |
| miR396-5p | DR455380 | 2.0 | 14.34 | Cleavage | 8.00E-40 | PREDICTED: uncharacterized protein LOC100250422 [Vitis vinifera] |
|  |  |  |  |  | 1.00E-39 | unnamed protein product [Vitis vinifera] |
|  |  |  |  |  | 2.00E-37 | predicted protein [Populus trichocarpa] |
| miR396-5p | TC264786 | 2.5 | 19.34 | Cleavage | 1.00E-77 | conserved hypothetical protein [Ricinus communis] |
|  |  |  |  |  | 2.00E-75 | PREDICTED: uncharacterized protein LOC100261588 isoform 1 [Vitis vinifera] |
|  |  |  |  |  | 2.00E-75 | unnamed protein product [Vitis vinifera] |
| miR397 | TC256899 | 0.0 | 13.82 | Cleavage | 3.00E-154 | laccase 1b [Populus trichocarpa] |
|  |  |  |  |  | 5.00E-153 | laccase 1a [Populus trichocarpa] |
|  |  |  |  |  | 8.00E-152 | laccase [Populus trichocarpa] |
| miR397 | TC267962 | 0.0 | 14.66 | Cleavage | 3.00E-154 | laccase 1b [Populus trichocarpa] |
|  |  |  |  |  | 5.00E-153 | laccase 1a [Populus trichocarpa] |
|  |  |  |  |  | 8.00E-152 | laccase [Populus trichocarpa] |
| miR397 | TC235401 | 1.0 | 15.82 | Cleavage | 0.00E+00 | laccase 1b [Populus trichocarpa] |
|  |  |  |  |  | 0.00E+00 | PREDICTED: laccase-4 [Vitis vinifera] |
|  |  |  |  |  | 0.00E+00 | laccase 1a [Populus trichocarpa] |
| miR397 | TC279559 | 1.0 | 16.14 | Cleavage | 0.00E+00 | laccase 1b [Populus trichocarpa] |
|  |  |  |  |  | 0.00E+00 | laccase 1a [Populus trichocarpa] |
|  |  |  |  |  | 0.00E+00 | laccase [Populus trichocarpa] |
| miR397 | TC242566 | 1.5 | 12.03 | Cleavage | 1.00E-140 | predicted protein [Populus trichocarpa] |
|  |  |  |  |  | 2.00E-140 | predicted protein [Populus trichocarpa] |
|  |  |  |  |  | 9.00E-132 | laccase 1b [Populus trichocarpa] |
| miR397 | TC262631 | 1.5 | 11.56 | Cleavage | 0.00E+00 | PREDICTED: laccase-4-like [Glycine max] |
|  |  |  |  |  | 0.00E+00 | PREDICTED: laccase-4-like [Glycine max] |
|  |  |  |  |  | 0.00E+00 | Laccase 1a [Medicago truncatula] |
| miR397 | DW224951 | 1.0 | 9.62 | Cleavage | 5.00E-143 | laccase, putative [Ricinus communis] |
|  |  |  |  |  | 1.00E-142 | laccase 110b [Populus trichocarpa] |
|  |  |  |  |  | 6.00E-142 | unnamed protein product [Vitis vinifera] |
| miR397 | TC237050 | 1.0 | 15.35 | Cleavage | 2.00E-171 | laccase, putative [Ricinus communis] |
|  |  |  |  |  | 2.00E-170 | PREDICTED: laccase-17-like [Glycine max] |
|  |  |  |  |  | 2.00E-170 | PREDICTED: laccase-17-like [Glycine max] |
| miR397 | TC230926 | 2.5 | 10.96 | Cleavage | 0.00E+00 | laccase, putative [Ricinus communis] |
|  |  |  |  |  | 0.00E+00 | predicted protein [Populus trichocarpa] |
|  |  |  |  |  | 0.00E+00 | predicted protein [Populus trichocarpa] |
| miR397 | TC233218 | 2.5 | 11.57 | Cleavage | 5.00E-155 | unnamed protein product [Vitis vinifera] |
|  |  |  |  |  | 1.00E-154 | PREDICTED: laccase-17-like [Vitis vinifera] |
|  |  |  |  |  | 2.00E-153 | laccase, putative [Ricinus communis] |
| miR397 | TC272856 | 2.5 | 7.02 | Translation | 9.00E-27 | PREDICTED: nephrocystin-3-like [Glycine max] |
|  |  |  |  |  | 1.00E-26 | PREDICTED: nephrocystin-3-like [Glycine max] |
|  |  |  |  |  | 2.00E-26 | kinesin light chain, putative [Ricinus communis] |
| miR397 | ES811692 | 2.5 | 16.89 | Cleavage | 3.00E-65 | unnamed protein product [Vitis vinifera] |
|  |  |  |  |  | 3.00E-65 | unknown [Medicago truncatula] |
|  |  |  |  |  | 4.00E-65 | PREDICTED: probable protein phosphatase 2C 38-like [Vitis vinifera] |
| miR397 | CO113170 | 3.0 | 19.70 | Cleavage | 5.00E-79 | conserved hypothetical protein [Ricinus communis] |
|  |  |  |  |  | 5.00E-76 | PREDICTED: uncharacterized protein LOC100242158 [Vitis vinifera] |
|  |  |  |  |  | 1.00E-75 | PREDICTED: uncharacterized protein LOC100813397 [Glycine max] |
| miR397 | CO093654 | 3.0 | 23.03 | Cleavage | 3.00E-51 | PREDICTED: uncharacterized protein LOC100266486 [Vitis vinifera] >emb\|CBI26411.3\| unnamed protein product [Vitis vinifera] |
|  |  |  |  |  | 4.00E-51 | conserved hypothetical protein [Ricinus communis] |
|  |  |  |  |  | 5.00E-51 | PREDICTED: uncharacterized protein LOC100813397 [Glycine max] |
| miR397 | TC234114 | 3.0 | 22.04 | Cleavage | 3.00E-101 | conserved hypothetical protein [Ricinus communis] |
|  |  |  |  |  | 3.00E-99 | PREDICTED: uncharacterized protein LOC100813397 [Glycine max] |
|  |  |  |  |  | 4.00E-99 | PREDICTED: uncharacterized protein LOC100781733 [Glycine max] |
| miR397 | TC234005 | 3.0 | 22.04 | Cleavage | 2.00E-150 | conserved hypothetical protein [Ricinus communis] |
|  |  |  |  |  | 3.00E-149 | PREDICTED: uncharacterized protein LOC100266486 [Vitis vinifera] |
|  |  |  |  |  | 4.00E-148 | PREDICTED: uncharacterized protein LOC100813397 [Glycine max] |
| miR397 | TC275490 | 3.0 | 16.08 | Cleavage | 3.00E-55 | steroid binding protein, putative [Ricinus communis] |
|  |  |  |  |  | 1.00E-53 | PREDICTED: uncharacterized protein LOC100252604 [Vitis vinifera] |
|  |  |  |  |  | 1.00E-53 | PREDICTED: probable steroid-binding protein 3-like [Glycine max] |
| miR397 | TC278175 | 3.0 | 16.00 | Cleavage | 1.00E-57 | steroid binding protein, putative [Ricinus communis] |
|  |  |  |  |  | 4.00E-56 | PREDICTED: uncharacterized protein LOC100252604 [Vitis vinifera] |
|  |  |  |  |  | 9.00E-56 | PREDICTED: probable steroid-binding protein 3-like [Glycine max] |
| miR397 | TC251826 | 3.0 | 15.67 | Cleavage | 2.00E-57 | steroid binding protein, putative [Ricinus communis] |
|  |  |  |  |  | 8.00E-56 | PREDICTED: uncharacterized protein LOC100252604 [Vitis vinifera] |
|  |  |  |  |  | 2.00E-55 | PREDICTED: probable steroid-binding protein 3-like [Glycine max] |
| miR397 | TC265556 | 3.0 | 15.96 | Cleavage | 4.00E-57 | steroid binding protein, putative [Ricinus communis] |
|  |  |  |  |  | 2.00E-55 | PREDICTED: uncharacterized protein LOC100252604 [Vitis vinifera] |
|  |  |  |  |  | 4.00E-55 | PREDICTED: probable steroid-binding protein 3-like [Glycine max] |
| miR397 | TC236053 | 3.0 | 14.37 | Translation |  |  |
|  |  |  |  |  |  |  |
|  |  |  |  |  |  |  |
| miR397 | EX165180 | 3.0 | 20.54 | Cleavage | 5.00E-83 | Glucan endo-1,3-beta-glucosidase precursor, putative [Ricinus communis] |
|  |  |  |  |  | 2.00E-81 | predicted protein [Populus trichocarpa] |
|  |  |  |  |  | 8.00E-80 | predicted protein [Populus trichocarpa] |
| miR397 | TC232202 | 3.0 | 18.03 | Cleavage | 0.00E+00 | Glucan endo-1,3-beta-glucosidase precursor, putative [Ricinus communis] |
|  |  |  |  |  | 0.00E+00 | predicted protein [Populus trichocarpa] |
|  |  |  |  |  | 0.00E+00 | predicted protein [Populus trichocarpa] |
| miR397 | ES798360 | 3.0 | 16.35 | Cleavage | 7.00E-131 | Glucan endo-1,3-beta-glucosidase precursor, putative [Ricinus communis] |
|  |  |  |  |  | 3.00E-126 | predicted protein [Populus trichocarpa] |
|  |  |  |  |  | 5.00E-126 | predicted protein [Populus trichocarpa] |
| miR399 | TC236756 | 1.5 | 10.69 | Translation | 1.00E-148 | DNA binding protein, putative [Ricinus communis] |
|  |  |  |  |  | 4.00E-146 | predicted protein [Populus trichocarpa] |
|  |  |  |  |  | 1.00E-145 | MYB transcription factor [Hevea brasiliensis] |
| miR399 | TC270621 | 3.0 | 16.15 | Cleavage | 4.00E-113 | conserved hypothetical protein [Ricinus communis] |
|  |  |  |  |  | 1.00E-111 | predicted protein [Populus trichocarpa] |
|  |  |  |  |  | 7.00E-106 | ethylene-responsive element-binding protein [Citrus sinensis] |
| miR399 | TC239324 | 2.5 | 16.52 | Translation | 7.00E-123 | DNA binding protein, putative [Ricinus communis] |
|  |  |  |  |  | 3.00E-120 | MYB transcription factor [Hevea brasiliensis] |
|  |  |  |  |  | 5.00E-120 | unknown [Hevea brasiliensis] |
| miR399 | TC264546 | 3.0 | 22.67 | Cleavage | 1.00E-86 | unnamed protein product [Vitis vinifera] |
|  |  |  |  |  | 3.00E-86 | PREDICTED: SWI/SNF complex subunit SWI3C-like [Vitis vinifera] |
|  |  |  |  |  | 9.00E-80 | chromatin remodeling complex subunit [Populus trichocarpa] |
| miR479 | ES845096 | 3.0 | 16.98 | Translation | 8.00E-49 | structural molecule, putative [Ricinus communis] |
|  |  |  |  |  | 6.00E-46 | unknown [Medicago truncatula] |
|  |  |  |  |  | 9.00E-46 | PREDICTED: probable plastid-lipid-associated protein 8, chloroplastic [Vitis vinifera] |
| miR479 | TC232019 | 3.0 | 15.64 | Translation | 9.00E-74 | structural molecule, putative [Ricinus communis] |
|  |  |  |  |  | 5.00E-72 | predicted protein [Populus trichocarpa] |
|  |  |  |  |  | 2.00E-71 | hypothetical protein ARALYDRAFT_910032 [Arabidopsis lyrata subsp. lyrata] |
| miR479 | DN780454 | 3.0 | 22.59 | Cleavage | 8.00E-25 | PREDICTED: scarecrow-like protein 22-like [Vitis vinifera] |
|  |  |  |  |  | 1.00E-24 | conserved hypothetical protein [Ricinus communis] |
|  |  |  |  |  | 1.00E-23 | GRAS family transcription factor [Populus trichocarpa] |
| miR482 | TC248482 | 2.5 | 17.28 | Cleavage | 1.00E-06 | glycine-rich protein [Gossypium hirsutum] |
|  |  |  |  |  | 2.00E-06 | hypothetical protein VITISV_031043 [Vitis vinifera] |
|  |  |  |  |  | 6.00E-06 | mannose-binding lectin [Amaryllis minuta] |
| miR482 | BG445413 | 3.0 | 1.56 | Cleavage | 4.00E-65 | predicted protein [Populus trichocarpa] |
|  |  |  |  |  | 6.00E-65 | predicted protein [Populus trichocarpa] |
|  |  |  |  |  | 6.00E-63 | PREDICTED: aspartic proteinase nepenthesin-2-like [Glycine max] |
| miR482 | NP673173 | 2.5 | 15.06 | Translation | 2.00E-125 | NBS-type resistance protein [Gossypium barbadense] |
|  |  |  |  |  | 4.00E-95 | NBS-LRR resistance protein-like protein [Gossypium hirsutum] |
|  |  |  |  |  | 2.00E-94 | NBS-type resistance protein [Gossypium barbadense] |
| miR535 | CO118493 | 2.0 | 7.81 | Cleavage | 9.00E-58 | conserved hypothetical protein [Ricinus communis] > |
|  |  |  |  |  | 5.00E-47 | presenilin family protein [Arabidopsis lyrata subsp. lyrata] |
|  |  |  |  |  | 8.00E-46 | PREDICTED: presenilin-like protein At1g08700-like [Vitis vinifera] |
| miR535 | DW516134 | 3.0 | 13.51 | Cleavage | 2.00E-13 | PREDICTED: abscisic acid receptor PYL8-like [Glycine max] |
|  |  |  |  |  | 2.00E-13 | hypothetical protein VITISV_029498 [Vitis vinifera] |
|  |  |  |  |  | 3.00E-13 | unnamed protein product [Vitis vinifera] |
| miR535 | CO127161 | 2.5 | 24.96 | Cleavage | 2.00E-114 | predicted protein [Populus trichocarpa] |
|  |  |  |  |  | 8.00E-114 | PREDICTED: bifunctional dihydroflavonol 4-reductase/flavanone 4-reductase-like [Vitis vinifera] |
|  |  |  |  |  | 1.00E-113 | unnamed protein product [Vitis vinifera] |
| miR535 | TC274268 | 3.0 | 6.75 | Cleavage | 1.00E-06 | predicted protein [Populus trichocarpa] |
|  |  |  |  |  | 1.00E-05 | predicted protein [Populus trichocarpa] |
|  |  |  |  |  | 2.00E-05 | hypothetical protein VITISV_033593 [Vitis vinifera] |
| miR535 | TC274268 | 3.0 | 10.05 | Cleavage | 1.00E-06 | predicted protein [Populus trichocarpa] |
|  |  |  |  |  | 1.00E-05 | predicted protein [Populus trichocarpa] |
|  |  |  |  |  | 2.00E-05 | hypothetical protein VITISV_033593 [Vitis vinifera] |
| miR535 | TC274268 | 3.0 | 6.79 | Cleavage | 1.00E-06 | predicted protein [Populus trichocarpa] |
|  |  |  |  |  | 1.00E-05 | predicted protein [Populus trichocarpa] |
|  |  |  |  |  | 2.00E-05 | hypothetical protein VITISV_033593 [Vitis vinifera] |
| miR6300 | TC265189 | 2.5 | 14.38 | Cleavage | 6.70E+00 | PREDICTED: protein ALWAYS EARLY 2-like isoform 2 [Brachypodium distachyon] |
|  |  |  |  |  | 7.20E+00 | PREDICTED: protein ALWAYS EARLY 2-like isoform 1 [Brachypodium distachyon] |
| miR6300 | TC270292 | 2.5 | 15.62 | Cleavage | 5.00E-126 | PREDICTED: ubiquitin carboxyl-terminal hydrolase 10-like isoform 1 [Glycine max] |
|  |  |  |  |  | 5.00E-126 | PREDICTED: ubiquitin carboxyl-terminal hydrolase 10-like isoform 1 [Glycine max] |
|  |  |  |  |  | 2.00E-125 | PREDICTED: ubiquitin carboxyl-terminal hydrolase 10-like isoform 2 [Glycine max] |
| miR6300 | TC276640 | 2.5 | 11.44 | Cleavage | 9.30E-02 | PREDICTED: uncharacterized protein LOC100817542 [Glycine max] |
|  |  |  |  |  | 1.50E-01 | membrane associated ring finger 1,8, putative [Ricinus communis] |
|  |  |  |  |  | 4.70E-01 | predicted protein [Populus trichocarpa] |
| miR6300 | TC239091 | 2.5 | 19.11 | Cleavage | 2.00E-126 | membrane associated ring finger 1,8, putative [Ricinus communis] |
|  |  |  |  |  | 2.00E-123 | predicted protein [Populus trichocarpa] |
|  |  |  |  |  | 3.00E-122 | PREDICTED: uncharacterized protein LOC100779674 [Glycine max] |
| miR6300 | TC233996 | 2.5 | 21.53 | Cleavage | 9.00E-97 | membrane associated ring finger 1,8, putative [Ricinus communis] |
|  |  |  |  |  | 7.00E-94 | predicted protein [Populus trichocarpa] |
|  |  |  |  |  | 2.00E-92 | PREDICTED: uncharacterized protein LOC100779674 [Glycine max] |
| miR6300 | DT053793 | 3.0 | 10.76 | Translation |  | None |
| miR6300 | TC276564 | 3.0 | 19.74 | Cleavage | 2.00E-62 | PREDICTED: uncharacterized protein LOC100260890 [Vitis vinifera] |
|  |  |  |  |  | 2.00E-47 | predicted protein [Populus trichocarpa] |
|  |  |  |  |  | 3.00E-47 | conserved hypothetical protein [Ricinus communis] |
| miR6300 | BE054437 | 3.0 | 14.57 | Cleavage |  | None |
| miR6478 | DV850090 | 2.5 | 21.61 | Cleavage |  | None |
| miR6478 | TC259543 | 3.0 | 14.04 | Translation | 4.00E-91 | calmodulin binding protein, putative [Ricinus communis] |
|  |  |  |  |  | 3.00E-90 | PREDICTED: uncharacterized protein LOC100249281 isoform 1 [Vitis vinifera] |
|  |  |  |  |  | 5.00E-89 | hypothetical protein VITISV_002643 [Vitis vinifera] |
| miR7492 | DW509241 | 3.0 | 11.59 | Cleavage | 2.00E-43 | unnamed protein product [Vitis vinifera] |
|  |  |  |  |  | 2.00E-43 | PREDICTED: uncharacterized protein LOC100246247 [Vitis vinifera] |
|  |  |  |  |  | 4.00E-43 | Protein NEDD1 [Medicago truncatula] |
| miR7492 | TC271910 | 3.0 | 21.27 | Translation | 1.00E-37 | unnamed protein product [Thellungiella halophila] |
|  |  |  |  |  | 2.00E-37 | endo-1,3-1,4-beta-d-glucanase, putative [Ricinus communis] |
|  |  |  |  |  | 4.00E-37 | predicted protein [Populus trichocarpa] |
| miR7495 | ES816423 | 1.5 | 23.25 | Cleavage |  | None |
| miR7495 | TC267154 | 1.5 | 22.32 | Cleavage | 2.00E-05 | Os05g0241000 [Oryza sativa Japonica Group] |
|  |  |  |  |  | 5.00E-05 | hypothetical protein VITISV_021893 [Vitis vinifera] |
|  |  |  |  |  | 6.00E-05 | conserved hypothetical protein [Ricinus communis] |
| miR7495 | CO491311 | 3.0 | 17.33 | Cleavage | 3.00E-11 | PREDICTED: probable serine/threonine-protein kinase At1g54610 [Vitis vinifera] |
|  |  |  |  |  | 3.00E-08 | Serine/threonine-protein kinase cdk9, putative [Ricinus communis] |
|  |  |  |  |  | 5.00E-08 | PREDICTED: probable serine/threonine-protein kinase At1g54610-like [Glycine max] |
| miR7495 | ES817139 | 2.5 | 16.83 | Translation | 6.00E-12 | uncharacterized protein LOC100811099 [Glycine max] |
|  |  |  |  |  | 1.00E-11 | predicted protein [Populus trichocarpa] |
|  |  |  |  |  | 2.00E-11 | unknown [Lotus japonicus] |
| miR7495 | TC271035 | 3.0 | 13.07 | Translation | 1.30E-02 | hypothetical protein VITISV_004870 [Vitis vinifera] |
|  |  |  |  |  | 1.30E-02 | PREDICTED: UDP-glucose flavonoid 3-O-glucosyltransferase 7 [Vitis vinifera] |
|  |  |  |  |  | 3.00E-02 | PREDICTED: UDP-glycosyltransferase 73B3-like [Glycine max] |
| miR7495 | TC233919 | 3.0 | 21.03 | Cleavage | 2.00E-77 | protein binding protein, putative [Ricinus communis] |
|  |  |  |  |  | 7.00E-77 | predicted protein [Populus trichocarpa] |
|  |  |  |  |  | 9.00E-77 | predicted protein [Populus trichocarpa] |
| miR7495 | TC246961 | 3.0 | 10.59 | Cleavage | 2.00E-73 | Myosin heavy chain, striated muscle, putative [Ricinus communis] |
|  |  |  |  |  | 2.00E-69 | predicted protein [Populus trichocarpa] |
|  |  |  |  |  | 5.00E-69 | predicted protein [Populus trichocarpa] |
| miR7495 | TC245220 | 3.0 | 10.42 | Cleavage | 2.00E-108 | putative monosaccharide transporter [Vitis vinifera] |
|  |  |  |  |  | 5.00E-108 | PREDICTED: plastid hexose transporter [Vitis vinifera] |
|  |  |  |  |  | 6.00E-106 | Plastidic glucose transporter 4 [Arabidopsis thaliana] |
| miR7504b | TC268902 | 2.0 | 18.33 | Translation | 1.00E-166 | PREDICTED: proline-rich receptor-like protein kinase PERK1-like isoform 1 [Vitis vinifera] |
|  |  |  |  |  | 1.00E-165 | PREDICTED: proline-rich receptor-like protein kinase PERK1-like |
|  |  |  |  |  | 6.00E-165 | isoform 2 [Vitis vinifera] |
| miR7504b | TC263274 | 2.0 | 21.52 | Translation | 3.00E-114 | PERK1-like protein kinase [Nicotiana tabacum] |
|  |  |  |  |  | 7.00E-112 | ATP binding protein, putative [Ricinus communis] |
|  |  |  |  |  | 4.00E-110 | PREDICTED: proline-rich receptor-like protein kinase PERK1-like isoform 1 [Vitis vinifera] |
| miR7504b | TC236311 | 2.0 | 18.99 | Translation | 0.00E+00 | ATP binding protein, putative [Ricinus communis] |
|  |  |  |  |  | 0.00E+00 | putative receptor protein kinase PERK1 [Glycine max] |
|  |  |  |  |  | 0.00E+00 | PREDICTED: proline-rich receptor-like protein kinase PERK1-like isoform 1 [Vitis vinifera] |
| miR7504b | TC258355 | 2.0 | 21.72 | Translation | 0.00E+00 | PREDICTED: proline-rich receptor-like protein kinase PERK1-like isoform 2 [Vitis vinifera] |
|  |  |  |  |  | 0.00E+00 | putative receptor protein kinase PERK1 [Glycine max] |
|  |  |  |  |  | 0.00E+00 | PREDICTED: proline-rich receptor-like protein kinase PERK1-like isoform 1 [Vitis vinifera] |
| miR7504b | DW224547 | 2.5 | 14.19 | Cleavage | 5.00E-44 | unnamed protein product [Vitis vinifera] |
|  |  |  |  |  | 1.00E-43 | PREDICTED: uncharacterized protein LOC100248912 [Vitis vinifera] |
|  |  |  |  |  | 6.00E-43 | PREDICTED: uncharacterized protein LOC100783884 [Glycine max] |
| miR7504b | DR459393 | 3.0 | 17.45 | Translation |  | None |
| miR7504b | AI725951 | 2.5 | 20.27 | Translation | 6.00E-35 | putative receptor protein kinase PERK1 [Glycine max] |
|  |  |  |  |  | 1.00E-34 | unknown [Zea mays] |
|  |  |  |  |  | 1.00E-34 | Cysteine-rich receptor-like protein kinase [Medicago truncatula] |
| miR7504b | TC244831 | 2.5 | 24.66 | Translation | 1.00E-111 | ATP binding protein, putative [Ricinus communis] |
|  |  |  |  |  | 3.00E-110 | PREDICTED: proline-rich receptor-like protein kinase PERK1-like isoform 1 [Vitis vinifera] |
|  |  |  |  |  | 2.00E-109 | putative receptor protein kinase PERK1 [Glycine max] |
| miR7504b | TC247634 | 2.5 | 20.37 | Translation | 3.00E-168 | putative receptor protein kinase PERK1 [Glycine max] |
|  |  |  |  |  | 5.00E-167 | PREDICTED: proline-rich receptor-like protein kinase PERK1-like isoform 2 [Vitis vinifera] |
|  |  |  |  |  | 3.00E-166 | ATP binding protein, putative [Ricinus communis] |
| miR7504b | TC248090 | 1.5 | 10.73 | Cleavage | 7.00E-132 | conserved hypothetical protein [Ricinus communis] |
|  |  |  |  |  | 4.00E-128 | predicted protein [Populus trichocarpa] |
|  |  |  |  |  | 2.00E-127 | predicted protein [Populus trichocarpa] |
| miR7504b | ES811286 | 2.0 | 11.33 | Cleavage | 2.00E-47 | PREDICTED: uncharacterized protein At1g51745-like [Glycine max] |
|  |  |  |  |  | 4.00E-46 | PREDICTED: uncharacterized protein At1g51745-like [Glycine max] |
|  |  |  |  |  | 2.00E-45 | hypothetical protein VITISV_024170 [Vitis vinifera] |
| miR7505 | AI727415 | 1.5 | 17.56 | Cleavage | 2.00E-61 | PREDICTED: hypothetical protein [Vitis vinifera] |
|  |  |  |  |  | 3.00E-60 | PREDICTED: hypothetical protein [Vitis vinifera] |
|  |  |  |  |  | 5.00E-60 | PREDICTED: hypothetical protein [Vitis vinifera] |
| miR7505 | CO111817 | 1.5 | 21.28 | Cleavage | 6.00E-79 | predicted protein [Populus trichocarpa] |
|  |  |  |  |  | 1.00E-78 | predicted protein [Populus trichocarpa] |
|  |  |  |  |  | 2.00E-78 | hypothetical protein VITISV_031896 [Vitis vinifera] |
| miR7505 | TC266681 | 1.5 | 23.91 | Cleavage | 4.00E-90 | predicted protein [Populus trichocarpa] |
|  |  |  |  |  | 8.00E-90 | predicted protein [Populus trichocarpa] |
|  |  |  |  |  | 1.00E-88 | PREDICTED: hypothetical protein [Vitis vinifera] |
| miR7505 | ES801805 | 1.5 | 15.69 | Translation | 2.00E-55 | pentatricopeptide repeat-containing protein, putative [Ricinus communis] |
|  |  |  |  |  | 1.00E-52 | predicted protein [Populus trichocarpa] |
|  |  |  |  |  | 2.00E-52 | predicted protein [Populus trichocarpa] |
| miR7505 | TC269108 | 2.5 | 14.60 | Translation | 4.00E-83 | predicted protein [Populus trichocarpa] |
|  |  |  |  |  | 3.00E-81 | predicted protein [Populus trichocarpa] |
|  |  |  |  |  | 4.00E-81 | predicted protein [Populus trichocarpa] |
| miR7505 | TC250123 | 3.0 | 17.67 | Cleavage | 2.00E-59 | PREDICTED: hypothetical protein isoform 1 [Vitis vinifera] |
|  |  |  |  |  | 2.00E-59 | PREDICTED: hypothetical protein isoform 2 [Vitis vinifera] |
|  |  |  |  |  | 2.00E-59 | hypothetical protein VITISV_020207 [Vitis vinifera] |
| miR7505 | TC246633 | 3.0 | 10.77 | Cleavage | 6.00E-148 | LRR receptor-like protein kinase [Nicotiana tabacum] |
|  |  |  |  |  | 5.00E-145 | PREDICTED: hypothetical protein [Vitis vinifera] |
|  |  |  |  |  | 2.00E-144 | PREDICTED: hypothetical protein [Vitis vinifera] |
| miR7505 | TC235469 | 3.0 | 10.97 | Cleavage | 0.00E+00 | receptor protein kinase, putative [Ricinus communis] |
|  |  |  |  |  | 0.00E+00 | PREDICTED: hypothetical protein [Vitis vinifera] |
|  |  |  |  |  | 0.00E+00 | LRR receptor-like protein kinase [Nicotiana tabacum] |
| miR7505 | TC249160 | 3.0 | 12.61 | Cleavage | 7.00E-175 | LRR receptor-like protein kinase [Nicotiana tabacum] |
|  |  |  |  |  | 9.00E-173 | PREDICTED: hypothetical protein [Vitis vinifera] |
|  |  |  |  |  | 2.00E-172 | hypothetical protein ARALYDRAFT_484098 [Arabidopsis lyrata subsp. lyrata] |
| miR7505 | TC237037 | 3.0 | 12.61 | Cleavage | 9.00E-162 | PREDICTED: hypothetical protein [Vitis vinifera] |
|  |  |  |  |  | 1.00E-158 | LRR receptor-like protein kinase [Nicotiana tabacum] |
|  |  |  |  |  | 2.00E-158 | receptor protein kinase, putative [Ricinus communis] |
| miR7505 | ES845683 | 3.0 | 18.15 | Cleavage | 5.00E-05 | unnamed protein product [Vitis vinifera] |
|  |  |  |  |  | 5.00E-05 | predicted protein [Populus trichocarpa] |
|  |  |  |  |  | 5.00E-05 | PREDICTED: hypothetical protein [Vitis vinifera] |
| miR7505 | CO104492 | 3.0 | 17.86 | Translation | 3.00E-36 | predicted protein [Populus trichocarpa] |
|  |  |  |  |  | 3.00E-36 | unknown [Populus trichocarpa] |
|  |  |  |  |  | 2.00E-35 | unnamed protein product [Vitis vinifera] |
| miR7505 | ES838952 | 3.0 | 20.40 | Translation | 2.00E-57 | DNA binding/zinc ion binding protein [Gossypium hirsutum] |
|  |  |  |  |  | 5.00E-41 | predicted protein [Populus trichocarpa] |
|  |  |  |  |  | 7.00E-41 | predicted protein [Populus trichocarpa] |
| miR7505 | ES811669 | 3.0 | 20.64 | Translation | 2.00E-66 | PREDICTED: hypothetical protein [Vitis vinifera] |
|  |  |  |  |  | 2.00E-66 | hypothetical protein VITISV_023407 [Vitis vinifera] |
|  |  |  |  |  | 3.00E-57 | predicted protein [Populus trichocarpa] |
| miR7508 | TC268751 | 3.0 | 19.53 | Cleavage | 2.00E-73 | predicted protein [Populus trichocarpa] |
|  |  |  |  |  | 2.00E-73 | predicted protein [Populus trichocarpa] |
|  |  |  |  |  | 3.00E-73 | big map kinase/bmk, putative [Ricinus communis] |
| miR7508 | TC232866 | 2.5 | 12.77 | Cleavage | 5.00E-10 | mitogen-activated protein kinase 2 [Glycine max] |
|  |  |  |  |  | 3.00E-09 | hypothetical protein VITISV_009157 [Vitis vinifera] |
|  |  |  |  |  | 5.00E-09 | unnamed protein product [Vitis vinifera] |
| miR7508 | TC232029 | 2.5 | 5.82 | Cleavage | 7.00E-28 | unnamed protein product [Vitis vinifera] |
|  |  |  |  |  | 7.00E-28 | putative spermine synthase [Solanum lycopersicum] |
|  |  |  |  |  | 1.00E-27 | unknown [Glycine max] |
| miR7508 | TC248543 | 2.5 | 1.57 | Cleavage |  | none |
| miR7508 | TC234748 | 3.0 | 21.96 | Cleavage | 2.00E-105 | Spermine synthase [Arabidopsis thaliana] |
|  |  |  |  |  | 1.00E-102 | PREDICTED: hypothetical protein [Vitis vinifera] |
|  |  |  |  |  | 5.00E-97 | 3-beta-hydroxy-delta5-steroid dehydrogenase, putative [Ricinus communis]. |
| miR7508 | TC256667 | 3.0 | 21.65 | Cleavage | 7.00E-92 | predicted protein [Populus trichocarpa] |
|  |  |  |  |  | 3.00E-89 | PREDICTED: hypothetical protein [Vitis vinifera] |
|  |  |  |  |  | 4.00E-84 | 3-beta-hydroxy-delta5-steroid dehydrogenase, putative [Ricinus communis]. |
| miR7508 | DR453793 | 3.0 | 5.28 | Cleavage | 1.00E-06 | predicted protein [Populus trichocarpa] |
|  |  |  |  |  | 2.00E-06 | ERS type ethylene receptor [Ziziphus jujuba] |
|  |  |  |  |  | 2.00E-06 | ethylene response sensor [Brassica oleracea] |
| miR7508 | BQ414952 | 3.0 | 0.02 | Cleavage | 5.00E-06 | ERS1 [Mangifera indica] |
| miR7508 | TC234481 | 2.5 | 13.30 | Cleavage | 2.00E-41 | hypothetical protein VITISV_013884 [Vitis vinifera] |
|  |  |  |  |  | 9.00E-31 | conserved hypothetical protein [Ricinus communis] |
|  |  |  |  |  | 4.00E-27 | predicted protein [Populus trichocarpa] |
| miR7508 | ES794114 | 2.5 | 19.24 | Cleavage | 2.00E-149 | predicted protein [Populus trichocarpa] |
|  |  |  |  |  | 2.00E-149 | annexin [Gossypium hirsutum] |
|  |  |  |  |  | 2.00E-149 | Chain A, Crystal Structure Of Calcium-Bound Cotton Annexin Gh1. |
| miR7508 | DW496569 | 2.5 | 5.97 | Cleavage | 8.00E-42 | Chain A, Annexin Gh1 From Cotton. |
|  |  |  |  |  | 4.00E-40 | hypothetical protein VITISV_001638 [Vitis vinifera] |
|  |  |  |  |  | 7.00E-40 | conserved hypothetical protein [Ricinus communis] |
| miR7508 | EX168376 | 3.0 | 17.72 | Cleavage | 1.00E-48 | predicted protein [Populus trichocarpa] |
|  |  |  |  |  | 1.00E-47 | predicted protein [Populus trichocarpa] |
|  |  |  |  |  | 5.00E-46 | Ocs element-binding factor, putative [Ricinus communis] |
| miR7508 | ES814095 | 2.5 | 6.11 | Cleavage | 2.00E-42 | PREDICTED: hypothetical protein [Vitis vinifera] |
|  |  |  |  |  | 1.00E-39 | hypothetical protein VITISV_001638 [Vitis vinifera] |
|  |  |  |  |  | 7.00E-39 | predicted protein [Populus trichocarpa] |
| miR7508 | DT460941 | 3.0 | 12.02 | Cleavage |  | none |
| miR7508 | TC248911 | 3.0 | 22.84 | Cleavage | 0.00E+00 | predicted protein [Populus trichocarpa] |
|  |  |  |  |  | 0.00E+00 | predicted protein [Populus trichocarpa] |
|  |  |  |  |  | 0.00E+00 | PREDICTED: hypothetical protein [Vitis vinifera] |
| miR7508 | TC263854 | 2.5 | 14.88 | Cleavage | 3.00E-143 | conserved hypothetical protein [Ricinus communis] |
|  |  |  |  |  | 1.00E-127 | predicted protein [Populus trichocarpa] |
|  |  |  |  |  | 2.00E-125 | PREDICTED: hypothetical protein [Vitis vinifera] |
| miR7508 | TC251247 | 3.0 | 6.57 | Cleavage | 4.00E-43 | unnamed protein product [Vitis vinifera] |
|  |  |  |  |  | 9.00E-41 | conserved hypothetical protein [Ricinus communis] |
|  |  |  |  |  | 3.00E-40 | unknown [Populus trichocarpa x Populus deltoides] |
| miR7513 | ES829912 | 1.5 | 11.08 | Cleavage | 2.00E-35 | predicted protein [Populus trichocarpa] |
|  |  |  |  |  | 3.00E-35 | unnamed protein product [Vitis vinifera] |
|  |  |  |  |  | 4.00E-35 | transmembrane protein 14, putative [Ricinus communis] |
| miR7513 | ES827820 | 1.5 | 11.08 | Cleavage | 3.00E-35 | predicted protein [Populus trichocarpa] |
|  |  |  |  |  | 6.00E-35 | unnamed protein product [Vitis vinifera] |
|  |  |  |  |  | 7.00E-35 | transmembrane protein 14, putative [Ricinus communis] |
| miR7513 | TC246279 | 2.0 | 17.32 | Cleavage | 0.00E+00 | PREDICTED: hypothetical protein [Vitis vinifera] |
|  |  |  |  |  | 0.00E+00 | predicted protein [Populus trichocarpa] |
|  |  |  |  |  | 0.00E+00 | predicted protein [Populus trichocarpa] |
| miR7513 | TC252347 | 3.0 | 24.33 | Cleavage | 4.00E-55 | unnamed protein product [Vitis vinifera] |
|  |  |  |  |  | 3.00E-53 | PREDICTED: hypothetical protein [Vitis vinifera] |
|  |  |  |  |  | 6.00E-53 | predicted protein [Populus trichocarpa] |
| miR7513 | DR455287 | 3.0 | 16.29 | Cleavage | 2.00E-48 | PREDICTED: hypothetical protein [Vitis vinifera] |
|  |  |  |  |  | 4.00E-46 | predicted protein [Populus trichocarpa] |
|  |  |  |  |  | 2.00E-45 | hypothetical protein RCOM_1596950 [Ricinus communis] |
| miR7513 | DR457218 | 3.0 | 7.37 | Cleavage | 3.00E-49 | receptor serine-threonine protein kinase, putative [Ricinus communis]. |
|  |  |  |  |  | 8.00E-47 | receptor serine-threonine protein kinase, putative [Ricinus communis]. |
|  |  |  |  |  | 1.00E-46 | avrPphB susceptible 1 [Arabidopsis lyrata] |
| miR7513 | ES830552 | 3.0 | 19.68 | Cleavage | 3.00E-27 | predicted protein [Populus trichocarpa] |
|  |  |  |  |  | 2.00E-26 | protein with unknown function [Ricinus communis] |
|  |  |  |  |  | 8.00E-25 | unnamed protein product [Vitis vinifera] |
| miR7513 | TC279787 | 3.0 | 20.43 | Cleavage | 3.00E-27 | predicted protein [Populus trichocarpa] |
|  |  |  |  |  | 2.00E-26 | protein with unknown function [Ricinus communis] |
|  |  |  |  |  | 8.00E-25 | unnamed protein product [Vitis vinifera] |
| miR894 | TC250145 | 2.5 | 23.07 | Cleavage | 1.00E-22 | predicted protein [Populus trichocarpa] |
|  |  |  |  |  | 2.00E-22 | predicted protein [Populus trichocarpa] |
|  |  |  |  |  | 4.00E-22 | predicted protein [Populus trichocarpa] |
| miR894 | TC275041 | 2.5 | 14.91 | Cleavage | 3.00E-39 | PREDICTED: uncharacterized protein LOC100249562 isoform 1 [Vitis vinifera] |
|  |  |  |  |  | 2.00E-36 | conserved hypothetical protein [Ricinus communis] |
|  |  |  |  |  | 7.00E-36 | predicted protein [Populus trichocarpa] |
| miR894 | TC238643 | 2.5 | 19.27 | Cleavage | 7.00E-56 | PREDICTED: uncharacterized protein LOC100249562 isoform 1 [Vitis vinifera] |
|  |  |  |  |  | 2.00E-52 | predicted protein [Populus trichocarpa] |
|  |  |  |  |  | 6.00E-52 | unknown [Populus trichocarpa] |
| miR894 | TC257473 | 3.0 | 21.82 | Cleavage | 2.00E-13 | hypothetical protein MTR_027s0011 [Medicago truncatula] |
|  |  |  |  |  | 2.00E-09 | uncharacterized protein [Arabidopsis thaliana] |
|  |  |  |  |  | 8.00E-08 | hypothetical protein ARALYDRAFT_481141 [Arabidopsis lyrata subsp. lyrata] |

a: Annotation based on BLASTn of the TAIR database
