# Supplementary material for: Genome-wide analysis of small RNAs reveals eight fiber elongation-related and 257 novel microRNAs in elongating cotton fiber cells
Source: BMC Genomics. 2013 Sep 17;14:629. doi: 10.1186/1471-2164-14-629 (PMC3849097; doi:10.1186/1471-2164-14-629)
Supplement: Additional file 12: Figure S7 — GhSPL9, a target of GhmiR156/157, may positively regulate anthocyanin synthesis in cotton fiber. (A) Quantitative RT-PCR analysis of GhDFR, GhANS, and GhF3H at different fiber developmental stages. Error bars indicate the ± SD of three replicates. (B) The anthocyanin content in the cotton fiber at different development stages. Error bars represent the SD. [file 1471-2164-14-629-S12.docx]

**Additional Figure S7:**

**GhSPL9, a target of GhmiR156/157, may positively regulate anthocyanin synthesis in cotton fiber.** (A) Quantitative RT-PCR analysis of *GhDFR*, *GhANS*, and *GhF3H* at different fiber developmental stages. Error bars indicate the ± SD of three replicates. (B) The anthocyanin content in the cotton fiber at different development stages. Error bars represent the ± SD.

**
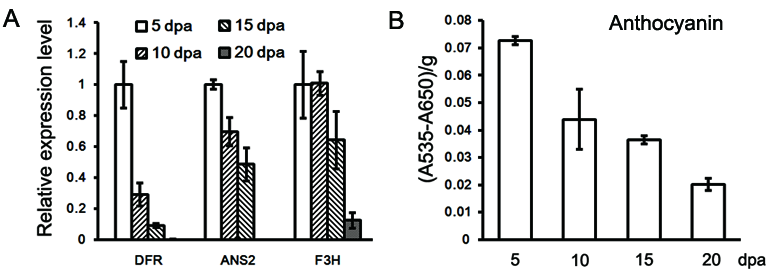
**
