# Supplementary material for: Genome-wide analysis of small RNAs reveals eight fiber elongation-related and 257 novel microRNAs in elongating cotton fiber cells
Source: BMC Genomics. 2013 Sep 17;14:629. doi: 10.1186/1471-2164-14-629 (PMC3849097; doi:10.1186/1471-2164-14-629)

**Additional Figure S8:**

**Quantitative RT-PCR analysis of *LRR-RLK* in the elongating fibers of *G. hirsutum* and *G. arboreum*.** Error bars indicate the ± SD of three replicates.


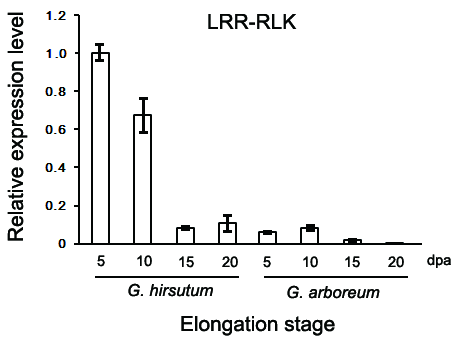

Supplement: Additional file 13: Figure S8 — Quantitative RT-PCR analysis of LRR-RLK in the elongating fibers of G. hirsutum and G. arboretum. [file 1471-2164-14-629-S13.docx]
