# Supplementary material for: Genome-wide analysis of small RNAs reveals eight fiber elongation-related and 257 novel microRNAs in elongating cotton fiber cells
Source: BMC Genomics. 2013 Sep 17;14:629. doi: 10.1186/1471-2164-14-629 (PMC3849097; doi:10.1186/1471-2164-14-629)
Supplement: Additional file 14: Table S6 — Normalized abundances of miRNAs and tasiRNA in fibers and seeds at 15 dpa. [file 1471-2164-14-629-S14.docx]

**Additional Table S6:**

**Normalized abundances of miRNAs and tasiRNAs in fibers and seeds at 15 dpa**

| Name | Abundance (TPTM)^a^ | | Relative to seeds^b^ | | Type^c^ |
| --- | --- | --- | --- | --- | --- |
|  | Seeds | Fibers |  |  |  |
| GhmiR156/157 | 62349 | 92994 | 49.20% |  | A |
| tasiR-ARF | 33 | 162 | 390.90% |  | B |
| GhmiR167 | 280854 | 214214 | 23.70% |  | B |
| GhmiR165/166 | 23855 | 65860 | 176.10% |  | B |
| GhmiRnE | 44 | 130 | 195.50% |  | B |
| GhmiRnC | 306 | 516 | 68.60% |  | B |
| GhmiR396 | 226 | 934 | 313.30% |  | B |
| GhmiR7505 | 36 | 89 | 147.20% |  | B |
| GhmiR168 | 3029 | 2665 | 12.20% |  | C |

a: RPTM, reads per ten million.

b: The black arrowheads indicate tendencies consistent with those in the rapid elongation stage (10 and 15 dpa) of the cotton fiber, and white arrowheads indicate tendencies that were not consistent.

c: A, B, and C represent the three types of expression patterns, as shown in Figure 2A; type A represents the miRNAs that gradually increased in abundance from 5-20 dpa, B represents miRNAs that mainly increased in abundance during the rapid elongation stage (10 or 15 dpa), and C represents the miRNAs that gradually decreased in abundance from 5-20 dpa.
