# Supplementary material for: Genome-wide analysis of small RNAs reveals eight fiber elongation-related and 257 novel microRNAs in elongating cotton fiber cells
Source: BMC Genomics. 2013 Sep 17;14:629. doi: 10.1186/1471-2164-14-629 (PMC3849097; doi:10.1186/1471-2164-14-629)

**Additional Figure S9:**

**RNA integrity of samples from 5, 10, 15, and 20 dpa fibers and 15 dpa seeds.** Right: Electropherograms of the samples, as generated by the Agilent 2100 Bioanalyzer. The x-axis indicates the size of the nucleic acid, and the y-axis indicates the fluorescence. The red and black arrowheads indicate the 28S and 18S ribosomal RNA peaks, respectively. The RIN and 28S to 18S ratio are also shown in the image. RIN: RNA integrity number; FU: Fluorescence units.
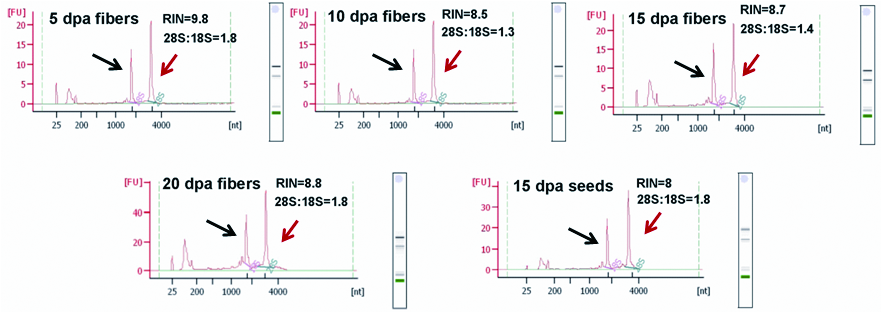

Supplement: Additional file 15: Figure S9 — RNA integrity of samples from 5, 10, 15 and 20 dpa fibers and 15 dpa seeds. Right: Electropherograms of the samples, as generated by the Agilent 2100 Bioanalyzer. The x-axis indicates the size of the nucleic acid, and the y-axis indicates the fluorescence. The red and black arrowheads indicate the 28S and 18S ribosomal RNA peaks, respectively. The RIN and 28S to 18S ratio are also shown in the image. RIN: RNA integrity number; FU: Fluorescence units. [file 1471-2164-14-629-S15.docx]
