# Supplementary material for: Genome-wide analysis of small RNAs reveals eight fiber elongation-related and 257 novel microRNAs in elongating cotton fiber cells
Source: BMC Genomics. 2013 Sep 17;14:629. doi: 10.1186/1471-2164-14-629 (PMC3849097; doi:10.1186/1471-2164-14-629)
Supplement: Additional file 16: Table S7 — The primers used in this study. [file 1471-2164-14-629-S16.docx]

**Additional Table S7:**

**The primers used in this study**

| **Primers for 5′ RACE mapping of GhmiRNA cleavage sites** | | |
| --- | --- | --- |
| TC272934 outer primer | GATGGGAAAGAGAAATGGGACGAA | |
| TC272934 inner primer | TGCTGCTCAAGTTCCATGTGTTGCAC | |
| TC259543 outer primer | CAACTCTCTTACCACACAAAATGC | |
| TC259543 inner primer | AATCCCTTCCTTTTTAATTGCCTCAC | |
| TC241907 outer primer | ATTCTTTGACCTGGGGTTGTGGGG | |
| TC241907 inner primer | CTAAAAGGCTCTGCCCGTTGCTCGCC | |
| TC236756 outer primer | AAAGAATCACGGCTATATCCAGGG | |
| TC236756 inner primer | TTCTCTGTATTGCTTGATGACATCCC | |
| TC279526 outer primer | GCGGCATGCTTGGACCATTT[1] | |
| TC279526 inner primer | TCCACAGCTCGGCAATCACG[1] | |
| TC246633 outer primer | GTTGAATAGATGCCGACTAAGGGT | |
| TC246633 inner primer | ATGGCGGTGACGAACCTTAGTAAGAA | |
| TC229767 outer primer | ACCTTTTTCG TAAAATCTCG TCTA | |
| TC229767 inner primer | GGATGGGTAG AAGAAGTTGT GATGCT | |
| TC251689 outer primer | GCACCTATTGGAGGTAAACTGGCA | |
| TC251689 inner primer | CGTTACGATCTTTCGGGACATTCTCT | |
| **Primers for quantitative real-time PCR** | | |
| TC272934(SPL9) forward | | GAGAATGCTTCACGGGAGT |
| TC272934 (SPL9) reverse | | TTCGGTGTTCATCATGTCG |
| TC241907(ARF8) forward | | GCTCGTTAGACATCTCCAGGTTCA |
| TC241907(ARF8) reverse | | GGGTCGTCTCCAAGGAGAAGAATA |
| TC279526(GHHB8) forward | | TTTCTTTCAAAGGCCACTGGAACT |
| TC279526(GHHB8) reverse | | GCCGATCTTTAAGGATTTCAGCAA |
| TC259543(CaMBP) forward | | CCTCAAATTTTCAATGTCACAAGG |
| TC259543(CaMBP) reverse | | TACAGCACAATAGGGAATCCAAGC |
| TC246633(LRR-RLK) forward | | TACTCATACACCCTCATCATTCCGA |
| TC246633(LRR-RLK) reverse | | TTTTCACAGCAATCTTTGTCCCATC |
| TC247863(DFR) forward | | TGCACTTGGATGATCTCTGC |
| TC247863(DFR) reverse | | TCCGACAAACATGTCTTCCA |
| TC269484(ANS) forward | | AAGTGGGTGACCGCTAAATG |
| TC269484(ANS) reverse | | AGGAGGGAACAGTGGAGGTT |
| GU062185.1(F3H) forward | | TTCCAAGCCGTCATCAAAG |
| GU062185.1(F3H) reverse | | TTCCACTCGTTAGGGTCTCG |
| TC251671(ARF4) forward | | GAAATTGATCCTTCTGCTTCTCTC |
| TC251671(ARF4) reverse | | CAAGACCTTCGGAAACCTATTAGA |
| TC251689(CIBP) forward | | TTATGAAGTGGAGATGTTGTCCGT |
| TC251689(CIBP) reverse | | GGTTGTTCCTTGAGATTTTGAGCC |
| TC229767 (ACCD) forward | | AATAAAAGACTAAACACAACTC |
| TC229767 (ACCD) reverse | | GCCAAAAGGACAACATACAGGA |
| *UBQ10* (TC235531) forward | | CCAGAAGGAATCCACTTTGC[2] |
| *UBQ10* (TC235531) reverse | | CCAGCTCACATCAGCATACG[2] |
| **Primer for mature miRNA** | | |
| miR156-Forward | | GGCGGttgacagaagataga |
| miR167-Forward | | TCGCGtgaagctgccagcat |
| miR168-Forward | | GCGGCGGtcgcttggtgcaggt |
| U6-F | | TACAGAGAAGATTAGCATGGCCCC |
| U6-R | | GGACCATTTCTCGATTTGTGCGTG |
| Universal reverse primer | | GTGCAGGGTCCGAGGT |
| miR156-stem-loop RT primer | | GTCGTATCCAGTGCAGGGTCCGAGGTATTCGCACTGGATACGACgtgctc |
| miR167-stem-loop RT primer | | GTCGTATCCAGTGCAGGGTCCGAGGTATTCGCACTGGATACGACgagatc |
| miR168-stem-loop RT primer | | GTCGTATCCAGTGCAGGGTCCGAGGTATTCGCACTGGATACGACttcccg |

**References:**

1. Pang M, Woodward AW, Agarwal V, Guan X, Ha M, Ramachandran V, Chen X, Triplett BA, Stelly DM, Chen ZJ: **Genome-wide analysis reveals rapid and dynamic changes in miRNA and siRNA sequence and expression during ovule and fiber development in allotetraploid cotton (Gossypium hirsutum L.)**. *Genome Biol* 2009, **10**(11):R122.

2. Walford SA, Wu Y, Llewellyn DJ, Dennis ES: **GhMYB25-like: a key factor in early cotton fibre development**. *Plant J* 2011, **65**(5):785-797.
